# Charting New Territory: Systematic Evaluation of the Drug Potential of *N*‑Trifluoromethyl Amides, Ureas & Carbamates

**DOI:** 10.1021/acs.jmedchem.6c01299

**Published:** 2026-07-03

**Authors:** Jacopo Garonzi, Gina Wycich, Stefanie Zich, Shakiba Vahdat, Beatriz Carvalho, Guillaume Benoit, Vijay K. Ahuja, Claire Le Manach, Hyeonglim Seo, Lionel E. Cheruzel, Christian Gampe, Xingyu Jiang, Franziska Schoenebeck, Stefan Schiesser

**Affiliations:** † BioPharma Chemistry, Discovery Sciences, BioPharmaceuticals R&D, 468087 AstraZeneca, Pepparedsleden 1, Mölndal 43183, Sweden; ‡ Department of Chemistry and Molecular Biology, Göteborgs Universitet, Medicinaregatan 7B, Göteborg 41390, Sweden; § Institute of Organic Chemistry, 9165 RWTH Aachen University, Landoltweg 1, Aachen 52074, Germany; ∥ Assay Profiling and Cell Sciences, Discovery Sciences, BioPharmaceuticals R&D, AstraZeneca, Pepparedsleden 1, Mölndal 43183, Sweden; ⊥ DMPK, Research and Early Development, Respiratory and Immunology (R&I), BioPharmaceuticals R&D, 128698 AstraZeneca, Pepparedsleden 1, Mölndal 43183, Sweden; # Drug Metabolism and Pharmacokinetics Department, 7412 Genentech, Inc., 1 DNA Way, South San Francisco, California 94080, United States; ∇ Discovery Chemistry Department, 7412 Genentech, Inc., 1 DNA Way, South San Francisco, California 94080, United States

## Abstract

Amides and trifluoromethyl groups are among the most widely used structural motifs in materials science, medicinal chemistry, and agrochemistry. In contrast, their direct combination as *N*-trifluoromethyl amides and the closely related *N*-trifluoromethyl carbamates and ureas has remained largely unexplored. This disconnect has primarily stemmed from the lack of synthetic methods and their unknown stabilities and physicochemical properties. Enabled by recently developed methodologies to synthesize *N*-trifluoromethyl carbonyl compounds, we systematically evaluated their aqueous stability and drug-relevant properties to assess their usefulness for compound optimization. All investigated *N*-trifluoromethyl derivatives display high aqueous stability at pH 7.4, including in human plasma, except for one *N*-trifluoromethyl carbamate series. *N*-Trifluoromethyl carbonyl motifs have a lipophilicity and Caco-2 permeability similar to their *N*-isopropyl analogues, while, in several cases, offering improved pharmacokinetic profiles. These findings establish *N*-trifluoromethyl carbonyl motifs as highly attractive functionalities, providing medicinal chemists with a framework for their incorporation into future drug-discovery programs.

## Graphical abstract

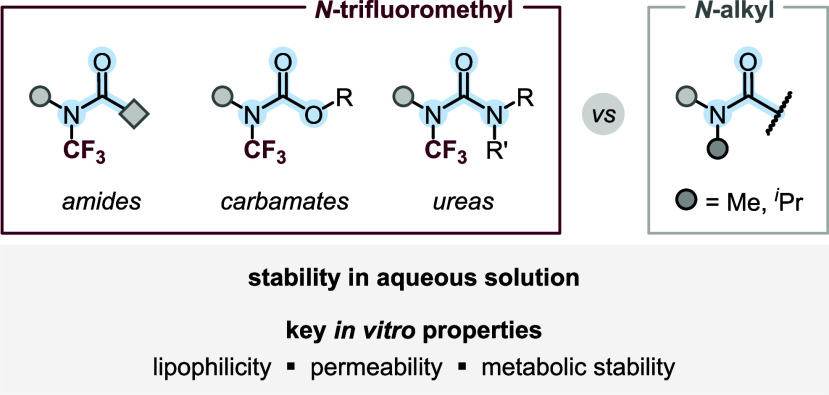

## Introduction

Amides and structurally related carbonyl derivatives are of central importance across the physical and life sciences owing to their exceptional stability and well-defined conformational properties.^1,2^ These functional groups are foundational to a wide range of applications, for example, in pharmaceuticals, agrochemicals, and materials science. Notably, more than 25% of the top 100 bestselling pharmaceutical agents have an amide moiety.^3^ Closely related carbonyl-containing scaffolds, including carbamates and ureas, further exemplify the structural and functional versatility of this motif. These functionalities are widely deployed as insecticides, polymeric materials (e.g., polyurethanes, elastomers, and foams), preservatives, cosmetics, and therapeutic agents, with prominent applications in chemotherapy and antiviral treatments.^4–7^

The strategic exploration of novel chemical space is widely recognized as a critical driver of innovation in drug discovery, materials design, and crop protection.^8–10^ In this context, the modification of canonical amide cores represents a powerful strategy to access previously unexplored molecular architectures with differentiated properties and applications. For example, *N*-methylation strategies in peptides and polymers have been widely exploited to enhance aqueous solubility, metabolic stability, and membrane permeability.^11^ On the other hand, fluorination of organic molecules is a well-established tool for fine-tuning key physicochemical parameters^12–15^ such as molecular conformation, chemical stability, acidity/basicity, membrane permeability, and lipophilicity, frequently leading to improved pharmacokinetic (PK) and pharmacodynamic profiles. Among fluorinated substituents, the trifluoromethyl group is especially prevalent in medicinal chemistry and appears in numerous marketed drugs in the form of O–CF_3_, C–CF_3_, and S–CF_3_ motifs.

Recognizing the complementary benefits of these approaches, the incorporation of an *N*-trifluoromethyl (*N*-CF_3_) substituent into carbamoyl frameworks represents an attractive yet largely untapped opportunity to fine-tune molecular entities and obtain potentially advantageous properties. Until recently, however, the lack of general and practical synthetic methods^16^ precluded the systematic exploration of such motifs in medicinal chemistry. Recent advances, first pioneered by Schoenebeck,^17–22^ have overcome this limitation and enabled access to a broad range of *N*-CF_3_ analogues of amides, ureas, carbamates, formamides,^23^ alkynamides,^24^ and related derivatives.^25–28^ These *N*-CF_3_ carbonyl compounds are compatible with diverse reaction conditions, including oxidative, reductive, acidic, basic, photochemical, and transition-metal-catalyzed processes. Previously published conformational studies^17,23,27^ further indicate that the strongly electron-withdrawing CF_3_ group attenuates nitrogen lone-pair delocalization, likely reducing amide resonance and conformational rigidity relative to classical amide analogues.

Despite these promising attributes, the potential of *N*-CF_3_ carbonyl motifs in medicinal chemistry remains largely unexplored. With recent synthetic breakthroughs enabling their systematic evaluation, this gap can be addressed. Herein, we report a comprehensive investigation of a structurally diverse set of *N*-CF_3_ amides, carbamates, and ureas, alongside their *N*-alkylated counterparts, to assess key drug-relevant properties, including aqueous stability, lipophilicity, solubility, membrane permeability, and metabolic stability. Across this compound set, the *N*-trifluoromethyl amides, carbamates, and ureas exhibit high aqueous and plasma stability, together with a lipophilicity and Caco-2 permeability comparable to their *N*-isopropyl analogues, while in several cases also providing enhanced metabolic stability. These results establish *N*-CF_3_ carbonyl motifs as robust and highly useful design elements for medicinal chemistry and future drug discovery efforts.

## Results and Discussion

In the absence of prior data on the medicinal chemistry potential of *N*-CF_3_ carbonyl derivatives, we reasoned that a systematic matched molecular pair (MMP) analysis^29^ would provide an informative framework for elucidating how targeted structural modifications influence properties. Accordingly, we initiated our investigation with structurally simple *N*-CF_3_ amides, ureas, and carbamates and compared them to their matched *N*-H and *N*-CH_3_ analogues. By initially focusing on structurally less complex molecules, we intended to most clearly delineate the impact of CF_3_ substitution on physicochemical and biological properties. We aimed for the *N*-CF_3_ amides, ureas, and carbamates to bear diverse functional groups to cover a broad property space. All fluorinated compounds were synthesized according to a recently reported procedure by Schoenebeck and coworkers,^17^ involving the conversion of isothiocyanates with AgF and triphosgene, followed by diversification of the resulting *N*-CF_3_ carbamoyl fluoride intermediate to the corresponding amides, carbamates, or ureas via reaction with appropriate nucleophiles (see Scheme 1 and the Supporting Information for details). The resulting compounds were evaluated using a suite of high-throughput assays to assess lipophilicity, kinetic solubility at pH 7.4, liver microsomal stability, and membrane permeability.

**Scheme 1:**
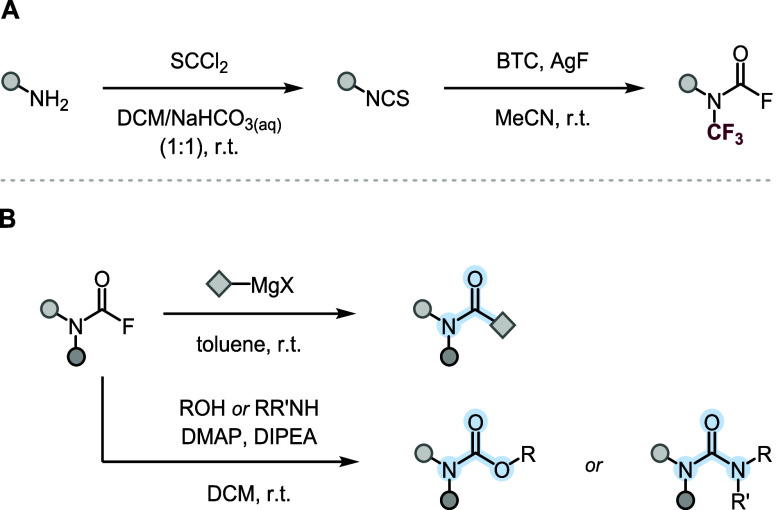
Synthesis of *N*-CF_3_ compounds; (**A**) Synthesis of Carbamoyl Fluoride Building Blocks; (**B**) Derivatization of Carbamoyl Fluorides into Amides, Carbamates, and Ureas

Our data show that consistent with established trends for fluorination, the *N*-CF_3_ amides generally exhibit higher lipophilicity (log*D*_7.4_) than their corresponding *N*-H and *N*-CH_3_ analogues (Tables 1 and 2).

**Table 1:**
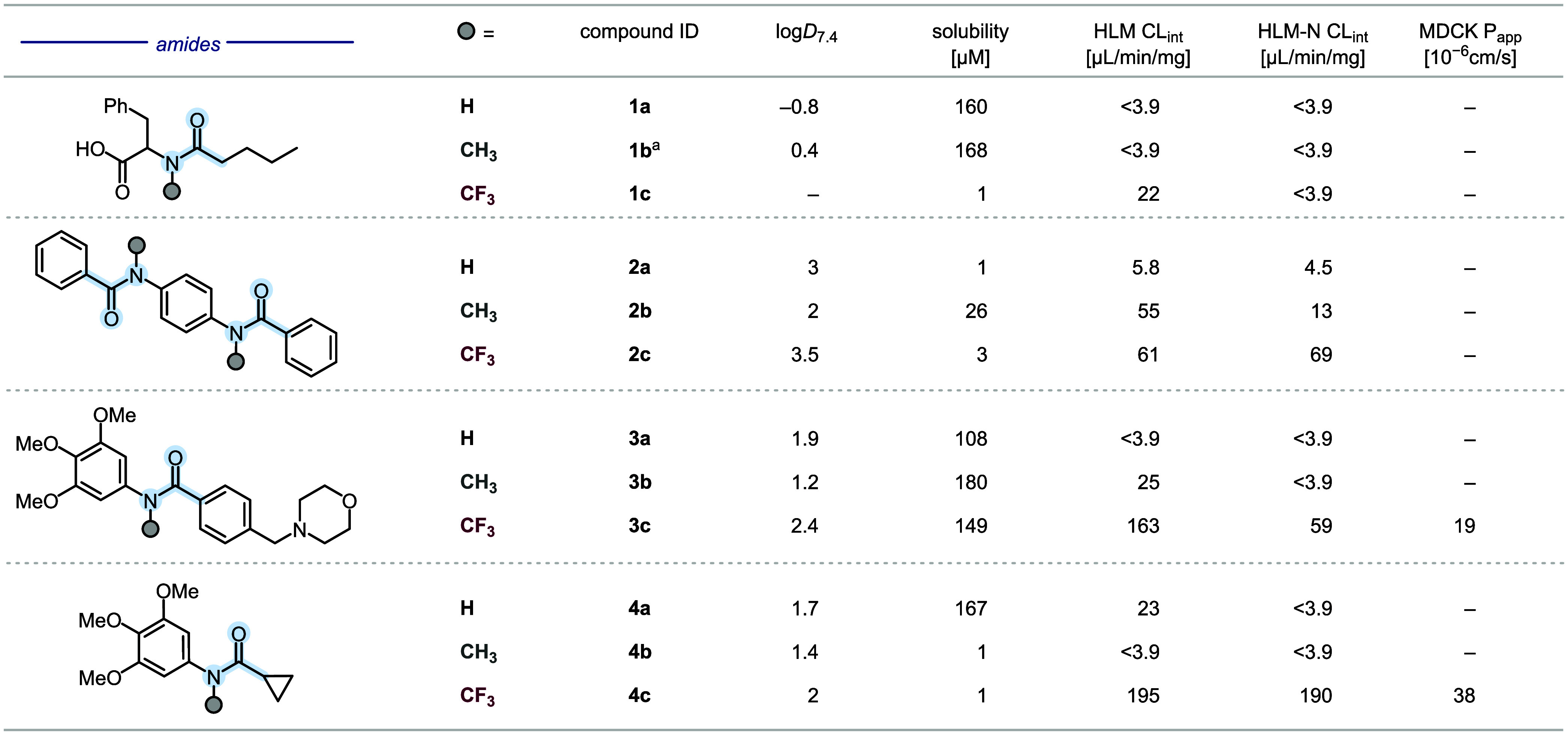
Comparison of Key *In Vitro* Parameters between *N*–H, *N*–Me, and *N*–CF_3_ Amide Matched Pairs

a (*S*)-Isomer was used; others racemic.

**Table 2:**
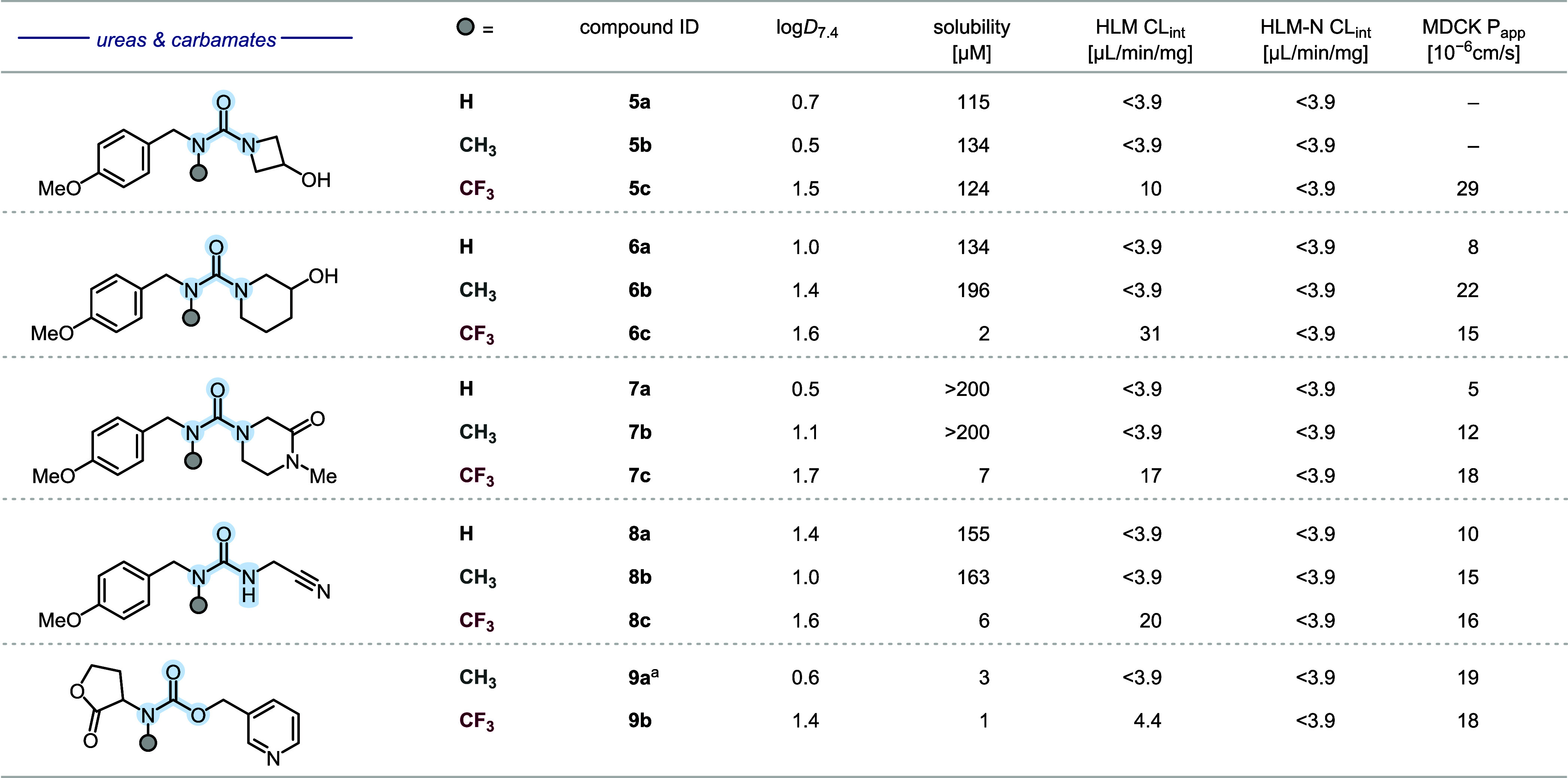
Comparison of Key *In Vitro* Parameters between *N*–H, *N*–Me, and *N*–CF_3_ Urea and Carbamate Matched Pairs^a^

a (*S*)-Isomer was used; other racemic.

For the aniline-derived *N*-CH_3_ amides **2b**, **3b**, and **4b**, log*D*_7.4_ values are lower than those of the corresponding *N*-H analogues **2a**, **3a**, and **4a**, likely reflecting steric disruption of coplanarity between the amide moiety and the aromatic ring upon *N*-CH_3_ substitution.^30^ That said, the bulkier *N*-CF_3_ amide analogues consistently display the highest lipophilicity across each matched series.

In line with their higher lipophilicity, *N*-CF_3_ carbonyl compounds generally show reduced kinetic solubility relative to their *N*-CH_3_ and *N*-H counterparts. However, high kinetic solubility can be achieved when additional solubilizing functionalities are incorporated, as exemplified by the morpholine substituent in **3c** and the hydroxyazetidine moiety in **5c**.

The *in vitro* metabolic stability of *N*-CF_3_ carbonyl compounds was next evaluated using human liver microsomes. Intrinsic clearance values correlate with lipophilicity and decrease overall in the order *N*-CF_3_ > *N*-CH_3_ > *N*-H, assuming similar assay binding between analogues or increasing assay binding with lipophilicity. A major concern regarding the *N*-CF_3_ carbonyl motif was that the strong electron-withdrawing effect of the trifluoromethyl substituent might activate these motifs to enzymatic hydrolysis, potentially rendering these compounds less stable than their *N*-CH_3_ analogues. To directly assess this risk, we compared intrinsic clearance (CL_int_) in human liver microsomes in the presence of NADPH (HLM) and without NADPH (HLM-N) to suppress cytochrome P450-mediated oxidation and thereby dissect hydrolytic liability.^31^

Under these conditions, *N*-CF_3_ amides derived from aliphatic amines (**1c**), as well as *N*-CF_3_ ureas (**5c**, **6c**, **7c,** and **8c**) and the *N*-CF_3_ carbamate **9b**, exhibited high stability in the HLM-N assay, with CL_int_ values below 3.9 μL min^–1^ mg^–1^, comparable to their *N*-CH_3_ and *N*-H analogues. In contrast, *N*-CF_3_ amides derived from anilines showed reduced stability in the HLM-N assay, consistent with increased susceptibility to hydrolytic turnover.

To further probe hydrolytic liability, metabolic profiling was performed for selected compounds (**3c**, **4c**, **6c**, **7c**, and **9b**) following incubation in human hepatocytes (Table S1).^32^ Consistent with the microsomal data, only compound **3c** displayed detectable amide hydrolysis, whereas the *N*-CF_3_ carbamate and urea derivatives remained stable with respect to carbonyl cleavage under identical conditions. Collectively, these results demonstrate that the hydrolytic susceptibility of *N*-CF_3_ carbonyl compounds is highly structure-dependent and can be effectively mitigated through appropriate substitution patterns. Importantly, many *N*-CF_3_ carbonyl derivatives exhibit excellent intrinsic stability, which underscores their promise as viable and robust motifs for medicinal chemistry applications.

Finally, membrane permeability was evaluated using Madin–Darby canine kidney (MDCK) cells in which endogenous canine MDR1 had been genetically ablated using a previously reported method.^33^ Overall, *N*-CF_3_ carbonyl compounds exhibited high permeability, with apparent A-to-B permeability coefficients (P_app_) exceeding 10 × 10^–6^ cm s^–1^, and consistently showed improved permeability relative to their *N*-H matched molecular pairs, in line with their increased lipophilicity.

Building on our studies with structurally simple compounds, we next turned to more complex systems by examining *N*-trifluoromethyl analogues of known bioactive compounds. These compounds were evaluated for aqueous stability at pH 1.0, 7.4, and 10.0 (25 °C), as well as for stability in human blood plasma at 37 °C. This set of experiments was designed to more systematically probe the influence of steric and electronic environments on the stability of the *N*-trifluoromethyl group.

To this end, we synthesized a series of *N*-CF_3_ amides, carbamates, and ureas in which the second substituent on the nitrogen was either methyl, cyclopropyl, benzyl, or phenyl (Table 3 and Scheme 2). The *N*-trifluoromethyl amide series comprises compounds **10a–d**, rivaroxaban analogues in which the CF_3_ group is attached to an aliphatic nitrogen, and compounds **11a–d**, paracetamol analogues bearing an anilinic *N*-trifluoromethyl amide. Within the carbamate class, compounds **12a–d** (metronidazole analogues) and **13a–d** (tiaramide analogues) contain aliphatic oxygen substituents, whereas compounds **14a–d** represent paracetamol analogues in which an *N*-trifluoromethyl carbamate is installed at the phenolic oxygen, in contrast to the corresponding amide series **11a–d**. The two *N*-trifluoromethyl urea series comprise compounds **15a–d** (ciprofloxacin analogues) and **16a–d** (sildenafil analogues).

**Table 3:**
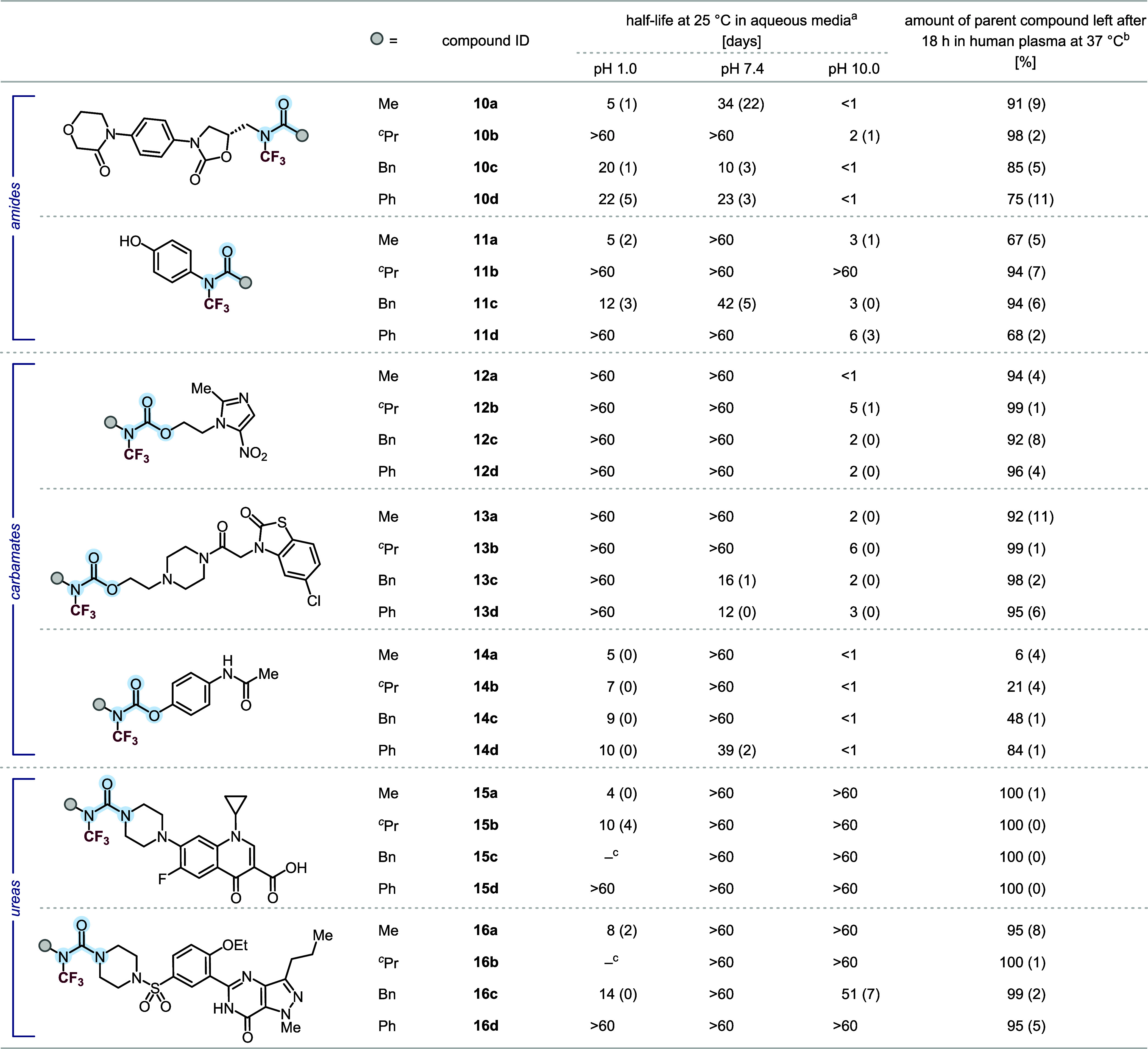
Stability of *N*-Trifluoromethyl Compounds **10–16** in Aqueous Media^a^ and Human Plasma^b^

a Half-life (in days) at 25 °C in 0.1 M HCl solution (pH 1.0), 20 mM sodium phosphate buffer (pH 7.4), or 20 mM sodium carbonate buffer (pH 10.0), given as the arithmetic mean of at least two independent measurements, with the standard deviation in brackets.

b Amount of compound left after 18 h in human plasma at 37 °C (in %) given as the arithmetic mean of at least three independent measurements, with the standard deviation in brackets.

c The half-life was not determined because of the compound being insoluble under these conditions.

**Scheme 2:**
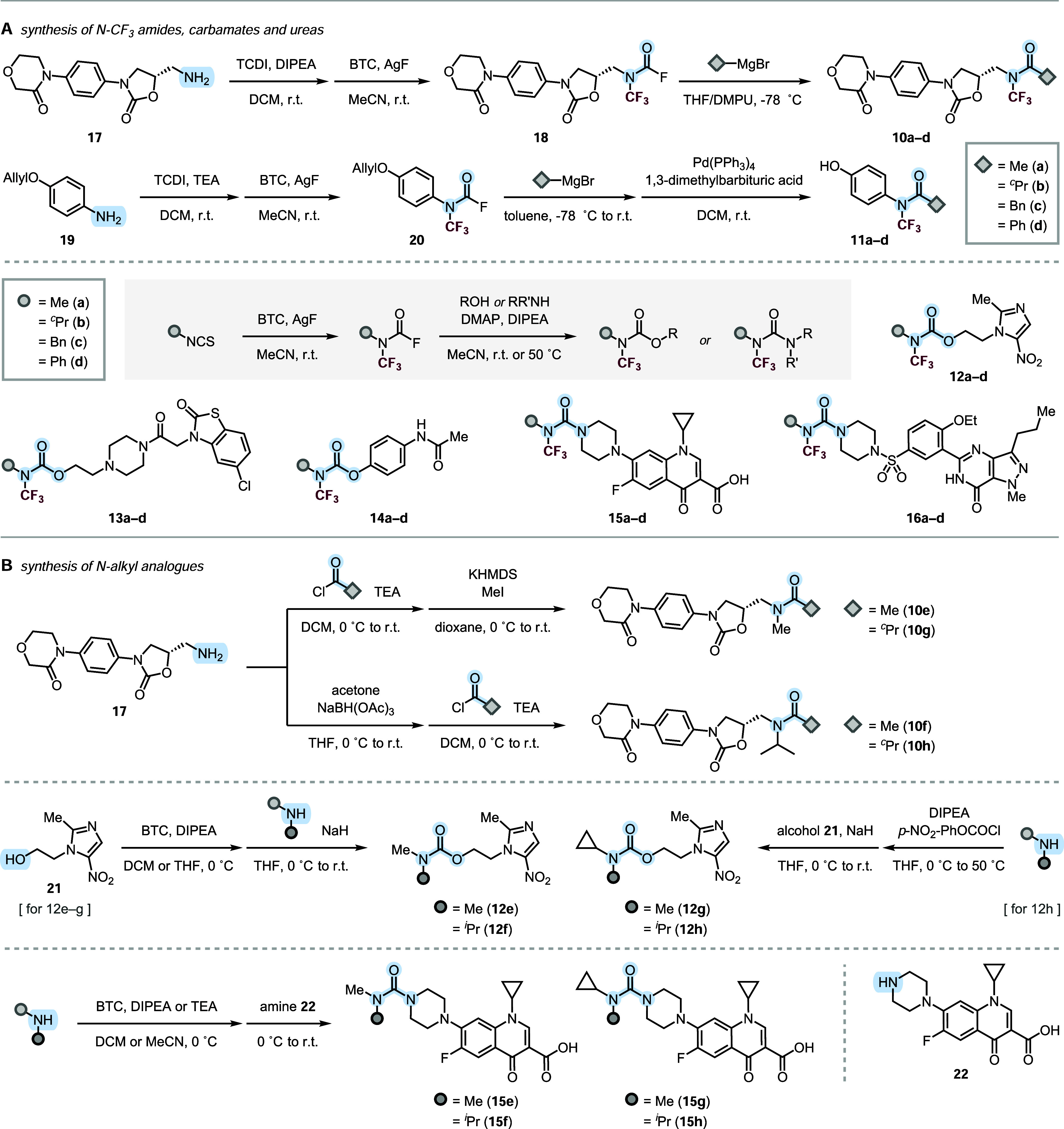
Synthesis of *N*-trifluoromethyl Compounds **10–16** and Non-Fluorinated Analogues; (A) Synthesis of *N*–CF_3_ Amides, Carbamates, and Ureas; (B) Synthesis of *N*-alkyl Analogues^a^

a For synthesis details, refer to the experimental section. BTC = bis­(trichloromethyl)­carbonate; TCDI = 1,1′-thiocarbonyldiimidazole.

Beyond stability assessment, we also compared key physicochemical and *in vitro* properties of selected *N*-trifluoromethyl compounds with those of their *N*-methyl and *N*-isopropyl matched molecular pairs to provide medicinal chemists with practical guidance on the property space associated with the *N*-trifluoromethyl moiety.

While all *N*-trifluoromethyl amides, carbamates, and ureas were synthesized according to reported procedures developed by the Schoenebeck group (Schemes 1 and 2A),^17^ the *N*-methyl analogues **10e** and **10g** were prepared via acylation of amine **17** with the corresponding acyl chlorides, followed by *N*-methylation using methyl iodide and potassium hexamethyldisilazide. The *N*-isopropyl analogues **10f** and **10h** were obtained through reductive amination of acetone with amine **17**, followed by acylation.

For the carbamate analogues **12e–g**, metronidazole **21** was first converted to the corresponding *O*-trichloromethyl carbonate using bis­(trichloromethyl)­carbonate and subsequently treated with the appropriate amine. The more sterically hindered analogue **12h** was synthesized by the reaction of *N*-isopropyl-*N*-cyclopropylamine with 4-nitrophenyl chloroformate, followed by coupling with deprotonated **21**. Finally, the urea analogues **15e–h** were prepared by the conversion of the corresponding amines to *O*-trichloromethyl carbamates using bis­(trichloromethyl)­carbonate, followed by reaction with ciprofloxacin **22**.

All investigated *N*-trifluoromethyl carbonyl compounds exhibit high to excellent aqueous stability at pH 7.4, with half-lives of at least 10 days and 21 of 28 compounds (75%) displaying half-lives exceeding 60 days (Table 3). This stability is striking, given that *N*-trifluoromethyl amines are known to undergo rapid hydrolysis at pH 7.4, typically with half-lives below 1 day,^34^ indicating that incorporation of a carbonyl functionality in *N*-trifluoromethyl amides, carbamates, and ureas markedly stabilizes the *N*-trifluoromethyl group. At pH 1.0 and 10.0, more pronounced differences between the compound classes were observed. Under acidic conditions, *N*-trifluoromethyl carbamates **12a–d** and **13a–d** showed excellent stability, with half-lives greater than 60 days, whereas most *N*-trifluoromethyl amides and ureas exhibited moderate stability, with half-lives ranging from 4 to 22 days. Notable exceptions include the cyclopropyl-substituted analogues **10b** and **11b**, as well as the phenyl-substituted analogues **11d**, **15d**, and **16d**, all of which retained half-lives exceeding 60 days. The reduced apparent stability of carbamates **14a–d** at pH 1.0 arises from hydrolysis of the additional acetamide moiety rather than cleavage of the *N*-trifluoromethyl carbamate itself. Under basic conditions (pH 10.0), excellent stability was largely restricted to the urea series **15a–d** and **16a–d**, along with amide **11b**, while the remaining analogues displayed half-lives of 6 days or less. In contrast, the corresponding *N*-methyl analogues of the two amide and three carbamate series exhibited substantially higher stability at pH 10.0 (see Supporting Information), highlighting the distinct reactivity profile of *N*-trifluoromethyl carbonyl motifs under strongly basic conditions.

In the next step, we sought to identify the decomposition products formed from each *N*-trifluoromethyl analogue at pH 1.0, 7.4, and 10.0. To this end, each compound was incubated for 24 h at 70 °C in aqueous solutions at the respective pH values, and the resulting mixtures were analyzed by LC-MS (for *N*-CF_3_ methyl analogues **10a–16a,** see Figure 1; for all other analogues, see Supporting Information). For the *N*-trifluoromethyl amide series **10a–10d**, the predominant chromatographic signal corresponded to the free amine **17**, arising from hydrolysis of both the amide bond and the trifluoromethyl group. An exception was observed for the cyclopropyl analogue **10b** at pH 1.0 and 7.4, where the intact parent compound remained the major species, consistent with having the longest half-life within this series. A secondary degradation product (**23**), resulting from additional hydrolysis of the δ-lactam, was only detected in significant amounts for **10b** at pH 10.0 and in trace amounts for **10a**, **10c,** and **10d** at pH 1.0 and 10.0. This indicates that the *N*-trifluoromethyl amide is generally more susceptible to hydrolysis than the δ-lactam within these compounds.

**Figure 1:**
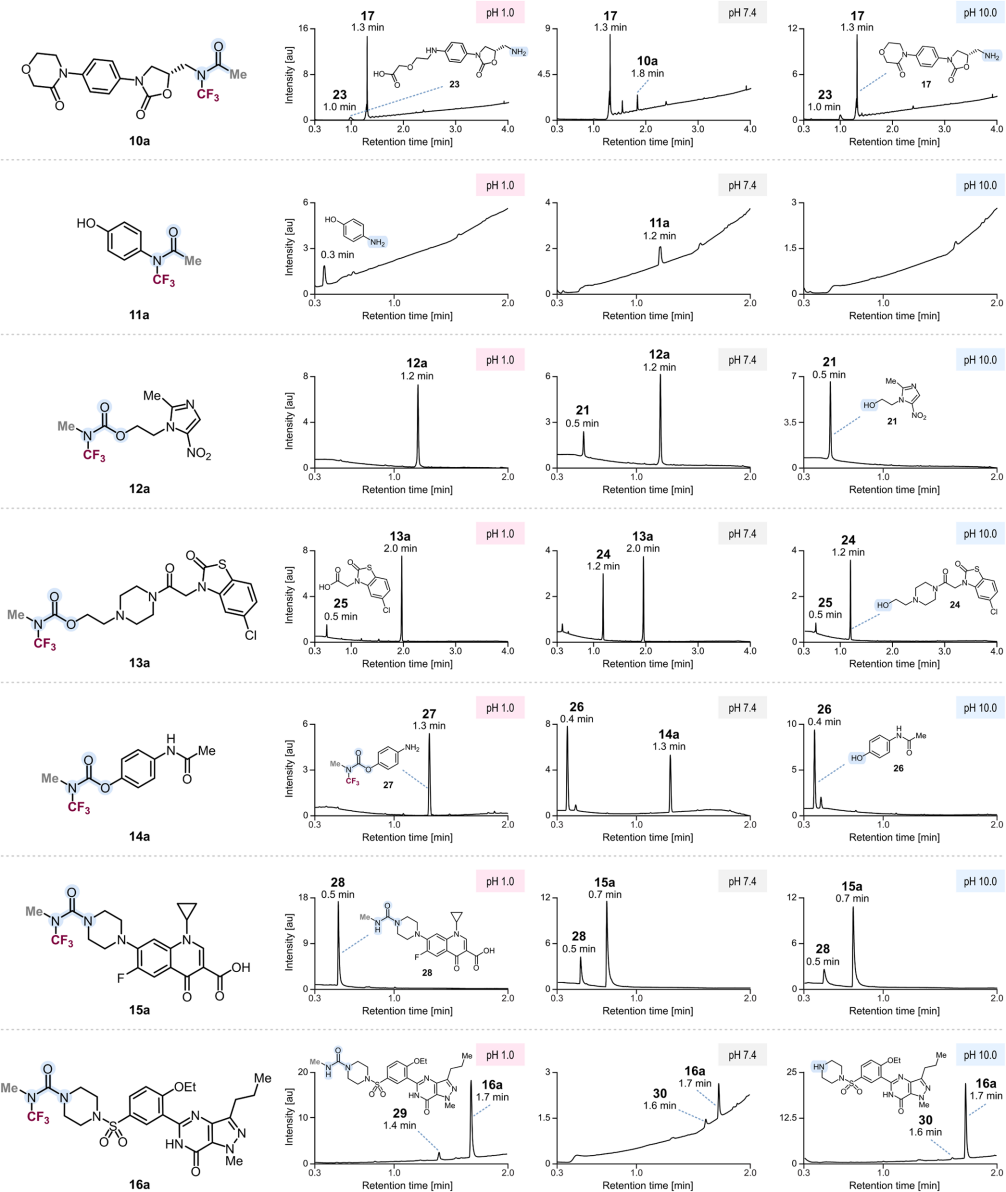
Chromatograms after incubation of *N*-CF_3_ analogues **10a**–**16a** in 0.1 M HCl solution (pH 1.0), 20 mM sodium phosphate buffer (pH 7.4), and 20 mM sodium carbonate buffer (pH 10.0) at 70 °C for 24 h. Each decomposition mixture was analyzed using LC-MS with six different gradients, and the gradient most suitable to depict all decomposition products is shown (for more details on the experimental procedure and the LC gradients, see Supporting Information). Decomposition products were identified based on their MS trace.

In contrast, the second *N*-trifluoromethyl amide series **11a–11d** exhibited markedly higher stability, with no or only minor decomposition detected under most conditions. Extensive degradation was observed only for **11a** at pH 1.0 and for **11a**, **11c**, and **11d** at pH 10.0. In agreement with series **10a–10d**, the principal decomposition pathway involved hydrolysis of the amide and loss of the trifluoromethyl group, resulting in the formation of *p*-aminophenol as the main product for series **11a–11d**.

Consistent with the long half-lives observed for the *N*-trifluoromethyl carbamate series **12a–d** at pH 1.0 and 7.4, no or only trace to minor amounts of decomposition products were detected under these conditions. In several cases (**12a**, **12b**, and **12d** at pH 1.0 and **12b** at pH 7.4), no detectable degradation was observed. When present, the decomposition product was identified as metronidazole **21**, arising from hydrolysis of both the carbamate linkage and the *N*-trifluoromethyl group. In contrast, at pH 10.0, metronidazole became the dominant chromatographic signal, with no residual parent compounds detected, in agreement with the reduced half-lives measured under basic conditions.

A similar degradation profile was observed for the second *N*-trifluoromethyl carbamate series **13a–d**. At pH 7.4 and 10.0, the primary decomposition pathway involved hydrolysis of the carbamate and the *N*-trifluoromethyl group to yield tiaramide **24**. While at pH 7.4, still intact parent compounds **13a–d** were detected, at pH 10.0, complete conversion to tiaramide occurred, consistent with the shorter half-lives at elevated pH. Notably, at pH 1.0, the major decomposition product detected was **25** and not tiaramide, indicating that the *N*-trifluoromethyl carbamate moiety is more stable under acidic conditions than the amide within this series.

For the third *N*-trifluoromethyl carbamate series **14a–d**, the predominant decomposition product at pH 7.4 and 10.0 was the free phenol **26**, resulting from the hydrolysis of the carbamate and the loss of the trifluoromethyl group. As observed for series **13a–d**, the *N*-trifluoromethyl carbamate functionality in **14a–d** proved more stable than the accompanying amide under acidic conditions, with the hydrolysis of the acetamide to yield the free aniline (for example, compound **27** as the decomposition product of **14a**) detected as the major degradation pathway at pH 1.0.

Notably, for none of the *N*-trifluoromethyl amides or carbamates, detrifluoromethylation to the corresponding *N*-H amides or carbamates was detected under any of the investigated conditions. This behavior stands in sharp contrast to the *N*-trifluoromethyl urea compounds **15a–c**, for which detrifluoromethylated products (for example, compound **28** as a decomposition product of **15a**) were detected at pH 1.0, 7.4, and 10.0, mostly in trace to small amounts. Importantly, hydrolysis of the urea to release ciprofloxacin **22** was not observed for any analog, with the exception of **15b**, where trace amounts were detected at pH 1.0.

For the second *N*-trifluoromethyl urea series **16a–d**, the parent *N*-trifluoromethyl urea remained the predominant species across all pH conditions. Minor amounts of detrifluoromethylation were detected only for **16a** at pH 1.0 (analogue **29**) and for **16c** at pH 7.4 and 10.0, while trace formation of free piperazine **30** was observed for **16a** at pH 7.4 and 10.0.

Collectively, these data demonstrate that *N*-trifluoromethyl amides, carbamates, and ureas possess good to excellent aqueous stability at pH 1.0 and 7.4, in marked contrast to *N*-trifluoromethyl amines, which undergo rapid hydrolysis.^34^ Under basic conditions (pH 10.0), *N*-trifluoromethyl ureas retain excellent stability, whereas prolonged exposure of *N*-trifluoromethyl amides and carbamates to strongly basic aqueous environments should be avoided. Overall, the aqueous stability profiles of *N*-trifluoromethyl amides, carbamates, and ureas are compatible with the majority of medicinal chemistry applications. With the exception of the urea series, the dominant degradation pathway for *N*-trifluoromethyl carbonyl compounds involves hydrolysis of the carbonyl linkage accompanied by loss of the *N*-trifluoromethyl group to yield the corresponding amines (from amides) or alcohols (from carbamates), whereas detrifluoromethylation is more pronounced for *N*-trifluoromethyl ureas.

Encouraged by the high stability of the investigated *N*-trifluoromethyl carbonyl compounds in a buffered solution at pH 7.4, we next investigated their stability in human blood plasma at pH 7.4 and 37 °C. As shown in Table 3 for 20 of the 28 analogues, at least 91% of the parent *N*-CF_3_ carbonyl compound is still present after incubation in human blood plasma at 37 °C for 18 h (for the 8 analogues with <91% recovery after 18 h, the corresponding *N*-methyl analogues showed excellent stability in human blood plasma, confirming that these *N*-trifluoromethyl compounds are less stable in human blood plasma). Both *N*-trifluoromethyl urea series and two of the three *N*-trifluoromethyl carbamate series showed very high to excellent stability in human blood plasma. However, strikingly, the *N*-trifluoromethyl carbamate series **14a**–**14d**, especially derivatives **14a** and **14b** with a small substituent at the nitrogen, degrade substantially in human blood plasma. For all four analogues, we detected *N*-(4-hydroxyphenyl)­acetamide **26** as the major metabolite, confirming the hydrolysis of the *N*-trifluoromethyl carbamate in human blood plasma (Figure 2). Interestingly, the amount of metabolite formed seems to depend on the size of the alkyl/aryl residue, with the most metabolite detected for *N*-methyl analogue **14a** and the least for *N*-phenyl derivative **14d**. The formation of the metabolite is reduced for **14a** and **14b** in the presence of the acetylcholinesterase inhibitor Neostigmine (3-((dimethylcarbamoyl)­oxy)-*N*,*N*,*N*-trimethylbenzenaminium). This confirms that the formation of the metabolite is an enzymatic process, which is at least partially due to acetylcholinesterase. For compounds **14c** and **14d** bearing larger *N*-substituents, the acetylcholinesterase inhibitor does not result in reduced carbamate hydrolysis (nor did a butyrylcholinesterase inhibitor, data not shown), suggesting a different enzyme being involved in the metabolite formation.

**Figure 2:**
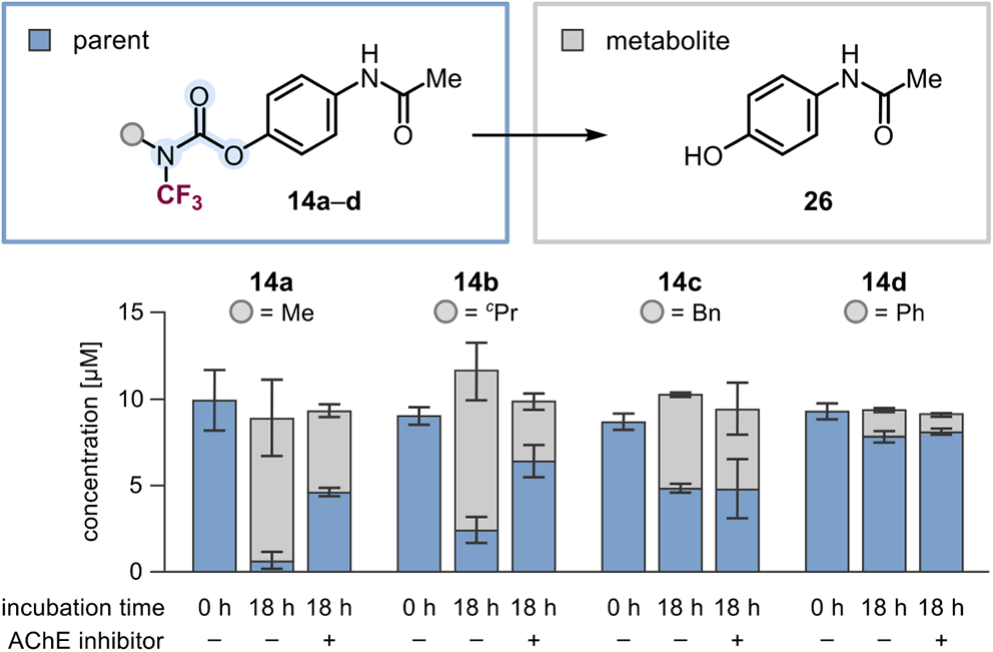
Amount of recovered parent *N*-trifluoromethyl carbamate (**14a**–**d**) and of formed metabolite *N*-(4-hydroxyphenyl)­acetamide (**26**) after incubation in human blood plasma at 37 °C for 18 h, with and without Neostigmine (3-((dimethylcarbamoyl)­oxy)-*N,N,N*-trimethylbenzenaminium) as an acetylcholinesterase (AChE) inhibitor.^35^

In the next step, we determined key *in vitro* properties of selected *N*-trifluoromethyl carbonyls and compared them to their *N*-methyl analogues (Table 4). In addition, we investigated *N*-isopropyl analogues to have MMPs with a lipophilicity similar to the *N*-trifluoromethyl compounds to give medicinal chemists guidance on which property space to expect when introducing a trifluoromethyl group onto the nitrogen of an amide, carbamate, or urea. The shake-flask log*D*_7.4_ of the *N*-trifluoromethyl carbonyls is 0.6–1.1 log units higher than the corresponding *N*-methyl (in line with our observations in Tables 1 and 2) and similar to their *N*-isopropyl analogues, except for the *N*-trifluoromethyl amides **10a** and **10b**, where the *N*-trifluoromethyl analogue is 0.4 log units more lipophilic than the *N*-isopropyl analog. Interestingly, for chromlog*D*_7.4_ (i.e., the chromatographically determined log*D*_7.4_), the difference in lipophilicity between the *N*-trifluoromethyl and the *N*-isopropyl analogues tends to be more pronounced, with the former having higher values for some matched molecular pairs. All *N*-trifluoromethyl compounds have high intrinsic Caco-2 permeability, similar to that of their *N*-isopropyl analogues, except for **10a** and **10b**, where the intrinsic Caco-2 permeability is higher for the *N*-trifluoromethyl analog, which might be (at least partly) due to the higher lipophilicity. Compared to *N*-methyl analogues, the *N*-trifluoromethyl derivatives **10a**, **10b**, and **15b** have a significantly higher intrinsic Caco-2 permeability compared to their *N*-methyl derivatives (*p* < 0.04%, 0.6%, and 0.4%, respectively), which might also be due (at least partly) to their higher lipophilicity. Efflux was not a concern for the compounds tested. Also, in line with their lipophilicity, the *N*-trifluoromethyl analogues and *N*-isopropyl analogues are bound to a similar extent to human plasma proteins (except for **10b**). All *N*-trifluoromethyl compounds except for **15b** have high to very high solubility. In line with the observations in Tables 1 and 2, in three out of six cases, the solubility of the *N*-trifluoromethyl compound is lower than the corresponding *N*-methyl analog. Encouragingly, *N*-trifluoromethyl ureas **15a** and **15b** are more metabolically stable in rat hepatocytes, human liver microsomes, and human hepatocytes than their isolipophilic *N*-isopropyl analogues **15f** and **15h,** and no difference in metabolic stability to their *N*-methyl analogues **15e** and **15g** was detected. This shows that exchanging an *N*-isopropyl substituent for an *N*-trifluoromethyl can be a viable strategy to increase the metabolic stability of a compound series. Finally, early CYP inhibition assessment for compounds **10a**, **12a**, **12b,** and **15a** did not raise any concerns (IC_50_ > 30 μM for all CYP tested—3A4, 2D6, 2C9, 2C19, 1A2).

**Table 4:**
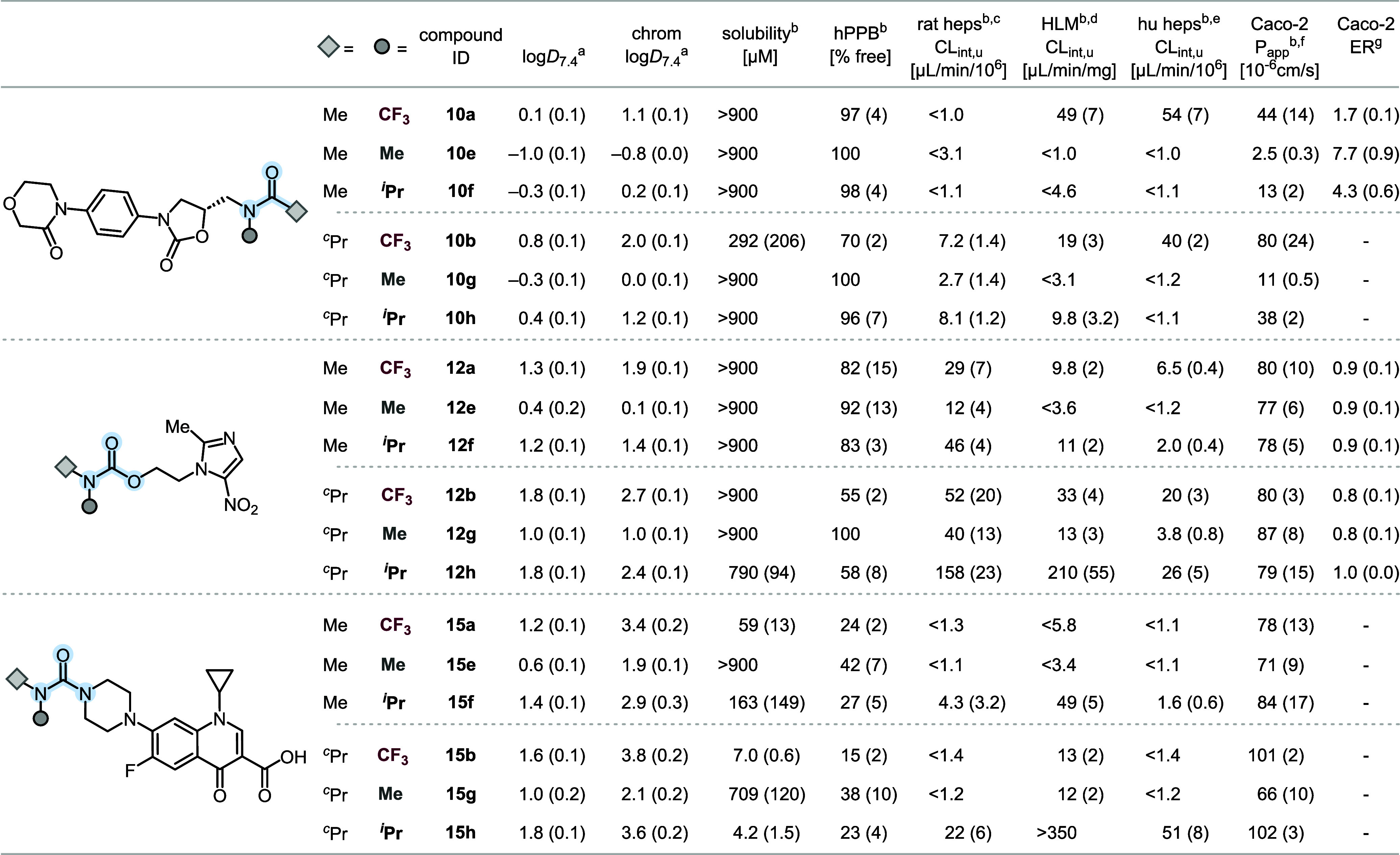
Overview of the Changes in Key *In Vitro* Properties Upon Exchanging *N*-CF_3_, *N*-Me, and *N-^i^* Pr Groups

a Values given as the arithmetic mean of at least three independent measurements, with the standard deviation in brackets.

b Values given as the geometric mean of at least three independent measurements, with the standard deviation in brackets.

c Metabolic stability of the compound was measured as the disappearance of the parent compound over time when incubated with rat hepatocytes. Corrected for f_u,inc_ in rat hepatocytes.

d Metabolic stability of the compound was measured as the disappearance of the parent compound over time when incubated with human liver microsomes (HLMs). Corrected for f_u,inc_ in HLM.

e Metabolic stability of the compound was measured as the disappearance of the parent compound over time when incubated with human hepatocytes. Corrected for f_u,inc_ (assuming human hepatocyte f_u,inc_ similar to rat hepatocyte f_u,inc_).^36^

f Apical-to-basolateral passive permeability across the Caco-2 cell monolayer in the presence of inhibitors against the three major efflux transporters: P-glycoprotein (P-gp), breast cancer resistance protein (BCRP), and multidrug-associated protein 2 (MRP2). For further details, see the Supporting Information.

g ER is the ratio of apical-to-basolateral passive permeability to basolateral-to-apical passive permeability across the Caco-2 cell monolayer in the absence of the inhibitors. Values given as the geometric mean of at least three independent measurements, with the standard deviation in brackets.

Encouraged by the low intrinsic *in vitro* clearance in rat and human hepatocytes of the *N*-trifluoromethyl compounds **15a** and **15b**, we assessed their *in vivo* PK properties in rats and compared them to those of their *N*-methyl (**15e**, **15g**) and *N*-isopropyl analogues (**15f**, **15h**). Overall, the *N*-trifluoromethyl analogues (**15a** and **15b**) display a better PK profile than their *N*-isopropyl MMP (**15f** and **15h**, respectively), with higher bioavailability, lower unbound clearance, and higher volume of distribution, resulting in a longer effective half-life and higher free exposure (Table 5). Comparing the *N*-trifluoromethyl analogues to their *N*-methyl MMP, **15a** has similar bioavailability, free AUC, and effective half-life to the *N*-methyl compound **15e**, while **15b** has a lower unbound clearance and higher V_ss,u_ than **15g**, resulting in a longer effective half-life. However, the free AUC is lower for **15b** than **15g**, likely due to lower absorption, as indicated by F_abs_. All in all, these data indicate that *N*-trifluoromethyl analogues may show a more desirable *in vivo* PK profile than their isolipophilic *N*-isopropyl counterparts while being fairly similar to their *N*-methyl analogues.

**Table 5:**
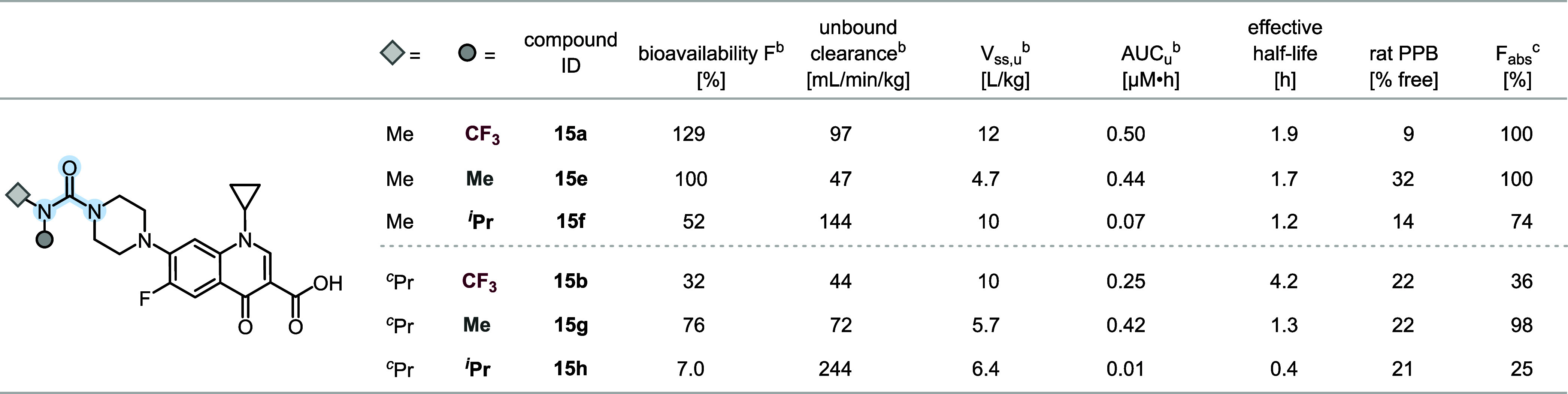
*In Vivo* Pharmacokinetic Properties in Rats of *N*-Trifluoromethyl Analogues **15a** and **15b** Compared to Their *N*-Methyl (**15e**, **15g**) and *N*-Isopropyl Analogues (**15f**, **15h**)­^a^

a *In vivo* pharmacokinetic properties in male Han Wistar rats (0.5 mg/kg i.v., 0.5 mg/kg p.o. for **15e**, **15f**; 1.0 mg/kg p.o. for **15a**, **15b**, **15g**, **15h**).

b Arithmetic mean of two animals.

c Formally, F_abs_ refers to $F_{a}\times F_{g}=\frac{F}{\left(1-\frac{CL_{b}}{LBF}\right)}$ , where CL_b_ is the blood clearance and LBF is the rat liver blood flow (72 mL/min/kg). F_g_ equals 1 assuming negligible gut metabolism.

## Conclusion

In summary, we have synthesized 37 *N*-trifluoromethyl amides, carbamates, and ureas and shown that the majority of them have a stability in aqueous media at pH 7.4 and in human blood plasma, which we deem sufficient for most applications in drug discovery. Exceptions are the *N*-trifluoromethyl amide **3c** and *N-*trifluoromethyl carbamates **14a**–**d** where enzymatic hydrolysis of the carbonyl motif was observed. The physicochemical properties of the *N*-trifluoromethyl compounds are similar to or better than those of their *N*-isopropyl analogues, which also translates to better PK profiles for the compounds tested in rats. Interestingly, for the vast majority of *N*-trifluoromethyl carbonyls, the major decomposition product is the free amine or alcohol. Given the increased awareness of sustainability in chemistry, installing the trifluoromethyl group onto the nitrogen of amides, carbamates, and ureas can be an exciting strategy to obtain compounds, which in the majority of cases are sufficiently stable for applications in drug discovery but, in contrast to the majority of C–CF_3_ containing compounds, might degrade with time. This could be a strategy to avoid persistence in the environment and might contribute to future endeavors toward compounds, which are degradable by design. We believe that the high aqueous stability of the investigated *N*-trifluoromethyl amides, carbamates, and ureas together with the demonstrated properties will facilitate the uptake of these functional groups in future drug discovery programs.

## Experimental Section

### General Methods

All reactions were performed utilizing standard Schlenk techniques under an argon atmosphere or inside a glovebox, unless otherwise stated. Glassware and magnetic stir bars were dried in an oven (130 °C) for at least 24 h prior to use or heated under vacuum at 600 °C for 2 min with a heat gun. Unless otherwise stated, experiments were carried out at room temperature (25 ± 2 °C). Reactions were monitored by analytical thin layer chromatography (TLC), gas chromatography coupled with mass spectrometry (GC-MS), or liquid chromatography coupled with mass spectrometry (LC-MS). TLC was performed on Macherey Nagel ALUGRAM Xtra SIL G UV254 aluminium plates with unmodified silica, or on silica-plated glass plates, employing Merck silica gel grade 60 F_254_, or on Biotage X50 KPNH TLC plates (5 × 10 cm). The spots were visualized either under UV light by a CAMAG UV lamp 4 dual wavelength 254/366 nm, 2 × 8W at λ = 254 nm, with iodine, or KMnO_4_ or phosphomolybdic acid (PMA) staining solutions. GC-MS analysis was performed on an Agilent Technologies 5975 series MSD mass spectrometer under electron ionization (EI) mode coupled with an Agilent Technologies 7820A gas chromatograph employing an Agilent 19091s-433 HP-5MS column (30 m × 0.250 μm × 0.250 μm). For LC-MS analysis, a GenTech Scientific Waters ACQ equipped with an Acquity ultra-performance liquid chromatography (UPLC) system, an HSS C18 column (1.8 μm, 2.1 × 50 mm column), and an SQ2 detector (ESI^+^ and ESI^–^) was employed. As mobile phases, acetonitrile and water (modified either with 47 mM ammonia and 6.5 mM ammonium carbonate, pH 10, or with 1 mM ammonium formate and 10 mM formic acid, pH3) were used. Flash silica gel column chromatography was performed with silica gel (0.04–0.063 mm particle size) purchased from Macherey Nagel, whereas, for automated flash column chromatography, a Biotage Selekt Flash Purification System with Biotage Sfär HC Duo and Biotage Sfär KP-Amino D prepacked columns was used. Preparative high-performance liquid chromatography (HPLC) was performed with 2 different systems. The former consisted of a Knauer Azura system (employing UV detector 2600 at 254 and 230 nm), using a Merck LiChrosorb Si60 column (7 μm, 250 × 25 mm). The latter consisted of a Waters Fraction Lynx system with a Waters Acquity SQD and a Waters binary gradient module 2525, with a flow of 60 mL/min at ambient temperature, equipped with different stationary phases: a Kromasil C8 column (10 μm, 250 mm × 20 mm), a Waters Sunfire C18 column (5 μm, 19 mm × 150 mm), a Waters XBridge C18 column (5 μm, 30 mm × 150 mm), a Waters Sunfire C18 column (5 μm, 10 mm × 100 mm), a Waters Sunfire C18 column (5 μm, 30 mm × 150 mm), a Waters BEH C18 column (1.7 μm, 2.1 mm × 50 mm), or a Waters Chromatorex C18 SMB 100–5T, Waters XBridge C18 5_MKM_ OBD.

The purity of all final compounds is 95% or higher. Solvents were removed *in vacuo* using either a Büchi Rotavap R200 with a Heating Bath B-490 and an external pump PC 3001 VARIO select or a Heidolph HeiVap Value Rotary Evaporator with bath temperatures up to 40 °C at a pressure of 20 mmHg (diaphragm pump) or at 0.1 mmHg (oil pump) at room temperature. For very high boiling solvents, a Biotage V10-Touch system was used. NMR spectra were recorded at an uncalibrated temperature of 26 °C on *Bruker Avance Neo* spectrometer with H, F, BB TBO probe at a frequency of 600.4 MHz (^1^H), 151.0 MHz (^13^C), and 564.9 MHz (^19^F); *Bruker Avance Neo* with H, F, BB TBO probe at a frequency of 400.0 MHz (^1^H), 100.6 MHz (^13^C), and 376.3 MHz (^19^F); *Varian* VNMRS with H, BB, P TBO probe at a frequency of 600.4 MHz (^1^H), 151.0 MHz (^13^C), and 564.9 MHz (^19^F) or *Varian* VNMRS H, BB, P TBO probe at a frequency of 400.0 MHz (^1^H), 100.6 MHz (^13^C), and 376.3 MHz (^19^F); *Bruker Avance Neo* spectrometer with a 5 mm QNP cryoprobe at a frequency of 500.1 MHz (^1^H), 127.5 MHz (^13^C) and 470.4 MHz (^19^F); *Bruker Avance Neo* spectrometer with a 5 mm BBO BB-H&F cryoprobe at a frequency of 500.1 MHz (^1^H), 121.5 MHz (^13^C), and 470.4 MHz (^19^F). ^13^C NMRs and ^19^F NMRs were run in proton-decoupled mode. Chemical shifts are reported in parts per million (δ) and referenced from the residual protonium for ^1^H NMR [CDCl_3_: δ 7.26 (CHCl_3_); DMSO-*d*_6_: δ 2.50 (DMSO-*d*_5_); acetone-*d*_6_: δ 2.05 (acetone-*d*_5_); CD_3_CN: δ 1.94 (CD_2_HCN); CD_2_Cl_2_: δ 5.32 (CDHCl_2_)]. ^13^C NMR shifts are referenced from the carbon reference of the solvent [CDCl_3_: δ 77.0; DMSO-*d*_6_: δ 39.5; acetone-*d*_6_: δ 29.8, 206.3; CD_3_CN: δ 1.3, 118.3; CD_2_Cl_2_: δ 54.3]. For ^19^F, shifts are referenced internally by the instrument after locking and shimming to the deuterated solvent. Coupling constants (*J*) are given in Hertz (Hz). Splitting patterns are denoted as s (singlet), d (doublet), t (triplet), q (quartet), hept (heptet), m (multiplet), and combinations thereof. Coupling constants (*J*) are reported in Hertz (Hz) and refer to H–H couplings in ^1^H NMR spectra or to C–F couplings in ^13^C NMR spectra, unless otherwise stated. High-resolution mass spectrometry (HRMS) was performed using a Thermo Scientific LTQ Orbitrap XL spectrometer (ESI), Finnigan MAT 95 (EI), or Bruker Maxis II LC-MS-System (APCI). Low-resolution masses of known compounds were extracted from the related GC-MS chromatograms.

### Materials

All anhydrous solvents were purchased from Sigma-Aldrich, VWR International, and Thermo Fisher Scientific. Dichloromethane, THF, and toluene were dried using an MBraun 800 or an Innovative Technology PS-MD-5 solvent purification system. Technical grade solvents were distilled prior to use for chromatography and extraction. All reagents and starting materials were purchased from Sigma-Aldrich, TCI Chemicals, Ambeed, Fluorochem, Combi-Blocks, Apollo Scientific, Chem-Impex, ChemBridge, Rieke Metals, Oakwood Chemicals, and BLDpharm and were used as received. Four Å molecular sieve powder was dried at 260 °C for 36 h under high vacuum, stored in an argon-filled glovebox, partially transferred into 20 mL vials, and stored in an oven at 120 °C prior to use (up to 5 days). Volumes lower than 0.1 mL were added with a Hamilton 1700 series, 100 μL Gastight Syringe, PTFE Luer Lock. Compounds **1a–c**, **2a–c** were synthesized according to the literature procedure.^17^

#### General Procedure **A** for the Synthesis of *N*-CF_3_ Amides **10a**–**d**

The corresponding carbamoyl fluoride (0.19 mmol, 1 equiv) was presolubilized in anhydrous 1,3-dimethyl-3,4,5,6-tetrahydro-2­(1*H*)-pyrimidinone (0.6 mL), and the mixture was diluted with dry THF (0.9 mL). The solution was transferred into a heat gun-dried vial and cooled down to −78 °C. Subsequently, the corresponding Grignard reagent (0.24 mmol, 1.3 equiv) was added dropwise, and the mixture was stirred at −78 °C for 15 min. The reaction was monitored via LC-MS analysis. If no conversion was observed, the temperature was raised to 0 °C and, after an additional 20 min, the mixture was checked again via LC-MS analysis. If partial conversion was observed, the mixture was cooled down to −78 °C, and an additional equivalent of the corresponding Grignard reagent was added, and the procedure was repeated. After complete conversion, the mixture was quenched with an aqueous saturated citric acid solution (0.5 mL) at 0 °C and allowed to reach room temperature. The resulting white-grayish suspension was extracted with dichloromethane (2×), the combined organic phases were washed with brine and dried over a phase separator, and the solvent was removed *in vacuo* at 43 °C. The resulting oil (due to the persistence of DMPU) was solubilized in DMSO (0.8 mL), filtered via a 0.45 μm filter, and purified via reversed-phase preparative HPLC, via the indicated conditions.

#### General Procedure **B** for the Synthesis of *N*-CF_3_ Amides **11a**–**d**

The corresponding carbamoyl fluoride (0.34 mmol, 1 equiv) was presolubilized in dry toluene (2.5 mL) and transferred into a heat gun-dried vial. The mixture was cooled down to −78 °C (unless otherwise stated), and the corresponding Grignard reagent (0.40 mmol, 1.3 equiv) was added dropwise. The reaction was monitored via LC-MS analysis after 10 and 30 min. After the consumption of the starting material, the reaction was quenched at 0 °C with 1 mL of an aqueous saturated ammonium chloride solution and was allowed to reach room temperature. The 2 phases were separated, the aqueous one was extracted with dichloromethane (2×), and the combined organic layers were washed with brine, dried over a phase separator, and the solvent removed *in vacuo* at 43 °C. This dark yellow viscous crude oil was directly predissolved in dry dichloromethane (0.8 mL) and transferred to a heat gun-dried vial. Subsequently, tetrakis­(triphenylphosphine)­palladium(0) (9 μmol, 5.5 mol %) and 1,3-dimethylbarbituric acid (0.21 mmol, 1.3 equiv) were sequentially added. The mixture was monitored via LC-MS analysis, showing full consumption of the starting material after 2 h. Then, the reaction was quenched with 0.5 mL of an aqueous saturated ammonium chloride solution, giving a biphasic mixture. The 2 phases were separated, and the yellow organic phase was dried *in vacuo* (43 °C) to deliver a greasy orange oil as crude. Subsequently, the crude was dissolved in DMSO (0.8 mL), filtered via a 0.45 μm filter, and purified via reversed-phase preparative HPLC, via the indicated conditions.

#### General Procedure **C** for the Synthesis of *N*-CF_3_ Carbamates **12a**–**d**, **13a**–**d**, **14a**–**d** and *N*-CF_3_ Ureas **15a**–**d** and **16a**–**d**

A heat gun-dried vial was sequentially charged with the corresponding amine or alcohol (0.15 mmol, 1 equiv), 4-dimethylaminopyridine (4 mg, 0.03 mmol, 0.2 equiv), the corresponding trifluoromethylcarbamoyl fluoride (0.30 mmol, 2 equiv) as ∼0.1 M acetonitrile solution (3.0 mL), and DIPEA (50 μL, 0.30 mmol, 2 equiv). The reaction mixture was stirred at 50 °C (unless stated otherwise) and monitored via LC-MS analysis. After 4–24 h, it was dried *in vacuo* at 43 °C, and the resulting crude was dissolved in 0.6 mL DMSO, filtered via a 0.45 μm filter, and purified via reversed-phase preparative HPLC, via the indicated conditions.

#### General Procedure **D** for the Synthesis of *N-*Isopropyl Analogues **10f**–**10h** of Compound Series **10a**–**d**

In a heat gun-dried vial, (*S*)-4-(4-(5-(aminomethyl)-2-oxooxazolidin-3-yl)­phenyl)­morpholin-3-one hydrochloride (**17**) (0.20 g, 0.61 mmol, 1 equiv) was suspended in THF (7.6 mL), and acetone (60 μL, 0.79 mmol, 1.3 equiv) was then added. Subsequently, sodium triacetoxyborohydride (0.19 g, 0.92 mmol, 1.5 equiv) was added in 2 portions at 0 °C. The mixture was left to stir at room temperature for 24 h, after which an aqueous saturated sodium bicarbonate solution was added (1 mL). The organic layer was separated and evaporated to dryness *in vacuo* at 43 °C to deliver a colorless oil. The oily crude was purified via normal phase chromatography with a Biotage KP-Amino column and EtOAc as the eluent phase, to deliver (*S*)-4-(4-(5-((isopropylamino)­methyl)-2-oxooxazolidin-3-yl)­phenyl)­morpholin-3-one (0.12 g, 0.35 mmol, 57%) as a colorless solid.

Subsequently, in a heat gun-dried 2–5 mL MW vial, (*S*)-4-(4-(5-((isopropylamino)­methyl)-2-oxooxazolidin-3-yl)­phenyl)-morpholin-3-one (50 mg, 0.15 mmol, 1 equiv) was solubilized in dry dichloromethane (1.4 mL), and triethylamine (32 μL, 0.23 mmol, 1.5 equiv) was added. The mixture was cooled down to 0 °C, and then, the corresponding acyl chloride (0.19 mmol, 1.3 equiv) was added dropwise. After the addition, the reaction was warmed up to room temperature and stirred for 16 h. Subsequently, the mixture was quenched with an aqueous saturated sodium bicarbonate solution (0.3 mL). The 2 phases were separated, the organic phase was washed with brine (0.5 mL) and dried over a phase separator, and the solvent was removed *in vacuo* at 43 °C to deliver a yellow oily crude. The crude was dissolved in 1.3 mL DMSO and purified via preparative HPLC, via the indicated conditions.

#### Characterization Data of (*S*)-4-(4-(5-((Isopropylamino)­methyl)-2-oxooxazolidin-3-yl)­phenyl)­morpholin-3-one

^**1**^ **H NMR** (500 MHz, acetone-*d*_6_) δ 7.67–7.62 (m, 2H), 7.44–7.38 (m, 2H), 4.75 (ddt, *J* = 8.8, 6.5, 5.2 Hz, 1H), 4.20–4.14 (m, 3H), 4.05–4.01 (m, 2H), 3.95 (dd, *J* = 8.7, 6.5 Hz, 1H), 3.82–3.77 (m, 2H), 2.94 (qd, *J* = 12.7, 5.2 Hz, 2H), 2.86–2.81 (m, 2H), 1.04 (dd, *J* = 6.2 Hz, 6H). ^**13**^ **C NMR** (126 MHz, acetone-*d*_6_) δ 166.7, 155.3, 138.3, 138.2, 126.7, 118.9, 73.7, 69.0, 64.8, 50.5, 50.3, 49.5, 48.8, 23.3. **HRMS** (ESI): *m*/*z* calculated for C_17_H_23_N_3_O_4_ + H^+^: 334.1767 [M + H]^+^, found: 334.1794.

#### General Procedure **E** for the Synthesis of *N*-Alkyl Analogues **12e**–**g** of Compound Series **12a**–**d**

In a heat gun-dried vial, NaH 60% in mineral oil (30 mg, 1.2 mmol, 2 equiv) was suspended in THF (1.0 mL) and stirred for 30 min at 0 °C, after which the corresponding amine hydrochloride (1.2 mmol, 2 equiv) was rapidly added. In a secondary heat gun-dried vial, 2-(2-methyl-5-nitro-1*H*-imidazol-1-yl)­ethan-1-ol (**21**) (0.10 g, 0.58 mmol, 1 equiv) and bis­(trichloromethyl) carbonate (BTC) (75 mg, 0.25 mmol, 0.5 equiv) were dissolved in THF (2.0 mL). The mixture was cooled down to 0 °C, DIPEA (0.41 mL, 2.3 mmol, 4 equiv) was added dropwise, and the suspension was stirred at 0 °C for 2 h. Subsequently, this suspension was transferred dropwise into the main vessel, and the resulting mixture was warmed up to room temperature. The reaction was monitored via LC-MS analysis. After 1.5 h, the reaction was quenched with an aqueous saturated sodium bicarbonate solution (0.5 mL). The 2 phases were separated, and the organic one was dried *in vacuo* at 43 °C. The resulting crude was solubilized in DMSO (0.8 mL), filtered via a 0.45 μm filter, and purified via the indicated conditions.

#### General Procedure **F** for the Synthesis of *N*-Alkyl Analogues **15e**–**g** of Compound Series **15a**–**d**

In a heat gun-dried vial, the corresponding amine (neat or as HCl salt) (0.18 mmol, 1.5 equiv) and bis­(trichloromethyl) carbonate (BTC) (14 mg, 48 μmol, 0.4 equiv) were dissolved in dry acetonitrile (2 mL). The mixture was cooled down to 0 °C and stirred for 10 min, after which DIPEA (80 μL, 0.48 mmol, 4.0 equiv) was added. After additional 30 min, 1-cyclopropyl-6-fluoro-4-oxo-7-(piperazin-1-yl)-1,4-dihydroquinoline-3-carboxylic acidhydrochloride **22** (44 mg, 0.12 mmol, 1.0 equiv) was added, and the resulting suspension was stirred at 0 °C for 2 h and 1 h at room temperature. The reaction was monitored via LC-MS, and after 3 h, the solvent was removed *in vacuo* at 43 °C. The resulting solid was suspended in dichloromethane (5 mL) and washed with an aqueous saturated NaHCO_3_ solution (5 mL). The 2 phases were separated, and the aqueous one was further extracted with dichloromethane (2 × 20 mL). The combined organic layers were washed with brine (10 mL) and dried over a phase separator, and the solvent was removed *in vacuo* at 43 °C. The resulting crude was solubilized in DMSO (0.8 mL), filtered via a 0.45 μm filter, and purified via the indicated conditions.

#### (*R*)-((2-Oxo-3-(4-(3-oxomorpholino)­phenyl)­oxazolidin-5-yl)­methyl)­(trifluoromethyl)­carbamoyl Fluoride (**18**)

A heat gun-dried vial was charged with (*S*)-4-(4-(5-(aminomethyl)-2-oxooxazolidin-3-yl)­phenyl)­morpholin-3-one hydrochloride (**17**) (0.20 g, 0.61 mmol, 1 equiv) and suspended in dry dichloromethane (1.7 mL). Then, DIPEA (0.14 mL, 0.79 mmol, 1.3 equiv) was added, and the mixture was stirred for 15 min. Subsequently, 1,1′-thiocarbonyldiimidazole (1,1′-TCDI) (0.14 g, 0.79 mmol, 1.3 equiv) was added in one portion, and the mixture was stirred for 16 h. Then, an aqueous saturated ammonium chloride solution (1 mL) was added, and the mixture was left to stir for 2–3 min. The organic phase was separated, and the aqueous phase was extracted with dichloromethane (2 × 10 mL). The combined organic layers were washed with brine (10 mL) and dried over a phase separator, and the solvent was removed *in vacuo* at 43 °C. The crude was purified via normal-phase automated flash chromatography with a Biotage Sfär HC Duo column (sample dissolved in acetone and loaded on a Samplet; the Samplet was dried *in vacuo* for 30 min) and EtOAc/ethanol as the eluent phase, following the gradient: 6–6% 1 CV; 6–50% 10 CV; 50–50% 2 CV. (*R*)-4-(4-(5-(isothiocyanatomethyl)-2-oxooxazolidin-3-yl)­phenyl)­morpholin-3-one was obtained as a yellow solid (0.13 g, 0.39 mmol, 64%).

Subsequently, a heat gun-dried vial was charged with silver­(I) fluoride (0.62 g, 4.9 mmol, 12.5 equiv) and bis­(trichloromethyl) carbonate (50 mg, 0.16 mmol, 0.4 equiv), and the vial was subsequently sealed, evacuated, and filled with Ar (cycle vacuum-Ar repeated 3 times). In a parallel pear-shaped flask, previously obtained (*R*)-4-(4-(5-(isothiocyanatomethyl)-2-oxooxazolidin-3-yl)­phenyl)­morpholin-3-one (0.13 g, 0.39 mmol, 1 equiv) was solubilized in dry acetonitrile (4 mL) and quickly transferred to the other vial. The suspension was vigorously stirred for 18 h at room temperature. Then, the obtained dark brown suspension was diluted with diethyl ether (3 mL) and further stirred for 15 min. The suspension was filtered through Celite and a phase separator to remove the last traces of salt byproducts. Then, the solvent mixture was removed *in vacuo* at 43 °C, and the crude was purified via normal-phase automated flash chromatography with a Biotage Sfär HC Duo 10 g column (sample dissolved in an acetone/dichloromethane/acetonitrile mixture and loaded on a Samplet support; the Samplet was dried *in vacuo* for 2 h) and EtOAc as the eluent phase. The title compound was obtained as a colorless solid (0.11 g, 0.27 mmol, 70%).

#### Characterization Data of (*R*)-4-(4-(5-(Isothiocyanatomethyl)-2-oxooxazolidin-3-yl)­phenyl)­morpholin-3-one

^**1**^ **H NMR** (500 MHz, DMSO-*d*_6_) δ 7.61–7.55 (m, 2H), 7.47–7.38 (m, 2H), 4.98 (dtd, *J* = 8.9, 5.4, 3.3 Hz, 1H), 4.24 (t, *J* = 9.3 Hz, 1H), 4.20 (s, 2H), 4.14 (dd, *J* = 15.3, 3.3 Hz, 1H), 4.06 (dd, *J* = 15.3, 5.2 Hz, 1H), 3.99–3.95 (m, 2H), 3.84 (dd, *J* = 9.5, 5.6 Hz, 1H), 3.74–3.70 (m, 2H). ^**13**^ **C NMR** (126 MHz, DMSO-*d*_6_) δ 166.0, 153.6, 137.3, 136.2, 130.7, 126.0, 118.6, 70.4, 67.7, 63.5, 49.0, 48.1, 46.9. **HRMS** (ESI): *m*/*z* calculated for C_15_H_15_N_3_O_4_S + H^+^: 334.0862 [M + H]^+^, found: 334.0910.

#### Characterization Data of (*R*)-((2-Oxo-3-(4-(3-oxomorpholino)­phenyl)­oxazolidin-5-yl)­methyl)­(trifluoromethyl)­carbamoyl Fluoride (**18**)

^**1**^ **H NMR** (500 MHz, acetone-*d*_6_) δ 7.66–7.59 (m, 2H), 7.47–7.39 (m, 2H), 5.04 (tdd, *J* = 9.2, 5.6, 3.3 Hz, 1H), 4.37 (t, *J* = 9.1 Hz, 1H), 4.27–4.19 (m, 2H), 4.15 (ddt, *J* = 14.0, 3.6, 1.9 Hz, 2H), 4.03 (t, *J* = 5.0 Hz, 2H), 4.01–3.90 (m, 1H), 3.80 (dd, *J* = 6.0, 4.1 Hz, 2H). ^**19**^ **F NMR** (471 MHz, acetone-*d*_6_) δ −8.9 (s, 1F), −56.8 (s, 3F). ^**13**^ **C NMR** (126 MHz, acetone-*d*_6_) δ 165.9, 153.5, 142.8 (d, *J =* 299.8 Hz), 137.8, 136.8, 125.9, 119.9 (q, *J =* 262.8 Hz), 118.3, 69.9, 68.1, 63.9, 49.3, 48.9, 47.2. **HRMS** (ESI): *m*/*z* calculated for C_16_H_15_F_4_N_3_O_5_ + H^+^: 406.1026 [M + H]^+^, found: 406.1058.

#### (*R*)-*N*-((2-Oxo-3-(4-(3-oxomorpholino)­phenyl)­oxazolidin-5-yl)­methyl)-*N*-trifluoromethyl Acetamide (**10a**)

The reaction was performed on a 0.19 mmol scale and stirred at 0 °C for 40 min, using (*R*)-((2-oxo-3-(4-(3-oxomorpholino)­phenyl)­oxazolidin-5-yl)­methyl)­(trifluoromethyl)­carbamoyl fluoride (**18**) (80 mg, 0.19 mmol, 1 equiv) and methylmagnesium chloride 3 M in THF (0.14 mL, 0.44 mmol, 2.3 equiv), following general procedure A. The title product was obtained as a beige solid (14 mg, 30 μmol, 19%) after purification via reversed-phase preparative HPLC, with a Waters Sunfire C18 5 μm ODB 19 × 150 mm column and water/acetonitrile in 0.015% difluoroacetic acid (pH 3) as the eluent phase, following the gradient: 3–95%. **^1^H NMR** (500 MHz, DMSO-*d*_6_) δ7.60–7.52 (m, 2H), 7.46–7.38 (m, 2H), 4.81 (tdd, *J* = 8.9, 6.0, 3.3 Hz, 1H), 4.19 (d, *J* = 2.9 Hz, 3H), 4.06–3.89 (m, 4H), 3.78 (dd, *J* = 9.3, 6.1 Hz, 1H), 3.74–3.68 (m, 2H), 2.31 (s, 3H). ^**19**^ **F NMR** (471 MHz, DMSO-*d*_6_) δ −51.9 (s, 3F). ^**13**^ **C NMR** (126 MHz, DMSO-*d*_6_) δ 169.9, 166.1, 153.9, 137.3, 136.5, 126.1, 121.4 (q, *J =* 261.7 Hz), 118.6, 70.8, 67.9, 63.6, 49.2, 47.4, 46.4, 23.3. **HRMS** (ESI): *m*/*z*calculated for C_17_H_18_F_3_N_3_O_5_+ H^+^: 402.1273 [M + H]^+^, found: 402.1298

#### (*R*)-*N*-((2-Oxo-3-(4-(3-oxomorpholino)­phenyl)­oxazolidin-5-yl)­methyl)-*N*-(trifluoromethyl) Cyclopropanecarboxamide (**10b**)

The reaction was performed on a 0.19 mmol scale and stirred at −78 °C for 80 min, using (*R*)-((2-oxo-3-(4-(3-oxomorpholino)­phenyl)­oxazolidin-5-yl)­methyl)­(trifluoro-methyl) carbamoyl fluoride (**18**) (80 mg, 0.19 mmol, 1 equiv) and cyclopropylmagnesium bromide 0.5 M in THF (0.85 mL, 0.44 mmol, 2.3 equiv), following general procedure A. The title product was obtained as an ocher viscous oil (7 mg, 0.02 mmol, 8%) after purification via reversed-phase preparative HPLC with a Waters XBridge C18 5 μm ODB 19 × 150 mm column and water/acetonitrile in 0.01 M NH_4_HCO_3_ (pH 9) as the eluent phase, following the gradient: 5–95%. ^**1**^ **H NMR** (500 MHz, DMSO-*d*_6_) δ 7.60–7.52 (m, 2H), 7.46–7.37 (m, 2H), 4.81 (tdd, *J* = 8.9, 5.9, 3.3 Hz, 1H), 4.24–4.15 (m, 3H), 4.14–4.07 (m, 1H), 4.04–3.94 (m, 3H), 3.78 (dd, *J* = 9.3, 6.0 Hz, 1H), 3.74–3.68 (m, 2H), 2.14–2.02 (m, 1H), 1.10–0.88 (m, 4H). ^**19**^ **F NMR** (471 MHz, DMSO-*d*_6_) δ −50.2 (s, 3F). ^**13**^ **C NMR** (126 MHz, DMSO-*d*_6_) δ 173.1, 166.0, 153.7, 137.2, 136.4, 125.9, 120.5 (q, *J =* 261.6 Hz), 118.4, 70.7, 67.7, 63.5, 49.0, 47.2, 46.1, 13.0, 9.8. **HRMS** (ESI): *m*/*z* calculated for C_19_H_20_F_3_N_3_O_5_ + H^+^: 428.1433 [M + H]^+^, found: 428.1474.

#### (*R*)-*N*-((2-Oxo-3-(4-(3-oxomorpholino)­phenyl)­oxazolidin-5-yl)­methyl)-2-phenyl-*N*-(trifluoromethyl)­acetamide (**10c**)

The reaction was performed on a 0.17 mmol scale and stirred at −78 °C for 10 min, using (*R*)-((2-oxo-3-(4-(3-oxomorpholino)­phenyl)­oxazolidin-5-yl)­methyl)­(trifluoro-methyl) carbamoyl fluoride (**18**) (70 mg, 0.17 mmol, 1 equiv) and benzylmagnesium chloride 1 M in Me-THF (0.2 mL, 0.2 mmol, 1.3 equiv) in a solvent mixture 1,3-dimethyl-3,4,5,6-tetrahydro-2­(1*H*)-pyrimidinone (0.5 mL)/THF (0.7 mL), following general procedure A. The title product was obtained as a colorless solid (51 mg, 0.11 mmol, 63%) after purification via reversed-phase preparative HPLC, with a Waters Sunfire C18 5 μm ODB 19 × 150 mm column and water/acetonitrile in 0.015% DFA (pH 3) as the eluent phase, following the gradient: 3–95%. ^**1**^ **H NMR** (500 MHz, DMSO-*d*_6_) δ 7.60–7.53 (m, 2H), 7.45–7.40 (m, 2H), 7.35–7.30 (m, 2H), 7.29–7.22 (m, 3H), 4.86 (tdd, *J* = 9.0, 6.0, 3.1 Hz, 1H), 4.20 (m, 3H), 4.16–4.09 (m, 1H), 4.03–3.94 (m, 5H), 3.79 (dd, *J* = 9.3, 6.2 Hz, 1H), 3.74–3.69 (m, 2H). ^**19**^ **F NMR** (471 MHz, DMSO-*d*_6_) δ −50.6 (s, 3F). ^**13**^ **C NMR** (126 MHz, DMSO-*d*_6_) δ 171.0, 166.0, 153.7, 137.2, 136.4, 133.8, 129.6, 128.3, 126.9, 126.0, 121.2 (q, *J =* 260.8 Hz), 118.4, 70.8, 67.7, 63.5, 49.0, 47.3, 46.5, 40.6. **HRMS** (ESI): *m*/*z* calculated for C_23_H_22_F_3_N_3_O_5_ + H^+^: 478.1582 [M + H]^+^, found: 478.1618.

#### (*R*)-*N*-((2-Oxo-3-(4-(3-oxomorpholino)­phenyl)­oxazolidine-5-yl)­methyl)-*N*-trifluoromethyl Benzamide (**10d**)

The reaction was performed on a 0.19 mmol scale and stirred at −78 °C for 10 min, using (*R*)-((2-oxo-3-(4-(3-oxomorpholino)­phenyl)-oxazolidin-5-yl)­methyl)­(trifluoro-methyl)­carbamoyl fluoride (**18**) (80 mg, 0.19 mmol, 1 equiv) and phenylmagnesium bromide 3 M in THF (0.16 mL, 0.49 mmol, 2.6 equiv), following g eneral procedure A. The title product was obtained as a colorless solid (6 mg, 0.01 mmol, 5%) after purification via reversed-phase preparative HPLC with a Waters Sunfire C18 5 μm ODB 19 × 150 mm column and water/acetonitrile in 0.015% DFA (pH 3) as the eluent phase, following the gradient: 3–95%. ^**1**^ **H NMR** (500 MHz, DMSO-*d*_6_) δ 7.60–7.54 (m, 3H), 7.53–7.47 (m, 4H), 7.43–7.36 (m, 2H), 4.90 (tq, *J* = 8.7, 4.1 Hz, 1H), 4.18 (m, 3H), 4.11–3.92 (m, 4H), 3.74 (dd, *J* = 9.4, 5.8 Hz, 1H), 3.72–3.66 (m, 2H). ^**19**^ **F NMR** (471 MHz, DMSO-*d*_6_) δ −51.2 (s, 3F). ^**13**^ **C NMR** (126 MHz, DMSO-*d*_6_) δ 170.5, 166.1, 153.7, 137.4, 136.4, 134.4, 131.6, 128.7, 127.4, 126.1, 120.2 (q, *J =* 262.7 Hz), 118.6, 70.5, 67.9, 63.6, 49.2, 48.2, 47.4. **HRMS** (ESI): *m*/*z* calculated for C_22_H_20_F_3_N_3_O_5_ + H^+^: 464.1433 [M + H]^+^, found: 464.1434.

#### (*S*)-*N*-Methyl-*N*-((2-oxo-3-(4-(3-oxomorpholino)­phenyl)­oxazolidin-5-yl)­methyl)­acetamide (**10e**)

In a heat gun-dried vial, (*S*)-4-(4-(5-(aminomethyl)-2-oxooxazolidin-3-yl)­phenyl)­morpholin 3-one hydrochloride (**17**) (0.45 g, 1.5 mmol, 1 equiv) was suspended in dichloromethane (6 mL), and triethylamine (281 μL, 1.98 mmol, 1.3 equiv) was added. The solution was cooled to 0 °C and stirred for 5 min. In a parallel heat gun-dried vial, acetyl chloride (0.10 mL, 1.5 mmol, 1 equiv) was dissolved in dichloromethane (2 mL) and added dropwise at 0 °C. After stirring for 15 min, the mixture was warmed up to room temperature. After 16 h, the mixture was filtered, and the solvent was removed *in vacuo* at 43 °C. The resulting oily crude was dissolved in DMSO (0.6 mL) and purified via reversed-phase chromatography, with a Biotage Sfär C18 D 30 g column and 0.1% formic acid in water/acetonitrile as the eluent phase, following the gradient: 5–5% 3 CV; 5–35% 15 CV; 35–95% 3 CV, delivering (*S*)-*N*-((2-oxo-3-(4-(3-oxomorpholino)­phenyl)-oxazolidin-5-yl)­methyl)­acetamide (0.22 g, 0.66 mmol, 44%).

Subsequently, a heat gun-dried flask was charged with the newly obtained amide (0.22 g, 0.66 mmol, 1 equiv) in dioxane (85 mL). The solution was cooled to 0 °C, and, after 15 min, a 1 M solution of potassium bis­(trimethylsilyl)­amide in THF (1.3 mL, 1.3 mmol, 2 equiv) was added dropwise. The mixture was stirred for an additional 15 min at 0 °C, and, subsequently, iodomethane (0.62 mL, 10 mmol, 15 equiv) was added dropwise. After 15 min at 0 °C, the mixture was warmed up to room temperature. After 22 h, the mixture was quenched with acetic acid (60 μL), and the solvent was removed *in vacuo* at 43 °C. The resulting crude was dissolved in DMSO (0.6 mL) and purified via reversed-phase HPLC, with a Waters XBridge C18 5 μm ODB 19 × 150 mm column and water/acetonitrile in 0.01 M NH_4_HCO_3_ (pH 9) as the eluent phase, following the gradient: 5–95%, delivering the title product (83 mg, 0.24 mmol, 36%).

#### Characterization Data of (*S*)-*N*-((2-Oxo-3-(4-(3-oxomorpho-lino)­phenyl)­oxazolidin-5-yl)­methyl)­acetamide

^**1**^ **H NMR** (500 MHz, DMSO-*d*_6_) δ 8.26 (t, *J* = 5.9 Hz, 1H), 7.59–7.53 (m, 2H), 7.44–7.37 (m, 2H), 4.72 (dq, *J* = 9.0, 5.4 Hz, 1H), 4.19 (s, 2H), 4.13 (t, *J* = 8.9 Hz, 1H), 3.97 (dd, *J* = 6.0, 4.2 Hz, 2H), 3.76 (dd, *J* = 9.1, 6.4 Hz, 1H), 3.71 (dd, *J* = 6.0, 4.2 Hz, 2H), 3.42 (t, *J* = 5.5 Hz, 2H), 1.84 (s, 3H). ^**13**^ **C NMR** (126 MHz, DMSO-*d*_6_) δ 170.0, 166.0, 154.1, 137.0, 136.5, 126.0, 118.3, 71.5, 67.7, 63.5, 49.0, 47.3, 41.4, 22.4. **HRMS** (ESI): *m*/*z* calculated for C_16_H_19_N_3_O_5_ + H^+^: 334.1403 [M + H]^+^, found: 334.1441.

#### Characterization Data of (*S*)-*N*-Methyl-*N*-((2-oxo-3-(4-(3-oxomorpholino)­phenyl)­oxazolidin-5-yl)­methyl)­acetamide (**10e**)

^**1**^ **H NMR** (500 MHz, DMSO-*d*_6_) δ 7.61–7.54 (m, 2H), 7.44–7.39 (m, 2H), 4.98–4.79 (m, 1H), 4.22–4.10 (m, 3H), 3.97 (ddd, *J* = 6.4, 3.9, 1.3 Hz, 2H), 3.85–3.55 (m, 5H), 3.07 (s, 2H), 2.88 (s, 1H), 2.03 (d, *J* = 9.0 Hz, 3H). ^**13**^ **C NMR** (126 MHz, DMSO-*d*_6_) δ 170.8, 170.0, 166.0, 154.1, 153.9, 137.1, 136.5, 125.9, 118.4, 118.4, 71.6, 71.1, 67.7, 63.5, 52.9, 49.7, 49.0, 47.4, 37.3, 33.4, 21.6. **HRMS** (ESI): *m*/*z* calculated for C_17_H_21_N_3_O_5_ + H^+^: 348.1554 [M + H]^+^, found: 348.1552.

#### (*S*)-*N*-Isopropyl-*N*-((2-oxo-3-(4-(3-oxomorpholino)­phenyl)­oxazolidin-5-yl)­methyl)­acetamide (**10f**)

The reaction was carried out on a 0.15 mmol scale, using acetyl chloride (14 μL, 0.19 mmol, 1.3 equiv), following general procedure D. The title product was obtained as a colorless viscous oil (25 mg, 67 μmol, 44%) after purification via reversed-phase preparative HPLC with a Kromasil C8 column (10 μm, 250 mm × 20 mm) and (water/acetonitrile/formic acid 95/5/0.2)/acetonitrile as the eluent phase, following the reported gradient: 10–70%. ^**1**^ **H NMR** (500 MHz, CDCl_3_) δ 7.60–7.54 (m, 2H), 7.35–7.28 (m, 2H), 4.81 (dddd, *J* = 8.6, 7.2, 6.0, 4.2 Hz, 1H), 4.30 (s, 2H), 4.16–4.10 (m, 1H), 4.09–3.96 (m, 3H), 3.83 (dd, *J* = 9.4, 7.2 Hz, 1H), 3.76–3.68 (m, 3H), 3.30 (dd, *J* = 14.5, 6.0 Hz, 1H), 2.15 (s, 3H), 1.29 (d, *J* = 6.7 Hz, 3H), 1.18 (d, *J* = 6.7 Hz, 3H). ^**13**^ **C NMR** (126 MHz, CDCl_3_) δ 171.6, 166.9, 154.4, 137.1, 137.0, 126.2, 119.0, 72.5, 68.6, 64.2, 49.7, 49.7, 49.7, 45.3, 22.0, 21.7, 21.2. **HRMS** (ESI): *m*/*z* calculated for C_19_H_25_N_3_O_5_ + H^+^: 376.1828 [M + H]^+^, found: 376.1842.

#### (*S*)-*N*-Methyl-*N*-((2-oxo-3-(4-(3-oxomorpholino)­phenyl)­oxazolidin-5-yl)­methyl)­cyclopropanecarboxamide (**10g**)

In a heat gun-dried vial, (*S*)-4-(4-(5-(aminomethyl)-2-oxooxazolidin-3-yl)­phenyl)-morpholin-3-one hydrochloride (**17**) (0.20 g, 0.61 mmol, 1 equiv) was suspended in dichloromethane (1.7 mL), and triethylamine (0.1 mL, 0.8 mmol, 1.3 equiv) was added. The solution was cooled to 0 °C and stirred for 5 min. In a parallel heat gun-dried vial, cyclopropanecarboxylic acid chloride (60 μL, 0.61 mmol, 1 equiv) was dissolved in dichloromethane (2 mL) and added dropwise at 0 °C. After stirring for 15 min, the mixture was warmed up to room temperature. After 2 h, the mixture was filtered, and the solvent was removed*in vacuo*at 43 °C. The resulting oily crude was dissolved in DMSO (0.5 mL) and purified via reversed-phase chromatography, with a BiotageSfär C18 D 30 g column and 0.1% formic acid in water/acetonitrile as the eluent phase, following the gradient: 5–5% 3 CV, 5–35% 15 CV, 35–100% 3 CV, 100–100% 3 CV. The (*S*)-*N*-((2-oxo-3-(4-(3-oxomorpholino)­phenyl)­oxazolidin-5-yl)­methyl)­cyclopropanecarboxamide was obtained as a colorless solid (74 mg, 0.21 mmol, 34%).

Subsequently, the newly obtained amide (74 mg, 210 μmol, 1 equiv) was added to a heat gun-dried flask and dissolved in dioxane (27 mL). The solution was cooled to 0 °C, and after 15 min, a 1 M solution of potassium bis­(trimethylsilyl)­amide in THF (0.46 mL, 0.46 mmol, 2.2 equiv) was added dropwise. The mixture was stirred for another 15 min at 0 °C, and, subsequently, iodomethane (0.19 mL, 3.1 mmol, 15 equiv) was added dropwise. After 15 min at 0 °C, the mixture was warmed up to room temperature. After 22 h, the mixture was quenched with acetic acid (30 μL), and the solvent was removed *in vacuo* at 43 °C. The resulting crude was dissolved in DMSO (0.5 mL) and purified via reversed-phase HPLC, with a Waters XBridge C18 5 μm ODB 19 × 150 mm column and water/acetonitrile in 0.01 M NH_4_HCO_3_ (pH 9) as the eluent phase, following the gradient 5–95%, delivering the title product (17 mg, 42 μmol, 21%).

#### Characterization Data of (*S*)-*N*-((2-Oxo-3-(4-(3-oxomorpholino)­phenyl)­oxazolidin-5-yl)­methyl)­cyclopropanecarboxamide

^**1**^ **H NMR** (500 MHz, DMSO-*d*_6_) δ 8.46 (t, *J* = 6.5 Hz, 1H), 7.59–7.53 (m, 2H), 7.44–7.38 (m, 2H), 4.73 (dq, *J* = 11.4, 5.4 Hz, 1H), 4.19 (s, 2H), 4.13 (t, *J* = 8.9 Hz, 1H), 3.97 (dd, *J* = 6.0, 4.2 Hz, 2H), 3.79–3.69 (m, 3H), 3.45 (t, *J* = 5.9 Hz, 2H), 1.60 (ddd, *J* = 12.6, 7.1, 4.6 Hz, 1H), 0.72–0.59 (m, 4H). ^**13**^ **C NMR** (126 MHz, DMSO-*d*_6_) δ 173.5, 166.0, 154.2, 137.0, 136.5, 126.0, 118.3, 71.6, 67.7, 63.5, 49.0, 47.3, 45.6, 41.6, 13.4, 6.5, 6.4. **HRMS** (ESI): *m*/*z* calculated for C_18_H_21_N_3_O_5_ + H^+^: 360.1559 [M + H]^+^, found: 360.1599.

#### Characterization Data of (*S*)-*N*-Methyl-*N*-((2-oxo-3-(4-(3-oxomorpholino)­phenyl)­oxazolidin-5-yl)­methyl)­cyclopropanecarboxamide (**10g**)

^**1**^ **H NMR** (500 MHz, DMSO-*d*_6_) δ 7.61–7.53 (m, 2H), 7.45–7.37 (m, 2H), 4.99–4.80 (m, 1H), 4.23–4.10 (m, 3H), 3.97 (dd, *J* = 6.0, 4.1 Hz, 2H), 3.87–3.60 (m, 5H), 3.24 (s, 2H), 2.91 (s, 1H), 2.06–1.90 (m, 1H), 0.80–0.65 (m, 4H). ^**13**^ **C NMR** (126 MHz, DMSO-*d*_6_) δ 173.4, 172.8, 166.0, 154.1, 154.0, 137.1, 136.5, 136.5, 129.6, 125.9, 118.3, 71.6, 71.4, 67.7, 63.5, 52.0, 50.4, 49.0, 47.5, 47.4, 36.7, 34.4, 22.1, 10.7, 10.6, 7.5, 7.3. **HRMS** (ESI): *m*/*z* calculated for C_19_H_23_N_3_O_5_ + H^+^: 374.1710 [M + H]^+^, found: 374.1719.

#### (*S*)-*N*-Isopropyl-*N*-((2-oxo-3-(4-(3-oxomorpholino)­phenyl)­oxazolidin-5-yl)­methyl)­cyclopropanecarboxamide (**10h**)

The reaction was carried out on a 0.15 mmol scale, using cyclopropanecarboxylic acid chloride (17 μL, 0.19 mmol, 1.3 equiv), following general procedure D. The title product was obtained as a yellow viscous oil (50 mg, 0.13 mmol, 83%) after purification via reversed-phase preparative HPLC, with a Kromasil C8 column (10 μm, 250 mm × 20 mm) and (water/acetonitrile/formic acid 95/5/0.2)/acetonitrile as the eluent phase, following the gradient: 0–60%. ^**1**^ **H NMR** (500 MHz, DMSO-*d*_6_) δ 7.57 (d, *J* = 8.4 Hz, 2H), 7.40 (d, *J* = 8.4 Hz, 2H), 4.97–4.75 (m, 1H), 4.58–4.32 (m, 1H), 4.19 (s, 3H), 4.03–3.37 (m, 7H), 2.08–1.88 (m, 1H), 1.31–1.09 (m, 6H), 0.88–0.62 (m, 4H). ^**13**^ **C NMR** (151 MHz, DMSO-*d*_6_) δ 173.3, 172.7, 166.0, 154.1, 137.0, 136.6, 125.9, 118.3, 72.7, 71.4, 67.7, 63.5, 49.0, 48.2, 47.7, 46.8, 46.6, 44.7, 21.4, 21.1, 20.1, 19.8, 11.7, 11.2, 7.8, 7.4, 7.2, 7.1. **HRMS** (ESI): *m*/*z* calculated for C_21_H_27_N_3_O_5_ + H^+^: 402.2029 [M + H]^+^, found: 402.2058.

#### (4-(Allyloxy)­phenyl)­(trifluoromethyl)­carbamoyl Fluoride (**20**)

A heat gun-dried vial was charged with 4-(allyloxy)­aniline (**19**) (0.62 g, 4.1 mmol, 1 equiv) and dissolved in dry dichloromethane (12 mL). Then, triethylamine (1.2 mL, 8.2 mmol, 2 equiv) was added, and the mixture was stirred for 15 min. Subsequently, 1,1′-Thiocarbonyldiimidazole (1,1′-TCDI) (0.96 g, 5.4 mmol, 1.3 equiv) was added in one portion, and the mixture was stirred for 16 h. Then, an aqueous saturated ammonium chloride solution (4 mL) was added, and the mixture was left to stir for 2–3 min. The organic phase was separated, and the aqueous phase was extracted with dichloromethane (2 × 30 mL). The combined organic layers were washed with brine (10 mL) and dried over a phase separator, and the solvent was removed *in vacuo* at 43 °C. The obtained crude was purified via normal-phase automated flash chromatography with a Biotage Sfär HC Duo 25 g column (sample dissolved in dichloromethane and loaded on a Samplet; the Samplet was dried *in vacuo* for 30 min) and *n*-heptane/EtOAc as the eluent phase, following the gradient: 5–5% 1 CV; 5–9% 1.2 CV; 9–9% 11.1 CV; 9–40% 8.8 CV; 40–40% 2 CV. 1-(Allyloxy)-4-isothiocyanatobenzene was obtained as an ocher oil (0.65 g, 3.4 mmol, 82%).

Subsequently, a heat gun-dried vial was charged with silver­(I) fluoride (1.71 g, 13.5 mmol, 12.5 equiv) and bis­(trichloromethyl) carbonate (0.13 g, 0.43 mmol, 0.4 equiv), and the vial was subsequently sealed, evacuated, and filled with Ar (the vacuum-Ar cycle was repeated 3 times). In a parallel pear-shaped flask, previously obtained 1-(allyloxy)-4-isothiocyanatobenzene (0.21 g, 1.1 mmol, 1 equiv) was diluted in dry acetonitrile (10 mL) and quickly transferred to the other vial. The suspension was vigorously stirred for 20 h at room temperature. Then, the obtained dark brown suspension was diluted with diethyl ether (8 mL) and further stirred for 15 min. The suspension was filtered through Celite and a phase separator to remove the last traces of salt byproducts. Then, the solvent mixture was removed *in vacuo* at 43 °C, and the crude was purified via normal-phase automated flash chromatography with a Biotage Sfär HC Duo 50 g column (sample dissolved in dichloromethane and loaded on a Samplet support; the Samplet was dried *in vacuo* for 30 min) and *n*-heptane/EtOAc as the eluent phase, following the gradient: 0–2% 2 CV; 2–2% 2 CV; 2–13% 4.1 CV; 13–13% 1 CV; 13–50% 12.9 CV; 50–50% 1 CV; 50–100% 10 CV. The title product was obtained as a pale yellow oil (0.24 g, 0.89 mmol, 83%).

#### Characterization Data of 1-(Allyloxy)-4-isothiocyanatobenzene

^**1**^ **H NMR** (500 MHz, CDCl_3_) δ 7.19–7.12 (m, 2H), 6.91–6.82 (m, 2H), 6.03 (ddt, *J* = 17.3, 10.5, 5.3 Hz, 1H), 5.41 (dq, *J* = 17.3, 1.6 Hz, 1H), 5.31 (dq, *J* = 10.6, 1.4 Hz, 1H), 4.53 (dt, *J* = 5.3, 1.5 Hz, 2H). ^**13**^ **C NMR** (126 MHz, CDCl_3_) δ 157.7, 134.1, 132.7, 127.1, 123.8, 118.2, 115.7, 69.2. *Note*: No HRMS data available due to lack of ionization.

#### Characterization Data of (4-(Allyloxy)­phenyl)­(trifluoromethyl)­carbamoyl Fluoride (**20**)

^**1**^ **H NMR** (500 MHz, CDCl_3_) δ 7.25–7.19 (m, 2H), 7.00–6.96 (m, 2H), 6.05 (ddt, *J* = 17.3, 10.6, 5.3 Hz, 1H), 5.43 (dq, *J* = 17.3, 1.6 Hz, 1H), 5.33 (dq, *J* = 10.5, 1.4 Hz, 1H), 4.57 (dt, *J* = 5.3, 1.5 Hz, 2H). ^**19**^ **F NMR** (471 MHz, DMSO-*d*_6_) δ −2.3 (s, 1F), – 56.7 (s, 3F). ^**13**^ **C NMR** (126 MHz, CDCl_3_) δ 160.0, 142.5 (d, *J =* 299.9 Hz), 132.6, 129.6, 125.8, 119.5 (q, *J =* 264.0 Hz), 118.2, 115.9, 69.2. *Note*: No HRMS data available due to lack of ionization.

#### *N*-(4-Hydroxyphenyl)-*N*-(trifluoromethyl)­acetamide (**11a**)

The reaction was performed on a 0.34 mmol scale and stirred at −78 °C for 40 min using (4-(allyloxy)­phenyl)­(trifluoromethyl)­carbamoyl fluoride (**20**) (90 mg, 0.34 mmol, 1 equiv) and methylmagnesium chloride 3 M in THF (0.15 mL, 0.44 mmol, 1.3 equiv) in toluene (2 mL). The obtained crude (50 mg, 0.26 mmol, 1 equiv) was reacted with tetrakis­(triphenyl-phosphine)­palladium(0) (12 mg, 10 μmol, 5.5 mol %) and 1,3-dimethylbarbituric acid (80 mg, 0.52 mmol, 1.3 equiv) in dichloromethane (1.2 mL), following general procedure B. The title product was obtained as a colorless viscous oil (21 mg, 99 μmol, 29% over 2 steps) after purification via reversed-phase preparative HPLC, with a Waters XBridge C18 5 μm ODB 19 × 150 mm column and water/acetonitrile in 0.01 M NH_4_HCO_3_ (pH 9) as the eluent phase, following the gradient: 5–95%. ^**1**^ **H NMR** (500 MHz, DMSO-*d*_6_) δ 9.99 (s, 1H), 7.25–7.20 (m, 2H), 6.88–6.83 (m, 2H), 1.85 (s, 3H). ^**19**^ **F NMR** (471 MHz, DMSO-*d*_6_) δ −54.1 (s, 3F). ^**13**^ **C NMR** (126 MHz, DMSO-*d*_6_) δ 170.7, 158.5, 130.5, 126.6, 120.0 (q, *J =* 262.5 Hz), 116.3, 23.9. **HRMS** (ESI): *m*/*z* calculated for C_9_H_8_F_3_NO_2_ + H^+^: 220.0583 [M + H]^+^, found: 220.0569.

#### *N*-(4-Hydroxyphenyl)-*N*-(trifluoromethyl)­cyclopropane Carboxamide (**11b**)

The reaction was performed on a 0.30 mmol scale and stirred at −78 °C for 30 min, using (4-(allyloxy)­phenyl)­(trifluoromethyl)­carbamoyl fluoride (**20**) (80 mg, 0.30 mmol, 1 equiv) and cyclopropylmagnesium bromide 0.5 M in THF (0.80 mL, 0.40 mmol, 1.3 equiv) in toluene (2.2 mL); the crude (50 mg, 0.16 mmol, 1 equiv) was then reacted with tetrakis­(triphenylphosphine)­palladium(0) (10 mg, 8.7 μmol, 5.5 mol %) and 1,3-dimethylbarbituric acid (30 mg, 0.21 mmol, 1.3 equiv) in dichloromethane (0.8 mL), following general procedure B. The title product was obtained as an ocher viscous oil (16 mg, 64 μmol, 21% over 2 steps) after purification via reversed-phase preparative HPLC, with a Waters XBridge C18 5 μm ODB 19 × 150 mm column and water/acetonitrile in 0.01 M NH_4_HCO_3_ (pH 9) as the eluent phase, following the gradient: 5–95%. ^**1**^ **H NMR** (500 MHz, DMSO-*d*_6_) δ 10.02 (s, 1H), 7.35–7.18 (m, 2H), 7.00–6.76 (m, 2H), 1.31 (tt, *J* = 7.9, 4.6 Hz, 1H), 0.87 (dt, *J* = 4.7, 3.2 Hz, 2H), 0.77 (dt, *J* = 8.3, 3.4 Hz, 2H). ^**19**^ **F NMR** (471 MHz, DMSO-*d*_6_) δ −54.3 (s, 3F). ^**13**^ **C NMR** (126 MHz, DMSO-*d*_6_) δ 173.6, 158.6, 130.8, 125.9, 119.8 (q, *J =* 262.9 Hz), 116.5, 13.6, 9.6. **HRMS** (ESI): *m*/*z* calculated for C_11_H_10_F_3_NO_2_ + H^+^: 246.0734 [M + H]^+^, found: 246.0749.

#### *N*-(4-Hydroxyphenyl)-2-phenyl-*N*-(trifluoromethyl)­acetamide (**11c**)

The reaction was performed on a 0.52 mmol scale and stirred at 0 °C for 1 h, using (4-(allyloxy)­phenyl)­(trifluoromethyl)­carbamoyl fluoride (**20**) (0.14 g, 0.52 mmol, 1 equiv) and benzylmagnesium chloride 1 M in Me-THF (2.2 mL, 2.2 mmol, 4.3 equiv) in toluene (3.4 mL); the crude (40 mg, 0.11 mmol, 1 equiv) was then reacted with tetrakis­(triphenylphosphine)­palladium(0) (7 mg, 6 μmol, 5.5 mol %) and 1,3-dimethylbarbituric acid (20 mg, 0.14 mmol, 1.3 equiv) in dichloromethane (0.6 mL), following general procedure B. The title product was obtained as a gray viscous oil (9 mg, 0.03 mmol, 6% over 2 steps) after purification via reversed-phase preparative HPLC, with a Waters XBridge C18 5 μm ODB 19 × 150 mm column and water/acetonitrile in 0.01 M NH_4_HCO_3_ (pH 9) as the eluent phase, following the gradient: 5–95%. ^**1**^ **H NMR** (500 MHz, DMSO-*d*_6_), δ 10.02 (s, 1H), 7.31–7.20 (m, 5H), 7.08–7.02 (m, 2H), 6.90–6.84 (m, 2H), 3.46 (s, 2H). ^**19**^ **F NMR** (471 MHz, DMSO-*d*_6_) δ −54.3 (s, 3F). ^**13**^ **C NMR** (126 MHz, DMSO-*d*_6_) δ 171.3, 158.6, 133.7, 130.9, 129.4, 128.2, 126.8, 125.7, 120.0 (q, *J =* 262.7 Hz), 116.3, 41.4. **HRMS** (ESI): *m*/*z* calculated for C_15_H_12_F_3_NO_2_ + H^+^: 296.0893 [M + H]^+^, found: 296.0919.

#### *N*-(4-Hydroxyphenyl)-*N*-(trifluoromethyl)­benzamide (**11d**)

The reaction was performed on a 0.30 mmol scale and stirred at −78 °C for 30 min, using (4-(allyloxy)­phenyl)­(trifluoromethyl)­carbamoyl fluoride (**20**) (80 mg, 0.30 mmol, 1 equiv) and phenylmagnesium bromide 3 M in THF (0.13 mL, 0.40 mmol, 1.3 equiv), in toluene (2 mL); the crude (60 mg, 0.18 mmol, 1 equiv) was then reacted with tetrakis­(triphenylphosphine)­palladium(0) (11 mg, 10 μmol, 5.5 mol %) and 1,3-dimethylbarbituric acid (40 mg, 0.23 mmol, 1.3 equiv) in dichloromethane (0.9 mL), following general procedure **B**. The title product was obtained as colorless viscous oil (24 mg, 80 μmol, 27% over 2 steps) after purification via reversed-phase preparative HPLC, with a Waters XBridge C18 5 μm ODB 19 × 150 mm column and water/acetonitrile in 0.01 M NH_4_HCO_3_ (pH 9) as the eluent phase, following the gradient: 5–95%. ^**1**^ **H NMR** (500 MHz, DMSO-*d*_6_) δ 9.86 (s, 1H), 7.53–7.47 (m, 2H), 7.41–7.35 (m, 1H), 7.32–7.27 (m, 2H), 7.22–7.16 (m, 2H), 6.71–6.65 (m, 2H). ^**19**^ **F NMR** (471 MHz, DMSO-*d*_6_) δ −56.6 (s, 3F). ^**13**^ **C NMR** (126 MHz, DMSO-*d*_6_) δ 170.0, 158.0, 133.6, 131.4, 131.4, 128.6, 128.1, 126.6, 120.4 (q, *J =* 262.9 Hz), 116.0. **HRMS** (ESI): *m*/*z* calculated for C_14_H_10_F_3_NO_2_ + H^+^: 282.0734 [M + H]^+^, found: 282.0772.

#### 2-(2-Methyl-5-nitro-1*H*-imidazol-1-yl)­ethylmethyl­(trifluoro-methyl)­carbamate (**12a**)

The reaction was performed on a 0.15 mmol scale and stirred for 20 h, using 2-(2-methyl-5-nitro-1*H*-imidazol-1-yl)­ethan-1-ol (**21**) (26 mg, 0.15 mmol, 1 equiv) and methyl­(trifluoromethyl)­carbamoyl fluoride (**S2**) (98 mg, 0.68 mmol, 4.9 equiv) in acetonitrile (6.8 mL), following general proced ure C. The title product was obtained as a colorless solid (10 mg, 34 μmol, 25%) after purification via reversed-phase preparative HPLC, with a Waters Sunfire C18 5 μm ODB 19 × 150 mm column and water/acetonitrile in 0.015% difluoroacetic acid (pH 3) as the eluent phase, following the gradient: 3–95%. ^**1**^ **H NMR** (500 MHz, DMSO-*d*_6_) δ 8.04 (s, 1H), 4.66 (dd, *J* = 5.6, 4.5 Hz, 2H), 4.52 (dd, *J* = 5.5, 4.4 Hz, 2H), 2.98 (q, *J* = 2.3 Hz, 3H), 2.44 (s, 3H). ^**19**^ **F NMR** (471 MHz, DMSO-*d*_6_) δ −55.8 (s, 3F). ^**13**^ **C NMR** (126 MHz, DMSO-*d*_6_) δ 151.8, 151.6, 138.6, 133.2, 120.5 (q, *J =* 259.9 Hz), 65.1, 44.4, 31.1, 13.8. **HRMS** (ESI): *m*/*z* calculated for C_9_H_11_F_3_N_4_O_4_ + H^+^: 297.1250 [M + H]^+^, found: 297.1275.

#### 2-(2-Methyl-5-nitro-1*H*-imidazol-1-yl)­ethylcyclopropyl­(trifluoromethyl)­carbamate (**12b**)

The reaction was performed on a 0.15 mmol scale and stirred at room temperature for 6 h, using 2-(2-methyl-5-nitro-1*H*-imidazol-1-yl)­ethan-1-ol (**21**) (26 mg, 0.15 mmol, 1 equiv) and cyclopropyl­(trifluoromethyl)­carbamoyl fluoride (**S3**) (50 mg, 0.30 mmol, 2 equiv) in acetonitrile (3 mL), following general procedure C. The title product was obtained as a colorless solid (20 mg, 63 μmol, 42%) after purification via reversed-phase preparative HPLC, with a Waters XBridge C18 5 μm ODB 19 × 150 mm column and water/acetonitrile in 0.01 M NH_4_HCO_3_ (pH 9) as the eluent phase, following the gradient: 5–95%. ^**1**^ **H NMR** (500 MHz, DMSO-*d*_6_) δ 8.05 (s, 1H), 4.66 (dd, *J* = 5.6, 4.5 Hz, 2H), 4.50 (dd, *J* = 5.6, 4.5 Hz, 2H), 2.60 (ttd, *J* = 8.4, 4.2, 2.4 Hz, 1H), 2.45 (s, 3H), 0.85–0.79 (m, 2H), 0.67–0.60 (m, 2H). ^**19**^ **F NMR** (471 MHz, DMSO-*d*_6_) δ −53.5 (s, 3F). ^**13**^ **C NMR** (126 MHz, DMSO-*d*_6_) δ 152.2, 151.6, 138.6, 133.2, 121.0 (q, *J =* 261.0 Hz), 65.0, 44.5, 26.0, 13.9, 8.0. **HRMS** (ESI): *m*/*z* calculated for C_11_H_13_F_3_N_4_O_4_ + H^+^: 323.0967 [M + H]^+^, found: 323.0968.

#### 2-(2-Methyl-5-nitro-1*H*-imidazol-1-yl)­ethylbenzyl­(trifluoro-methyl)­carbamate (**12c**)

The reaction was performed on a 0.17 mmol scale and stirred at room temperature for 4 h using 2-(2-methyl-5-nitro-1*H*-imidazol-1-yl)­ethan-1-ol (**21**) (29 mg, 170 μmol), benzyl­(trifluoromethyl)­carbamoyl fluoride (**S4**) (80 mg, 0.34 mmol, 2 equiv), DIPEA (60 μL, 0.34 mmol, 2 equiv), and 4-dimethylaminopyridine (4 mg, 0.03 mmol, 0.2 equiv) in acetonitrile (3 mL), following general procedu re C. The title product was obtained as a colorless solid (27 mg, 73 μmol, 43%) after purification via reversed-phase preparative HPLC, with a Waters XBridge C18 5 μm ODB 19 × 150 mm column and water/acetonitrile in 0.01 M NH_4_HCO_3_ (pH 9) as the eluent phase, following the gradient: 5–95%. ^**1**^ **H NMR** (500 MHz, DMSO-*d*_6_) δ 8.04 (s, 1H), 7.37–7.27 (m, 3H), 7.19 (dd, *J* = 7.1, 1.8 Hz, 2H), 4.67 (dd, *J* = 9.0, 3.7 Hz, 4H), 4.60 (q, *J* = 4.3 Hz, 2H), 2.34 (s, 3H). ^**19**^ **F NMR** (471 MHz, DMSO-*d*_6_) δ −53.7 (s, 3F). ^**13**^ **C NMR** (126 MHz, DMSO-*d*_6_) δ 152.1, 151.5, 138.6, 136.4, 133.3, 128.7, 127.7, 126.7, 120.7 (q, *J =* 261.3 Hz), 65.4, 47.9, 44.5, 13.7. **HRMS** (ESI): *m*/*z* calculated for C_15_H_15_F_3_N_4_O_4_ + H^+^: 373.1124 [M + H]^+^, found: 373.1152.

#### 2-(2-Methyl-5-nitro-1*H*-imidazol-1-yl)­ethyl Phenyl­(trifluoro-methyl)­carbamate (**12d**)

The reaction was performed on a 0.12 mmol scale and stirred for 1 h using 2-(2-methyl-5-nitro-1*H*-imidazol-1-yl)­ethan-1-ol (**21**) (21 mg, 0.12 mmol, 1 equiv), phenyl­(trifluoromethyl)­carbamoyl fluoride (**S5**) (50 mg, 0.24 mmol, 2 equiv), DIPEA (40 μL, 0.24 mmol, 2 equiv), and 4-dimethylaminopyridine (3 mg, 0.02 mmol, 0.2 equiv) in acetonitrile (2.5 mL), following general procedur e C. The title product was obtained as a colorless solid (18 mg, 49 μmol, 41%) after purification via reversed-phase preparative HPLC, with a Waters Sunfire C18 5 μm ODB 19 × 150 mm column and water/acetonitrile in 0.015% difluoroacetic acid (pH 3) as the eluent phase, following the gradient: 3–95%. ^**1**^ **H NMR** (500 MHz, DMSO-*d*_6_) δ 8.00 (s, 1H), 7.52–7.43 (m, 3H), 7.29 (dt, *J* = 6.6, 1.6 Hz, 2H), 4.54–4.45 (m, 4H), 1.96 (s, 3H). ^**19**^ **F NMR** (470 MHz, DMSO-*d*_6_) δ −54.0 (s, 3F). ^**13**^ **C NMR** (126 MHz, DMSO-*d*_6_) δ 151.5, 151.1, 138.4, 134.2, 133.2, 129.8, 129.7, 128.9, 119.7 (q, *J =* 260.5 Hz), 65.5, 44.3, 13.3. **HRMS** (ESI): *m*/*z* calculated for C_14_H_13_F_3_N_4_O_4_ + H^+^: 359.0967 [M + H]^+^, found: 359.0958.

#### 2-(2-Methyl-5-nitro-1*H*-imidazol-1-yl)­ethyl Dimethylcarbamate (**12e**)

The reaction was carried out on a 0.29 mmol scale, using dimethylamine hydrochloride (50 mg, 0.58 mmol, 2 equiv), following general procedu re E. The title compound was obtained as a colorless oil (5 mg, 0.02 mmol, 7%) after purification via reversed-phase preparative HPLC with a Waters XBridge C18 5 μm ODB 19 × 150 mm column and water/acetonitrile in 0.01 M NH_4_HCO_3_ (pH 9) as the eluent phase, following the gradient: 5–95%. ^**1**^ **H NMR** (600 MHz, DMSO-*d*_6_) δ 8.04 (s, 1H), 4.59 (t, *J* = 5.1 Hz, 2H), 4.31 (t, *J* = 5.1 Hz, 2H), 2.76–2.73 (m, 6H), 2.44 (s, 3H). ^**13**^ **C NMR** (151 MHz, DMSO-*d*_6_) δ 155.0, 151.5, 138.5, 133.1, 63.0, 45.0, 36.1, 35.3, 13.8. **HRMS** (ESI): *m*/*z* calculated for C_9_H_14_N_4_O_4_ + H^+^: 243.1082 [M + H]^+^, found: 243.1066.

#### 2-(2-Methyl-5-nitro-1*H*-imidazol-1-yl)­ethyl Isopropyl­(methyl)­carbamate (**12f**)

The reaction was carried out on a 0.58 mmol scale, using *N*–methylpropan-2-amine hydrochloride (0.13 g, 1.2 mmol, 2 equiv), following general procedu re E. The title compound was obtained as a colorless viscous oil (3 mg, 17 μmol, 2%) after purification via reversed-phase preparative HPLC, with a Waters Sunfire C18 5 μm ODB 19 × 150 mm column and water/acetonitrile in 0.015% difluoroacetic acid (pH 3) as the eluent phase, following the gradient: 10–70%. ^**1**^ **H NMR** (600 MHz, DMSO-*d*_6_) δ 8.03 (s, 1H), 4.59 (t, *J* = 4.9 Hz, 2H), 4.33 (t, *J* = 5.1 Hz, 2H), 4.20–3.94 (m, 1H), 2.64–2.51 (m, 3H), 2.43 (s, 3H), 0.99 (d, *J* = 6.8 Hz, 6H). ^**13**^ **C NMR** (151 MHz, DMSO-*d*_6_) δ 154.4, 151.5, 138.6, 133.2, 62.9, 62.6, 46.3, 46.0, 45.2, 27.0, 26.7, 19.6, 19.2, 13.8. **HRMS** (ESI): *m*/*z* calculated for C_11_H_18_N_4_O_4_ + H^+^: 271.1401 [M + H]^+^, found: 271.1413.

#### 2-(2-Methyl-5-nitro-1*H*-imidazol-1-yl)­ethylcyclopropyl­(methyl)­carbamate (**12g**)

The reaction was carried out on a 0.58 mmol scale, using *N*-methylcyclopropanamine hydrochloride (0.13 g, 1.2 mmol, 2 equiv), following general procedure E. The title compound was obtained as a colorless solid (as difluoroacetate) (7 mg, 23 μmol, 4%) after purification via reversed-phase preparative HPLC with a Waters Sunfire C18 5 μm ODB 19 × 150 mm column and water/acetonitrile in 0.015% difluoroacetic acid (pH 3) as the eluent phase, following the gradient: 10–70%. ^**1**^ **H NMR** (600 MHz, DMSO-*d*_6_) δ 8.00 (s, 1H), 4.56 (t, *J* = 5.0 Hz, 2H), 4.29 (t, *J* = 5.1 Hz, 2H), 2.67 (s, 3H), 2.41 (s, 4H), 0.58 (d, *J* = 6.9 Hz, 2H), 0.44 (d, *J* = 4.0 Hz, 2H). ^**13**^ **C NMR** (151 MHz, DMSO-*d*_6_) δ 156.1, 151.6, 138.5, 133.1, 63.0, 45.0, 34.4, 29.9, 13.9, 7.2. **HRMS** (ESI): *m*/*z* calculated for C_11_H_16_N_4_O_4_ + H^+^: 269.1245 [M + H]^+^, found: 269.1275.

#### 2-(2-Methyl-5-nitro-1*H*-imidazol-1-yl)­ethylcyclopropyl­(isopropyl)­carbamate (**12h**)

In a heat gun-dried vial, *N*-isopropylcyclopropanamine hydrochloride (60 mg, 0.44 mmol, 1 equiv) and 4-dimethylaminopyridine (4.9 mg, 40 μmol, 0.1 equiv) were suspended in dry THF (6.5 mL). Subsequently, DIPEA (0.31 mL, 1.8 mmol, 4 equiv) was added dropwise, and the mixture was stirred for 30 min at room temperature. Then, the reaction was cooled to 0 °C, and 4-nitrophenyl chloroformate (0.12 g, 0.58 mmol, 1.3 equiv) was added in one portion. After 10 min of stirring at 0 °C, the mixture was warmed up to 50 °C, affording a pale-yellow suspension. The reaction was monitored via LC-MS analysis, showing complete conversion after 6 h. Then, MeOH (1 mL) was added to quench the reaction, providing a yellow solution. The solvent was removed *in vacuo* at 43 °C, and dichloromethane (20 mL) was added. The resulting solution was washed with an aqueous saturated ammonium chloride solution (10 mL), water (10 mL), and brine (10 mL) and dried over a phase separator, and the solvent was removed *in vacuo* at 43 °C to afford 4-nitrophenyl cyclopropyl­(isopropyl)­carbamate as a pale-yellow oil. The resulting crude was used for the following step without further purification. In a heat gun-dried vial, NaH 60% in mineral oil (50 mg, 0.20 mmol, 1.3 equiv) was suspended in dry THF (1.0 mL) and stirred at 0 °C for 30 min. 2-(2-Methyl-5-nitro-1*H*-imidazol-1-yl)­ethan-1-ol **21** (26 mg, 0.15 mmol, 1 equiv) as a solution in THF (1.0 mL) was added dropwise, forming a dark brown suspension. The suspension was stirred for 40 min at 0 °C, gradually becoming more fluid, after which 4-nitrophenyl cyclopropyl­(isopropyl)­carbamate (40 mg, 0.15 mmol, 1 equiv) was added dropwise as a THF solution (1.0 mL). The reaction was stirred at 0 °C for 15 min, then warmed up to 20 °C and stirred for 42 h, and quenched with an aqueous saturated sodium bicarbonate solution (0.5 mL). The mixture was dried *in vacuo*; the crude was redissolved in dichloromethane (20 mL) and washed with water (10 mL) and brine (10 mL). The organic phase was dried over a phase separator, and subsequently, the solvent was removed *in vacuo* at 43 °C, to deliver a brown oily crude. The crude was dissolved in 1.0 mL DMSO and purified via prep HPLC with a Waters XBridge C18 column (5 μm, 30 mm × 150 mm), with (water/acetonitrile/ammonia 95/5/0.2)/acetonitrile as the eluent phase, following a linear gradient of 10–70%, delivering 2-(2-methyl-5-nitro-1*H*-imidazol-1-yl)­ethylcyclopropyl­(isopropyl)­carbamate **12h** as a viscous brown oil (12 mg, 40 μmol, 27%). ^**1**^ **H NMR** (500 MHz, acetone-*d*_6_) δ 7.90 (s, 1H), 4.69 (t, *J* = 5.2 Hz, 2H), 4.41 (t, *J* = 5.2 Hz, 2H), 3.87 (hept, *J* = 6.9 Hz, 1H), 2.49 (s, 3H), 2.36 (tt, *J* = 7.3, 3.9 Hz, 1H), 1.18 (d, *J* = 6.9 Hz, 6H), 0.71–0.50 (m, 4H). ^**13**^ **C NMR** (151 MHz, acetone-*d*_6_) δ 156.0, 151.4, 138.9, 132.7, 62.6, 51.0, 45.4, 27.1, 20.2, 13.6, 7.6. **HRMS** (ESI): *m*/*z* calculated for C_13_H_20_N_4_O_4_ + H^+^: 297.1558 [M + H]^+^, found: 297.1584.

#### 2-(4-(2-(5-Chloro-2-oxobenzo­[*d*]­thiazol-3­(2*H*)-yl)­acetyl)­piperazin-1-yl)­ethylmethyl­(trifluoromethyl)­carbamate (**13a**)

The reaction was performed on a 0.15 mmol scale and stirred for 20 h, using 5-chloro-3-(2-(4-(2-hydroxyethyl)­piperazin-1-yl)-2-oxoethyl)­benzo­[*d*]­thiazol-2­(3*H*)-one (**24**) (53 mg, 0.15 mmol, 1 equiv) and methyl­(trifluoromethyl)­carbamoyl fluoride (**S2**) (98 mg, 0.68 mmol, 4.9 equiv) in acetonitrile (6.8 mL), following general procedure C. The title product was obtained as a colorless solid (9 mg, 0.02 mmol, 13%) after purification via reversed-phase preparative HPLC, with a Waters Sunfire C18 5 μm ODB 19 × 150 mm column and water/acetonitrile in 0.015% difluoroacetic acid (pH 3) as the eluent phase, following the gradient: 3–95%. ^**1**^ **H NMR** (500 MHz, DMSO-*d*_6_) δ 7.70 (d, *J* = 8.4 Hz, 1H), 7.46 (d, *J* = 2.0 Hz, 1H), 7.26 (dd, *J* = 8.4, 2.0 Hz, 1H), 4.94 (s, 2H), 4.31 (t, *J* = 5.6 Hz, 2H), 3.53 (t, *J* = 5.0 Hz, 2H), 3.45–3.40 (m, 2H), 3.08 (q, *J* = 2.3 Hz, 3H), 2.67 (t, *J* = 5.6 Hz, 2H), 2.56 (t, *J* = 5.0 Hz, 2H), 2.44 (t, *J* = 5.1 Hz, 2H). ^**19**^ **F NMR** (471 MHz, DMSO-*d*_6_) δ −55.5 (s, 3F). ^**13**^ **C NMR** (126 MHz, DMSO-*d*_6_) δ 169.4, 163.6, 152.2, 138.8, 131.3, 124.2, 122.9, 120.8 (q, *J =* 259.3 Hz), 119.8, 111.7, 64.2, 55.7, 52.5, 52.3, 44.2, 43.8, 41.7, 31.1. **HRMS** (ESI) *m*/*z* calculated for C_18_H_20_ClF_3_N_4_O_4_S + H^+^: 481.0924 [M + H]^+^, found: 481.0901.

#### 2-(4-(2-(5-Chloro-2-oxobenzo­[*d*]­thiazol-3­(2*H*)-yl)­acetyl)­piperazin-1-yl)­ethylcyclopropyl­(trifluoromethyl)­carbamate (**13b**)

The reaction was performed on a 0.15 mmol scale and stirred at room temperature for 4 h, using 5-chloro-3-(2-(4-(2-hydroxyethyl)­piperazin-1-yl)-2-oxoethyl)­benzo-[*d*]­thiazol-2­(3*H*)-one (**24**) (53 mg, 0.15 mmol, 1 equiv), cyclopropyl­(trifluoromethyl)­carbamoyl fluoride (**S3**) (50 mg, 0.30 mmol, 2 equiv) in acetonitrile (3 mL), following general procedure C. The title product was obtained as a yellow solid (18 mg, 34 μmol, 24%) after purification via reversed-phase preparative HPLC, with a Waters XBridge C18 5 μm ODB 19 × 150 mm column and water/acetonitrile in 0.01 M NH_4_HCO_3_ (pH 9) as the eluent phase, following the gradient: 5–95%. ^**1**^ **H NMR** (600 MHz, DMSO-*d*_6_) δ 7.69 (d, *J* = 8.2 Hz, 1H), 7.45 (s, 1H), 7.24 (d, *J* = 8.0 Hz, 1H), 4.93 (s, 2H), 4.30 (d, *J* = 5.6 Hz, 2H), 3.54 (s, 2H), 3.43 (s, 2H), 2.69 (s, 3H), 2.58 (s, 2H), 2.46 (s, 2H), 0.93 (d, *J* = 6.9 Hz, 2H), 0.78 (s, 2H). ^**19**^ **F NMR** (471 MHz, DMSO-*d*_6_) δ −53.4 (s, 3F). ^**13**^ **C NMR** (151 MHz, DMSO-*d*_6_) δ 169.3, 163.6, 152.5, 138.7, 131.2, 124.2, 122.8, 121.2 (q, *J =* 261.1 Hz), 119.8, 111.6, 63.6, 55.6, 52.4, 52.1, 44.1, 43.7, 41.5, 26.0, 8.2. **HRMS** (ESI) *m*/*z* calcd for C_20_H_22_ClF_3_N_4_O_4_S + H^+^: 507.1081 [M + H]^+^, found: 507.1065.

#### 2-(4-(2-(5-Chloro-2-oxobenzo­[*d*]­thiazol-3­(2*H*)-yl)­acetyl)­piperazin-1-yl)­ethylbenzyl­(trifluoromethyl)­carbamate (**13c**)

The reaction was performed on a 0.17 mmol scale and stirred for 1.5 h using 5-chloro-3-(2-(4-(2-hydroxyethyl)­piperazin-1-yl)-2-oxoethyl)­benzo­[*d*]­thiazol-2­(3*H*)-one (**24**) (60 mg, 0.17 mmol, 1 equiv), benzyl­(trifluoromethyl)­carbamoyl fluoride (**S4**) (80 mg, 0.34 mmol, 2 equiv), DIPEA (60 μL, 0.34 mmol, 2 equiv), and 4-dimethylaminopyridine (4 mg, 0.03 mmol, 0.2 equiv) in acetonitrile (3 mL), following general procedu re C. The title product was obtained as a yellow solid (30 mg, 54 μmol, 32%) after purification via reversed-phase preparative HPLC, with a Waters Sunfire C18 5 μm ODB 19 × 150 mm column and water/acetonitrile in 0.015% difluoroacetic acid (pH 3) as the eluent phase, following the gradient: 3–95%. ^**1**^ **H NMR** (500 MHz, CD_3_CN) δ 7.50 (dd, *J* = 8.5, 1.9 Hz, 1H), 7.41–7.30 (m, 5H), 7.19 (dd, *J* = 8.3, 2.0 Hz, 1H), 7.13 (d, *J* = 2.0 Hz, 1H), 4.76 (q, *J* = 2.2 Hz, 2H), 4.74 (s, 2H), 4.37–4.33 (m, 2H), 3.46 (q, *J* = 5.1 Hz, 4H), 2.70–2.64 (m, 2H), 2.54 (t, *J* = 5.0 Hz, 2H), 2.43 (t, *J* = 5.1 Hz, 2H). ^**19**^ **F NMR** (471 MHz, CD_3_CN) δ −55.2 (s, 3F). ^**13**^ **C NMR** (126 MHz, DMSO-*d*_6_) δ 169.8, 163.9, 152.5, 138.9, 137.0, 131.6, 128.9, 127.9, 127.1, 124.5, 123.2, 122.1 (q, *J =* 261.1 Hz), 120.2, 111.9, 64.3, 56.0, 52.7, 52.4, 48.0, 44.4, 44.0, 41.9. **HRMS** (ESI): *m*/*z* calculated for C_24_H_24_ClF_3_N_4_O_4_S + H^+^: 557.1237 [M + H]^+^, found: 557.1220.

#### 2-(4-(2-(5-Chloro-2-oxobenzo­[*d*]­thiazol-3­(2*H*)-yl)­acetyl)­piperazin-1-yl)­ethyl Phenyl­(trifluoromethyl)­carbamate (**13d**)

The reaction was performed on a 0.17 mmol scale and stirred for 1.5 h, using 5-chloro-3-(2-(4-(2-hydroxyethyl)­piperazin-1-yl)-2-oxoethyl)-benzo­[*d*]­thiazol-2­(3*H*)-one (**24**) (60 mg, 0.17 mmol, 1 equiv), phenyl­(trifluoromethyl)­carbamoyl fluoride (**S5**) (80 mg, 0.34 mmol, 2 equiv), DIPEA (60 μL, 0.34 mmol, 2 equiv), and 4-dimethylaminopyridine (4 mg, 0.03 mmol, 0.2 equiv) in acetonitrile (3 mL), following general procedu re C. The title product was obtained as a yellow solid (21 mg, 40 μmol, 40%) after purification via reversed-phase preparative HPLC, with a Waters Sunfire C18 5 μm ODB 19 × 150 mm column and water/acetonitrile in 0.015% difluoroacetic acid (pH 3) as the eluent phase, following the gradient: 3–95%. ^**1**^ **H NMR** (500 MHz, CD_3_CN) δ 7.54–7.47 (m, 4H), 7.41–7.36 (m, 2H), 7.20 (dd, *J* = 8.4, 2.0 Hz, 1H), 7.15 (d, *J* = 2.0 Hz, 1H), 4.74 (s, 2H), 4.34 (t, *J* = 5.0 Hz, 2H), 3.52 (dt, *J* = 11.8, 5.1 Hz, 4H), 2.78 (t, *J* = 5.1 Hz, 2H), 2.48 (t, *J* = 5.1 Hz, 2H). ^**19**^ **F NMR** (470 MHz, DMSO-*d*_6_) δ –53.7 (s, 3F). ^**13**^ **C NMR** (126 MHz, DMSO-*d*_6_) δ 169.4, 163.7, 151.4, 138.8, 134.6, 131.3, 129.7, 129.6, 129.1, 124.3, 123.0, 120.0, 119.9 (q, *J =* 259.9 Hz), 111.8, 64.3, 55.2, 52.2, 52.0, 43.8, 43.8, 41.2. **HRMS** (ESI): *m*/*z* calculated for C_23_H_22_ClF_3_N_4_O_4_S + H^+^: 543.1080 [M + H]^+^, found: 543.1047.

#### 4-Acetamidophenyl Methyl­(trifluoromethyl)­carbamate (**14a**)

The reaction was performed on a 0.45 mmol scale and stirred for 1 h, using *N*-(4-hydroxyphenyl)­acetamide (**26**) (68 mg, 0.45 mmol, 1 equiv), methyl­(trifluoromethyl)­carbamoyl fluoride (**S2**) (70 mg, 0.45 mmol, 1 equiv), DIPEA (0.16 mL, 0.92 mmol, 2 equiv), and 4-dimethylaminopyridine (11 mg, 90 μmol, 0.2 equiv), in acetonitrile (4.5 mL), following general procedure C. The title product was obtained as a colorless solid (8.9 mg, 32 μmol, 8%) after purification via reversed-phase preparative HPLC, with a Waters XBridge C18 5 μm ODB 19 × 150 mm column and water/acetonitrile in 0.01 M NH_4_HCO_3_ (pH 9) as the eluent phase, following the gradient: 2–94%. ^**1**^ **H NMR** (500 MHz, DMSO-*d*_6_) δ 10.04 (s, 1H), 7.66–7.59 (m, 2H), 7.18–7.12 (m, 2H), 3.20 (q, *J* = 2.3 Hz, 3H), 2.05 (s, 3H). ^**19**^ **F NMR** (471 MHz, DMSO-*d*_6_) δ −53.8 (s, 3F). ^**13**^ **C NMR** (126 MHz, DMSO-*d*_6_) δ 168.3, 151.0, 144.9, 137.5, 121.8, 120.6 (q, *J =* 260.7 Hz), 119.8, 31.6, 23.9. **HRMS** (ESI): *m*/*z* calculated for C_11_H_11_F_3_N_2_O_3_ + H^+^: 277.0800 [M + H]^+^, found: 277.0778.

#### 4-Acetamidophenyl Cyclopropyl­(trifluoromethyl)­carbamate (**14b**)

The reaction was performed on a 0.30 mmol scale and stirred at room temperature for 1 h, using *N*-(4-hydroxyphenyl)­acetamide (**26**) (50 mg, 0.30 mmol, 1 equiv), cyclopropyl­(trifluoromethyl)­carbamoyl fluoride (**S3**) (50 mg, 0.30 mmol, 1 equiv), DIPEA (0.10 mL, 0.60 mmol, 2 equiv), and 4-dimethylaminopyridine (7 mg, 0.06 mmol, 0.2 equiv) in acetonitrile (3.0 mL), following general procedure C. The title product was obtained as a colorless solid (31 mg, 0.10 mmol, 34%) after purification via reversed-phase preparative HPLC, with a Waters XBridge C18 5 μm ODB 19 × 150 mm column and water/acetonitrile in 0.01 M NH_4_HCO_3_ (pH 9) as the eluent phase, following the gradient: 2–94%. ^**1**^ **H NMR** (500 MHz, DMSO-*d*_6_) δ 10.03 (s, 1H), 7.68–7.56 (m, 2H), 7.16 (ddt, *J* = 8.9, 3.6, 1.5 Hz, 2H), 2.93–2.79 (m, 1H), 2.09–1.97 (m, 3H), 1.08–0.82 (m, 4H). ^**19**^ **F NMR** (471 MHz, DMSO-*d*_6_) δ −53.5 (s, 3F). ^**13**^ **C NMR** (126 MHz, DMSO-*d*_6_) δ 168.5, 151.5, 145.1, 137.5, 121.8, 121.1 (q, *J =* 262.0 Hz), 119.9, 26.6, 24.0, 8.3. **HRMS** (ESI): *m*/*z* calculated for C_13_H_13_F_3_N_2_O_3_ + H^+^: 303.0956 [M + H]^+^, found: 303.0969.

#### 4-Acetamidophenyl Benzyl­(trifluoromethyl)­carbamate (**14c**)

The reaction was performed on a 0.34 mmol scale and stirred at room temperature for 1.5 h using *N*-(4-hydroxyphenyl)­acetamide (**26**) (51 mg, 0.34 mmol, 1 equiv), benzyl­(trifluoromethyl)­carbamoyl fluoride (**S4**) (75 mg, 0.34 mmol, 1 equiv), DIPEA (0.12 mL, 0.69 mmol, 2 equiv), and 4-dimethylaminopyridine (8 mg, 0.07 mmol, 0.2 equiv) in acetonitrile (3.5 mL), following general procedure C. The title product was obtained as a colorless solid (37 mg, 0.11 mmol, 31%) after purification via reversed-phase preparative HPLC, with a Waters Sunfire C18 5 μm ODB 19 × 150 mm column and water/acetonitrile in 0.015% difluoroacetic acid (pH 3) as the eluent phase, following the gradient: 3–95%. ^**1**^ **H NMR** (500 MHz, DMSO-*d*_6_) δ 10.06 (s, 1H), 7.67–7.61 (m, 2H), 7.47–7.39 (m, 2H), 7.38–7.29 (m, 3H), 7.16 (m, 2H), 4.90 (s, 2H), 2.05 (s, 3H). ^**19**^ **F NMR** (471 MHz, DMSO-*d*_6_) δ −53.8 (s, 3F). ^**13**^ **C NMR** (126 MHz, DMSO-*d*_6_) δ 168.4, 151.1, 144.9, 137.6, 136.4, 128.8, 127.8, 126.8, 120.8 (q, *J =* 261.6 Hz), 121.7, 119.9, 48.3, 24.0. **HRMS** (ESI): *m*/*z* calculated for C_17_H_15_F_3_N_2_O_3_ + H^+^: 353.1113 [M + H]^+^, found: 353.1119.

#### 4-Acetamidophenyl Phenyl­(trifluoromethyl)­carbamate (**14d**)

The reaction was performed on a 0.34 mmol scale and stirred at room temperature for 1 h, using *N*-(4-hydroxyphenyl)­acetamide (**26**) (51 mg, 0.34 mmol, 1 equiv), phenyl­(trifluoromethyl)­carbamoyl fluoride (**S5**) (70 mg, 0.34 mmol, 1 equiv), DIPEA (0.12 mL, 0.69 mmol, 2 equiv), and 4-dimethylaminopyridine (8 mg, 0.07 mmol, 0.2 equiv) in acetonitrile (3.5 mL), following general procedure C. The title product was obtained as a brown solid (4.5 mg, 14 μmol, 4%) after purification via reversed-phase preparative HPLC, with a Waters Sunfire C18 5 μm ODB 19 × 150 mm column and water/acetonitrile in 0.015% difluoroacetic acid (pH 3) as the eluent phase, following the gradient: 3–95%. ^**1**^ **H NMR** (600 MHz, DMSO-*d*_6_) δ 10.09 (s, 1H), 7.54 (m, 7H), 7.09 (d, *J* = 8.0 Hz, 2H), 2.01 (s, 3H). ^**19**^ **F NMR** (471 MHz, DMSO-*d*_6_) δ −53.9 (s, 3F). ^**13**^ **C NMR** (151 MHz, DMSO-*d*_6_) δ 168.9, 150.8, 145.2, 138.1, 134.9, 130.3, 130.2, 129.5, 120.3 (q, *J* = 260.4 Hz), 122.2, 120.3, 24.4. **HRMS** (ESI): *m*/*z* calculated for C_16_H_13_F_3_N_2_O_3_ + H^+^: 339.0956 [M + H]^+^, found: 339.0927.

#### 1-Cyclopropyl-6-fluoro-7-(4- (methyl­(trifluoromethyl)­carbamoyl)­piperazin-1-yl)-4-oxo-1,4-dihydroquinoline-3-carboxylic Acid (**15a**)

The reaction was performed on a 0.18 mmol scale and stirred at room temperature for 20 h using 1-cyclopropyl-6-fluoro-4-oxo-7-(piperazin-1-yl)-1,4-dihydroquinoline-3-carboxylic acid hydrochloride (80 mg, 0.21 mmol, 1.2 equiv) (**22**) and methyl­(trifluoromethyl)­carbamoyl fluoride (**S2**) (30 mg, 0.18 mmol, 1 equiv), DIPEA (60 μL, 0.36 mmol, 2 equiv), and 4-dimethylaminopyridine (2 mg, 0.02 mmol, 0.1 equiv), in acetonitrile (2.0 mL), following general procedure C. The title product was obtained as a colorless solid (5.9 mg, 13 μmol, 8%) after purification via reversed-phase preparative HPLC, with a Waters XBridge C18 5 μm ODB 19 × 150 mm column and water/acetonitrile in 0.01 M NH_4_HCO_3_ (pH 9), as the eluent phase, following the gradient: 2–94%. ^**1**^ **H NMR** (500 MHz, CD_3_CN) δ 8.72 (s, 1H), 7.97 (d, *J* = 13.4 Hz, 1H), 7.56 (d, *J* = 7.5 Hz, 1H), 3.67–3.65 (m, 5H), 3.37–3.35 (m, 4H), 2.94 (q, *J* = 1.0 Hz, 3H), 1.37–1.32 (m, 2H), 1.15–1.13 (m, 2H). ^**19**^ **F NMR** (470 MHz, CD_3_CN) δ −58.3 (s, 3F), −123.3 (s, 1F). ^**13**^ **C NMR** (126 MHz, CD_3_CN) δ 177.3, 166.6, 156.7, 154.6 (d, *J =* 248.8 Hz), 148.3, 146.4 (d, *J =* 10.6 Hz), 139.5, 122.5 (q, *J =* 258.6 Hz), 119.9 (d, *J =* 7.2 Hz), 111.4 (d, *J =* 23.5 Hz), 107.5, 106.8, 49.4, 44.8, 35.7, 33.2, 7.6. **HRMS** (ESI): *m*/*z* calculated for C_20_H_20_F_4_N_4_O_4_ + H^+^: 457.1493 [M + H]^+^, found: 457.1541.

#### 1-Cyclopropyl-7-(4-(cyclopropyl­(trifluoromethyl)­carbamoyl)­piperazin-1-yl)-6-fluoro-4-oxo-1,4-dihydroquinoline-3-carboxylic Acid (**15b**)

The reaction was performed on a 0.13 mmol scale and stirred at room temperature for 22 h using 1-cyclopropyl-6-fluoro-4-oxo-7-(piperazin-1-yl)-1,4-dihydroquinoline 3-carboxylic acid hydrochloride (60 mg, 0.16 mmol, 1.2 equiv) (**22**) and cyclopropyl­(trifluoromethyl)­carbamoyl fluoride (**S3**) (20 mg, 0.13 mmol, 1 equiv), DIPEA (50 μL, 0.26 mmol, 2 equiv), and 4-dimethylaminopyridine (2 mg, 0.02 mmol, 0.15 equiv), in acetonitrile (1.0 mL), following general procedure C. The title product was obtained as a colorless solid (2 mg, 4 μmol, 3%) after purification via reversed-phase preparative HPLC, with a Waters XBridge C18 5 μm ODB 19 × 150 mm column and water/acetonitrile in 0.01 M NH_4_HCO_3_ (pH 9) as the eluent phase, following the gradient: 2–94%. ^**1**^ **H NMR** (500 MHz, CD_3_CN) δ 8.75 (s, 1H), 8.01 (d, *J* = 13.2 Hz, 1H), 7.60 (d, *J* = 7.4 Hz, 1H), 3.81–3.60 (m, 5H), 3.49–3.26 (m, 4H), 2.86–2.79 (m, 1H), 1.42–1.35 (m, 2H), 1.20–1.14 (m, 2H), 0.91–0.85 (m, 2H), 0.81–0.76 (m, 2H). ^**19**^ **F NMR** (470 MHz, CD_3_CN) δ −58.3 (s, 3F), −123.3 (s, 1F). ^**13**^ **C NMR** (126 MHz, CD_3_CN) δ 178.3, 167.5, 155.9, 154.7 (d, *J* = 249.2 Hz), 149.3, 146.3 (d, *J* = 10.5 Hz), 123.5 (q, *J* = 258.7 Hz), 120.9 (d, *J* = 7.8 Hz), 112.4 (d, *J =* 23.0 Hz), 108.5, 107.8, 50.5, 50.5, 45.6, 41.4, 36.7, 28.3, 8.6, 7.3. **HRMS** (ESI): *m*/*z* calculated for C_22_H_22_F_4_N_4_O_4_ + H^+^: 483.1611 [M + H]^+^, found: 483.1661.

#### 7-(4-(Benzyl­(trifluoromethyl)­carbamoyl)­piperazin-1-yl)-1-cyclopropyl-6-fluoro-4-oxo-1,4-dihydroquinoline-3-carboxylic Acid (**15c**)

The reaction was performed on a 0.23 mmol scale and stirred at room temperature for 18 h using 1-cyclopropyl-6-fluoro-4-oxo-7­(piperazin-1-yl)-1,4-dihydroquinoline-3-carboxylic acid hydrochloride (89 mg, 0.27 mmol, 1.2 equiv) (**22**), benzyl­(trifluoromethyl)­carbamoyl fluoride (**S4**) (50 mg, 0.23 mmol, 1 equiv), DIPEA (80 μL, 0.46 mmol, 2 equiv), and 4-dimethylaminopyridine (2 mg, 0.02 mmol, 0.1 equiv), in acetonitrile (2.0 mL), following general procedure C. The title product was obtained as a colorless solid (9.1 mg, 17 μmol, 8%) after purification via reversed-phase preparative HPLC, with a Waters XBridge C18 5 μm ODB 19 × 150 mm column and water/acetonitrile in 0.01 M NH_4_HCO_3_ (pH 9) as the eluent phase, following the gradient: 2–94%. ^**1**^ **H NMR** (500 MHz, DMSO-*d*_6_) δ 8.66 (s, 1H), 7.91 (d, *J* = 13.0 Hz, 1H), 7.49 (d, *J* = 7.3 Hz, 1H), 7.42–7.29 (m, 5H), 4.46 (s, 2H), 3.80 (tt, *J* = 7.3, 4.1 Hz, 1H), 3.63 (t, *J* = 5.0 Hz, 4H), 3.13 (t, *J* = 5.0 Hz, 4H), 1.35–1.14 (m, 4H). ^**19**^ **F NMR** (471 MHz, DMSO-*d*_6_) δ −57.3 (s, 3F), −121.9 (s, 1F). ^**13**^ **C NMR** (151 MHz, DMSO-*d*_6_) δ 176.4, 166.0, 154.2, 153.0 (d, *J =* 249.3 Hz), 148.2, 144.6 (d, *J =* 10.5 Hz), 139.1, 135.3, 128.8, 128.6, 128.1, 121.8 (q, *J =* 259.3 Hz), 119.2 (d, *J =* 7.0 Hz), 111.1 (d, *J =* 23.5 Hz), 107.1, 106.8, 49.3, 49.1, 44.6, 35.9, 7.6. **HRMS** (ESI): *m*/*z* calculated for C_26_H_24_F_4_N_4_O_4_ + H^+^: 533.1734 [M + H]^+^, found: 533.1793.

#### 1-Cyclopropyl-6-fluoro-4-oxo-7-(4-(phenyl­(trifluoromethyl)­carbamoyl)­piperazin-1-yl)-1,4-dihydroquinoline-3-carboxylic Acid (**15d**)

The reaction was performed on a 0.21 mmol scale and stirred at room temperature for 24 h, using 1-cyclopropyl-6-fluoro-4-oxo-7­(piperazin-1-yl)-1,4-dihydroquinoline-3-carboxylic acid hydrochloride (83 mg, 0.25 mmol, 1.2 equiv) (**22**), phenyl­(trifluoromethyl)­carbamoyl fluoride (**S5**) (40 mg, 0.21 mmol, 1 equiv), DIPEA (70 μL, 0.42 mmol, 2 equiv), and 4-dimethylaminopyridine (2 mg, 0.020 mmol, 0.1 equiv), in dichloromethane (1.5 mL), following general procedure C. The title product was obtained as a colorless solid (20 mg, 39 μmol, 19%) after purification via reversed-phase preparative HPLC, with a Waters Sunfire C18 5 μm ODB 19 × 150 mm column and water/acetonitrile in 0.015% difluoroacetic acid (pH 3) as the eluent phase, following the gradient: 23–78%. ^**1**^ **H NMR** (600 MHz, CD_3_CN) δ 8.69 (s, 1H), 7.91 (d, *J* = 13.1 Hz, 1H), 7.55–7.37 (m, 6H), 3.61 (tt, *J* = 7.4, 4.1 Hz, 1H), 3.56 (t, *J* = 5.1 Hz, 4H), 3.06 (t, *J* = 5.0 Hz, 4H), 1.31 (t, *J* = 6.7 Hz, 2H), 1.11 (dd, *J* = 6.4, 4.2 Hz, 2H). ^**19**^ **F NMR** (471 MHz, DMSO-*d*_6_) δ −56.9 (s, 3F), −121.9 (s, 1F). ^**13**^ **C NMR** (151 MHz, CD_3_CN) δ 178.2, 167.5, 155.6, 154.5 (d, *J =* 248.8 Hz), 149.2, 146.1 (d, *J =* 10.6 Hz), 140.4, 137.9, 130.9, 129.8, 129.1, 122.2 (q, *J =* 259.0 Hz), 120.8 (d, *J =* 7.7 Hz), 112.4 (d, *J =* 23.5 Hz), 108.5, 107.5 (d, *J =* 3.5 Hz), 49.8, 45.6, 36.7, 8.6. **HRMS** (ESI): *m*/*z* calculated for C_25_H_22_F_4_N_4_O_4_ + H^+^: 519.1577 [M + H]^+^, found: 519.1661.

#### 1-Cyclopropyl-7-(4-(dimethylcarbamoyl)­piperazin-1-yl)-6-fluoro-4-oxo-1,4-dihydroquinoline-3-carboxylic Acid (**15e**)

The reaction was carried out on a 0.12 mmol scale, using dimethylamine hydrochloride (15 mg, 0.18 mmol, 1.5 equiv), following general procedur e F. The title compound was obtained as a colorless solid (10 mg, 26 μmol, 21%) after purification via reversed-phase preparative HPLC with a Waters Sunfire C18 5 μm ODB 19 × 150 mm column and water/acetonitrile in 0.015% difluoroacetic acid (pH 3) as the eluent phase, following the gradient: 10–94%. ^**1**^ **H NMR** (500 MHz, CD_3_CN) δ 8.72 (s, 1H), 7.97 (d, *J* = 13.3 Hz, 1H), 7.56 (d, *J* = 7.4 Hz, 1H), 3.66 (tt, *J* = 7.3, 4.0 Hz, 1H), 3.40–3.32 (m, 8H), 2.83 (s, 6H), 1.42–1.10 (m, 4H). ^**19**^ **F NMR** (471 MHz, CD_3_CN) δ −123.0 (s, 1F). ^**13**^ **C NMR** (126 MHz, CD_3_CN) δ 178.2, 167.5, 165.2, 154.7 (d, *J =* 249.6 Hz), 149.2, 146.8 (d, *J =* 10.4 Hz), 140.6, 120.6 (d, *J =* 7.8 Hz), 112.3 (d, *J =* 23.5 Hz), 108.4, 107.5, 50.6, 47.4, 38.6, 36.7, 8.6. **HRMS** (ESI): *m*/*z* calculated for C_20_H_23_FN_4_O_4_ + H^+^: 403.1703 [M + H]^+^, found: 403.1781.

#### 1-Cyclopropyl-6-fluoro-7-(4-(isopropyl­(methyl)­carbamoyl)­piperazin-1-yl)-4-oxo-1,4-dihydroquinoline-3-carboxylic Acid (**15f**)

The reaction was carried out on a 0.12 mmol scale, using *N*-methylpropan-2-amine hydrochloride (20 mg, 0.18 mmol, 1.5 equiv), following general procedure F. The title compound was obtained as a colorless solid (4 mg, 8 μmol, 7%) after purification via reversed-phase preparative HPLC with a Waters Sunfire C18 5 μm ODB 19 × 150 mm column and water/acetonitrile in 0.015% difluoroacetic acid (pH 3) as the eluent phase, following the gradient: 10–94%. ^**1**^ **H NMR** (500 MHz, CD_3_CN) δ 8.71 (s, 1H), 7.96 (d, *J* = 13.3 Hz, 1H), 7.56 (d, *J* = 7.4 Hz, 1H), 4.05 (hept, *J* = 6.7 Hz, 1H), 3.66 (tt, *J* = 7.3, 4.0 Hz, 1H), 3.35 (s, 8H), 2.72 (s, 3H), 1.39–1.32 (m, 2H), 1.16–1.11 (m, 8H). ^**19**^ **F NMR** (471 MHz, CD_3_CN) δ −123.0 (s, 1F). ^**13**^ **C NMR** (126 MHz, CD_3_CN) δ 178.2, 167.5, 165.1, 154.7 (d, *J =* 250.0 Hz), 149.2, 146.8 (d, *J =* 10.4 Hz), 140.6, 120.6 (d, *J =* 7.4 Hz), 112.3 (d, *J =* 23.8 Hz), 108.4, 107.5, 50.6, 49.0, 47.6, 36.7, 29.9, 19.7, 8.6. **HRMS** (ESI): *m*/*z* calculated for C_22_H_27_FN_4_O_4_ + H^+^: 431.2016 [M + H] ^+^, found: 431.2093.

#### 1-Cyclopropyl-7-(4-(cyclopropyl­(methyl)­carbamoyl)­piperazin-1-yl)-6-fluoro-4-oxo-1,4-dihydroquinoline-3-carboxylic Acid (**15g**)

The reaction was carried out on a 0.18 mmol scale, using 1-cyclopropyl-6-fluoro-4-oxo-7-(piperazin-1-yl)-1,4-dihydroquinoline-3-carboxylic acid hydrochloride **22** (59 mg, 0.18 mmol, 1.0 equiv), bis­(trichloromethyl) carbonate (BTC) (18 mg, 60 μmol, 0.3 equiv), *N*-methylcyclopropanamine (23 μL, 0.27 mmol, 1.5 equiv), and DIPEA (0.10 mL, 0.54 mmol, 3.0 equiv) in acetonitrile (3 mL), following general procedure F. The title compound was obtained as a colorless solid (16 mg, 37 μmol, 20%) after purification via reversed-phase preparative HPLC with a Waters Sunfire C18 5 μm ODB 19 × 150 mm column and water/acetonitrile in 0.015% difluoroacetic acid (pH 3) as the eluent phase, following the gradient: 10–94%. ^**1**^ **H NMR** (500 MHz, CD_3_CN) δ 8.70 (s, 1H), 7.94 (d, *J* = 13.3 Hz, 1H), 7.55 (d, *J* = 7.4 Hz, 1H), 3.66 (s, 1H), 3.48 (dd, *J* = 6.4, 3.5 Hz, 4H), 3.37–3.29 (m, 4H), 2.82 (s, 3H), 2.62 (tt, *J* = 6.8, 3.8 Hz, 1H), 1.40–1.30 (m, 2H), 1.14 (d, *J* = 3.2 Hz, 2H), 0.73–0.56 (m, 4H). ^**19**^ **F NMR** (471 MHz, CD_3_CN) δ −123.0 (s, 1F). ^**13**^ **C NMR** (126 MHz, CD_3_CN) δ 178.2, 167.5, 164.2, 154.7 (d, *J =* 249.8 Hz), 149.1, 146.8 (d, *J =* 10.5 Hz), 140.5, 120.5 (d, *J =* 7.7 Hz), 112.3 (d, *J =* 23.5 Hz), 108.4, 107.4, 50.7, 46.6, 37.8, 36.7, 32.9, 8.6, 8.4. **HRMS** (ESI): *m*/*z* calculated for C_22_H_25_FN_4_O_4_ + H^+^: 429.1933 [M + H]^+^, found: 429.1939.

#### 1-Cyclopropyl-7-(4-(cyclopropyl­(isopropyl)­carbamoyl)­piperazin-1-yl)-6-fluoro-4-oxo-1,4-dihydroquinoline-3-carboxylic Acid (**15h**)

In a heat gun-dried vial, bis­(trichloromethyl) carbonate (BTC) (50 mg, 0.17 mmol, 0.4 equiv) was dissolved in dry dichloromethane (0.3 mL). In a second heat gun-dried vial, *N*-isopropylcyclopropanamine (50 mg, 0.50 mmol, 1 equiv) was dissolved in dichloromethane (0.7 mL), and triethylamine (70 μL, 0.50 mmol, 1 equiv) was added dropwise. The bis­(trichloromethyl) carbonate solution in dichloromethane was cooled to 0 °C, and the mixture of *N*-isopropylcyclopropanamine and triethylamine was transferred dropwise. The reaction was stirred at 0 °C for 4 h and warmed up to room temperature. The solvent was removed via the Schlenk line, delivering cyclopropyl­(isopropyl)­carbamoyl chloride as a crude. The obtained crude (80 mg, 0.50 mmol, 1 equiv) and 1-cyclopropyl-6-fluoro-4-oxo-7-(piperazin-1-yl)-1,4-dihydroquinoline-3-carboxylic acid **22** (0.17 g, 0.50 mmol, 1 equiv) were dissolved in dichloromethane (1 mL) in a heat gun-dried vial. The mixture was stirred for 5 min, and then, triethylamine (0.21 mL, 1.5 mmol, 3 equiv) was added dropwise. The reaction was stirred for 12 h at room temperature, resulting in a white suspension. The reaction was diluted with water (3 mL) and extracted with dichloromethane (3 × 3 mL). The combined organic layers were washed with brine (2 mL) and dried over a phase separator, and the solvent was removed *in vacuo* at 43 °C. The resulting oily crude was solubilized in DMSO (0.8 mL), filtered via a 0.45 μm filter, and purified via reversed-phase preparative HPLC with a Waters Sunfire C18 5 μm ODB 19 × 150 mm column and water/acetonitrile in 0.015% difluoroacetic acid (pH 3) as the eluent phase, following the gradient 3–95%, delivering 1-cyclopropyl-7-(4-(cyclopropyl­(isopropyl)­carbamoyl)­piperazin-1-yl)-6-fluoro-4-oxo-1,4-dihydroquinoline-3-carboxylic acid (as difluoroacetate) (**15h**) (9.1 mg, 20 μmol, 4%). ^**1**^ **H NMR** (500 MHz, CD_3_CN) δ 8.70 (s, 1H), 7.94 (d, *J* = 13.6 Hz, 1H), 7.54 (d, *J* = 7.3 Hz, 1H), 3.86–3.73 (m, 1H), 3.67 (s, 1H), 3.49 (d, *J* = 4.9 Hz, 4H), 3.31 (t, *J* = 4.9 Hz, 4H), 2.91–2.74 (m, 1H), 2.46 (s, 1H), 1.38–1.11 (m, 13H), 0.77–0.49 (m, 4H). ^**13**^ **C NMR** (126 MHz, DMSO-*d*_6_) δ 176.4, 165.9, 162.9, 153.0 (*J* = 249.7 Hz), 148.0, 145.1 (*J* = 9.7 Hz), 139.2, 118.7, 111.0 (*J* = 23.8 Hz), 106.6 (*J* = 26.0 Hz), 51.1, 49.4, 45.2, 35.9, 20.8, 7.7, 7.6. **HRMS** (ESI): *m*/*z* calculated for C_24_H_29_FN_4_O_4_ + H^+^: 457.2246 [M + H]^+^, found: 457.2248.

#### 4-((4-Ethoxy-3-(1-methyl-7-oxo-3-propyl-6,7-dihydro-1*H*-pyrazolo­[4,3-*d*]­pyrimidin-5-yl)­phenyl)­sulfonyl)-*N*-methyl-*N*-(trifluoromethyl)­piperazine-1-carboxamide (**16a**)

The reaction was performed on a 0.34 mmol scale and stirred for 24 h at room temperature, using 5-(2-ethoxy-5-(piperazin-1-ylsulfonyl)-phenyl)-1-methyl-3-propyl-1,6-dihydro-7*H*-pyrazolo­[4,3-*d*]­pyrimidin-7-one (0.19 g, 0.41 mmol, 1.2 equiv) (**30**), methyl­(trifluoromethyl)­carbamoyl fluoride (**S2**(S)) (50 mg, 0.34 mmol, 1 equiv), DIPEA (0.12 mL, 0.68 mmol, 2 equiv), and 4-dimethylaminopyridine (4.2 mg, 34 μmol, 0.1 equiv) in acetonitrile (2.0 mL), following general procedure C. The title product was obtained as a colorless solid (2 mg, 3 μmol, 1%) after purification via reversed-phase preparative HPLC, with a Waters XBridge C18 5 μm ODB 19 × 150 mm column and water/acetonitrile in 0.01 M NH_4_HCO_3_ (pH 9) as the eluent phase, following the gradient: 5–95%. ^**1**^ **H NMR** (500 MHz, CD_3_CN) δ 8.44 (d, *J* = 2.6 Hz, 1H), 7.85 (d, *J* = 9.0 Hz, 1H), 7.31 (d, *J* = 8.9 Hz, 1H), 4.33 (q, *J* = 7.0 Hz, 2H), 4.17 (s, 3H), 3.51 (t, *J* = 5.3 Hz, 4H), 3.01 (d, *J* = 5.3 Hz, 4H), 2.87 (t, *J* = 7.6 Hz, 2H), 2.81 (s, 3H), 1.81 (q, *J* = 7.4 Hz, 2H), 1.50 (t, *J* = 7.0 Hz, 3H), 0.99 (t, *J* = 7.4 Hz, 3H). ^**19**^ **F NMR** (470 MHz, CD_3_CN) δ −62.2 (s, 3F). ^**13**^ **C NMR** (151 MHz, CD_3_CN) δ 161.1, 157.6, 154.3, 148.3, 147.0, 139.1, 132.8, 131.4, 128.9, 125.6, 123.4 (q, *J* = 257.7 Hz), 122.8, 114.7, 67.0, 46.7, 45.5, 38.6, 34.1, 28.3, 23.0, 14.7, 14.2. **HRMS** (ESI): *m*/*z* calculated for C_24_H_30_F_3_N_7_O_5_S + H^+^: 586.1981 [M + H]^+^, found: 586.2062.

#### *N*-Cyclopropyl-4-((4-ethoxy-3-(1-methyl-7-oxo-3-propyl-6,7-dihydro-1*H*-pyrazolo­[4,3-*d*]­pyrimidin-5-yl)­phenyl)­sulfonyl)-*N*-(trifluoromethyl)­piperazine-1-carboxamide (**16b**)

The reaction was performed on a 0.13 mmol scale and stirred at room temperature for 22 h using 5-(2-ethoxy-5-(piperazin-1-ylsulfonyl)­phenyl)-1-methyl-3-propyl-1,6-dihydro-7*H*-pyrazolo­[4,3-*d*]­pyrimidin-7-one (80 mg, 0.16 mmol, 1.2 equiv) (**30**), cyclopropyl­(trifluoromethyl)­carbamoyl fluoride (**S3**) (22 mg, 0.13 mmol, 1 equiv), DIPEA (45 μL, 0.26 mmol, 2 equiv), and 4-dimethylaminopyridine (3.2 mg, 26 μmol, 0.2 equiv) in acetonitrile (1.0 mL), following general procedure C. The title product was obtained as a colorless solid (7.3 mg, 12 μmol, 9%) after purification via reversed-phase preparative HPLC, with a Waters XBridge C18 5 μm ODB 19 × 150 mm column and water/acetonitrile in 0.01 M NH_4_HCO_3_ (pH 9) as the eluent phase, following the gradient: 2–94%. ^**1**^ **H NMR** (500 MHz, CD_3_CN) δ 8.42 (d, *J* = 2.4 Hz, 1H), 7.85 (dd, *J* = 8.8, 2.4 Hz, 1H), 7.31 (d, *J* = 8.9 Hz, 1H), 4.32 (q, *J* = 6.9 Hz, 2H), 4.17 (s, 3H), 3.57 (t, *J* = 5.1 Hz, 4H), 3.01 (t, *J* = 5.1 Hz, 4H), 2.86 (t, *J* = 7.5 Hz, 2H), 2.68 (tdd, *J* = 6.6, 5.4, 3.3 Hz, 1H), 1.81 (h, *J* = 7.4 Hz, 2H), 1.50 (t, *J* = 6.9 Hz, 3H), 0.98 (t, *J* = 7.4 Hz, 3H), 0.77–0.58 (m, 4H). ^**19**^ **F NMR** (471 MHz, CD_3_CN) δ −58.3 (s, 3F). ^**13**^ **C NMR** (151 MHz, CD_3_CN) δ 161.1, 155.7, 154.4, 148.4, 146.9, 139.1, 132.7, 131.4, 128.9, 125.6, 123.3 (q, *J* = 259.4 Hz), 122.9, 114.7, 66.9, 46.8, 45.2, 38.6, 28.3, 28.2, 23.0, 14.7, 14.2, 7.3. **HRMS** (ESI): *m*/*z* calculated for C_26_H_32_F_3_N_7_O_5_S + H^+^: 612.2138 [M + H]^+^, found: 612.2227.

#### *N*-Benzyl-4-((4-ethoxy-3-(1-methyl-7-oxo-3-propyl-6,7-dihydro-1*H*-pyrazolo­[4,3-*d*]­pyrimidin-5-yl)­phenyl)­sulfonyl)-*N*-(trifluoromethyl)­piperazine-1-carboxamide (**16c**)

The reaction was performed on a 0.23 mmol scale and stirred at room temperature for 18 h, using 5-(2-ethoxy-5-(piperazin-1-ylsulfonyl)­phenyl)-1-methyl-3-propyl-1,6-dihydro-7*H*-pyrazolo­[4,3-*d*]­pyrimidin-7-one (0.13 g, 0.27 mmol, 1.2 equiv) (**30**), benzyl­(trifluoromethyl)­carbamoyl fluoride (**S4**) (50 mg, 0.23 mmol, 1 equiv), DIPEA (80 μL, 0.46 mmol, 2 equiv), and 4-dimethylaminopyridine (2.8 mg, 23 μmol, 0.1 equiv) in acetonitrile (2.0 mL), following general procedure C. The title product was obtained as a colorless solid (16 mg, 23 μmol, 11%) after purification via reversed-phase preparative HPLC, with a Waters XBridge C18 5 μm ODB 19 × 150 mm column and water/acetonitrile in 0.01 M NH_4_HCO_3_ (pH 9) as the eluent phase, following the gradient: 2–94%. ^**1**^ **H NMR** (600 MHz, DMSO-*d*_6_) δ 7.79 (d, *J* = 2.4 Hz, 1H), 7.73 (dd, *J* = 8.8, 2.5 Hz, 1H), 7.40 (d, *J* = 8.9 Hz, 1H), 7.23–7.17 (m, 4H), 7.08 (tt, *J* = 6.0, 2.3 Hz, 1H), 4.32 (s, 2H), 4.25 (q, *J* = 6.9 Hz, 2H), 4.17 (s, 3H), 3.47 (t, *J* = 5.1 Hz, 4H), 2.79 (t, *J* = 7.5 Hz, 2H), 2.65–2.55 (m, 4H), 1.75 (hept, *J* = 7.4 Hz, 2H), 1.36 (t, *J* = 6.9 Hz, 3H), 0.93 (t, *J* = 7.4 Hz, 3H). ^**19**^ **F NMR** (471 MHz, DMSO-*d*_6_) δ −57.8 (s, 3F). ^**13**^ **C NMR** (151 MHz, DMSO-*d*_6_) δ 160.1, 154.0, 153.9, 148.3, 145.0, 137.9, 134.7, 131.4, 130.0, 128.8, 128.4, 127.9, 125.9, 124.5, 124.0, 121.5 (q, *J* = 259.3 Hz), 113.4, 65.0, 49.2, 45.4, 44.1, 37.9, 27.2, 21.8, 14.3, 14.8. **HRMS** (ESI): *m*/*z* calculated for C_30_H_34_F_3_N_7_O_5_S + H^+^: 662.2328 [M + H]^+^, found: 662.2362.

#### 4-((4-Ethoxy-3-(1-methyl-7-oxo-3-propyl-6,7-dihydro-1*H*-pyrazolo­[4,3-*d*]­pyrimidin-5-yl)­phenyl)­sulfonyl)-*N*-phenyl-*N*-(trifluoromethyl)­piperazine-1-carboxamide (**16d**)

The reaction was performed on a 0.12 mmol scale and stirred at room temperature for 16 h using 5-(2-ethoxy-5-(piperazin-1-ylsulfonyl)­phenyl)-1-methyl-3-propyl-1,6-dihydro-7*H*-pyrazolo­[4,3-*d*]­pyrimidin-7-one (69 mg, 0.15 mmol, 1.2 equiv) (**30**), phenyl­(trifluoromethyl)­carbamoyl fluoride (**S5**) (25 mg, 0.12 mmol, 1 equiv), DIPEA (40 μL, 0.24 mmol, 2 equiv), and 4-dimethylaminopyridine (2.9 mg, 24 μmol, 0.2 equiv) in dichloromethane (1.0 mL), following general procedure C. The title product was obtained as a colorless solid (4 mg, 7 μmol, 5%) after purification via reversed-phase preparative HPLC, with a Waters XBridge C18 5 μm ODB 19 × 150 mm column and water/acetonitrile in 0.01 M NH_4_HCO_3_ (pH 9) as the eluent phase, following the gradient: 2–94%. ^**1**^ **H NMR** (500 MHz, DMSO-*d*_6_) δ 12.27 (s, 1H), 7.71 (d, *J* = 2.4 Hz, 1H), 7.65 (dd, *J* = 8.8, 2.4 Hz, 1H), 7.37 (t, *J* = 7.7 Hz, 3H), 7.33–7.24 (m, 3H), 4.24 (q, *J* = 6.9 Hz, 2H), 4.18 (s, 3H), 3.40 (d, *J* = 5.2 Hz, 9H), 2.79 (t, *J* = 7.5 Hz, 2H), 1.75 (hept, *J* = 7.4 Hz, 2H), 1.36 (t, *J* = 6.9 Hz, 3H), 0.94 (t, *J* = 7.3 Hz, 3H). ^**19**^ **F NMR** (471 MHz, DMSO-*d*_6_) δ −57.2 (s, 3F). ^**13**^ **C NMR** (126 MHz, DMSO-*d*_6_) δ 160.0, 154.3, 153.8, 148.6, 144.9, 137.9, 136.1, 131.2, 129.9, 128.9, 128.0, 125.7, 124.5, 124.2, 120.7 (*J* = 259.3 Hz), 113.4, 79.2, 64.9, 44.7, 44.0, 37.9, 27.2, 21.7, 14.3, 13.9. **HRMS** (ESI): *m*/*z* calculated for C_29_H_32_F_3_N_7_O_5_S + H^+^: 648.2211 [M + H]^+^, found: 648.2222.

### Determination of Kinetic Solubility

The assay plates were prepared from compounds as DMSO stock solutions (10 mM). Samples (4 μL, 10 mM) were transferred into a 96-well microplate containing PBS buffer (pH = 7.4, 196 μL), affording a final concentration of 200 μM for each sample. To prevent evaporation, samples were sealed with a heat-activated aluminum sheet. The sample plates were shaken on a vibrating platform shaker for 24 h at 1000 rpm and kept at room temperature. After 24 h, the sample plates were removed from the shaker, centrifuged, and transferred (200 μL) to 96-well filter plates (Millipore Multiscreen HTS). A positive pressure manifold (Waters, Positive Pressure-96 Processor) was then utilized to filter precipitate and collect the filtrate into separate 96-well microplates. After filtration, 100 μL of filtrate was diluted into 100 μL of DMSO for a final theoretical maximum compound concentration of 100 μM. Plates were then sealed and shaken before being transferred to an LCMS-UV-CAD for analysis (Tables 1 and 2).

### Determination of Log*D*_7.4_

Experimental log*D*_7.4_ measurements were determined by a literature microscale shake-flask method.^37^ (Tables 1 and 2)

### MDCK Permeability Assay

Madin–Darby Kidney cells (MDCK) were obtained from the ATCC (Manassas, VA). CRISPR Cas9 was used to knock out the endogenous canine Mdr1 gene. Cells were maintained in Dulbecco’s Modified Eagle Medium supplemented with 10% fetal bovine serum, 1% pen-strep, and 5 μg/mL plasmocin before being seeded on Millipore Millicell-24 well plates at 2.5 × 105 cells/mL and allowed to grow for 5 days. Prior to the permeability experiment, cell monolayers were equilibrated in transport buffer (Hank’s Balanced Salt Solution with 10 mM Hepes, pH 7.4) for 60 min at 37 °C with 5% CO_2_ and 95% relative humidity. Test compound dose solutions were prepared at 10 μM in transport buffer containing the monolayer integrity marker lucifer yellow (100 μM). The dose solutions were added to the donor chambers, and transport buffer was added to all receiver chambers. The permeability was examined in the apical-to-basolateral (A:B) and basolateral-to-apical (B:A) directions. The receiver chambers were sampled at 60, 120, and 180 min and were replenished with fresh transport buffer. Lucifer yellow was measured using a fluorescence plate reader (ex: 425 nm; em: 530 nm), and compound concentrations in the donor and receiving compartments were determined by LC-MS/MS analysis. The apparent permeability (P_app_) in the A:B and B:A directions was calculated as follows:

$$P_{\mathrm{app}}=\left(\mathrm{dQ}/\mathrm{dt}\right)\cdot \left({1/AC}_{0}\right)$$

where d*Q*/d*t* is the rate of compound appearance in the receiver compartment; *A* is the surface area of the inset; and *C*_0_ is the initial substrate concentration at time 0 min (Tables 1 and 2).

### Determination of Liver Microsome Stability

All chemicals and reagents were of analytical grade, purchased from Sigma-Aldrich (St. Louis, MO) unless specified otherwise. Pooled liver microsomes were obtained from Corning (Corning, NY). A liver microsomes solution containing 0.625 mg/mL liver microsomes was prepared in 100 mM potassium phosphate buffer. A 10 mM NADPH solution was also prepared in 100 mM potassium phosphate buffer. 10 mM DMSO stock test compounds were diluted with DMSO from 10 mM to 1 mM, and then, the 1 mM compound solutions were further diluted to 10 μM working solution with 100 mM potassium phosphate buffer. 60 μL of 0.625 mg/mL liver microsomes solution was transferred to the well of a 384-well plate, and the liver microsomes solution and NADPH solution were prewarmed for 10 min at 37 °C. 7.5 μL of the NADPH solution and 7.5 μL of the compound working solution were added to start the reaction. The reaction mixture contained 1 mM NADPH, 0.5 mg/mL microsomal protein, and 1 μM test compound in 100 mM phosphate buffer. Aliquots of 12 μL of 4 test compounds (48 μL in total) were taken from the reaction solutions into one well of a new 384-well plate containing 96 μL of cold acetonitrile with internal standards (1 μM propranolol and 200 nM labetalol). Time points were taken at 20, 40, and 60 min. Samples were centrifuged at 3220 g for 40 min. An aliquot of 40 μL of the supernatant was mixed with an appropriate volume of HPLC-grade water (depending on the LC-MS/MS signal response and peak shape) for LC-MS/MS analysis done on Triple Quad 5500 (AB SCIEX, Framingham, MA). In the assay, the way samples are quenched would be able to recover what’s bound to liver microsomes, unless it is covalently bound or nonspecifically bound. For compounds that have high nonspecific binding, instead of aliquoting samples out of incubation wells and adding them into organic media, discrete incubation samples are used for each time point, and organic solvent is added directly into the wells. In the study, the controls for LM stability include Amitriptyline, Clozapine, Temozolomide, Rosiglitazone, Diclofenac, Imipramine, Metoprolol, Zoniporide, Diazepam, and Antipyrine (Tables 1 and 2).

### Determination of Stability at pH 1.0, 7.4, and 10.0

1 mL of a 5 μM solution of the respective compound in an aqueous 0.1 M HCl solution (pH 1.0), 20 mM sodium phosphate buffer (pH 7.4), or 20 mM sodium carbonate buffer (pH 10.0) was incubated at 70 °C at 300 rpm using an Eppendorf Thermomixer Comfort plate shaker. After 0, 2, 4, 8, and 24 h, an aliquot (200 μL) was taken and quenched with cold acetonitrile (800 μL containing 100 nM labetalol, 100 nM tolbutamide, and 100 nM ketoprofen as internal standards) and analyzed using a Waters Acquity UPLC H-Class/QDA equipped with a Waters Xselect HSS T3 C18 column (2.5 μm, 2.1 mm × 50 mm) with a gradient of 5–98% acetonitrile in water (modified with 0.1% formic acid) within 1.4 min at 40 °C to determine the peak area of the parent compound using a Triple Quad 6500+. The slope, k, is determined by linear regression of the natural logarithm of the peak area of the parent compound against incubation time using Microsoft Excel. The half-life *t*_1/2_ is determined using the following equation:

$$t_{1/2}=-0.693/k$$

The extrapolated half-life at 25 °C is calculated by taking the measured half-life at 70 °C and with a factor of 2 change in reaction rate for each 10 °C reduction in temperature (Table 3 and Table S2).

### Determination of Degradation Products after Incubation of *N*-Trifluoromethyl Analogues at pH 1.0, 7.4, and 10.0 for 24 h at 70 °C

A 100 μM solution of the respective compound in aqueous 0.1 M HCl (pH 1.0), 20 mM sodium phosphate buffer (pH 7.4), or 20 mM sodium carbonate buffer (pH 10.0), each containing 1% DMSO, was incubated at 70 °C for 24 h. Then, the mixture was allowed to cool to room temperature and analyzed via LC-MS using a GenTech Scientific Waters ACQ equipped with an Acquity ultra performance liquid chromatography (UPLC) system at 60 °C and a flow rate of 1 mL/min, and an SQD detector with either an acidic or basic mobile phase. For the acidic mobile phase, acetonitrile and water (modified with 1 mM ammonium formate and 10 mM formic acid, pH 3) and an HSS C18 column (1.8 μm, 2.1 × 50 mm) were used. For the basic mobile phase, acetonitrile and water (modified with 47 mM ammonia and 6.5 mM ammonium carbonate, pH 10) and a BEH C18 column (1.7 μm, 2.1 × 50 mm) were used. The exact gradient for each compound is given in the Supporting Information (Figures 1, S1–S3).

### Identification of Metabolites after Incubation of **14a–d** in Human Plasma at 37 °C for 18 h

5 μL of a 1 mM DMSO solution of the respective compound was diluted with 495 μL of plasma (Pooled Gender Human K2EDTA Plasma from BIOIVT, as per the IEC guidelines). The solution was incubated at 37 °C with 500 rpm shaking in the presence or absence of 12.5 μM Neostigmine as an inhibitor of acetylcholinesterase. At 0 and 18 h, a 50 μL aliquot was taken and quenched with 400 μL of acetonitrile containing 5,5-diethyl-2-imino-1,3-diphenyldihydropyrimidine-4,6­(1*H*,5*H*)-dione as the internal standard. A 9-point cassette calibration curve in plasma for both the compound and metabolite was generated for the respective compound over a concentration range between 70 nM and 12,500 nM. Both aliquot and calibration plates were centrifuged at 4000 rpm for 20 min. 40 μL of supernatant was taken and diluted with 40 μL of water and analyzed using an Acquity UPLC system coupled to a Xevo TQ-XS triple quadrupole mass spectrometry (MS/MS) system (Waters Corporation, Milford, MA, USA). Chromatographic separation was achieved using reversed-phase chromatography on a Waters HSS T3 column (50 mm × 2.1 mm, 1.8 μm) using a fast gradient from 0.2% to 70% acetonitrile in water (modified with 0.1% formic acid) in 1.3 min at 40 °C at 1.0 mL/min. The positive electrospray ionization-based MRM data acquisition and quantification were performed using Masslynx/targetlynx 4.2 software (Figure 2).

### Measurements of Shake Flask log*D*_7.4_, Solubility, Stability in Human Plasma, Human Plasma Protein Binding, and Metabolic Stability in Human Liver Microsomes and Rat Hepatocytes

Log*D*_7.4_, solubility, human plasma protein binding, and stability in human liver microsomes, human plasma, and rat hepatocytes were performed according to Wernevik et al.^38^ (Table 4)

### Determination of Compound Free Fraction in Incubations

The free fraction of compounds in HLM (1 mg/mL) and rat hepatocytes (1 million cells/mL) was determined by equilibrium dialysis in an HTD96B device, as described by Austin et al.^39,40^

### Measurement of Stability in Human Hepatocytes

The stability of compounds in human hepatocytes was determined according to Jacobson et al.^41^ (Table 4)

### Chromlog*D*_7.4_ Measurements

A 0.5 mm DMSO solution (1 μL) of the compound is analyzed by LC-MS using a Waters Acquity with a BEH C18 column (1.7 μm, 50 mm × 2.1 mm) using a gradient from 0% to 100% acetonitrile/H_2_O 95:5 in acetonitrile/H_2_O 5:95 (adjusted with ammonia to pH 7.4) within 1.76 min, with a flow rate of 1.0 mL/min at 40 °C. The retention factor *k*′ is calculated from the measured retention time *r*_*t*_ of the compound using the following formula:

$$k^{\prime }=\left(r_{t}-t_{0}\right)/t_{0}$$

with *t*_0_ being the retention time of DMSO. Metoprolol, warfarin, propranolol, chlorpromazine, and felodipine are used as standards. A calibration curve by plotting the measured k′ of the standards against their literature-known log*D*_7.4_ values is used to transform the determined *k*′ of the sample into chromlog*D*_7.4_ (Table 4).

### Caco-2 Measurements

Caco-2 measurements were performed by the DMPK department at Pharmaron, as described by Fredlund et al.^42^ (Table 4)

### CYP Inhibition Assay

The inhibition of CYP3A4, 2D6, 2C9, 2C19, and 1A2 was assessed by Pharmaron, as described by Terstiege et al.^43^

### *In Vivo* Rat PK Study

Rat PK experiments were conducted at Pharmaron, China. The animals were housed in an Association for Assessment and Accreditation of Laboratory Animal Care (AAALAC)-accredited animal facility (accreditation number: 001322) in a controlled environment (20–25 °C and 40–70% relative humidity) with a 12 h light/12 h dark cycle with free access to food and water. Pharmaron’s (Beijing Co., Ltd., China) Institutional Animal Care and Use Committee (IACUC) approved the studies, and the animal experiments conform to all relevant regulatory standards. Samples were dissolved in 5% DMSO, 95% SBE-B-CD (30% w/v) at 0.5 mg/kg for IV dosing, and 100% (0.5% HPMC, 0.1% Tween in water) at 0.5 mg/kg (for **15e** and **15f**) or 1 mg/kg (for **15a**, **15b**, **15g**, and **15h**) for PO dosing, before being administered in cassettes of five compounds, to male Han Wistar rats, using two animals per route of administration. IV dosing was performed via the tail vein, and oral dosing was performed via oral gavage. Blood samples were collected from the dorsal metatarsal vein at different time points after administration (0.03, 0.08, 0.17, 0.5, 1, 2, 4, 8, and 24 h for IV and 0.08, 0.25, 0.5, 1, 2, 4, 8, and 24 h) and treated with EDTA-K2 to avoid coagulation. The samples were centrifuged at 4,000 g for 5 min at 4 °C to obtain plasma that was stored at −75 ± 15 °C prior to analysis. After protein precipitation, the plasma concentration was determined by LC/MS/MS, and PK parameters were calculated using NCA data analysis (Table 5).

## Supplementary Material





## Notes

Molecular formula strings and associated data­(CSV)
Additional experimental details, NMR spectra for novel compounds and purity analyses (PDF)

## Abbreviations

AChE: acetylcholinesterase
AUC: area under the curve
BCRP: breast cancer resistance protein
BTC: bis­(trichloromethyl)­carbonate
Caco-2: human epithelial colorectal adenocarcinoma cells
chromlog*D*_7.4_: chromatographically determined logarithm of distribution coefficient at pH 7.4
CL_int_: intrinsic clearance
DIPEA: diisopropylethylamine
DMAP: 4-(dimethylamino)­pyridine
DMPU: *N*,*N*′-dimethylpropyleneurea
f_u,inc_: unbound fraction in the incubation system
HLM: human liver microsomes
HLM-N: human liver microsomes under NADPH-free conditions
hPPB: human plasma protein binding
Hu Heps: human hepatocytes
KHMDS: potassium hexamethyldisilazide
LC-MS: liquid chromatography-mass spectrometry
MDCK: Madin Darby canine kidney
MMP: matched molecular pair
MRP2: multidrug-associated protein 2
NADPH: reduced form of nicotinamide adenine dinucleotide phosphate
P_app_: apparent permeability
P-gp: P-glycoprotein
Rat Heps: rat hepatocytes
TEA: triethylamine
TCDI: 1,1′-thiocarbonyldiimidazole
V_ss,u_: unbound volume of distribution at steady state

## References

[1] John Wiley & Sons Inc. The Amide Linkage: Structural Significance in Chemistry, Biochemistry, and Materials Science, 1st ed.; Greenberg, A. ; Breneman, C. M. ; Liebman, J. F. Eds.; John Wiley & Sons Inc.: New York, 1999.

[2] Pattabiraman V. R., Bode J. W. (2011). Rethinking amide bond synthesis. Nature.

[3] Schneider N., Lowe D. M., Sayle R. A., Tarselli M. A., Landrum G. A. (2016). Big Data
from Pharmaceutical Patents: A Computational Analysis of Medicinal
Chemists’ Bread and Butter. J. Med. Chem..

[4] Metcalf, R. L. Insect Control. In Ullmann’s Encyclopedia of Industrial Chemistry. Wiley 2000.

[5] Vardanyan, R. ; Hruby, V. Chapter 23 - Drugs for Treating Respiratory System Diseases In Synthesis of Best-Seller Drugs. Vardanyan, R. ; Hruby, V. Eds.; Academic Press, 2016 pp. 357–381.

[6] Holland J. R., Hosley H., Scharlau C., Carbone P. P., Frei E., Brindley C. O., Hall T. C., Shnider B. I., Gold G. L., Lasagna L. (1966). A controlled trial of
urethane treatment in multiple myeloma. Blood.

[7] Sijbesma R.
P., Beijer F. H., Brunsveld L., Folmer B. J., Hirschberg J. H., Lange R. F., Lowe J. K., Meijer E. W. (1997). Reversible Polymers
Formed from Self-Complementary Monomers Using Quadruple Hydrogen Bonding. Science.

[8] Ball, P. Navigating chemical space. 2015. https://www.chemistryworld.com/features/navigating-chemical-space/8983.article Accessed 16 December 2022.

[9] Schiesser S., Cox R. J., Czechtizky W. (2021). The powerful
symbiosis between synthetic
and medicinal chemistry. Future Med. Chem.

[10] Campos K. R., Coleman P. J., Alvarez J. C., Dreher S. D., Garbaccio R. M., Terrett N. K., Tillyer R. D., Truppo M. D., Parmee E. R. (2019). The importance
of synthetic chemistry in the pharmaceutical industry. Science.

[11] Chatterjee J., Gilon C., Hoffman A., Kessler H. (2008). *N*-Methylation
of Peptides: A New Perspective in Medicinal Chemistry. Acc. Chem. Res.

[12] Müller K., Faeh C., Diederich F. (2007). Fluorine in Pharmaceuticals: Looking
Beyond Intuition. Science.

[13] Purser S., Moore P. R., Swallow S., Gouverneur V. (2008). Fluorine in
medicinal chemistry. Chem. Soc. Rev.

[14] Isanbor C., O’Hagan D. (2006). Fluorine in
medicinal chemistry: A review of anti-cancer
agents. J. Fluorine Chem.

[15] Clayden J. (2019). Fluorinated
compounds present opportunities for drug discovery. Nature.

[16] Milcent T., Crousse B. (2018). The main and recent syntheses of the *N*-CF_3_ motif. C. R. Chim..

[17] Scattolin T., Bouayad-Gervais S., Schoenebeck F. (2019). Straightforward access to *N*-trifluoromethyl
amides, carbamates, thiocarbamates and
ureas. Nature.

[18] Zhang Z., He J., Zhu L., Xiao H., Fang Y., Li C. (2020). Silver-Mediated *N*-Trifluoromethylation of Amides and Peptides. Chin. J. Chem.

[19] Liu J., Parker M. F. L., Wang S., Flavell R. R., Toste F. D., Wilson D. M. (2021). Synthesis of *N*-trifluoromethyl amides
from carboxylic acids. Chem.

[20] Liu S., Huang Y., Wang J., Qing F.-L., Xu X.-H. (2022). General
Synthesis of *N*-Trifluoromethyl Compounds with *N*-Trifluoromethyl Hydroxylamine Reagents. J. Am. Chem. Soc.

[21] Luo D., Ruan L., Xue J., Jiang Z., Nie C., Zeng T., Luo L., Li J. (2025). Bench-Stable Carbamoylzinc
Pivalates for Modular Access to Amides, Ureas, and Thiocarbamates. J. Am. Chem. Soc.

[22] Zhang R. Z., Liu Y., Xu C., Wang M. (2025). Direct synthesis of N-trifluoromethyl
amides via photocatalytic trifluoromethylamidation. Nat. Commun.

[23] Zivkovic F. G., Nielsen C. D.-T., Schoenebeck F. (2022). Access to *N*-CF_3_ Formamides by Reduction of *N*-CF_3_ Carbamoyl Fluorides. Angew.
Chem., Int. Ed.

[24] Nielsen C. D.-T., Zivkovic F. G., Schoenebeck F. (2021). Synthesis of *N*-CF_3_ Alkynamides and Derivatives Enabled by Ni-Catalyzed Alkynylation
of *N*-CF_3_ Carbamoyl Fluorides. J. Am. Chem. Soc.

[25] Bouayad-Gervais S., Scattolin T., Schoenebeck F. (2020). *N*-Trifluoromethyl Hydrazines, Indoles
and Their Derivatives. Angew. Chem., Int. Ed.

[26] Bouayad-Gervais S., Nielsen C. D. T., Turksoy A., Sperger T., Deckers K., Schoenebeck F. (2022). Access to
Cyclic *N*-Trifluoromethyl Ureas through Photocatalytic
Activation of Carbamoyl Azides. J. Am. Chem.
Soc.

[27] Zivkovic F. G., Wycich G., Liu L., Schoenebeck F. (2024). Access to *N*-Difluoromethyl Amides,
(Thio)­Carbamates, Ureas, and Formamides. J.
Am. Chem. Soc.

[28] Zivkovic F. G., Bahns F., Hsu C.-M., Schoenebeck F. (2025). Access to *N*-Monofluoromethylated (Thio)­Carbamates,
Formamides, Alkynamides, and Related Derivatives. Angew. Chem., Int. Ed.

[29] Yang Z., Shi S., Fu L., Lu A., Hou T., Cao D. (2023). Matched Molecular
Pair Analysis in Drug Discovery: Methods and Recent Applications. J. Med. Chem.

[30] Perry M. W. D., Börjesson U., Nikitidis A., Tyrchan C. (2022). Surprising lipophilicity observations identify unexpected
conformational effects. Bioorg. Med. Chem. Lett.

[31] Landry M. L., Trager R., Broccatelli F., Crawford J. J. (2022). When Cofactors Aren’t
X Factors: Functional Groups That Are Labile in Human Liver Microsomes
in the Absence of NADPH. ACS Med. Chem. Lett.

[32] Leung C., Liu J., Cunico K., Johnson K., Yan Z., Cai J. (2024). An Integrated
Hepatocyte Stability Assay for Simultaneous Metabolic Stability Assessment
and Metabolite Profiling. Drug Metab. Dispos.

[33] Chen E. C., Broccatelli F., Plise E., Chen B., Liu L., Cheong J., Zhang S., Jorski J., Gaffney K., Umemoto K. K. (2018). Evaluating the Utility of Canine Mdr1 Knockout
Madin-Darby Canine Kidney I Cells in Permeability Screening and Efflux
Substrate Determination. Mol. Pharmaceutics.

[34] Schiesser S., Chepliaka H., Kollback J., Quennesson T., Czechtizky W., Cox R. J. (2020). *N*-Trifluoromethyl
Amines and Azoles: An Underexplored Functional Group in the Medicinal
Chemist’s Toolbox. J. Med. Chem.

[35] Zheng M., Liu M., Ma F., Song Z., Zhu H., Guo H., Sun H. (2025). Novel colorimetric-fluorescent
dual-mode biosensing platform for
detecting acetylcholinesterase and screening acetylcholinesterase
inhibitors based on trimetallic nanozymes. Chem.
Eng. J.

[36] Gardner I., Xu M., Han C., Wang Y., Jiao X., Jamei M., Khalidi H., Kilford P., Neuhoff S., Southall R. (2022). Non-specific
binding of compounds in in vitro metabolism assays:
a comparison of microsomal and hepatocyte binding in different species
and an assessment of the accuracy of prediction models. Xenobiotica.

[37] Lin B., Pease J. H. (2013). A Novel Method for High Throughput Lipophilicity Determination
by Microscale Shake Flask and Liquid Chromatography Tandem Mass Spectrometry. Comb. Chem. High Throughput Screening.

[38] Wernevik J., Bergström F., Novén A., Hulthe J., Fredlund L., Addison D., Holmgren J., Strömstedt P.-E., Rehnström E., Lundbäck T. (2020). A Fully Integrated Assay Panel for
Early Drug Metabolism and Pharmacokinetics Profiling. Assay Drug Dev. Technol.

[39] Austin R. P., Barton P., Cockroft S. L., Wenlock M. C., Riley R. J. (2002). The Influence
of Nonspecific Microsomal Binding on Apparent Intrinsic Clearance,
and Its Prediction from Physicochemical Properties. Drug Metab. Dispos.

[40] Austin R. P., Barton P., Mohmed S., Riley R. J. (2005). The Binding of Drugs
to Hepatocytes and Its Relationship to Physicochemical Properties. Drug Metab. Dispos.

[41] Jacobson L., Middleton B., Holmgren J., Eirefelt S., Fröjd M., Blomgren A., Gustavsson L. (2007). An Optimized Automated Assay for
Determination of Metabolic Stability Using Hepatocytes: Assay Validation,
Variance Component Analysis, and In Vivo Relevance. Assay Drug Dev. Technol.

[42] Fredlund L., Winiwarter S., Hilgendorf C. (2017). In Vitro Intrinsic Permeability:
A Transporter-Independent Measure of Caco-2 Cell Permeability in Drug
Design and Development. Mol. Pharmaceutics.

[43] Terstiege I., Aagaard A., Berggren K., Bird J., Cumming I. A., Groombridge S. D., Hidestål L., Johannesson P., Korsgren P., Leuchowius K.-J. (2025). Generation of a potent
& selective series of IRAK4 inhibitors based on a structure based,
hybridization approach. Bioorg. Med. Chem.

