# Supplementary material for: Charting New Territory: Systematic Evaluation of the Drug Potential of N‑Trifluoromethyl Amides, Ureas & Carbamates
Source: J Med Chem. 2026 Jul 3;69(14):17319–41. doi: 10.1021/acs.jmedchem.6c01299 (PMC13403232; doi:10.1021/acs.jmedchem.6c01299)
Supplement: Supplementary file 2 [file jm6c01299_si_002.pdf]

Supporting Information

Charting New Territory: Systematic Evaluation of the Drug Potential of *N*-Trifluoromethyl Amides, Ureas & Carbamates

Jacopo Garonzi,[a,b,c] Gina Wycich,[c] Stefanie Zich,[a,b] Shakiba Vahdat,[a,b] Beatriz Carvalho,[a,b] Guillaume Benoit,[a] Vijay K. Ahuja,[d] Claire Le Manach,[e] Hyeonglim Seo,[f] Lionel E. Cheruzel,[f] Christian Gampe,[g] Xingyu Jiang,*,[g] Franziska Schoenebeck*,[c], and Stefan Schiesser*,[a]

[a] BioPharma Chemistry, Discovery Sciences, BioPharmaceuticals R&D, AstraZeneca, Pepparedsleden 1, 43183 Mölndal, Sweden

[b] Department of Chemistry and Molecular Biology, Göteborgs Universitet, Medicinaregatan 7B, 41390 Göteborg, Sweden

[c] Institute of Organic Chemistry, RWTH Aachen University, Landoltweg 1, 52074 Aachen, Germany

[d] Assay Profiling and Cell Sciences, Discovery Sciences, BioPharmaceuticals R&D, AstraZeneca, Pepparedsleden 1, 43183 Mölndal, Sweden

[e] DMPK, Research and Early Development, Respiratory and Immunology (R&I), BioPharmaceuticals R&D, AstraZeneca, Pepparedsleden 1, 43183 Mölndal, Sweden

[f] Drug Metabolism and Pharmacokinetics Department, Genentech, Inc., 1 DNA Way, South San Francisco, CA 94080, USA

[g] Discovery Chemistry Department, Genentech, Inc., 1 DNA Way, South San Francisco, CA 94080, USA

*Corresponding authors. Email: jiang.xingyu@gene.com (Xingyu Jiang), franziska.schoenebeck@rwth-aachen.de (Franziska Schoenebeck), stefan.schiesser@astrazeneca.com (Stefan Schiesser).

Table of contents

1. Synthetic Protocols S4

1.1 Synthesis of carbamoyl fluorides S4

1.1.1 General procedure G for the synthesis of *N*-trifluoromethylcarbamoyl fluorides S4

1.1.2 Characterization data of carbamoyl fluorides S4

1.2 Synthesis of *N*-CF_3 amides S5

1.2.1 General procedure H for the synthesis of *N*-CF_3 amides **3c**–**4c** S5

1.2.2 Characterization data of the *N*-CF_3 amides S5

1.3 Synthesis of *N*-CF_3 ureas and carbamates S6

1.3.1 General procedure I for the synthesis of *N*-CF_3 ureas and carbamates **5c**–**6c**–**7c**–**8c**–**9b** S6

1.3.2 Characterization data of the *N*-CF_3 ureas and carbamates S6

1.4 Synthesis of *N*-Methyl analogues **3b**–**4b**–**5b**–**6b**–**7b**–**8b**–**9a** of compounds **3c**–**4d**–**5c**–**6c**–**7c**–**8c**–**9b** S8

1.4.1 General procedure J for the synthesis of *N*-methyl amides **3b**–**4b** S8

1.4.2 General procedure K for the synthesis of *N*-methyl ureas **5b**–**6b**–**7b**–**8b** S8

1.4.3 Characterization data of compounds **3b**–**4b**–**5b**–**6b**–**7b**–**8b** S9

1.4.4 Synthesis and characterization data of *N*-Me analogue **9a** of compound **9b** S11

1.5 Synthesis of *N*-Methyl analogues **S14**–**S15** of compound series **10a**–**d** S11

1.5.1 General procedure L for the synthesis of compounds **S14**–**S15** S11

1.5.2 Characterization data of compounds **S14**–**S15** S12

1.6 Synthesis of *N*-Me analogues **S17**–**S18** of compound series **12a**–**d** S13

1.6.1 General procedure M for the synthesis of compounds **S17**–**S18** S13

1.6.2 Characterization data of *N*-Me and *N*-^{i}Pr analogues of compound series **12a**–**d** S13

1.7 Synthesis of *N*-Me analogues **S19**–**S20**–**S21**–**S22** of compound series **13a**–**d** S14

1.7.1 General procedure N for the synthesis of compound series **S19**–**S20**–**S21** S14

1.7.2 Characterization data of compound series **S19**–**S20**–**S21** S14

1.7.3 Synthesis and characterization data of 2-(4-(2-(5-chloro-2-oxobenzo[*d*]thiazol-3(2*H*)-yl)acetyl)piperazin-1-yl)ethyl methyl(phenyl)carbamate (**S22**) S15

1.8 Synthesis of *N*-Me analogues **S23**–**S24**–**S25**–**S26** of compound series **14a**–**d** S16

1.8.1 General procedure O for the synthesis of compounds **S23**–**S24** S16

1.8.2 General procedure P for the synthesis of compounds **S25**–**S26** S16

1.8.3 Characterization data of compounds **S23**–**S24**–**S25**–**S26** S16

1.9 Synthesis of *N*-H analogues **3a**–**8a** S18

1.9.1 General procedure Q for the synthesis of **3a**–**4a** S18

1.9.2 General procedure R for the synthesis of **5a**–**6a**–**7a**–**8a** S18

1.9.3 Characterization data of compounds **3a**–**4a**–**5a**–**6a**–**7a**–**8a** S18

1.10 Miscellaneous S20

1.10.1 General procedure S for the synthesis of **S12**–**S13** S20

1.10.2 Characterization data of compounds **S12**–**S13** S21

2. Biological assays........ S21

2.1 Metabolic profiling for compounds **3c**, **4c**, **6c**, **7c**, and **9b** S21

2.1.1 Metabolite Identification........ S21

2.1.2 Procedures S24

2.2 Stability of *N*-methyl analogs in aqueous media and human plasma S25

2.3 Determination of degradation products after incubation of *N*-trifluoromethyl analogs **10**–**16** at pH 1.0, 7.4, and 10.0 for 24 h at 70 °C........ S26

2.4 In vivo rat PK of *N*-trifluoromethyl compounds **15a** and **15b** and their *N*-methyl (**15e**, **15g**) and *N*-isopropyl analogs (**15f**, **15h**)........ S31

3. NMR Spectra........ S33

3.1 Isothiocyanates S33

3.2 *N*-CF_3 carbamoyl fluorides........ S35

3.3 *N*-CF_3 amides S41

3.4 *N*-CF_3 ureas and carbamates........ S56

3.5 *N*-Me and *N*-^{i}Pr analogues........ S94

3.6 *N*-H analogues........ S127

3.7 Miscellaneous........ S133

4. Purity analysis........ S138

5. References S152

1. Synthetic Protocols

1.1 Synthesis of carbamoyl fluorides

1.1.1 General procedure G for the synthesis of *N*-trifluoromethylcarbamoyl fluorides

Based on previously reported procedure:[1] A 20 mL vial was charged with the corresponding isothiocyanate (2.0 mmol, 1 equiv.), silver(I) fluoride (1.3 g, 10 mmol, 5 equiv.) and bis(trichloromethyl) carbonate (BTC) (0.24 g, 0.80 mmol, 0.4 equiv.). Acetonitrile (10 mL) was added quickly, and the vial was sealed (if the isothiocyanate was a liquid or an oil it was added as a solution in the solvent). The mixture was stirred at 50 °C for 16 h. Afterwards, the crude mixture was added at once to Et_2O (40 mL) and stirred for 10 minutes. The solid was filtered through a pad of celite and the solvent mixture was then evaporated. The crude material was redissolved in Et_2O and refiltered through a pad of celite, to remove the last traces of salt byproducts. The *N*-trifluoromethylcarbamoyl fluoride was then obtained in a technical grade purity.

1.1.2 Characterization data of carbamoyl fluorides

_N_-(Trifluoromethyl)(3,4,5-trimethoxyphenyl)carbamoyl fluoride (S8)

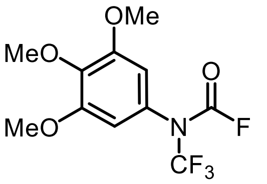

The reaction was performed on a 2.00 mmol scale, using 5-isothiocyanato-1,2,3-trimethoxybenzene (0.45 g, 2.0 mmol, 1 equiv.), following general procedure G. The title product was obtained after filtration as a colourless solid (0.53 g, 1.8 mmol, 90%).

^{1}H NMR (600 MHz, $CDCl_3$) δ 6.52 (s, 2H), 3.88 (s, 3H), 3.87 (s, 6H). **^{19}F NMR** (376 MHz, $CDCl_3$) δ −3.0 (s, 1F), −56.5 (br s, 3F). **^{13}C NMR** (151 MHz, $CDCl_3$) δ 154.0, 142.3 (d, *J* = 298.5 Hz), 139.8, 128.4, 119.4 (q, *J* = 265.0 Hz), 105.9, 61.1, 56.5. **HRMS** (EI): *m/z* calculated for $C_{11}H_{11}NF_4O_4$: 297.0619 $[M]^+$, found: 297.0624.

(4-Methoxybenzyl)-_N_-(trifluoromethyl)carbamoyl fluoride (S9)

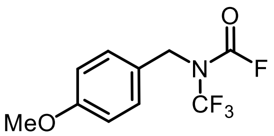

The reaction was performed on a 2.00 mmol scale, using 1-(isothiocyanatomethyl)-4-methoxybenzene (0.31 mL, 2.00 mmol, 1 equiv.), following general procedure G. The title product was obtained after filtration as a light yellow oil (0.46 g, 1.8 mmol, 91%).

^{1}H NMR (400 MHz, $CDCl_3$) δ 7.25 (d, *J* = 8.5 Hz, 2H), 6.88 (d, *J* = 8.7 Hz, 2H), 4.65 (q, *J* = 2.1 Hz, 2H), 3.80 (s, 3H). **^{19}F NMR** (565 MHz, $CDCl_3$) δ −7.2 (s, 1F), −54.9 (br s, 3F). **^{13}C NMR** (151 MHz, $CDCl_3$) δ 160.0, 143.2 (d, *J* = 301.0 Hz), 129.6, 126.5, 119.5 (q, *J* = 265.3 Hz), 114.5, 55.4, 49.4. **HRMS** (EI): *m/z* calculated for $C_{10}H_9NF_4O_2$: 251.0564 $[M]^+$, found: 251.0563.

1.2 Synthesis of *N*-CF_3 amides

1.2.1 General procedure H for the synthesis of *N*-CF_3 amides 3c–4c

Based on previously reported procedure:[1] A 4 mL vial was charged with the corresponding *N*-trifluoromethylcarbamoyl fluoride (0.20 mmol, 1 equiv.), dry toluene (1.5 mL), purged with argon and placed into a water bath at room temperature unless stated otherwise. The corresponding Grignard reagent (0.24 mmol, 1.2 equiv.) was subsequently rapidly added to the solution unless stated otherwise. The reaction mixture was stirred for 10 minutes at room temperature unless stated otherwise. Saturated aqueous ammonium chloride solution (1.5 mL) was then added. The phases were separated and the aqueous phase was further extracted with EtOAc (2 ×), dried over $MgSO_4$ and concentrated under reduced pressure. The crude material was then purified by column chromatography on silica gel using the indicated solvent system.

1.2.2 Characterization data of the *N*-CF_3 amides

4-(Morpholinomethyl)-*N*-(trifluoromethyl)-*N*-(3,4,5-trimethoxyphenyl)benzamide (3c)

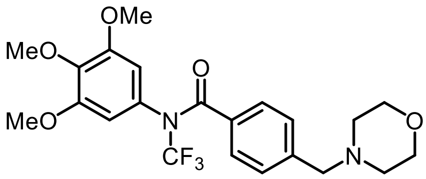

The reaction was performed on a 0.20 mmol scale, using *N*-(trifluoromethyl)(3,4,5-trimethoxyphenyl)carbamoyl fluoride (**S8**) (59 mg, 0.20 mmol, 1 equiv.) and 4-[(4-morpholino)methyl]phenylmagnesium bromide (0.24 mmol, 1.2 equiv.), following general procedure H. The title product was obtained as a yellow solid (61 mg, 14 µmol, 67%), after purification via silica gel column chromatography (1:1 *n*-pentane/EtOAc), followed by normal-phase preparative HPLC, with a Knauer Azura system, using a Merck LiChrosorb® Si60 7 µm, 250 × 25 mm column, and dichloromethane/ethanol 95:5 as eluent phase, in an isocratic elution.

^{1}H NMR (600 MHz, $CDCl_3$) δ 7.47 (d, J = 8.0 Hz, 2H), 7.24 (d, J = 7.9 Hz, 2H), 6.46 (s, 2H), 3.73 (s, 6H), 3.71 (s, 3H), 3.62 (t, J = 4.6 Hz, 4H), 3.42 (s, 2H), 2.34 (t, J = 4.6 Hz, 4H). **^{19}F NMR** (376 MHz, $CDCl_3$) δ −57.1 (s, 3F). **^{13}C NMR** (151 MHz, $CDCl_3$) δ 170.1, 153.4, 142.2, 138.8, 132.6, 131.7, 128.8, 128.7, 120.6 (q, J = 264.7 Hz), 107.8, 67.0, 62.9, 61.0, 56.4, 53.7. **HRMS** (EI): m/z calculated for $C_{22}H_{25}N_2F_3O_5$: 454.1710 $[M]^+$, found: 454.1711.

***N*-(Trifluoromethyl)-*N*-(3,4,5-trimethoxyphenyl)cyclopropanecarboxamide (4c)**

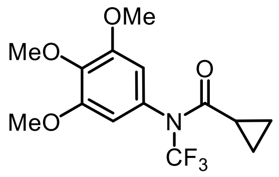

The reaction was performed on a 0.20 mmol scale, using *N*-(trifluoromethyl)(3,4,5-trimethoxyphenyl)carbamoyl fluoride (**S8**) (59 mg, 0.20 mmol, 1 equiv.) and cyclopropylmagnesium bromide 0.5 M in THF (0.48 mL, 0.24 mmol, 1.2 equiv.), following general procedure H. The title product was obtained as a light yellow solid (26 mg, 80 µmol, 40%), after purification via silica gel column chromatography (85:15 *n*-hexane/EtOAc), followed by normal-phase preparative HPLC, with a Knauer Azura system, using a Merck LiChrosorb® Si60 7 µm, 250 × 25 mm column, and *n*-hexane/EtOAc 6:4 as eluent phase, in an isocratic elution.

^{1}H NMR (600 MHz, $CDCl_3$) δ 6.55 (s, 2H), 3.88 (s, 3H), 3.87 (s, 6H), 1.42 (tt, J = 8.0, 4.5 Hz, 1H), 1.14 – 1.09 (m, 2H), 0.78 (dt, J = 7.2, 3.6 Hz, 2H). **^{19}F NMR** (376 MHz, $CDCl_3$) δ −54.9 (s, 3F). **^{13}C NMR** (151 MHz, $CDCl_3$) δ 173.8, 153.8, 139.1, 131.0, 120.0 (q, J = 263.6 Hz), 107.1, 61.0, 56.4, 13.8 (q, J = 2.5 Hz), 10.2. **HRMS** (EI): m/z calculated for $C_{14}H_{16}O_4NF_3$: 319.1026 $[M]^+$, found: 319.1027.

1.3 Synthesis of *N*-CF_3 ureas and carbamates

1.3.1 General procedure I for the synthesis of *N*-CF_3 ureas and carbamates 5c–6c–7c–8c–9b

Based on our previously reported procedure:[1] A 4 mL vial was charged with the corresponding *N*-trifluoromethylcarbamoyl fluoride (0.20 mmol, 1 equiv.) and CH_2Cl_2 (1.5 mL). The corresponding amine or alcohol (0.24 mmol, 1.2 equiv.), DIPEA (40 µL, 0.24 mmol, 1.2 equiv.) and DMAP (2.4 mg, 20 µmol, 0.1 equiv.) were subsequently added to the solution. The reaction mixture was stirred for 15 h at room temperature (unless otherwise stated). Hexane (1 mL) was then added and the reaction mixture was filtered through a pad of celite before evaporation. The crude residue was then purified by column chromatography on silica gel, using the indicated solvent system.

1.3.2 Characterization data of the *N*-CF_3 ureas and carbamates

3-Hydroxy-*N*-(4-methoxybenzyl)-*N*-(trifluoromethyl)azetidine-1-carboxamide (5c)

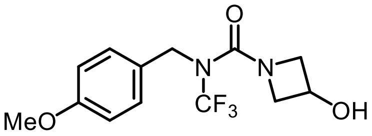

The reaction was performed on a 0.20 mmol scale, using (4-methoxybenzyl)-*N*-(trifluoromethyl)carbamoyl fluoride (**S9**) (50 mg, 0.20 mmol, 1 equiv.) and azetidin-3-ol hydrochloride (26 mg, 0.24 mmol, 1.2 equiv.), following general procedure I. The title product was obtained as a colourless solid (11 mg, 36 µmol, 18%), after purification via silica gel column chromatography (1:1 *n*-hexane/EtOAc), followed by normal-phase preparative HPLC, with a Knauer Azura system, using a Merck LiChrosorb® Si60 7 µm, 250 × 25 mm column, and *n*-hexane/EtOAc 3:7 as eluent phase, in an isocratic elution.

^{1}H NMR (600 MHz, $CDCl_3$) δ 7.21 (d, J = 8.5 Hz, 2H), 6.86 (d, J = 8.7 Hz, 2H), 4.55 (tt, J = 6.7, 4.4 Hz, 1H), 4.50 (s, 2H), 4.22 (dd, J = 10.0, 6.9 Hz, 2H), 3.88 (dd, J = 10.1, 4.4 Hz, 2H), 3.79 (s, 3H), 2.99 – 2.52 (m, 1H). **^{19}F NMR** (564 MHz, $CDCl_3$) δ −54.9 (s, 3F). **^{13}C NMR** (151 MHz, $CDCl_3$) δ 159.2, 156.1, 128.8, 121.6 (q, J = 260.7 Hz), 114.1, 61.4, 60.8, 55.4, 48.1. **HRMS** (EI): *m/z* calculated for $C_{13}H_{15}O_3N_2F_3Na$: 327.0927 $[M + Na]^+$, found: 327.0931.

3-Hydroxy-*N*-(4-methoxybenzyl)-*N*-(trifluoromethyl)piperidine-1-carboxamide (6c)

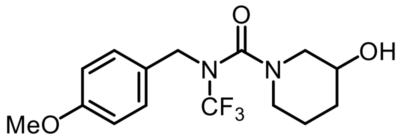

The reaction was performed on a 0.20 mmol scale, using (4-methoxybenzyl)-*N*-(trifluoromethyl)carbamoyl fluoride (**S9**) (50 mg, 0.20 mmol, 1 equiv.) and 3-hydroxypiperidine (24 mg, 0.24 mmol, 1.2 equiv.), following general procedure I. The title product was obtained as a colourless oil (22 mg, 67 µmol, 33%), after purification via silica gel column chromatography (1:1 *n*-hexane/EtOAc), followed by normal-phase preparative HPLC, with a Knauer Azura system, using a Merck LiChrosorb® Si60 7 µm, 250 × 25 mm column, and ethanol as eluent phase.

^{1}H NMR (600 MHz, $CDCl_3$) δ 7.23 (d, J = 8.5 Hz, 2H), 6.84 (d, J = 8.6 Hz, 2H), 4.32 (d, J = 1.7 Hz, 2H), 3.78 (s, 3H), 3.75 (dd, J = 13.1, 3.0 Hz, 1H), 3.57 (dt, J = 12.6, 4.7 Hz, 1H), 3.46 (tt, J = 7.7, 3.7 Hz, 1H), 3.12 (ddd, J = 12.6, 9.1, 3.1 Hz, 1H), 3.05 (dd, J = 12.9, 7.7 Hz, 1H), 1.96 (s, 1H), 1.78 (ddt, J = 10.7, 6.8, 3.5 Hz, 1H), 1.59 (dtt, J = 13.5, 6.9, 3.8 Hz, 1H), 1.44 (dtd, J = 12.8, 9.0, 4.0 Hz, 1H), 1.20 (dtt, J = 13.2, 9.1, 3.8 Hz, 1H). **^{19}F NMR** (564 MHz, $CDCl_3$) δ −59.2 (s, 3F). **^{13}C NMR** (151 MHz, $CDCl_3$) δ 159.5, 155.4, 130.4, 127.5, 121.8 (q, J = 260.2 Hz), 114.0, 66.0, 55.4, 51.8, 48.9, 46.0, 32.3, 22.2. **HRMS** (EI): m/z calculated for $C_{15}H_{19}O_3N_2F_3Na$: 355.1240 [M + Na]$^+$, found: 355.1233.

N-(4-Methoxybenzyl)-4-methyl-3-oxo-N-(trifluoromethyl)piperazine-1-carboxamide (7c)

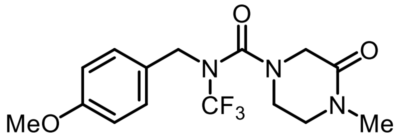

The reaction was performed on a 0.20 mmol scale, using (4-methoxybenzyl)-N-(trifluoromethyl)carbamoyl fluoride (**S9**) (50 mg, 0.20 mmol, 1 equiv.) and 1-methylpiperazin-2-one hydrochloride (36 mg, 0.24 mmol, 1.2 equiv.), following general procedure I. The title product was obtained as a colourless oil (26 mg, 74 µmol, 37%), after purification via silica gel column chromatography (EtOAc), followed by normal-phase preparative HPLC, with a Knauer Azura system, using a Merck LiChrosorb® Si60 7 µm, 250 × 25 mm column, and dichloromethane/ethanol 95:5 as eluent phase, in an isocratic elution.
^{1}H NMR (600 MHz, $CDCl_3$) δ 7.19 (d, J = 8.6 Hz, 2H), 6.82 (d, J = 8.7 Hz, 2H), 4.30 (s, 2H), 4.06 (s, 2H), 3.77 (s, 3H), 3.60 (t, J = 5.5 Hz, 2H), 3.00 (t, J = 5.5 Hz, 2H), 2.84 (s, 3H). **^{19}F NMR** (564 MHz, $CDCl_3$) δ −59.6 (s, 3F). **^{13}C NMR** (151 MHz, $CDCl_3$) δ 164.2, 159.6, 154.3, 130.4, 126.6, 121.5 (q, J = 261.0 Hz), 114.1, 55.3, 49.1, 49.0 (q, J = 2.0 Hz), 47.6, 41.8, 34.3. **HRMS** (EI): m/z calculated for $C_{15}H_{20}O_2N_3F_3$: 345.1300 [M]$^+$, found: 345.1293.

3-(Cyanomethyl)-1-(4-methoxybenzyl)-1-(trifluoromethyl)urea (8c)

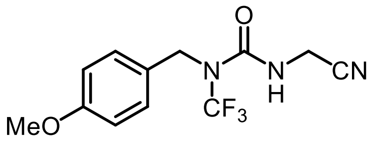

The reaction was performed on a 0.20 mmol scale, using (4-methoxybenzyl)-N-(trifluoromethyl)carbamoyl fluoride (**S9**) (50 mg, 0.20 mmol, 1 equiv.) and 2-aminoacetonitrile hydrochloride (22 mg, 0.24 mmol, 1.2 equiv.), following general procedure I. The title product was obtained as a colourless oil (14 mg, 50 µmol, 25%), after purification via silica gel column chromatography (7:3 n-hexane/EtOAc), followed by normal-phase preparative HPLC, with a Knauer Azura system, using a Merck LiChrosorb® Si60 7 µm, 250 × 25 mm column, and n-hexane/EtOAc 1:1 as eluent phase, in an isocratic elution.
^{1}H NMR (600 MHz, $CDCl_3$) δ 7.24 (d, J = 8.6 Hz, 2H), 6.87 (d, J = 8.7 Hz, 2H), 5.62 (s, 1H), 4.69 (q, J = 2.1 Hz, 2H), 4.18 (d, J = 5.7 Hz, 2H), 3.80 (s, 3H). **^{19}F NMR** (564 MHz, $CDCl_3$) δ −52.3 (s, 3F). **^{13}C NMR** (151 MHz, $CDCl_3$) δ 159.5, 152.8, 129.1, 128.2, 121.7 (q, J = 261.2 Hz), 115.9, 114.3, 55.4, 47.5, 29.4. **HRMS** (EI): m/z calculated for $C_{12}H_{12}O_2N_3F_3Na$: 310.0774 [M + Na]$^+$, found: 310.0776.

Pyridin-3-ylmethyl (2-oxotetrahydrofuran-3-yl)-*N*-(trifluoromethyl)carbamate (9b)

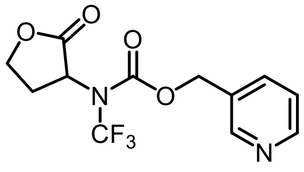

The reaction was performed on a 0.20 mmol scale, using (2-oxotetrahydrofuran-3-yl)-*N*-(trifluoromethyl)carbamoyl fluoride (**S1**) (43 mg, 0.20 mmol, 1 equiv.) and 3-pyridinemethanol (23 µL, 0.24 mmol, 1.2 equiv.), following general procedure I. The title product was obtained as a red oil (14 mg, 47 µmol, 24%), after purification via silica gel column chromatography (1:1 *n*-pentane/EtOAc) followed by normal-phase preparative HPLC, with a Knauer Azura system, using a Merck LiChrosorb® Si60 7 µm, 250 × 25 mm column, and *n*-hexane/ethanol 4:6 as eluent phase, in an isocratic elution.

^{1}H NMR (400 MHz, $CDCl_3$) δ 8.70 (s, 2H), 7.71 (d, *J* = 7.8 Hz, 1H), 7.42 – 7.29 (m, 1H), 5.28 (s, 2H), 4.73 (t, *J* = 10.3 Hz, 1H), 4.47 (td, *J* = 9.2, 2.6 Hz, 1H), 4.27 (td, *J* = 9.5, 7.4 Hz, 1H), 2.61 – 2.45 (m, 2H). **^{19}F NMR** (376 MHz, $CDCl_3$) δ –54.3 (s, 3F). **^{13}C NMR** (151 MHz, $CDCl_3$) δ 171.9, 151.6, 150.2, 149.5, 136.0, 130.4, 124.0, 120.2 (q, *J* = 263.7 Hz), 67.1, 65.4, 53.6 (q, *J* = 1.5 Hz), 26.6. **HRMS** (EI): *m/z* calculated for $C_{12}H_{11}O_4N_2F_3Na$: 327.0563 $[M + Na]^+$, found: 327.0564.

1.4 Synthesis of *N*-methyl analogues 3b–4b–5b–6b–7b–8b–9a of compounds 3c–4d–5c–6c–7c–8c–9b

1.4.1 General procedure J for the synthesis of *N*-methyl amides 3b–4b

In an oven-dried 4 mL vial, 3,4,5-trimethoxy-*N*-methylaniline **31** (47 mg, 0.24 mmol, 1 equiv.), the corresponding carboxylic acid (0.28 mmol, 1.1 equiv.) and EDC (43 mg, 0.28 mmol, 1.1 equiv.) were mixed in dry DMSO (0.3 mL). If the carboxylic acid was present as hydrochloride salt, triethylamine (TEA) (40 µL, 0.29 mmol, 1.2 equiv.) was added. The vial was sealed and the reaction was stirred at room temperature for 20 h. The solution was filtered and purified via reversed-phase HPLC, following the indicated conditions.

1.4.2 General procedure K for the synthesis of *N*-methyl ureas 5b–6b–7b–8b

In an oven-dried 4 mL vial, the corresponding amine (0.35 mmol, 1 equiv.) and triethylamine (TEA) (53 µL, 0.38 mmol, 1.1 equiv.) were mixed in dry acetonitrile (0.4 mL). Then *N*-[(4-methoxyphenyl)methyl]-*N*-methylcarbamoyl chloride **32** (74 mg, 0.35 mmol, 1 equiv.) was added in one portion. If the carboxylic acid was present as hydrochloride salt, an additional amount of triethylamine (53 µL, 0.38 mmol, 1.1 equiv.) was added. The vial was sealed, and the reaction was stirred at 60 °C for 16 h. Then, the mixture was cooled down to room temperature and the solvent was evaporated *in vacuo* at 40 °C. The obtained crude was dissolved in DMSO, filtered and purified via reversed-phase HPLC, following the indicated conditions.

1.4.3 Characterization data of compounds 3b–4b–5b–6b–7b–8b

_N_-Methyl-4-(morpholinomethyl)-_N_-(3,4,5-trimethoxyphenyl)benzamide (3b)

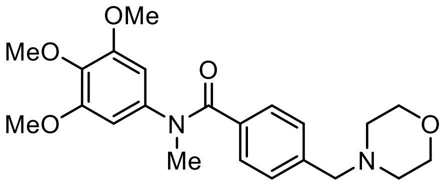

The reaction was performed on a 0.24 mmol scale, using 4-[(morpholin-4-yl)methyl]benzoic acid hydrochloride (71 mg, 0.28 mmol, 1.1 equiv.), triethylamine (45 μL, 0.33 mmol, 1.4 equiv.) in DMSO (0.3 mL), following general procedure J. The title product was obtained as brown solid (29 mg, 69 μmol, 29%), after purification via reversed-phase preparative HPLC, with a Waters XBridge C18 5мкм OBD column, and 0.1% NH_3 in water/0.1% NH_3 in methanol as eluent phase, following the reported gradient: 30 – 80%.

^{1}H NMR (400 MHz, DMSO-d_6) δ 7.25 – 7.12 (m, 4H), 6.44 (s, 2H), 3.57 (s, 6H), 3.56 (s, 3H), 3.52 (t, J = 4.5 Hz, 4H), 3.39 (s, 2H), 3.37 (s, 3H), 2.25 (t, J = 4.7 Hz, 4H). **^{13}C NMR** (101 MHz, DMSO-d_6) δ 169.5, 152.6, 140.2, 138.9, 135.7, 135.4, 128.2, 127.7, 105.0, 66.1, 61.9, 60.0, 55.9, 53.0, 37.5. **HRMS** (ESI): m/z calculated for $C_{22}H_{28}N_2O_5$ + H^+: 401.2071 $[M + H]^+$, found: 401.2069.

_N_-Methyl-_N_-(3,4,5-trimethoxyphenyl)cyclopropanecarboxamide (4b)

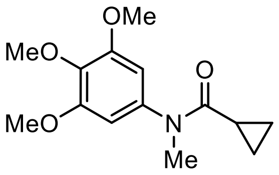

The reaction was performed on a 0.48 mmol scale, using 3,4,5-trimethoxy-_N_-methylaniline **31** (95 mg, 0.48 mmol, 1 equiv.), cyclopropanecarboxylic acid (51 μL, 0.52 mmol, 1.1 equiv.), EDC (85 mg, 0.55 mmol, 1.13 equiv.) in DMSO (0.7 mL), following general procedure J. The title product was obtained as low-melting brown solid (63 mg, 237 μmol, 50%), after purification via reversed-phase preparative HPLC, with a Waters Chromatorex C18 SMB 100-5T, and water/methanol as eluent phase, following the reported gradient: 30 – 80%.

^{1}H NMR (400 MHz, DMSO-d_6) δ 6.69 (s, 2H), 3.78 (s, 6H), 3.67 (s, 3H), 3.17 (s, 3H), 1.44 (s, 1H), 0.81 – 0.74 (m, 2H), 0.64 (dq, J = 10.2, 3.3 Hz, 2H). **^{13}C NMR** (101 MHz, DMSO-d_6) δ 172.1, 153.2, 139.6, 136.4, 105.0, 60.0, 56.1, 36.9, 12.3, 7.9. **HRMS** (ESI): m/z calculated for $C_{14}H_{19}NO_4$ + H^+: 266.1387 $[M + H]^+$, found: 266.1383.

3-Hydroxy-_N_-(4-methoxybenzyl)-_N_-methylazetidine-1-carboxamide (5b)

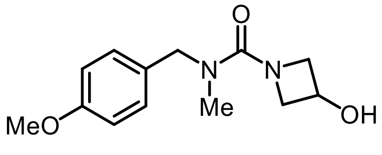

The reaction was performed on a 0.35 mmol scale, using azetidin-3-ol hydrochloride (38 mg, 0.35 mmol, 1 equiv.), triethylamine (0.11 mL, 0.79 mmol, 2.2 equiv.) in acetonitrile (0.4 mL). following general procedure K. The title product was obtained as colourless solid (62 mg, 0.24 mmol, 70%), after purification via reversed-phase preparative HPLC, with a Waters Chromatorex C18 SMB 100-5T column, and water/methanol as eluent phase, following the reported gradient: 30 – 80%.

^{1}H NMR (400 MHz, DMSO-d_6) δ 7.19 – 7.11 (m, 2H), 6.93 – 6.85 (m, 2H), 5.53 (d, J = 6.2 Hz, 1H), 4.41 – 4.30 (m, 1H), 4.27 (s, 2H), 4.07 – 3.99 (m, 2H), 3.73 (s, 3H), 3.65 (ddd, J = 8.3, 4.9, 1.0 Hz, 2H), 2.64 (s, 3H). **^{13}C NMR** (101 MHz, DMSO-d_6) δ 162.4, 158.3, 130.1, 128.6, 113.8, 60.7, 59.9, 55.0, 51.1, 34.3. **HRMS** (ESI): m/z calculated for $C_{13}H_{18}N_2O_3$: 251.1390 [M + H]+, found: 251.1396.

3-Hydroxy-*N*-(4-methoxybenzyl)-*N*-methylpiperidine-1-carboxamide (6b)

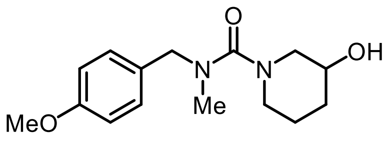

The reaction was performed on a 0.43 mmol scale, using piperidin-3-ol (43 mg, 0.43 mmol, 1 equiv.), *N*-[(4-methoxyphenyl)methyl]-*N*-methylcarbamoyl chloride **32** (91 mg, 0.43 mmol, 1 equiv.), triethylamine (63 µL, 0.46 mmol, 1.1 equiv.) in acetonitrile (0.5 mL), following general procedure K. The title product was obtained as colorless oil (75 mg, 0.27 mmol, 63%), after purification via reversed-phase preparative HPLC, with a Waters Chromatorex C18 SMB 100-5T column, and water/methanol as eluent phase, following the reported gradient: 30 – 80%.

^{1}H NMR (400 MHz, DMSO-d_6) δ 7.22 – 7.13 (m, 2H), 6.94 – 6.84 (m, 2H), 4.81 (d, *J* = 4.0 Hz, 1H), 4.22 (d, *J* = 2.5 Hz, 2H), 3.73 (s, 3H), 3.57 – 3.41 (m, 2H), 3.29 (t, *J* = 4.4 Hz, 1H), 2.75 (ddd, *J* = 13.1, 10.2, 2.8 Hz, 1H), 2.61 (s, 3H), 2.57 (dd, *J* = 10.9, 2.6 Hz, 1H), 1.82 (dt, *J* = 12.0, 4.7 Hz, 1H), 1.65 (dp, *J* = 12.1, 4.0 Hz, 1H), 1.46 – 1.19 (m, 2H). **^{13}C NMR** (101 MHz, DMSO-d_6) δ 163.8, 158.3, 129.9, 128.9, 113.8, 113.8, 65.1, 55.0, 53.8, 52.4, 46.5, 35.8, 33.1, 22.8. **HRMS** (ESI): *m/z* calculated for $C_{15}H_{22}N_2O_3 + H^+$: 279.1703 [M + H]$^+$, found: 279.1700.

***N*-(4-Methoxybenzyl)-*N*,4-dimethyl-3-oxopiperazine-1-carboxamide (7b)**

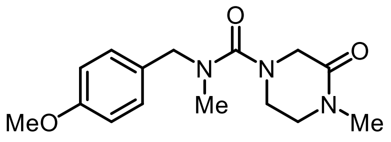

The reaction was performed on a 0.30 mmol scale, using 1-methylpiperazin-2-one (34 mg, 0.30 mmol, 1 equiv.), *N*-[(4-methoxyphenyl)methyl]-*N*-methylcarbamoyl chloride **32** (64 mg, 0.30 mmol, 1 equiv.), triethylamine (44 µL, 0.32 mmol, 1.1 equiv.) in acetonitrile (0.3 mL), following general procedure K. The title product was obtained as light yellow solid (57 mg, 0.20 mmol, 66%), after purification via reversed-phase preparative HPLC, with a Waters Chromatorex C18 SMB 100-5T column, and water/methanol as eluent phase, following the reported gradient: 40 – 90%.

^{1}H NMR (400 MHz, DMSO-d_6) δ 7.24 – 7.09 (m, 2H), 6.96 – 6.84 (m, 2H), 4.27 (s, 2H), 3.73 – 3.72 (m, 5H), 3.42 (t, *J* = 5.4 Hz, 2H), 2.84 (s, 3H), 2.67 (s, 3H). **^{13}C NMR** (101 MHz, DMSO-d_6) δ 165.2, 162.7, 158.4, 129.5, 128.9, 128.9, 113.9, 55.0, 52.2, 50.2, 47.3, 43.4, 35.5, 33.5. **HRMS** (ESI): *m/z* calculated for $C_{15}H_{21}N_3O_3 + H^+$: 292.1656 [M + H]$^+$, found: 292.1653.

3-(Cyanomethyl)-1-(4-methoxybenzyl)-1-methylurea (8b)

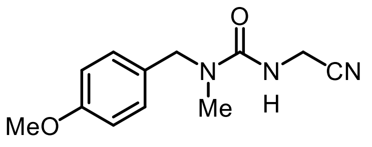

The reaction was performed on a 0.46 mmol scale, using 2-aminoacetonitrile hydrochloride (43 mg, 0.46 mmol, 1 equiv.), *N*-[(4-methoxyphenyl)methyl]-*N*-methylcarbamoyl chloride **32** (97 mg, 0.46 mmol, 1 equiv.), triethylamine (0.104 g, 1.03 mmol, 2.3 equiv.) in acetonitrile 0.5 mL, following general procedure K. The title product was obtained as colourless solid (75 mg, 0.32 mmol, 70%), after purification via reversed-phase preparative HPLC, with a Waters Chromatorex C18 SMB 100-5T column, and water/methanol as eluent phase, following the reported gradient: 20 – 70%.

^{1}H NMR (400 MHz, DMSO-d_6) δ 7.22 – 7.06 (m, 3H), 6.94 – 6.82 (m, 2H), 4.35 (s, 2H), 4.04 (d, *J* = 5.5 Hz, 2H), 3.73 (s, 3H), 2.72 (s, 3H). **^{13}C NMR** (101 MHz, DMSO-d_6) δ 158.4, 157.3, 130.0, 128.7, 118.8, 113.9,

55.0, 50.4, 33.4, 29.2. **HRMS** (ESI): *m/z* calculated for $C_{12}H_{15}N_3O_2$ + H^+: 234.1237 $[M + H]^+$, found: 234.1235.

1.4.4 Synthesis and characterization data of *N*-Me analogue 9a of compound 9b

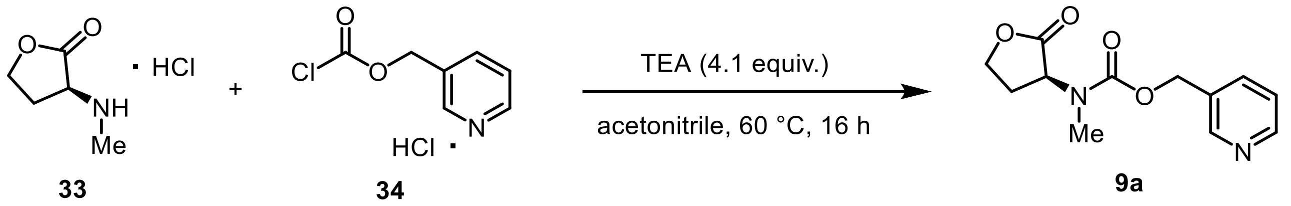

(3S)-3-(Methylamino)oxolan-2-one hydrochloride (63 mg, 0.42 mmol, 1 equiv.) and triethylamine (0.18 mL, 1.3 mmol, 1.3 equiv.) were mixed in dry acetonitrile (1 mL), and (pyridin-3-yl)methyl carbonochloridate hydrochloride (86 mg, 0.42 mmol, 1 equiv.) was added in one portion. The reaction mixture was sealed and heated for 16 h at 60 °C. Then, the mixture was cooled down to room temperature, and the solvent was evaporated *in vacuo*. The obtained crude was dissolved in DMSO, the solution was filtered and purified via reversed-phase preparative HPLC, with a Waters Chromatorex C18 SMB 100-5T column, and water/(acetonitrile:water 95:5 vol%) as eluent phase, following the gradient 0 – 25%, delivering pyridin-3-ylmethyl (*S*)-methyl(2-oxotetrahydrofuran-3-yl)carbamate **9a** (8.3 mg, 33 µmol, 8%).

^{1}H NMR (400 MHz, DMSO-d_6) δ 8.59 (d, J = 8.0 Hz, 1H), 8.54 (d, J = 4.8 Hz, 1H), 7.79 (dd, J = 15.4, 7.8 Hz, 1H), 7.42 (dd, J = 7.9, 4.6 Hz, 1H), 5.15 (s, 2H), 4.88 (dt, J = 28.9, 10.2 Hz, 1H), 4.41 – 4.14 (m, 2H), 2.84 (d, J = 15.5 Hz, 3H), 2.37 (ddd, J = 13.2, 6.5, 3.3 Hz, 2H). **^{13}C NMR** (101 MHz, DMSO-d_6) δ 174.1, 155.3, 154.9, 149.2, 149.0, 148.9, 135.7, 135.5, 132.2, 123.6, 65.3, 64.5, 56.6, 56.0, 32.8, 25.6, 25.0. **HRMS** (ESI): *m/z* calculated for $C_{12}H_{14}N_2O_4$ + H^+: 251.1027 $[M + H]^+$, found: 251.1025.

1.5 Synthesis of *N*-methyl analogues S14–S15 of compound series 10a–d

1.5.1 General procedure L for the synthesis of compounds S14–S15

In a heat gun-dried 100 mL flask, the corresponding amide (0.15 mmol, 1 equiv.) was dissolved in dioxane (19 mL). The solution was cooled down to 0 °C and stirred for 15 minutes, potassium bis(trimethylsilyl)amide 1 M in THF (0.37 mL, 0.37 mmol, 2.2 equiv.) was added dropwise and the mixture was stirred for another 15 minutes at 0 °C. Subsequently, iodomethane (0.14 mL, 2.3 mmol, 15 equiv.) was added dropwise and, after 15 minutes stirring at 0 °C, the mixture was warmed up to room temperature. The reaction was monitored via LC-MS. After 22 h, the mixture was quenched with acetic acid (0.02 mL) and the solvent was removed *in vacuo* at 43 °C. The resulting crude was purified via reversed-phase HPLC, following the indicated conditions.

1.5.2 Characterization data of compounds S14–S15

(*S*)-*N*-Methyl-*N*-((2-oxo-3-(4-(3-oxomorpholino)phenyl)oxazolidin-5-yl)methyl)-2-phenylacetamide (S14)

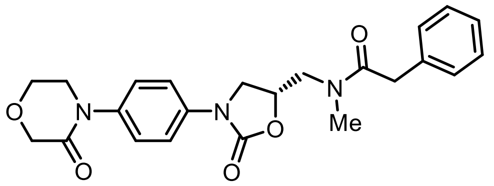

The reaction was performed in a 50 mL flask, on a 0.15 mmol scale, using (*S*)-*N*-((2-oxo-3-(4-(3-oxomorpholino)phenyl)-oxazolidin-5-yl)methyl)-2-phenylacetamide (**S12**) (61 mg, 0.15 mmol, 1 equiv.), following general procedure L. The title product (19 mg, 45 μmol, 30%) was obtained after purification with a Waters XBridge C18 5μm ODB 19 × 150 mm column and water/acentonitrile in 0.01 M NH_4HCO_3 (pH 9) as eluent phase, following the reported gradient: 5 – 95%.

^{1}H NMR (500 MHz, DMSO-d_6) δ 7.61 – 7.52 (m, 2H), 7.46 – 7.38 (m, 2H), 7.33 – 7.15 (m, 5H), 4.96 – 4.81 (m, 1H), 4.20 (s, 2H), 4.14 (td, J = 8.9, 6.0 Hz, 1H), 3.97 (dd, J = 6.0, 4.1 Hz, 2H), 3.82 – 3.63 (m, 7H), 3.11 (s, 2H), 2.92 (s, 1H). **^{13}C NMR** (126 MHz, DMSO-d_6) δ 171.4, 170.7, 166.0, 154.0, 153.9, 137.1, 137.1, 136.5, 136.0, 135.5, 129.3, 129.0, 128.2, 128.2, 126.3, 125.9, 125.9, 118.4, 118.3, 71.7, 71.3, 67.7, 63.5, 52.3, 50.1, 49.0, 47.4, 47.3, 37.1, 33.9. **HRMS** (ESI): *m/z* calculated for $C_{23}H_{25}N_3O_5$ + H^+: 424.1872 [M + H] $^+$, found: 424.1874.

(*S*)-*N*-Methyl-*N*-((2-oxo-3-(4-(3-oxomorpholino)phenyl)oxazolidin-5-yl)methyl)benzamide (S15)

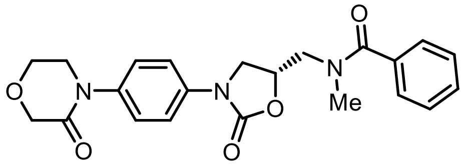

The reaction was performed in a 50 mL flask, on a 0.13 mmol scale, using (*S*)-*N*-((2-oxo-3-(4-(3-oxomorpholino)phenyl)-oxazolidin-5-yl)methyl)benzamide (**S13**) (51 mg, 0.13 mmol, 1 equiv.), potassium bis(trimethylsilyl)amide 1 M in THF (0.32 mL, 0.32 mmol, 2.5 equiv.), iodomethane (0.12 mL, 1.9 mmol, 15 equiv.) in dioxane (16 mL), and quenched with acetic acid (0.02 mL), following general procedure L. The title product (12 mg, 29 μmol, 23%) was obtained, after purification with a Waters XBridge C18 5μm ODB 19 × 150 mm column and water/acentonitrile in 0.01 M NH_4HCO_3 (pH 9) as eluent phase, following the reported gradient: 5 – 95%.

^{1}H NMR (500 MHz, CD_3CN) δ 7.61 (s, 2H), 7.48 – 7.27 (m, 7H), 4.98 (s, 1H), 4.19 (s, 3H), 4.00 – 3.95 (m, 2H), 3.94 – 3.83 (m, 2H), 3.70 (t, J = 5.1 Hz, 3H), 3.03 (s, 3H). **^{13}C NMR** (151 MHz, CD_3CN) δ 172.9, 167.6, 155.6, 138.6, 138.2, 137.6, 130.5, 129.5, 129.4, 128.1, 127.8, 127.3, 119.6, 119.4, 72.9, 71.6, 69.0, 64.8, 54.9, 51.3, 50.5, 49.1, 48.6, 40.0. **HRMS** (ESI): *m/z* calculated for $C_{22}H_{23}N_3O_5$ + H^+: 410.1710 [M + H] $^+$, found: 410.1701.

1.6 Synthesis of *N*-Me analogues S17–S18 of compound series 12a–d

1.6.1 General procedure M for the synthesis of compounds S17–S18

2-(2-Methyl-5-nitro-1*H*-imidazol-1-yl)ethan-1-ol (**21**) (50 mg, 0.29 mmol, 1 equiv.) and bis(trichloromethyl) carbonate (BTC) (45 mg, 0.15 mmol 0.5 equiv.) were solubilized in dry dichloromethane (2.5 mL). Subsequently, the solution was cooled down to 0 °C and DIPEA (0.21 mL, 1.2 mmol, 4.1 equiv.) was added dropwise. The mixture was stirred at 0 °C for 2 h, after which a THF solution (0.2 mL) of the corresponding amine HCl (0.58 mmol, 2 equiv.) was slowly added. The reaction was warmed up to room temperature and monitored via LCMS analysis. After 1.5 h, the solvent mixture was removed *in vacuo* at 43 °C, to deliver an oily crude. This crude was solubilized in DMSO (0.7 mL), filtered via 0.45 µm filter and purified via the indicated conditions.

1.6.2 Characterization data of *N*-Me and *N*-^{i}Pr analogues of compound series 12a–d

2-(2-Methyl-5-nitro-1*H*-imidazol-1-yl)ethyl benzyl(methyl)carbamate (S17)

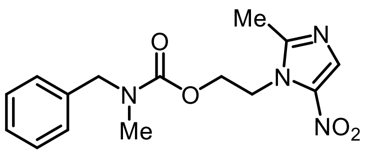

The reaction was carried out on a 0.29 mmol scale, using *N*-methyl-1-phenylmethanamine (70 µL, 0.58 mmol, 2 equiv.), following general procedure M. The title compound was obtained as colourless oil (7.0 mg, 22 µmol, 8%), after purification via reversed-phase preparative HPLC with a Waters Sunfire C18 5µm ODB 19 × 150 mm column and water/acentonitrile in 0.015% difluoroacetic acid (pH 3) as eluent phase, following the reported gradient: 3 – 95%.

^{1}H NMR (600 MHz, DMSO-*d*$_6$) δ 8.09 – 7.97 (m, 1H), 7.33 (t, *J* = 7.4 Hz, 2H), 7.26 (t, *J* = 7.3 Hz, 1H), 7.20 – 7.04 (m, 2H), 4.60 (dt, *J* = 16.7, 5.1 Hz, 2H), 4.46 – 4.29 (m, 4H), 2.79 – 2.62 (m, 3H), 2.44 – 2.28 (m, 3H). **^{13}C NMR** (151 MHz, DMSO-*d*$_6$) δ 155.8, 155.5, 151.8, 138.9, 138.7, 137.6, 137.4, 133.4, 128.9, 128.8, 127.7, 127.6, 127.2, 63.6, 63.3, 52.0, 51.5, 45.5, 45.3, 34.4, 33.6, 14.0. **HRMS** (ESI): *m/z* calculated for $C_{15}H_{18}N_4O_4 + H^+$: 319.1406 [M + H]$^+$, found 319.1422.

2-(2-Methyl-5-nitro-1*H*-imidazol-1-yl)ethyl methyl(phenyl)carbamate (S18)

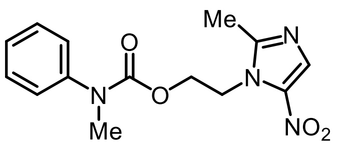

The reaction was carried out on a 0.29 mmol scale, using *N*-methylaniline (0.32 mL, 2.9 mmol, 10 equiv.), following general procedure M. The title compound was obtained as colourless oil (6.7 mg, 22 µmol, 8%), after purification via reversed-phase preparative HPLC with a Waters XBridge C18 5µm ODB 19 × 150 mm column and water/acetonitrile in 0.01 M NH_4HCO_3 (pH 9) as eluent phase, following the reported gradient 2 – 94%.

^{1}H NMR (600 MHz, DMSO-*d*$_6$) δ 8.02 (s, 1H), 7.38 – 7.31 (m, 2H), 7.26 – 7.21 (m, 1H), 7.18 (d, *J* = 7.8 Hz, 2H), 4.63 – 4.45 (m, 2H), 4.36 (t, *J* = 4.9 Hz, 2H), 3.13 (s, 3H), 2.43 – 2.10 (s, 3H). **^{13}C NMR** (151 MHz, DMSO-*d*$_6$) δ 154.2, 151.6, 142.7, 138.4, 133.2, 129.0, 126.2, 125.8, 63.6, 44.9, 37.5, 13.8, 13.5. **HRMS** (ESI): *m/z* calculated for $C_{14}H_{16}N_4O_4 + H^+$: 305.1250 [M + H]$^+$, found 305.1230.

1.7 Synthesis of *N*-Me analogues S19–S20–S21–S22 of compound series 13a–d

1.7.1 <u>General procedure N</u> for the synthesis of compound series S19–S20–S21

5-Chloro-3-(2-(4-(2-hydroxyethyl)piperazin-1-yl)-2-oxoethyl)benzo[*d*]thiazol-2(3*H*)-one (**24**) (050 mg, 0.14 mmol, 1 equiv.) was solubilized in dichloromethane (2.5 mL). The mixture was cooled down to 0 °C and 4–nitrophenyl chloroformate (34 mg, 0.17 mmol, 1.2 equiv.) was added in one portion. Subsequently, DIPEA (30 µL, 0.17 mmol, 1.2 equiv.) was added dropwise and the mixture was stirred for 1.5 h at 0 °C. The reaction was monitored via LC-MS and, after full conversion of compound **24** into the activated carbamate, the corresponding amine (neat or as hydrochloride salt) was added (0.28 mmol, 2 equiv.). The mixture was warmed up to room temperature and additionally stirred for 2 h. Then, the reaction was quenched with MeOH (0.5 mL) and the solvent was removed *in vacuo* at 43 °C. The resulting oily crude was solubilized in 0.6 mL DMSO, filtered via 0.45 µm filter and purified via reversed-phase HPLC, following the indicated conditions.

1.7.2 Characterization data of compound series S19–S20–S21

2-(4-(2-(5-Chloro-2-oxobenzo[*d*]thiazol-3(2*H*)-yl)acetyl)piperazin-1-yl)ethyl dimethylcarbamate (S19)

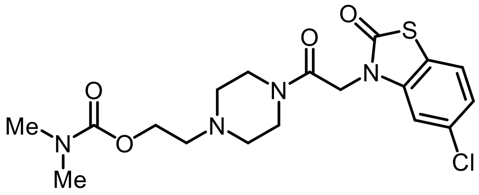

The reaction was carried out on 0.11 mmol scale, using 5-chloro-3-(2-(4-(2-hydroxyethyl)piperazin-1-yl)-2-oxoethyl)-benzo[*d*]thiazol-2(3*H*)-one (**24**) (40 mg, 0.11 mmol, 1 equiv.), DIPEA (0.02 mL, 0.13 mmol, 1.2 equiv.) and dimethylamine hydrochloride (17 mg, 0.22 mmol, 2 equiv.), following <u>general procedure N</u>. The title compound was obtained (25 mg, 59 µmol, 54%), after purification via reversed-phase HPLC with a Waters XBridge C18 5µm ODB 19 × 150 mm column and water/acentonitrile in 0.01 M NH_4HCO_3 (pH 9) as eluent phase, following the reported gradient: 5 – 95%.

^{1}H NMR (600 MHz, DMSO-*d*$_6$) δ 7.68 (d, *J* = 8.4 Hz, 1H), 7.44 (d, *J* = 2.0 Hz, 1H), 7.24 (dd, *J* = 8.3, 2.0 Hz, 1H), 4.92 (s, 2H), 4.10 (t, *J* = 5.8 Hz, 2H), 3.56 – 3.51 (m, 2H), 3.43 (d, *J* = 4.9 Hz, 2H), 2.83 (d, *J* = 14.2 Hz, 6H), 2.61 (t, *J* = 5.8 Hz, 2H), 2.55 (t, *J* = 5.1 Hz, 2H), 2.43 (t, *J* = 5.1 Hz, 2H). **^{13}C NMR** (151 MHz, DMSO-*d*$_6$) δ 169.5, 163.7, 155.8, 138.8, 131.4, 124.3, 123.0, 120.0, 111.8, 62.4, 56.4, 52.8, 52.5, 44.3, 43.9, 41.8, 36.1, 35.6. **HRMS** (ESI): *m/z* calculated for $C_{18}H_{23}ClN_4O_4S + H^+$: 427.1201 [M + H]$^+$, found 427.1199.

2-(4-(2-(5-Chloro-2-oxobenzo[*d*]thiazol-3(2*H*)-yl)acetyl)piperazin-1-yl)ethyl cyclopropyl(methyl)carbamate (S20)

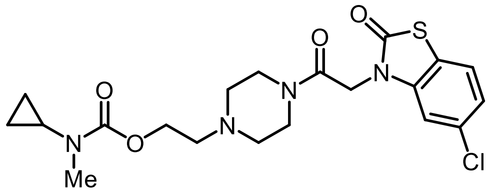

The reaction was carried out on 0.14 mmol scale, using *N*-methylcyclopropan-2-amine HCl (30 mg, 0.28 mmol, 2 equiv.), following <u>general procedure N</u>. The title compound was obtained (32 mg, 71 µmol, 51%), after purification via reversed-

phase HPLC with a Waters XBridge C18 5μm ODB 19 × 150 mm column and water/acentonitrile in 0.01 M NH_4HCO_3 (pH 9) as eluent phase, following the reported gradient: 5 – 95%.
^{1}H NMR (600 MHz, DMSO-*d*$_6$) *δ* 7.69 (d, *J* = 8.4 Hz, 1H), 7.45 (d, *J* = 2.0 Hz, 1H), 7.24 (dd, *J* = 8.4, 2.0 Hz, 1H), 4.93 (s, 2H), 4.12 (t, *J* = 5.7 Hz, 2H), 3.57 – 3.51 (m, 2H), 3.43 (dd, *J* = 6.2, 4.0 Hz, 2H), 2.79 (s, 3H), 2.61 (t, *J* = 5.7 Hz, 2H), 2.56 (dq, *J* = 6.9, 3.5 Hz, 3H), 2.44 (t, *J* = 5.1 Hz, 2H), 0.80 – 0.44 (m, 4H). **^{13}C NMR** (151 MHz, DMSO-*d*$_6$) *δ* 169.6, 163.8, 156.9, 138.9, 131.4, 124.3, 123.1, 120.0, 111.8, 62.5, 56.4, 52.9, 52.6, 44.4, 43.9, 41.8, 34.7, 30.2, 7.5. **HRMS** (ESI): *m/z* calculated for $C_{20}H_{25}ClN_4O_4S + H^+$: 453.1358 $[M + H]^+$, found 453.1379.

2-(4-(2-(5-Chloro-2-oxobenzo[*d*]thiazol-3(2*H*)-yl)acetyl)piperazin-1-yl)ethyl benzyl(methyl)carbamate (*S*21)

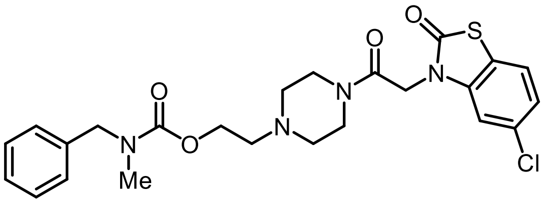

The reaction was carried out on 0.14 mmol scale, using *N*-methyl-1-phenylmethanamine (36 μL, 0.28 mmol, 2 equiv.), following general procedure N. The title compound was obtained (47 mg, 93 μmol, 66%), after purification via reversed-phase HPLC with a Waters Sunfire C18 5μm ODB 19 × 150 mm column and water/acentonitrile in 0.015% difluoroacetic acid (pH 3) as eluent phase, following the reported gradient: 3 – 95%.
^{1}H NMR (600 MHz, DMSO-*d*$_6$) *δ* 7.70 (d, *J* = 8.3 Hz, 1H), 7.45 (d, *J* = 1.9 Hz, 1H), 7.36 (t, *J* = 7.4 Hz, 2H), 7.31 – 7.20 (m, 4H), 4.98 – 4.88 (m, 2H), 4.44 (s, 2H), 4.19 (s, 2H), 3.61 – 3.37 (m, 4H), 2.82 (s, 3H), 2.63 – 2.38 (m, 6H). **^{13}C NMR** (151 MHz, DMSO-*d*$_6$) *δ* 169.4, 163.7, 156.0, 155.6, 138.8, 137.9, 137.7, 131.3, 128.6, 127.4, 127.3, 127.2, 124.3, 123.0, 120.0, 111.8, 62.3, 56.4, 52.7, 52.4, 51.7, 51.5, 44.2, 43.8, 41.6, 34.3, 33.7. **HRMS** (ESI): *m/z* calculated for $C_{24}H_{27}ClN_4O_4S + H^+$: 503.1514 $[M + H]^+$, found 503.1534.

1.7.3 Synthesis and characterization data of 2-(4-(2-(5-chloro-2-oxobenzo[*d*]thiazol-3(2*H*)-yl)acetyl)piperazin-1-yl)ethyl methyl(phenyl)carbamate (S22)

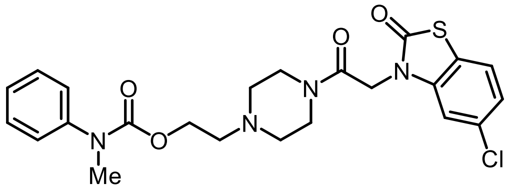

5-Chloro-3-(2-(4-(2–hydroxyethyl) piperazin-1-yl)-2-oxoethyl)benzo[*d*]thiazol-2(3*H*)-one (**24**) (50 mg, 0.14 mmol, 1 equiv.) was solubilized in THF (2 mL), the mixture was cooled down to 0 °C and 4-nitrophenyl chloroformate (34 mg, 0.17 mmol, 1.2 equiv.) was added in one portion. Subsequently, DIPEA (30 μL, 0.17 mmol, 1.2 equiv.) was added dropwise at 0 °C and the mixture was stirred at 0 °C for 1.5 h. The reaction was monitored via LC–MS and after full conversion of compound **24** into the activated carbamate, *N*-methylaniline (0.15 mL, 1.4 mmol, 10 equiv.) was added dropwise at 0 °C. The mixture was warmed up to 70 °C and stirred for 2 h. Then, the reaction was quenched with MeOH (0.5 mL) and the solvent was removed *in vacuo* at 43 °C. The resulting oily crude was solubilized in 0.6 mL DMSO, filtered via 0.45 μm filter and purified via reversed-phase HPLC with a Waters XBridge C18 5μm ODB 19 × 150 mm column and water/acentonitrile in 0.01 M NH_4HCO_3 (pH 9) as eluent phase, following the reported gradient 5 – 95%,

delivering 2-(4-(2-(5-chloro-2-oxobenzo[*d*]thiazol-3(2*H*)-yl)acetyl)piperazin-1-yl)ethylmethyl-(phenyl)carbamate (8.8 mg, 18 µmol, 13%).

^{1}H NMR (600 MHz, DMSO-d_6) δ 7.70 (d, J = 8.4 Hz, 1H), 7.45 (d, J = 2.0 Hz, 1H), 7.40 – 7.35 (m, 2H), 7.35 – 7.31 (m, 2H), 7.25 (dd, J = 8.3, 2.0 Hz, 1H), 7.22 (tt, J = 6.9, 1.3 Hz, 1H), 4.92 (s, 2H), 4.15 (t, J = 5.6 Hz, 2H), 3.51 (t, J = 5.1 Hz, 2H), 3.39 (t, J = 5.1 Hz, 2H), 3.23 (s, 3H), 2.57 (t, J = 5.7 Hz, 2H), 2.49 – 2.45 (m, 2H), 2.35 (t, J = 5.1 Hz, 2H). **^{13}C NMR** (151 MHz, DMSO-d_6) δ 169.4, 163.6, 154.7, 143.1, 138.8, 131.3, 128.7, 125.9, 125.8, 124.3, 122.9, 119.9, 111.8, 62.8, 56.1, 52.7, 52.4, 44.3, 43.8, 41.7, 37.4. **HRMS** (ESI): *m/z* calculated for $C_{23}H_{25}ClN_4O_4S + H^+$: 489.1358 [M + H]$^+$, found 489.1364.

1.8 Synthesis of *N*-Me analogues S23–S24–S25–S26 of compound series 14a–d

1.8.1 General procedure O for the synthesis of compounds S23–S24

Acetaminophen (**26**) (50 mg, 0.33 mmol, 1 equiv.) was solubilized in dichloromethane (0.5 mL), the mixture was cooled down to 0 °C, and bis(trichloromethyl) carbonate (BTC) (53 mg, 0.17 mmol, 0.5 equiv) was added as dichloromethane solution (0.5 mL). Subsequently, DIPEA (0.32 mL, 1.3 mmol, 4 equiv.) was added dropwise and the reaction was stirred at 0 °C. After 1.5 h, the mixture was transferred dropwise into a heat gun-dried flask, containing a solution of NaH 60% in mineral oil (16 mg, 0.66 mmol, 2 equiv.) in dichloromethane (1.0 mL) and the corresponding amine (0.66 mmol, 2 equiv.), previously stirred at 0 °C for 1 h. Then, the reaction mixture was warmed up to room temperature and additionally stirred for 1 h, after which the solvent was removed *in vacuo* at 43 °C. The resulting crude was solubilized in 0.6 mL DMSO, filtered via 0.45 µm filter and purified via the indicated conditions.

1.8.2 General procedure P for the synthesis of compounds S25–S26

Acetaminophen (**26**) (50 mg, 0.33 mmol, 1 equiv.) was solubilized in dichloromethane (0.5 mL), the mixture was cooled down to 0 °C, and bis(trichloromethyl) carbonate (BTC) (53 mg, 0.17 mmol, 0.5 equiv) was added as dichloromethane solution (0.5 mL). Subsequently, DIPEA (0.32 mL, 1.3 mmol, 4 equiv.) was added dropwise and the reaction was stirred at 0 °C. After 1.5 h, the corresponding amine (0.66 mmol, 2 equiv.) was added dropwise at 0 °C, the mixture was warmed up to room temperature and additionally stirred for 3 h. Subsequently, the solvent was removed *in vacuo* at 43 °C, the resulting crude was solubilized in 0.6 mL DMSO, filtered via 0.45 µm filter and purified via the indicated conditions.

1.8.3 Characterization data of compounds S23–S24–S25–S26

4-Acetamidophenyl dimethylcarbamate (S23)

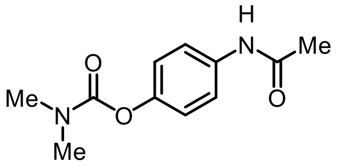

The reaction was carried out on 0.33 mmol scale, using dimethylamine, 2 M in THF solution (0.33 mL, 0.66 mmol, 2 equiv.), following general procedure O. The title compound was obtained (14 mg, 63 µmol, 19%) after purification via reversed-phase HPLC with a Waters Sunfire C18 5µm ODB 19

× 150 mm column and water/acentonitrile in 0.015% difluoroacetic acid (pH 3) as eluent phase, following the reported gradient: 3 – 95%.

^{1}H NMR (600 MHz, DMSO-d_6) δ 9.95 (s, 1H), 7.59 – 7.50 (m, 2H), 7.08 – 6.95 (m, 2H), 3.02 (s, 3H), 2.89 (s, 3H), 2.03 (s, 3H). **^{13}C NMR** (151 MHz, DMSO-d_6) δ 168.2, 154.2, 146.5, 136.4, 122.0, 119.6, 36.3, 36.1, 23.9. **HRMS** (ESI): *m/z* calculated for $C_{11}H_{14}N_2O_3$ + H^+: 223.1082 $[M + H]^+$, found 223.1066.

4-Acetamidophenyl cyclopropyl(methyl)carbamate (S24)

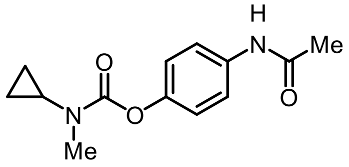

The reaction was carried out on 0.33 mmol scale, using *N*-methylcyclopropan-2-amine HCl (70 mg, 0.66 mmol, 2 equiv.), following general procedure O. The title compound was obtained (16 mg, 64 µmol, 19%), after purification via reversed-phase HPLC with a Waters XBridge C18 5µm ODB 19 × 150 mm column and water/acentonitrile in 0.01 M NH_4HCO_3 (pH 9) as eluent phase, following the reported gradient: 5 – 95%.

^{1}H NMR (600 MHz, DMSO-d_6) δ 9.96 (s, 1H), 7.56 (d, J = 8.3 Hz, 2H), 7.02 (d, J = 8.4 Hz, 2H), 2.89 (s, 3H), 2.73 (m, 1H), 2.03 (s, 3H), 0.75 (m, 4H). **^{13}C NMR** (151 MHz, DMSO-d_6) δ 168.2, 155.4, 146.5, 136.4, 122.0, 119.6, 34.9, 30.2, 23.9, 7.7. **HRMS** (ESI): *m/z* calculated for $C_{13}H_{16}N_2O_3$ + H^+: 249.1239 $[M + H]^+$, found 249.1232.

4-Acetamidophenyl benzyl(methyl)carbamate (S25)

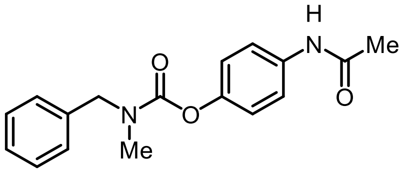

The reaction was carried out on 0.33 mmol scale, using *N*-methyl-1-phenylmethanamine (90 µL, 0.66 mmol, 2 equiv.), following general procedure P. The title compound was obtained (21 mg, 70 µmol, 21%) after purification via reversed-phase HPLC with a Waters XBridge C18 5µm ODB 19 × 150 mm column and water/acentonitrile in 0.01 M NH_4HCO_3 (pH 9) as eluent phase, following the reported gradient: 5 – 95%.

^{1}H NMR (600 MHz, DMSO-d_6) δ 9.96 (s, 1H), 7.57 (t, J = 8.5 Hz, 2H), 7.39 (dt, J = 14.4, 7.4 Hz, 2H), 7.35 – 7.31 (m, 2H), 7.30 (s, 1H), 7.13 – 6.96 (m, 2H), 4.73 – 4.36 (m, 2H), 3.04 – 2.84 (m, 3H), 2.04 (s, 3H). **^{13}C NMR** (151 MHz, DMSO-d_6) δ 168.2, 154.6, 154.1, 146.5, 137.5, 137.3, 136.5, 128.7, 128.6, 127.5, 127.4, 127.2, 122.0, 121.9, 119.7, 52.0, 34.6, 34.2, 23.9. **HRMS** (ESI): *m/z* calculated for $C_{17}H_{18}N_2O_3$ + H^+: 299.1396 $[M + H]^+$, found 299.1385.

4-Acetamidophenyl methyl(phenyl)carbamate (S26)

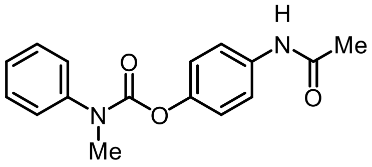

The reaction was carried out on 0.33 mmol scale, stirring for 3 h after the addition of *N*–methylaniline (70 µL, 0.66 mmol, 2 equiv.), following general procedure P. The title compound was obtained (18 mg, 63 µmol, 19%) after purification via reversed-phase HPLC with a Waters Sunfire C18 5µm ODB 19 × 150 mm column and water/acentonitrile in 0.015% difluoroacetic acid (pH 3) as eluent phase, following the reported gradient: 3 – 95%.

^{1}H NMR (600 MHz, DMSO-d_6) δ 9.96 (s, 1H), 7.55 (d, J = 8.7 Hz, 2H), 7.45 (dd, J = 8.4, 1.5 Hz, 2H), 7.41 (dd, J = 8.6, 6.9 Hz, 2H), 7.27 (tt, J = 7.2, 1.5 Hz, 1H), 7.05 (d, J = 8.4 Hz, 2H), 3.33 (s, 3H), 2.03 (s, 3H). **^{13}C NMR** (151 MHz, DMSO-d_6) δ 168.5, 153.5, 146.4, 143.0, 136.7, 129.1, 126.6, 126.1, 122.1, 119.9, 38.1, 24.0. **HRMS** (ESI): *m/z* calculated for $C_{16}H_{16}N_2O_3$ + H^+: 285.1239 [M + H]$^+$, found 285.1256.

1.9 Synthesis of *N*-H analogues 3a–8a

1.9.1 General procedure Q for the synthesis of 3a–4a

In an oven-dried 4 mL vial, 3,4,5-trimethoxyaniline **35** (46 mg, 0.25 mmol, 1 equiv.), the corresponding carboxylic acid (0.28 mmol, 1.1 equiv.) and EDC (43 mg, 0.28 mmol, 1.1 equiv.) were mixed in dry DMSO (0.3 mL). If the carboxylic acid was present as hydrochloride salt, triethylamine (TEA) (40 µL, 0.29 mmol, 1.2 equiv.) was added. The vial was sealed and the reaction was stirred at room temperature for 20 h. The solution was filtered and purified via reversed-phase HPLC, following the indicated conditions.

1.9.2 General procedure R for the synthesis of 5a–6a–7a–8a

In an oven-dried 4 mL vial, the corresponding amine (0.42 mmol, 1 equiv.) and 1-(isocyanatomethyl)-4-methoxybenzene **36** (66 mg, 0.41 mmol, 1.1 equiv.) were mixed in dry acetonitrile (0.6 mL). If the amine was present as hydrochloride salt, triethylamine (TEA) (68 µL, 0.49 mmol, 1.1 equiv.) was added. The vial was sealed and the reaction was stirred at 100 °C for 16 h. Then, the mixture was cooled down to room temperature and the solvent was evaporated *in vacuo* at 40 °C. The obtained crude was dissolved in DMSO, filtered and purified via reversed-phase HPLC, following the indicated conditions.

1.9.3 Characterization data of compounds 3a–4a–5a–6a–7a–8a

4-(Morpholinomethyl)-*N*-(3,4,5-trimethoxyphenyl)benzamide (3a)

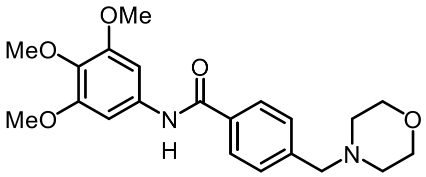

The reaction was carried out on 0.25 mmol, using 4-[(morpholin-4-yl)methyl]benzoic acid hydrochloride (62 mg, 0.28 mmol, 1.1 equiv.), in DMSO (0.3 mL), following general procedure Q. The title product was obtained as colourless solid (65 mg, 0.17 mmol, 65%) after purification via reversed-phase HPLC, with a Waters XBridge Prep C18 5мкм OBD column, and 0.1% NH_3 in water/0.1% NH_3 in methanol as eluent phase, following the reported gradient: 30 – 80%.

^{1}H NMR (400 MHz, DMSO-d_6) δ 10.10 (s, 1H), 7.92 – 7.90 (m, 2H), 7.46 (d, J = 8.0 Hz, 2H), 7.23 (s, 2H), 3.77 (s, 6H), 3.64 (s, 3H), 3.59 (t, J = 4.6 Hz, 4H), 3.54 (s, 2H), 2.38 – 2.36 (m, 4H). **^{13}C NMR** (101 MHz, DMSO-d_6) δ 165.2, 152.6, 141.7, 135.4, 133.7, 133.7, 128.8, 127.5, 98.0, 66.2, 62.0, 60.1, 55.7, 53.2. **HRMS** (ESI): *m/z* calculated for $C_{21}H_{26}N_2O_5$ + H^+: 387.1915 [M + H]$^+$, found: 387.1911.

N-(3,4,5-Trimethoxyphenyl)cyclopropanecarboxamide (4a)

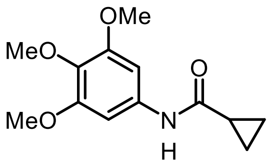

The reaction was performed on a 0.40 mmol scale, using 3,4,5-trimethoxyaniline **35** (74 mg, 0.40 mmol, 1 equiv.), cyclopropanecarboxylic acid (39 mg, 0.45 mmol, 1.1 equiv.), EDC (78 mg, 0.50 mmol, 1.2 equiv.) in DMSO (0.5 mL), following general procedure Q. The title product was obtained as colourless solid (61 mg, 0.24 mmol, 59%), after purification via reversed-phase HPLC, with a Waters Chromatorex C18 SMB 100-5T column, and water/methanol as eluent phase, following the reported gradient: 30 – 80%.

^{1}H NMR (400 MHz, DMSO-d_6) δ 10.11 (s, 1H), 6.98 (s, 2H), 3.72 (s, 6H), 3.60 (s, 3H), 1.73 (tt, J = 7.4, 5.1 Hz, 1H), 0.77 (dd, J = 6.7, 4.3 Hz, 4H). **^{13}C NMR** (101 MHz, DMSO-d_6) δ 171.5, 152.7, 135.6, 133.1, 96.6, 60.1, 55.6, 14.5, 7.0. **HRMS** (ESI): *m/z* calculated for $C_{13}H_{17}NO_4 + H^+$: 252.1231 $[M + H]^+$, found: 252.1227.

3-Hydroxy-N-(4-methoxybenzyl)azetidine-1-carboxamide (5a)

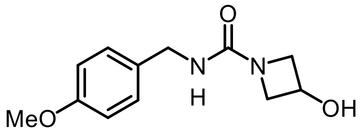

The reaction was carried out on a 0.41 mmol scale, using azetidin-3-ol hydrochloride (46 mg, 0.42 mmol, 1.1 equiv.), following general procedure R. The title product was obtained as light yellow solid (52 mg, 0.22 mmol, 54%), after purification via reversed-phase HPLC with Waters Chromatorex C18 SMB 100-5T column, and water/methanol as eluent phase, following the reported gradient: 30 – 80%.

^{1}H NMR (400 MHz, DMSO-d_6) δ 7.20 – 7.12 (m, 2H), 6.89 – 6.82 (m, 2H), 6.74 (t, J = 6.1 Hz, 1H), 5.53 (d, J = 6.5 Hz, 1H), 4.36 (qt, J = 6.6, 4.7 Hz, 1H), 4.10 (d, J = 6.0 Hz, 2H), 3.99 – 3.91 (m, 2H), 3.72 (s, 3H), 3.55 (ddd, J = 8.4, 4.7, 0.9 Hz, 2H). **^{13}C NMR** (101 MHz, DMSO-d_6) δ 159.7, 158.0, 132.9, 128.3, 113.5, 59.9, 59.0, 55.0, 42.3. **HRMS** (ESI): *m/z* calculated for $C_{12}H_{16}N_2O_3 + H^+$: 237.1234 $[M + H]^+$, found: 237.1230.

3-Hydroxy-N-(4-methoxybenzyl)piperidine-1-carboxamide (6a)

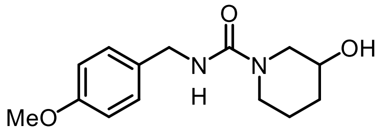

The reaction was carried out on 0.37 mmol scale, using 1-(isocyanatomethyl)-4-methoxybenzene **36** (60 mg, 0.37 mmol, 1 equiv.), piperidin-3-ol (39 mg, 0.39 mmol, 1.1 equiv.) in acetonitrile (0.6 mL), following general procedure R. The title compound was obtained as a colorless oil (60 mg, 0.22 mmol, 59%), after purification via reversed-phase HPLC with Waters Chromatorex C18 SMB 100-5T column, and water/methanol as eluent phase, following the reported gradient: 30 – 80%.

^{1}H NMR (400 MHz, DMSO-d_6) δ 7.20 – 7.11 (m, 2H), 6.90 (t, J = 5.9 Hz, 1H), 6.87 – 6.81 (m, 2H), 4.76 (d, J = 4.6 Hz, 1H), 4.13 (d, J = 5.7 Hz, 2H), 3.86 (dt, J = 12.5, 2.8 Hz, 1H), 3.71 (s, 3H), 3.67 (dt, J = 13.4, 3.0 Hz, 1H), 3.36 (dd, J = 9.1, 4.7 Hz, 1H), 2.71 (tt, J = 10.5, 3.1 Hz, 1H), 2.53 (d, J = 9.1 Hz, 1H), 1.89 – 1.77 (m, 1H), 1.60 (dq, J = 13.4, 3.6 Hz, 1H), 1.36 – 1.17 (m, 2H). **^{13}C NMR** (101 MHz, DMSO-d_6) δ 157.9, 157.3, 133.2, 128.3, 113.5, 65.2, 55.0, 50.9, 43.5, 42.9, 33.4, 23.2. **HRMS** (ESI): *m/z* calculated for $C_{14}H_{20}N_2O_3 + H^+$: 265.1547 $[M + H]^+$, found: 265.1542.

N-(4-Methoxybenzyl)-4-methyl-3-oxopiperazine-1-carboxamide (7a)

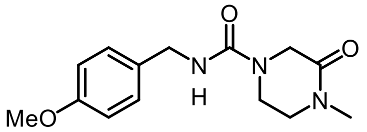

The reaction was carried out on a 386 µmol scale, using 1-methylpiperazin-2-one (44 mg, 0.39 mmol, 1 equiv.), 1-(isocyanatomethyl)-4-methoxybenzene **36** (64 mg, 0.39 mmol, 1 equiv.) in acetonitrile (0.4 mL), following general procedure R. The title compound was obtained as a colorless oil (51 mg, 0.18 mmol, 47%), after purification via reversed-phase HPLC with Waters Chromatorex C18 SMB 100-5T column, and water/methanol as eluent phase, following the reported gradient: 30 – 80%.

^{1}H NMR (400 MHz, DMSO-d_6) δ 7.20 – 7.15 (m, 2H), 7.13 (t, J = 5.8 Hz, 1H), 6.89 – 6.82 (m, 2H), 4.16 (d, J = 5.8 Hz, 2H), 3.91 (s, 2H), 3.72 (s, 3H), 3.59 – 3.53 (m, 2H), 3.29 (t, J = 5.5 Hz, 2H), 2.84 (s, 3H). **^{13}C NMR** (101 MHz, DMSO-d_6) δ 165.1, 158.0, 156.6, 132.7, 128.4, 113.5, 55.0, 47.7, 47.6, 42.9, 33.6. **HRMS** (ESI): *m/z* calculated for $C_{14}H_{19}N_3O_3$ + H^+: 278.1499 $[M + H]^+$, found: 278.1498.

1-(Cyanomethyl)-3-(4-methoxybenzyl)urea (8a)

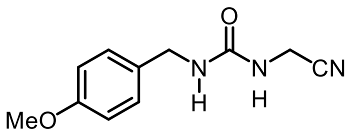

The reaction was carried out on a 429 µmol scale, using 1-(isocyanatomethyl)-4-methoxybenzene **36** (70 mg, 0.43 mmol, 1 equiv.), 2-aminoacetonitrile hydrochloride (40 mg, 0.44 mmol, 1 equiv.), triethylamine (51 mg, 0.50 mmol, 1.2 equiv.) in acetonitrile (0.7 mL), following general procedure R. The title compound was obtained as brown solid (27 mg, 0.12 mmol, 29%), after purification via reversed-phase HPLC with Waters Chromatorex C18 SMB 100-5T column, and water/methanol as eluent phase, following the reported gradient: 20 – 70%.

^{1}H NMR (400 MHz, DMSO-d_6) δ 7.20 – 7.13 (m, 2H), 6.90 – 6.84 (m, 2H), 6.76 (t, J = 6.0 Hz, 1H), 6.49 (t, J = 6.0 Hz, 1H), 4.14 (d, J = 5.8 Hz, 2H), 4.04 (d, J = 5.9 Hz, 2H), 3.72 (s, 3H). **^{13}C NMR** (101 MHz, DMSO-d_6) δ 158.2, 157.3, 132.2, 128.4, 118.8, 113.7, 55.1, 42.5, 28.4. **HRMS** (ESI): *m/z* calculated for $C_{11}H_{13}N_3O_2$ + H^+: 220.1081 $[M + H]^+$, found: 220.1079.

1.10 Miscellaneous

1.10.1 General procedure S for the synthesis of S12–S13

(*S*)-4-(4-(5-(Aminomethyl)-2-oxooxazolidin-3-yl)phenyl)morpholin-3-one hydrochloride (**17**) (0.20 g, 0.61 mmol, 1 equiv.) was suspended in dichloromethane (1.7 mL) and triethylamine (0.11 mL, 0.79 mmol, 1.3 equiv.) was added. The solution was cooled down to 0 °C and stirred for 5 min. In a parallel heat gun-dried flask, the corresponding acyl chloride (0.61 mmol, 1 equiv.) was dissolved in dichloromethane (2 mL), and added dropwise at 0 °C. After stirring for 15 min, the mixture was warmed up to room temperature and monitored via LC-MS analysis. The mixture was filtered and the solvent was removed *in vacuo* at 43 °C, and the resulting oily crude was purified via reversed-phase chromatography, following the indicated conditions.

1.10.2 Characterization data of compounds S12–S13

(*S*)-*N*-((2-Oxo-3-(4-(3-oxomorpholino)phenyl)oxazolidin-5-yl)methyl)-2-phenylacetamide (S12)

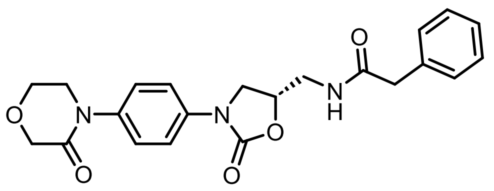

The reaction was performed on a 0.61 mmol scale and stirred for 5 h, using 2-phenylacetyl chloride (80 µL, 0.61 mmol, 1 equiv.), following general procedure S. The title product was obtained (60 mg, 0.15 mmol, 24%), after purification with Biotage® Sfär C18 D 30 g column and 0.1 % formic acid in water/acetonitrile as eluent phase, following the reported gradient: 10 – 10% 3 column volumes (CV); 10 – 60% 15 CV, 60 – 100% 3 CV, 100 – 100% 3 CV.

^{1}H NMR (500 MHz, DMSO-d_6) δ 8.54 – 8.47 (m, 1H), 7.56 – 7.50 (m, 2H), 7.43 – 7.37 (m, 2H), 4.76 (dtd, J = 9.5, 5.8, 3.9 Hz, 1H), 4.20 (s, 2H), 4.10 (t, J = 9.0 Hz, 1H), 4.00 – 3.95 (m, 2H), 3.75 – 3.66 (m, 3H), 3.55 – 3.38 (m, 4H). **^{13}C NMR** (126 MHz, DMSO-d_6) δ 171.1, 166.0, 154.1, 137.0, 136.5, 136.2, 128.8, 128.2, 126.3, 126.0, 118.2, 71.6, 67.7, 63.5, 49.1, 47.0, 42.2, 41.3. **HRMS** (ESI): *m/z* calculated for $C_{22}H_{23}N_3O_5$ + H^+: 410.1716 [M + H]$^+$, found: 410.1740.

(*S*)-*N*-((2-Oxo-3-(4-(3-oxomorpholino)phenyl)oxazolidin-5-yl)methyl)benzamide (S13)

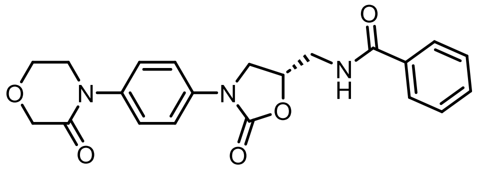

The reaction was performed on a 0.61 mmol scale and stirred for 5 h, using benzoyl chloride (70 µL, 0.61 mmol, 1 equiv.) following general procedure S. The title product was obtained (0.11 g, 0.28 mmol, 46%), after purification with Biotage® Sfär C18 D 30 g column and 0.1 % formic acid in water/acetonitrile as eluent phase, following the reported gradient: 5 – 5% 3 column volumes (CV); 5 – 45% 10 CV; 45 – 100% 3 CV, 100 – 100% 3 CV.

^{1}H NMR (500 MHz, DMSO-d_6) δ 8.82 (t, J = 5.8 Hz, 1H), 7.87 – 7.82 (m, 2H), 7.60 – 7.51 (m, 3H), 7.50 – 7.37 (m, 4H), 4.87 (dq, J = 8.7, 5.6 Hz, 1H), 4.20 (d, J = 5.5 Hz, 3H), 4.00 – 3.93 (m, 2H), 3.91 (dd, J = 9.1, 5.9 Hz, 1H), 3.74 – 3.69 (m, 2H), 3.68 – 3.58 (m, 2H). **^{13}C NMR** (126 MHz, DMSO-d_6) δ 167.0, 165.9, 154.2, 137.0, 136.5, 134.0, 131.4, 128.3, 127.3, 125.9, 118.3, 71.3, 67.7, 63.5, 49.0, 47.5, 42.3. **HRMS** (ESI): *m/z* calculated for $C_{21}H_{21}N_3O_5$ + H^+: 396.1559 [M + H]$^+$, found: 396.1596.

2. Biological assays

2.1 Metabolic profiling for compounds 3c, 4c, 6c, 7c, and 9b

2.1.1 Metabolite Identification

The metabolic profiles of five analogs **3c** (G03789402), **4c** (G03789401), **6c** (G03789407), **7c** (G03789405), and **9b** (G03789410) were characterized following incubation in human hepatocytes. The primary metabolites are summarized in Table S1. **3c** (G03789402) underwent amide hydrolysis, yielding metabolite M1. **7c** (G03789405) predominantly formed an *O*-dealkylated/glucuronide conjugate

(M1). **6c** (G03789407) generated an *O*-dealkylated/glucuronide conjugate (M1) and an oxidized product (M2). **9b** (G03789410) produced a hydrolyzed metabolite (M1) and an oxidized metabolite (M2). No metabolites were detected for **4c** (G03789401, human hep CL_{int} = 334 µL/min/mg) under the same conditions.

Analogs bearing an anisole or aryl *O*-alkyl motif (G03789405 and G03789407) consistently underwent *O*-dealkylation followed by glucuronidation, indicating this substituent as a metabolic hotspot within this series. Notably, only G03789402, which contains an *N*-trimethoxyphenyl amide linked to benzylmorpholine, displayed measurable amide hydrolysis, whereas the corresponding *N*-CF_3 carbamate and urea derivatives remained stable with respect to amide cleavage under identical conditions. These findings demonstrate that modifying the substituents around the *N*-CF_3-amide region effectively reduces susceptibility to amide hydrolysis.

Table S1. Structures of compounds and their metabolites identified in human hepatocytes.

Compound	Biotransformation	Formula	$[M+H]^+$	Mass Shift (Δ)	hu heps CL_{int} [µL/min/mg]
**3c** (G03789402) 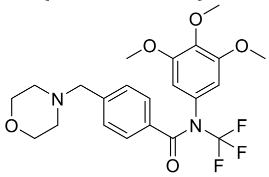	Parent	$C_{22}H_{25}F_3N_2O_5$	455.1788		151
M1 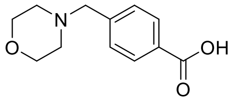	Amide hydrolysis	$C_{12}H_{15}NO_3$	222.1125	−233	
**6c** (G03789407) 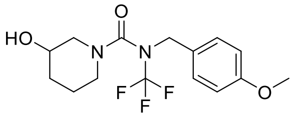	Parent	$C_{15}H_{19}F_3N_2O_3$	333.1421		103
M1 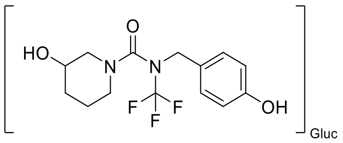	*O*-Dealkylation and glucuronidation	$C_{20}H_{25}F_3N_2O_9$	495.1585	+162	
M2 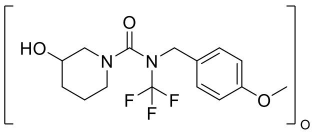	Oxidation	$C_{15}H_{19}F_3N_2O_4$	348.1297	+16	
**7c** (G03789405) 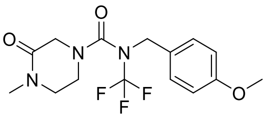	Parent	$C_{15}H_{18}F_3N_3O_3$	346.1373		23

M1 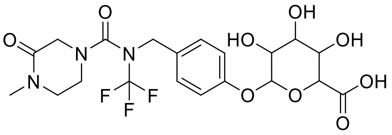	*O*-Dealkylation and glucuronidation	$C_{20}H_{24}F_3N_3O_9$	508.1537	+162	
**9b** (G03789410) 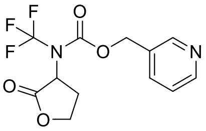	Parent	$C_{12}H_{11}F_3N_2O_4$	305.0744		22
M1 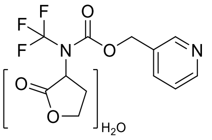	Hydrolysis	$C_{12}H_{13}F_3N_2O_5$	323.0849	+18	
M2 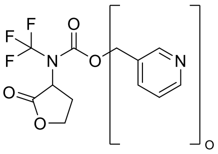	Oxidation	$C_{12}H_{11}F_3N_2O_5$	321.0693	+16	

2.1.2 Procedures

Metabolite identification assay conditions

As previously described (Cai *et al.*),[3] hepatocyte incubations were carried out at a compound concentration of 1 μM and a cell density of 0.5×10^6 cells mL^{-1} at 37 °C in an atmosphere of 5% CO_2. Time points were collected at 0, 60, 120, 180, and 240 minutes. Hepatocytes were prepared by thawing in INVITROGRO HT medium and subsequently diluted to 1×10^6 cells mL^{-1} in DMEM buffer. Compound dilution, incubation, and liquid-handling procedures were automated using a Tecan Fluent liquid-handling platform. Quenched samples at designated time intervals were then processed for LC–HRMS analysis.

HRMS data acquisition and processing

High-resolution mass spectrometric (HRMS) data were acquired using a Shimadzu Nexera X2 ultra-performance liquid chromatography (UPLC) system coupled to a Thermo Fisher Orbitrap Exploris 480 mass spectrometer equipped with a Heated Electrospray Ionization (HESI) source. Separation was achieved on a Kinetex C18 column (2.6 μm, 100 Å, 100 × 2.1 mm). LC–MS conditions were identical to those previously described by Cai *et al.*[3] The flow rate was set at 0.4 mL min^{-1}, starting with 5% solvent B (0.1% formic acid in acetonitrile) and 95% solvent A (0.1% formic acid in water) for 0.5 min. Solvent B was ramped to 75% at 3 min, increased to 95% by 3.8 min and held until 4.3 min, then returned to 5% by 4.5 min and maintained until 5.3 min. MS acquisition was performed in positive ion mode with a spray voltage of 3.2 kV, sheath gas at 60 psi, and ion transfer tube temperature of 320 °C. Data were collected using full-scan data-dependent MS^2 ($ddMS^2$) over m/z 100–1000 at 15,000 resolution for full scans. MS^2 spectra were triggered under stepped normalized collision energies of 25, 45, and 60 eV, acquired at 7,500 resolution within a 0.8 s cycle time.

Metabolite identification profiling

LC-MS raw data were processed using MassMetaSite (version 4.7) and analyzed in ONIRO for metabolite identification and relative quantification (Zamora *et al.*).[4] Data acquired from chromatography method (LC gradient for the metabolite identification assay) was processed by MassMetaSite and uploaded into ONIRO. Within ONIRO, the proposed metabolite structures were refined or confirmed through additional manual analysis of the corresponding MS2 data.

2.2 Stability of *N*-methyl analogs in aqueous media and human plasma

Table S2. Stability of *N*-methyl analogs in aqueous media[a] and human plasma.[b]

		● =	compound ID	half-life at 25 °C in aqueous media[a] [days] pH 1.0	pH 7.4	pH 10.0	amount of parent compound left after 18 h in human plasma at 37 °C[b] [%]
amides	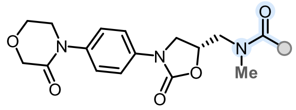	Me	Me analog of **10a**	17 (3)	>60	15 (0.2)	100
		^{c}Pr	Me analog of **10b**	>60	>60	19 (0.4)	98 (3)
		Bn	Me analog of **10c**	24 (6)	>60	20 (1)	100
		Ph	Me analog of **10d**	37 (10)	>60	17 (1)	100
	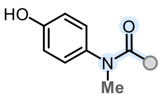	Me	Me analog of **11a**	>60	>60	>60	100
		^{c}Pr	Me analog of **11b**	>60	>60	>60	100
		Bn	Me analog of **11c**	>60	>60	>60	98 (3)
		Ph	Me analog of **11d**	>60	>60	>60	99 (1)
carbamates	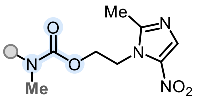	Me	Me analog of **12a**	>60	>60	>60	100
		^{c}Pr	Me analog of **12b**	>60	>60	>60	96 (8)
		Bn	Me analog of **12c**	>60	>60	>60	95 (4)
		Ph	Me analog of **12d**	>60	>60	>60	96 (2)
	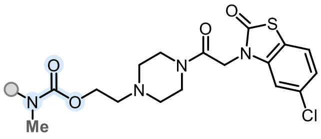	Me	Me analog of **13a**	>60	>60	32 (2)	98 (6)
		^{c}Pr	Me analog of **13b**	>60	>60	>60	100
		Bn	Me analog of **13c**	>60	>60	>60	98 (4)
		Ph	Me analog of **13d**	>60	>60	>60	100
	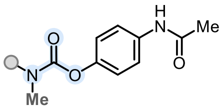	Me	Me analog of **14a**	12 (5)	>60	>60	97 (4)
		^{c}Pr	Me analog of **14b**	7.9 (2.8)	>60	>60	98 (2)
		Bn	Me analog of **14c**	15 (3)	>60	>60	99 (1)
		Ph	Me analog of **14d**	9.1 (0.2)	>60	>60	99 (1)

[a]Half-life (in days) at 25 °C in 0.1 M HCl solution (pH 1.0), 20 mM sodium phosphate buffer (pH 7.4), or 20 mM sodium carbonate buffer (pH 10.0), given as the arithmetic mean of at least two independent measurements with standard deviation in brackets. [b]Amount of compound left after 18 h in human plasma at 37 °C (in %) given as the arithmetic mean of at least three independent measurements with standard deviation in brackets.

2.3 Determination of degradation products after incubation of *N*-trifluoromethyl analogs 10–16 at pH 1.0, 7.4, and 10.0 for 24 h at 70 °C

After incubating the aqueous mixtures at 70 °C for 24 h, the solutions were allowed to cool to room temperature and analysed using 6 different methods (see Table S3).

Table S3. Explored gradients to determine gradient resulting in best resolution of the different degradation products.

**Method A**	Solvent A	1 mM ammonium formate and 10 mM formic acid, pH 3
	Solvent B	95% MeCN in milliQ water
	Gradient	0 – 0.2 min: 3% B<br>0.2 – 1.3 min 3 to 50% B<br>1.3 – 1.8 min 50 to 99% B<br>1.8 – 2.0 min 3% B
**Method B**	Solvent A	47 mM ammonia and 6.5 mM ammonium carbonate, pH 10
	Solvent B	95% MeCN in milliQ water
	Gradient	0 – 0.2 min: 3%B<br>0.2 – 1.3 min 3 to 50% B<br>1.3 – 1.8 min 50 to 99% B<br>1.8 – 2.0 min 3% B
**Method C**	Solvent A	1 mM ammonium formate and 10 mM formic acid, pH 3
	Solvent B	95% MeCN in milliQ water
	Gradient	0 – 0.2 min: 10%B<br>0.2 – 3.7 min 10 to 99% B<br>3.7 – 3.8 min 99% B<br>3.8 – 4.0 min 10% B
**Method D**	Solvent A	47 mM ammonia and 6.5 mM ammonium carbonate, pH 10
	Solvent B	95% MeCN in milliQ water
	Gradient	0 – 0.2 min: 10%B<br>0.2 – 3.7 min 10 to 99% B<br>3.7 – 3.8 min 99% B<br>3.8 – 4.0 min 10% B
**Method E**	Solvent A	1 mM ammonium formate and 10 mM formic acid, pH 3
	Solvent B	95% MeCN in milliQ water
	Gradient	0 – 0.2 min: 10%B<br>0.2 – 1.3 min 10 to 99% B<br>1.3 – 1.8 min 99% B<br>1.8 – 2.0 min 10% B
**Method F**	Solvent A	47 mM ammonia and 6.5 mM ammonium carbonate, pH 10
	Solvent B	95% MeCN in milliQ water
	Gradient	0 – 0.2 min: 10%B<br>0.2 – 1.3 min 10 to 99% B<br>1.3 – 1.8 min 99% B<br>1.8 – 2.0 min 10% B

The gradient resulting in the best separation of the different degradation products is given in Table S4 and the corresponding chromatograms in Figures S1–3.

Table S4. Method resulting in best resolution of the different degradation products.

		● =	compound ID	Method with best separation of degradation products		
				pH 1.0	pH 7.4	pH 10.0
amides	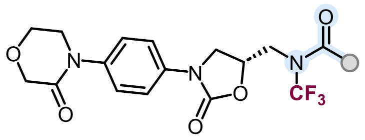	Me	**10a**	C	C	C
		^{c}Pr	**10b**	B	B	B
		Bn	**10c**	C	C	C
		Ph	**10d**	A	A	A
	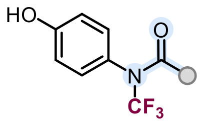	Me	**11a**	A	F	A
		^{c}Pr	**11b**	F	F	F
		Bn	**11c**	D	F	B
		Ph	**11d**	F	F	F
carbamates	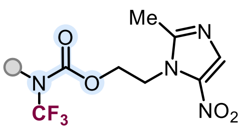	Me	**12a**	B	B	B
		^{c}Pr	**12b**	A	A	A
		Bn	**12c**	B	B	B
		Ph	**12d**	D	D	D
	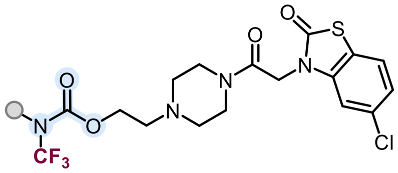	Me	**13a**	D	D	D
		^{c}Pr	**13b**	F	F	F
		Bn	**13c**	B	B	B
		Ph	**13d**	D	D	D
	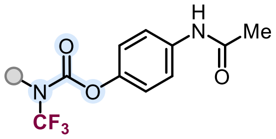	Me	**14a**	B	B	B
		^{c}Pr	**14b**	B	B	B
		Bn	**14c**	B	B	B
		Ph	**14d**	F	F	F
ureas	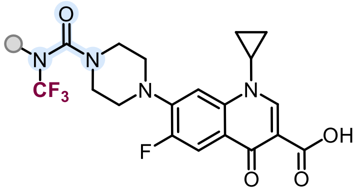	Me	**15a**	F	F	F
		^{c}Pr	**15b**	C	C	C
		Bn	**15c**	A	A	A
		Ph	**15d**	D	D	D
	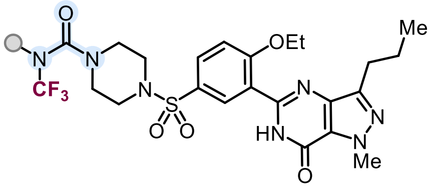	Me	**16a**	A	A	A
		^{c}Pr	**16b**	F	F	F
		Bn	**16c**	E	E	E
		Ph	**16d**	D	D	D

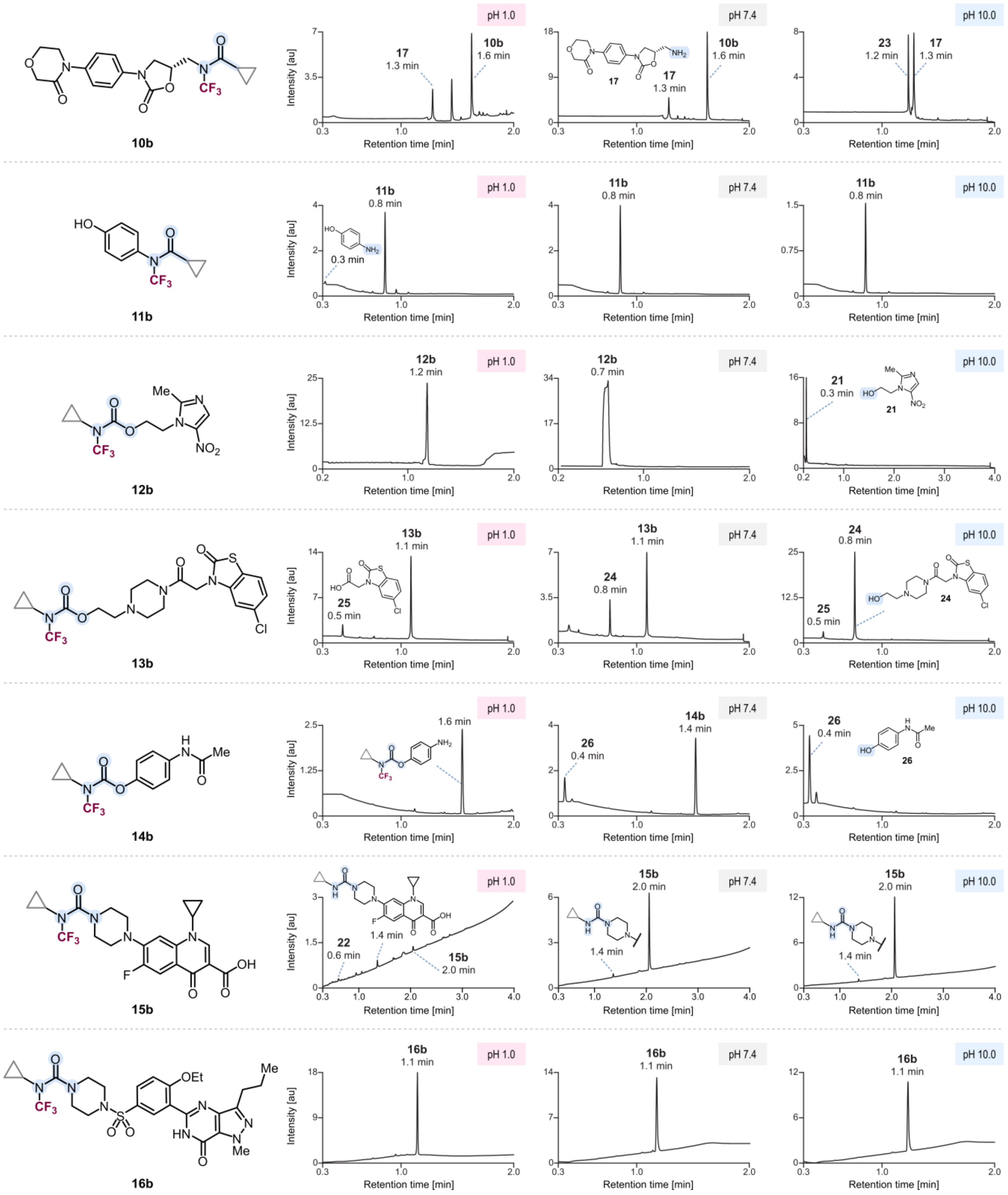

Figure S1. Chromatograms after incubation of *N*-CF_3 cyclopropyl analogs **10b–16b** in 0.1 M HCl solution (pH 1.0), 20 mM sodium phosphate buffer (pH 7.4), and 20 mM sodium carbonate buffer (pH 10.0) at 70 °C for 24 h. Decomposition products were identified based on their MS trace.

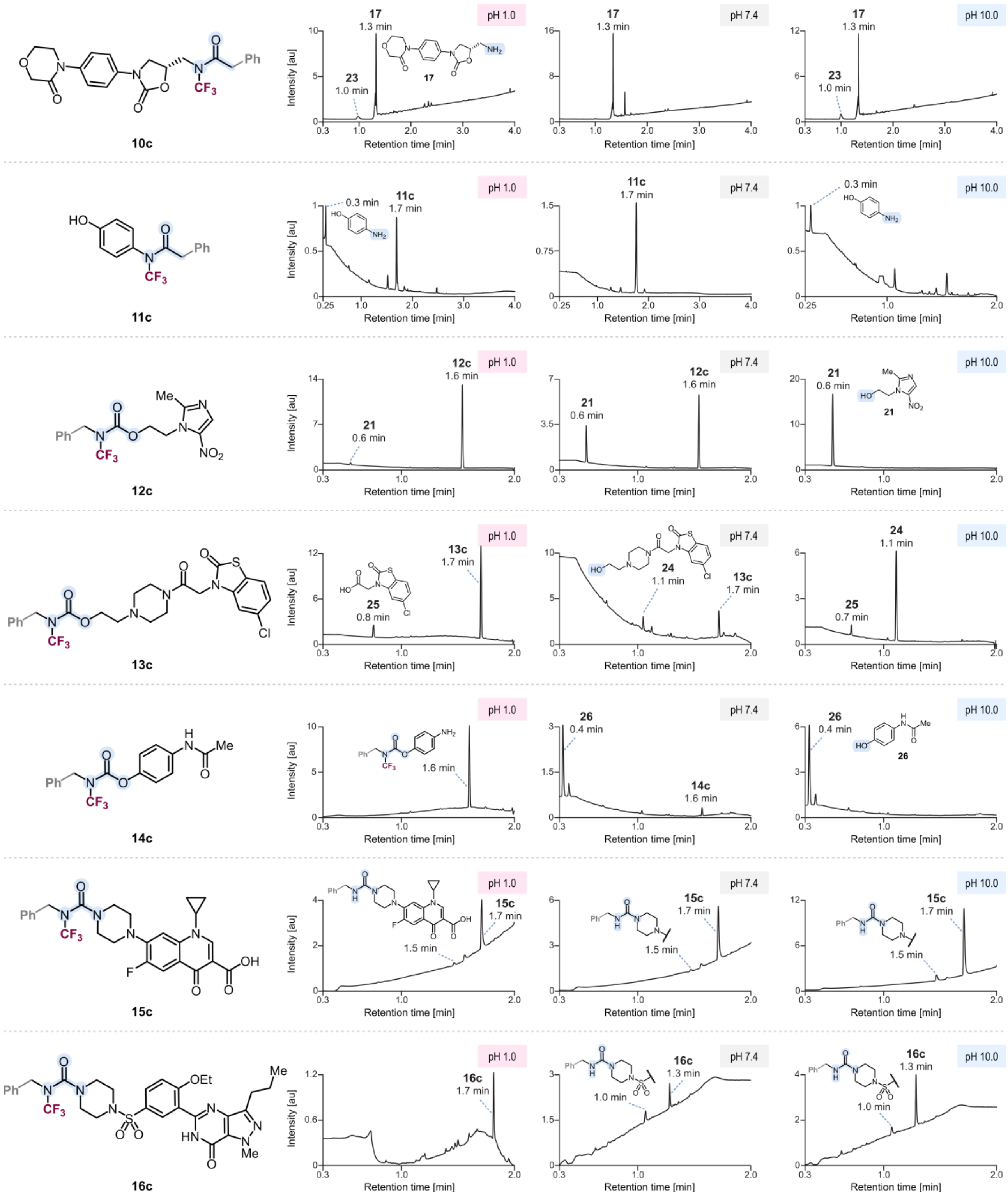

Figure S2. Chromatograms after incubation of *N*-CF_3 benzyl analogs **10c**–**16c** in 0.1 M HCl solution (pH 1.0), 20 mM sodium phosphate buffer (pH 7.4), and 20 mM sodium carbonate buffer (pH 10.0) at 70 °C for 24 h. Decomposition products were identified based on their MS trace.

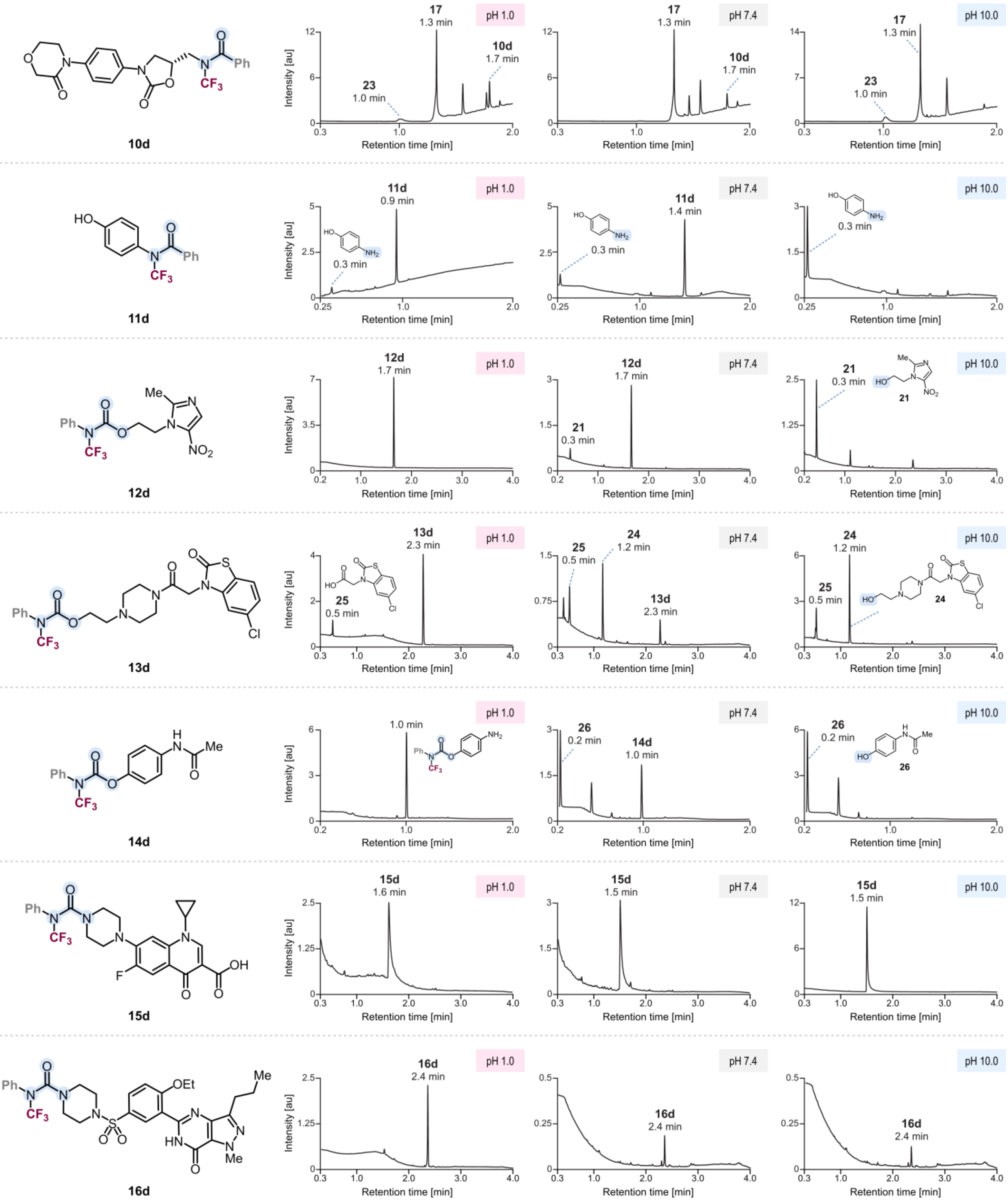

Figure S3. Chromatograms after incubation of *N*-CF_3 phenyl analogs **10d–16d** in 0.1 M HCl solution (pH 1.0), 20 mM sodium phosphate buffer (pH 7.4), and 20 mM sodium carbonate buffer (pH 10.0) at 70 °C for 24 h. Decomposition products were identified based on their MS trace.

2.4 *In vivo* rat PK of *N*-trifluoromethyl compounds 15a and 15b and their *N*-methyl (15e, 15g) and *N*-isopropyl analogs (15f, 15h)

Table S5. Detailed results of *in vivo* rat PK study.

		Animal	CL [mL/min/kg]	V_{ss} [L/kg]	V_z [L/kg]	eff $t_{1/2}$[a] [h]	**F [%]**	C_{max} [µmol/L]	t_{max} [h]	AUC [h*µmol/L]
**15a**	iv	1	13.2	1.3	1.8	1.6	-	1.37	-	1.36
		2	5.8	0.89	2.1	2.6	-	2.41	-	3.15
		Mean	9.5	1.1	1.95	1.9	-	1.89	-	2.26
	oral	3	-	-	-	-	160	1.43	1	7.25
		4	-	-	-	-	98	1.05	1	4.40
		Mean	-	-	-	-	**129**	1.24	1	5.83
**15e**	iv	1	17	1.5	2.2	1.5	-	1.44	-	1.19
		2	13.2	1.5	4.7	1.9	-	1.89	-	1.56
		Mean	15.1	1.5	3.5	1.7	-	1.67	-	1.38
	oral	3	-	-	-	-	119	0.38	1	1.65
		4	-	-	-	-	81	0.31	1	1.12
		Mean	-	-	-	-	**100**	0.34	1	1.39
**15f**	iv	1	28.5	1.7	2.7	1.0	-	1.08	-	0.677
		2	14.2	1.3	5.1	1.5	-	1.71	-	1.36
		Mean	21.4	1.5	3.9	1.2	-	1.40	-	1.02
	oral	3	-	-	-	-	63	0.154	1	0.631
		4	-	-	-	-	42	0.112	1	0.423
		Mean	-	-	-	-	**52**	0.133	1	0.527
**15b**	iv	1	10.1	1.6	2.0	2.6	-	0.885	-	1.60
		2	8.4	3.1	5.5	6.1	-	1.01	-	1.93
		Mean	9.2	2.3	3.8	4.2	-	0.948	-	1.77
	oral	3	-	-	-	-	21	0.139	1.5	0.720
		4	-	-	-	-	43	0.260	1.5	1.49
		Mean	-	-	-	-	**32**	0.200	1.5	1.11
**15g**	iv	1	18.7	1.4	2.2	1.3	-	1.26	-	1.03
		2	13.3	1.1	1.6	1.4	-	1.69	-	1.44
		Mean	16.0	1.3	1.9	1.3	-	1.48	-	1.24
	oral	3	-	-	-	-	86	0.609	1	1.98
		4	-	-	-	-	66	0.523	1	1.60
		Mean	-	-	-	-	**76**	0.566	1	1.79
**15h**	iv	1	52.9	1.4	1.9	0.45	-	0.732	-	0.346
		2	50.4	1.3	2.2	0.43	-	0.842	-	0.363
		Mean	51.7	1.4	2.1	0.44	-	0.787	-	0.355
	oral	3	-	-	-	-	4	0.014	1.5	0.029
		4	-	-	-	-	10	0.021	1	0.069
		Mean	-	-	-	-	**7**	0.017	1.25	0.049

[a]effective half-life [h] = $0.693 \times V_{ss}/(CL \times 60/1000)$

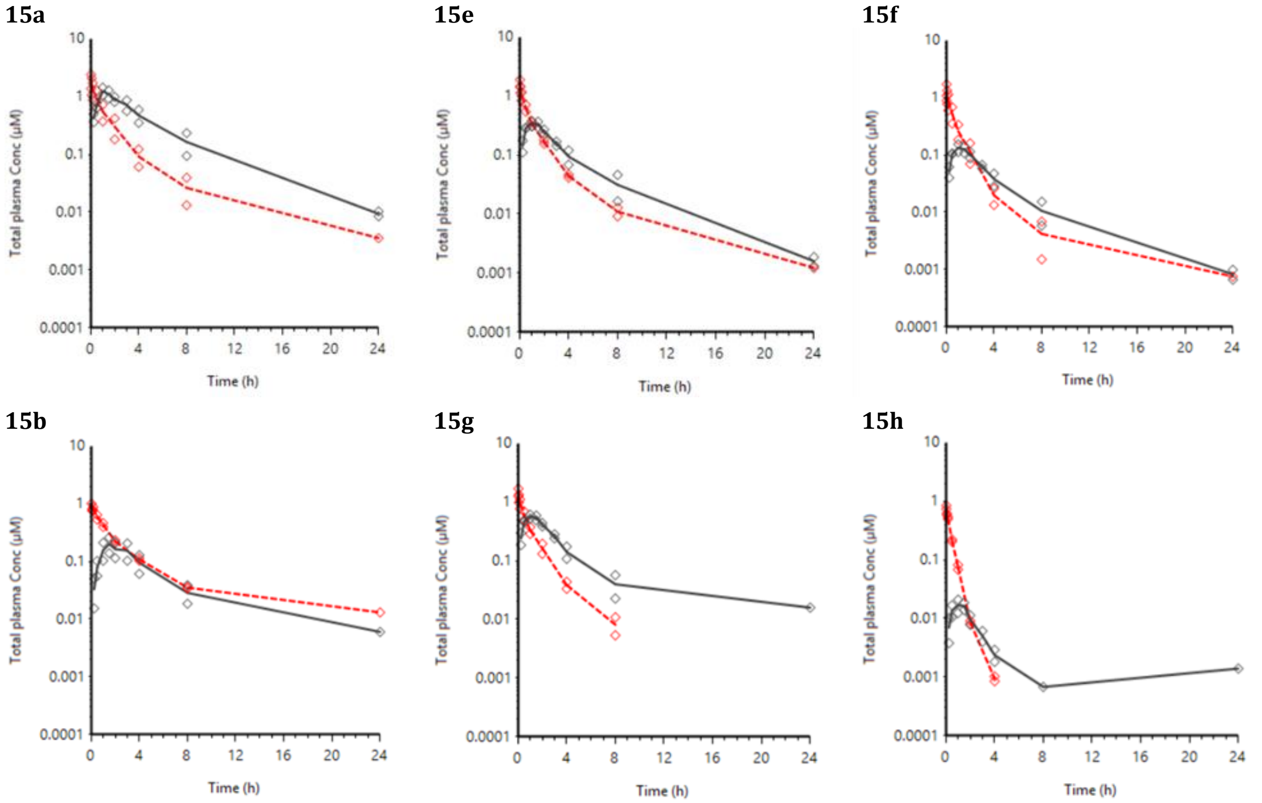

Figure S4. Total plasma concentration over time. Solid black line: Mean PO (0.5 mg/kg p.o. for **15e**, **15f** and 1.0 mg/kg PO for **15a**, **15b**, **15g**, **15h**); Dashed red line: Mean IV (0.5mg/kg); Open black diamonds: Individual rat PO data; Open red diamonds: Individual rat IV data.

3. NMR Spectra

3.1 Isothiocyanates

(*R*)-4-(4-(5-(Isothiocyanatomethyl)-2-oxooxazolidin-3-yl)phenyl)morpholin-3-one (*S6*)

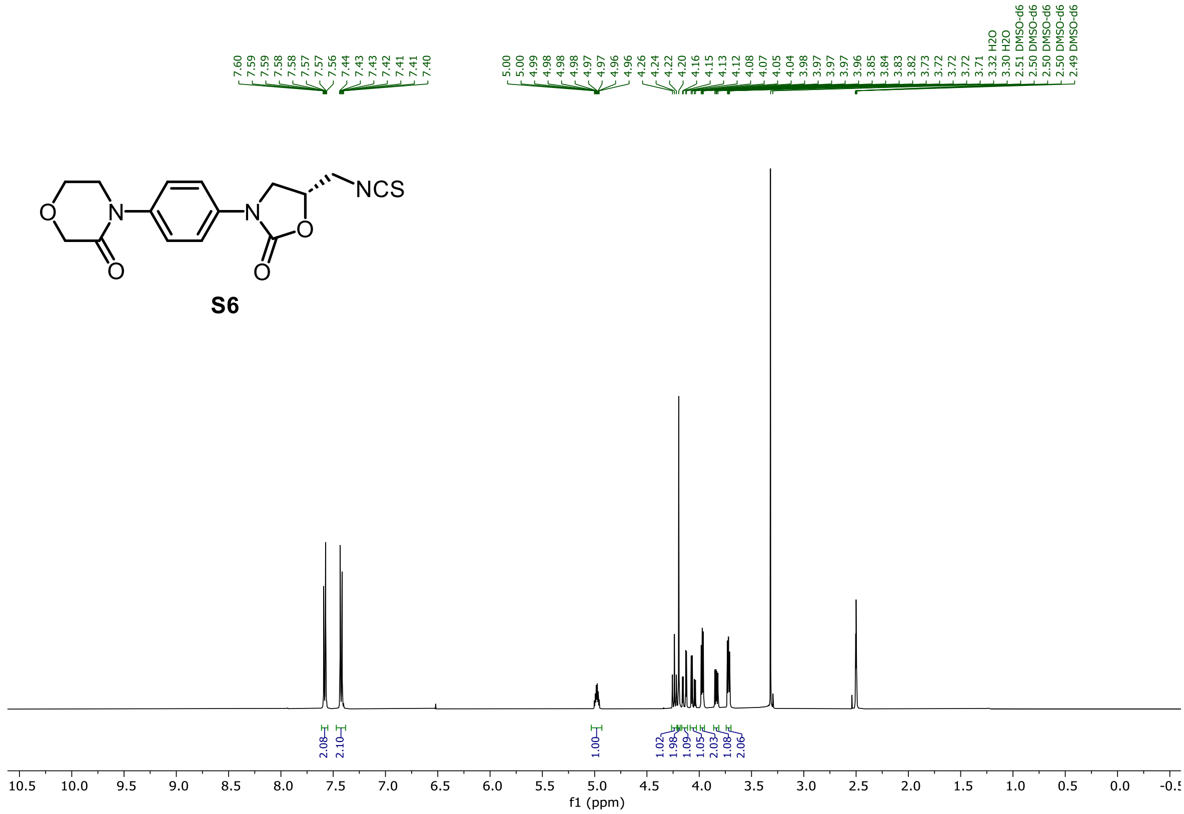

^{1}HNMR spectrum of **S6** run in DMSO-d_6 at 500 MHz.

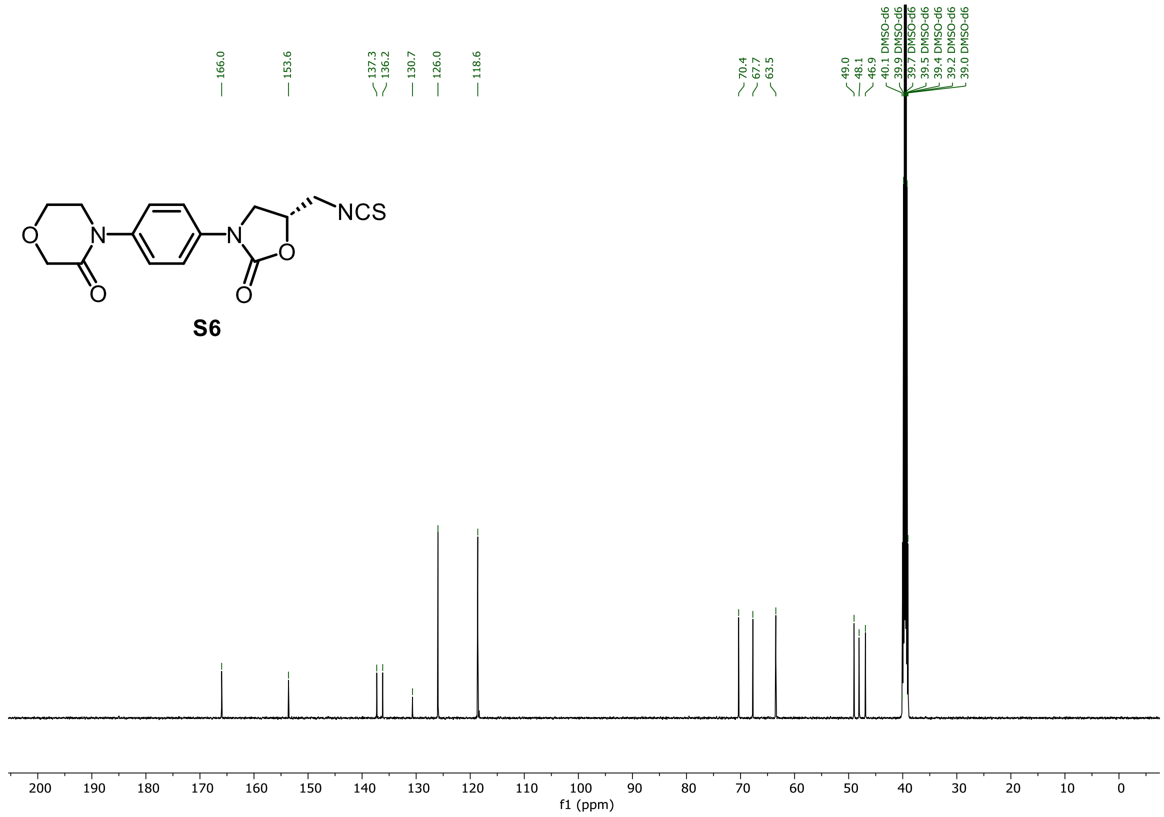

^{13}CNMR spectrum of **S6** run in DMSO-d_6 at 126 MHz.

1-(Allyloxy)-4-isothiocyanatobenzene (*S7*)

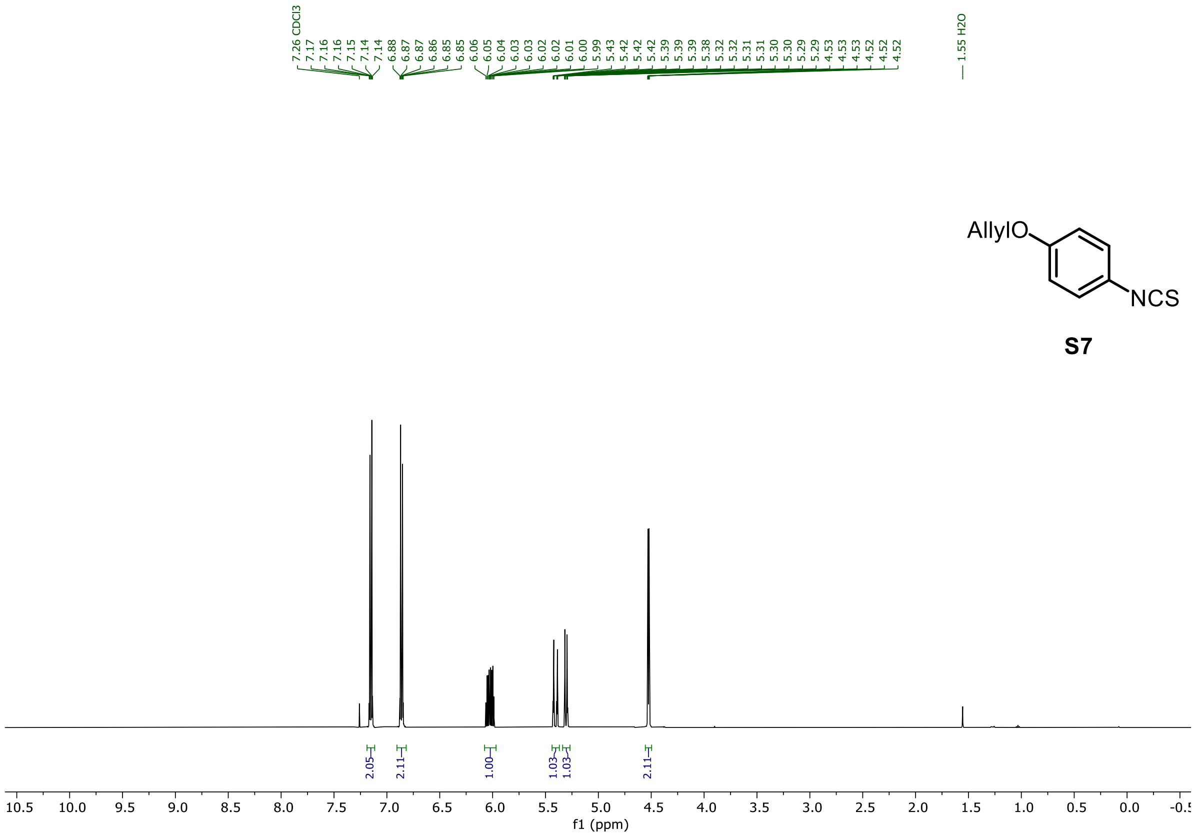

^{1}HNMR spectrum of **S7** run in $CDCl_3$ at 500 MHz.

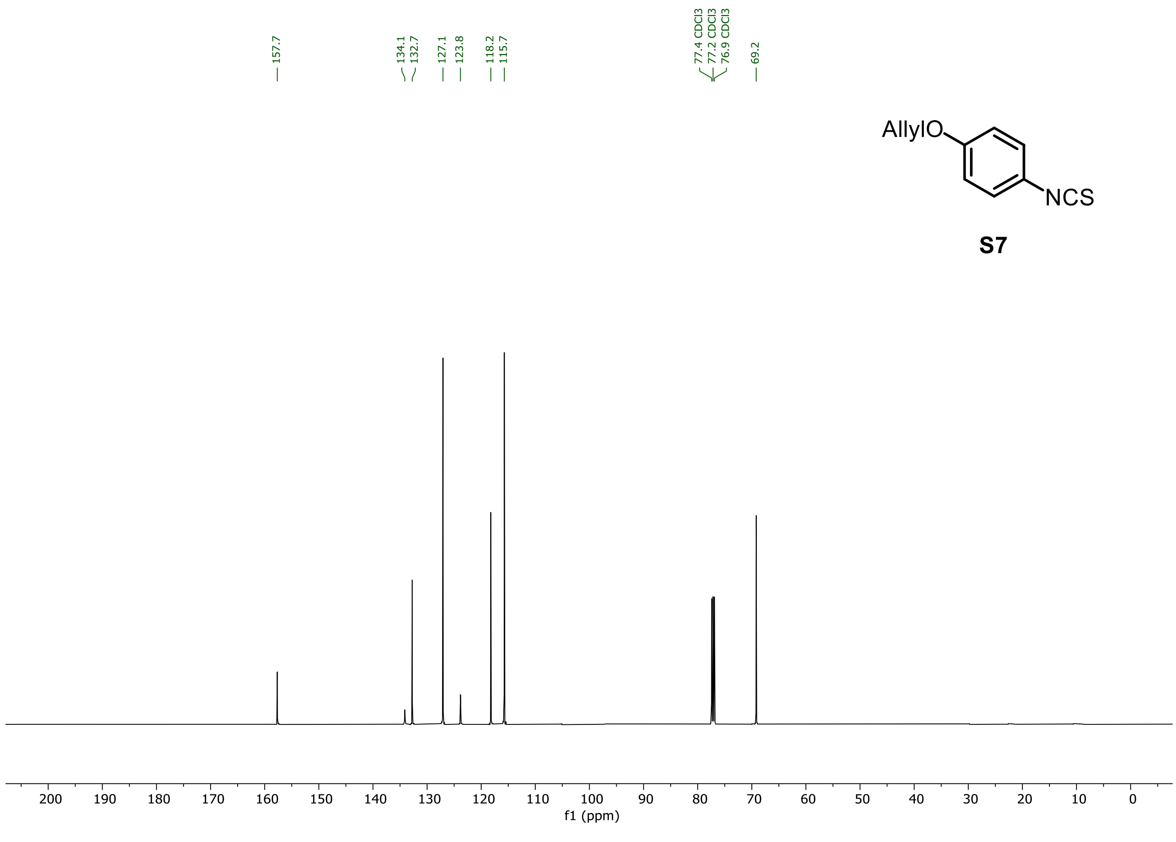

^{13}CNMR spectrum of **S7** run in $CDCl_3$ at 126 MHz.

3.2 *N*-CF_3 carbamoyl fluorides

N-(Trifluoromethyl)(3,4,5-trimethoxyphenyl)carbamoyl fluoride (S8)

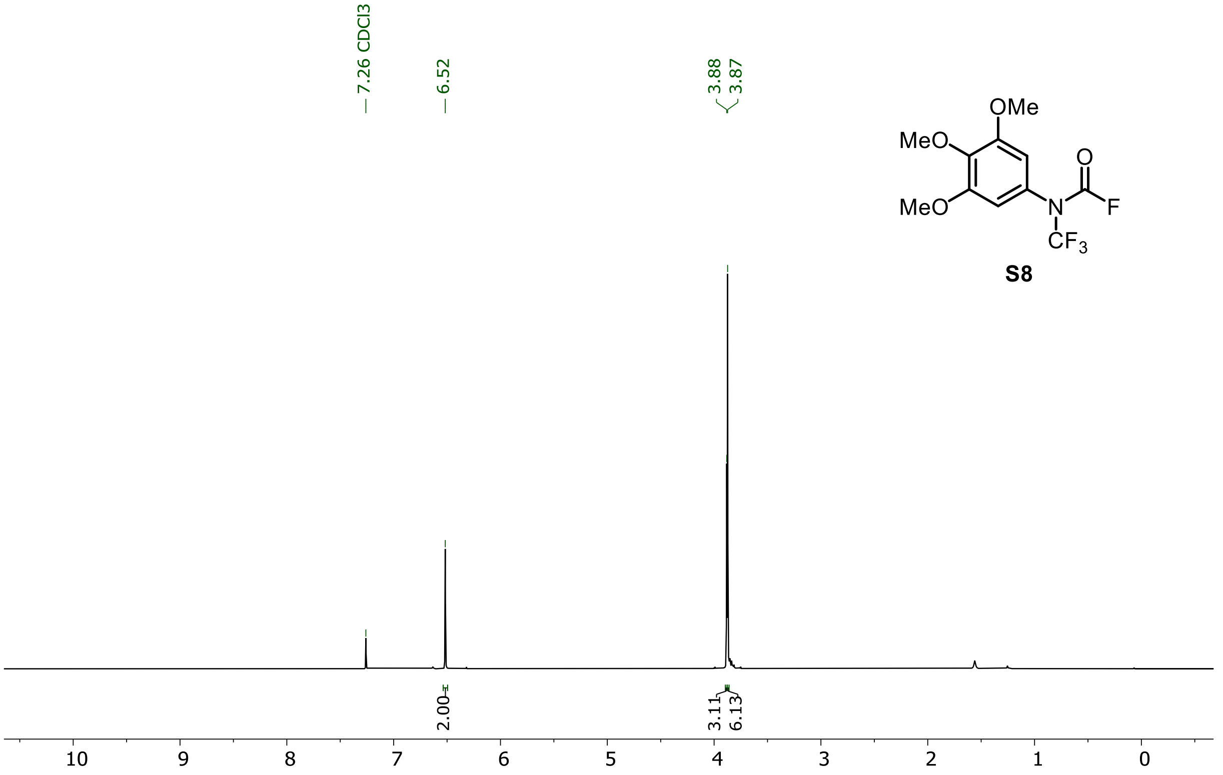

^{1}HNMR spectrum of **S8** run in $CDCl_3$ at 600 MHz.

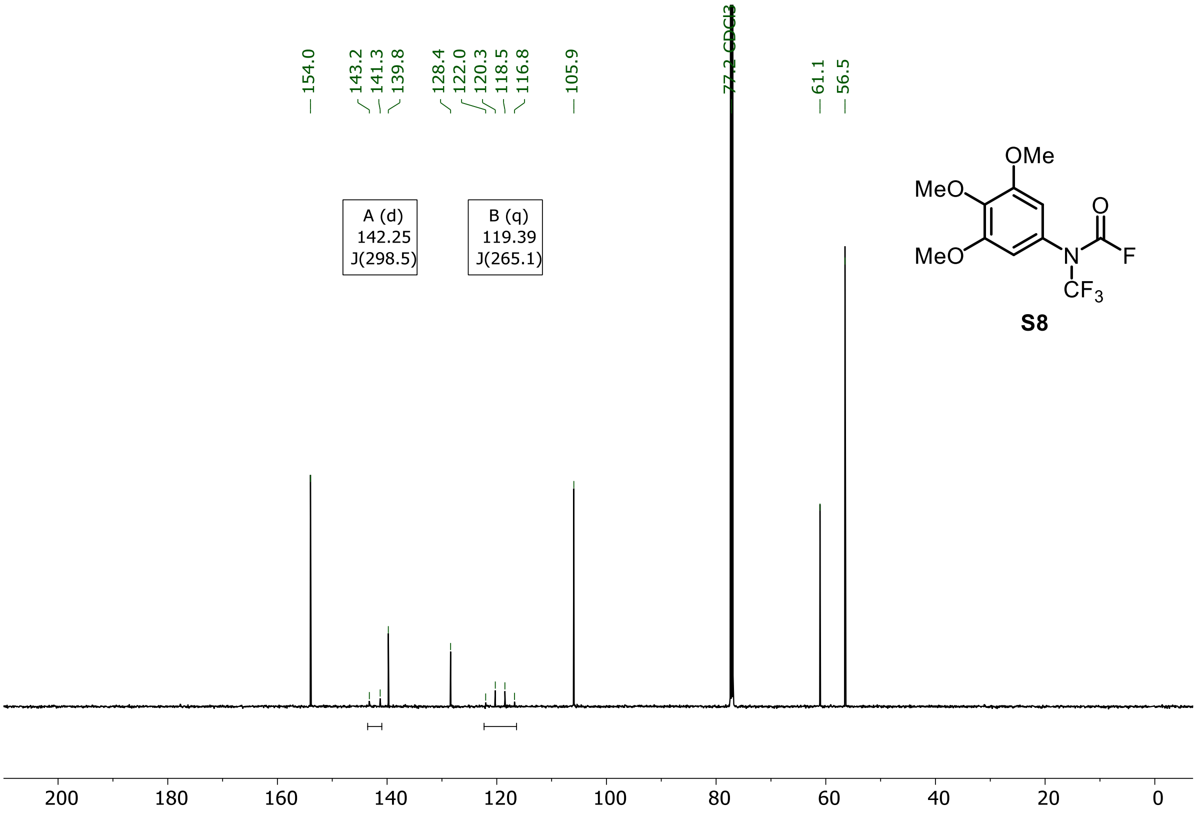

^{13}CNMR spectrum of **S8** run in $CDCl_3$ at 151 MHz.

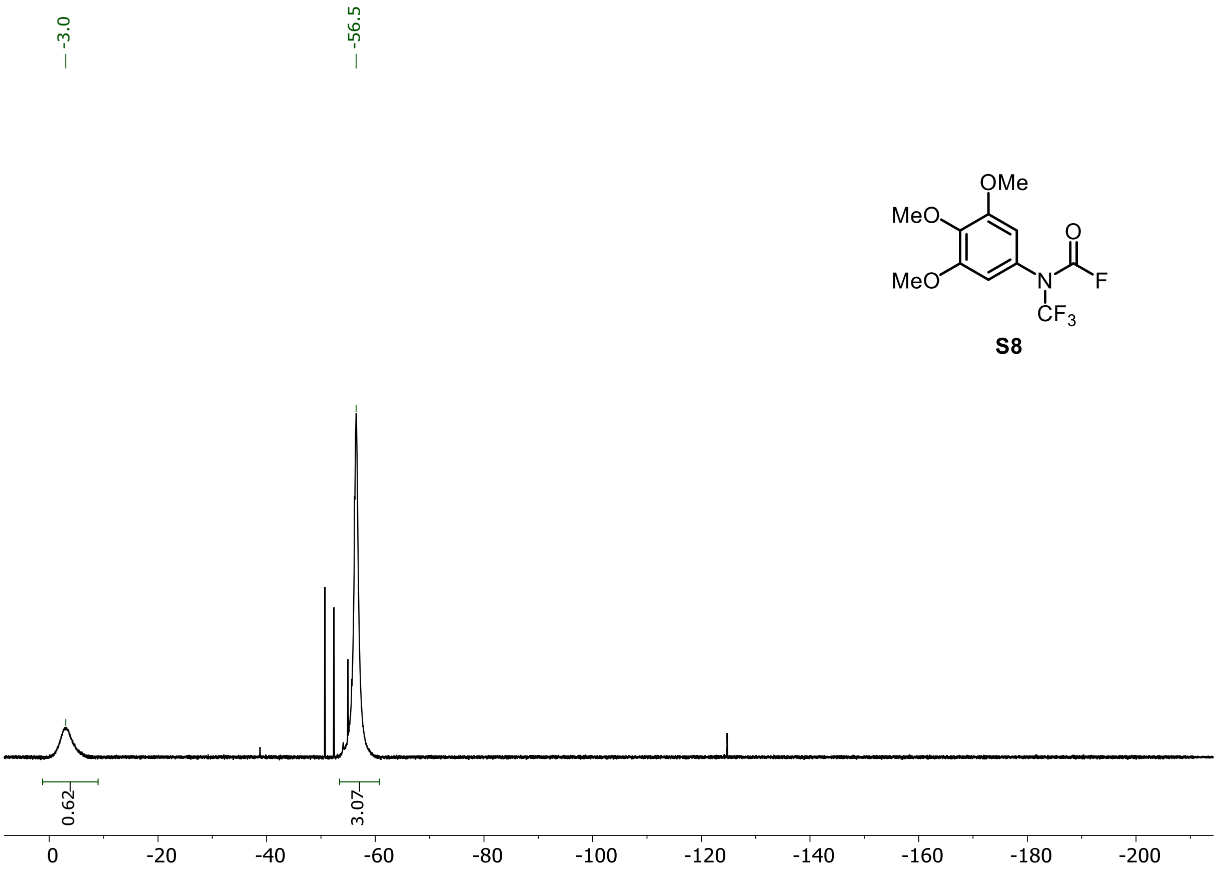

^{19}FNMR spectrum of **S8** run in $CDCl_3$ at 376 MHz

(4-Methoxybenzyl)-*N*-(trifluoromethyl)carbamoyl fluoride (*S*9)

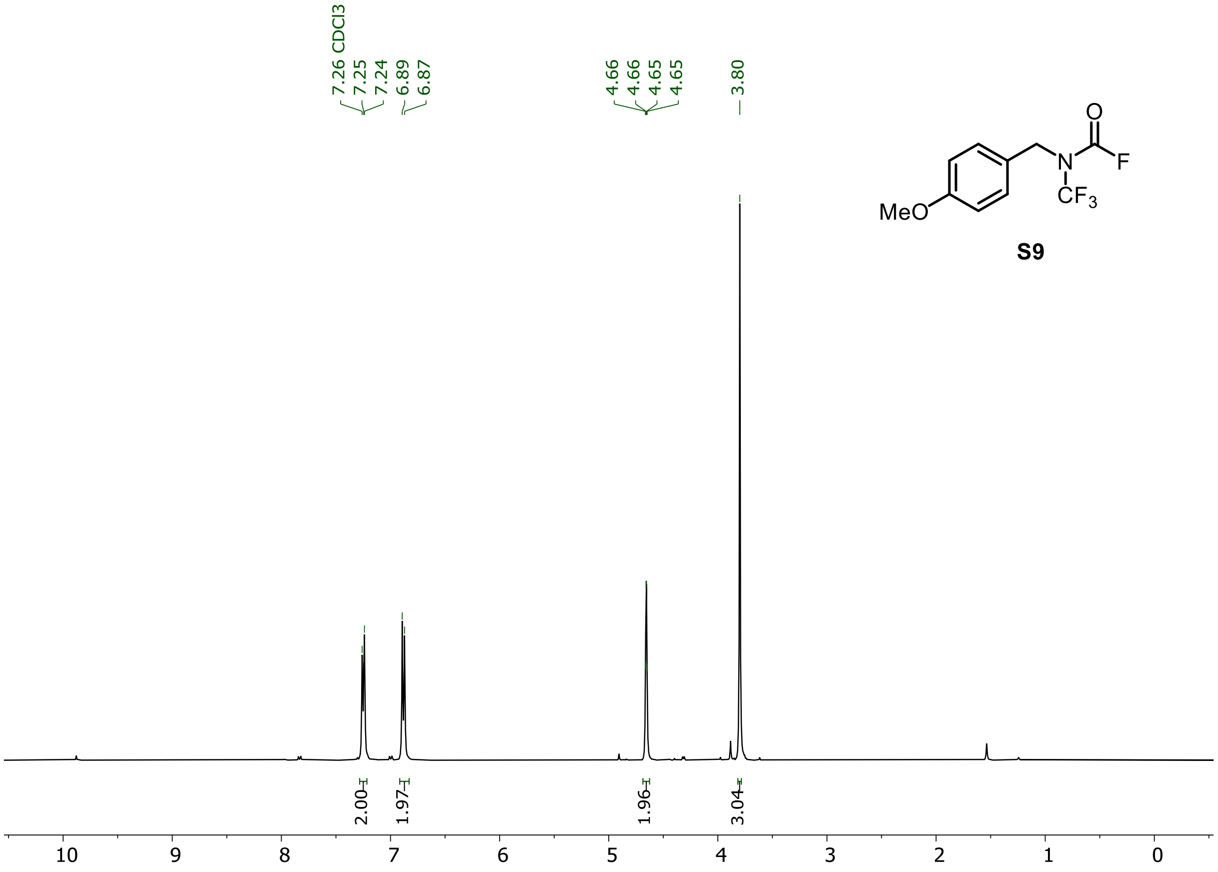

^{1}HNMR spectrum of **S9** run in $CDCl_3$ at 400 MHz.

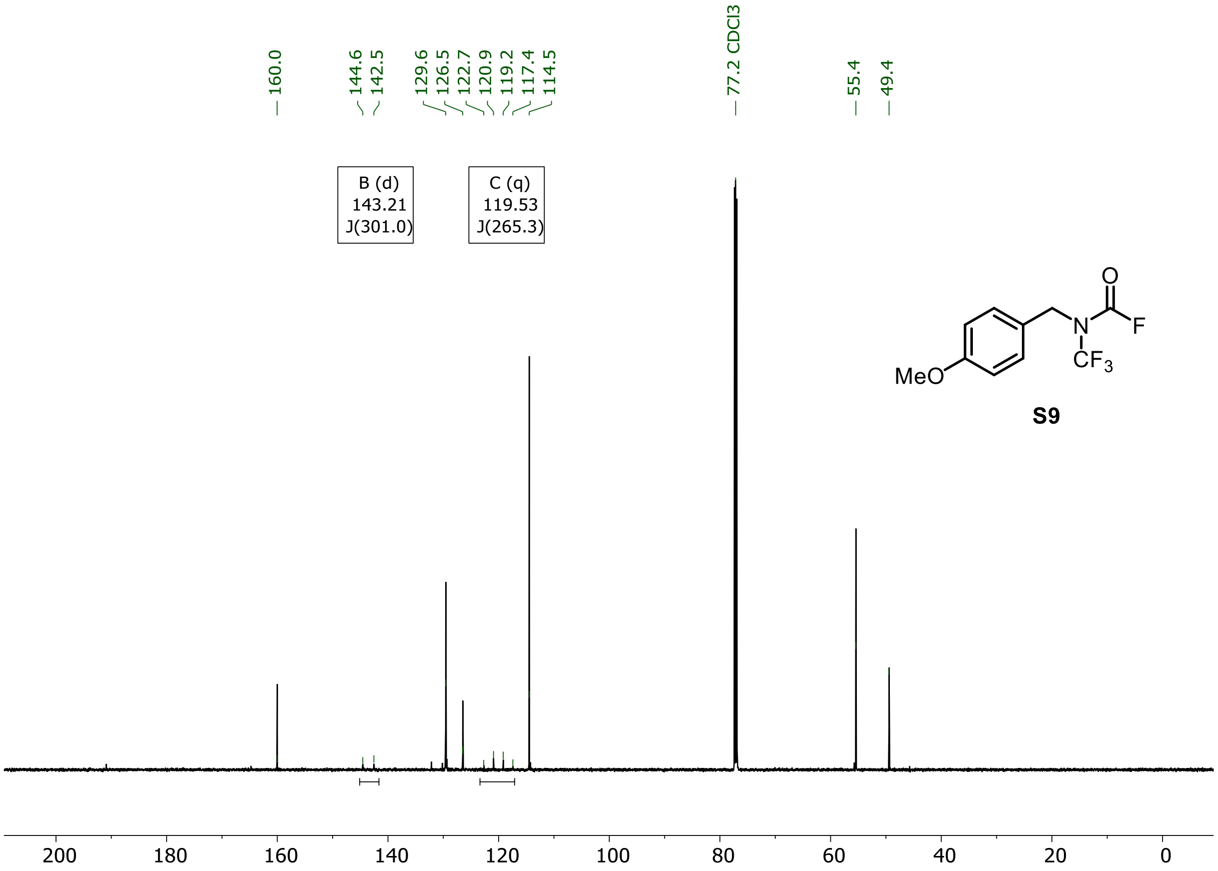

^{13}CNMR spectrum of **S9** run in $CDCl_3$ at 151 MHz.

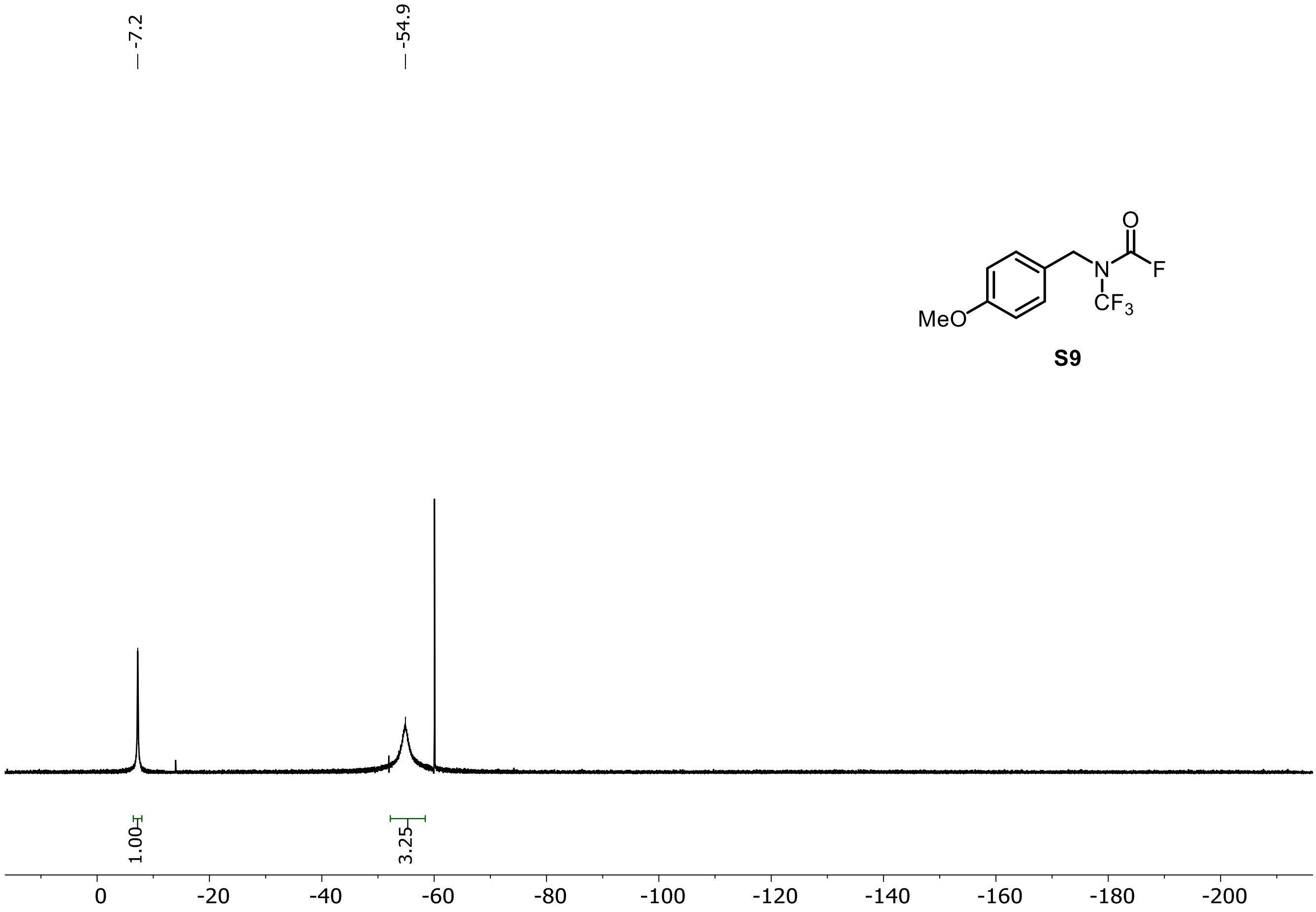

^{19}FNMR spectrum of **S9** run in $CDCl_3$ at 565 MHz.

(*R*)-((2-Oxo-3-(4-(3-oxomorpholino)phenyl)oxazolidin-5-yl)methyl)(trifluoromethyl) carbamoyl fluoride (18)

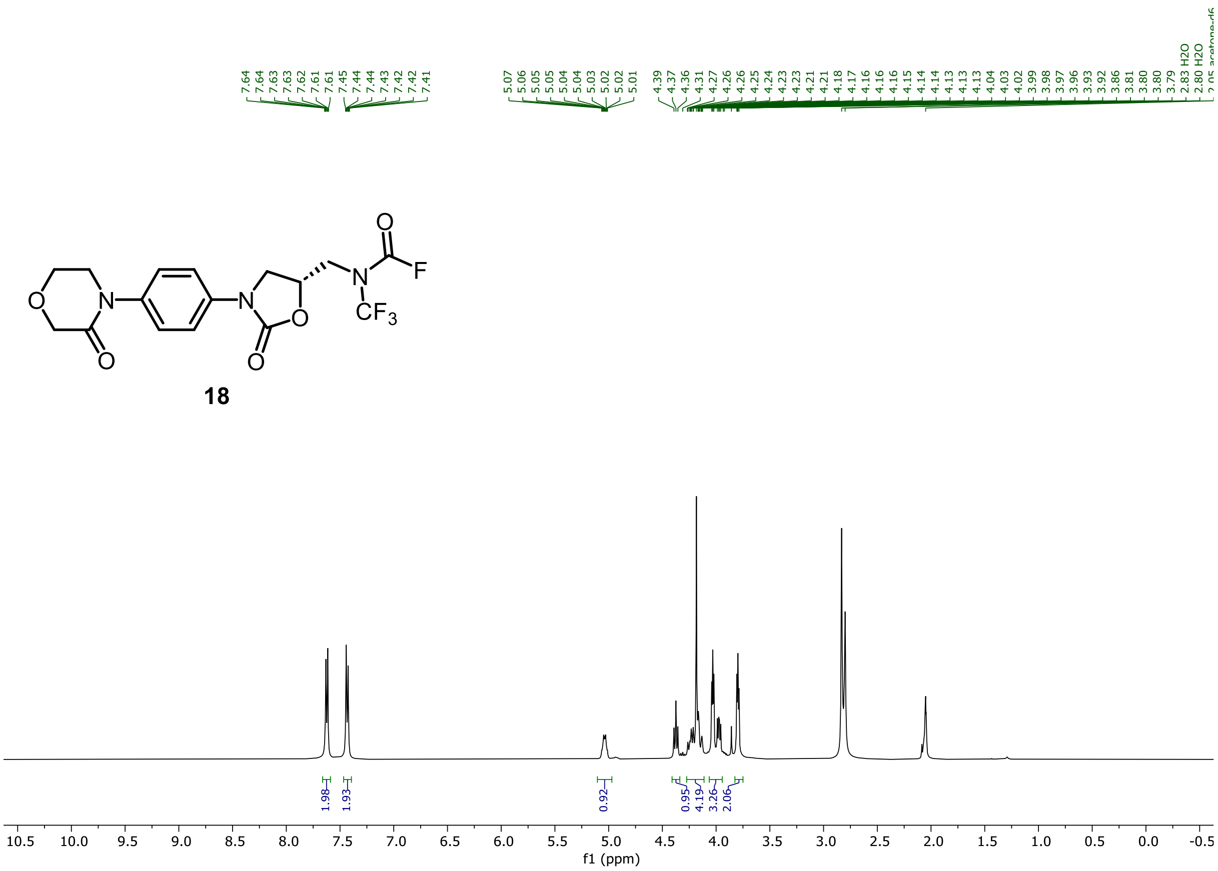

^{1}HNMR spectrum of **18** run in acetone-d_6 at 500 MHz.

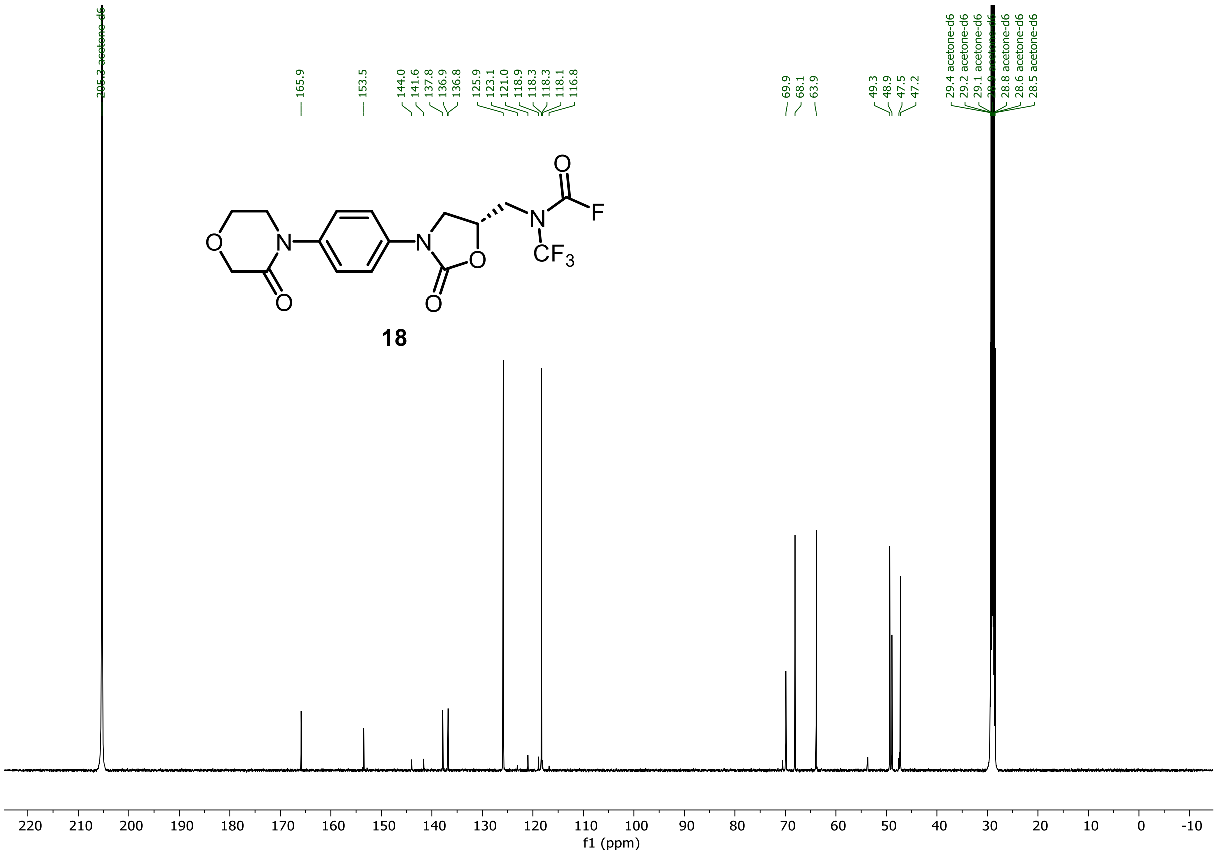

^{13}CNMR spectrum of **18** run in acetone-d_6 at 126 MHz.

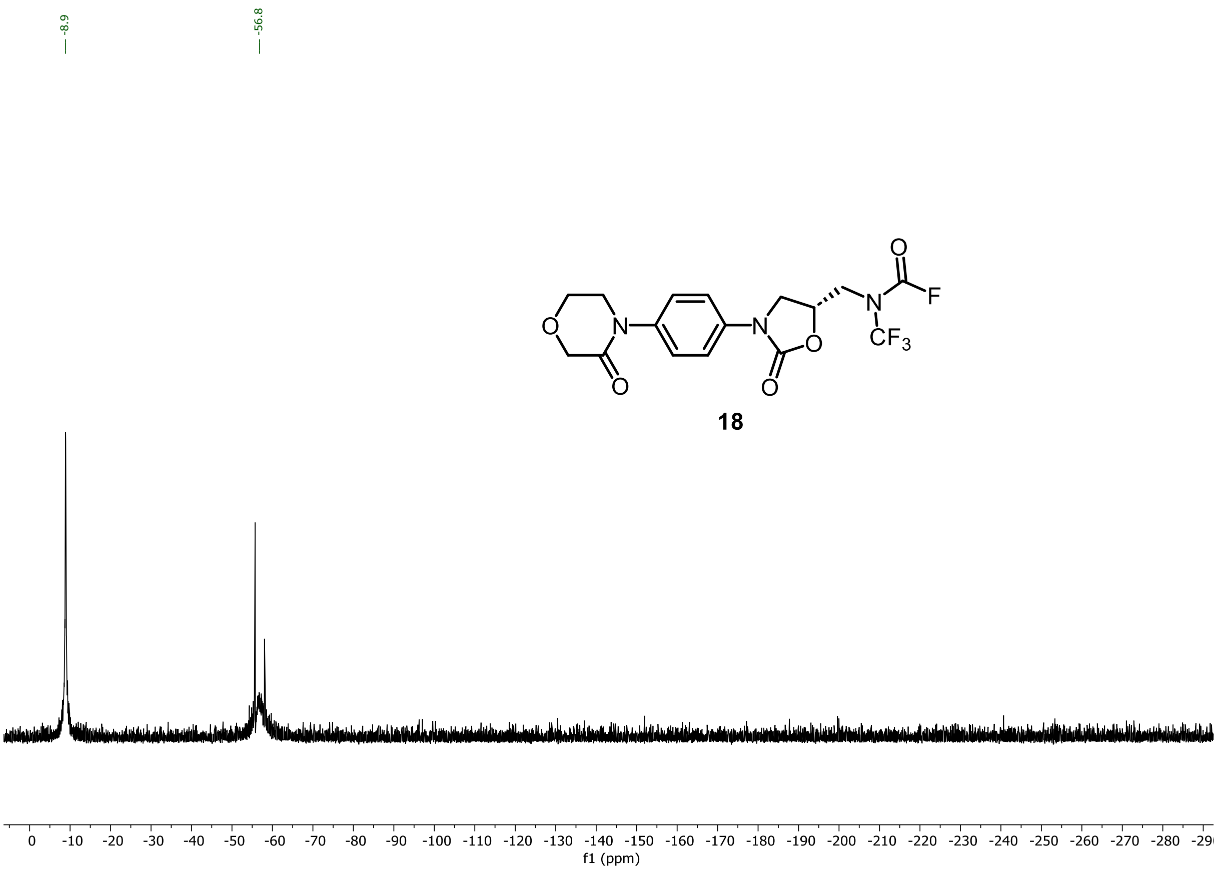

^{19}FNMR spectrum of **18** run in acetone-d_6 at 471 MHz.

(4-(Allyloxy)phenyl)-*N*-(trifluoromethyl)carbamoyl fluoride (20)

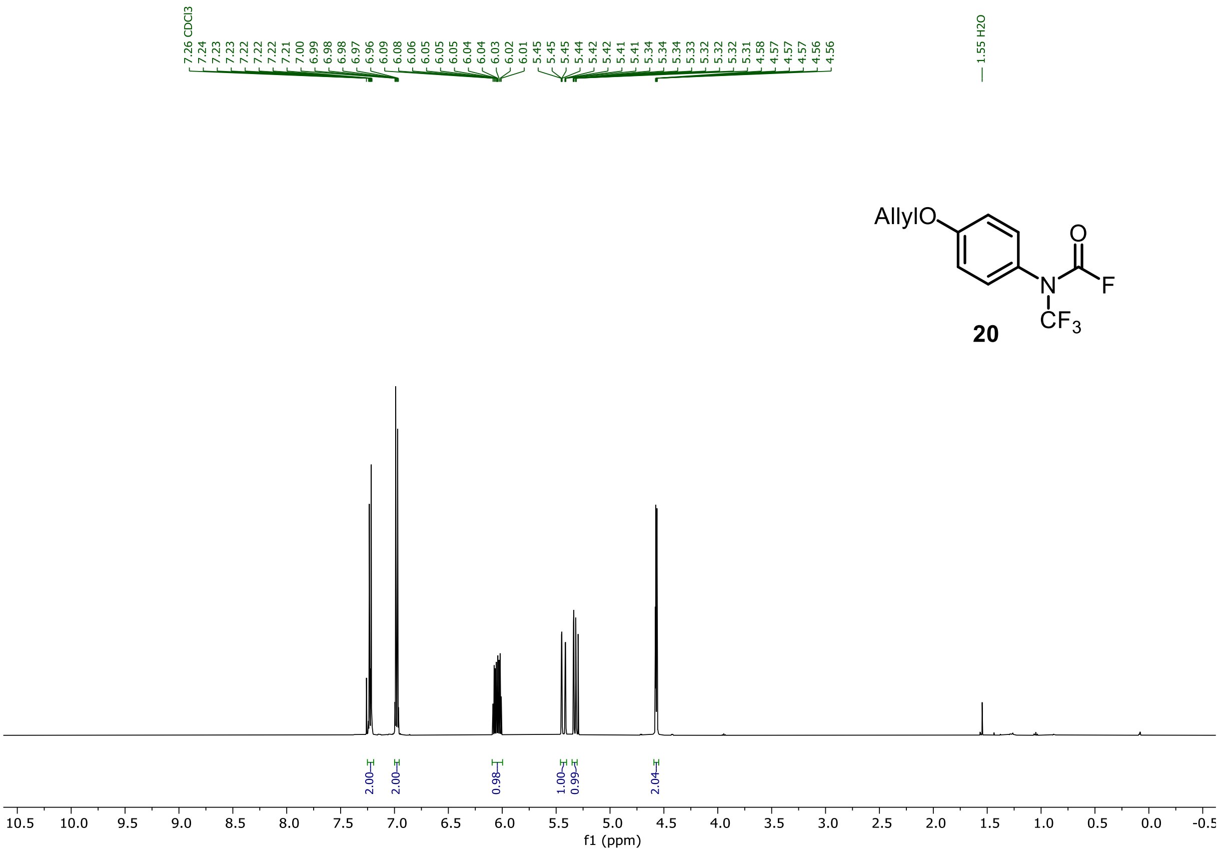

^{1}HNMR spectrum of **20** run in $CDCl_3$ at 500 MHz.

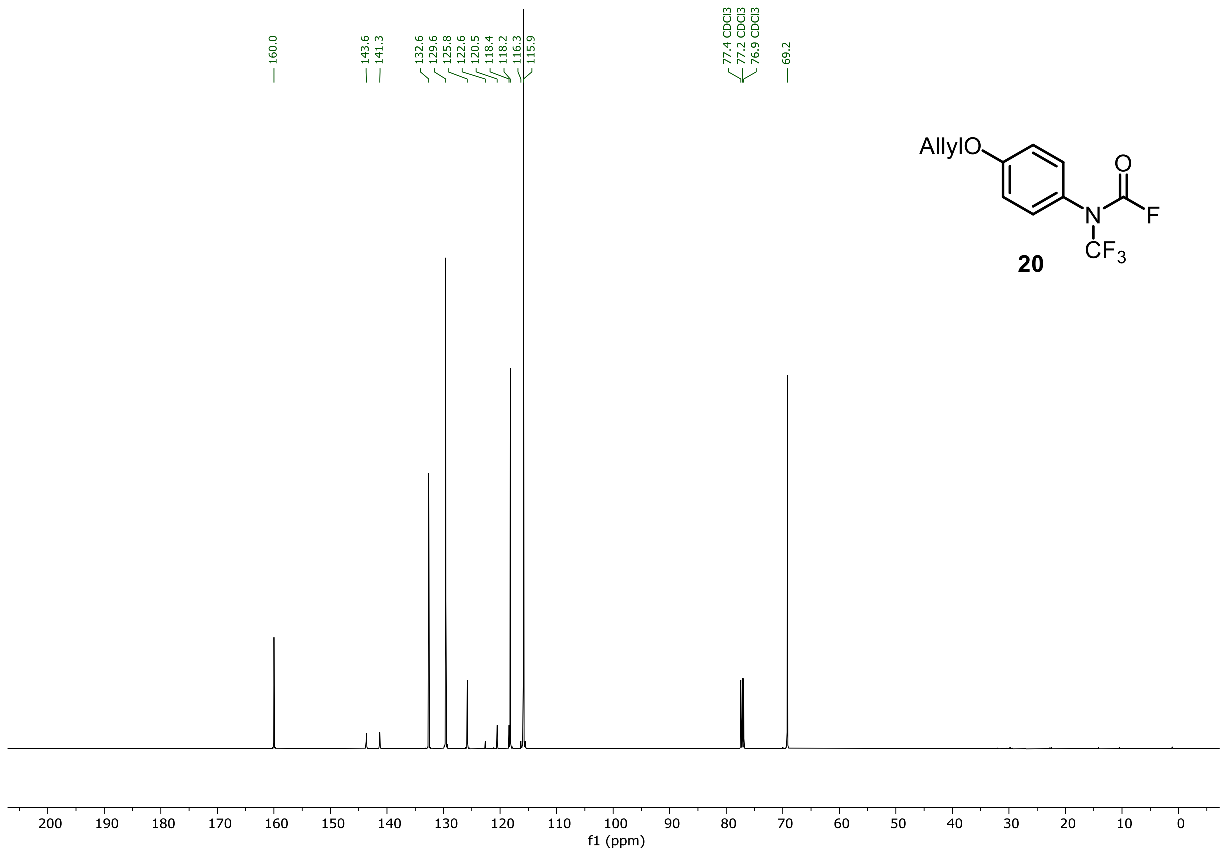

^{13}CNMR spectrum of **20** run in $CDCl_3$ at 126 MHz.

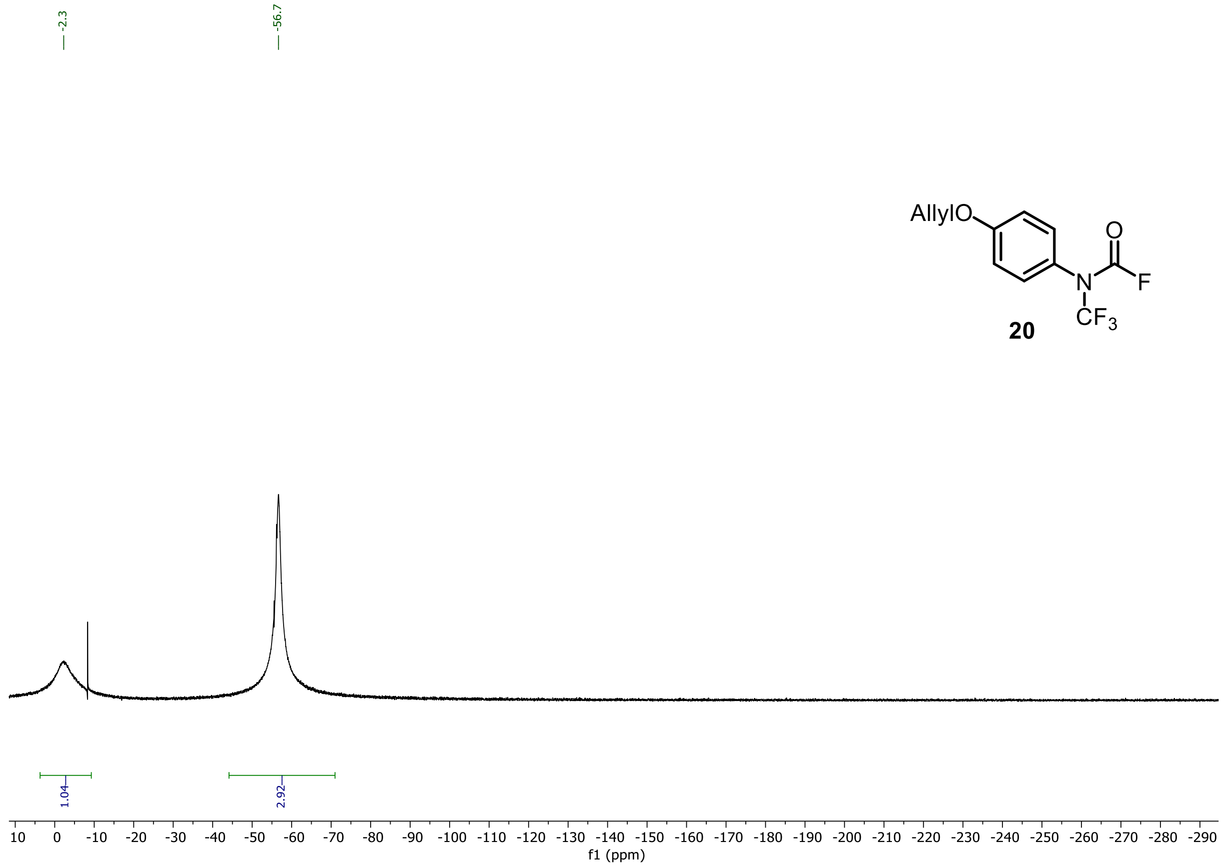

^{19}FNMR spectrum of **20** run in $CDCl_3$ at 471 MHz.

3.3 *N*-CF_3 amides

4-(Morpholinomethyl)-*N*-(trifluoromethyl)-*N*-(3,4,5-trimethoxyphenyl)benzamide (3c)

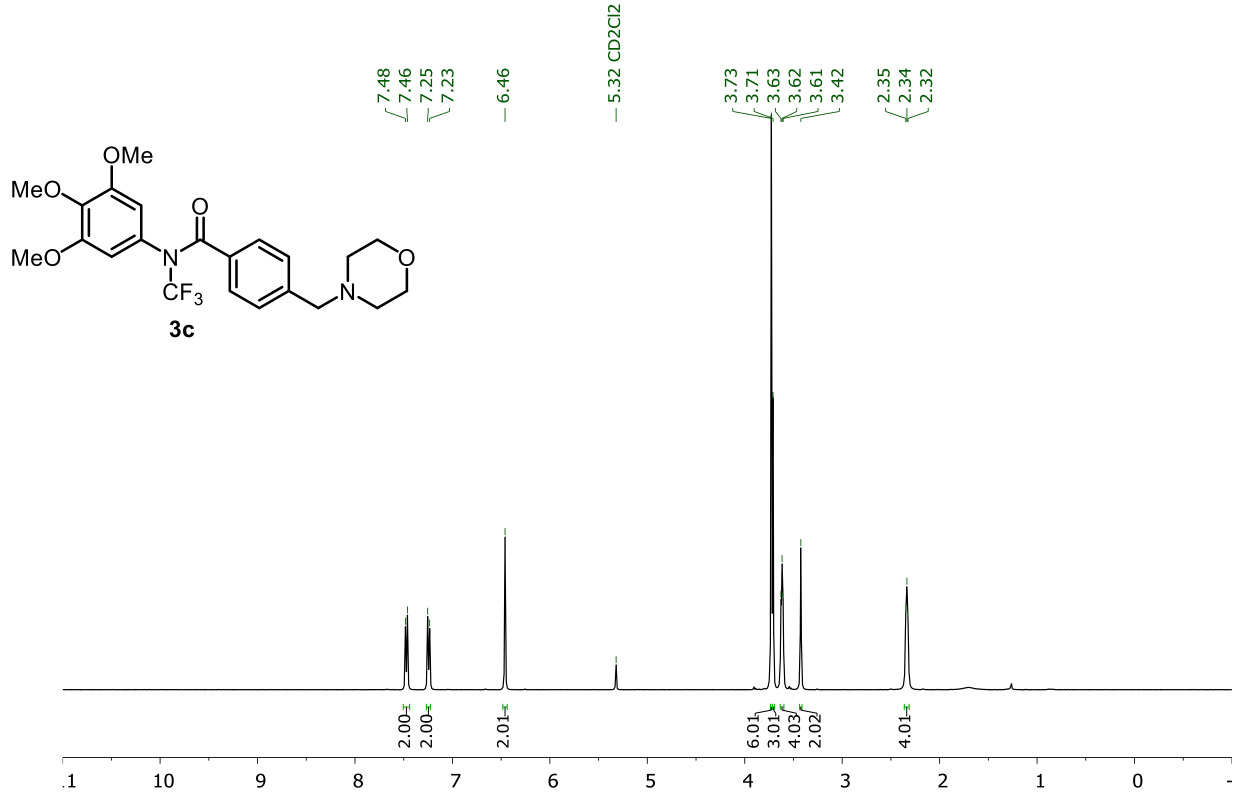

^{1}HNMR spectrum of **3c** run in CD_2Cl_2 at 400 MHz.

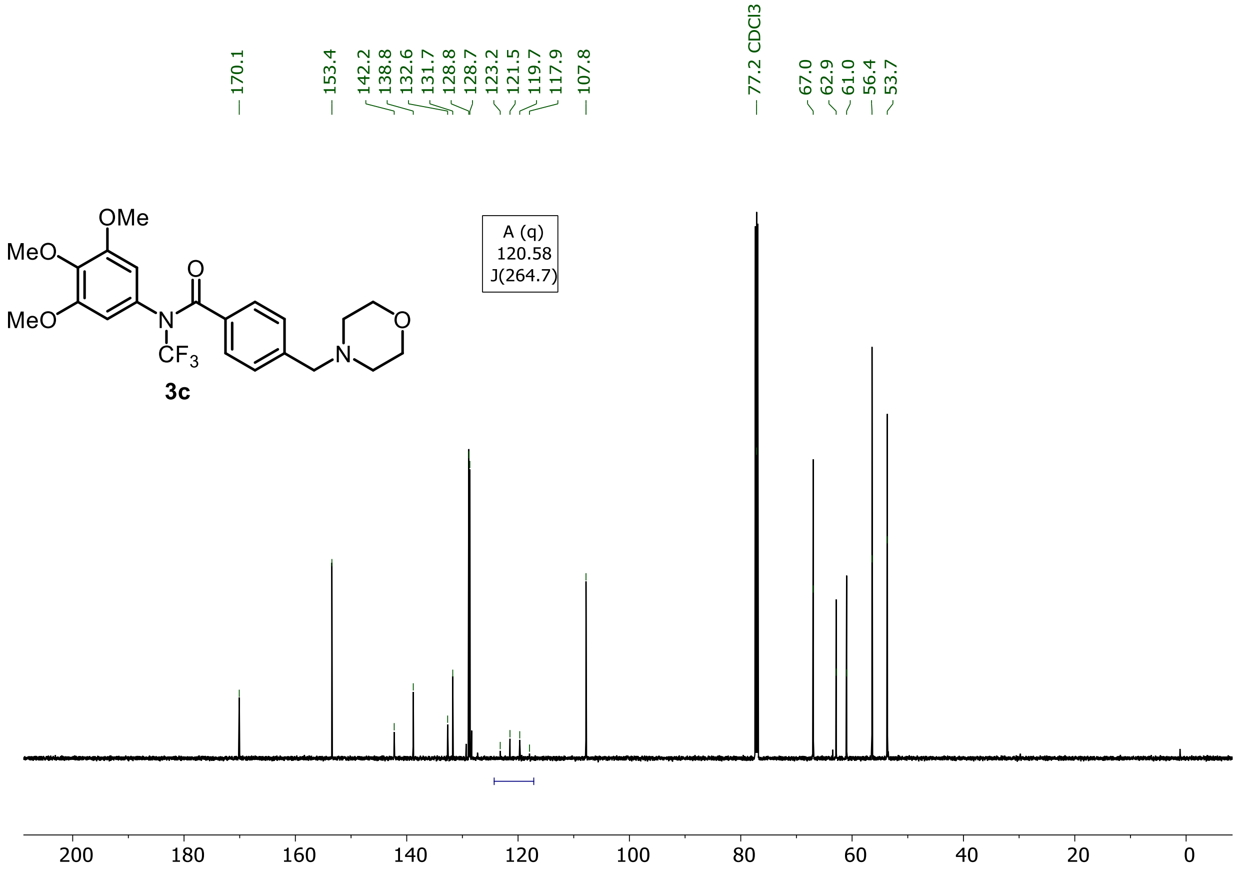

^{13}CNMR spectrum of **3c** run in CD_2Cl_2 at 151 MHz.

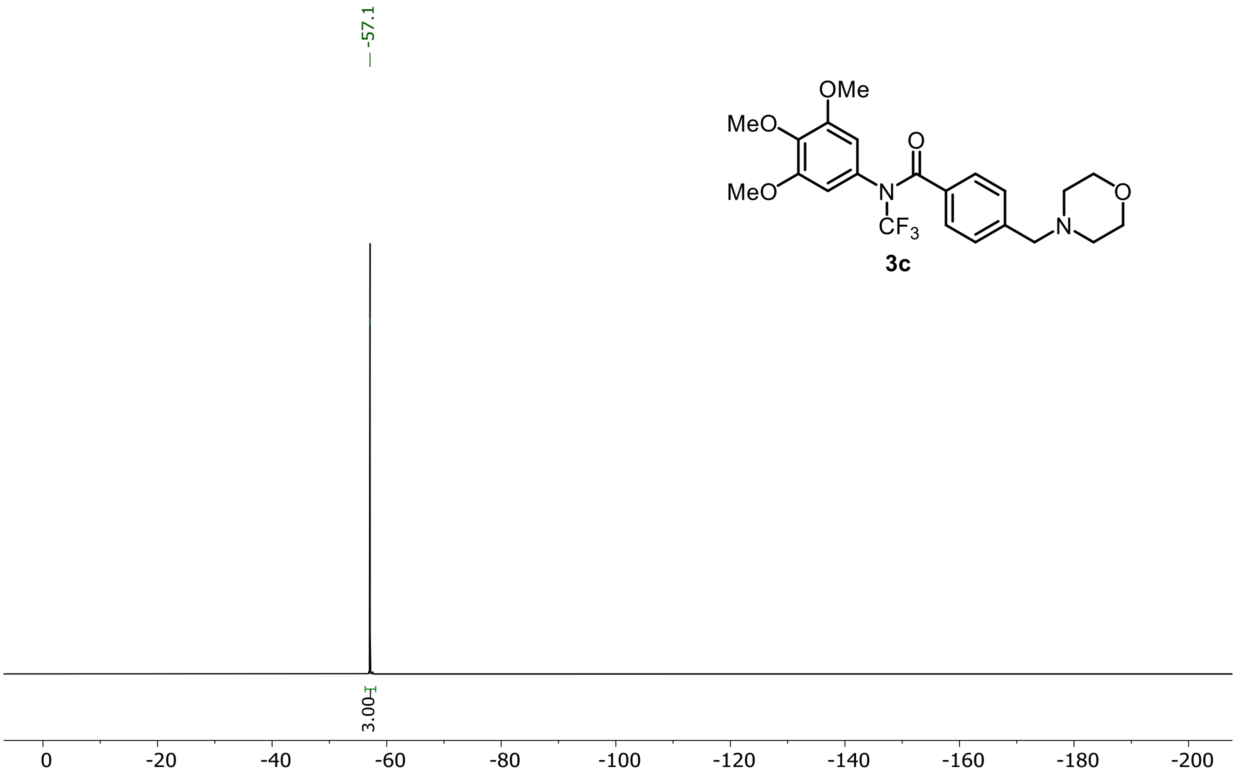

^{19}FNMR spectrum of **3c** run in CD_2Cl_2 at 376 MHz.

***N*-(Trifluoromethyl)-*N*-(3,4,5-trimethoxyphenyl)cyclopropanecarboxamide (4c)**

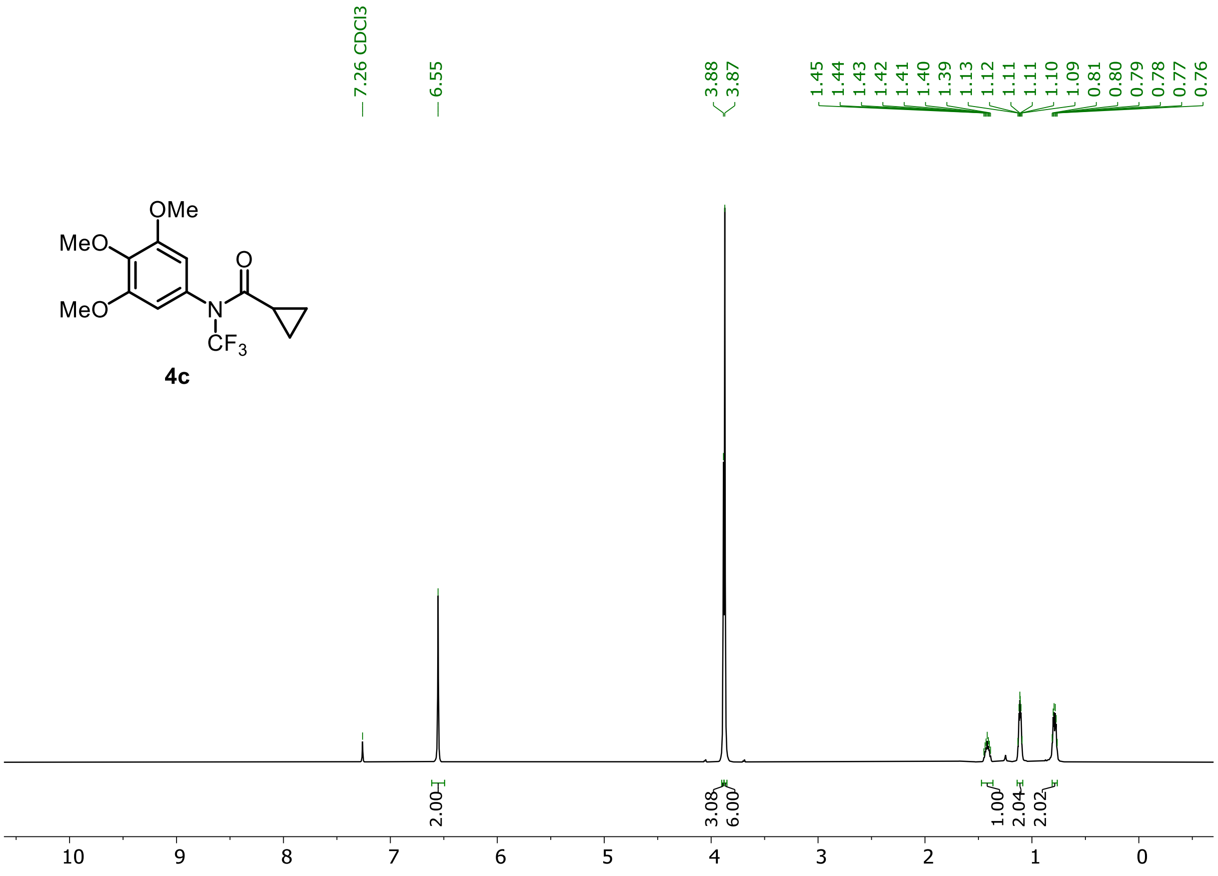

^{1}HNMR spectrum of **4c** run in $CDCl_3$ at 400 MHz.

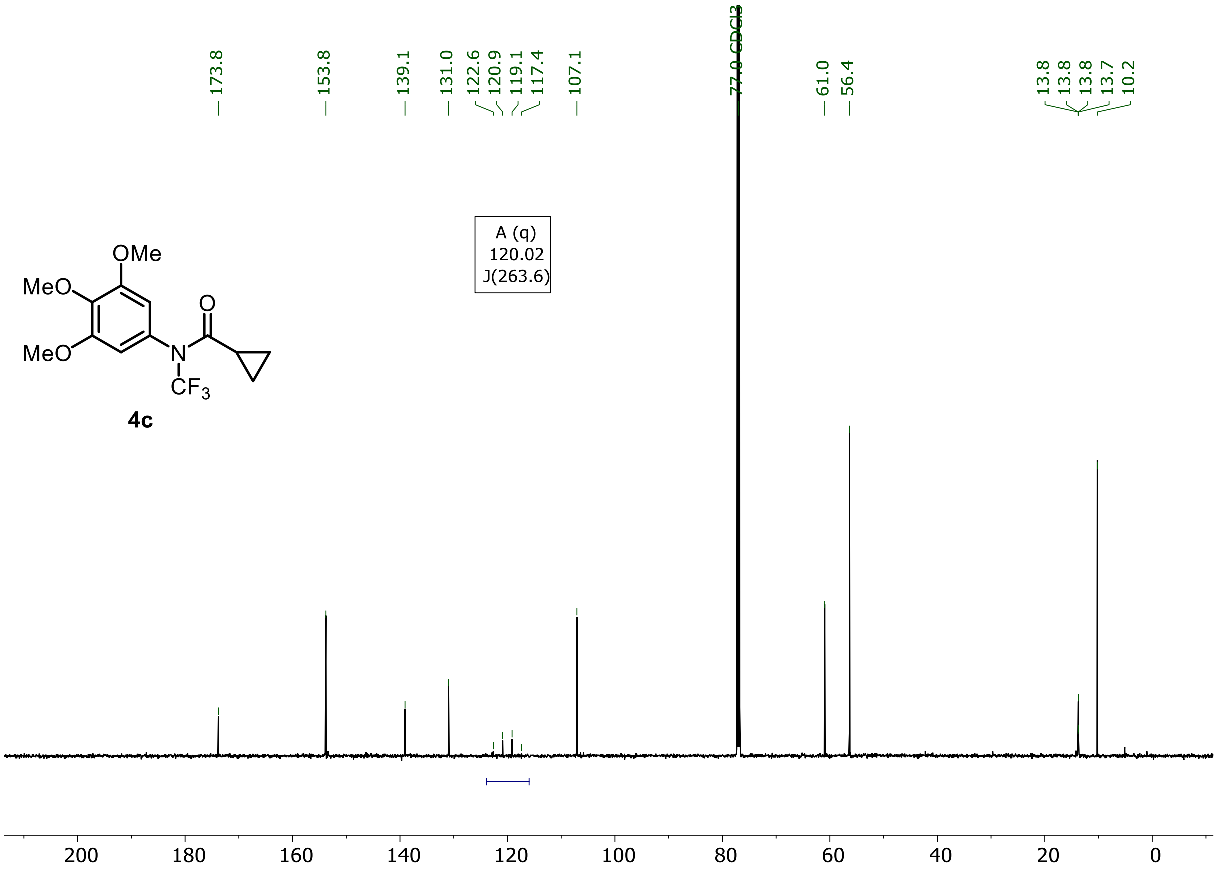

^{13}CNMR spectrum of **4c** run in $CDCl_3$ at 151 MHz.

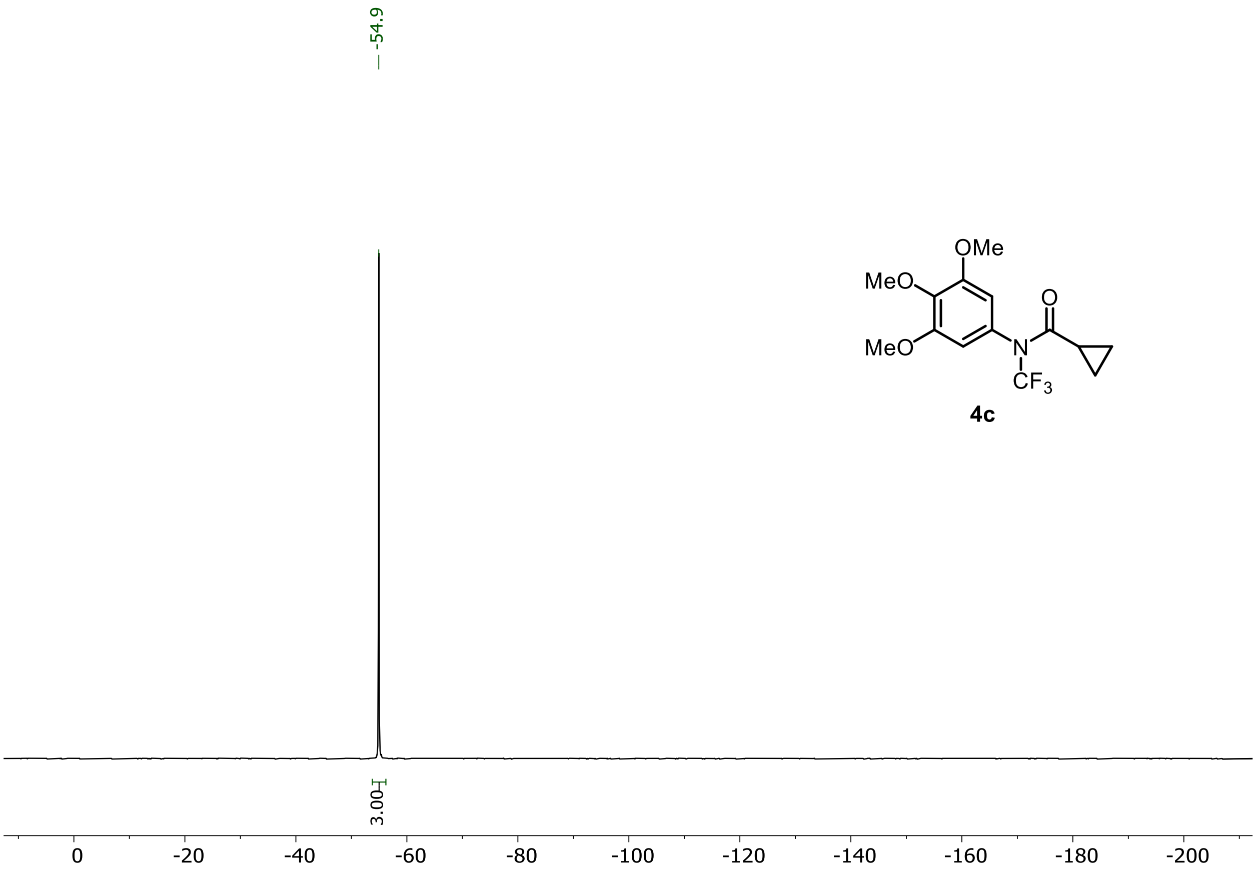

^{19}FNMR spectrum of **4c** run in $CDCl_3$ at 376 MHz.

(*R*)-*N*-((2-Oxo-3-(4-(3-oxomorpholino)phenyl)oxazolidin-5-yl)methyl)-*N*-trifluoromethyl acetamide (10a)

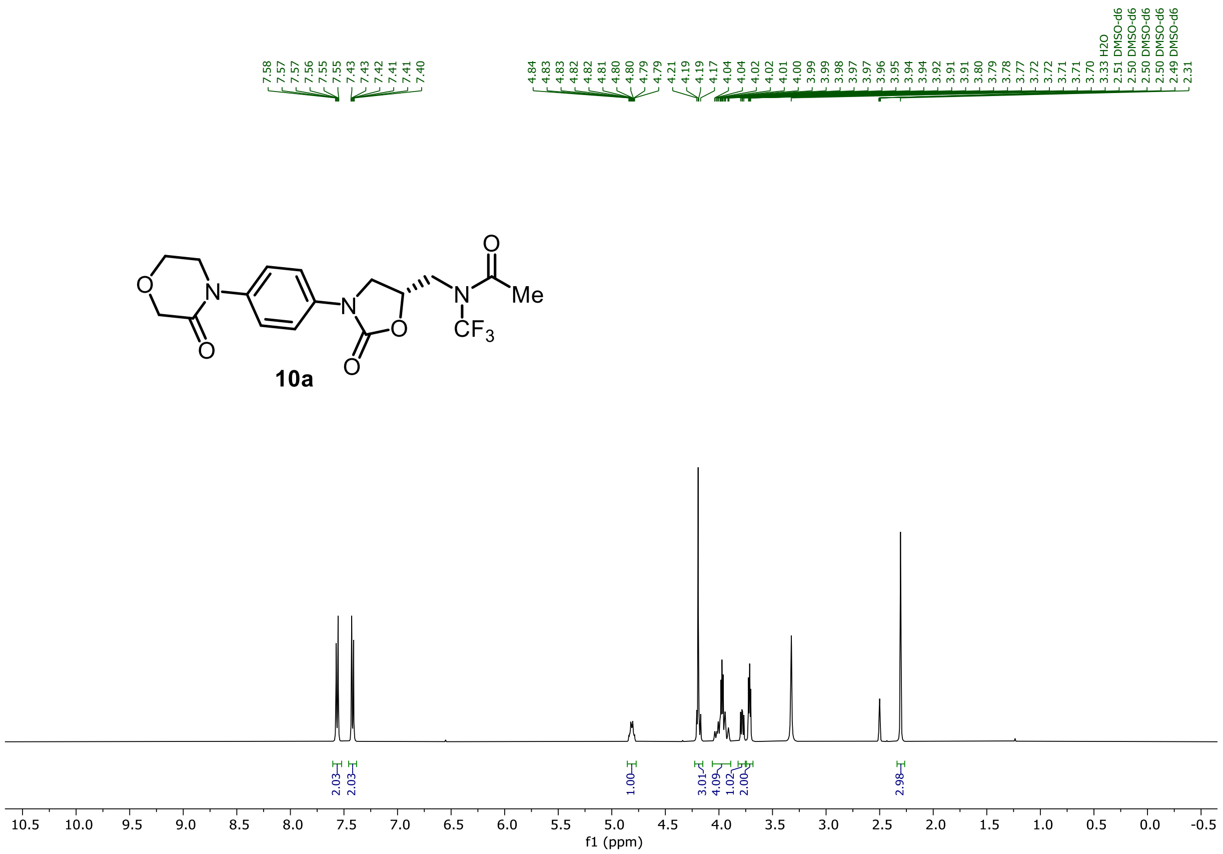

^{1}HNMR spectrum of **10a** run in DMSO-d_6 at 500 MHz.

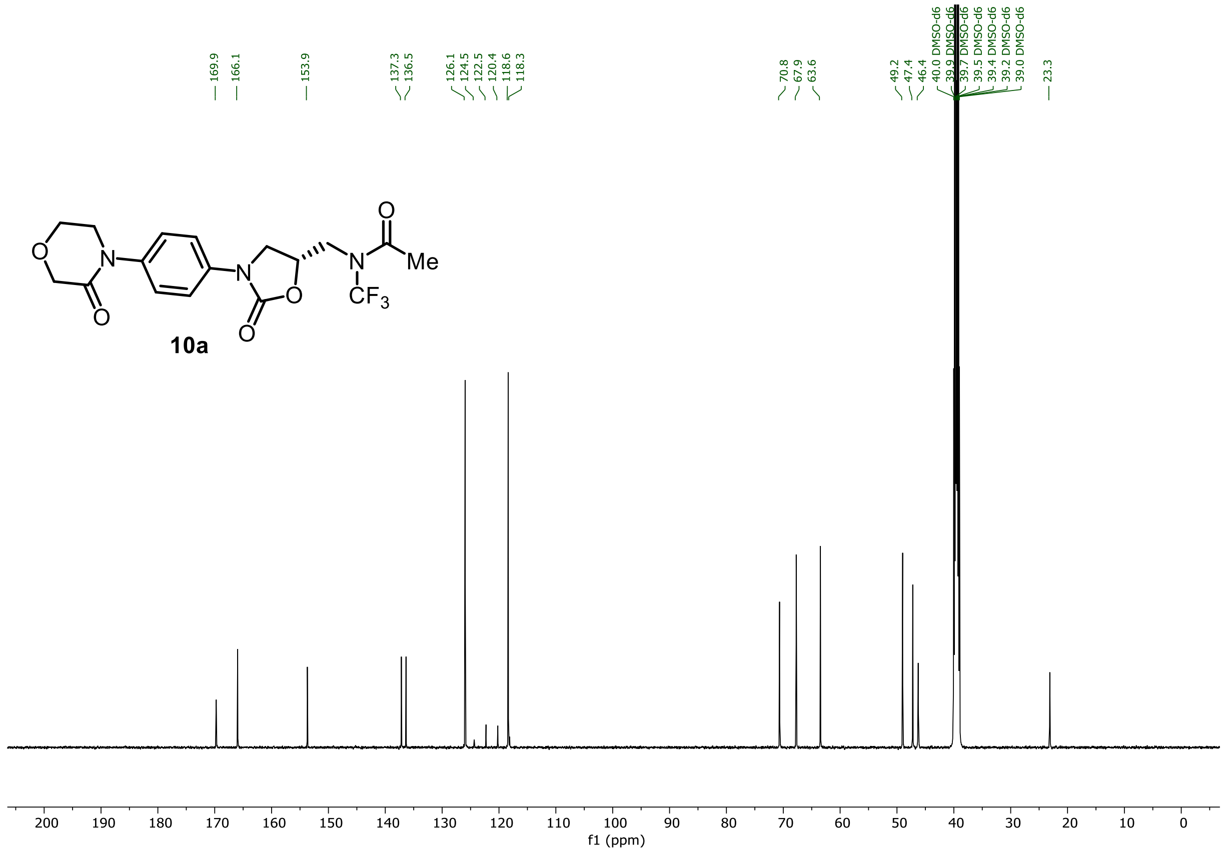

^{13}CNMR spectrum of **10a** run in DMSO-d_6 at 126 MHz.

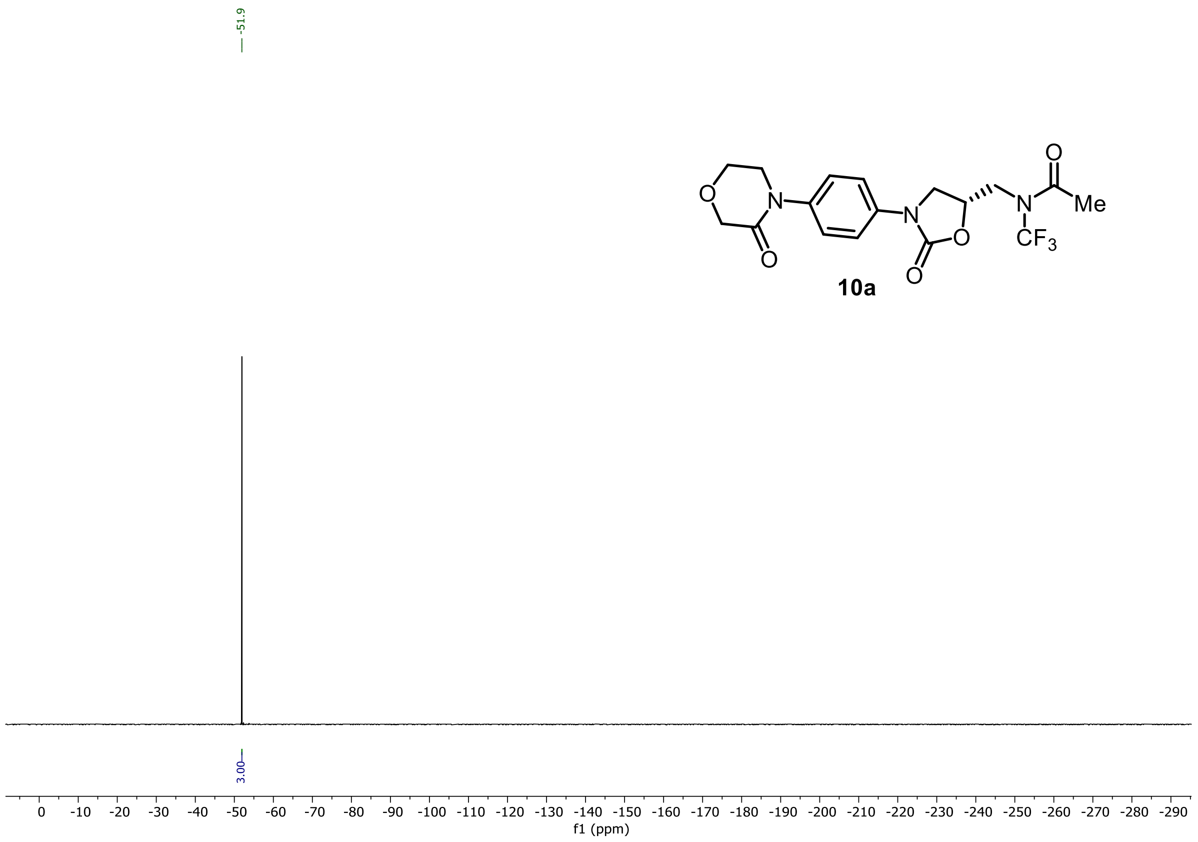

^{19}FNMR spectrum of **10a** run in DMSO-d_6 at 471 MHz.

(*R*)-*N*-((2-Oxo-3-(4-(3-oxomorpholino)phenyl)oxazolidin-5-yl)methyl)-*N*-(trifluoromethyl) cyclopropanecarboxamide (10b)

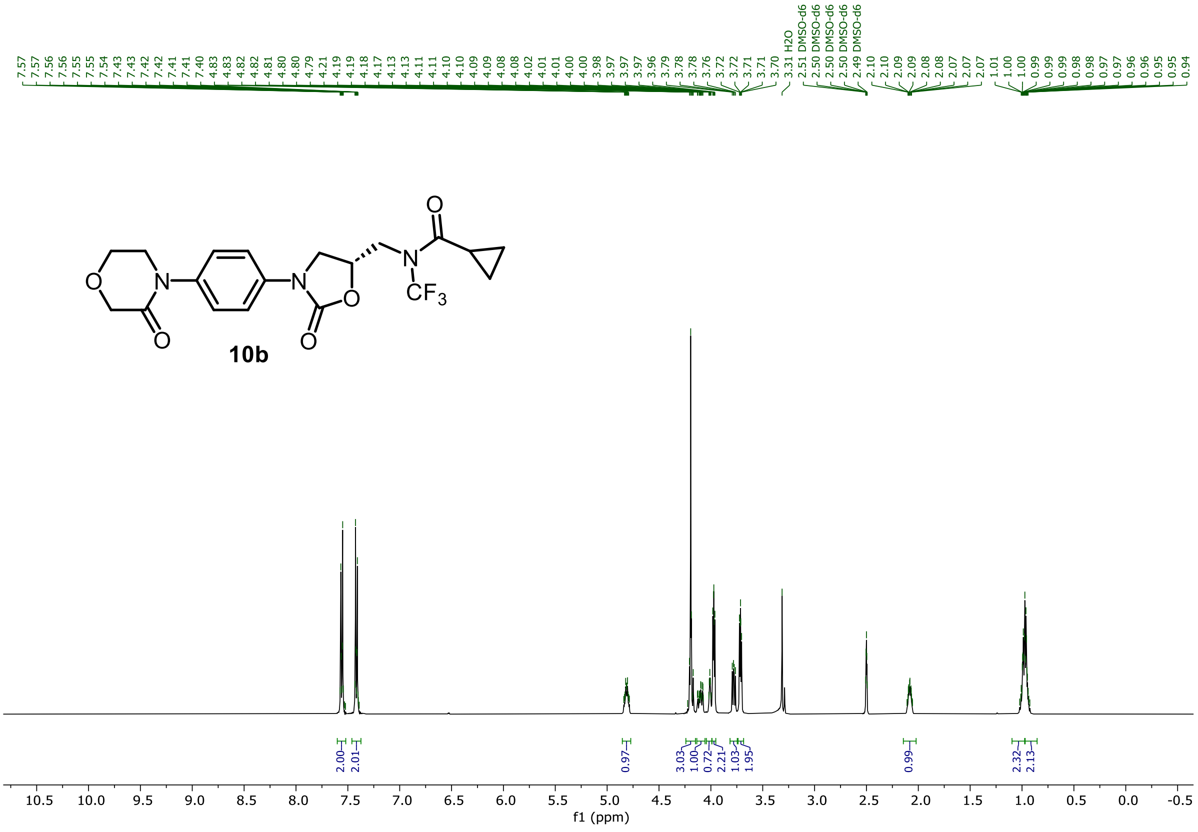

^{1}HNMR spectrum of **10b** run in DMSO-d_6 at 500 MHz.

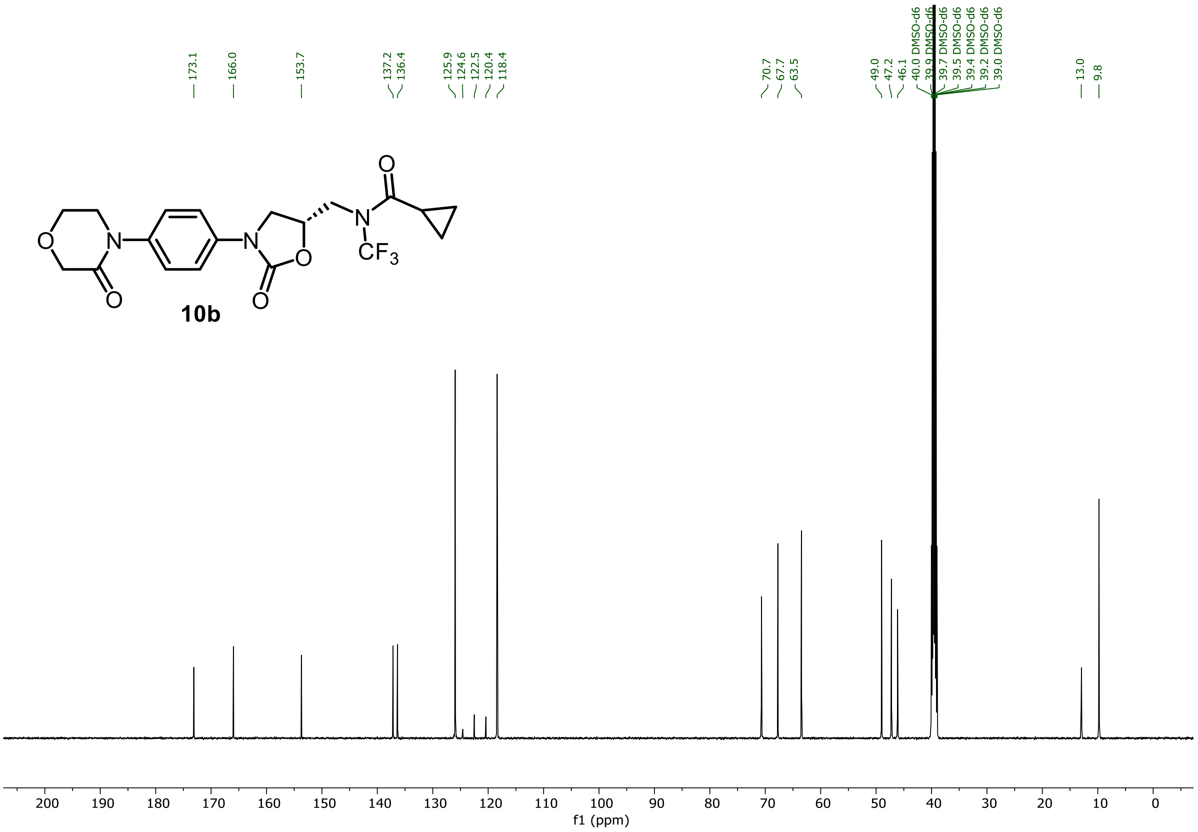

^{13}CNMR spectrum of **10b** run in DMSO-d_6 at 126 MHz.

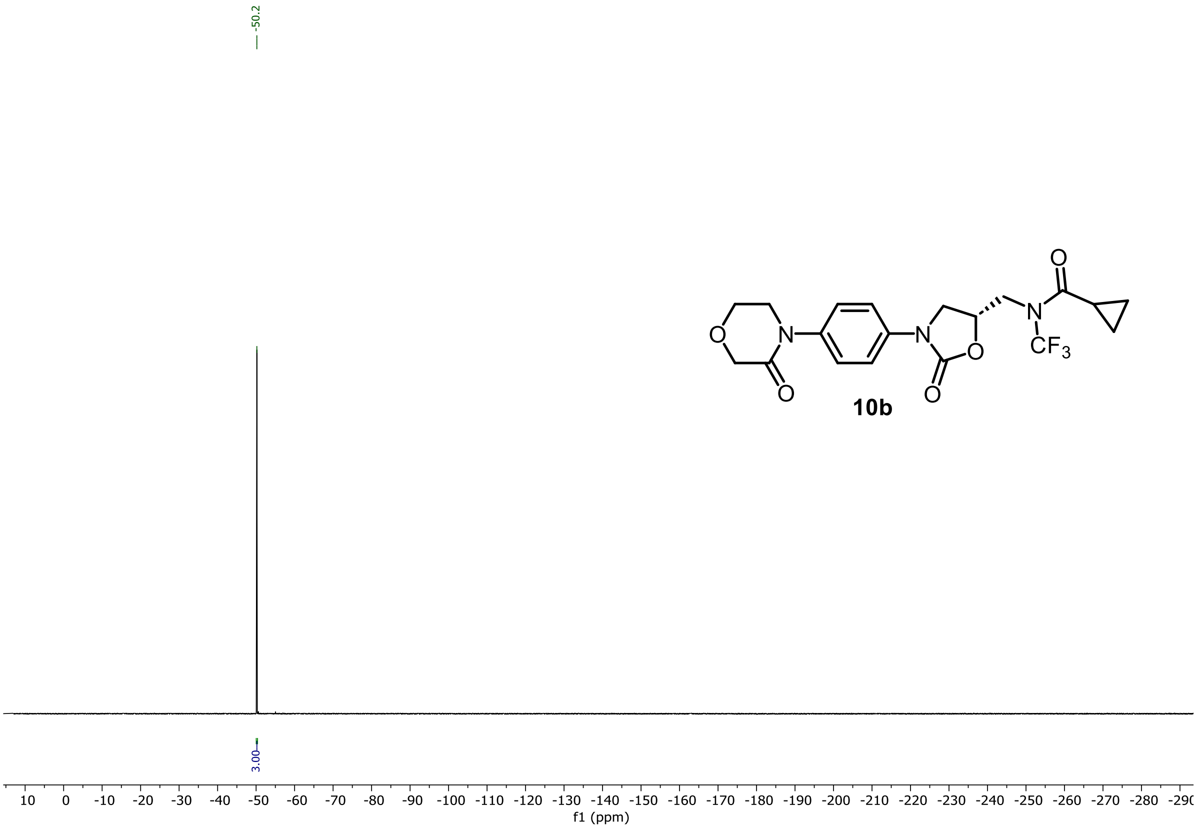

^{19}FNMR spectrum of **10b** run in DMSO-d_6 at 471 MHz.

(*R*)-*N*-((2-Oxo-3-(4-(3-oxomorpholino)phenyl)oxazolidin-5-yl)methyl)-2-phenyl-*N*-(trifluoro-methyl)acetamide (10c)

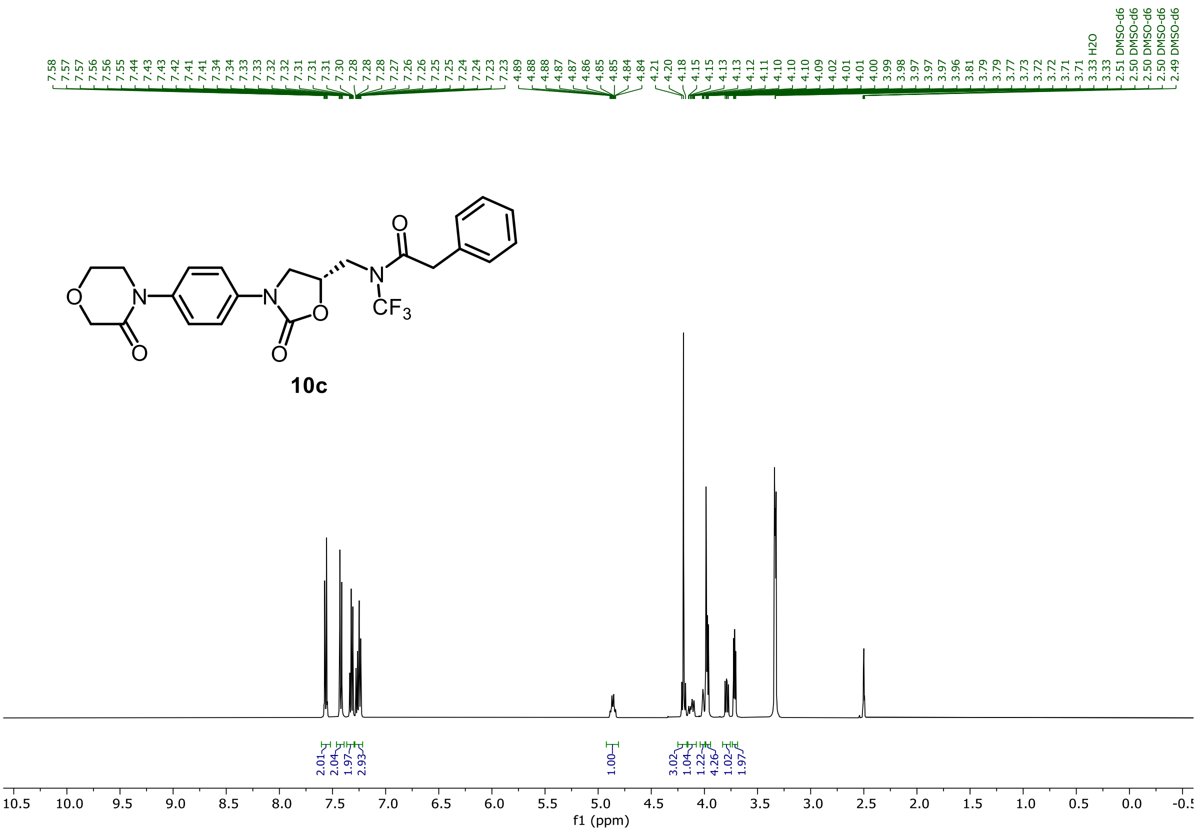

^{1}HNMR spectrum of **10c** run in DMSO-d_6 at 500 MHz.

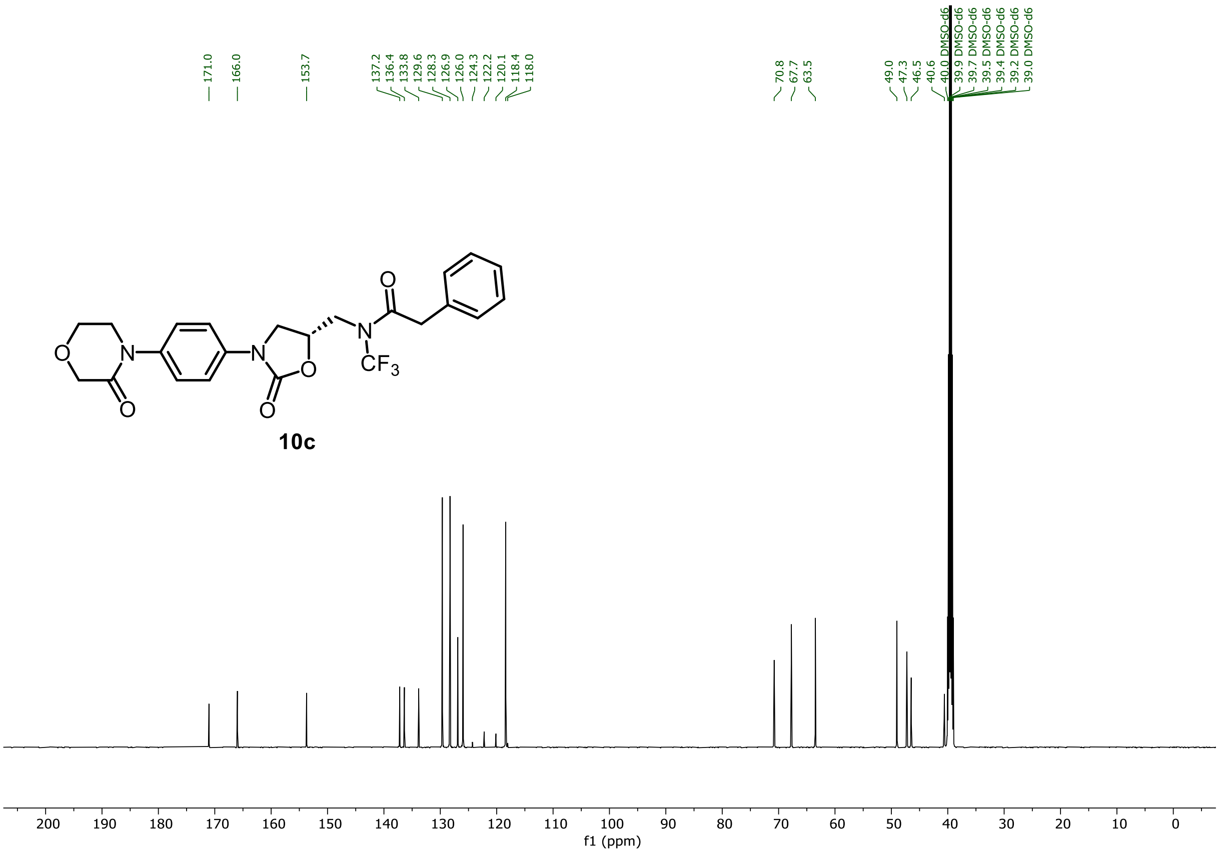

^{13}CNMR spectrum of **10c** run in DMSO-d_6 at 126 MHz.

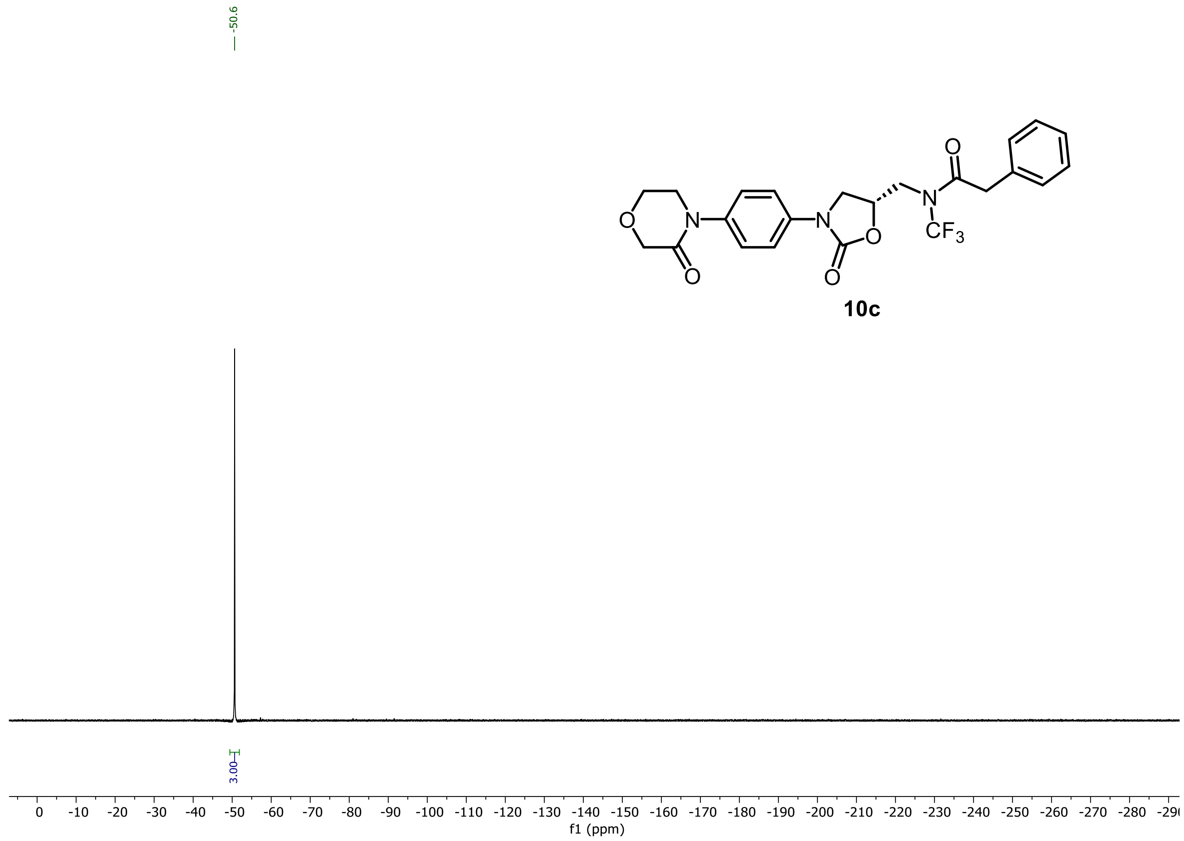

^{19}FNMR spectrum of **10c** run in DMSO-d_6 at 471 MHz.

(*R*)-*N*-((2-Oxo-3-(4-(3-oxomorpholino)phenyl)oxazolidin-5-yl)methyl)-*N*-trifluoromethyl benzamide (10d)

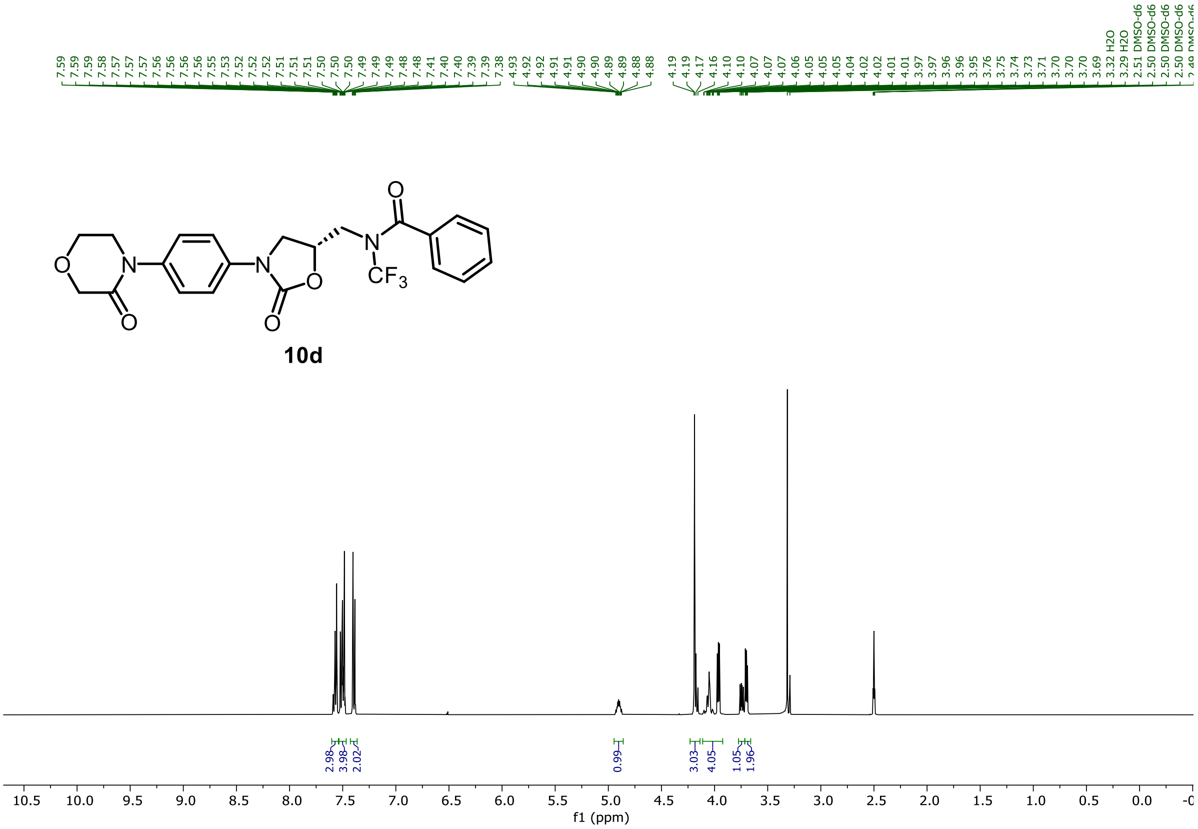

^{1}HNMR spectrum of **10d** run in DMSO-d_6 at 500 MHz.

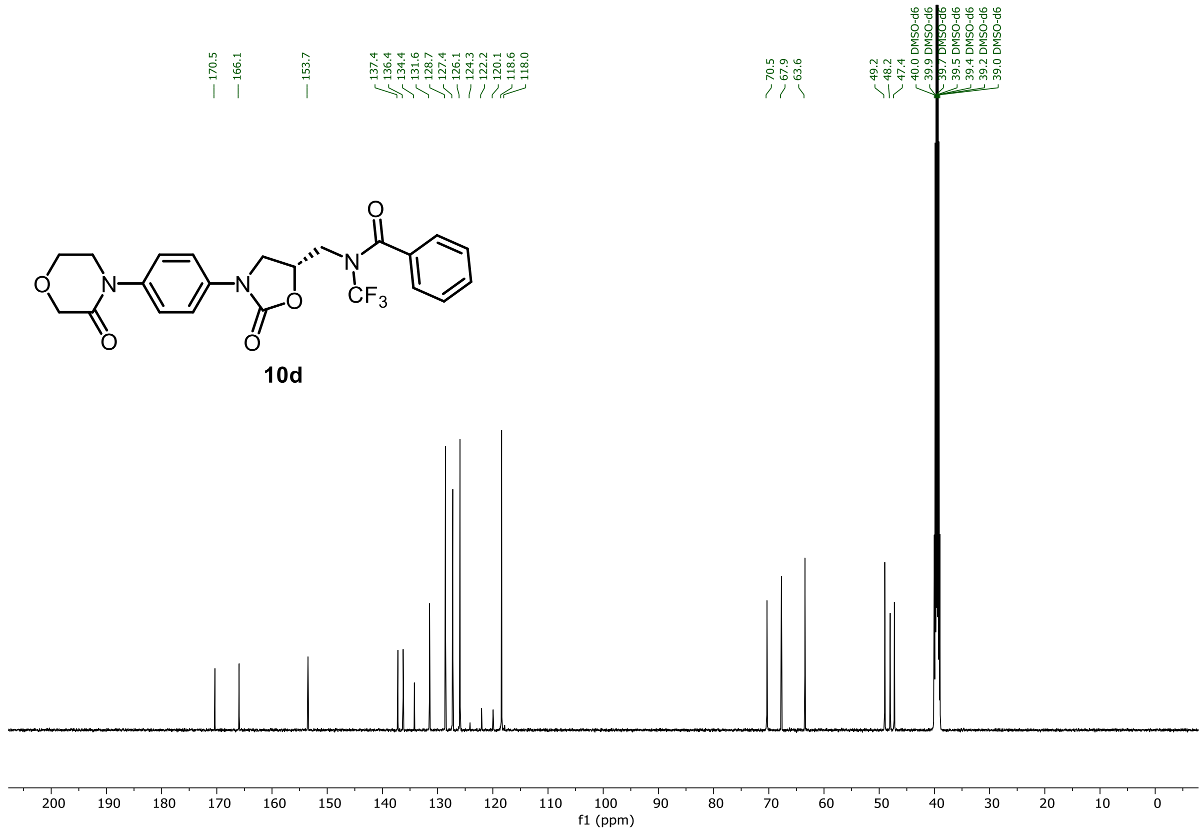

^{13}CNMR spectrum of **10d** run in DMSO-d_6 at 126 MHz.

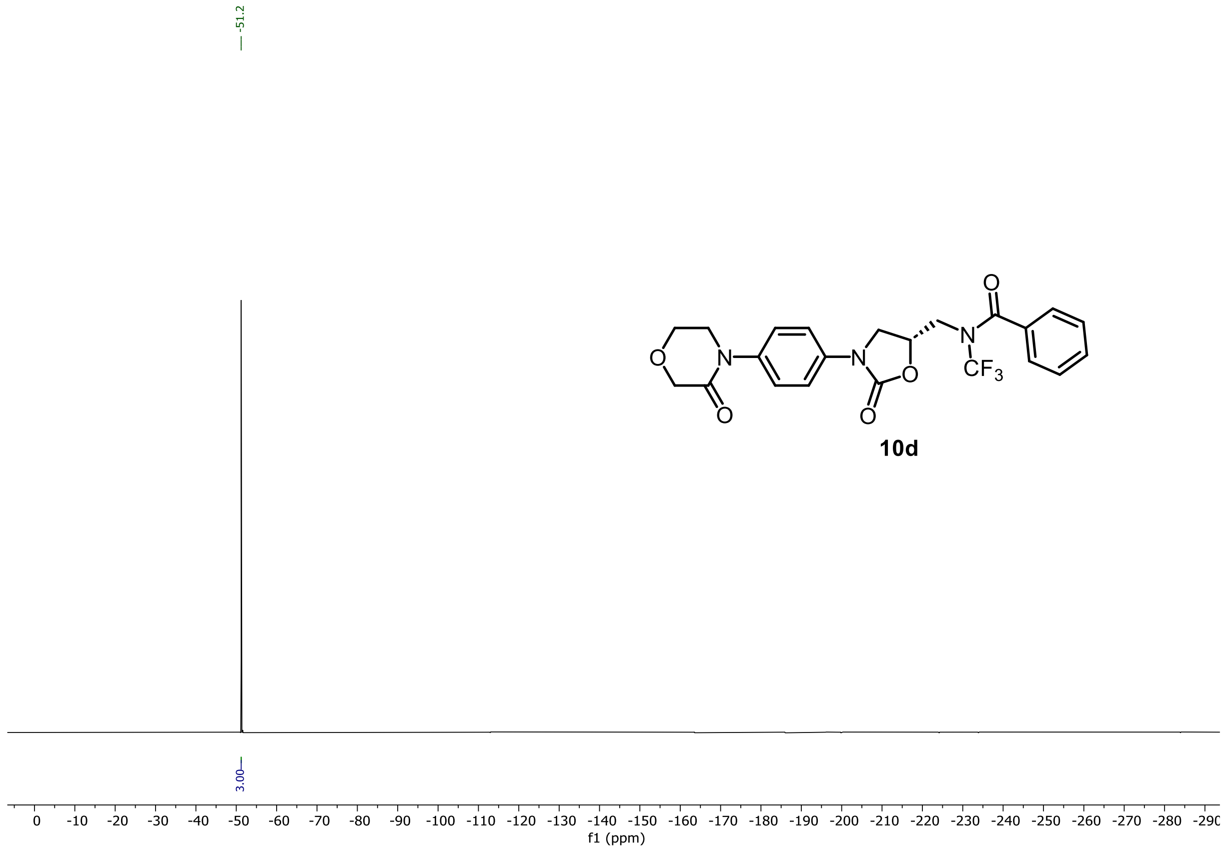

^{19}FNMR spectrum of **10d** run in DMSO-d_6 at 471 MHz.

N-(4-Hydroxyphenyl)-*N*-(trifluoromethyl)acetamide (11a)

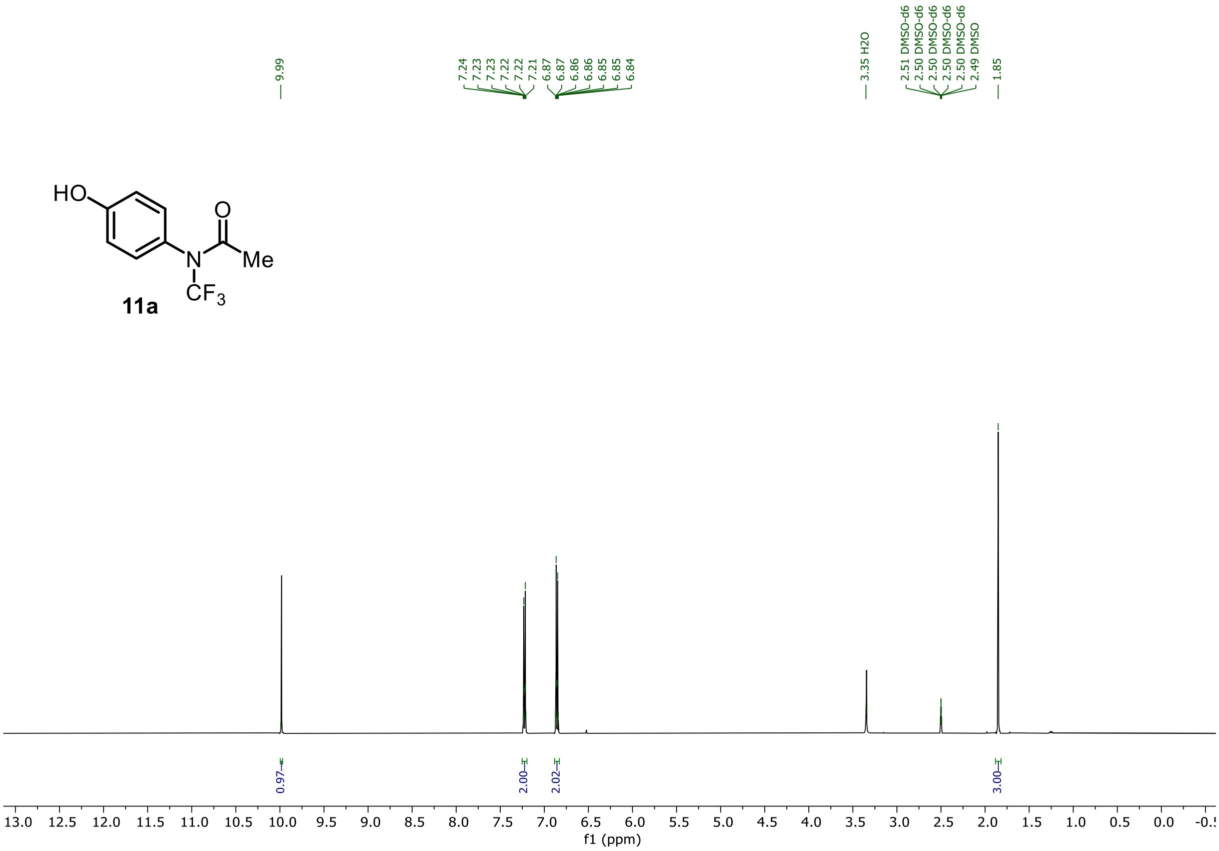

^{1}HNMR spectrum of **11a** run in DMSO-d_6 at 500 MHz.

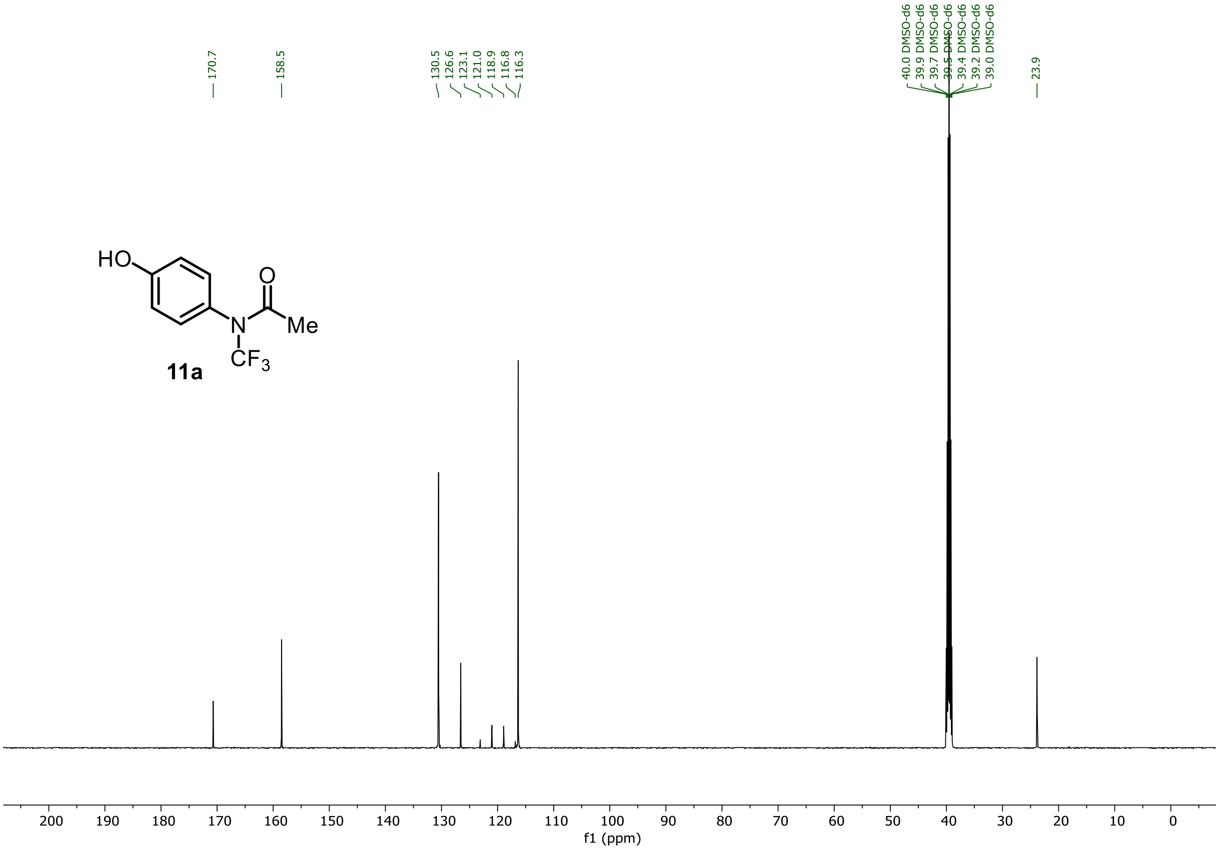

^{13}CNMR spectrum of **11a** run in DMSO-d_6 at 126 MHz.

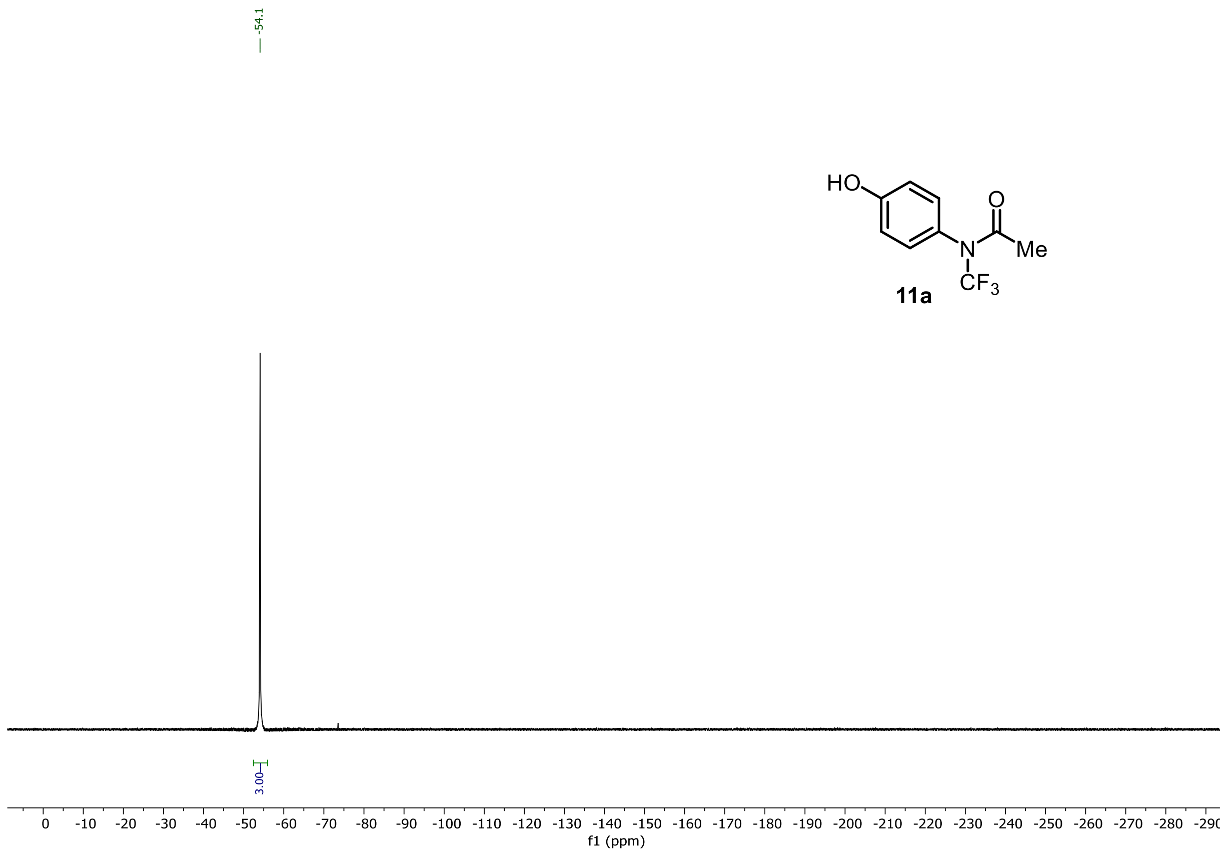

^{19}FNMR spectrum of **11a** run in DMSO-d_6 at 471 MHz.

N-(4-Hydroxyphenyl)-*N*-(trifluoromethyl)cyclopropanecarboxamide (11b)

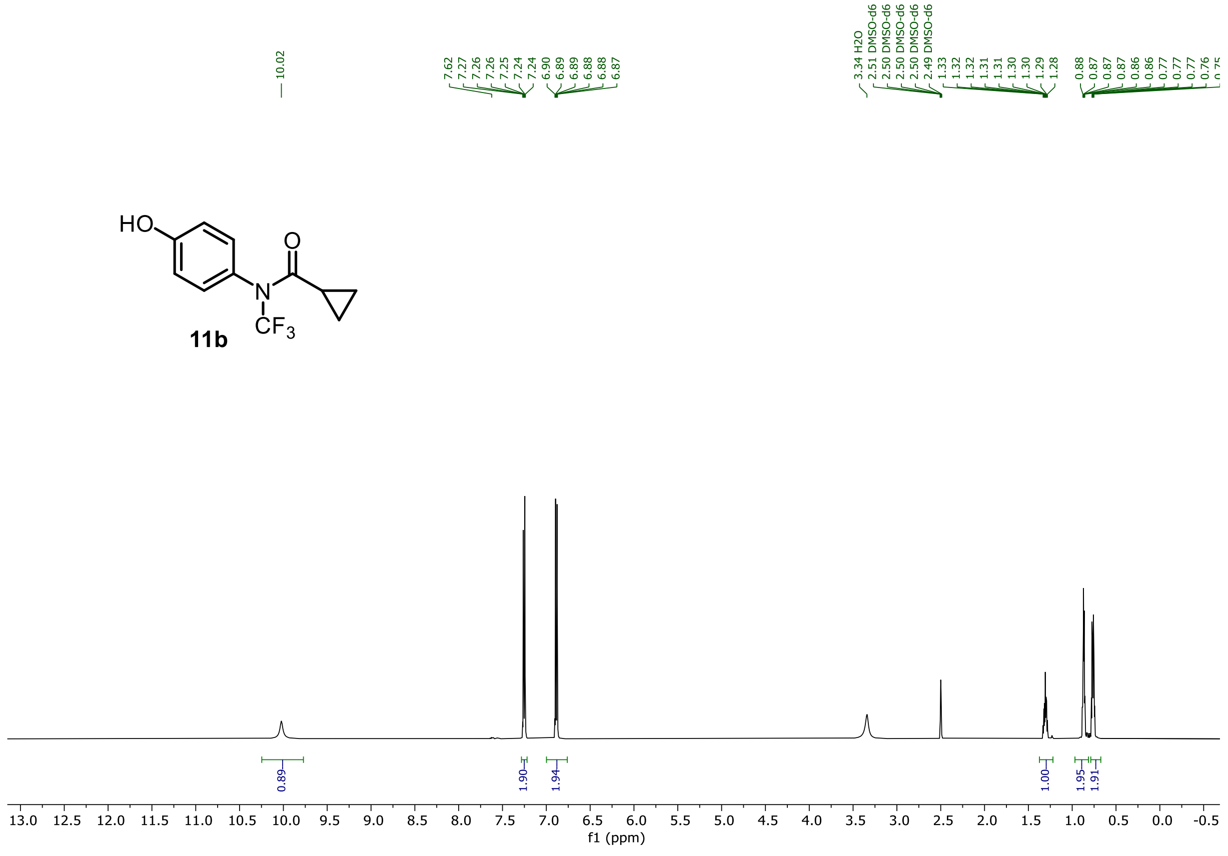

^{1}HNMR spectrum of **11b** run in DMSO-d_6 at 500 MHz.

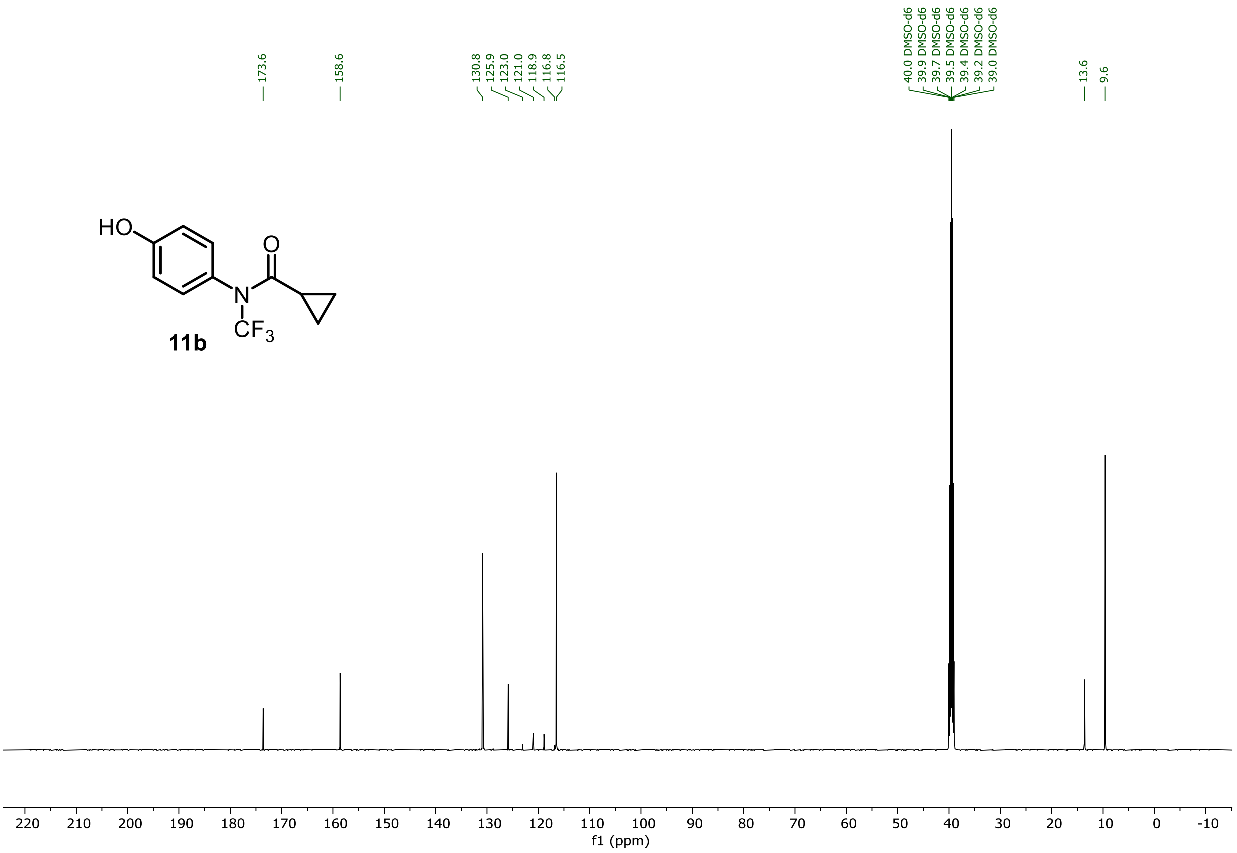

^{13}CNMR spectrum of **11b** run in DMSO-d_6 at 126 MHz.

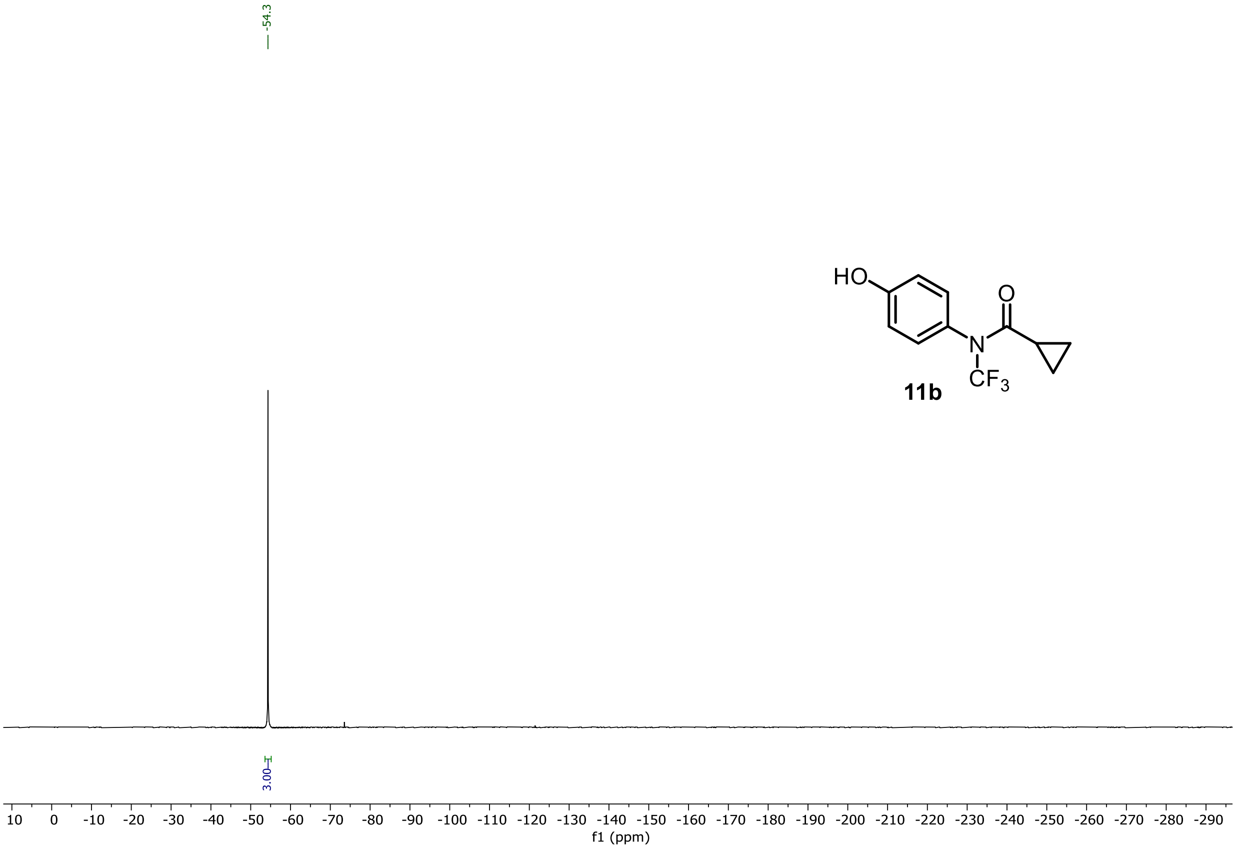

^{19}FNMR spectrum of **11b** run in DMSO-d_6 at 471 MHz.

N-(4-Hydroxyphenyl)-2-phenyl-*N*-(trifluoromethyl)acetamide (11c)

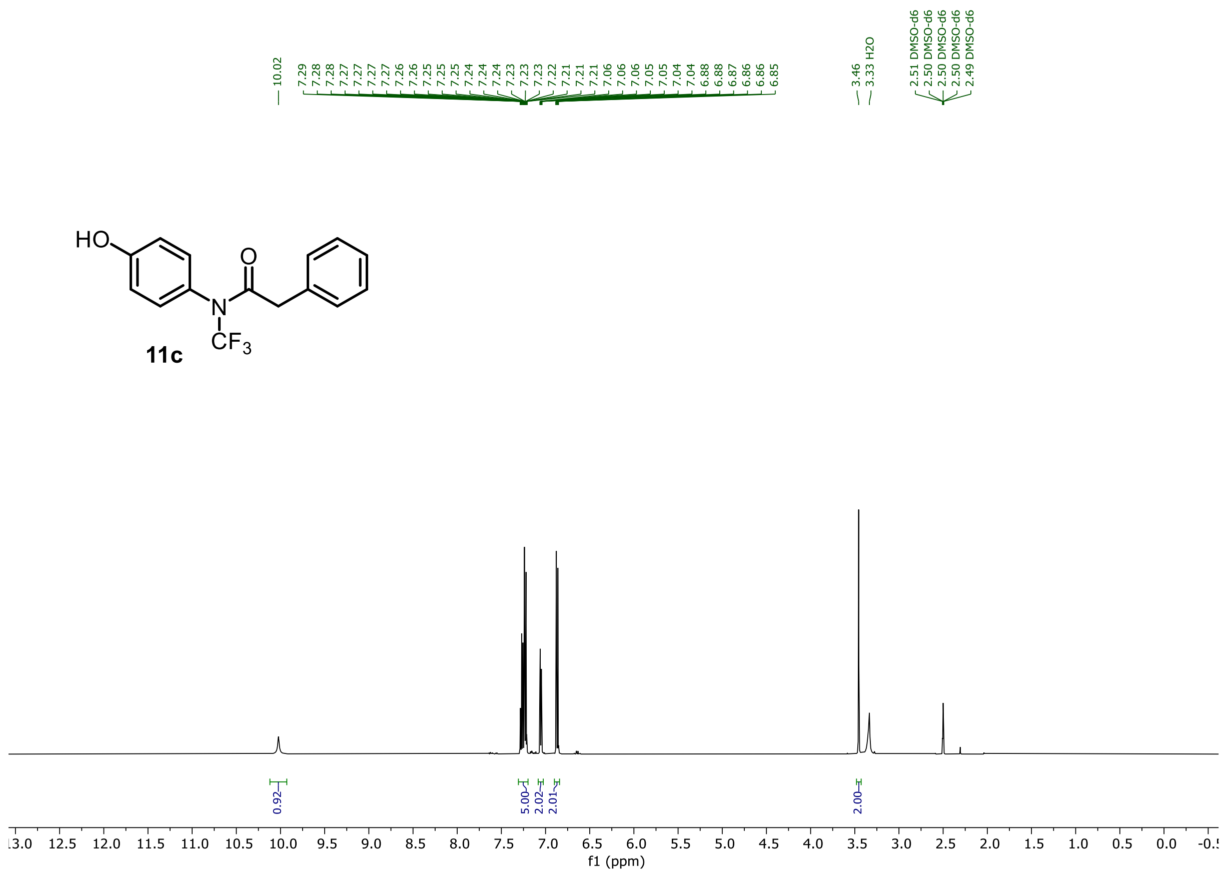

^{1}HNMR spectrum of **11c** run in DMSO-d_6 at 500 MHz.

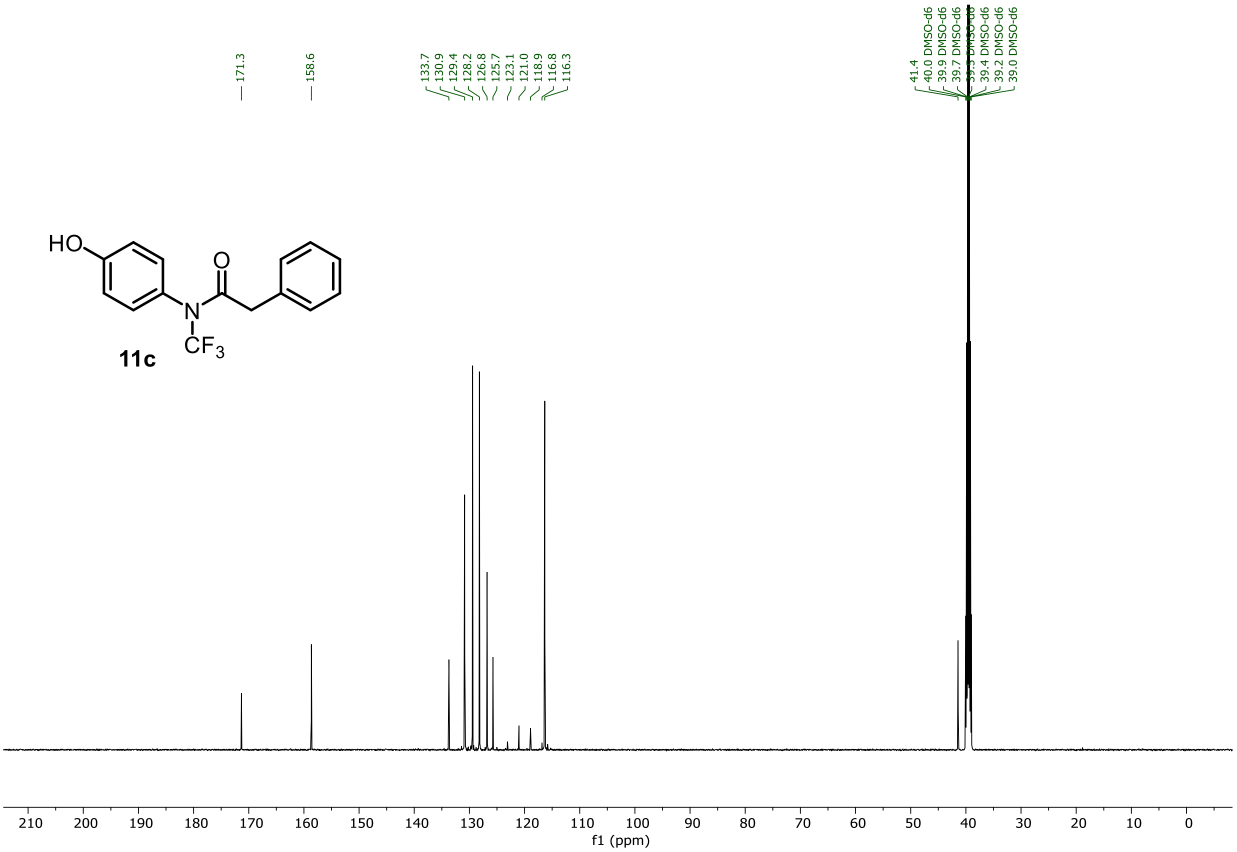

^{13}CNMR spectrum of **11c** run in DMSO-d_6 at 126 MHz.

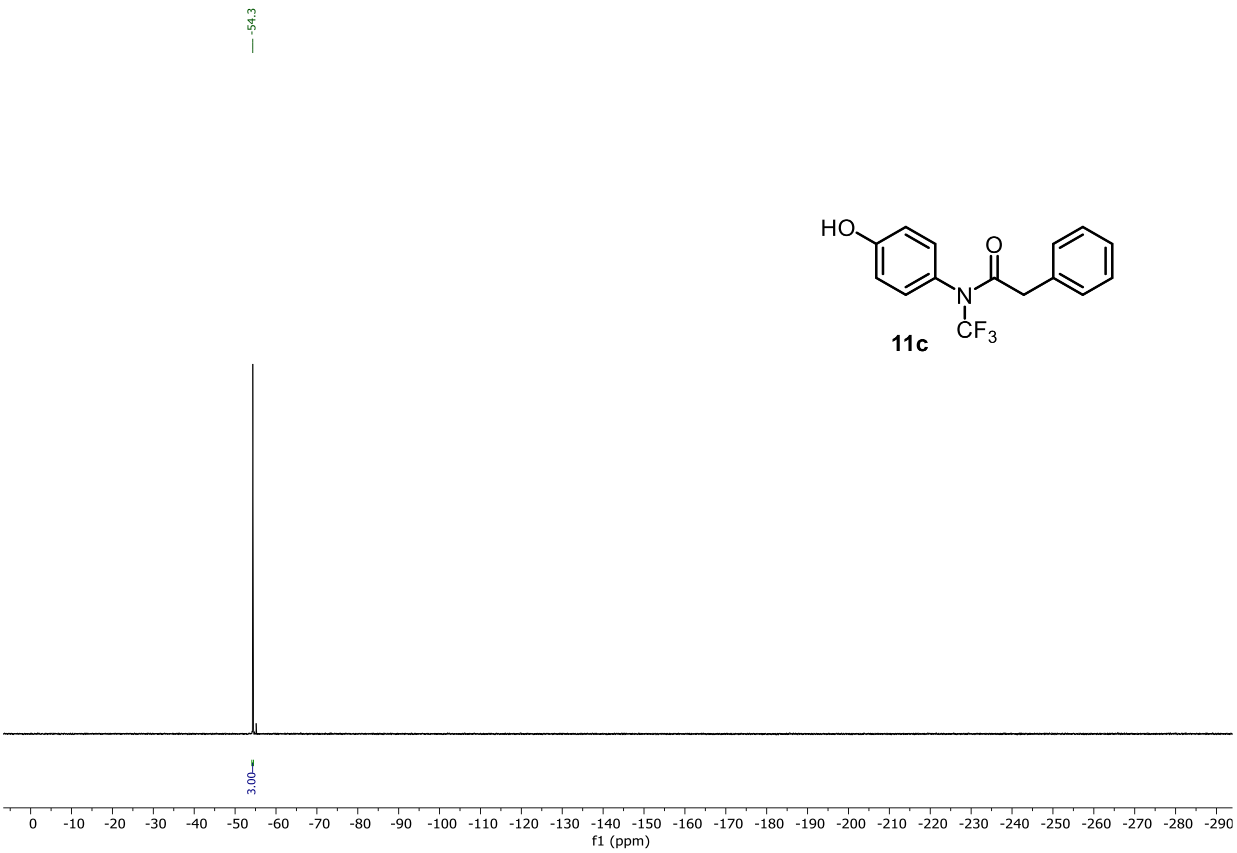

^{19}FNMR spectrum of **11c** run in DMSO-d_6 at 471 MHz.

N-(4-Hydroxyphenyl)-*N*-(trifluoromethyl)benzamide (11d)

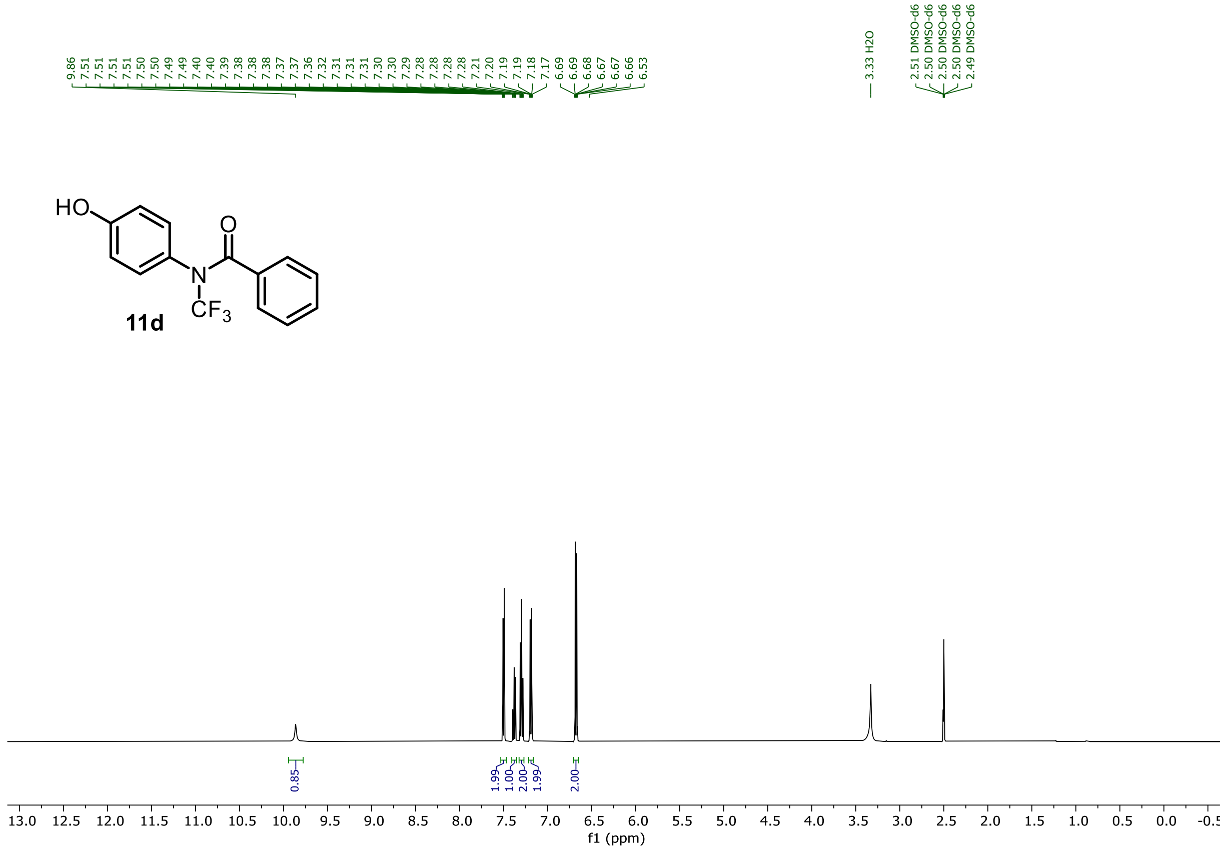

^{1}HNMR spectrum of **11d** run in DMSO-d_6 at 500 MHz.

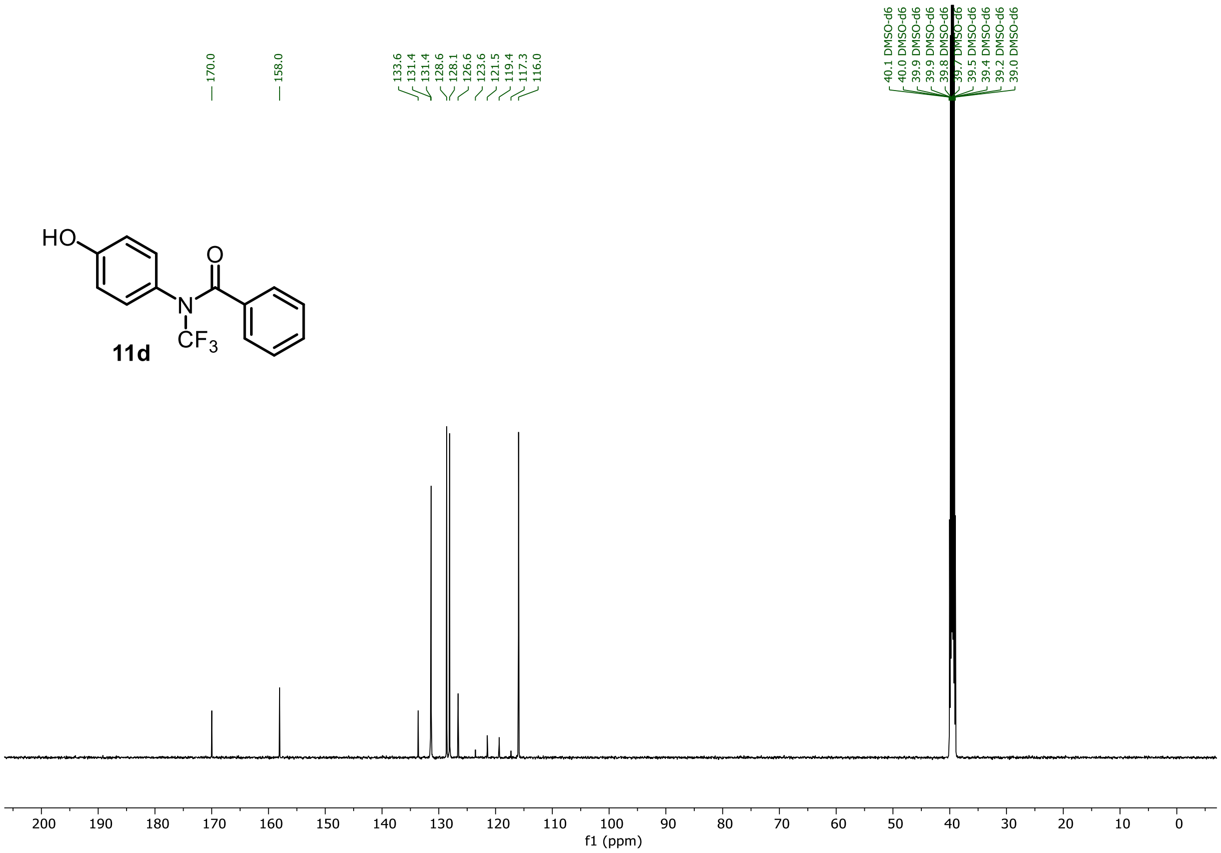

^{13}CNMR spectrum of **11d** run in DMSO-d_6 at 126 MHz.

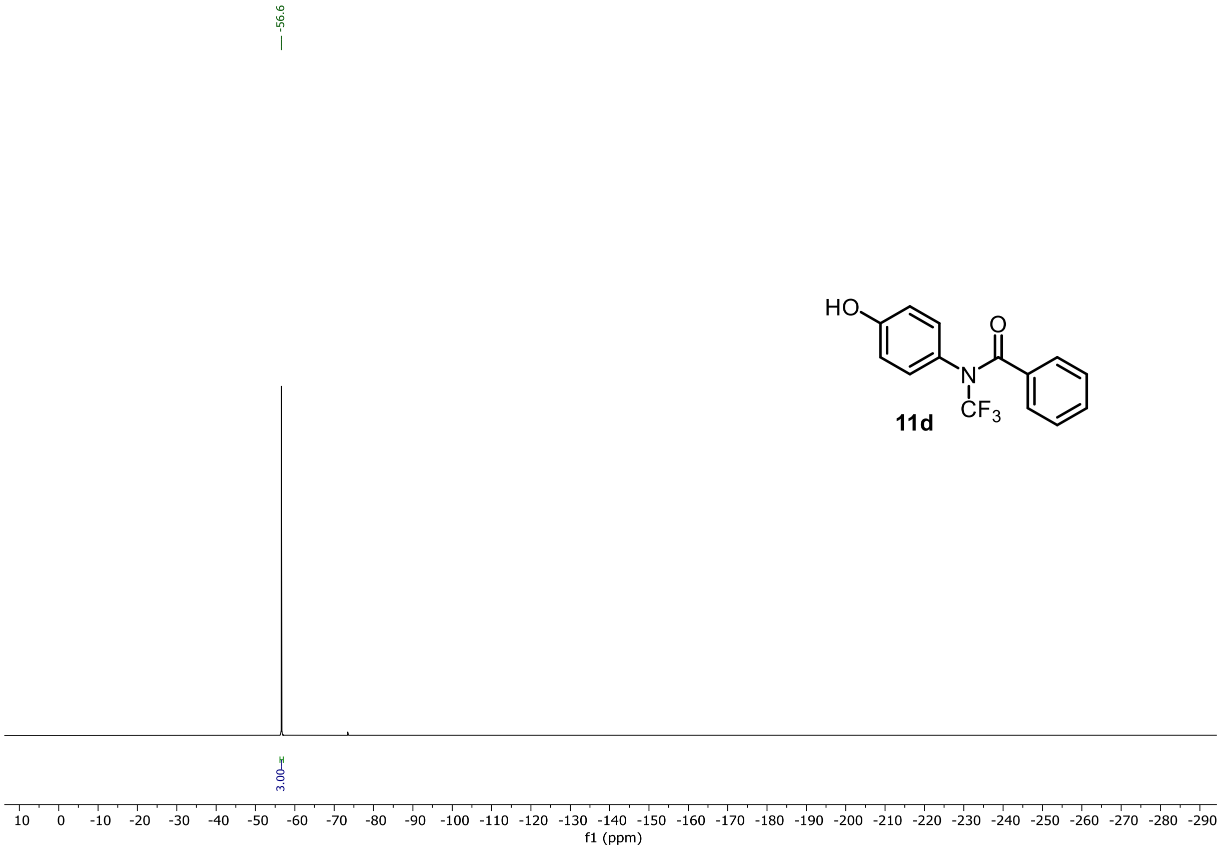

^{19}FNMR spectrum of **11d** run in DMSO-d_6 at 471 MHz.

3.4 *N*-CF_3 ureas and carbamates

3-Hydroxy-*N*-(4-methoxybenzyl)-*N*-(trifluoromethyl)azetidine-1-carboxamide (5c)

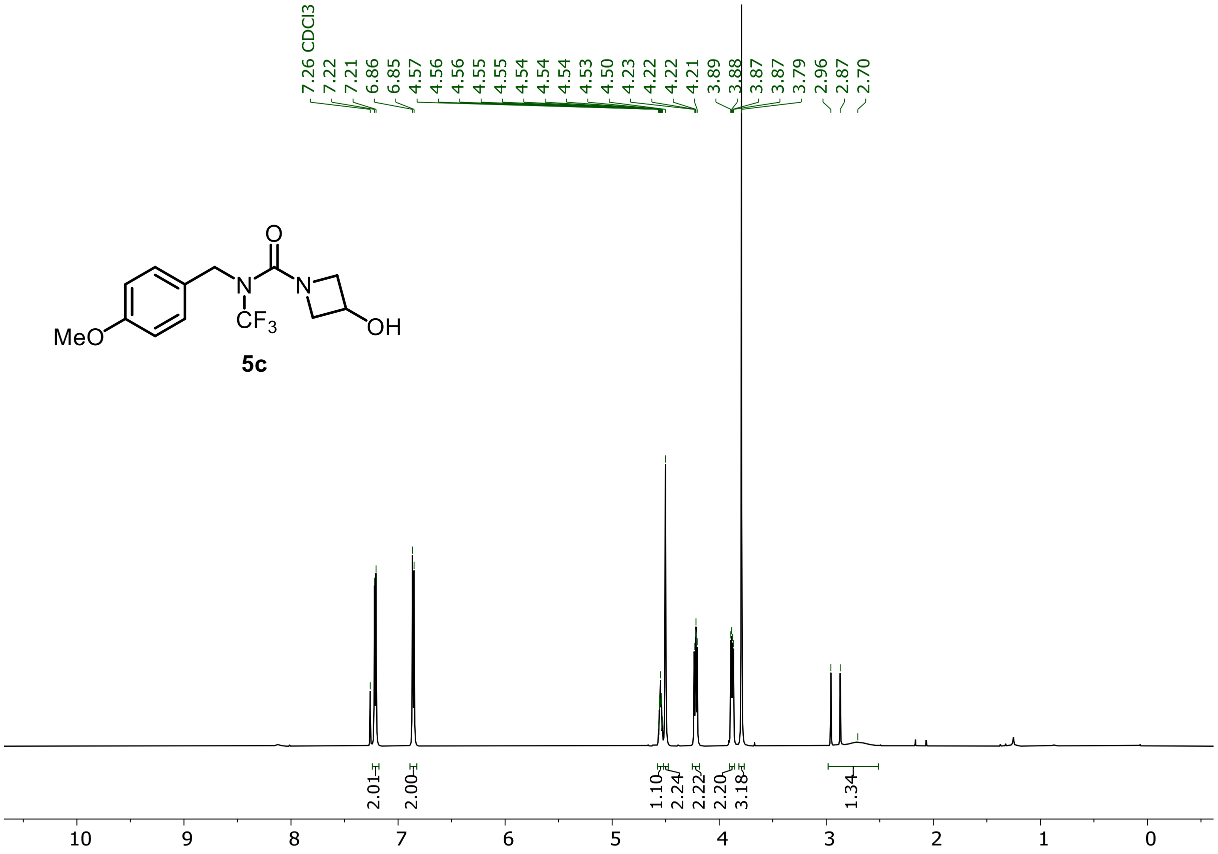

^{1}HNMR spectrum of **5c** run in $CDCl_3$ at 600 MHz.

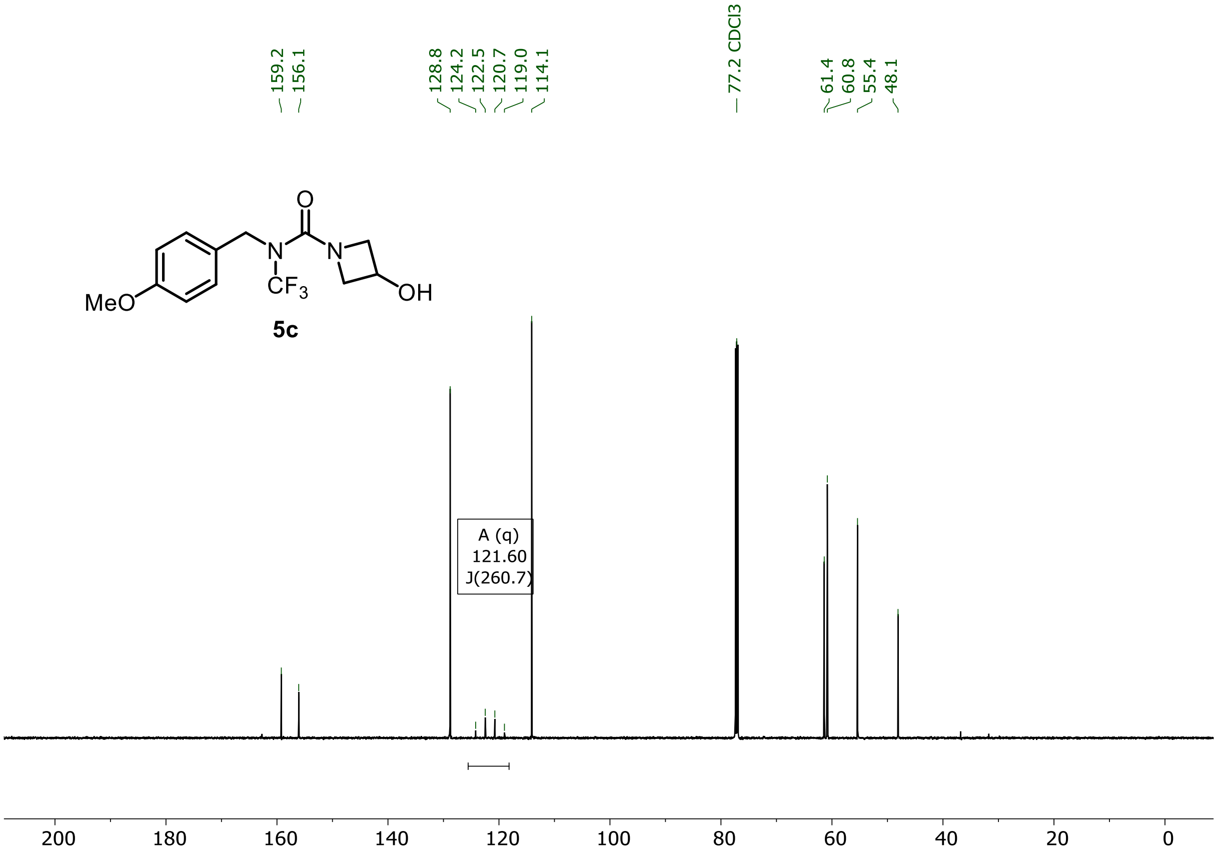

^{13}CNMR spectrum of **5c** run in $CDCl_3$ at 151 MHz.

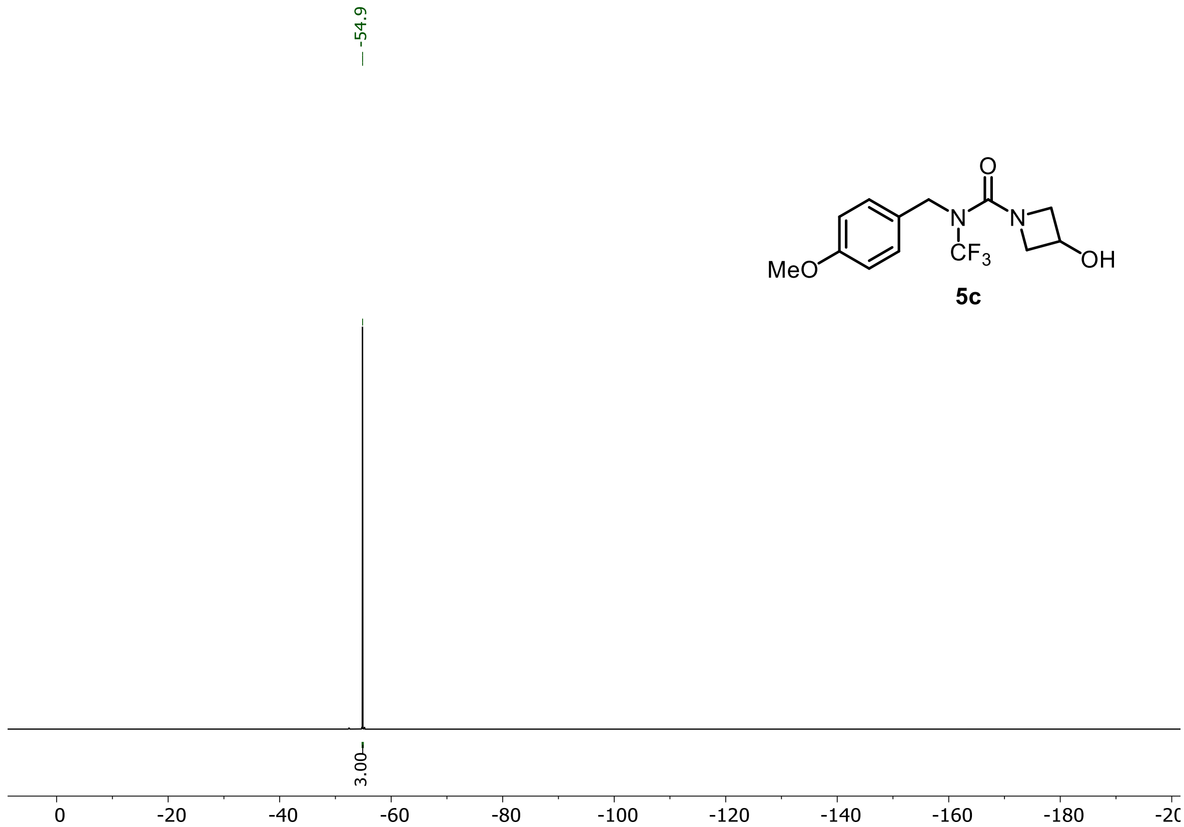

^{19}FNMR spectrum of **5c** run in $CDCl_3$ at 565 MHz.

3-Hydroxy-*N*-(4-methoxybenzyl)-*N*-(trifluoromethyl)piperidine-1-carboxamide (6c)

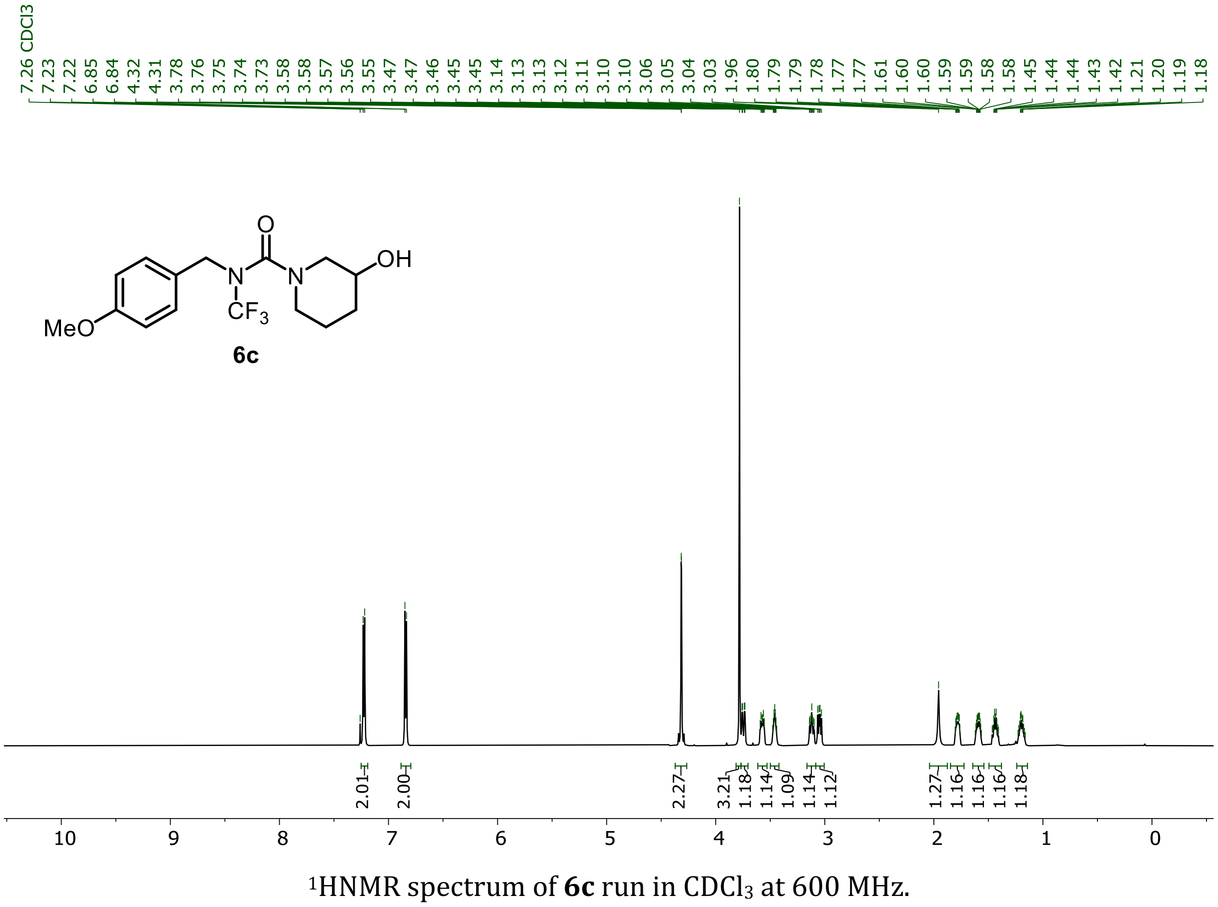

^{1}HNMR spectrum of **6c** run in $CDCl_3$ at 600 MHz.

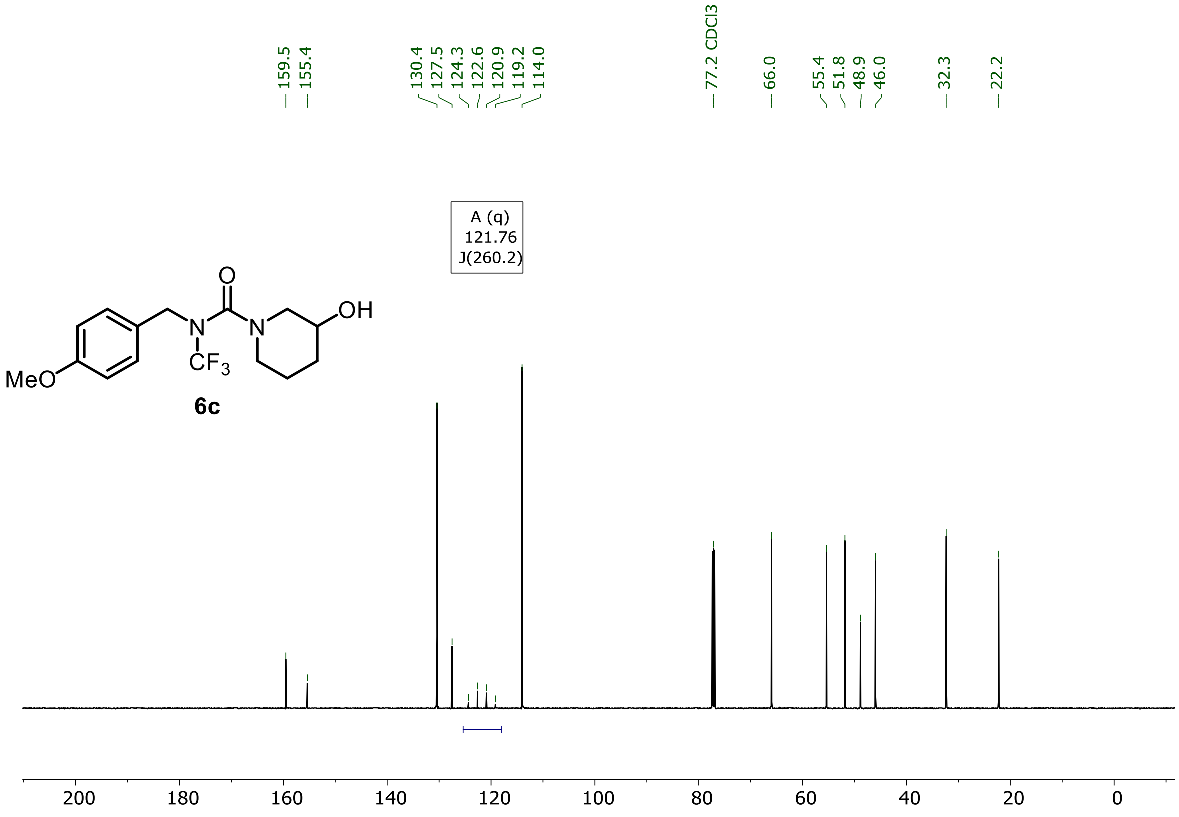

^{13}CNMR spectrum of **6c** run in $CDCl_3$ at 151 MHz.

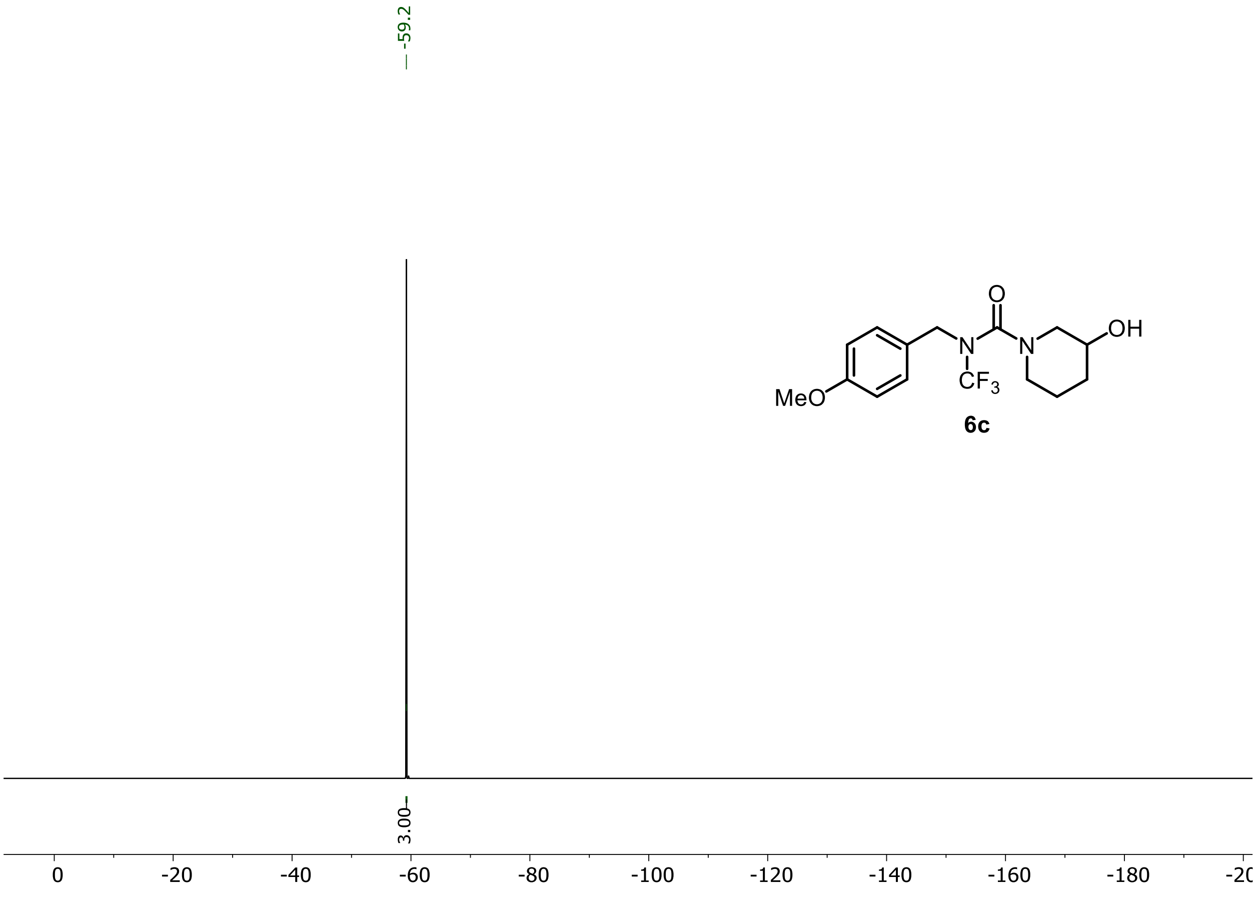

^{19}FNMR spectrum of **6c** run in $CDCl_3$ at 565 MHz.

_N_-(4-Methoxybenzyl)-4-methyl-3-oxo-_N_-(trifluoromethyl)piperazine-1-carboxamide (7c)

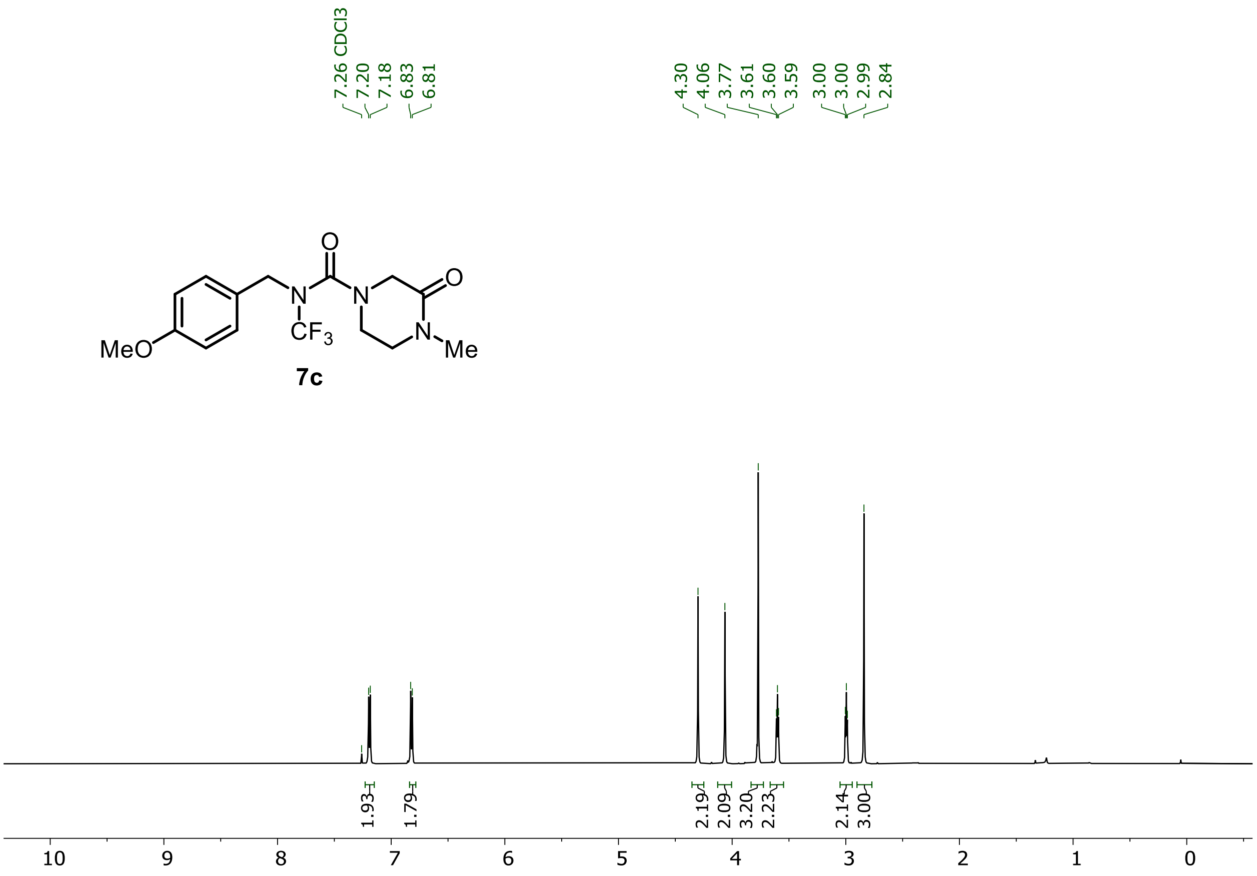

^{1}HNMR spectrum of **7c** run in $CDCl_3$ at 600 MHz.

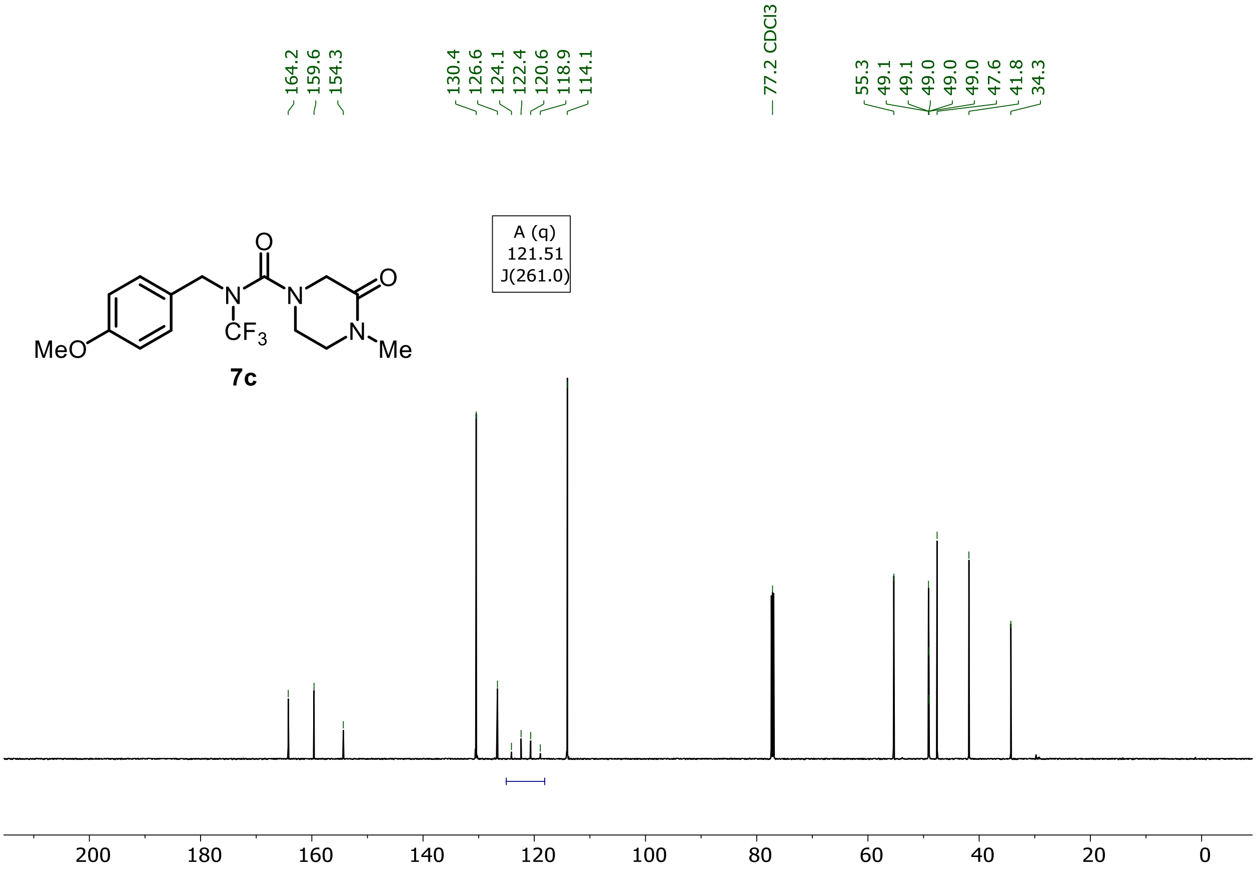

^{13}CNMR spectrum of **7c** run in $CDCl_3$ at 151 MHz.

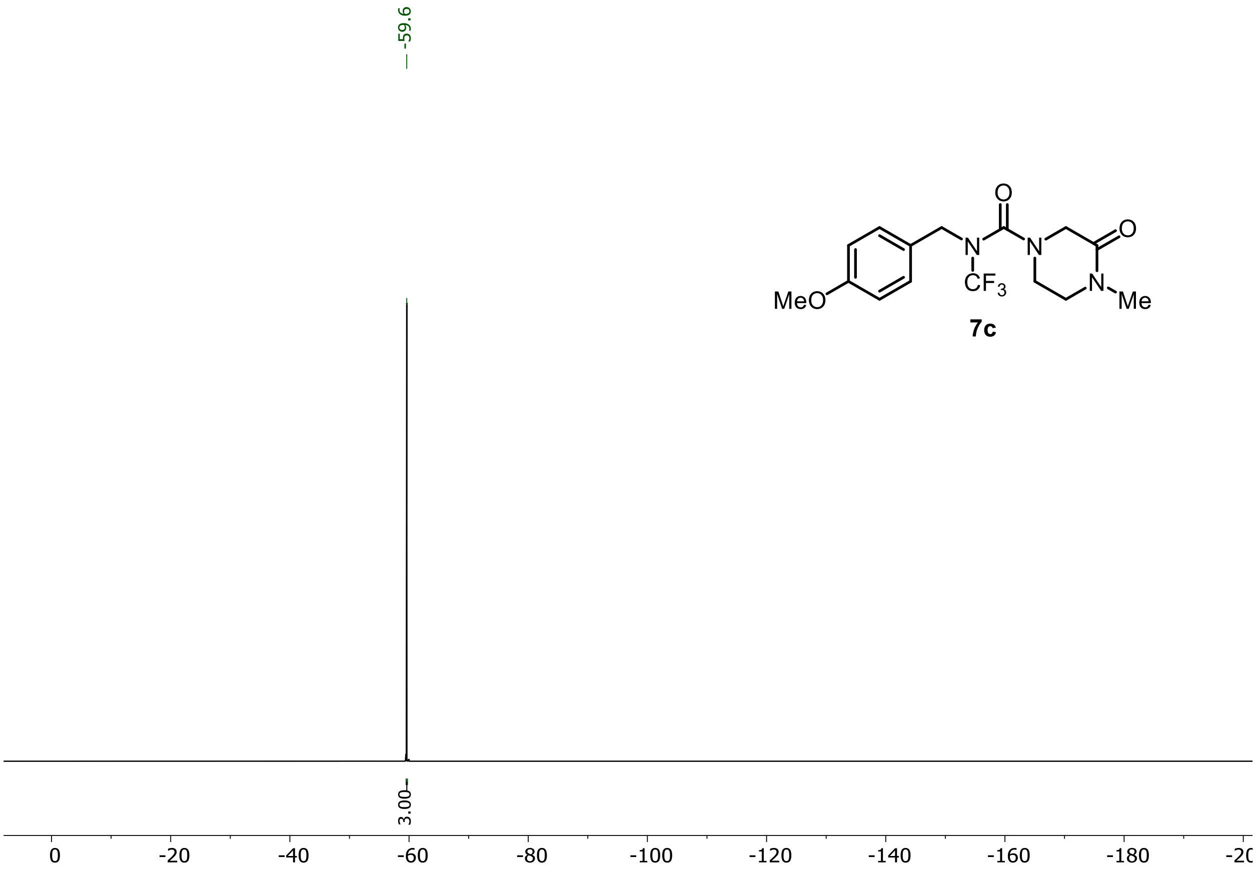

^{19}FNMR spectrum of **7c** run in $CDCl_3$ at 565 MHz.

3-(Cyanomethyl)-1-(4-methoxybenzyl)-1-(trifluoromethyl)urea (8c)

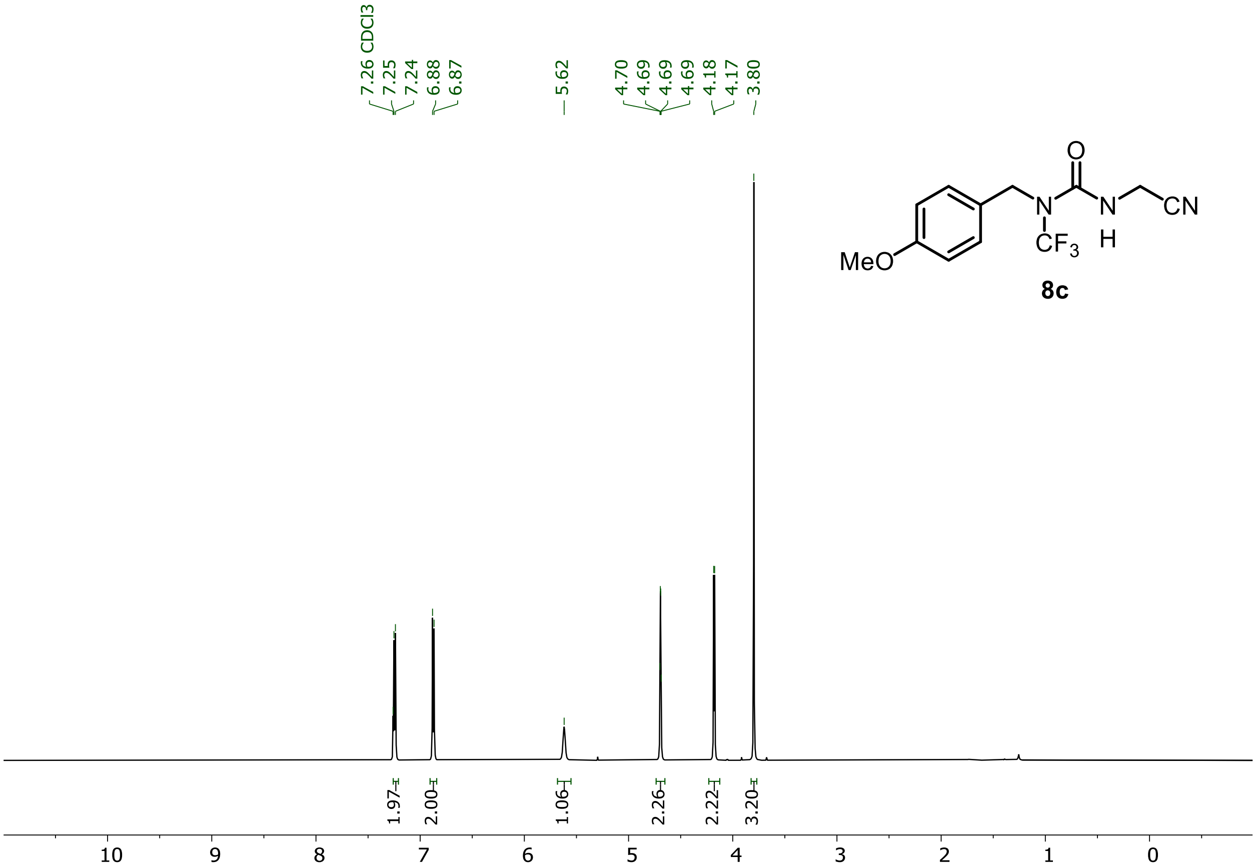

^{1}HNMR spectrum of **8c** run in $CDCl_3$ at 600 MHz.

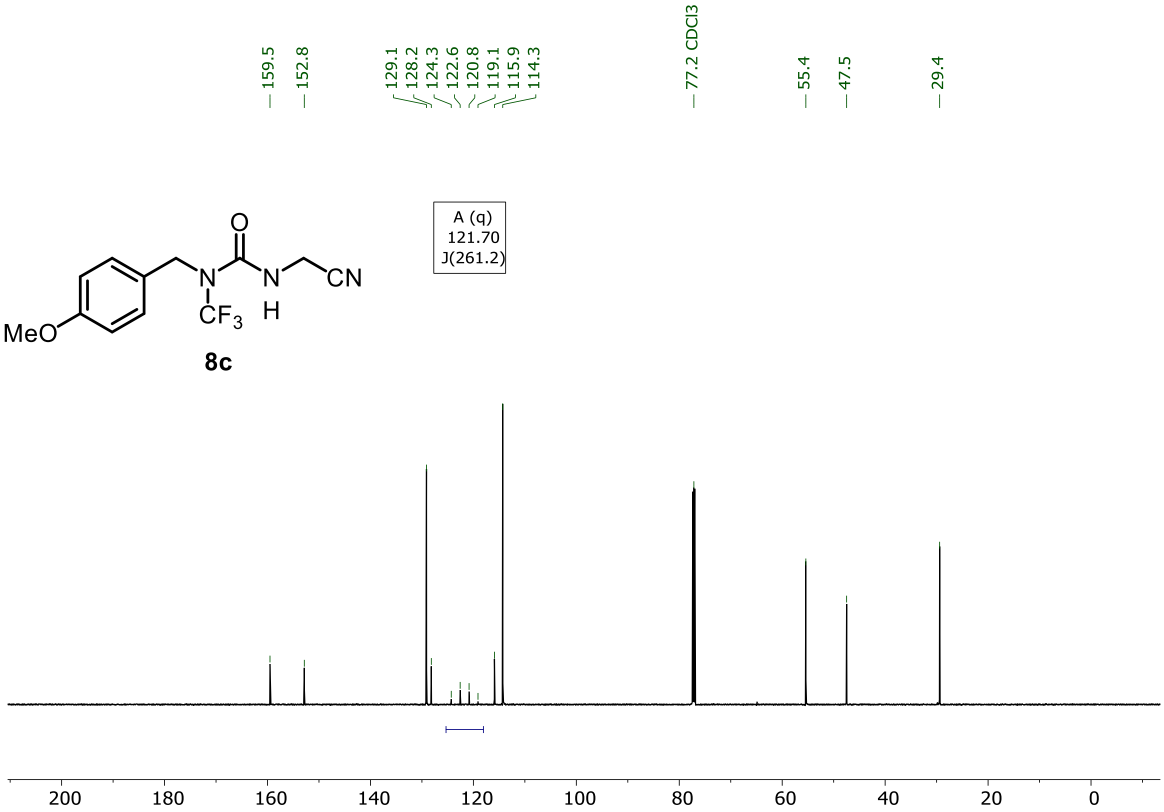

^{13}CNMR spectrum of **8c** run in $CDCl_3$ at 151 MHz.

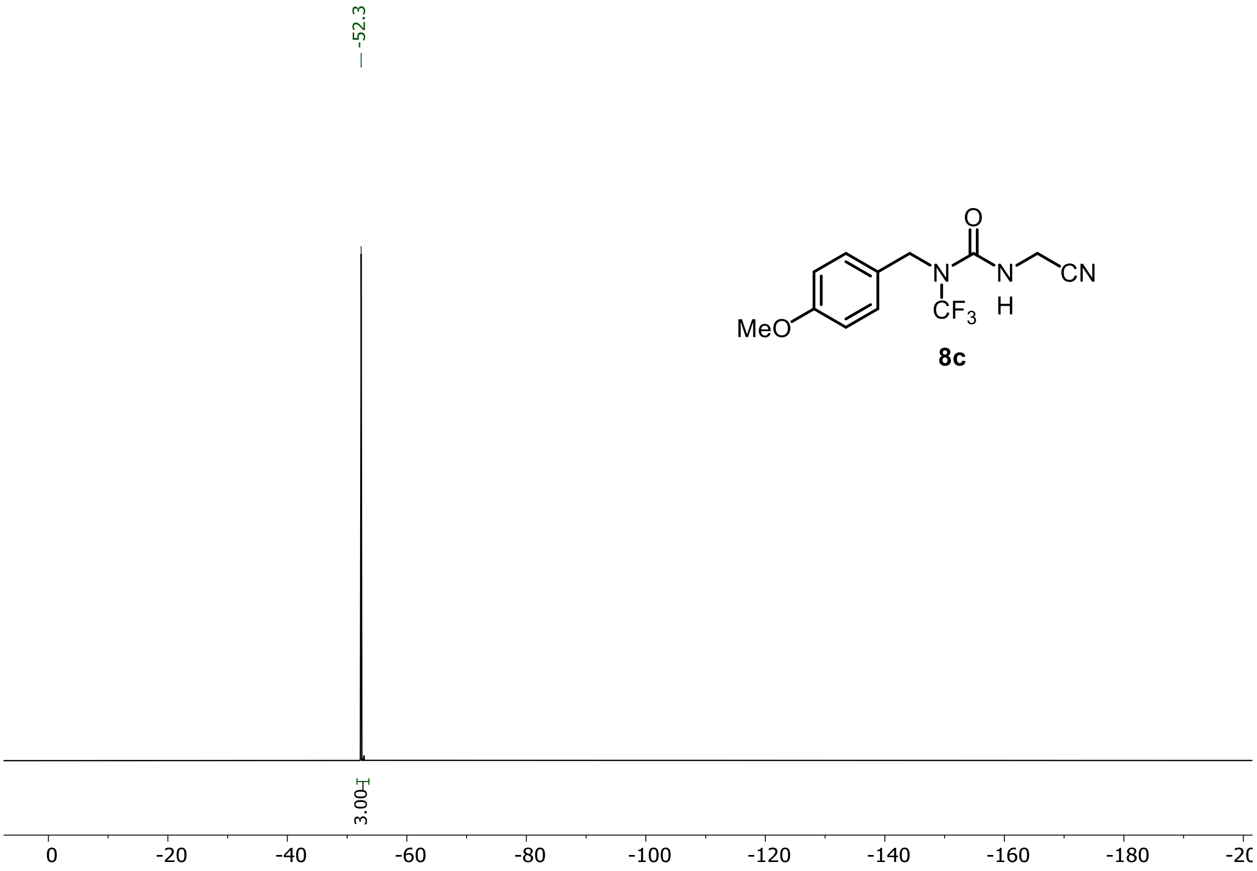

^{19}FNMR spectrum of **8c** run in $CDCl_3$ at 565 MHz.

Pyridin-3-ylmethyl (2-oxotetrahydrofuran-3-yl)(trifluoromethyl)carbamate (9b)

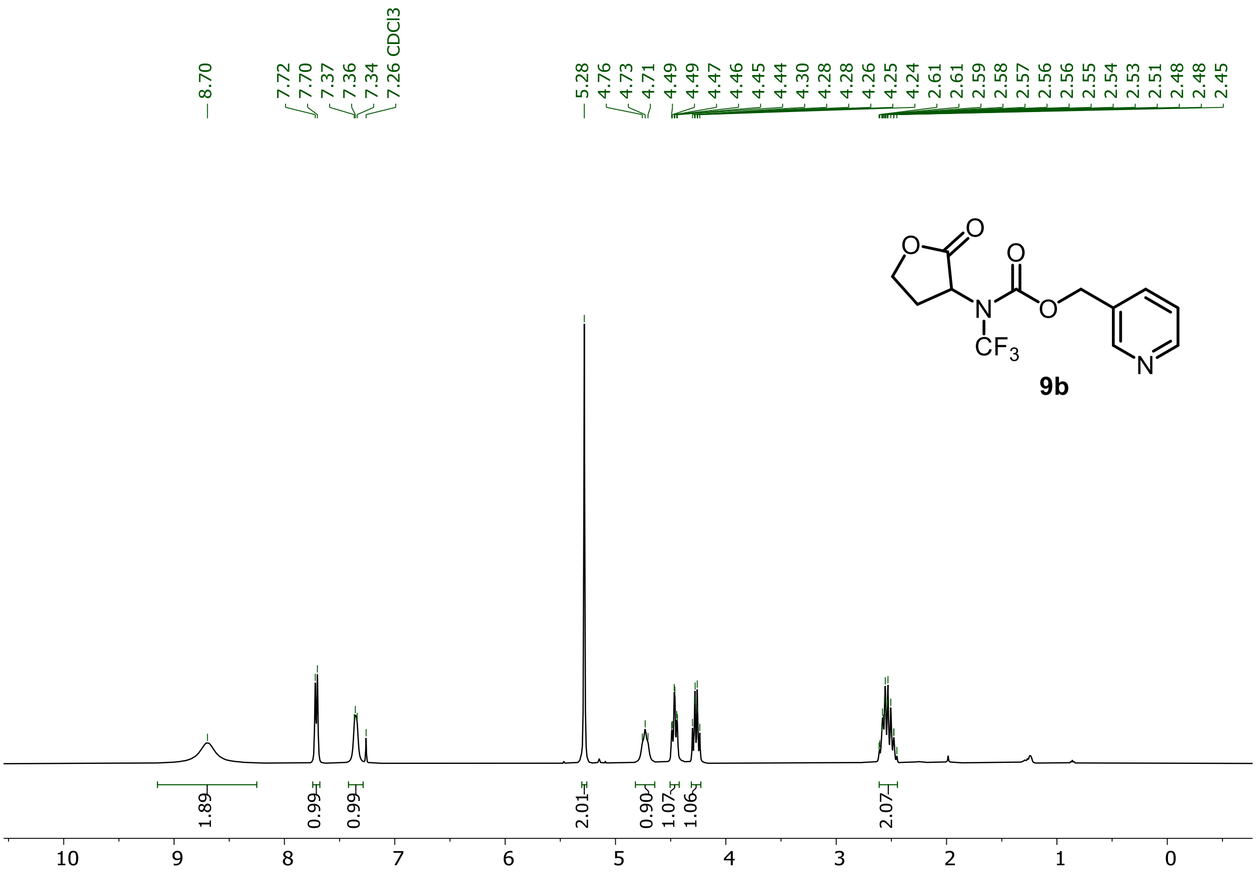

^{1}HNMR spectrum of **9b** run in $CDCl_3$ at 400 MHz.

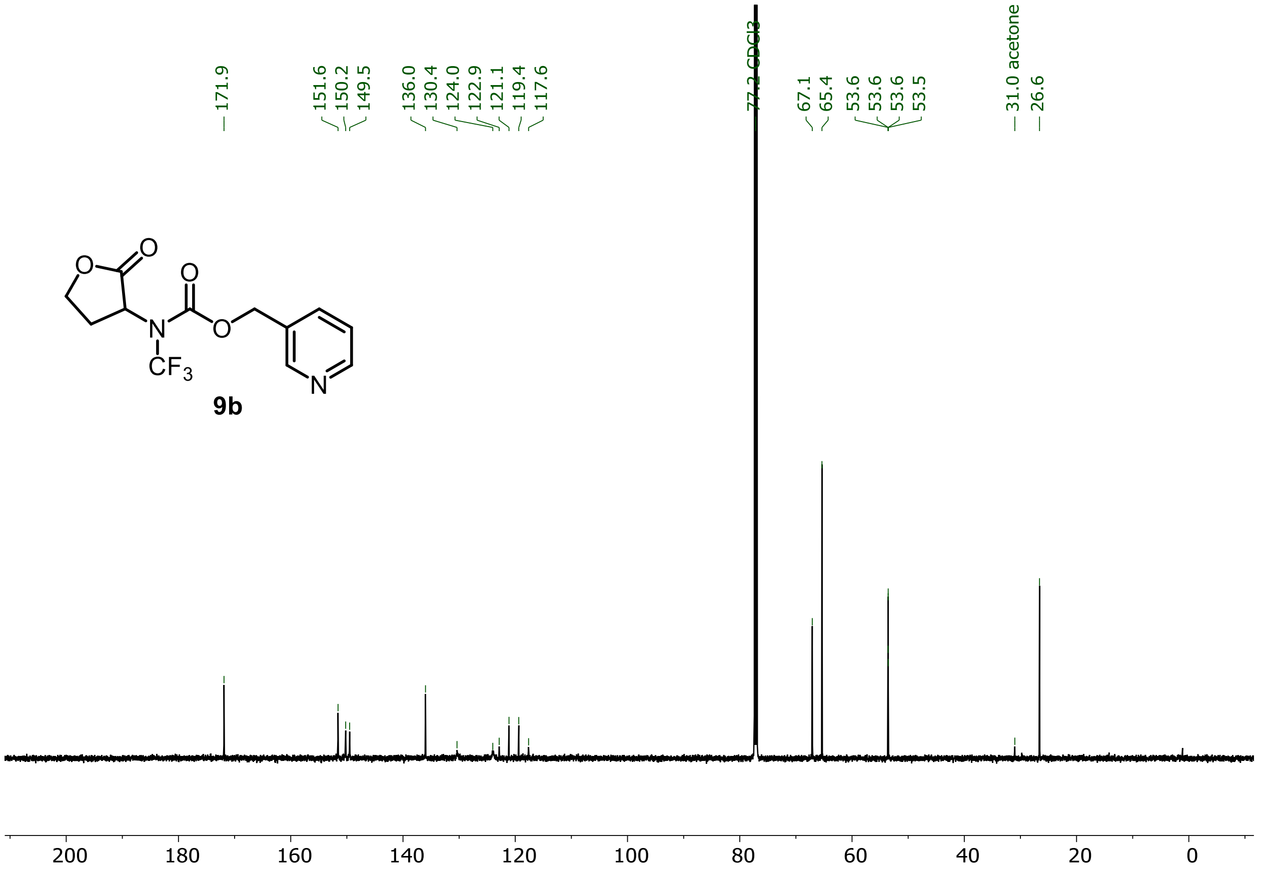

^{13}CNMR spectrum of **9b** run in $CDCl_3$ at 151 MHz.

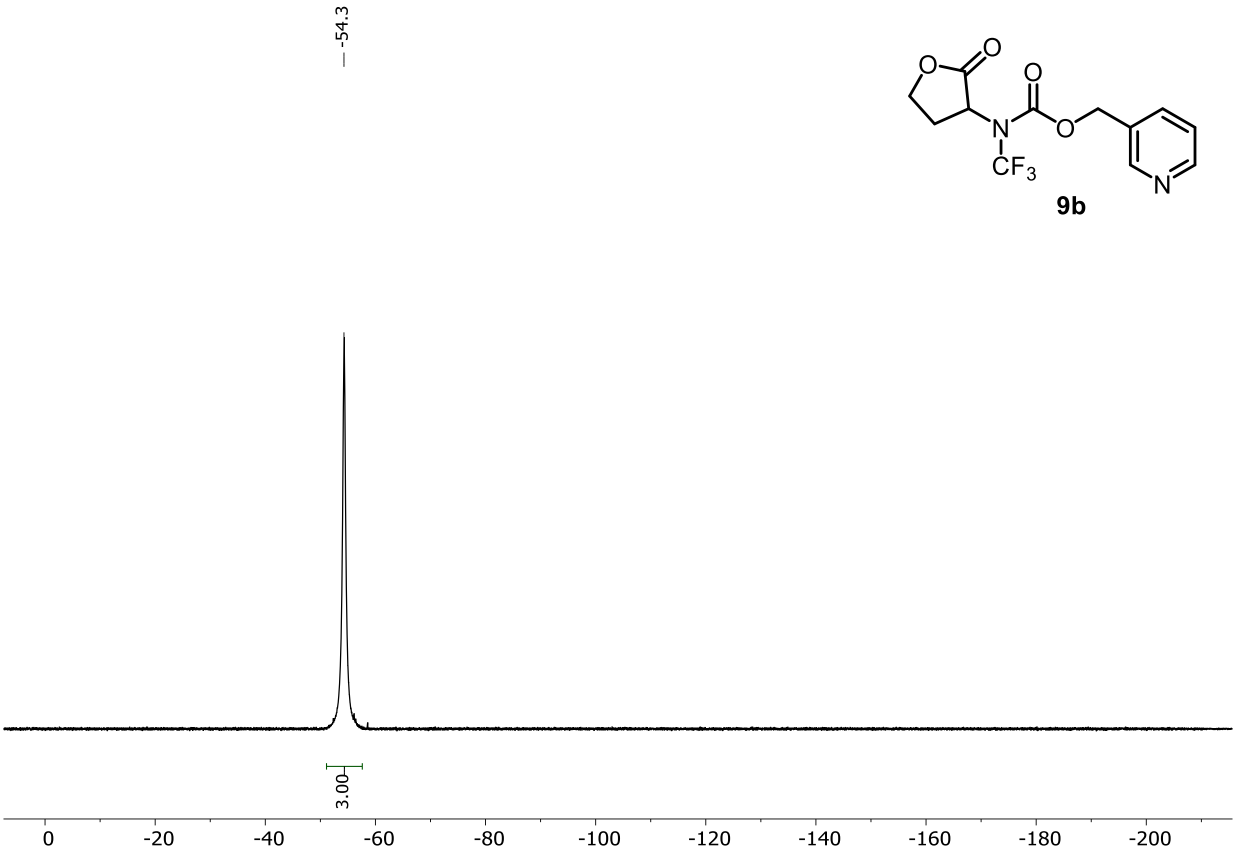

^{19}FNMR spectrum of **9b** run in $CDCl_3$ at 376 MHz.

2-(2-Methyl-5-nitro-1*H*-imidazol-1-yl)ethyl methyl(trifluoromethyl)carbamate (12a)

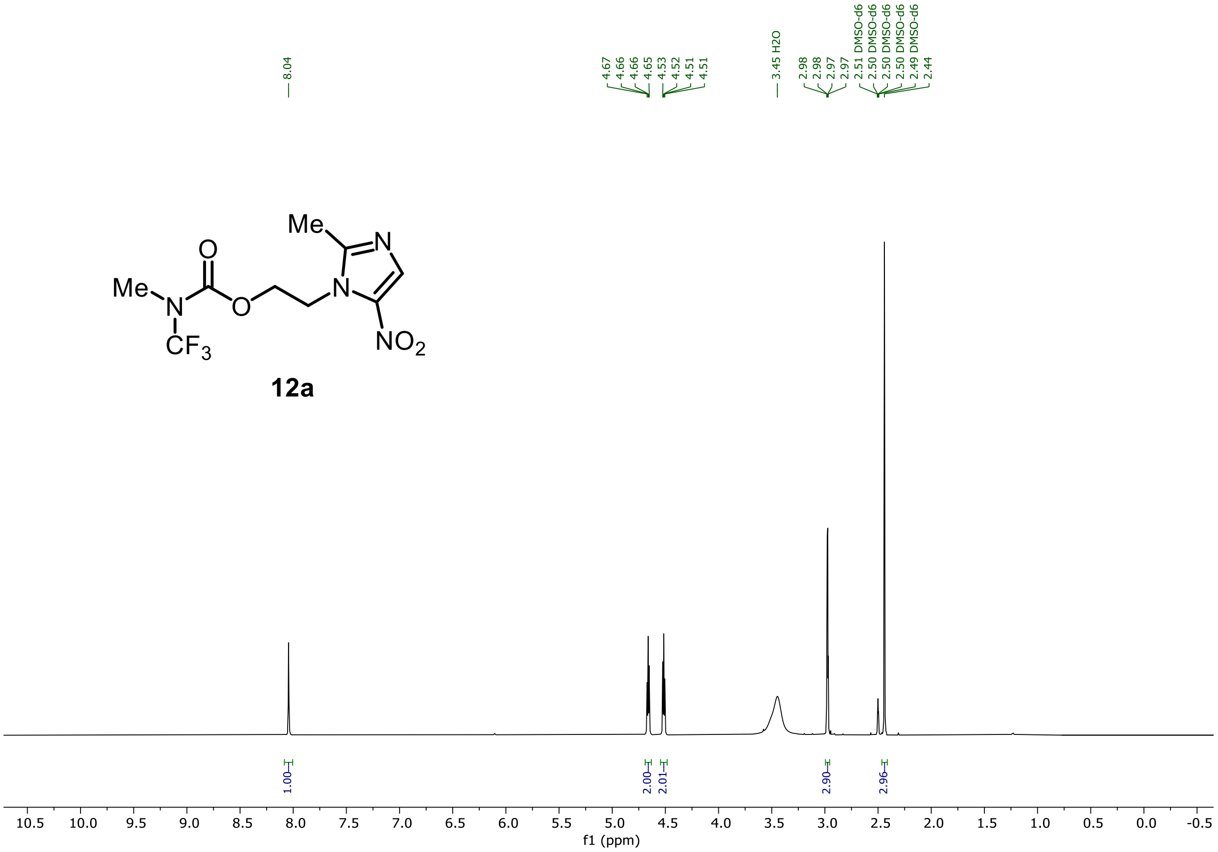

^{1}HNMR spectrum of **12a** run in DMSO-d_6 at 500 MHz.

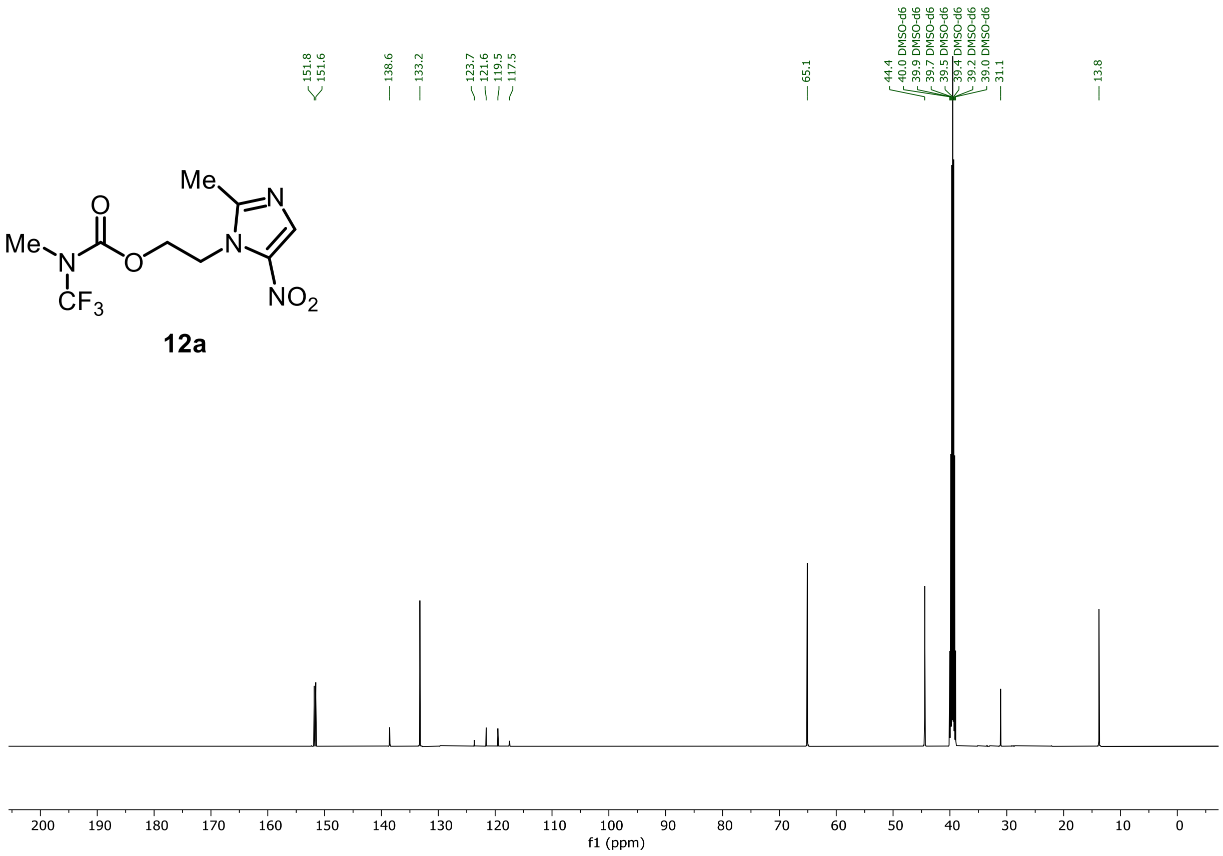

^{13}CNMR spectrum of **12a** run in DMSO-d_6 at 126 MHz.

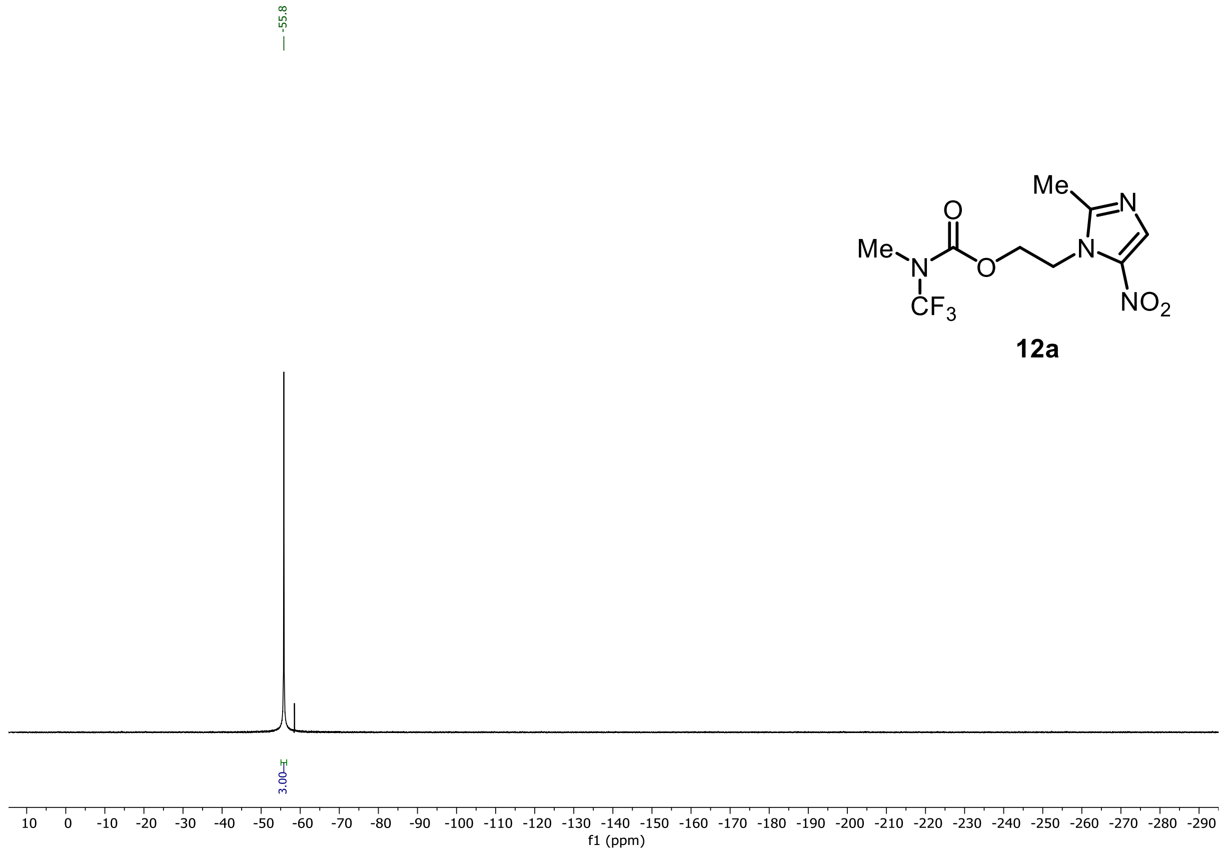

^{19}FNMR spectrum of **12a** run in DMSO-d_6 at 471 MHz.

2-(2-Methyl-5-nitro-1*H*-imidazol-1-yl)ethyl cyclopropyl(trifluoromethyl)carbamate (12b)

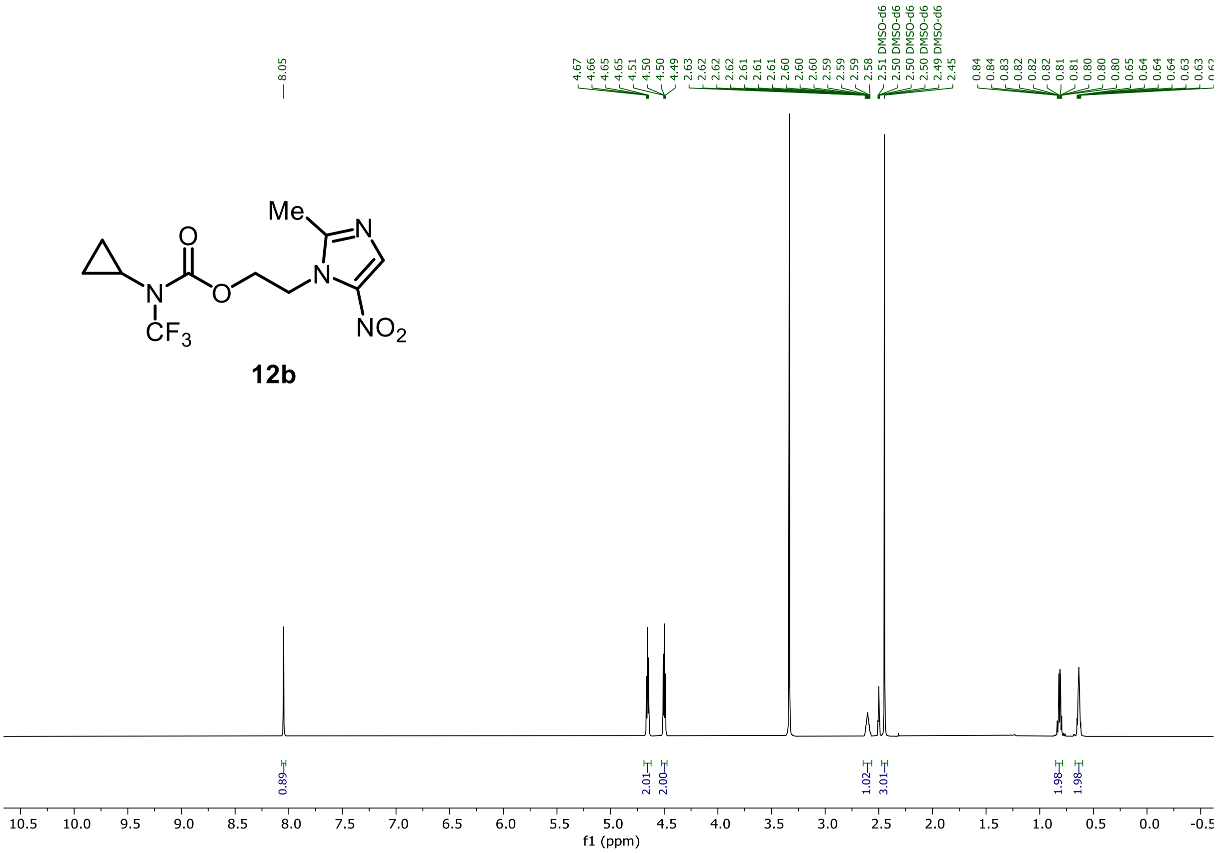

^{1}HNMR spectrum of **12b** run in DMSO-d_6 at 500 MHz.

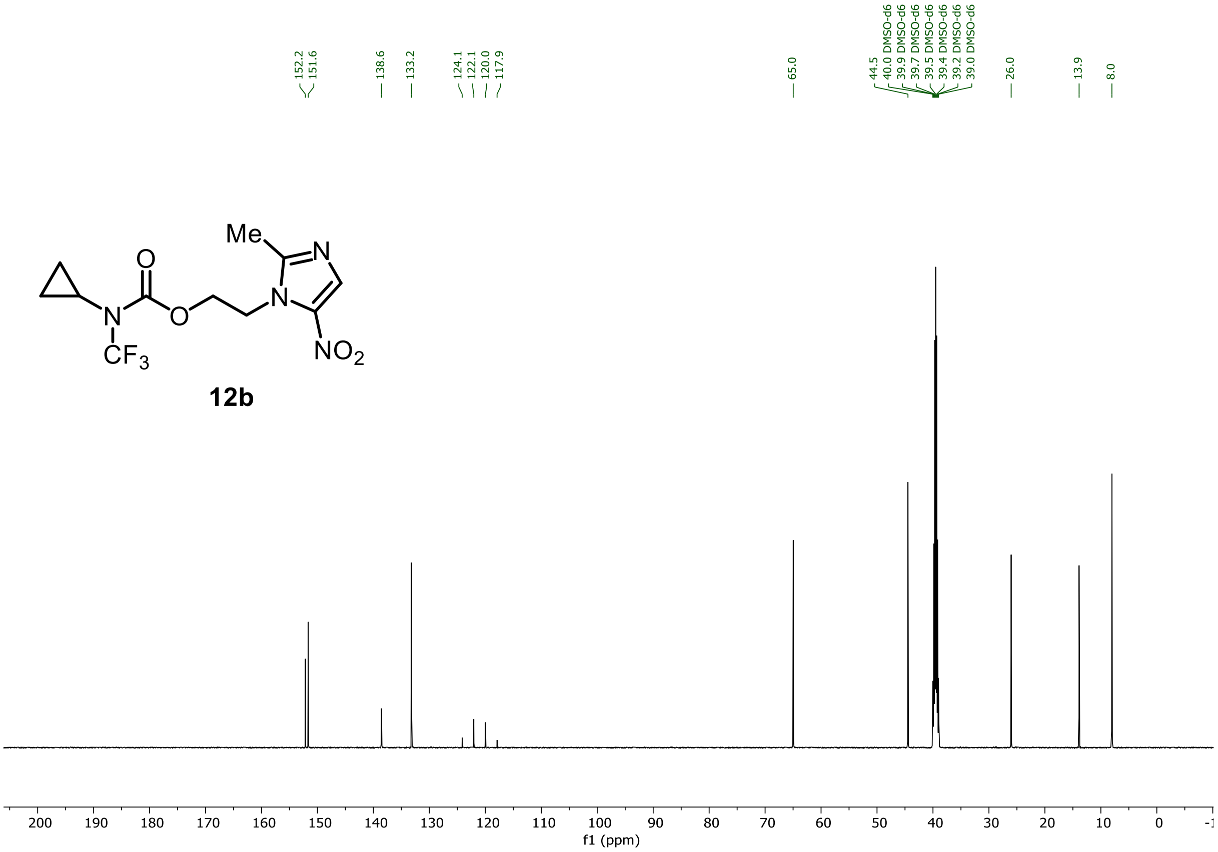

^{13}CNMR spectrum of **12b** run in DMSO-d_6 at 126 MHz.

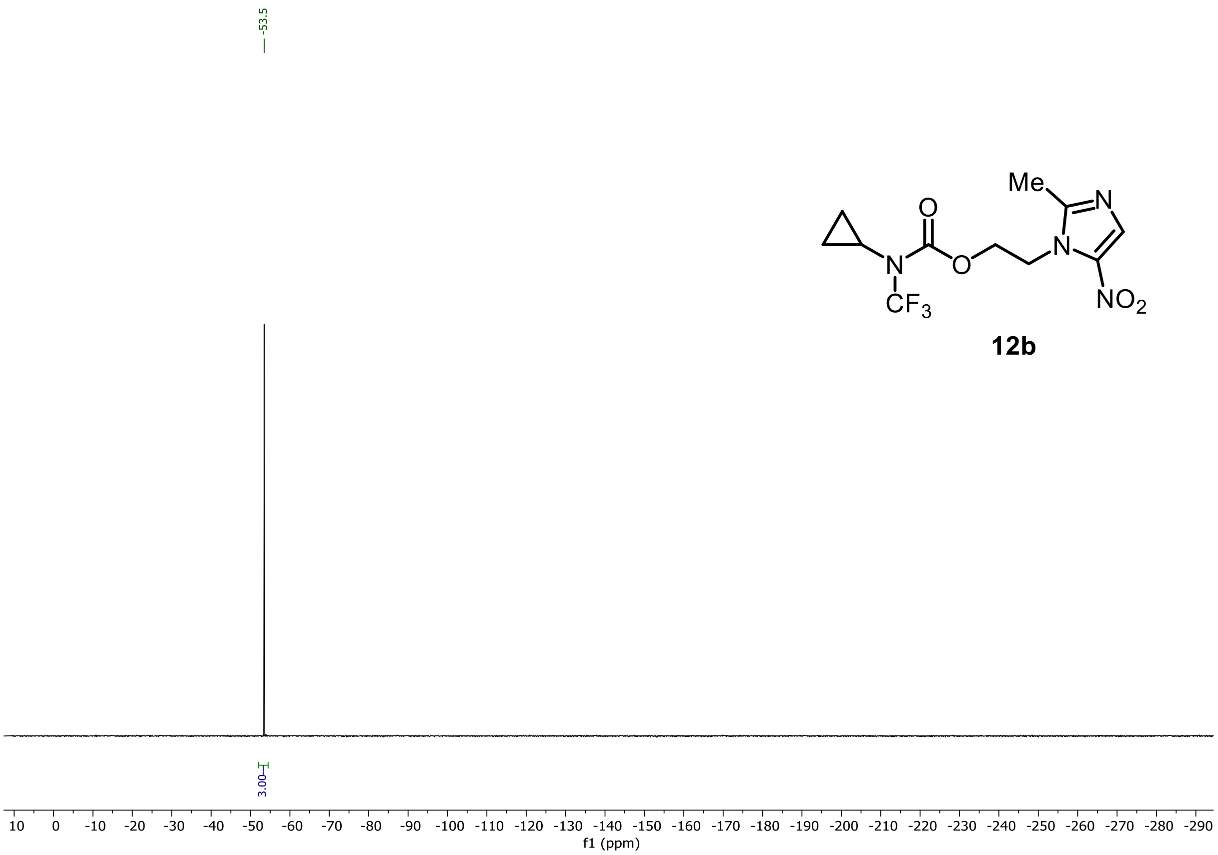

^{19}FNMR spectrum of **12b** run in DMSO-d_6 at 471 MHz.

2-(2-Methyl-5-nitro-1*H*-imidazol-1-yl)ethyl benzyl(trifluoromethyl)carbamate (12c)

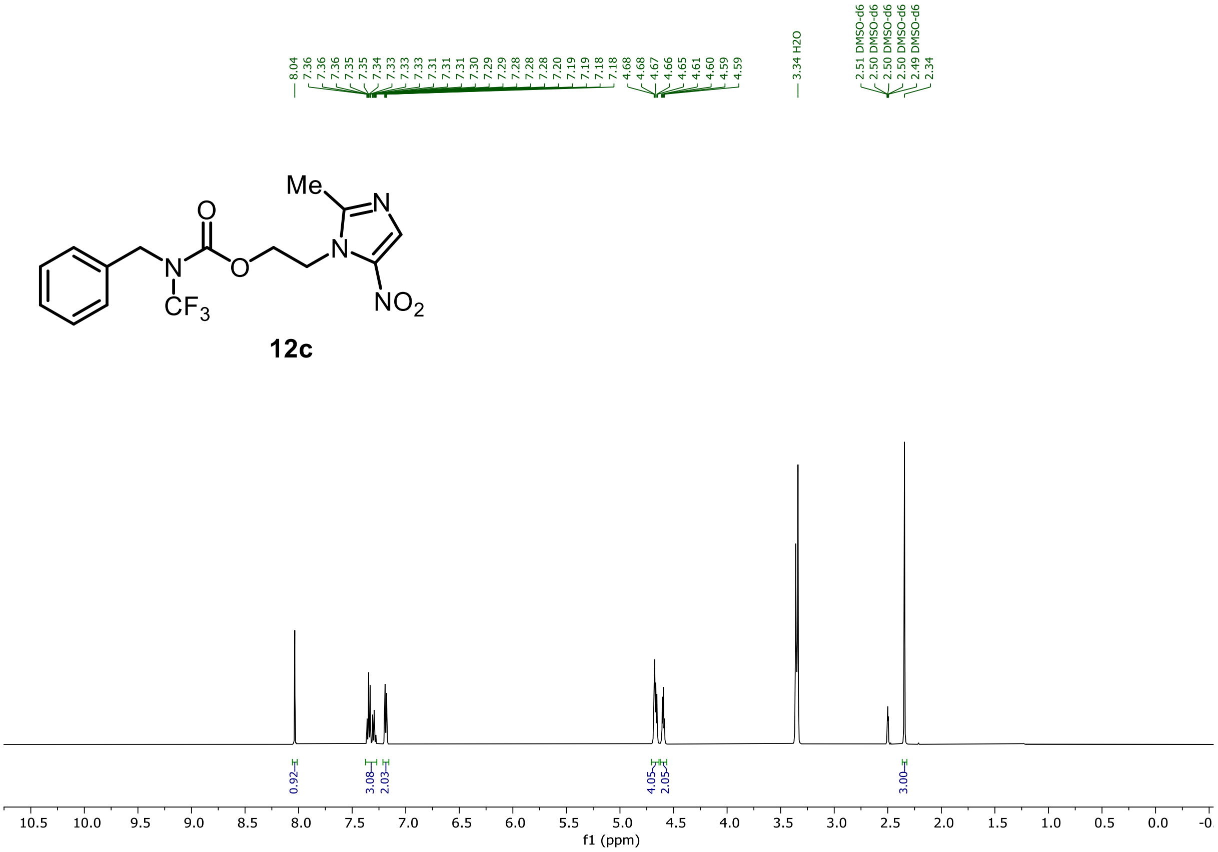

^{1}HNMR spectrum of **12c** run in DMSO-d_6 at 500 MHz.

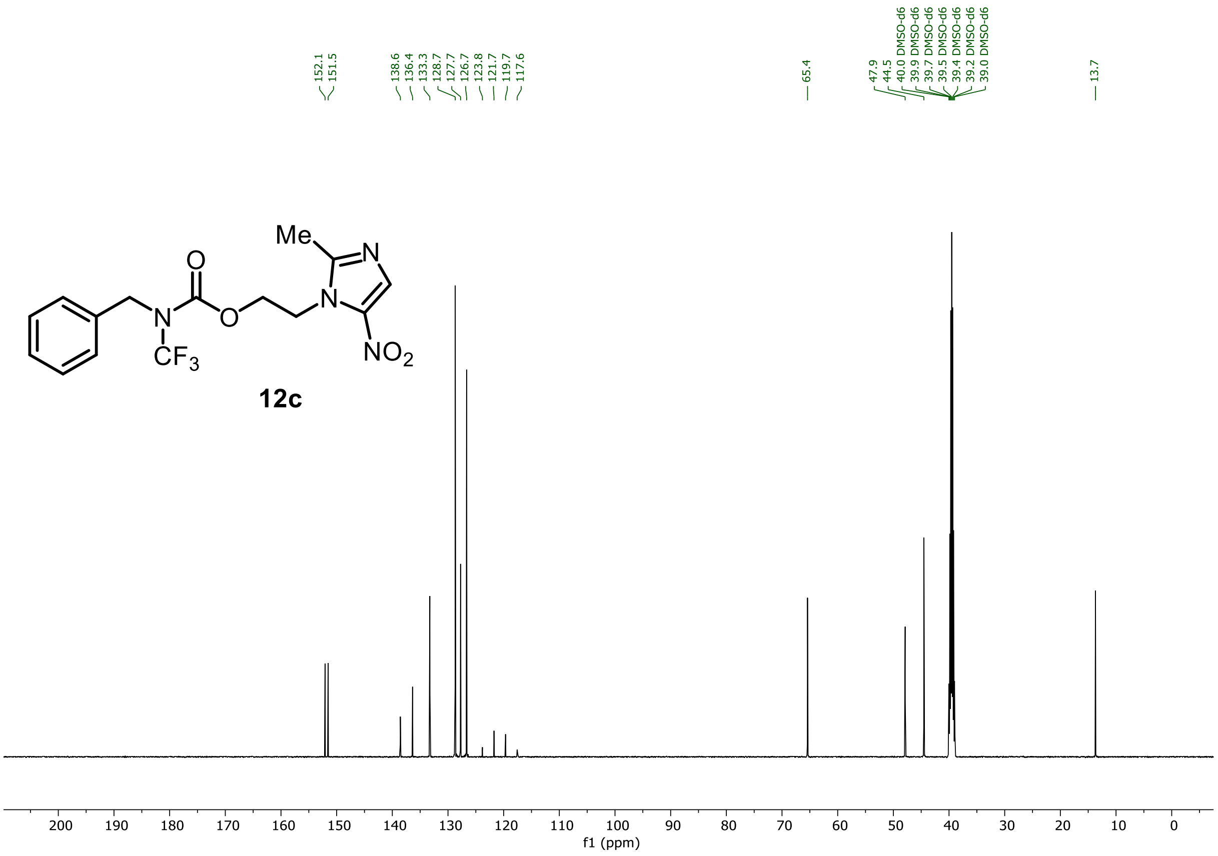

^{13}CNMR spectrum of **12c** run in DMSO-d_6 at 126 MHz.

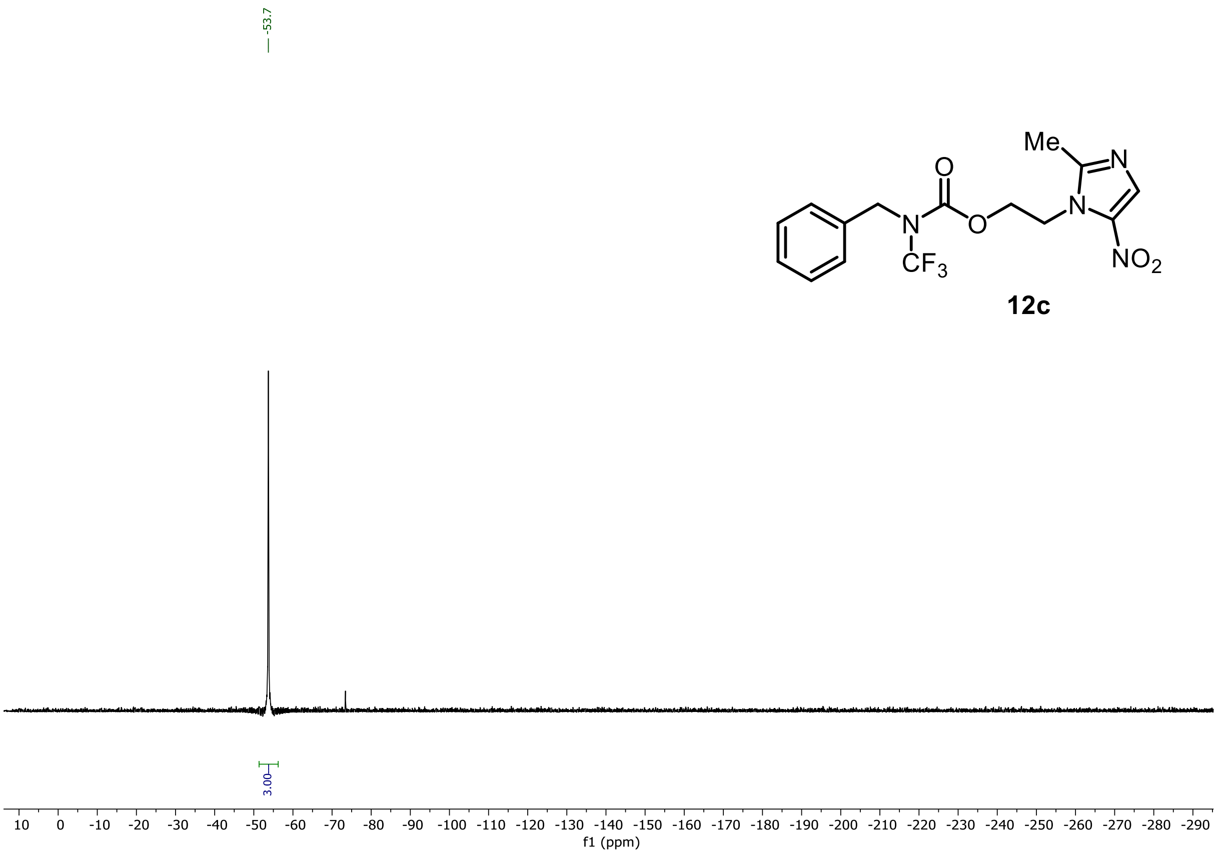

^{19}FNMR spectrum of **12c** run in DMSO-d_6 at 471 MHz.

2-(2-Methyl-5-nitro-1*H*-imidazol-1-yl)ethyl phenyl(trifluoromethyl)carbamate (12d)

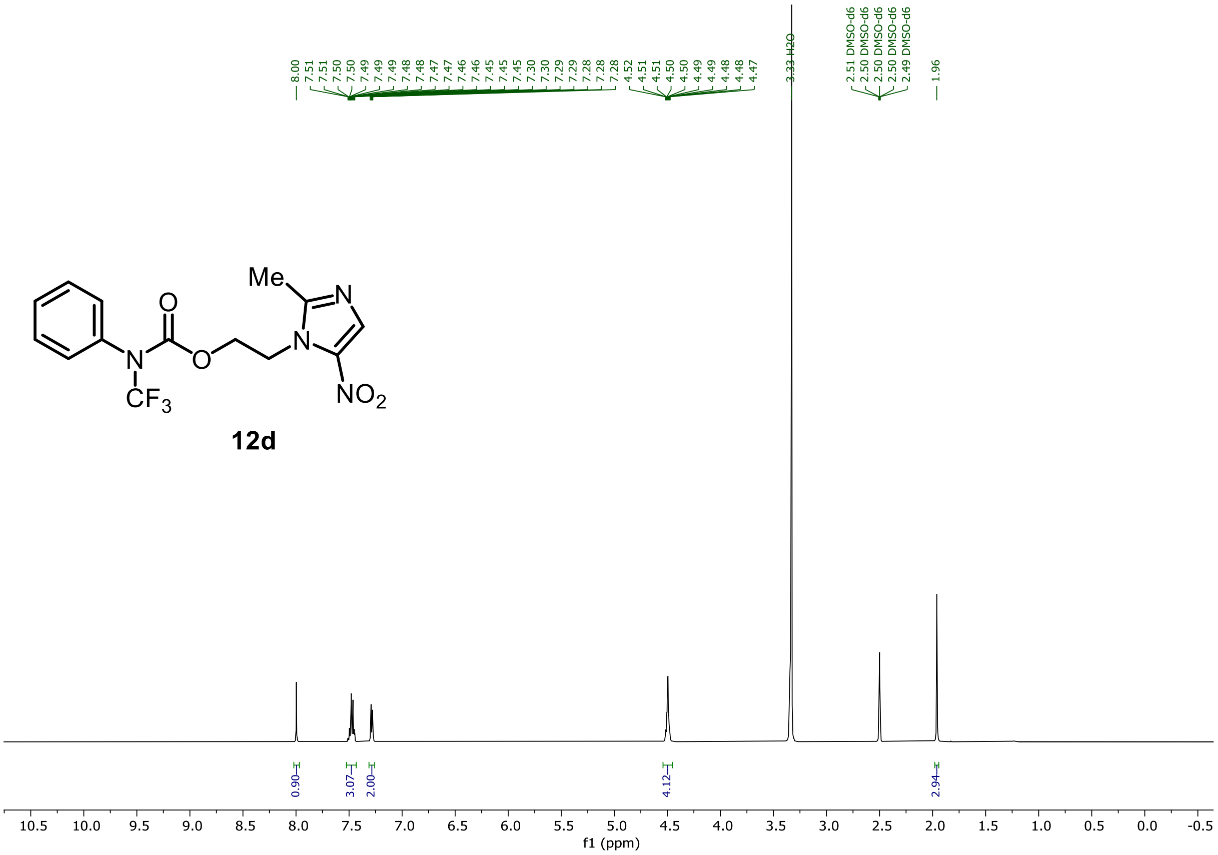

^{1}HNMR spectrum of **12d** run in DMSO-d_6 at 500 MHz.

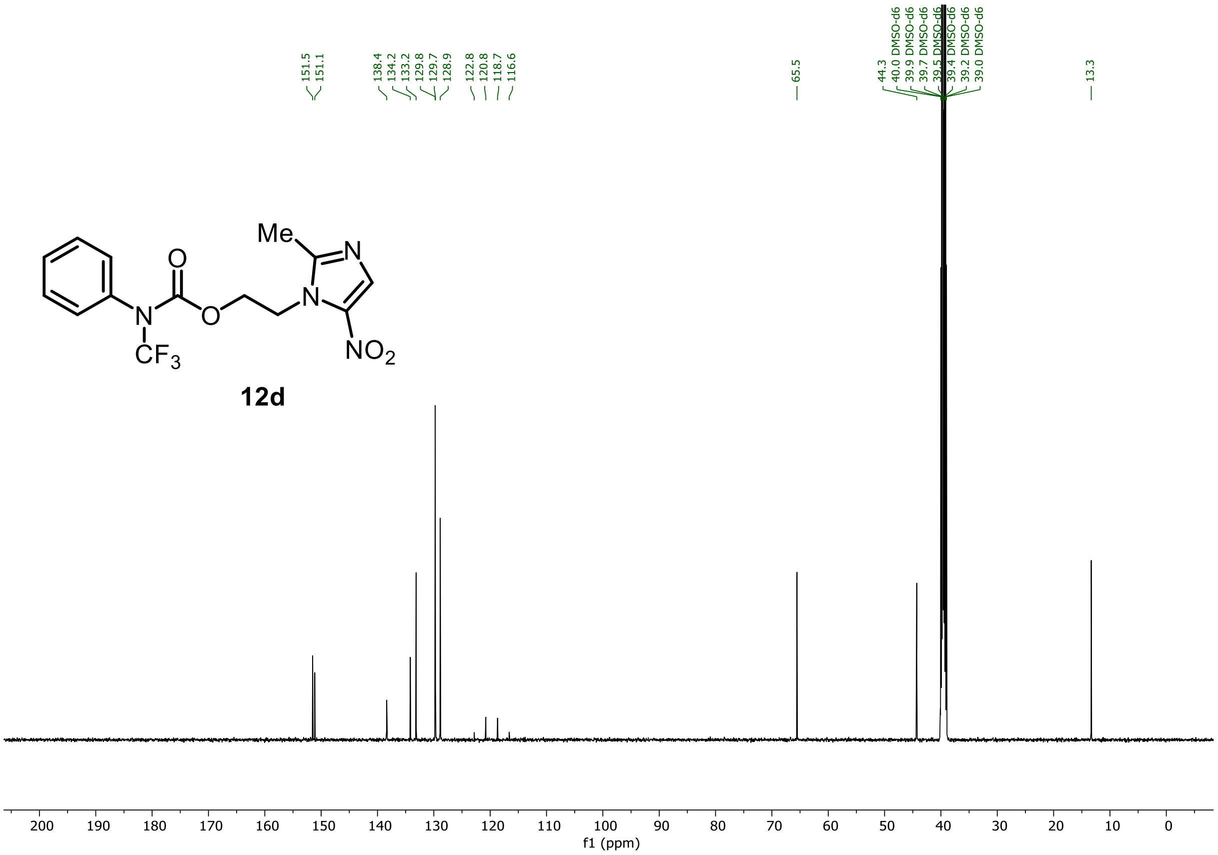

^{13}CNMR spectrum of **12d** run in DMSO-d_6 at 126 MHz.

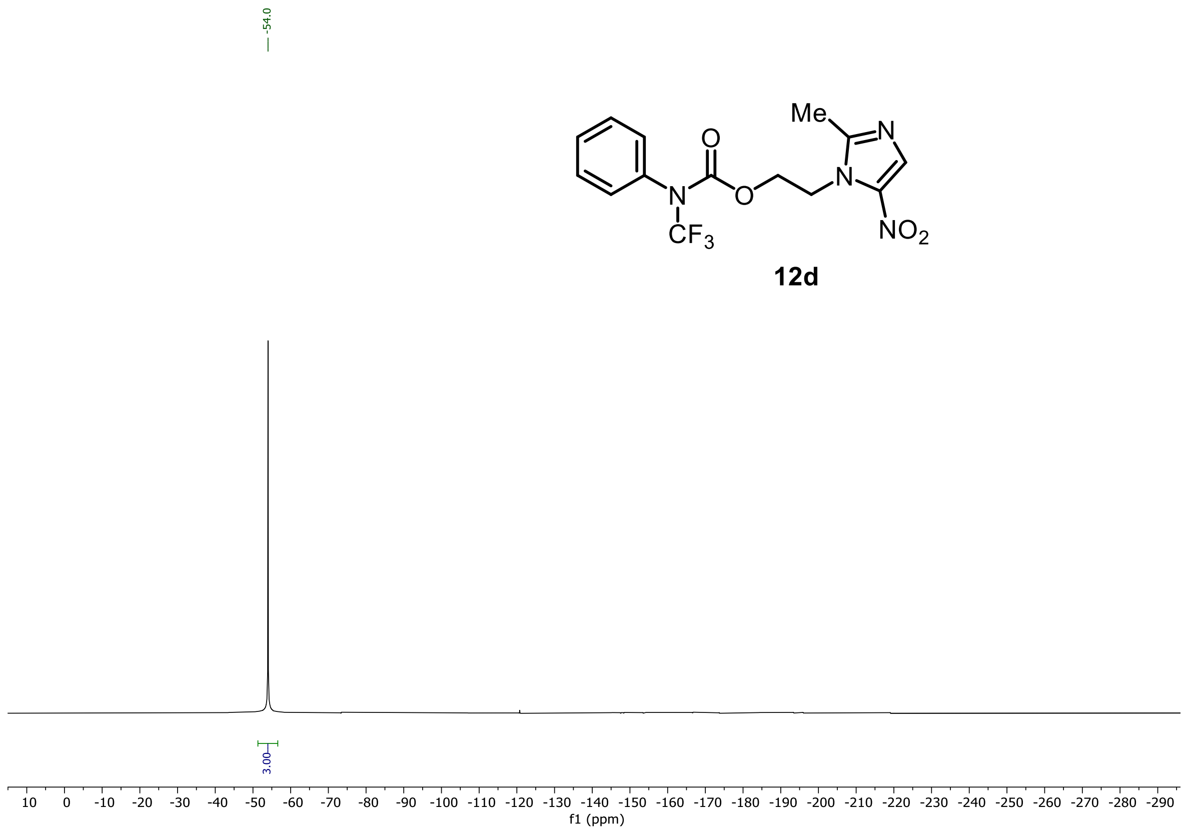

^{19}FNMR spectrum of **12d** run in DMSO-d_6 at 471 MHz.

2-(4-(2-(5-Chloro-2-oxobenzo[d]thiazol-3(2*H*)-yl)acetyl)piperazin-1-yl)ethyl methyl(trifluoro-methyl)carbamate (13a)

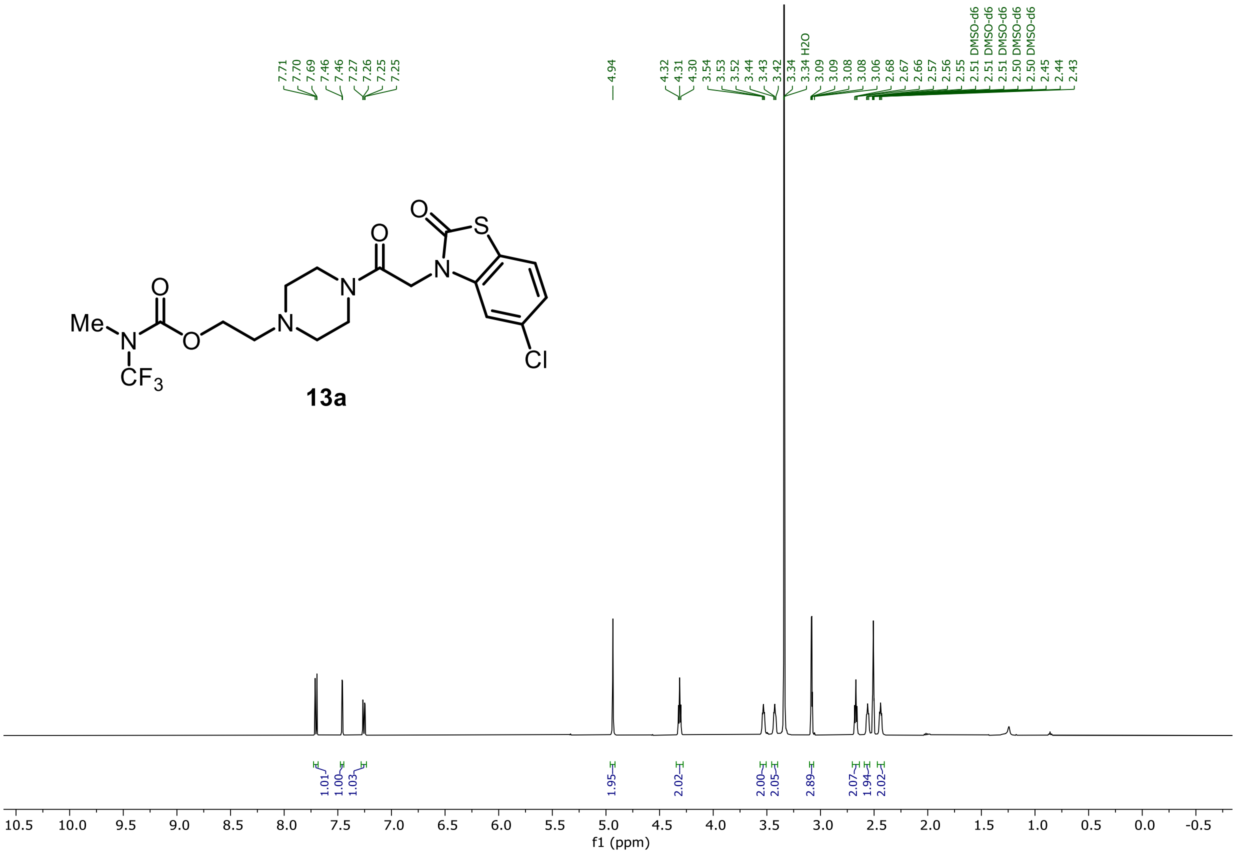

^{1}HNMR spectrum of **13a** run in DMSO-d_6 at 500 MHz.

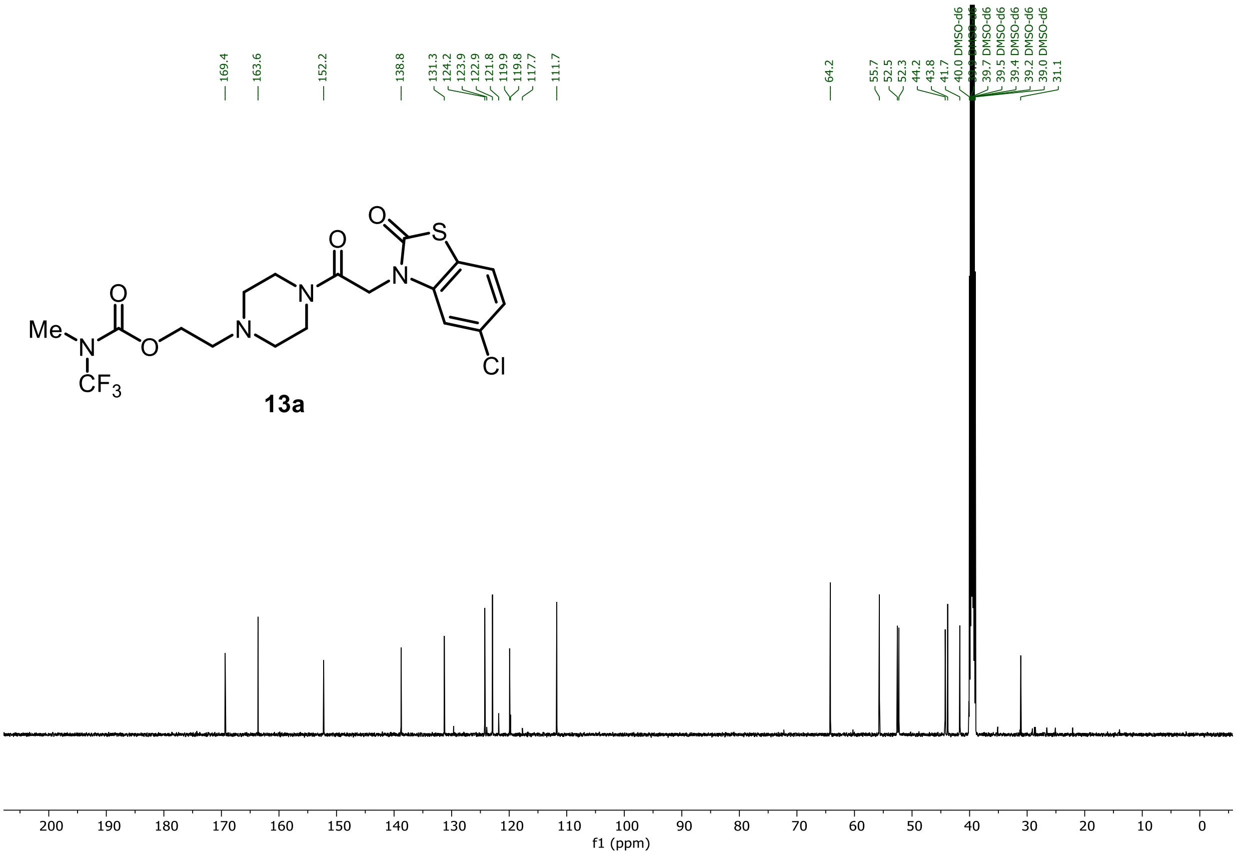

^{13}CNMR spectrum of **13a** run in DMSO-d_6 at 126 MHz.

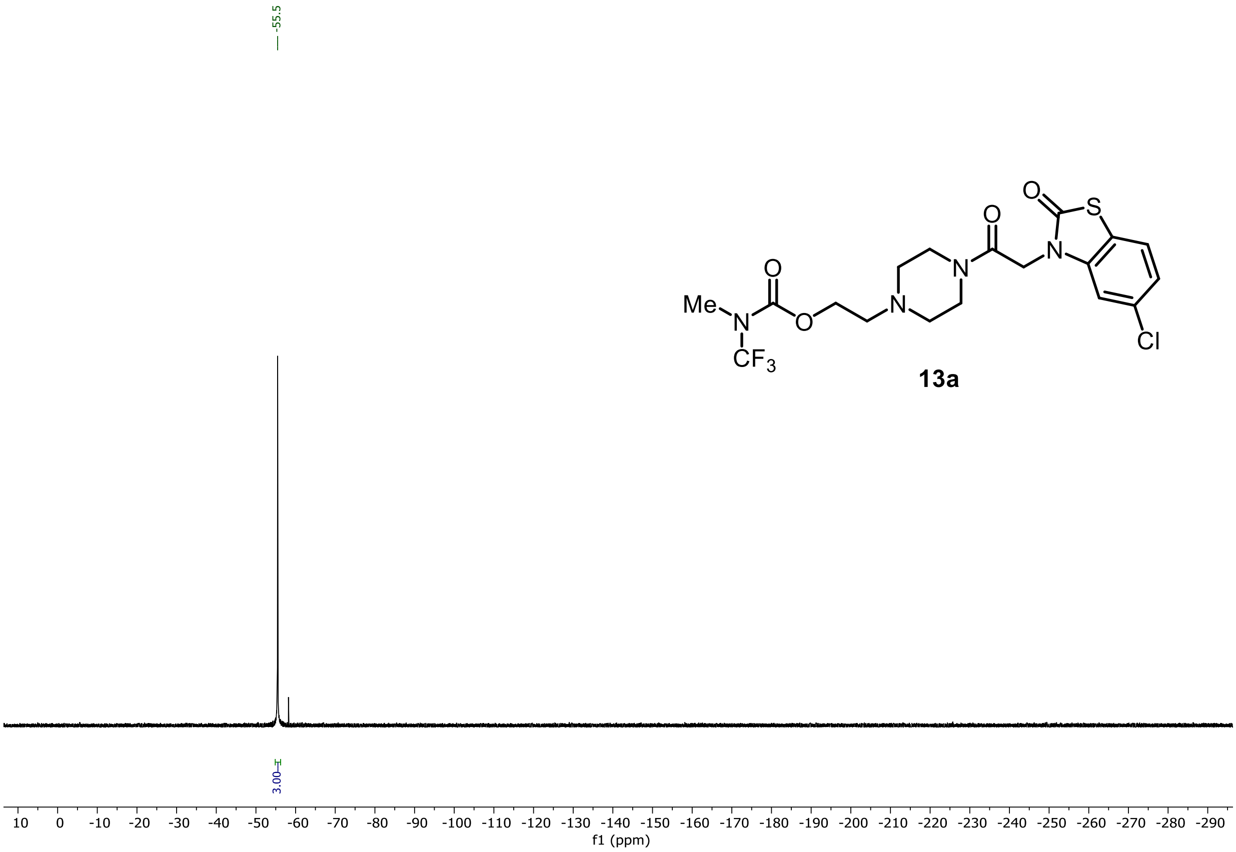

^{19}FNMR spectrum of **13a** run in DMSO-d_6 at 471 MHz.

2-(4-(2-(5-Chloro-2-oxobenzo[*d*]thiazol-3(2*H*)-yl)acetyl)piperazin-1-yl)ethyl cyclopropyl(trifluoromethyl)carbamate (13b)

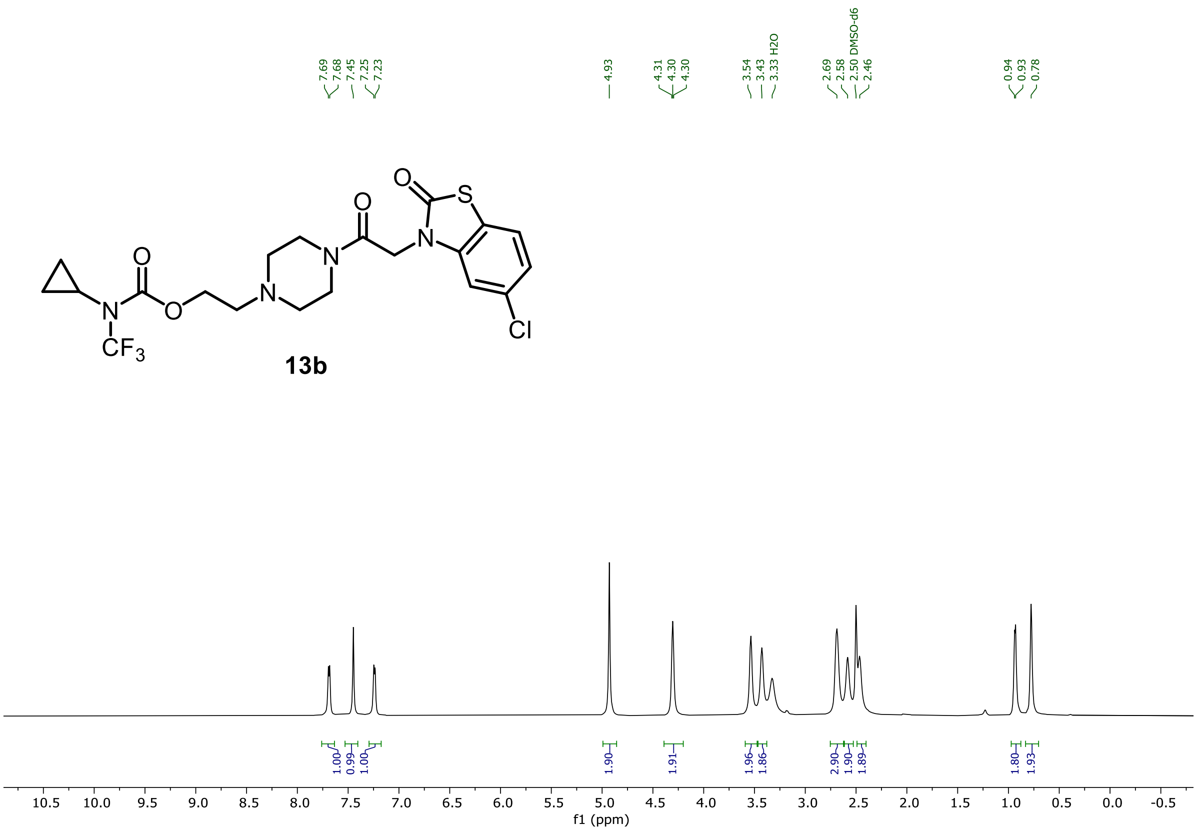

^{1}HNMR spectrum of **13b** run in DMSO-d_6 at 600 MHz.

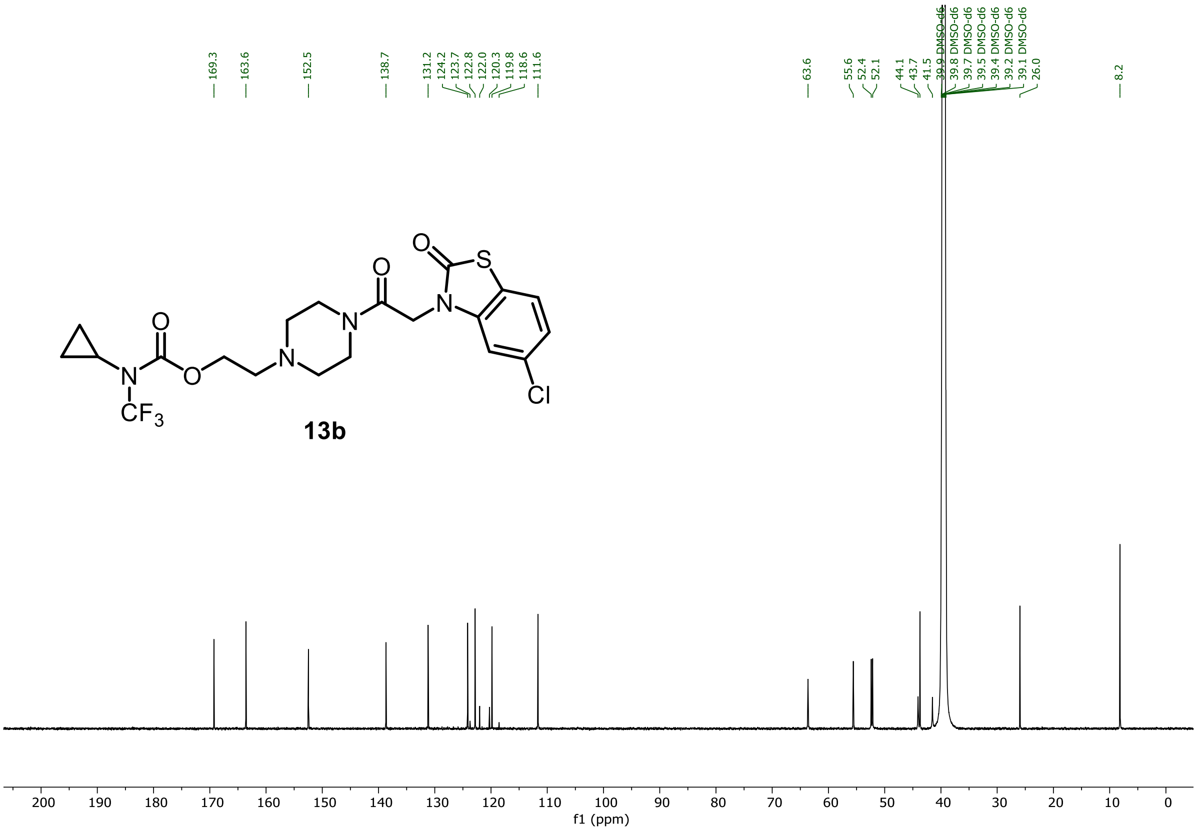

^{13}CNMR spectrum of **13b** run in DMSO-d_6 at 151 MHz.

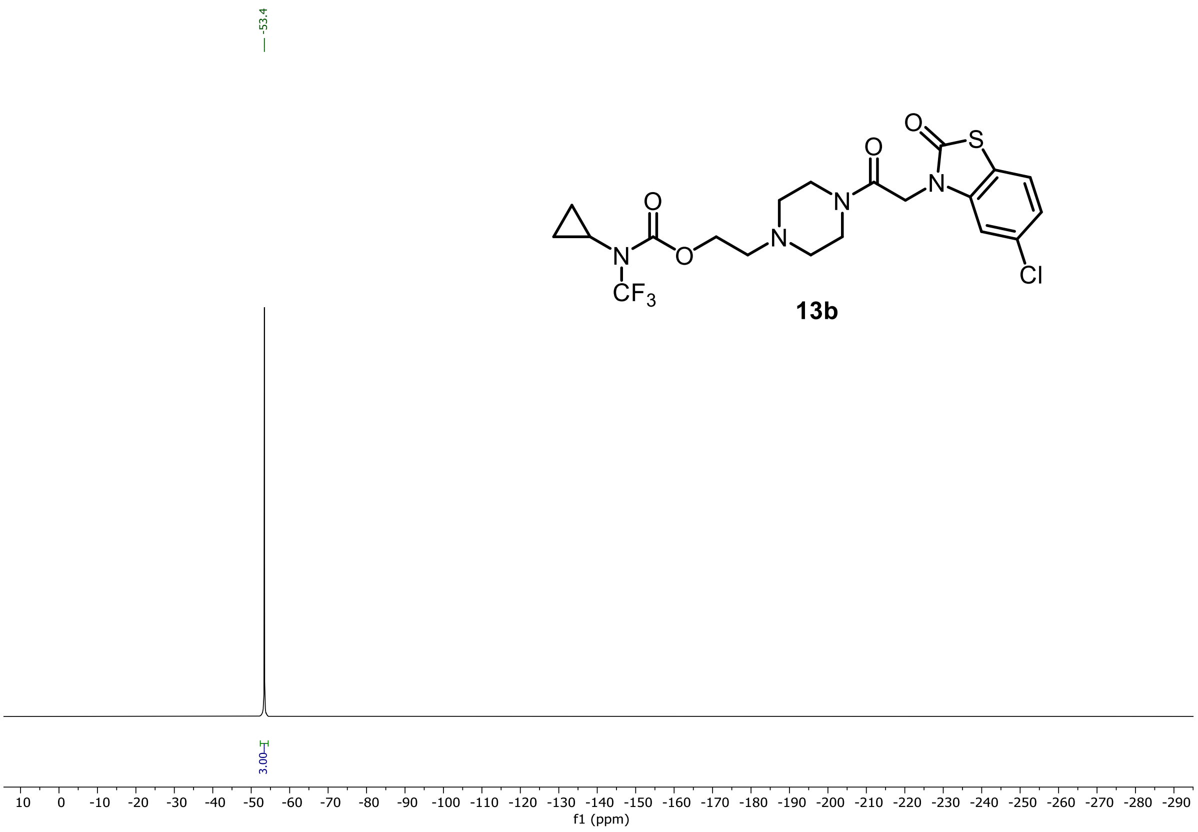

^{19}FNMR spectrum of **13b** run in DMSO-d_6 at 471 MHz.

2-(4-(2-(5-Chloro-2-oxobenzo[*d*]thiazol-3(2*H*)-yl)acetyl)piperazin-1-yl)ethyl benzyl(trifluoromethyl)carbamate (13c)

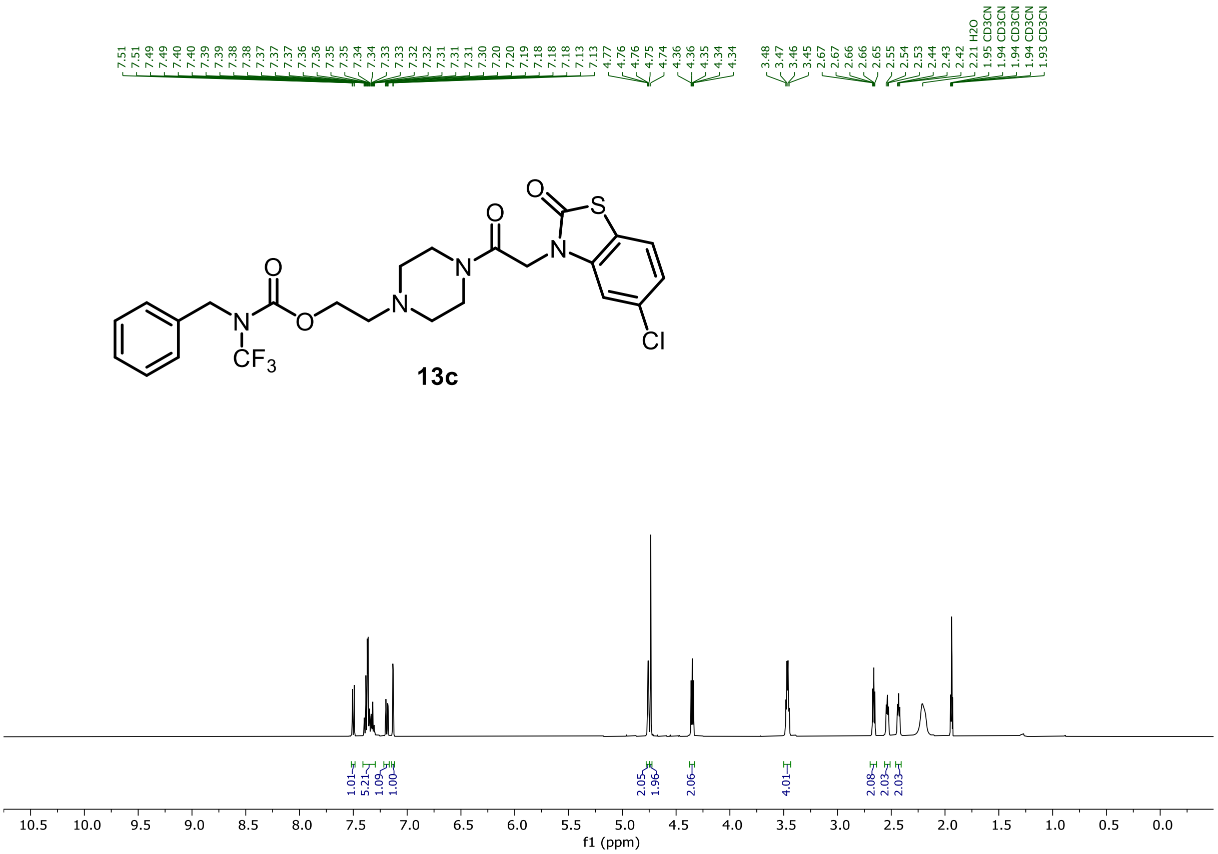

^{1}HNMR spectrum of **13c** run in CD_3CN at 500 MHz.

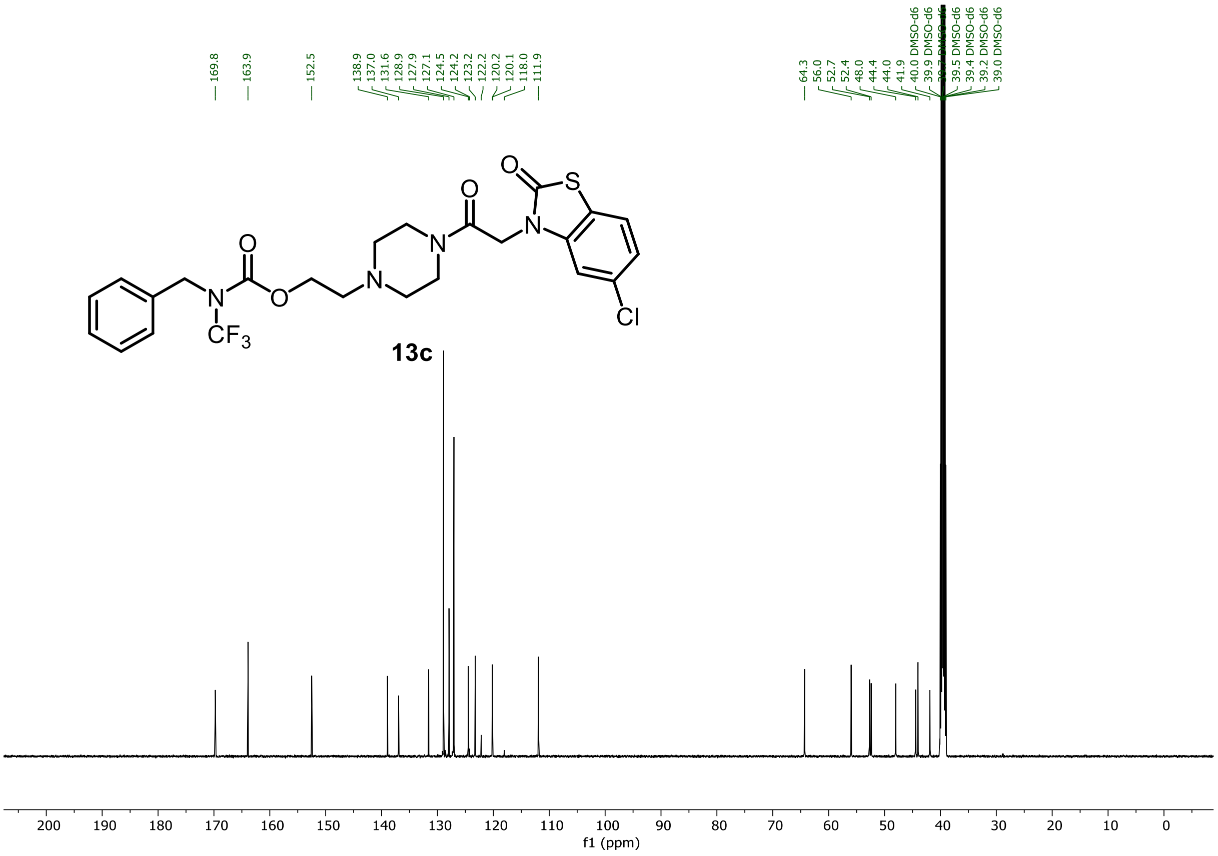

^{13}CNMR spectrum of **13c** run in DMSO-d_6 at 126 MHz.

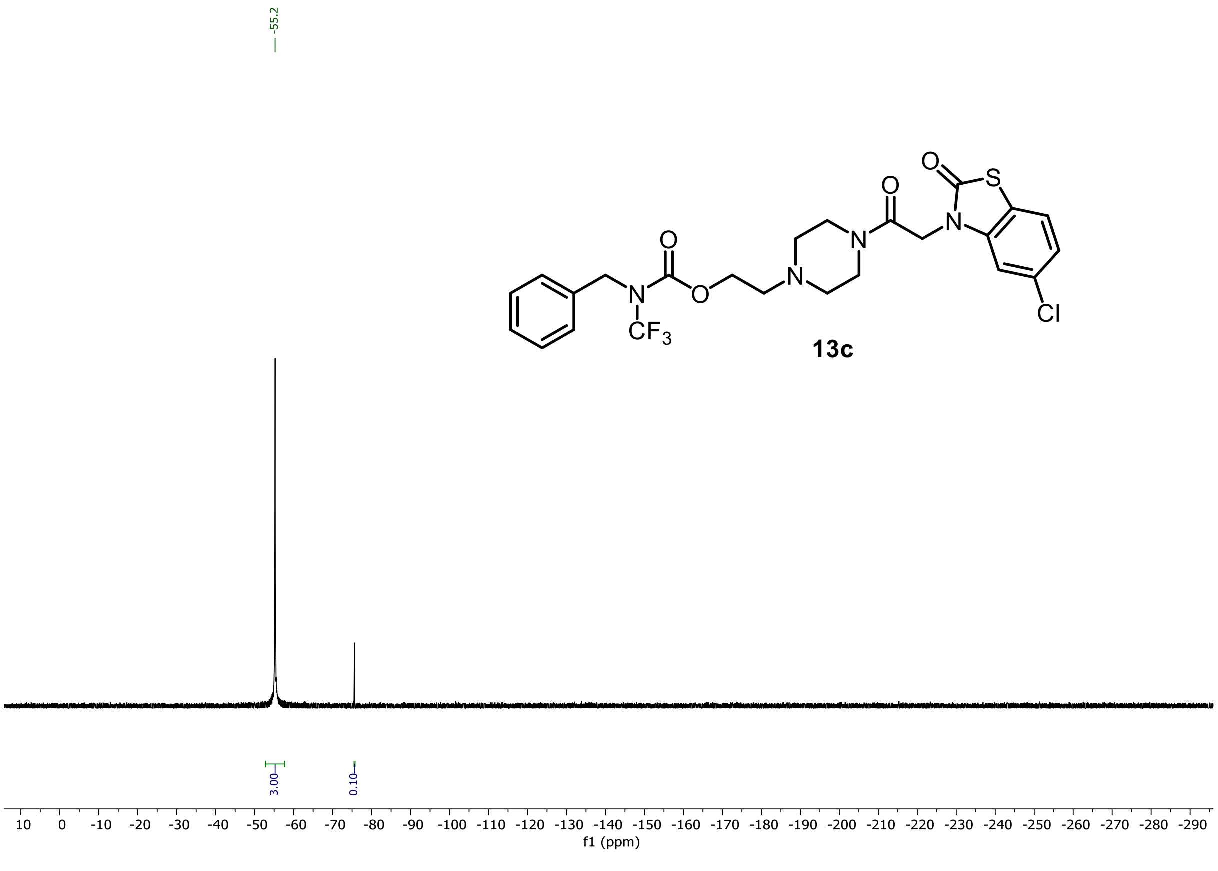

^{19}FNMR spectrum of **13c** run in DMSO-d_6 at 471 MHz.

2-(4-(2-(5-Chloro-2-oxobenzo[*d*]thiazol-3(2*H*)-yl)acetyl)piperazin-1-yl)ethyl phenyl(trifluoromethyl)carbamate (13d)

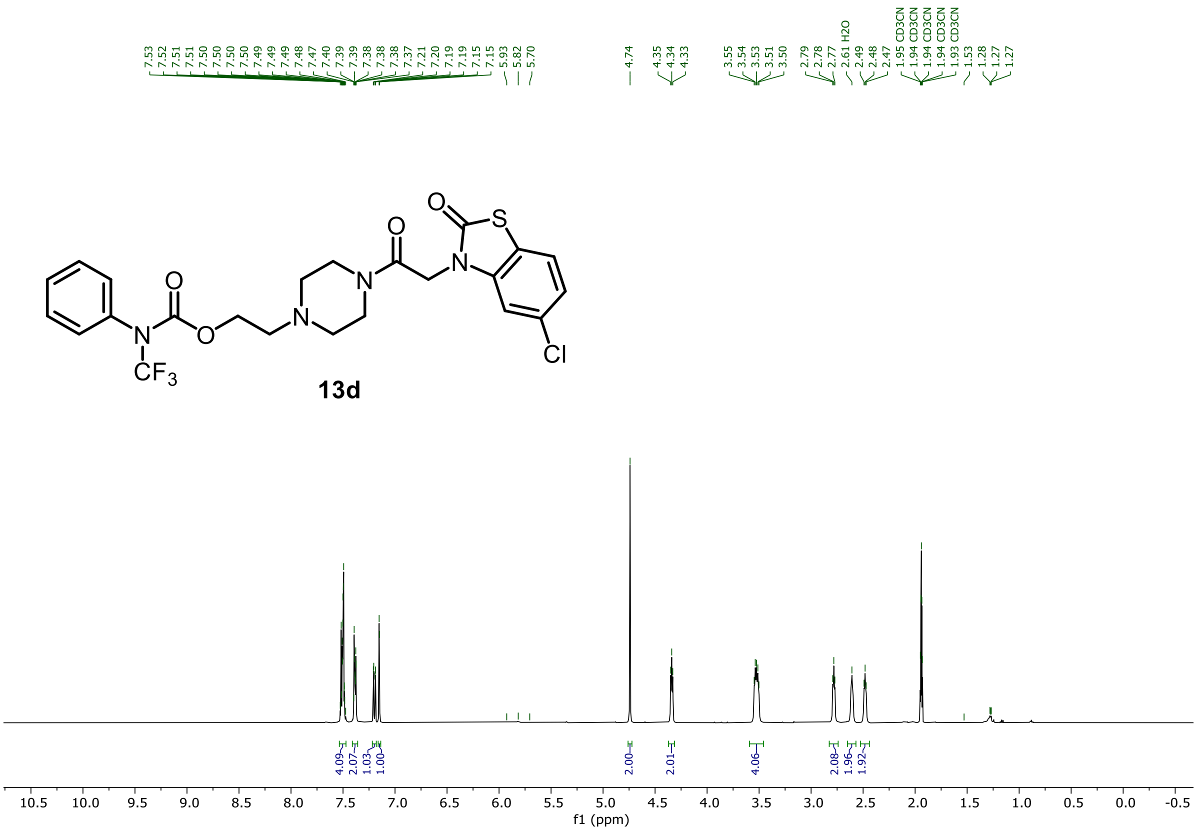

^{1}HNMR spectrum of **13d** run in CD_3CN at 500 MHz.

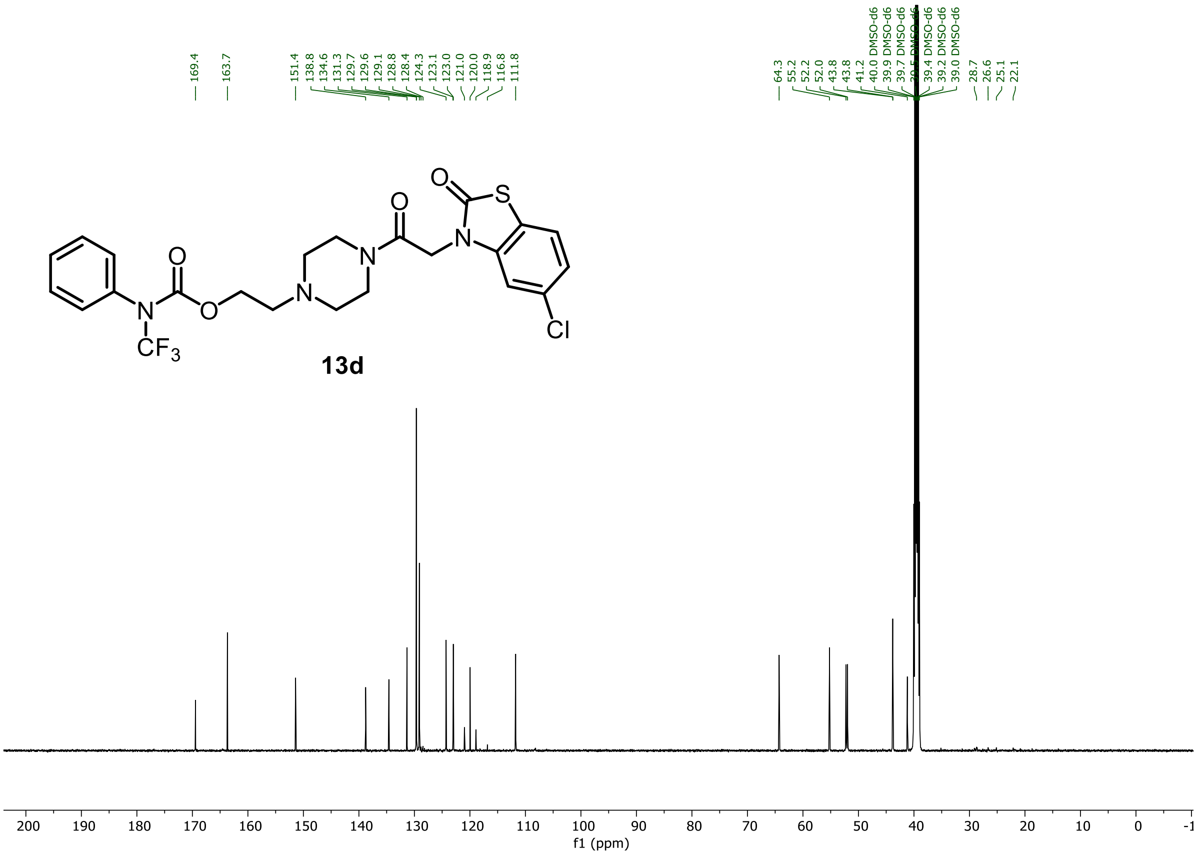

^{13}CNMR spectrum of **13d** run in DMSO-d_6 at 126 MHz.

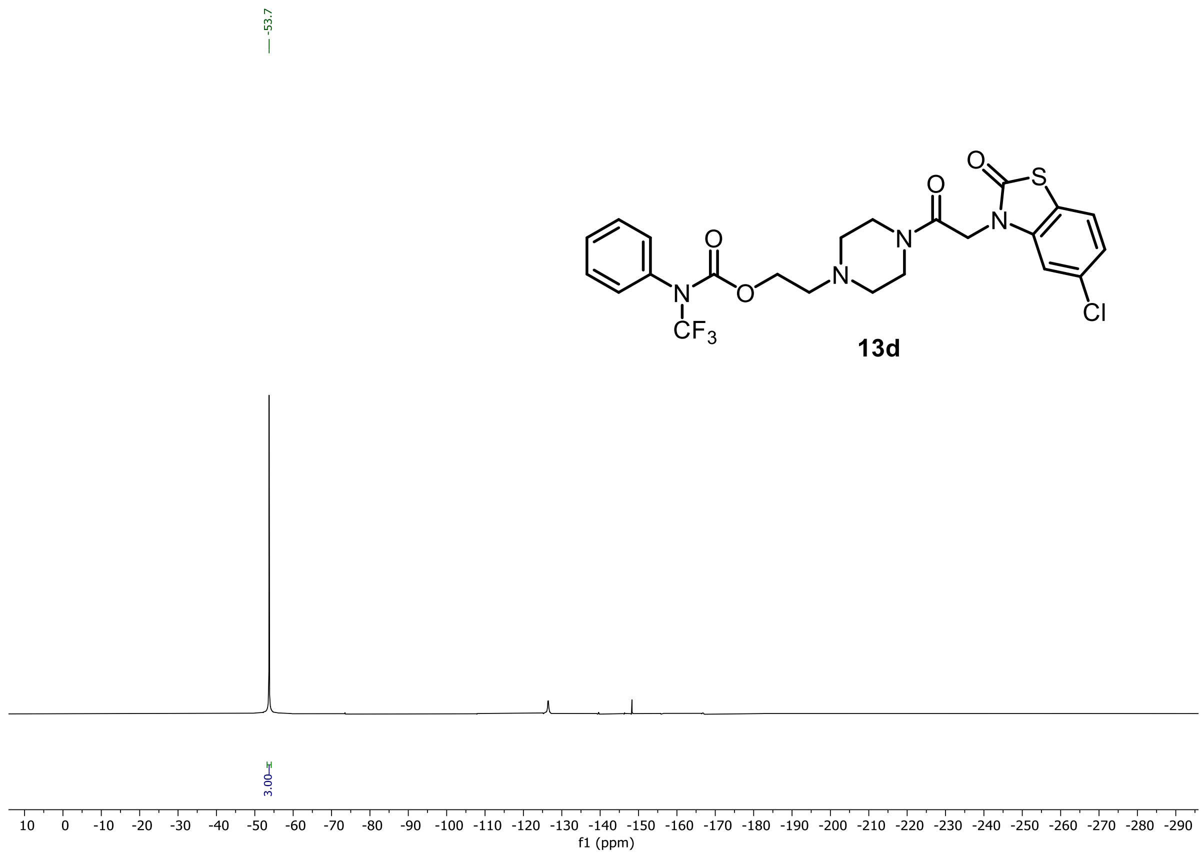

^{19}FNMR spectrum of **13d** run in DMSO-d_6 at 471 MHz.

4-Acetamidophenyl methyl(trifluoromethyl)carbamate (14a)

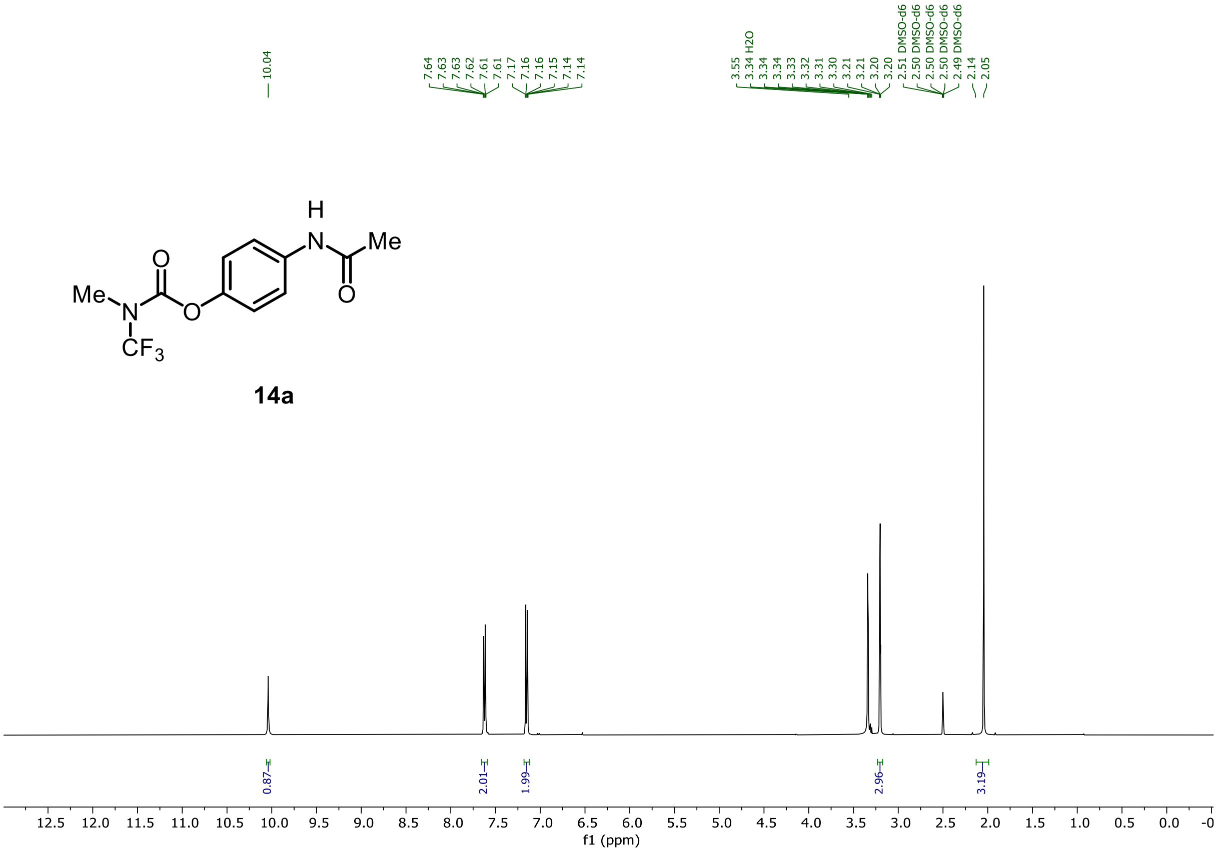

^{1}HNMR spectrum of **14a** run in DMSO-d_6 at 500 MHz.

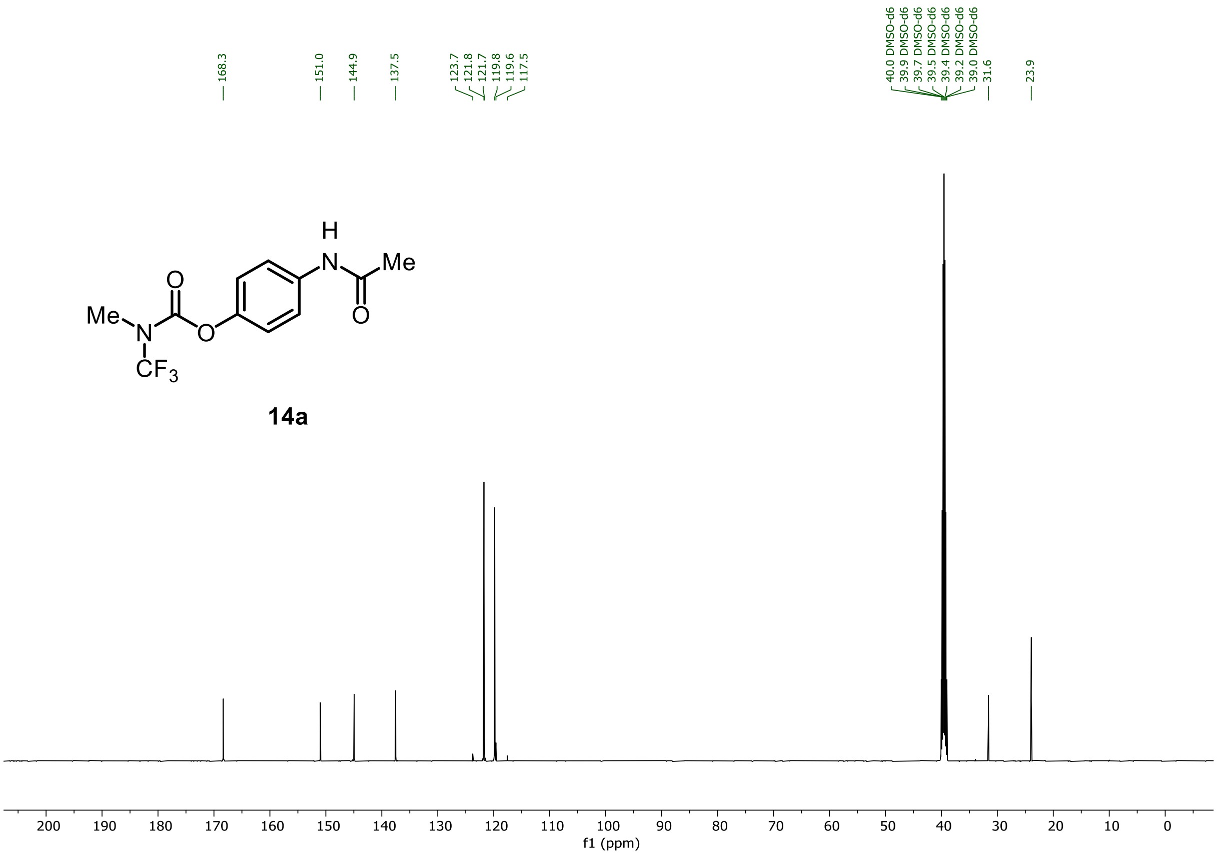

^{13}CNMR spectrum of **14a** run in DMSO-d_6 at 126 MHz.

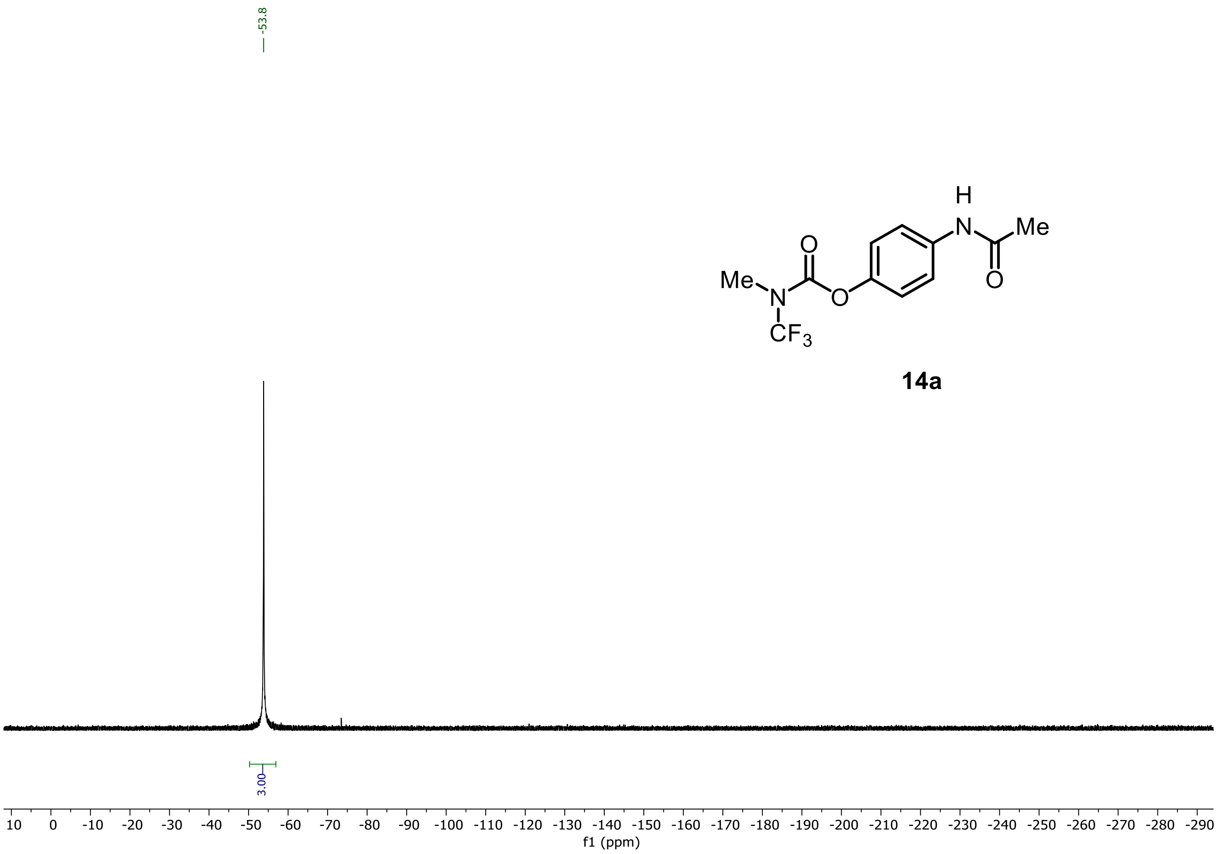

^{19}FNMR spectrum of **14a** run in DMSO-d_6 at 471 MHz.

4-Acetamidophenyl cyclopropyl(trifluoromethyl)carbamate (14b)

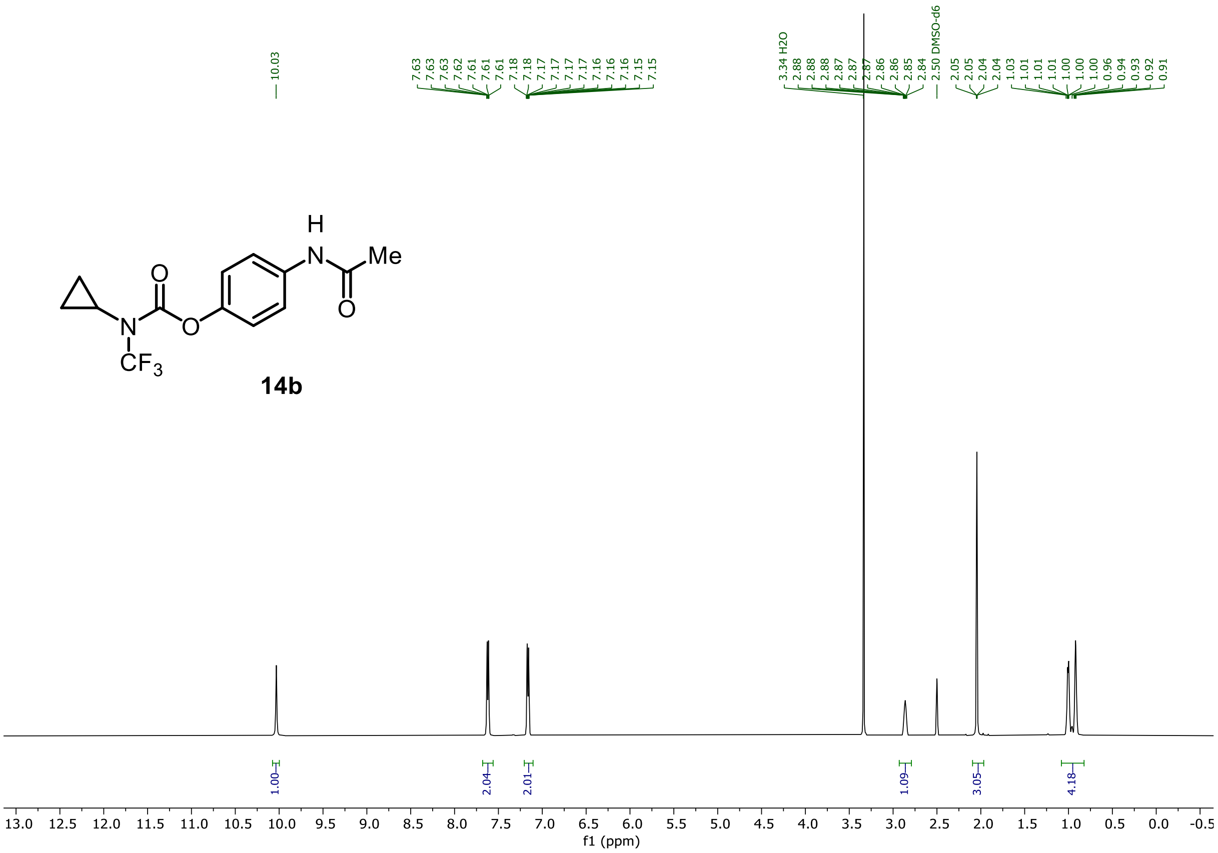

^{1}HNMR spectrum of **14b** run in DMSO-d_6 at 500 MHz.

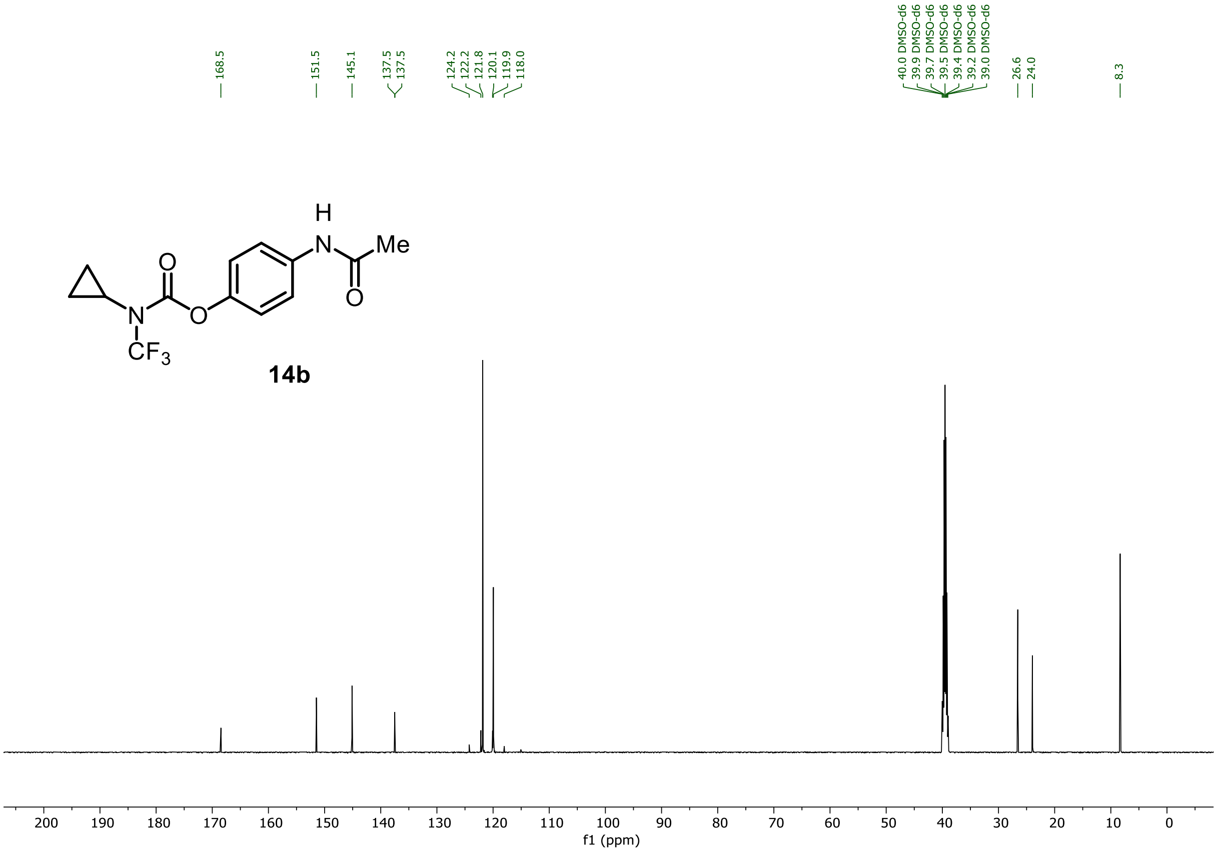

^{13}CNMR spectrum of **14b** run in DMSO-d_6 at 126 MHz.

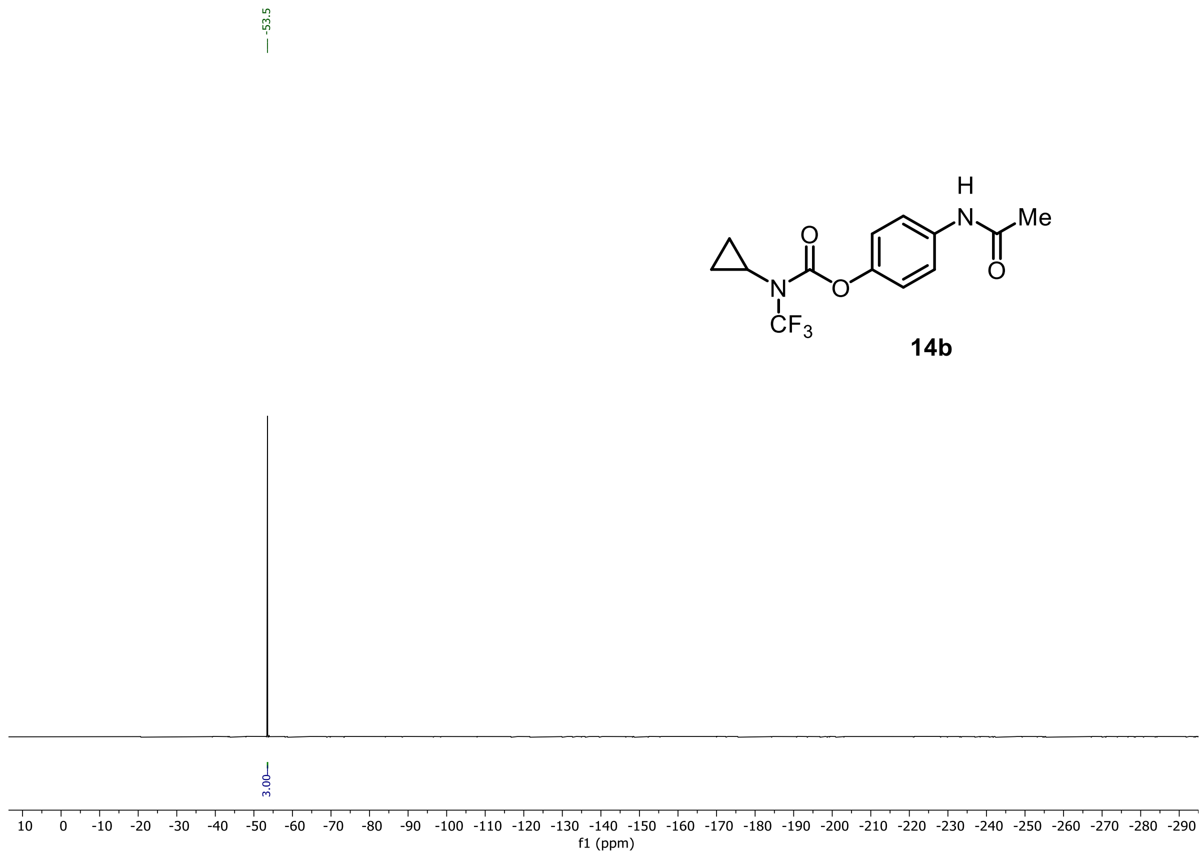

^{19}FNMR spectrum of **14b** run in DMSO-d_6 at 471 MHz.

4-Acetamidophenyl benzyl(trifluoromethyl)carbamate (14c)

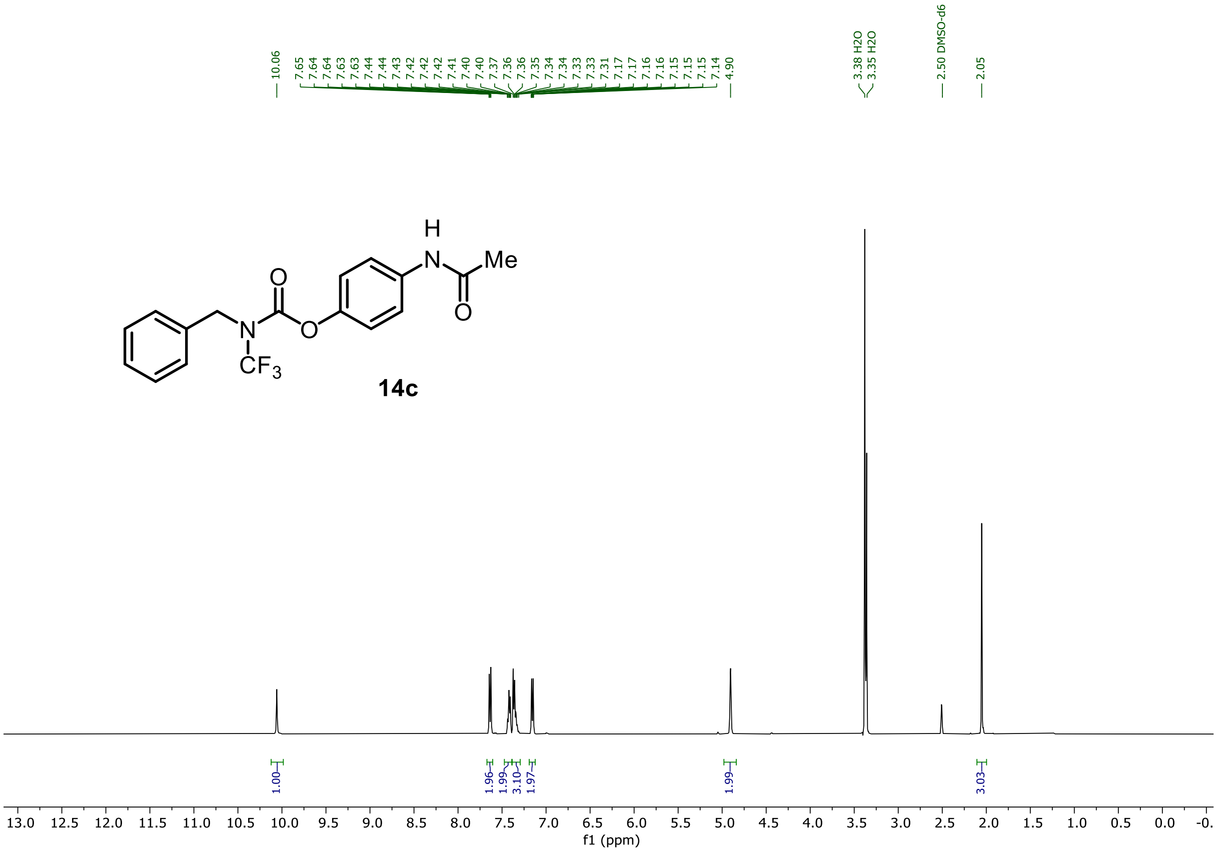

^{1}HNMR spectrum of **14c** run in DMSO-d_6 at 500 MHz.

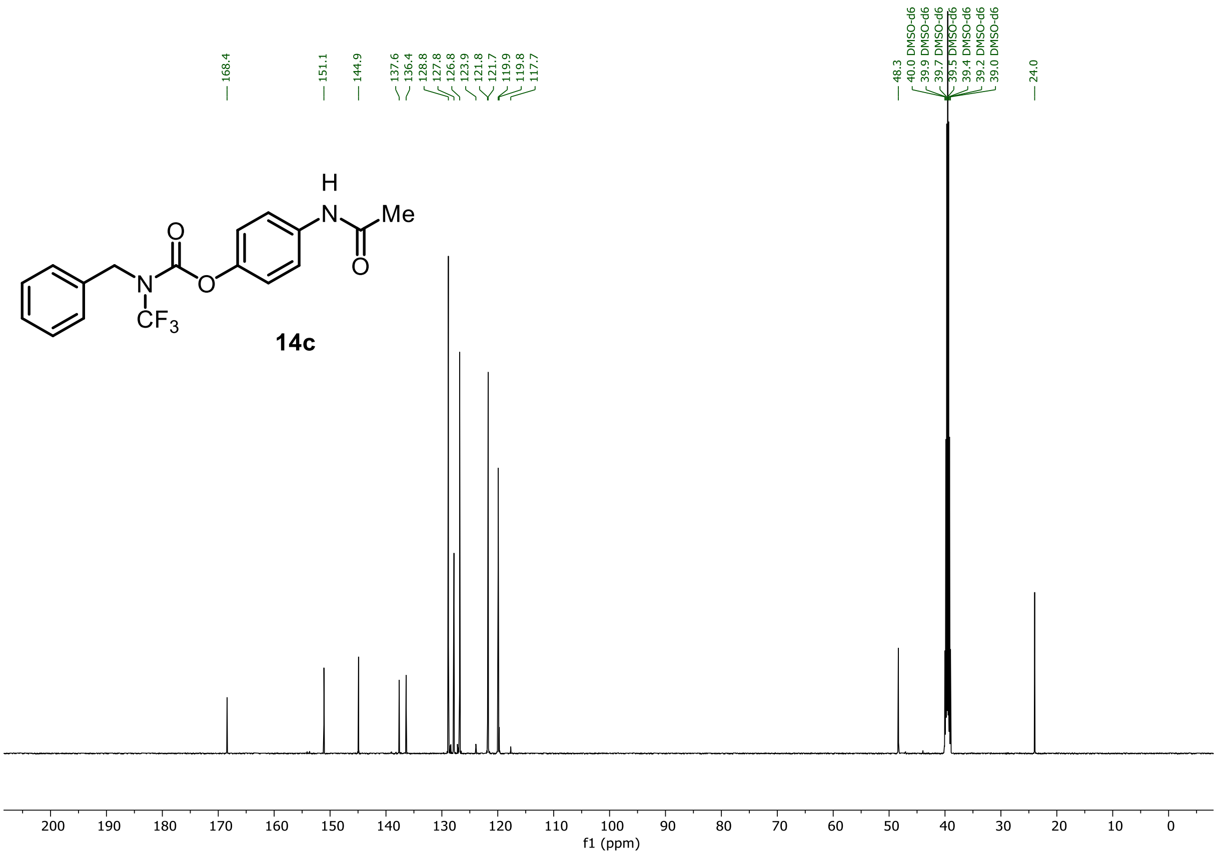

^{13}CNMR spectrum of **14c** run in DMSO-d_6 at 126 MHz.

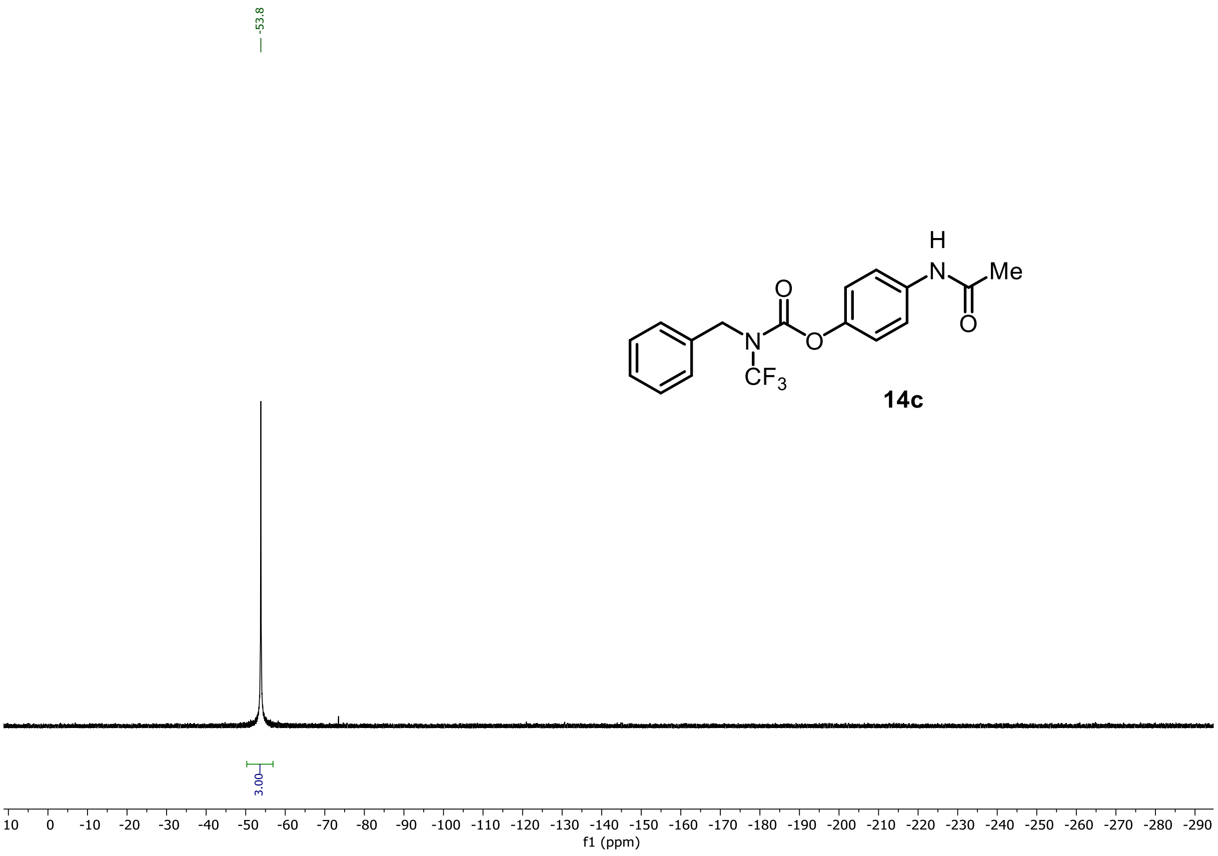

^{19}FNMR spectrum of **14c** run in DMSO-d_6 at 471 MHz.

4-Acetamidophenyl phenyl(trifluoromethyl)carbamate (14d)

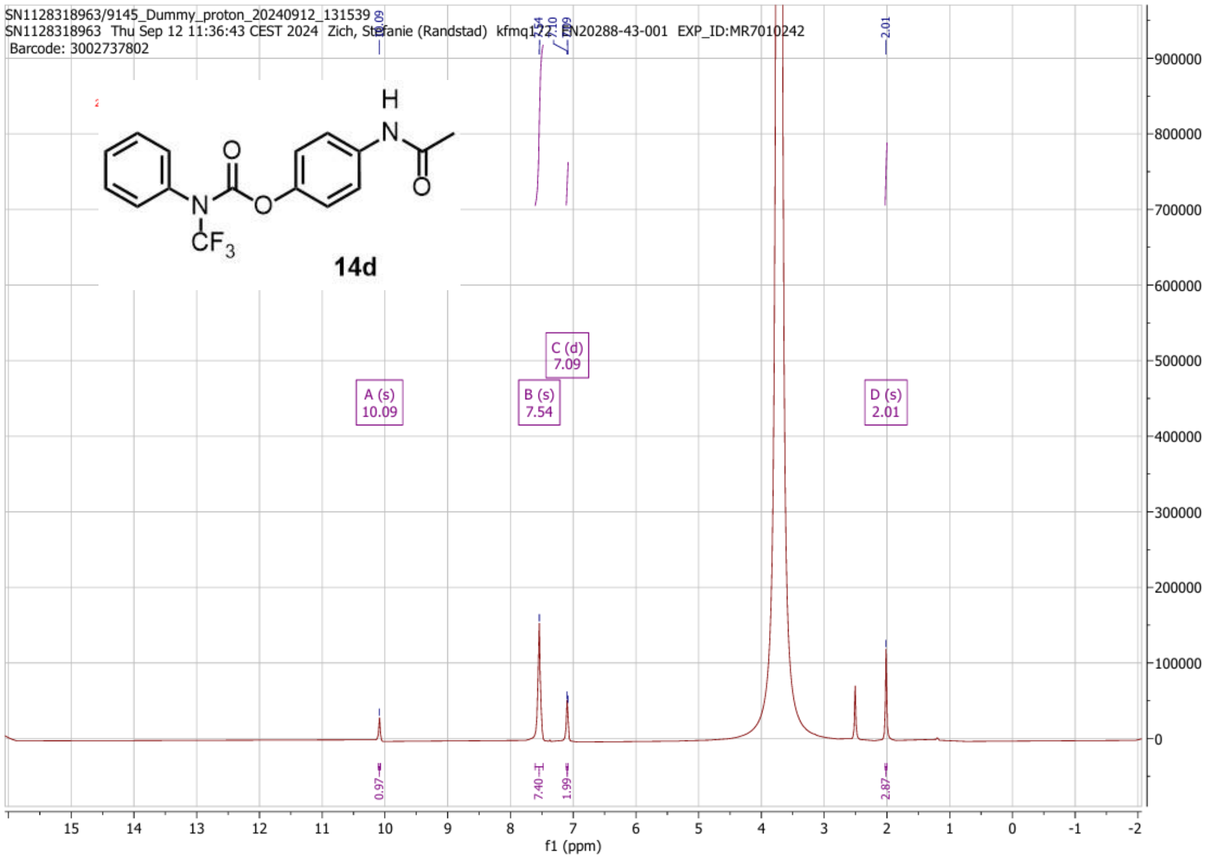

^{1}HNMR spectrum of **14d** run in DMSO-d_6 at 500 MHz.

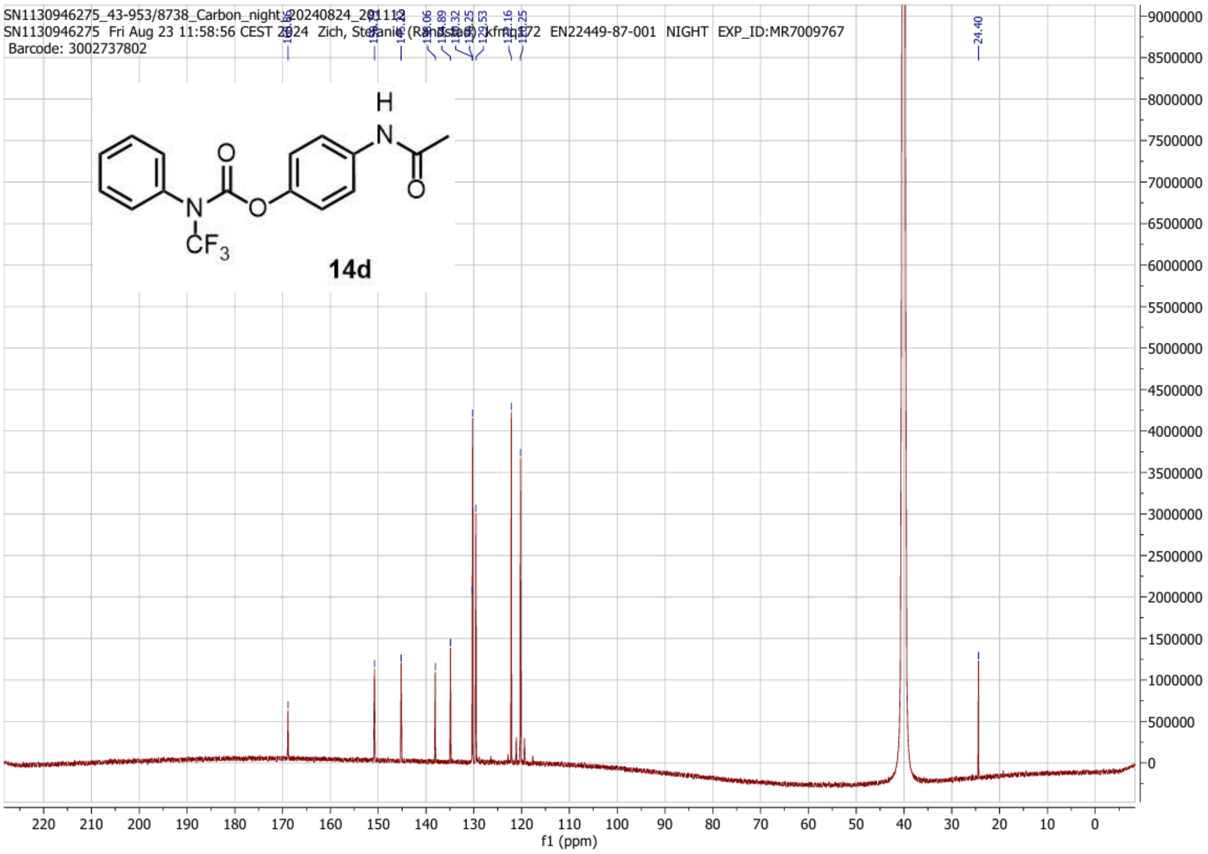

^{13}CNMR spectrum of **14d** run in DMSO-d_6 at 126 MHz.

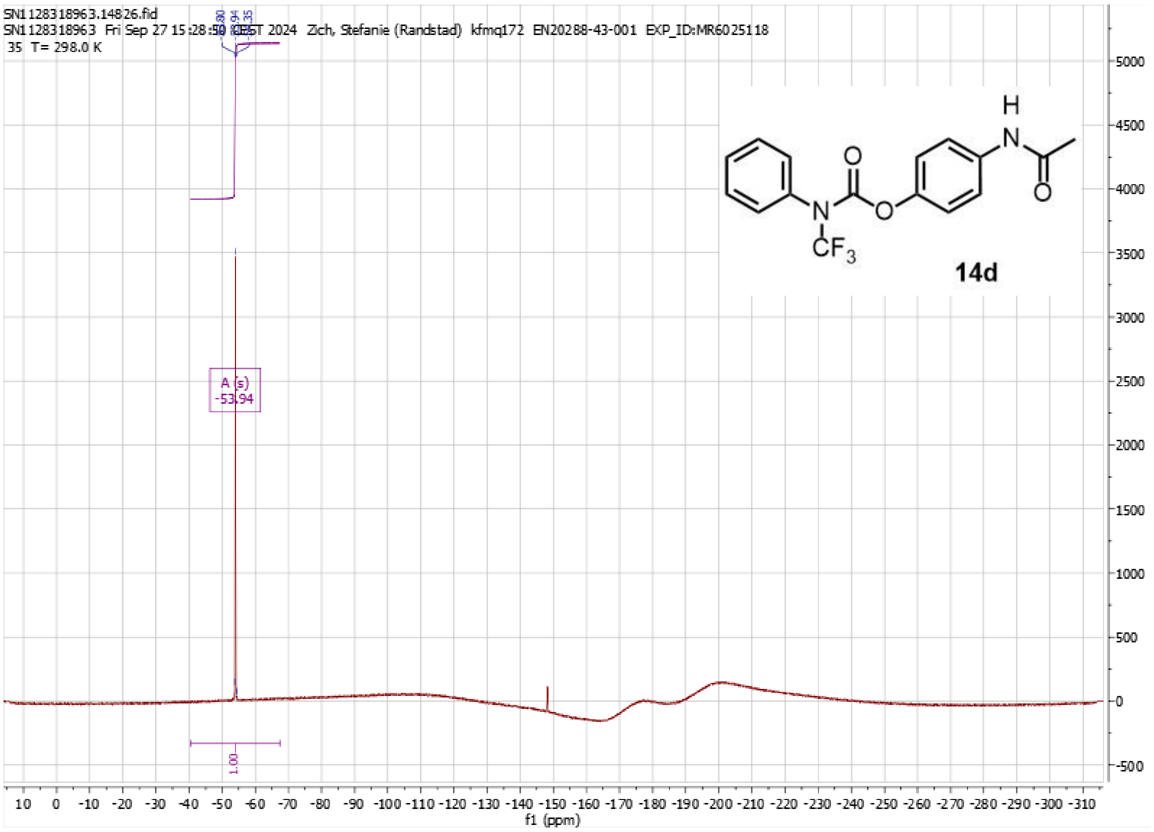

^{19}FNMR spectrum of **14d** run in DMSO-d_6 at 471 MHz.

1-Cyclopropyl-6-fluoro-7-(4-(methyl(trifluoromethyl)carbamoyl)piperazin-1-yl)-4-oxo-1,4-dihydroquinoline-3-carboxylic acid (15a)

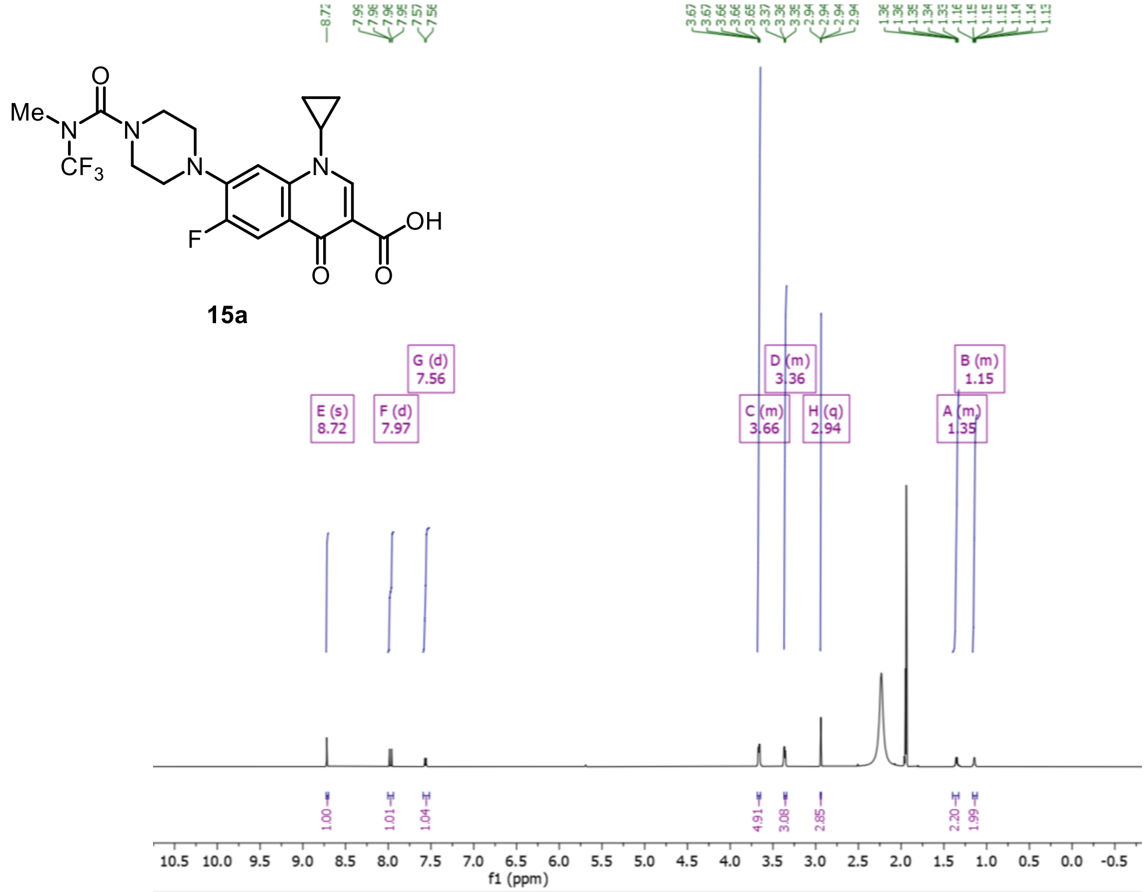

^{1}HNMR spectrum of **15a** run in CD_3CN at 500 MHz.

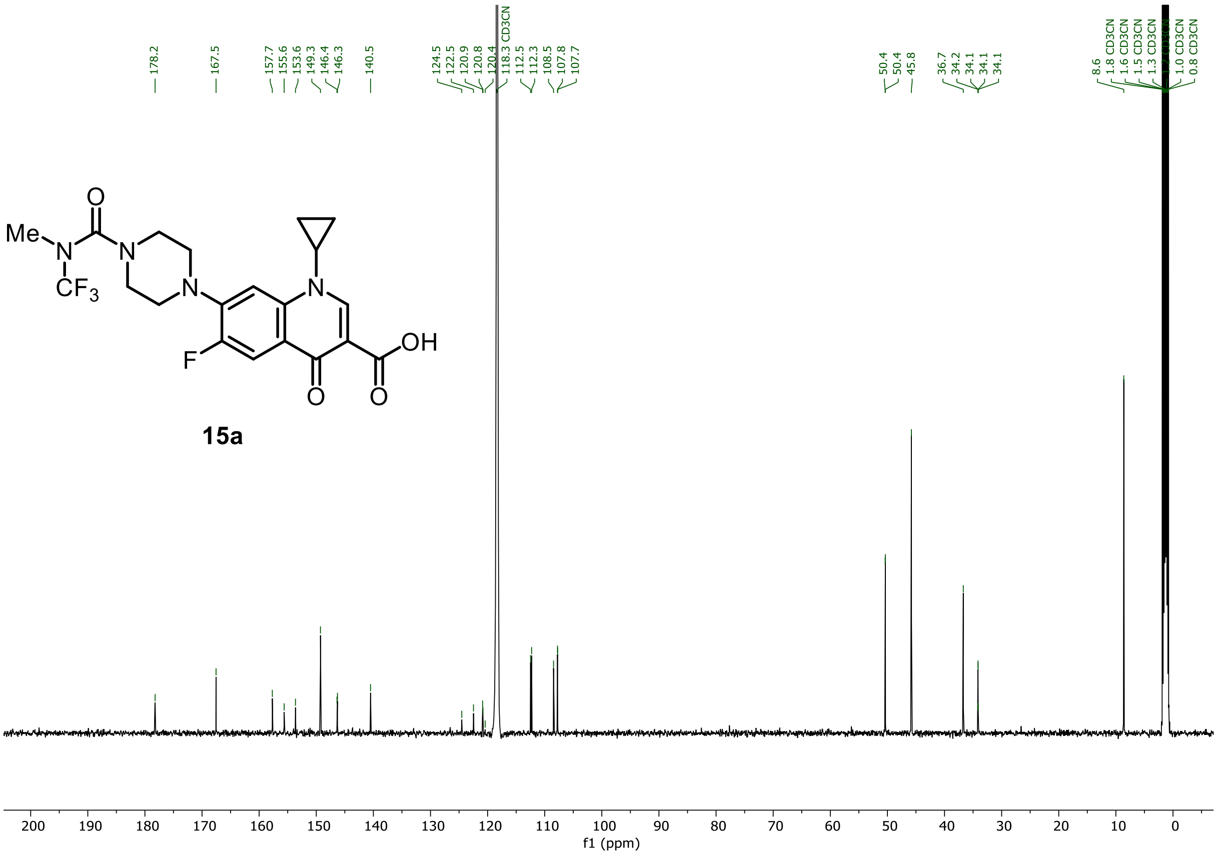

^{13}CNMR spectrum of **15a** run in CD_3CN at 126 MHz.

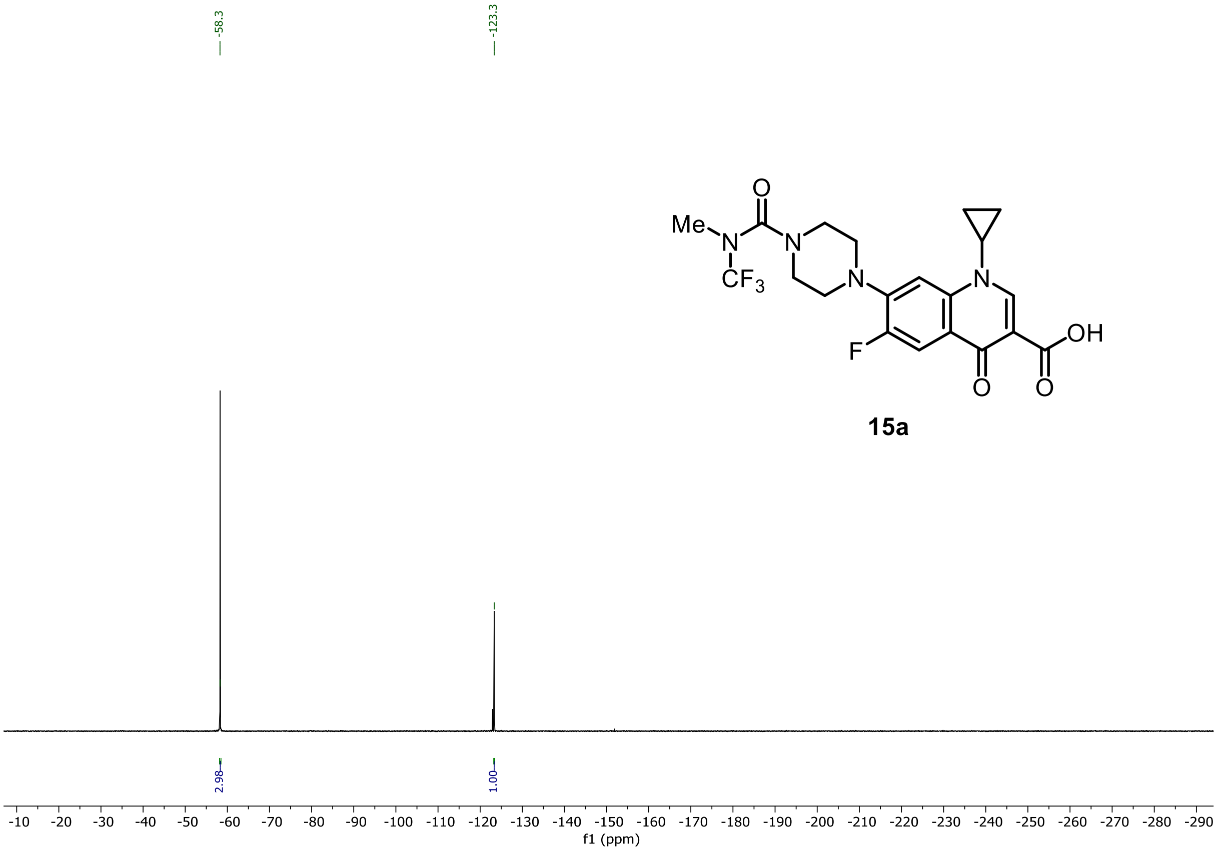

^{19}FNMR spectrum of **15a** run in CD_3CN at 470 MHz.

1-Cyclopropyl-7-(4-(cyclopropyl(trifluoromethyl)carbamoyl)piperazin-1-yl)-6-fluoro-4-oxo-1,4-dihydroquinoline-3-carboxylic acid (15b)

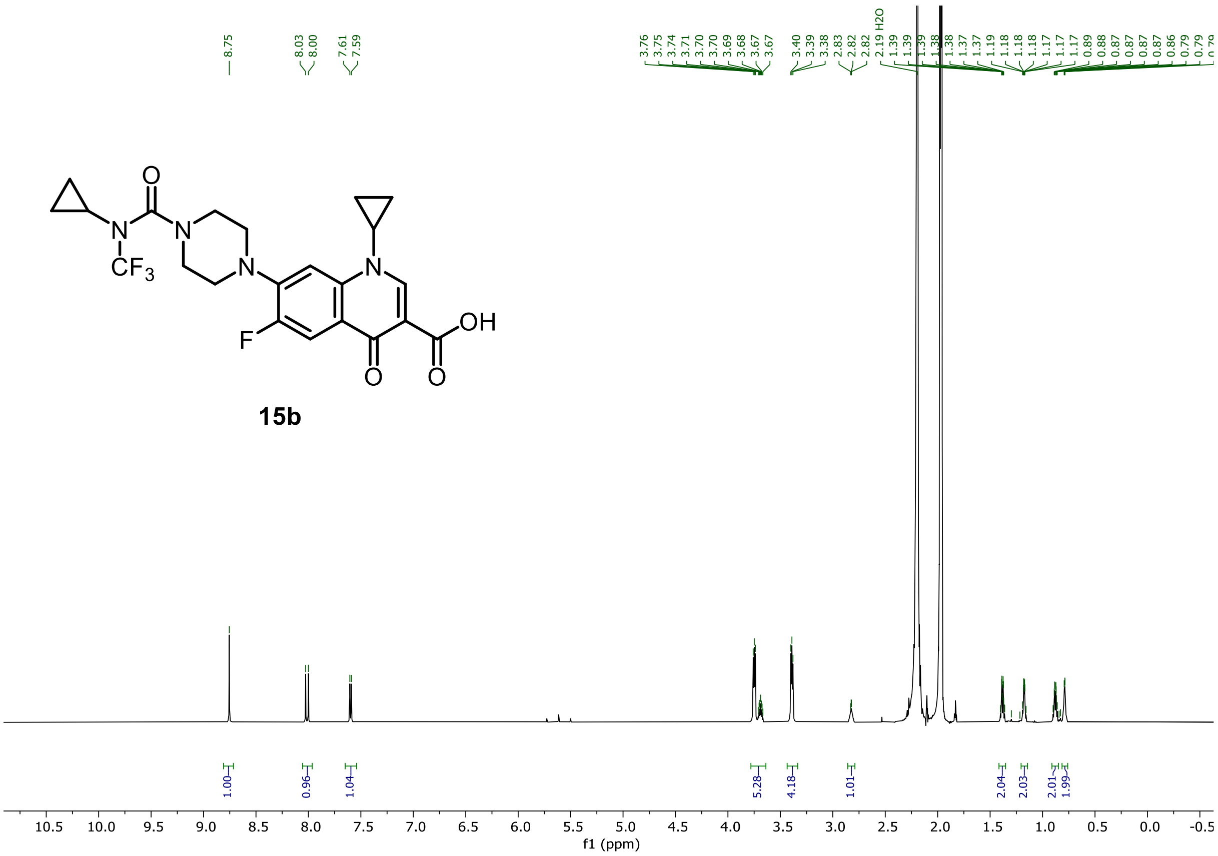

^{1}HNMR spectrum of **15b** run in CD_3CN at 500 MHz.

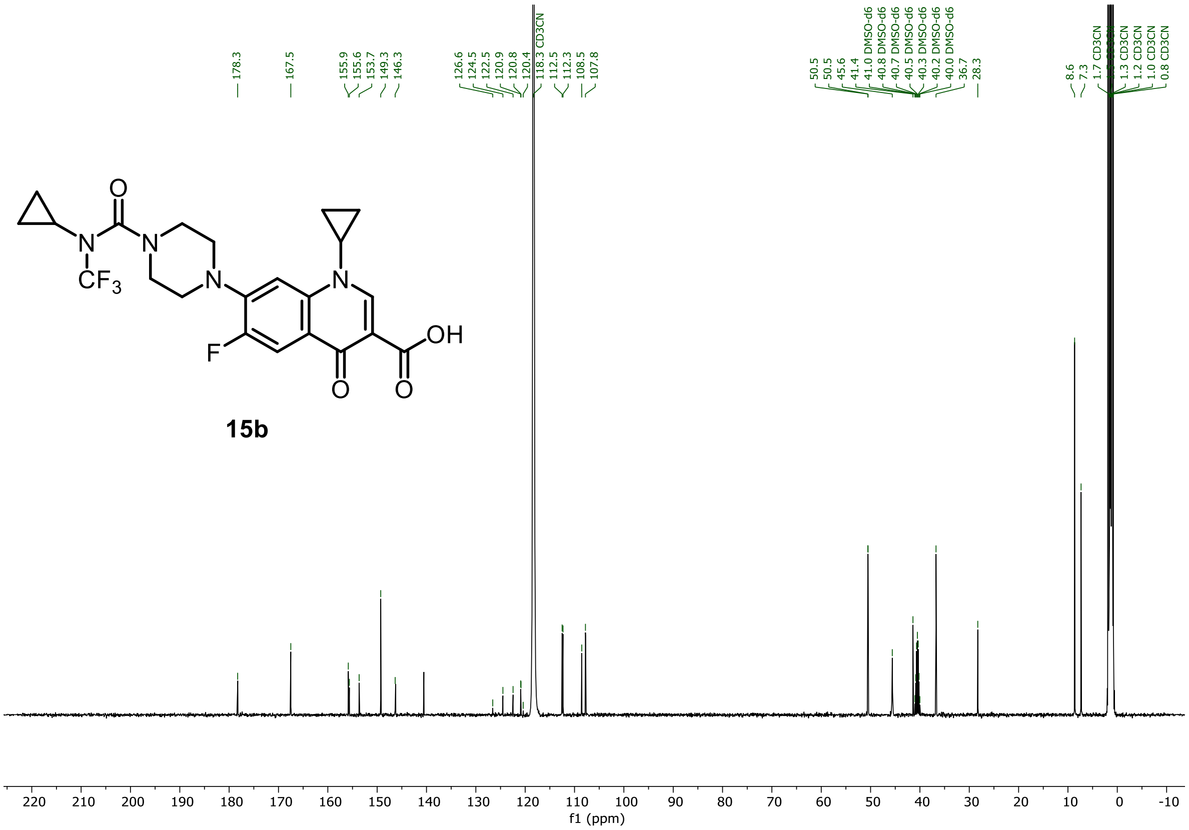

^{13}CNMR spectrum of **15b** run in CD_3CN at 126 MHz.

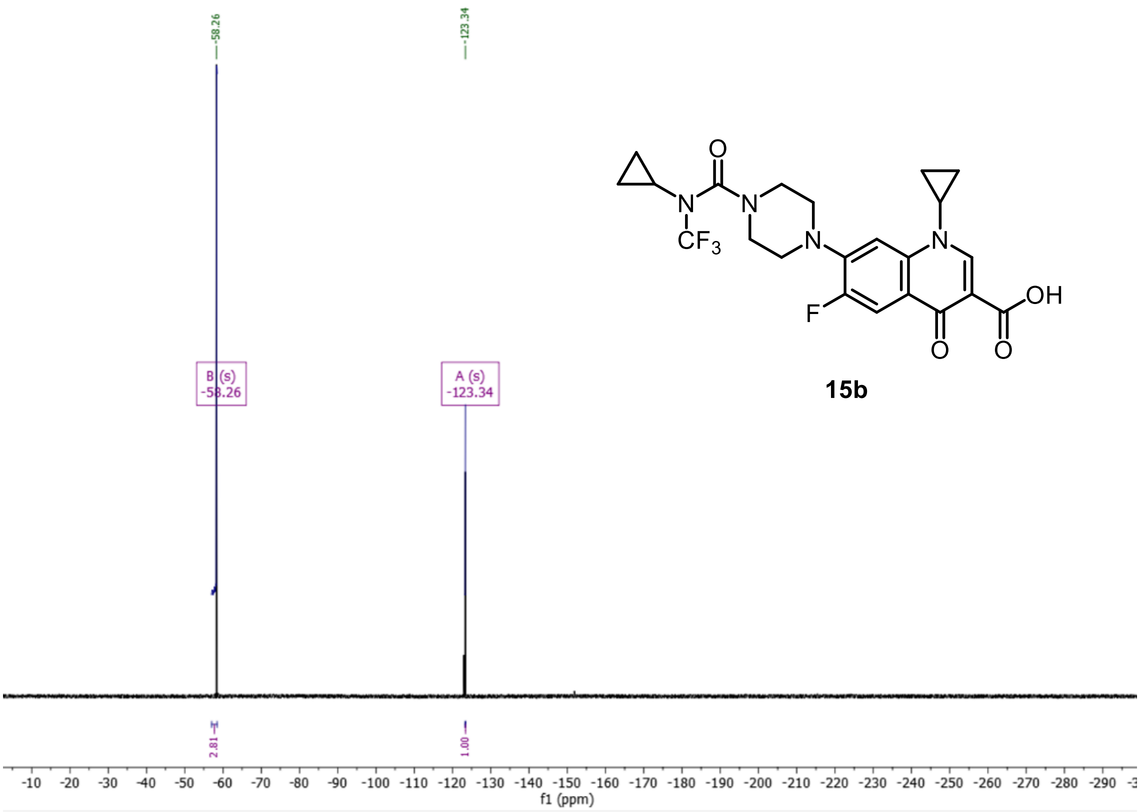

^{19}FNMR spectrum of **15b** run in CD_3CN at 470 MHz.

7-(4-(Benzyl(trifluoromethyl)carbamoyl)piperazin-1-yl)-1-cyclopropyl-6-fluoro-4-oxo-1,4-dihydroquinoline-3-carboxylic acid (15c)

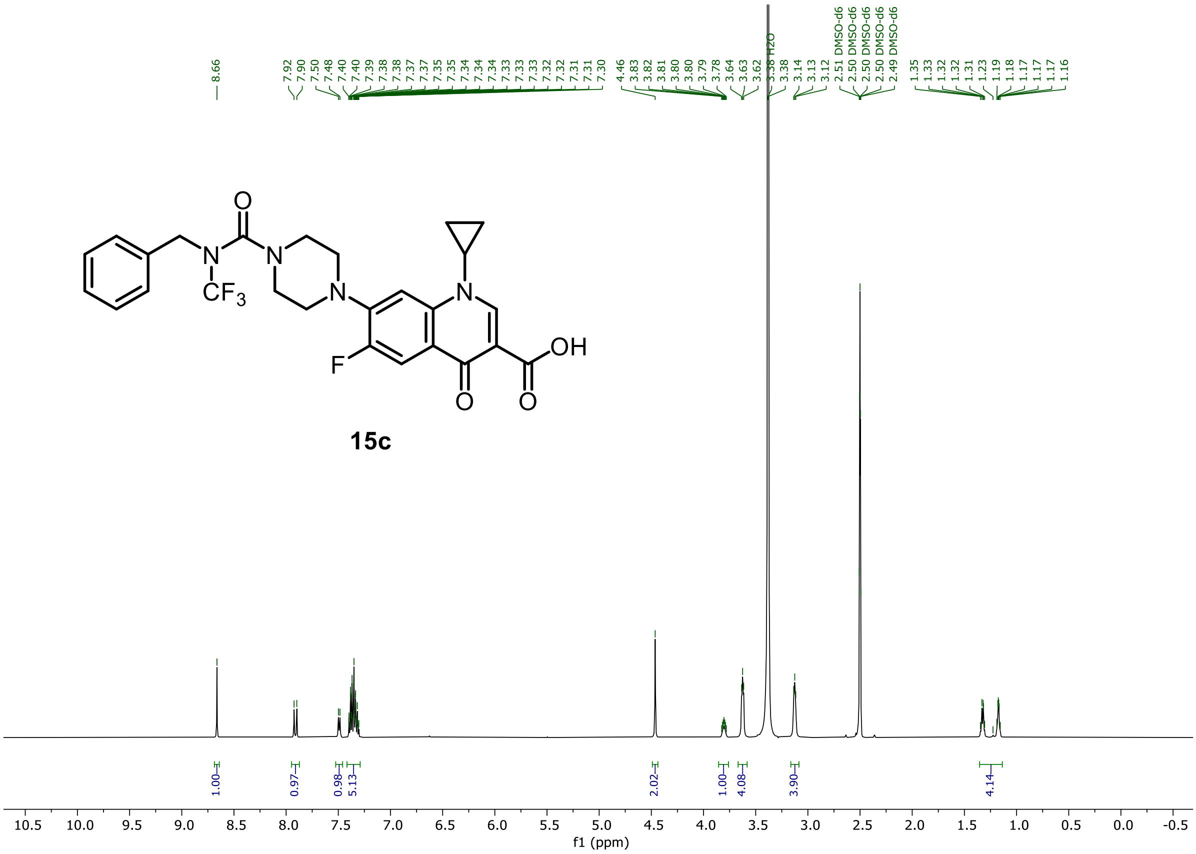

^{1}H NMR spectrum of **15c** run in DMSO-d_6 at 500 MHz.

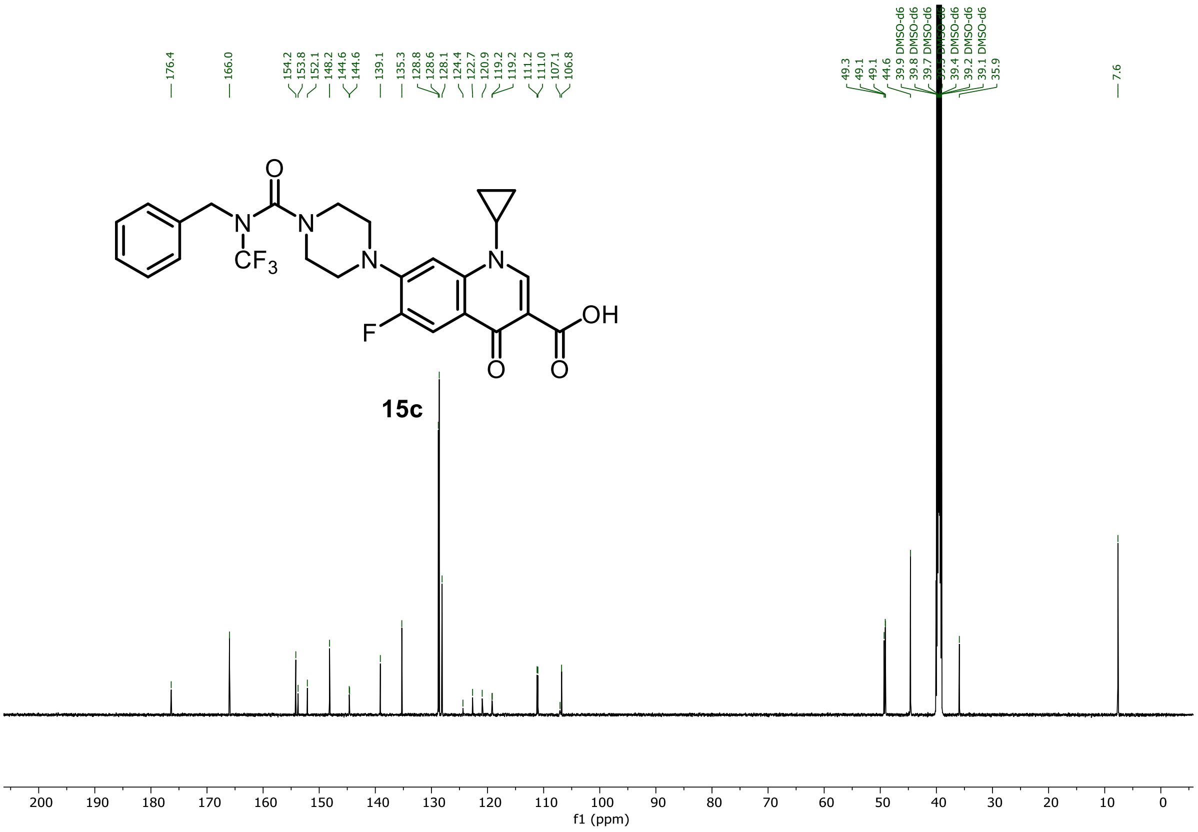

^{13}CNMR spectrum of **15c** run in DMSO-d_6 at 126 MHz.

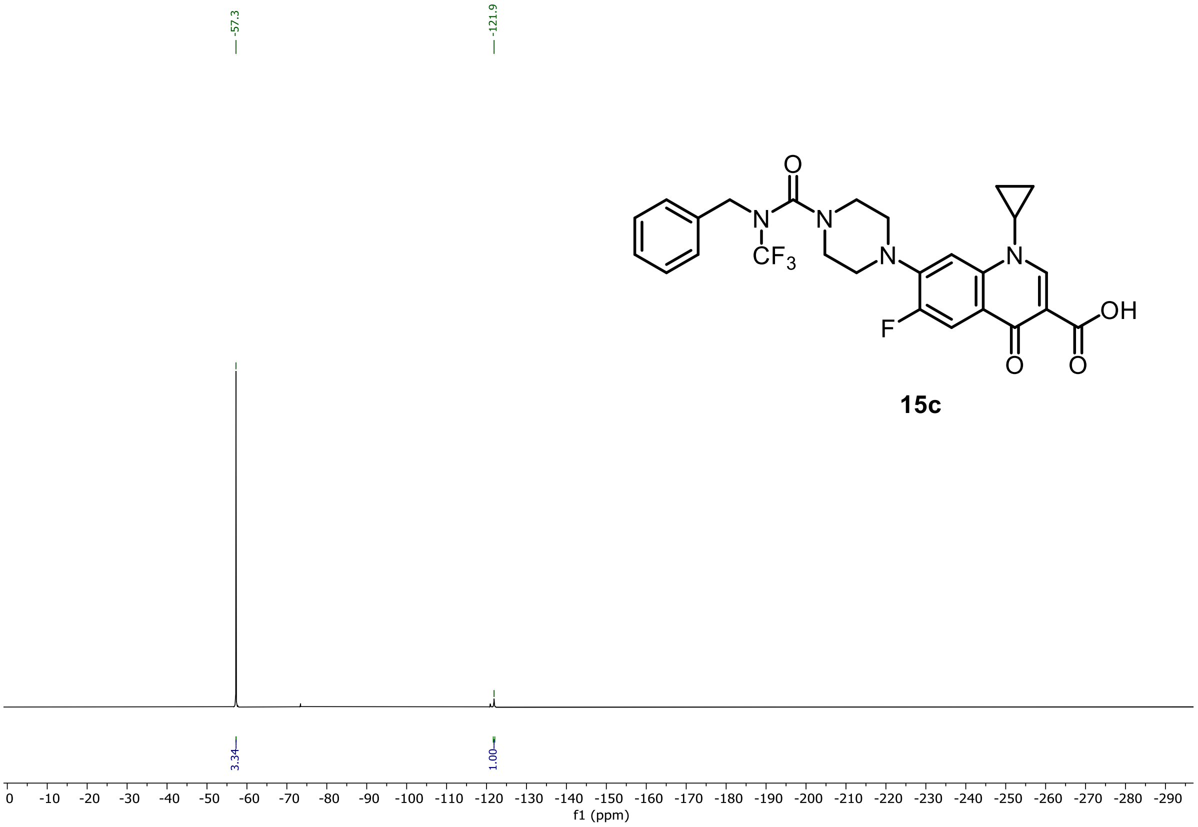

^{19}FNMR spectrum of **15c** run in DMSO-d_6 at 470 MHz.

1-Cyclopropyl-6-fluoro-4-oxo-7-(4-(phenyl(trifluoromethyl)carbamoyl)piperazin-1-yl)-1,4-dihydroquinoline-3-carboxylic acid (15d)

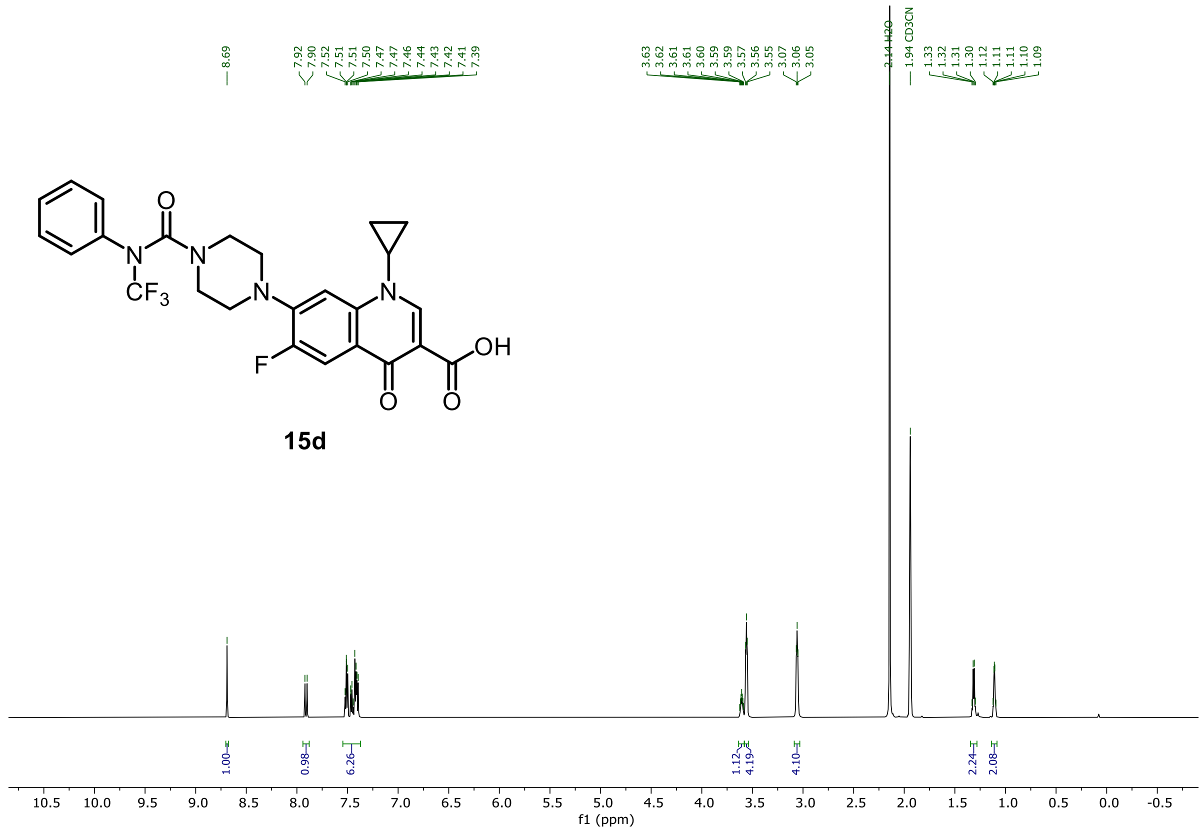

^{1}HNMR spectrum of **15d** run in CD_3CN at 600 MHz.

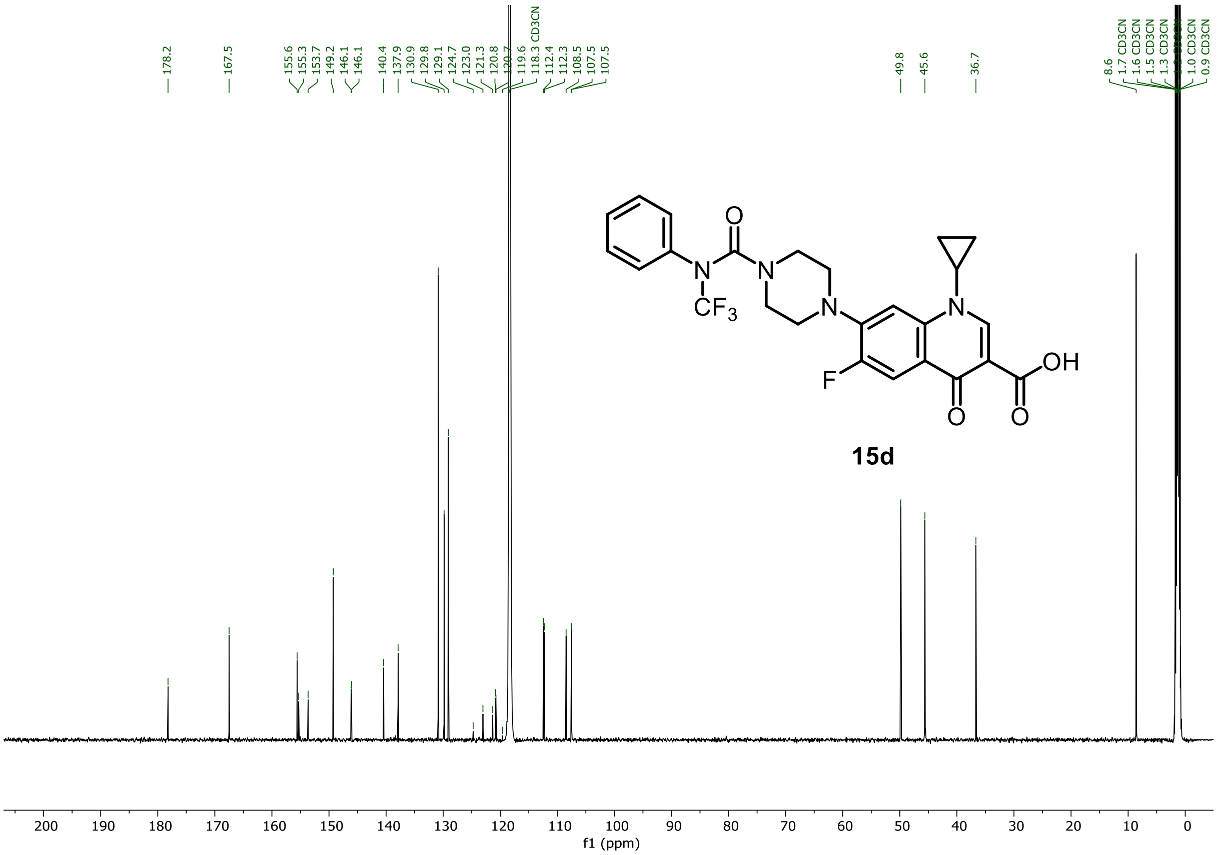

^{13}CNMR spectrum of **15d** run in CD_3CN at 151 MHz.

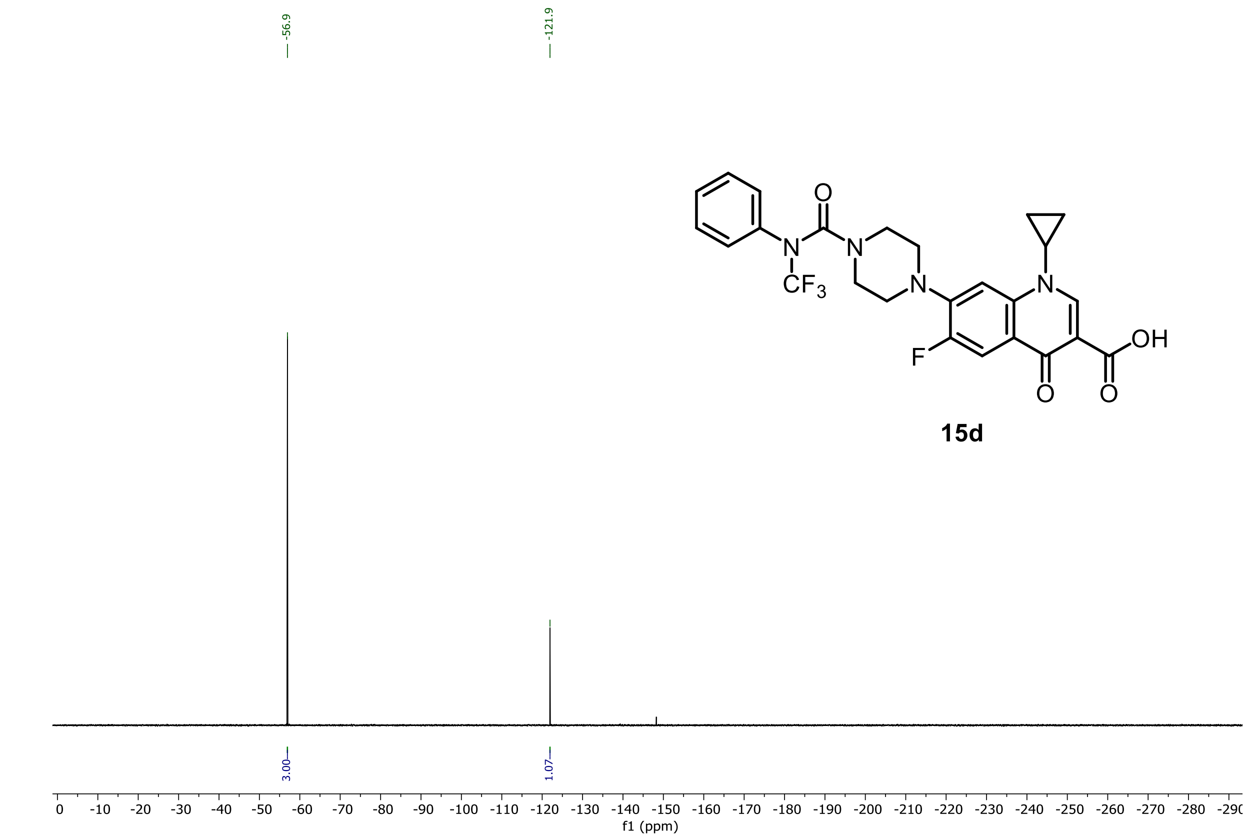

^{19}FNMR spectrum of **15d** run in CD_3CN at 470 MHz.

4-((4-Ethoxy-3-(1-methyl-7-oxo-3-propyl-6,7-dihydro-1*H*-pyrazolo[4,3-*d*]pyrimidin-5-yl)phenyl)sulfonyl)-*N*-methyl-*N*-(trifluoromethyl)piperazine-1-carboxamide (16a)

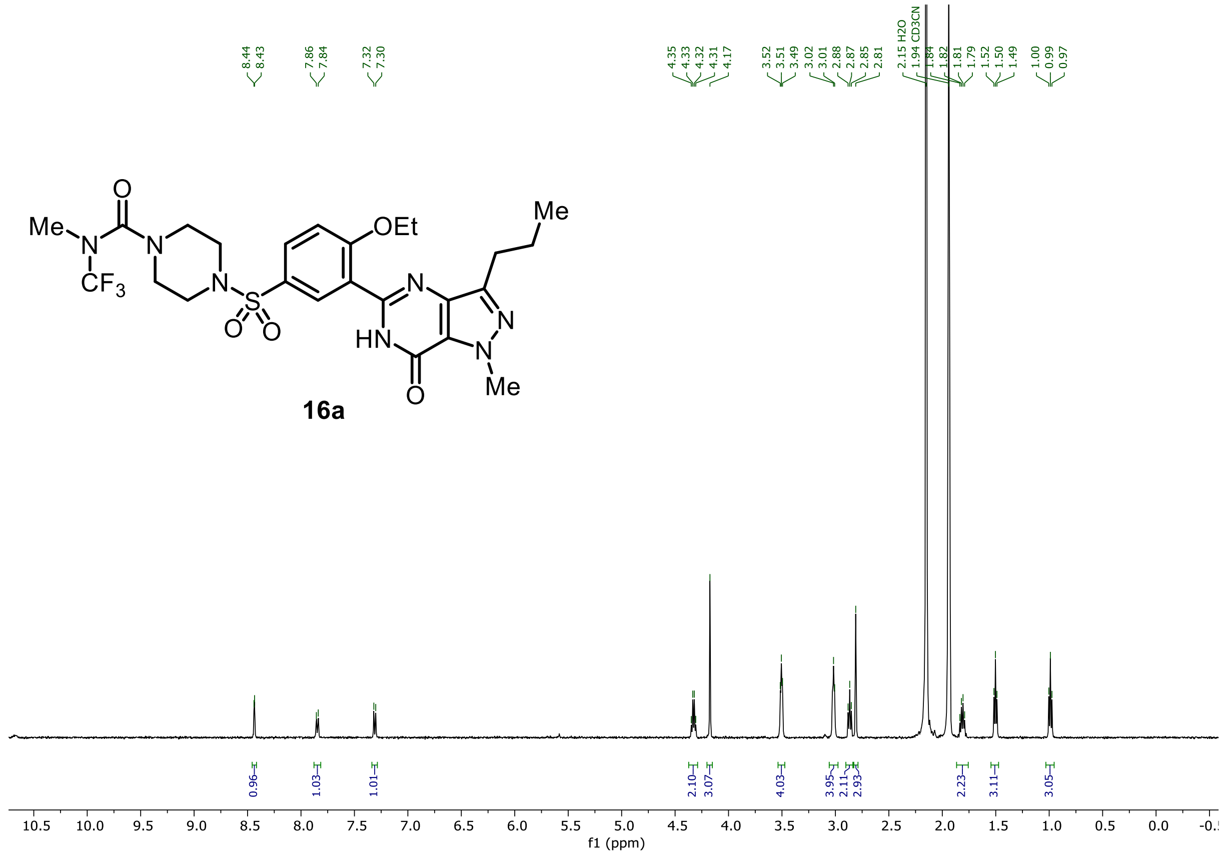

^{1}HNMR spectrum of **16a** run in CD_3CN at 500 MHz.

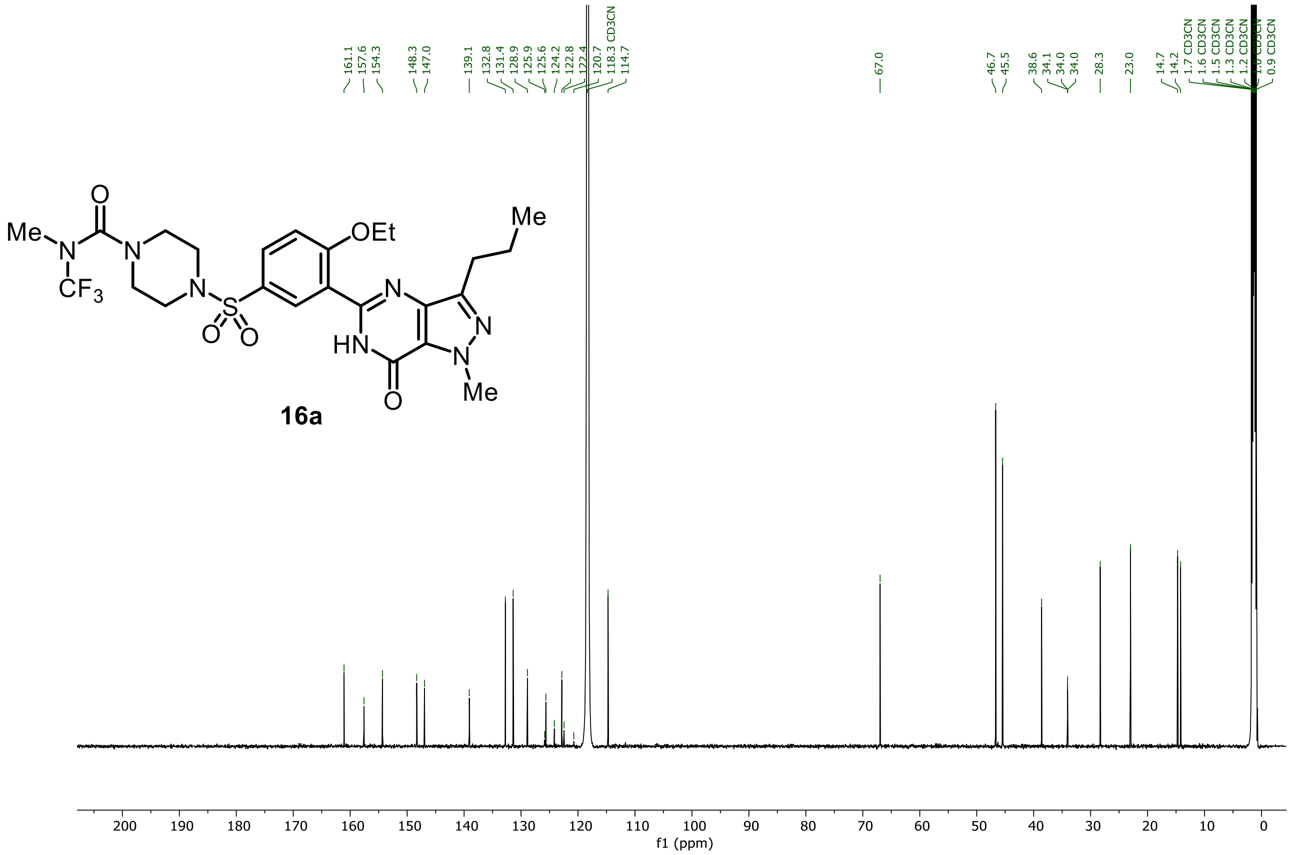

^{13}CNMR spectrum of **16a** run in CD_3CN at 151 MHz.

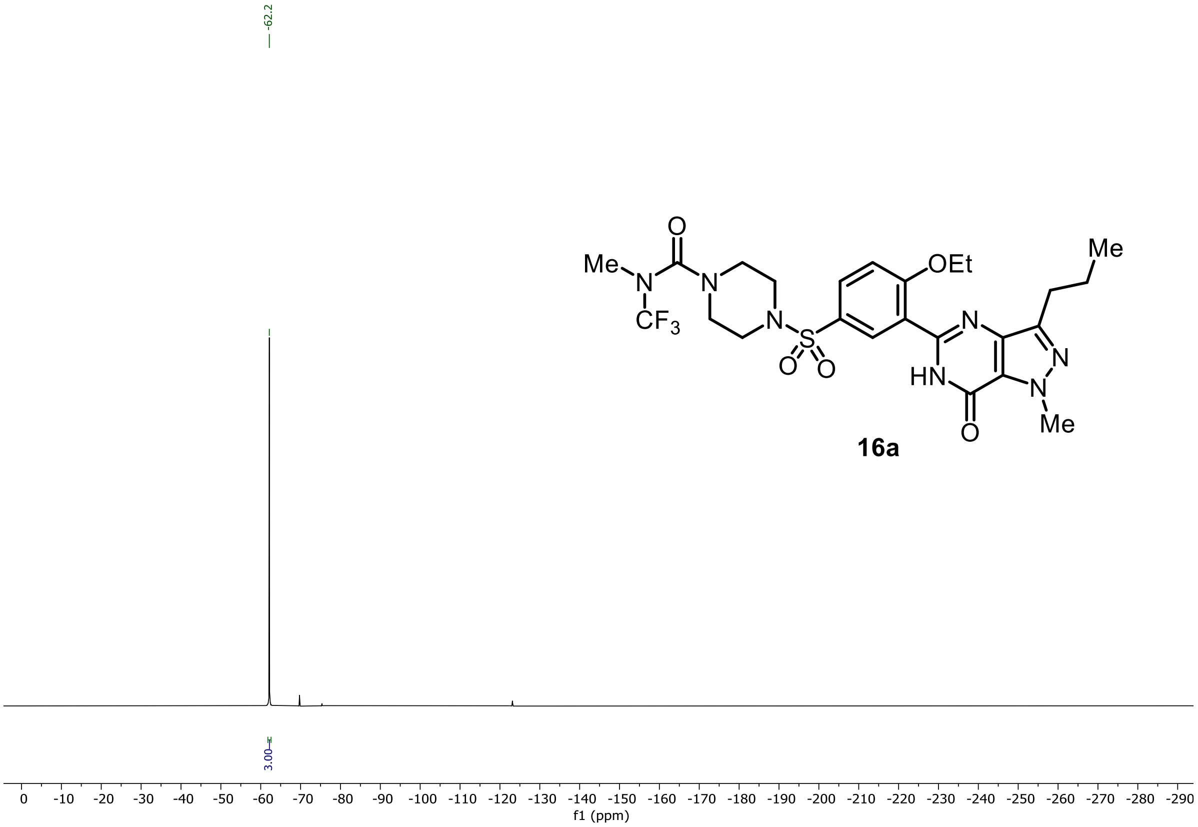

^{19}FNMR spectrum of **16a** run in CD_3CN at 470 MHz.

***N*-Cyclopropyl-4-((4-ethoxy-3-(1-methyl-7-oxo-3-propyl-6,7-dihydro-1*H*-pyrazolo[4,3-*d*]pyrimidin-5-yl)phenyl)sulfonyl)-*N*-(trifluoromethyl)piperazine-1-carboxamide (16b)**

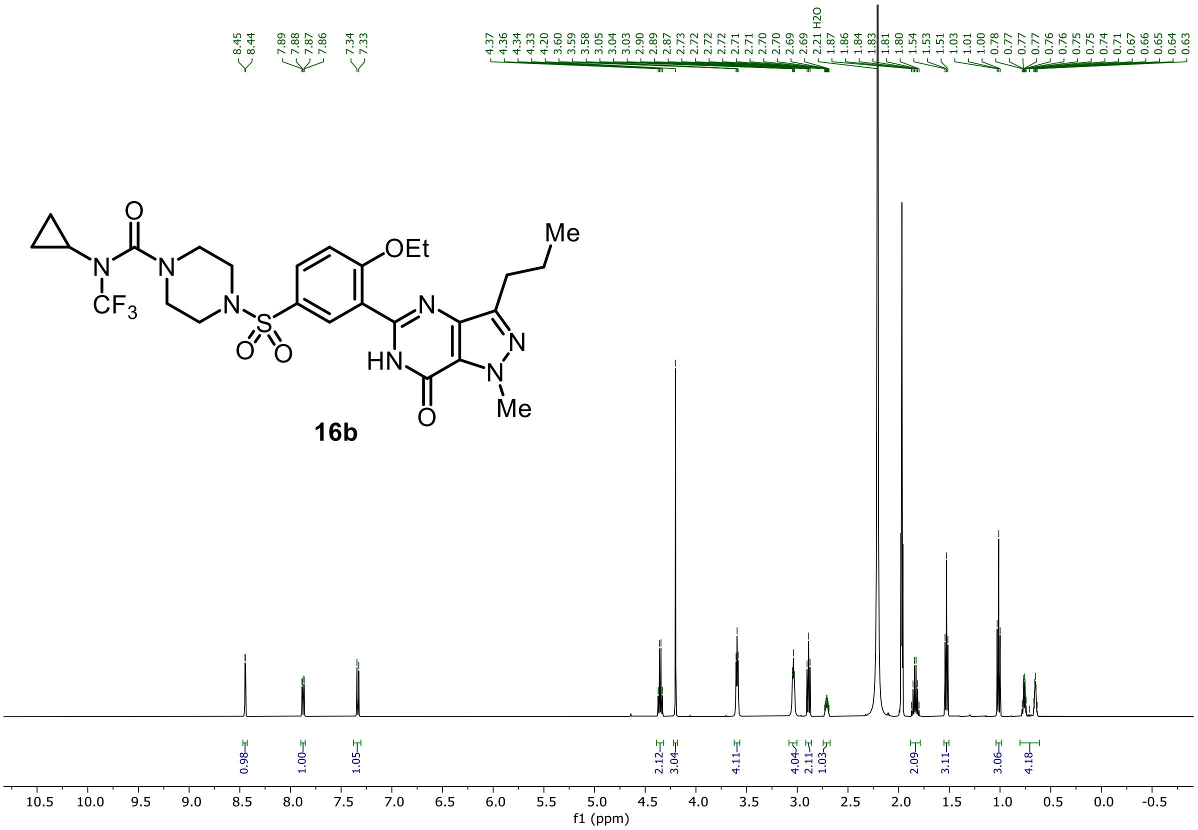

^{1}HNMR spectrum of **16b** run in CD_3CN at 500 MHz.

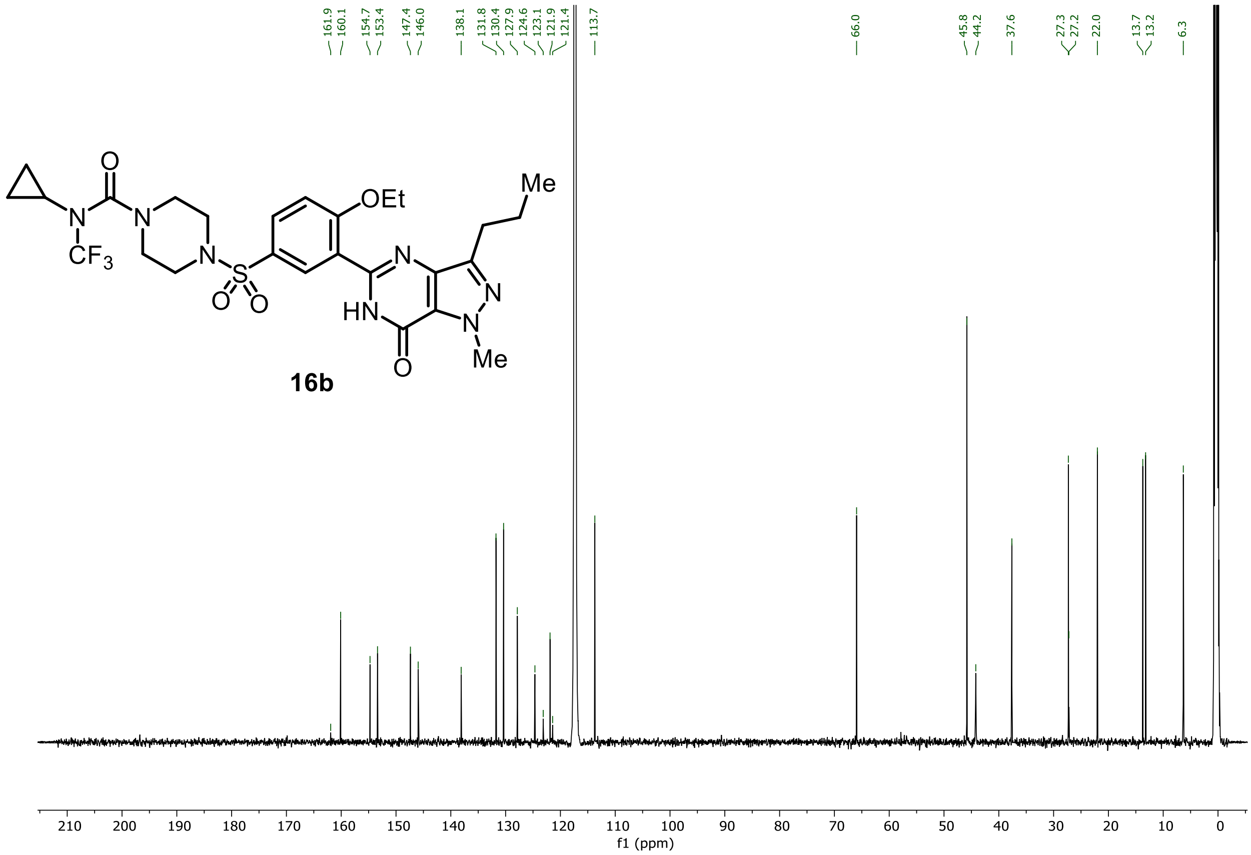

^{13}CNMR spectrum of **16b** run in CD_3CN at 151 MHz.

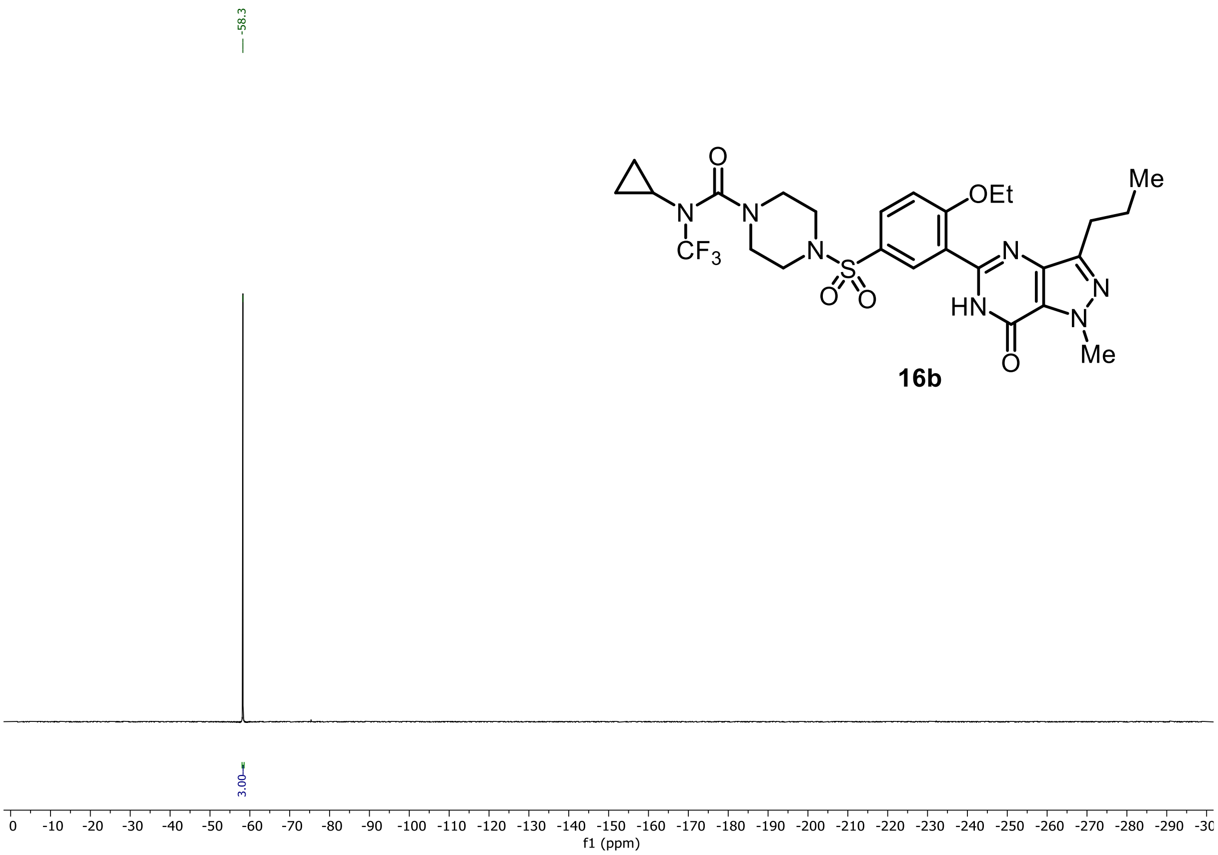

16b

^{19}FNMR spectrum of **16b** run in CD_3CN at 470 MHz.

_N_-Benzyl-4-((4-ethoxy-3-(1-methyl-7-oxo-3-propyl-6,7-dihydro-1_H_-pyrazolo[4,3-_d_]pyrimidin-5-yl)phenyl)sulfonyl)-_N_-(trifluoromethyl)piperazine-1-carboxamide (16c)

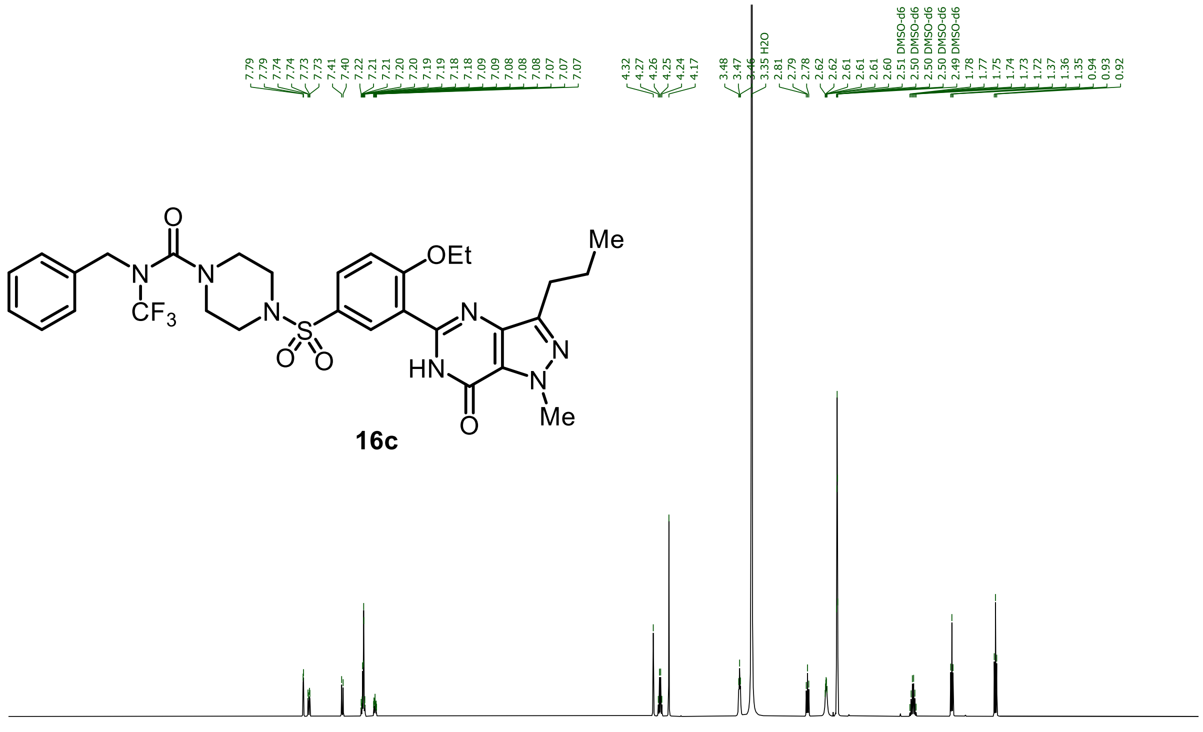

16c

^{1}HNMR spectrum of **16c** run in DMSO-d_6 at 600 MHz.

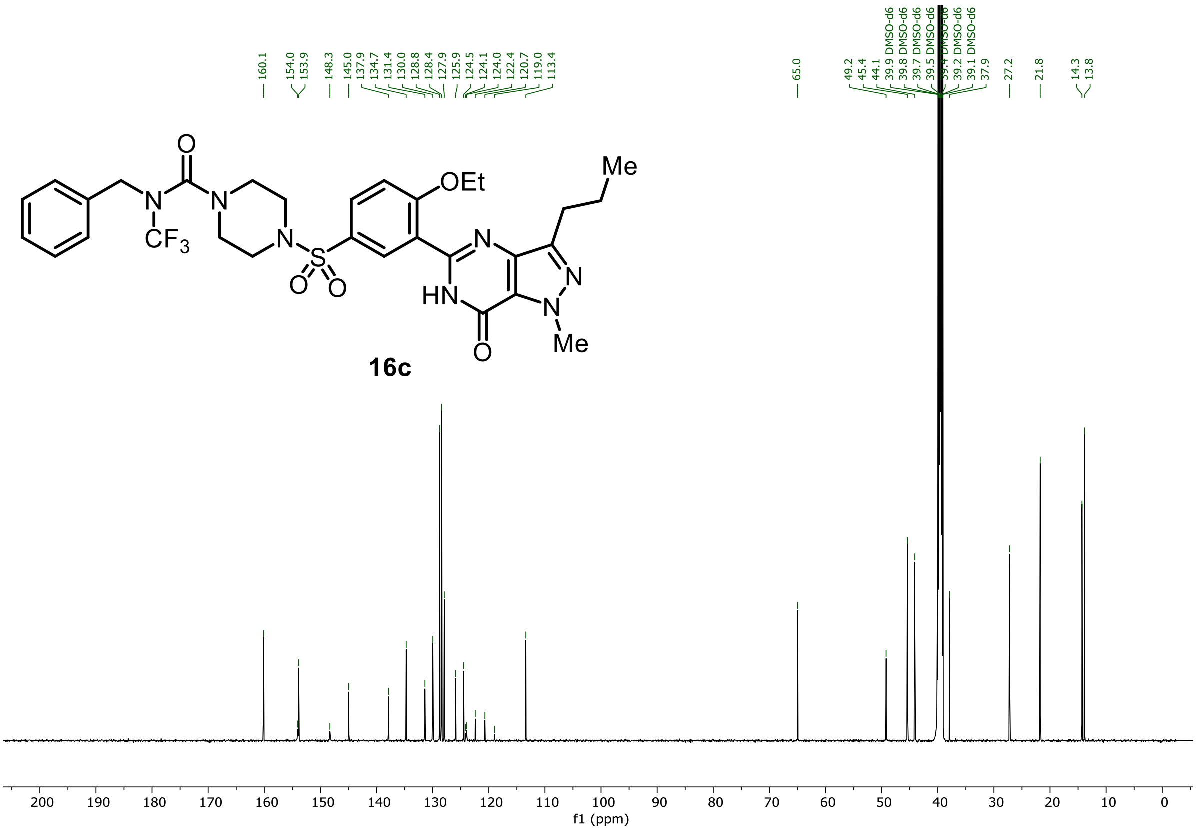

^{13}CNMR spectrum of **16c** run in DMSO-d_6 at 151 MHz.

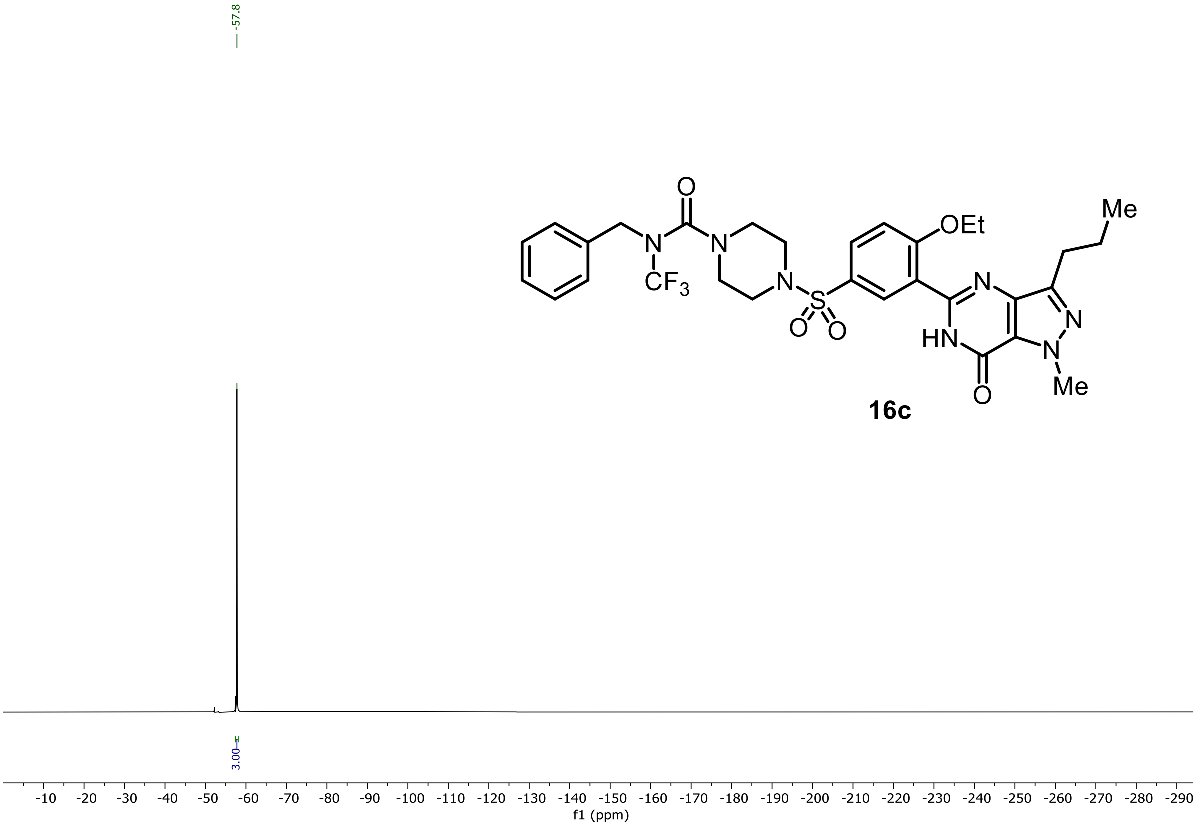

^{19}FNMR spectrum of **16c** run in DMSO-d_6 at 470 MHz.

4-((4-Ethoxy-3-(1-methyl-7-oxo-3-propyl-6,7-dihydro-1*H*-pyrazolo[4,3-*d*]pyrimidin-5-yl)phenyl)sulfonyl)-*N*-phenyl-*N*-(trifluoromethyl)piperazine-1-carboxamide (16d)

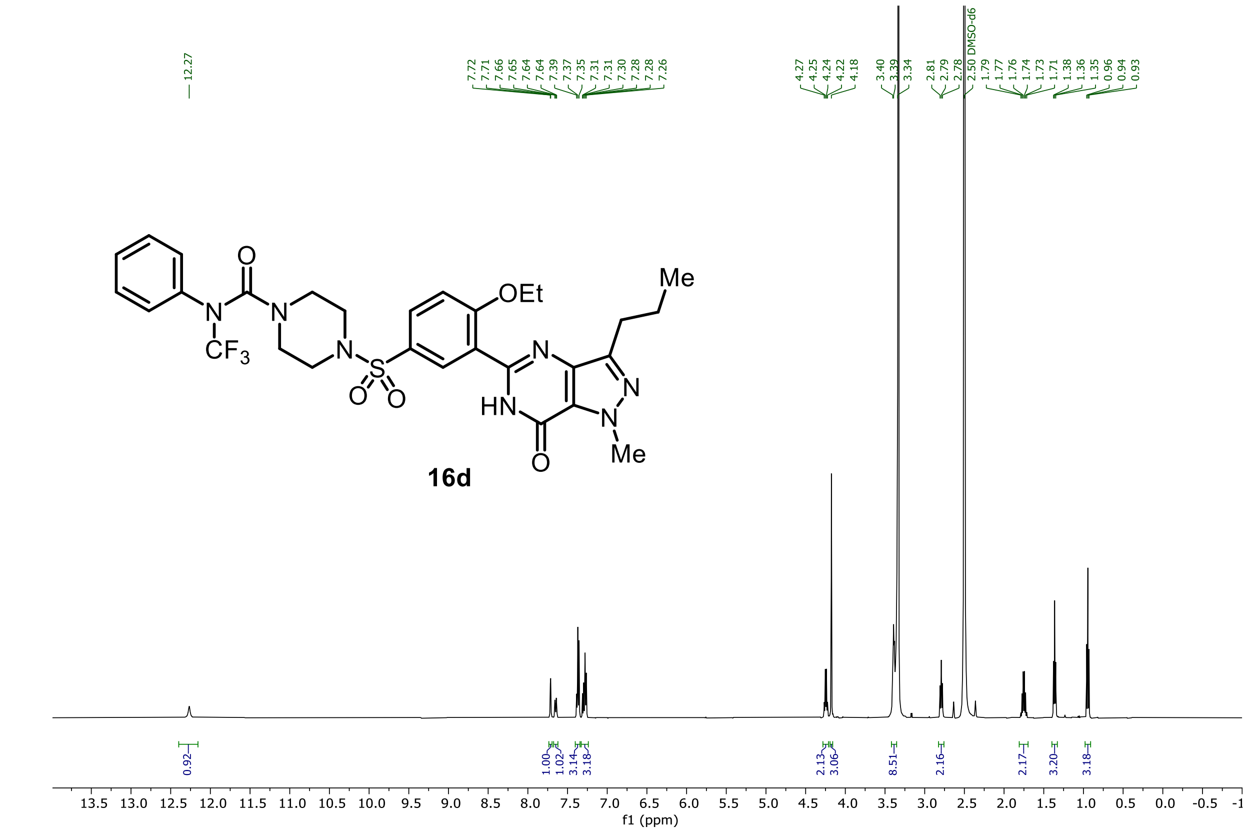

^{1}HNMR spectrum of **16d** run in DMSO-d_6 at 500 MHz.

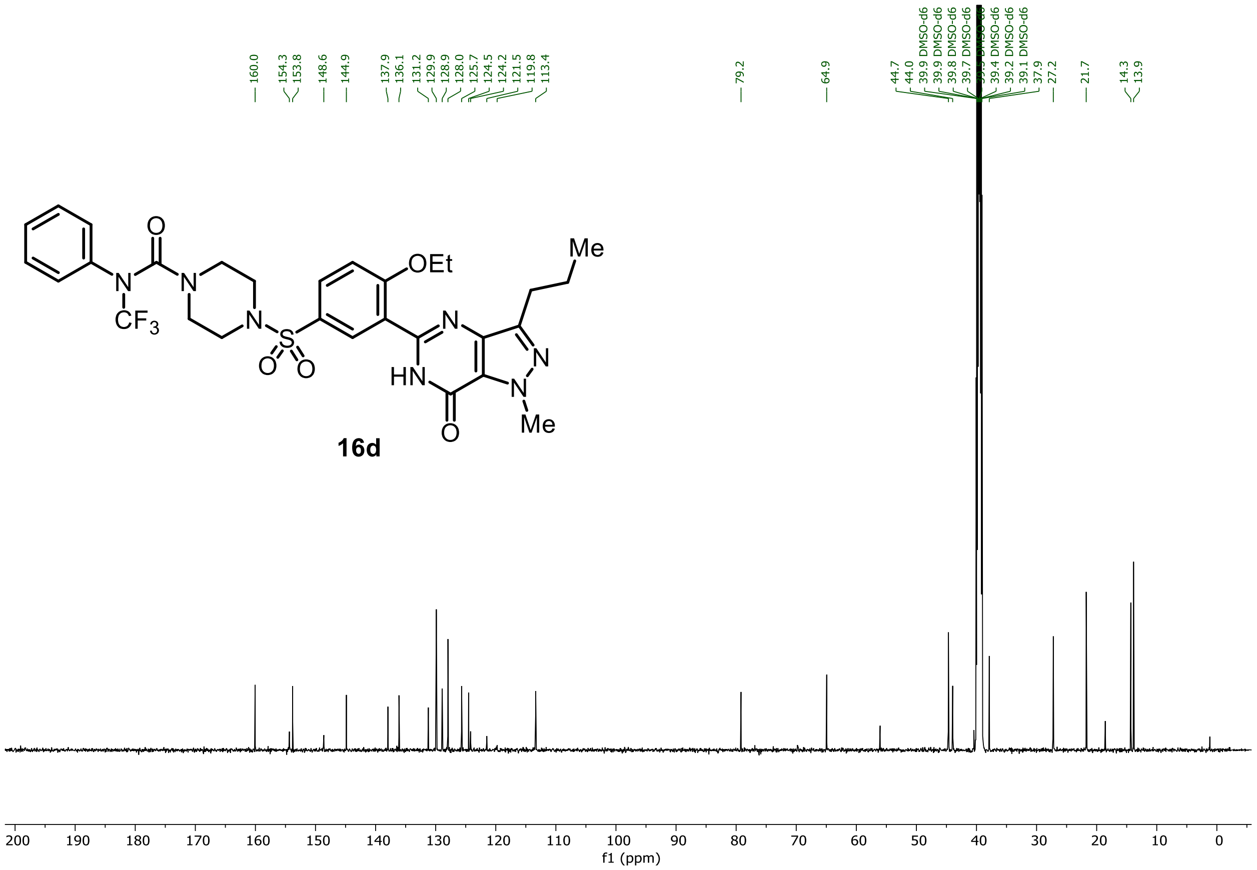

^{13}CNMR spectrum of **16d** run in DMSO-d_6 at 151 MHz.

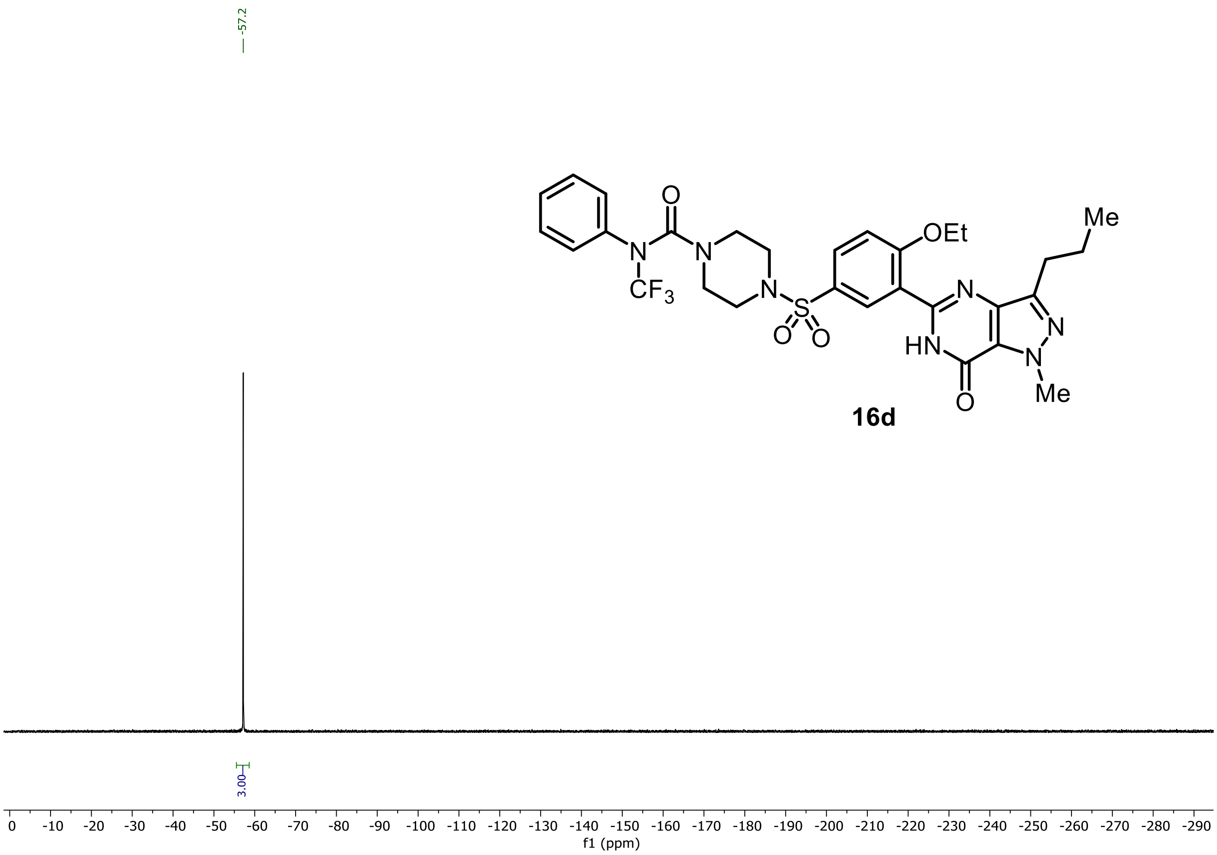

16d

^{19}FNMR spectrum of **16d** run in DMSO-d_6 at 470 MHz.

3.5 *N*-Me and *N*-*i*Pr analogues

(*S*)-*N*-Methyl-*N*-((2-oxo-3-(4-(3-oxomorpholino)phenyl)oxazolidin-5-yl)methyl)acetamide (10e)

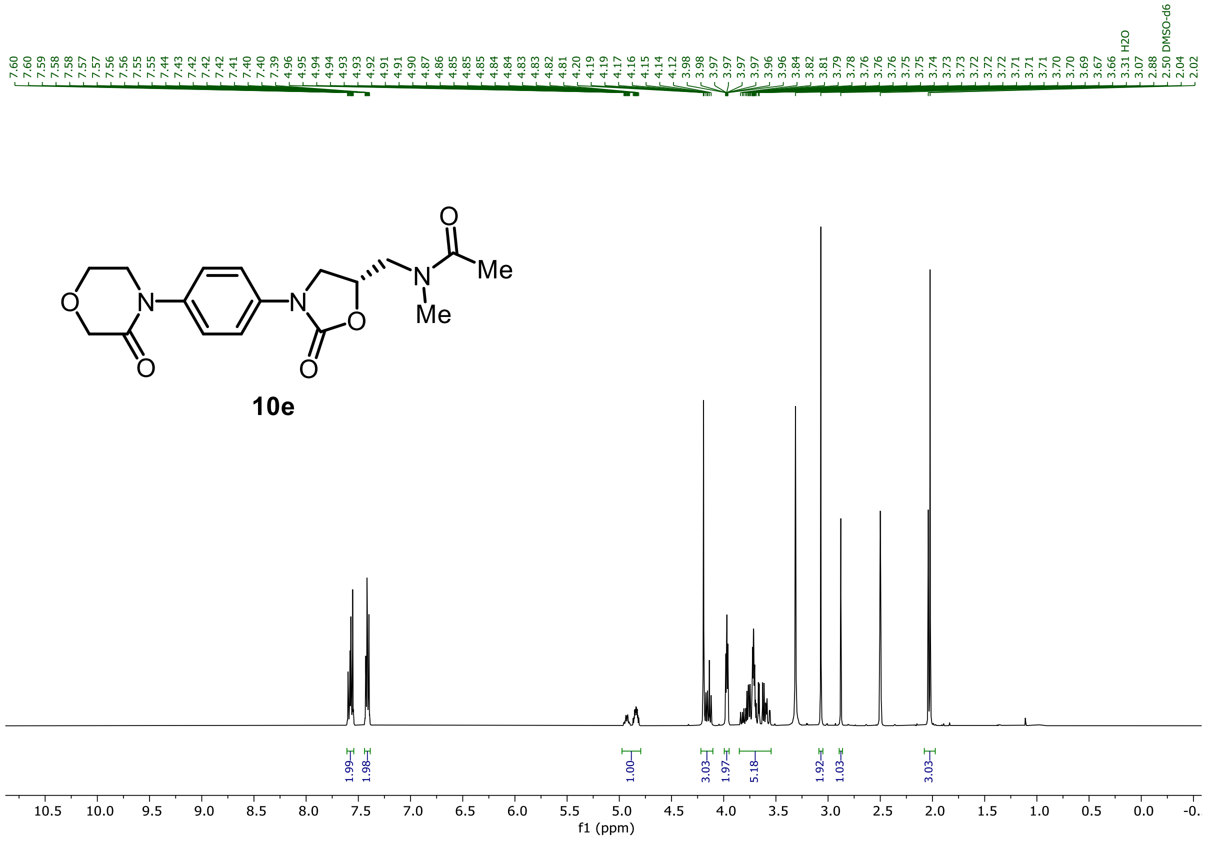

^{1}HNMR spectrum of **10e** run in DMSO-d_6 at 500 MHz.

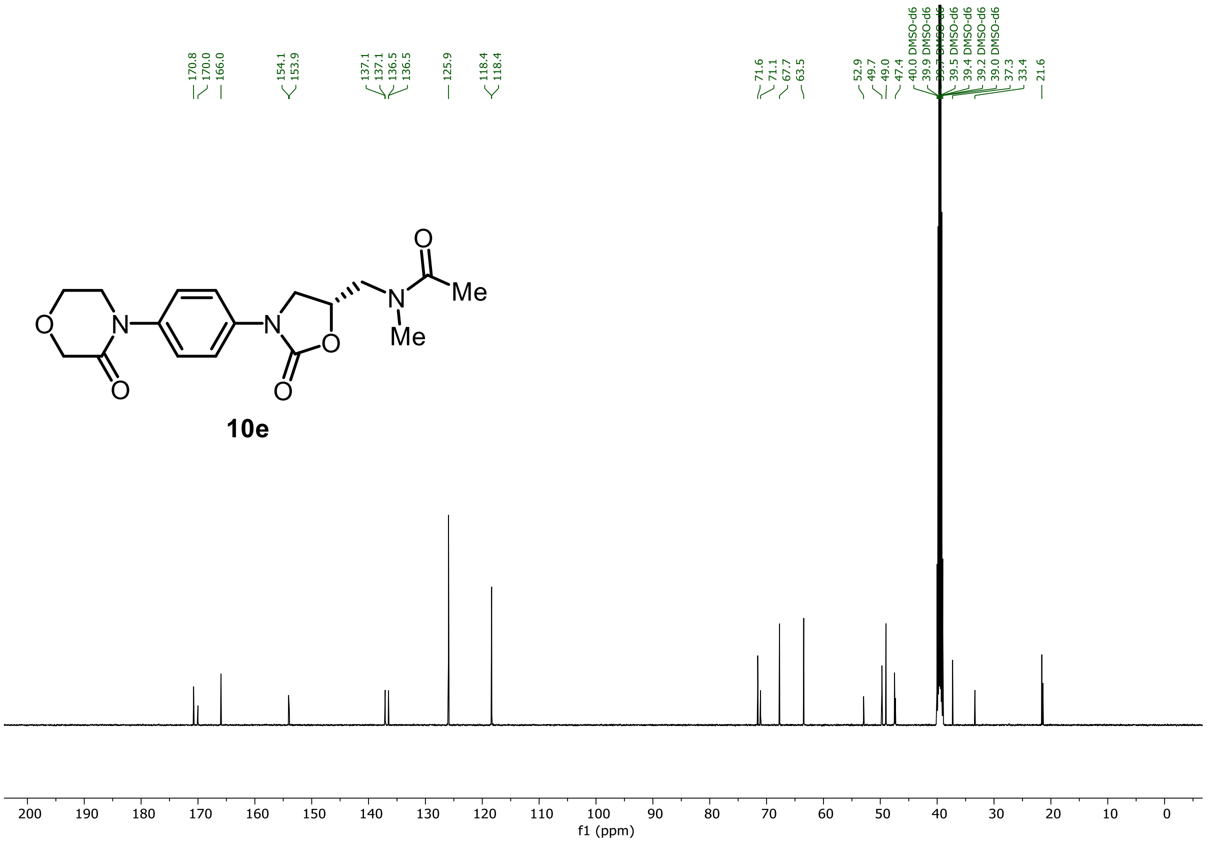

^{13}CNMR spectrum of **10e** run in DMSO-d_6 at 126 MHz.

(*S*)-*N*-Isopropyl-*N*-((2-oxo-3-(4-(3-oxomorpholino)phenyl)oxazolidin-5-yl)methyl)acetamide (10f)

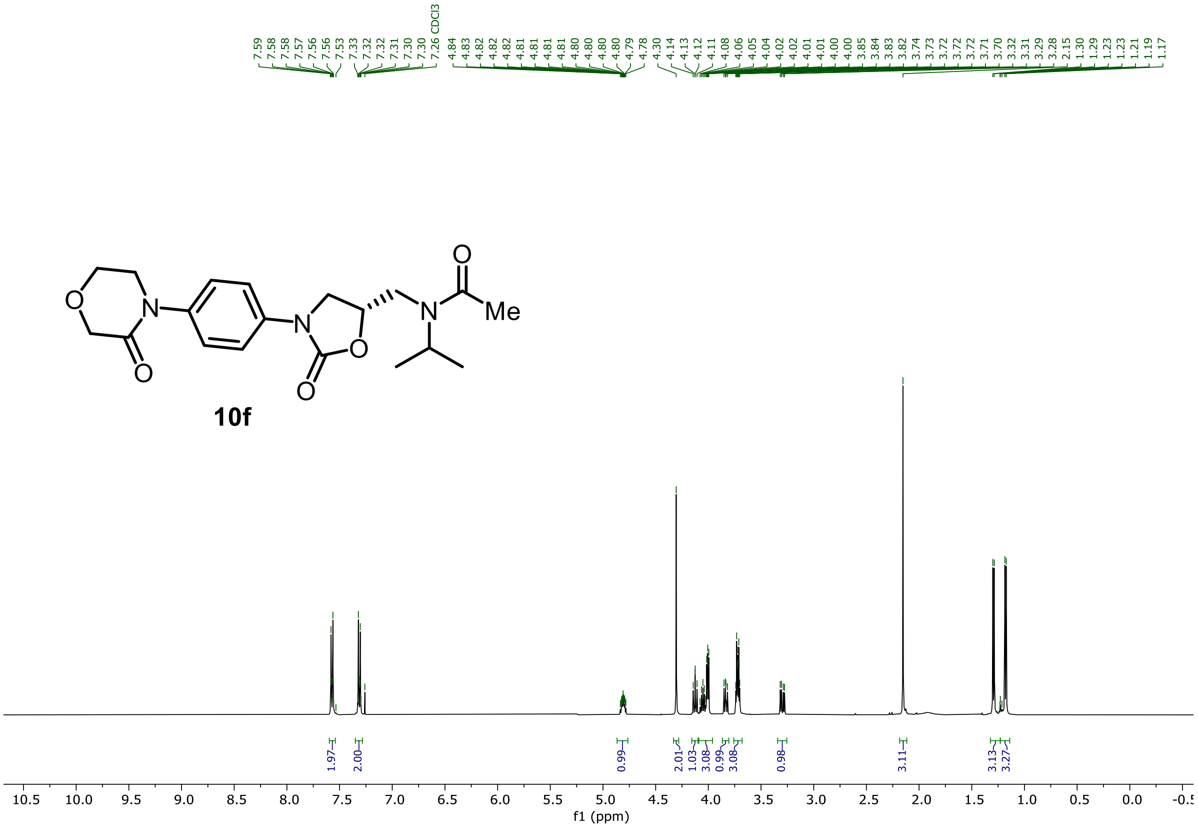

^{1}HNMR spectrum of **10f** run in $CDCl_3$ at 500 MHz.

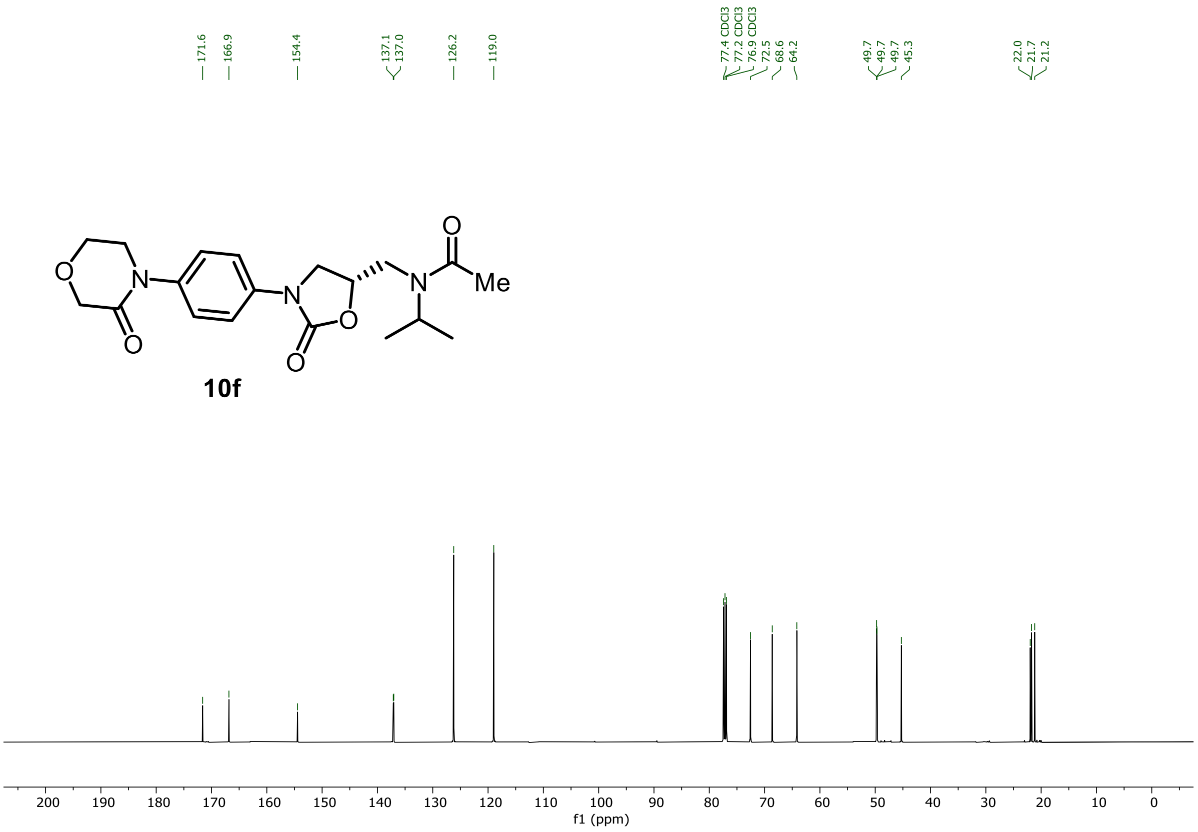

^{13}CNMR spectrum of **10f** run in $CDCl_3$ at 126 MHz.

(*S*)-*N*-Methyl-*N*-((2-oxo-3-(4-(3-oxomorpholino)phenyl)oxazolidin-5-yl)methyl)cyclopropane-carboxamide (10g)

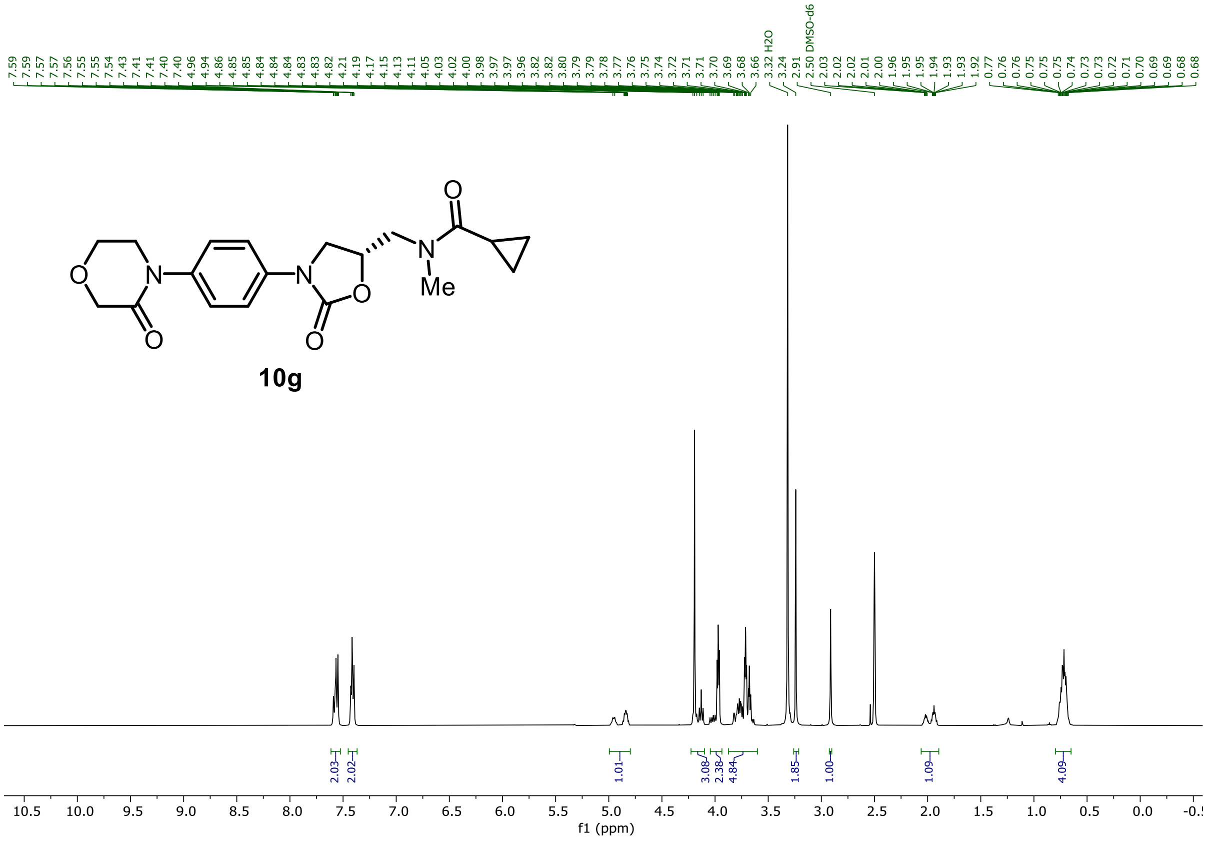

^{1}HNMR spectrum of **10g** run in DMSO-d_6 at 500 MHz.

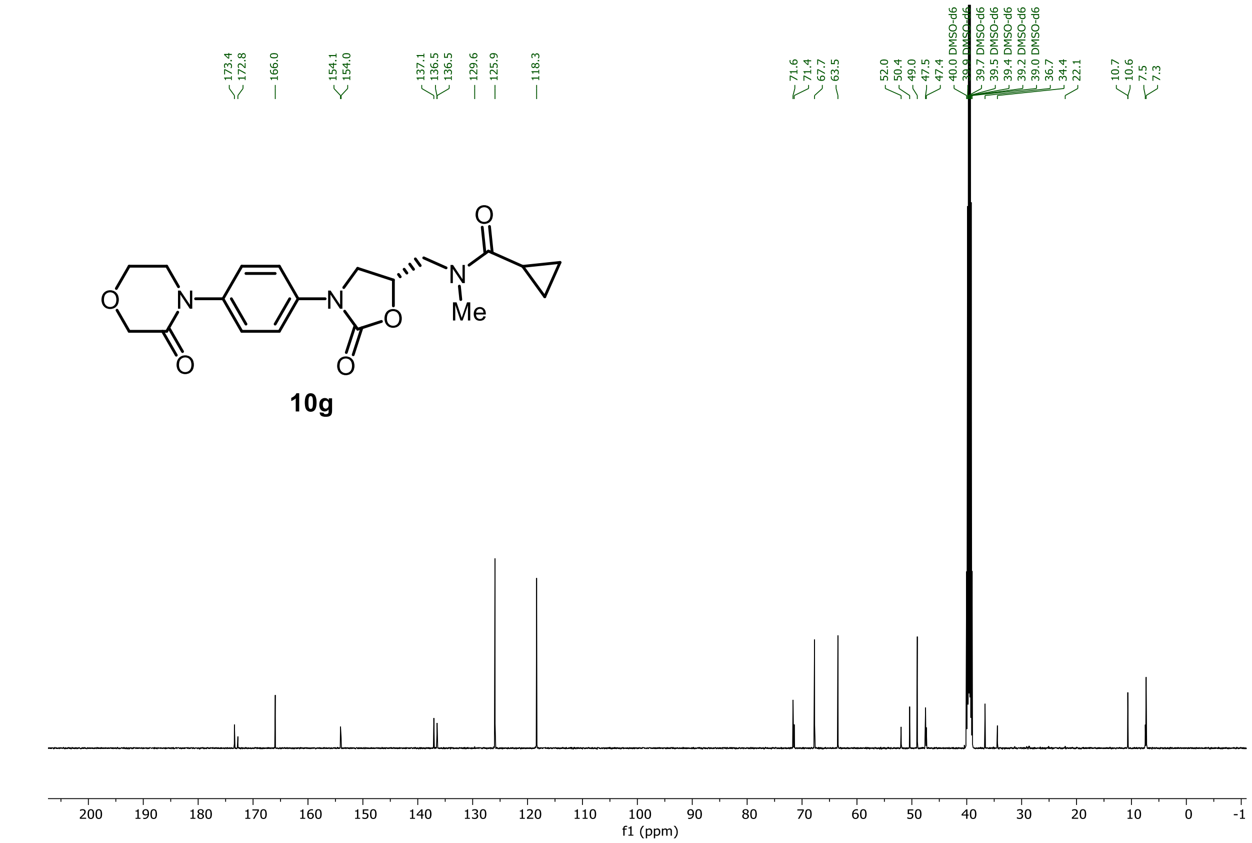

^{13}CNMR spectrum of **10g** run in DMSO-d_6 at 126 MHz.

(S)-*N*-Isopropyl-*N*-((2-oxo-3-(4-(3-oxomorpholino)phenyl)oxazolidin-5-yl)methyl)cyclopropanecarboxamide (10h)

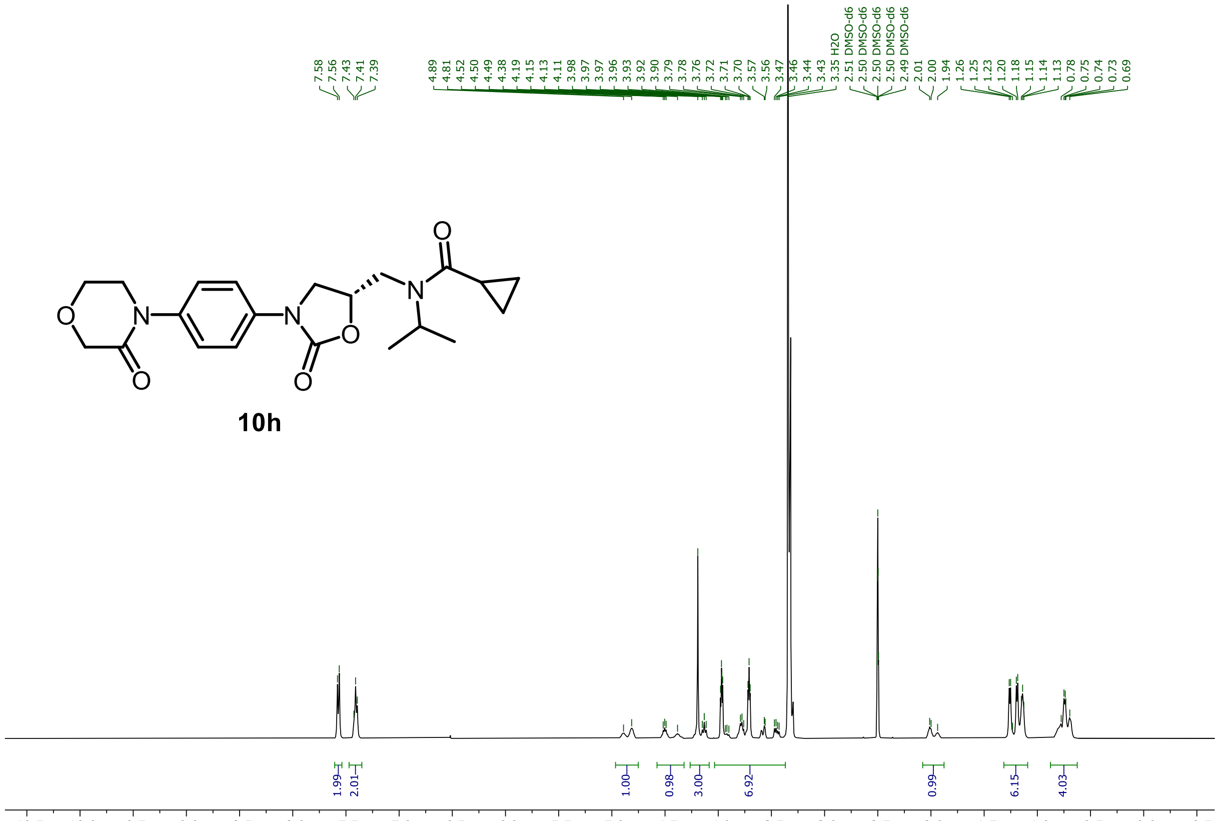

^{1}HNMR spectrum of **10h** run in DMSO-d_6 at 500 MHz.

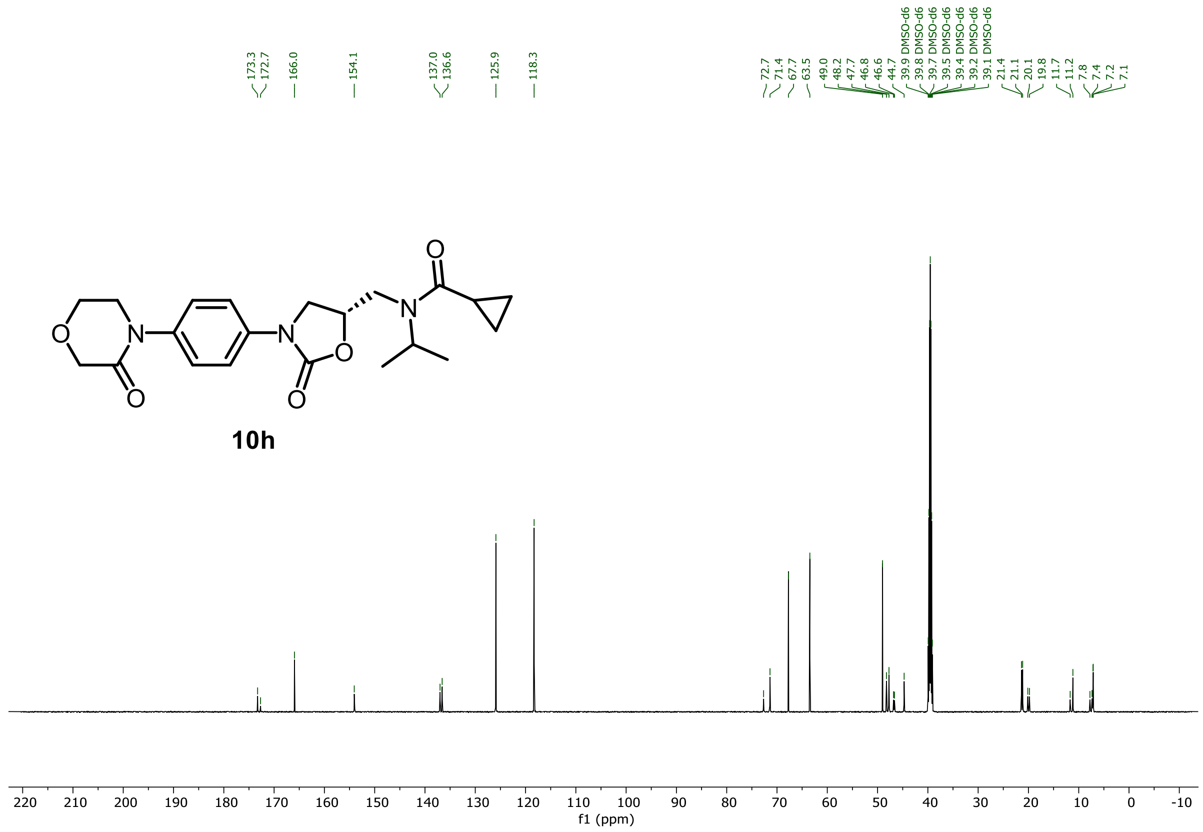

^{13}CNMR spectrum of **10h** run in DMSO-d_6 at 151 MHz.

2-(2-Methyl-5-nitro-1*H*-imidazol-1-yl)ethyl dimethylcarbamate (12e)

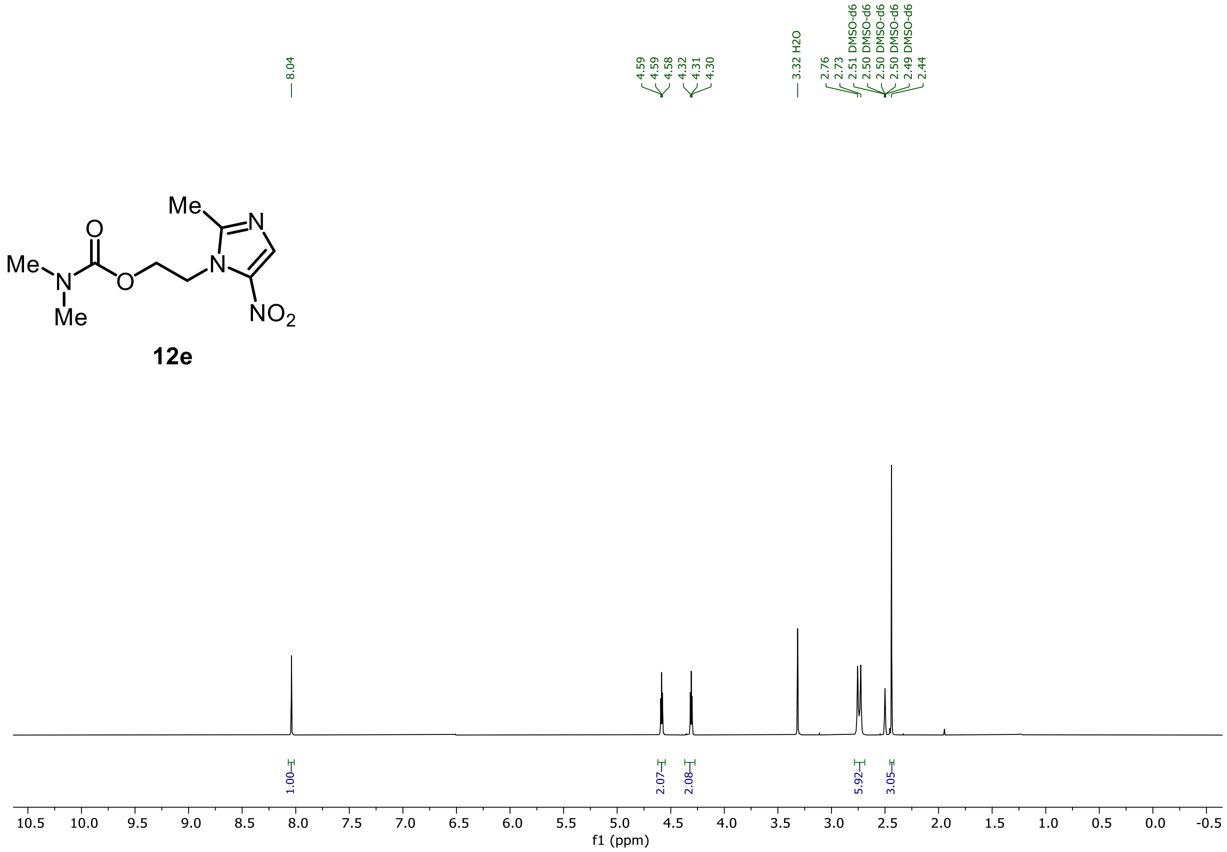

^{1}HNMR spectrum of **12e** run in DMSO-d_6 at 600 MHz.

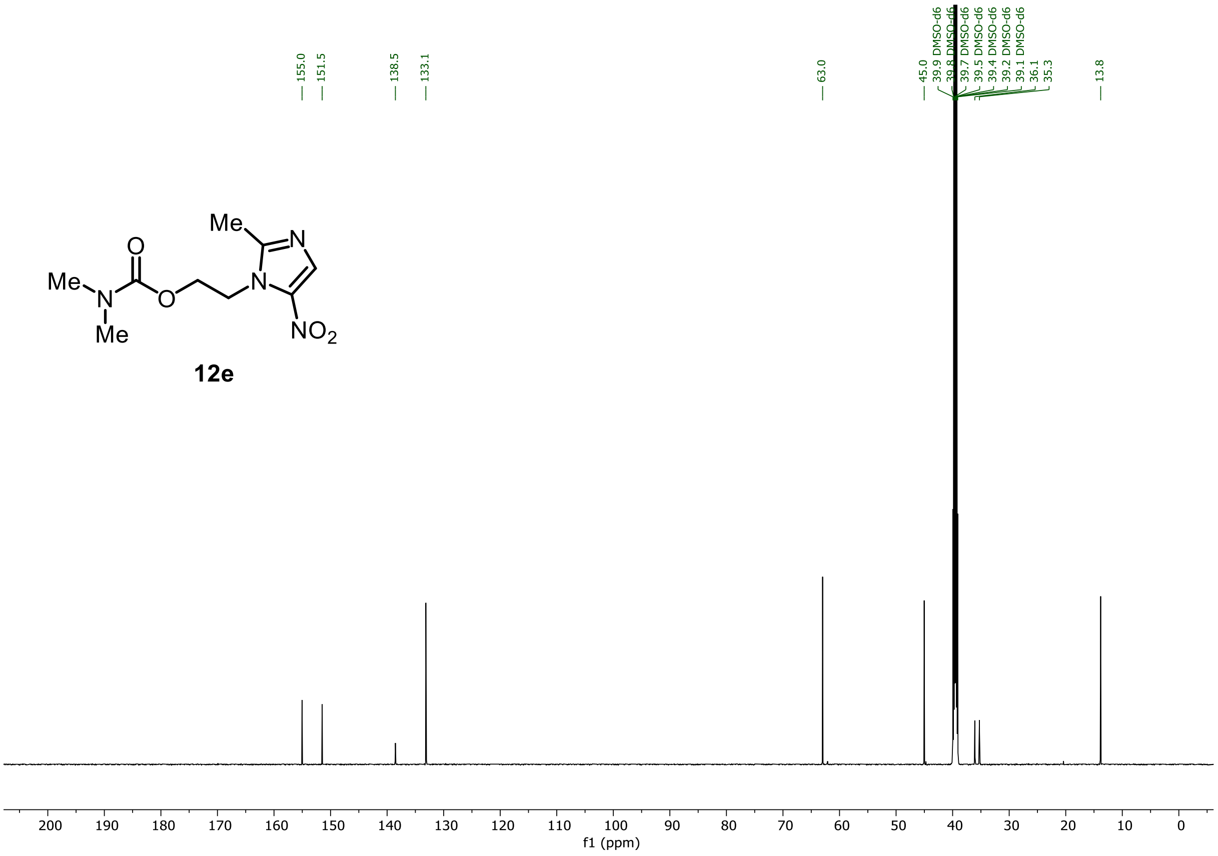

^{13}CNMR spectrum of **12e** run in DMSO-d_6 at 151 MHz.

2-(2-Methyl-5-nitro-1*H*-imidazol-1-yl)ethyl isopropyl(methyl)carbamate (12f)

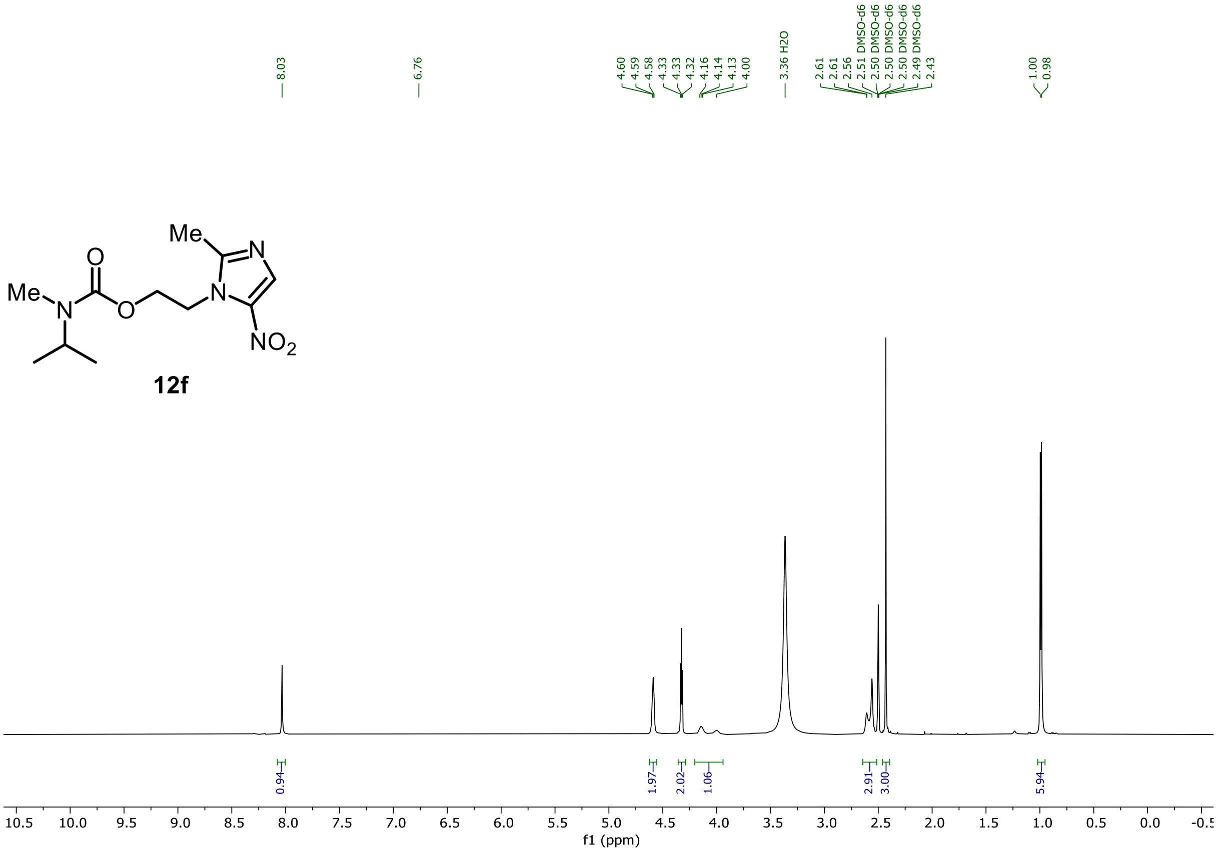

^{1}HNMR spectrum of **12f** run in DMSO-d_6 at 600 MHz.

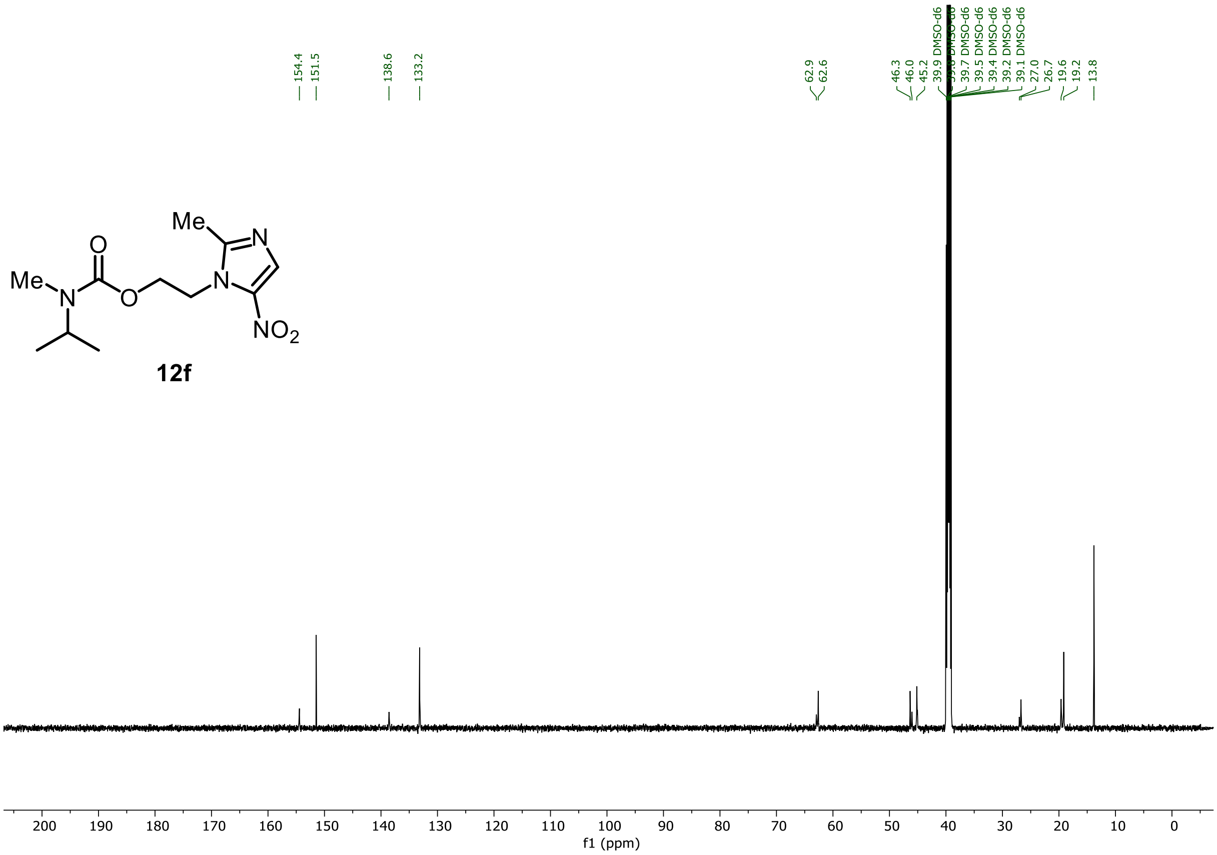

12f

^{13}CNMR spectrum of **12f** run in DMSO-d_6 at 151 MHz.

2-(2-Methyl-5-nitro-1*H*-imidazol-1-yl)ethyl cyclopropyl(methyl)carbamate (12g)

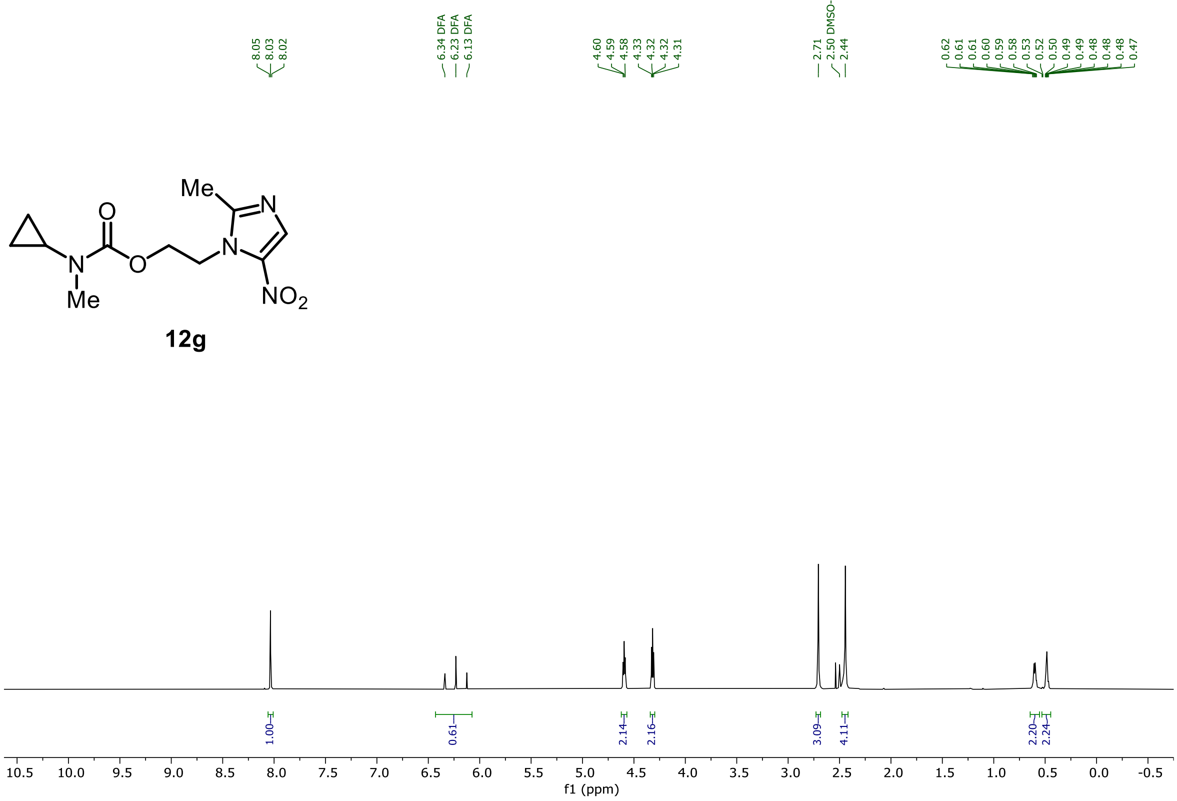

12g

^{1}HNMR spectrum of **12g** run in DMSO-d_6 at 600 MHz.

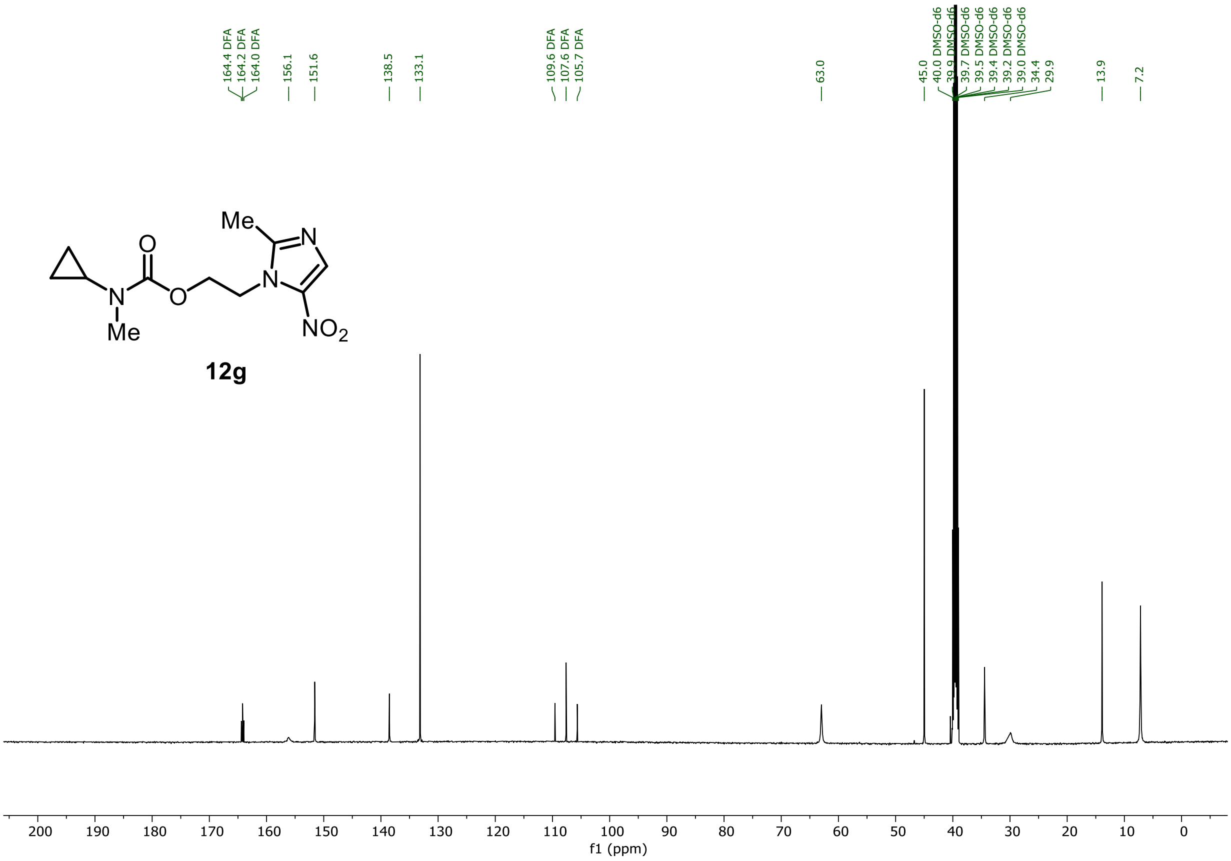

^{13}CNMR spectrum of **12g** run in DMSO-d_6 at 151 MHz.

2-(2-Methyl-5-nitro-1*H*-imidazol-1-yl)ethyl cyclopropyl(isopropyl)carbamate (12h)

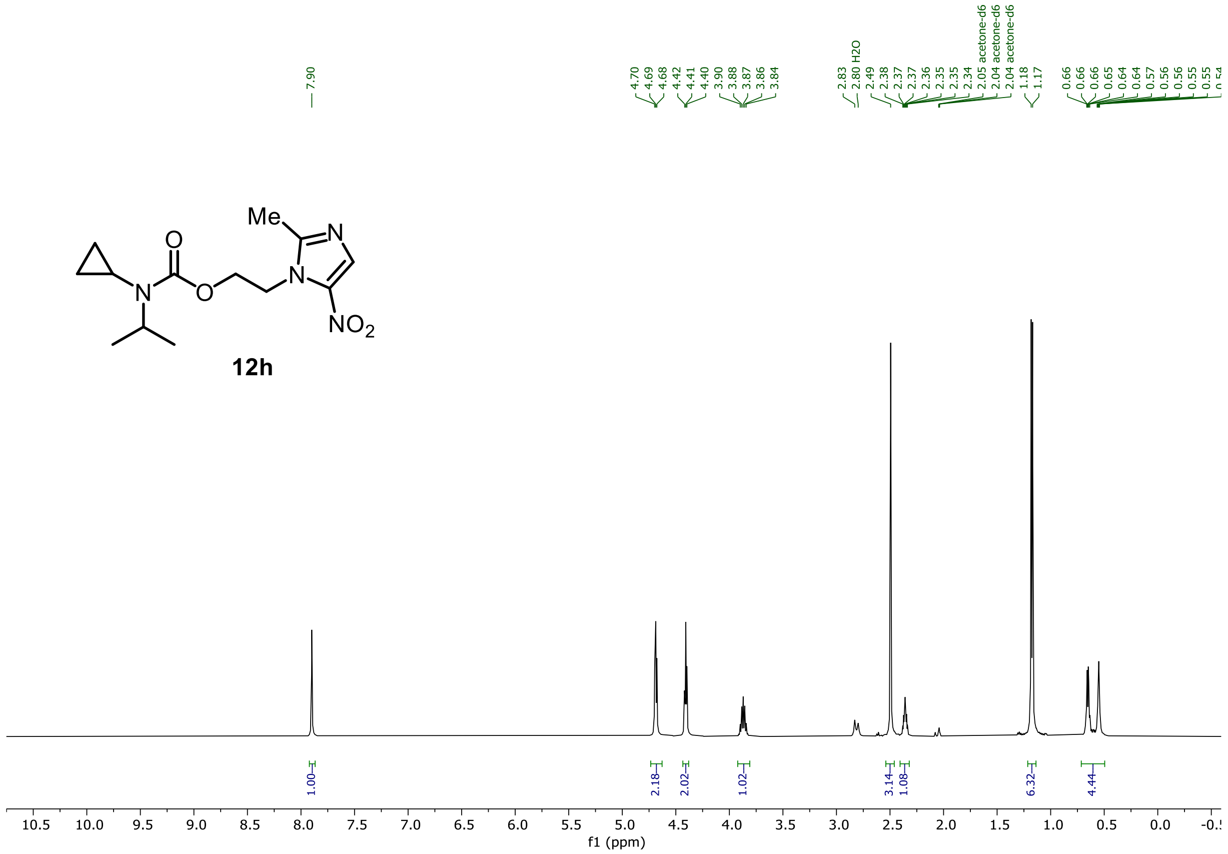

^{1}HNMR spectrum of **12h** run in acetone-d_6 at 500 MHz.

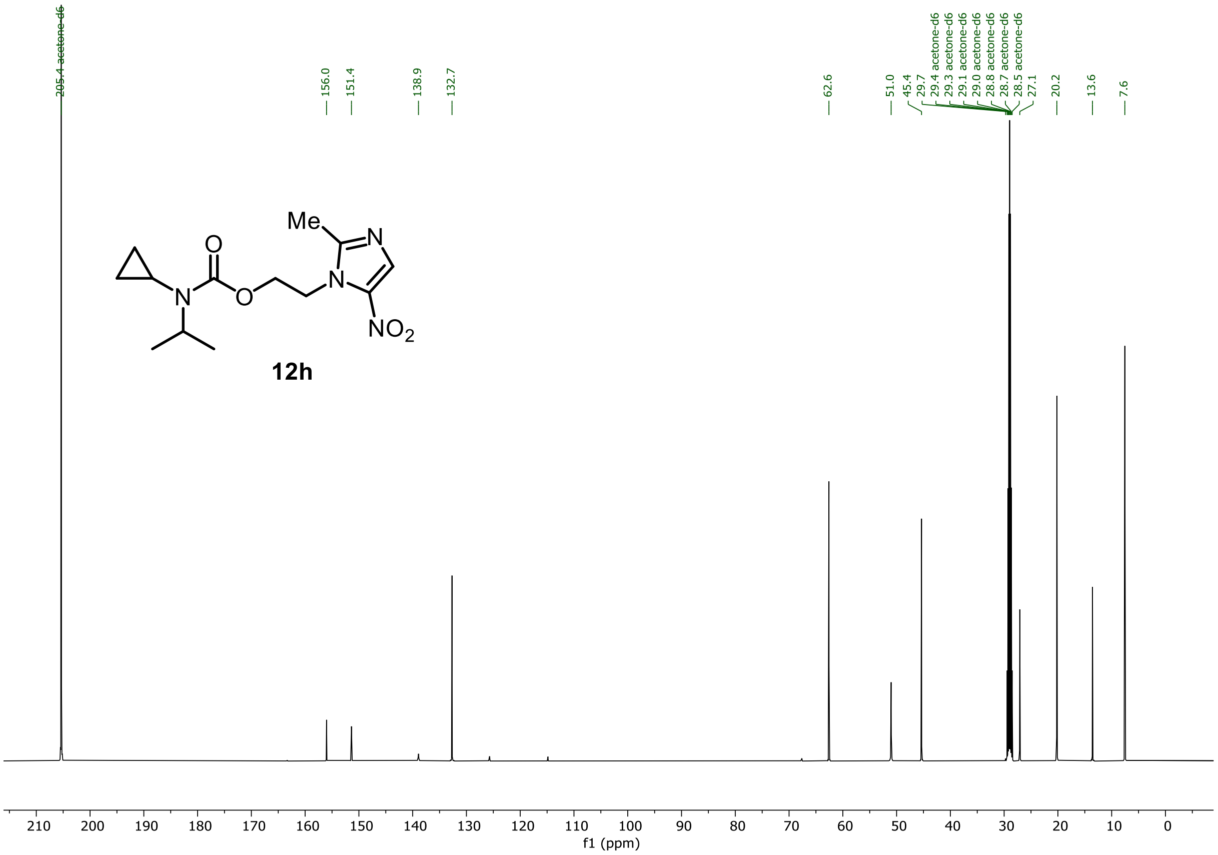

^{13}CNMR spectrum of **12h** run in acetone-d_6 at 126 MHz.

1-Cyclopropyl-7-(4-(dimethylcarbamoyl)piperazin-1-yl)-6-fluoro-4-oxo-1,4-dihydroquinoline-3-carboxylic acid (15e)

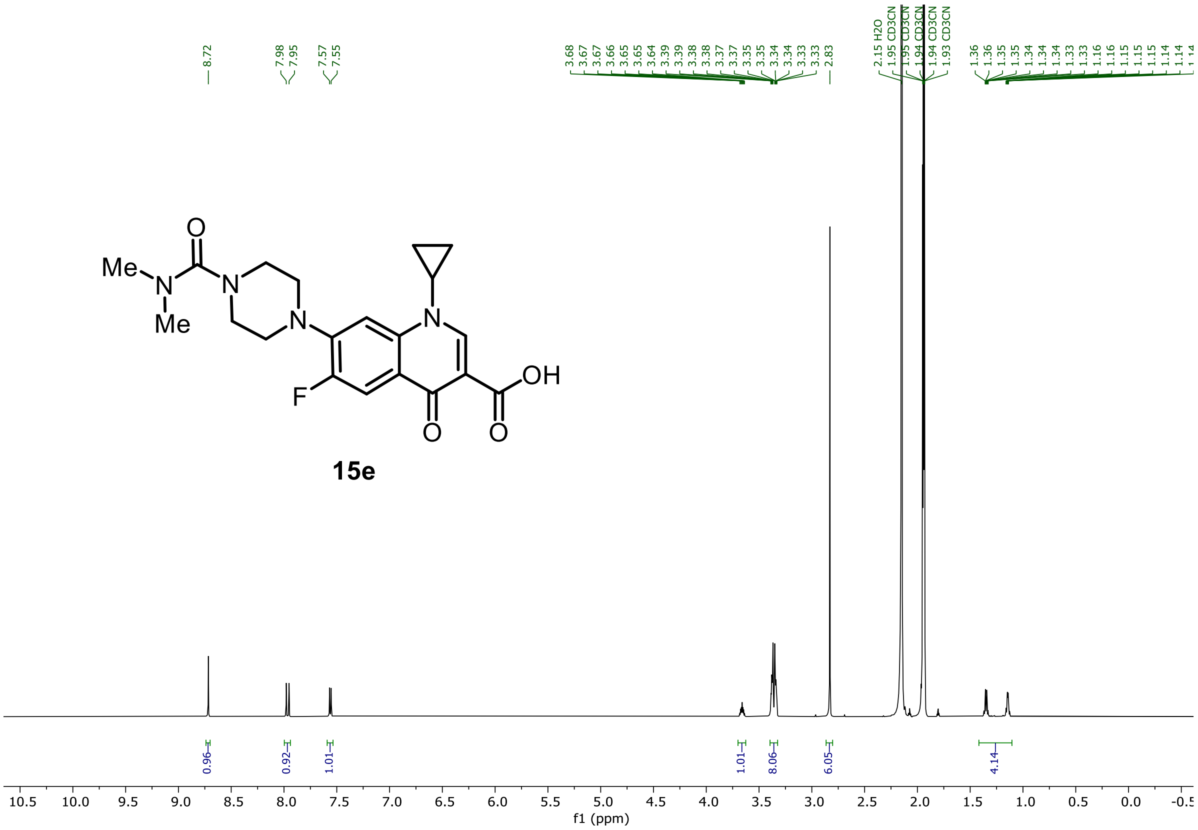

^{1}HNMR spectrum of **15e** run in CD_3CN at 500 MHz.

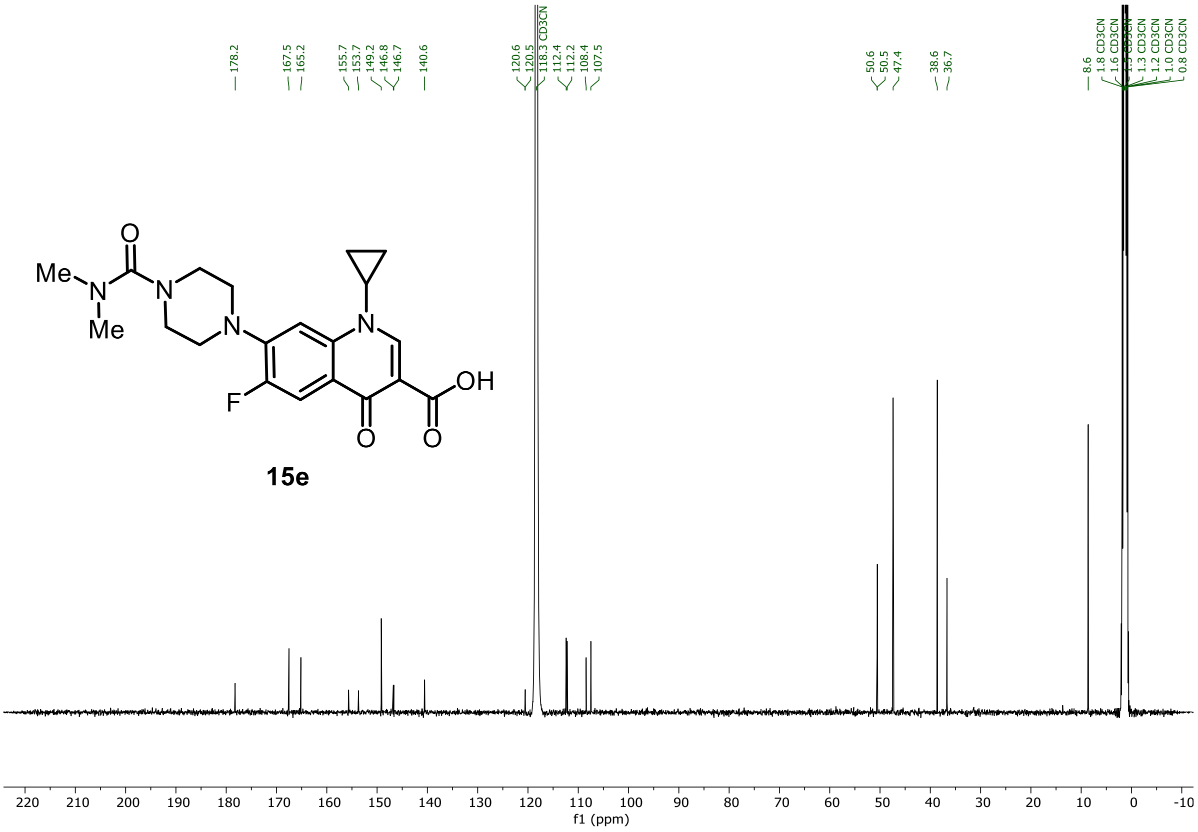

^{13}CNMR spectrum of **15e** run in CD_3CN at 126 MHz.

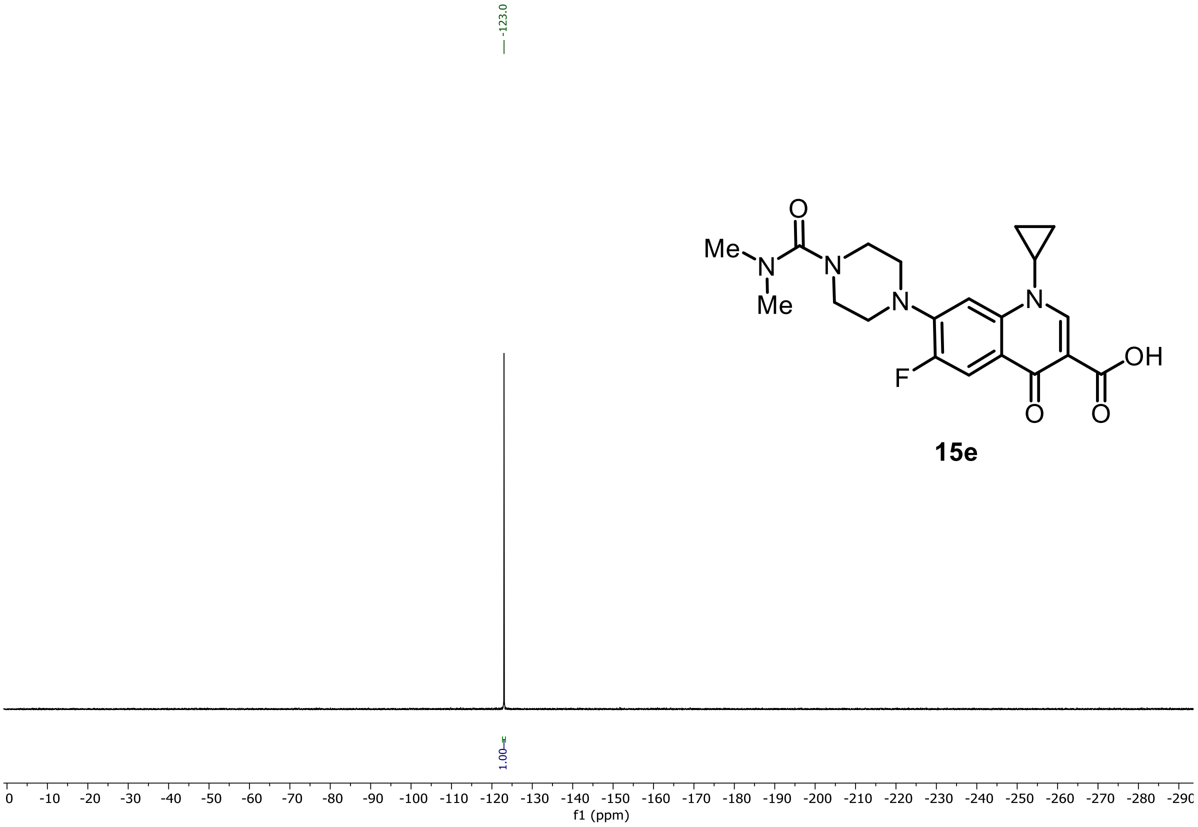

^{19}FNMR spectrum of **15e** run in CD_3CN at 470 MHz.

1-Cyclopropyl-6-fluoro-7-(4-(isopropyl(methyl)carbamoyl)piperazin-1-yl)-4-oxo-1,4-dihydro-quinoline-3-carboxylic acid (15f)

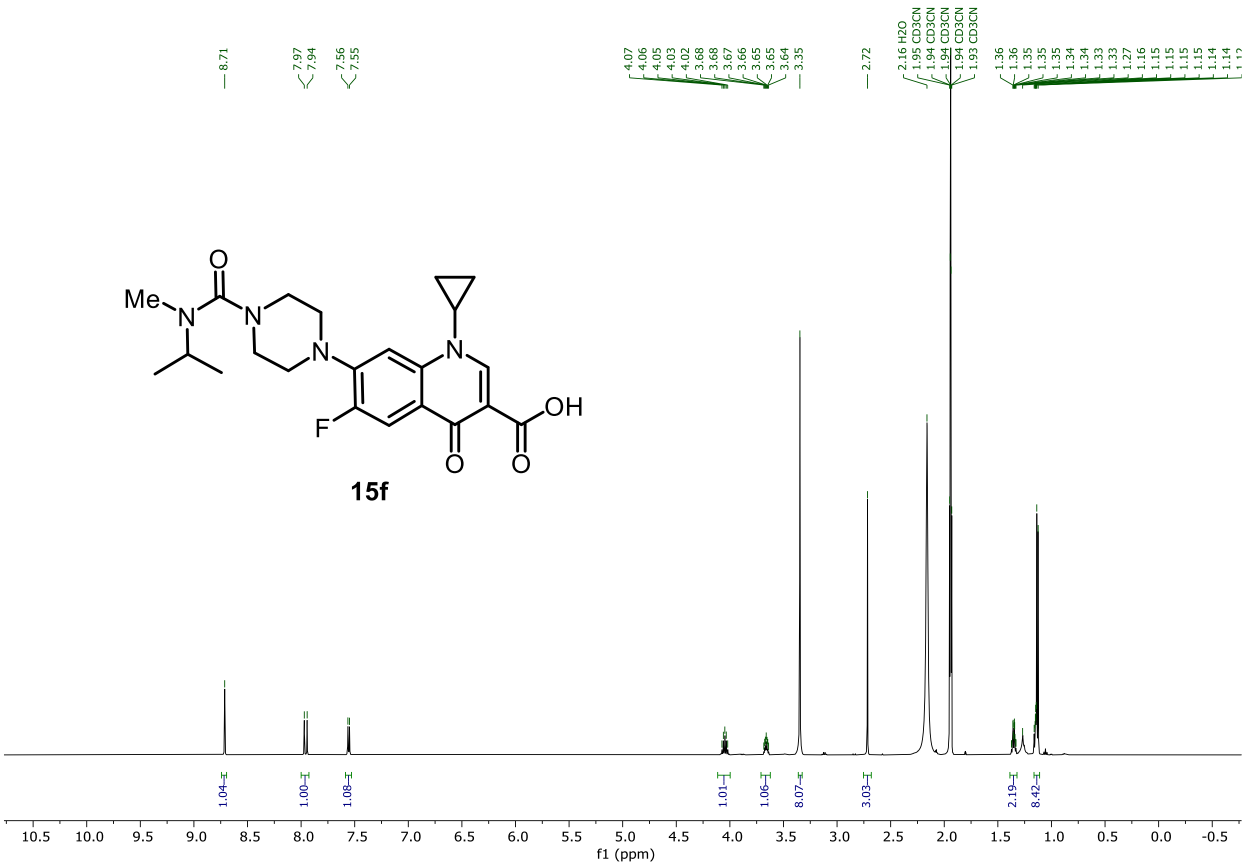

^{1}HNMR spectrum of **15f** run in CD_3CN at 500 MHz.

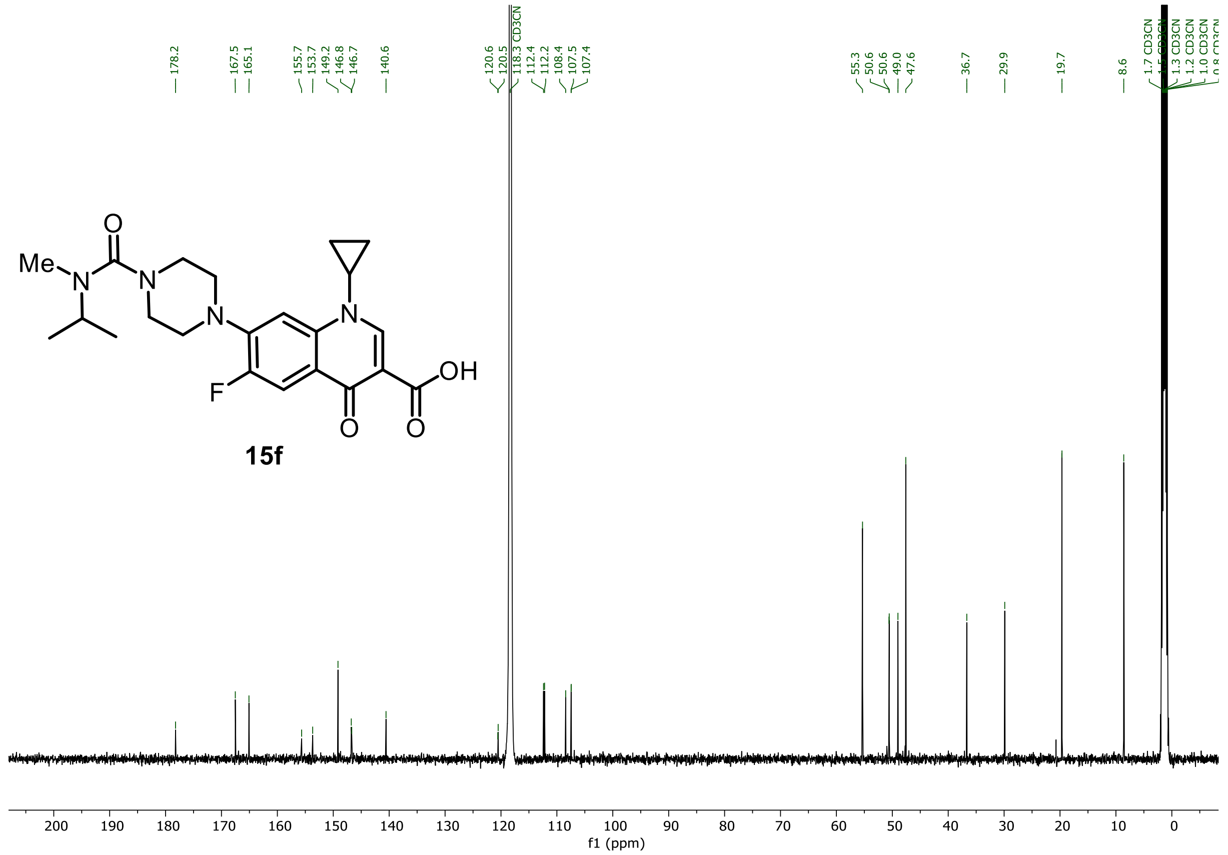

^{13}CNMR spectrum of **15f** run in CD_3CN at 126 MHz.

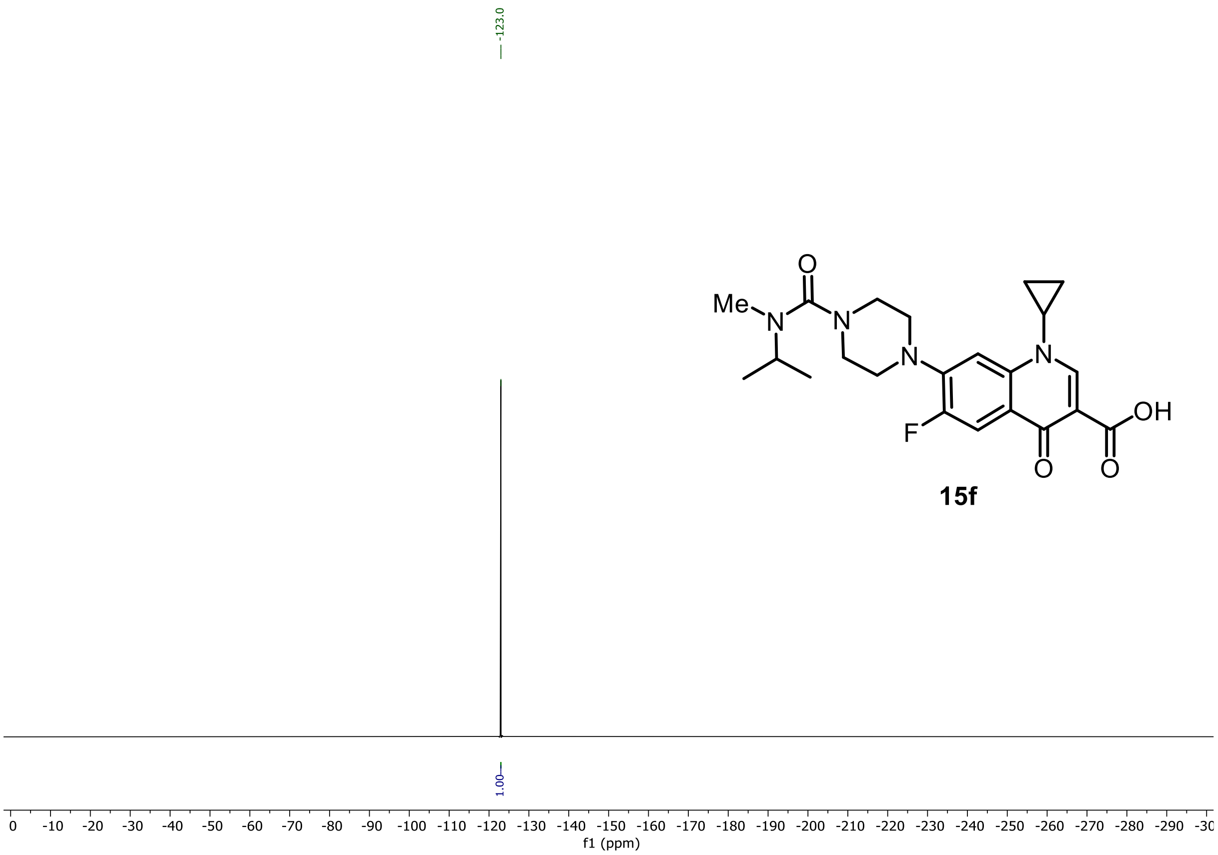

^{19}FNMR spectrum of **15f** run in CD_3CN at 470 MHz.

1-Cyclopropyl-7-(4-(cyclopropyl(methyl)carbamoyl)piperazin-1-yl)-6-fluoro-4-oxo-1,4-dihydroquinoline-3-carboxylic acid (15g)

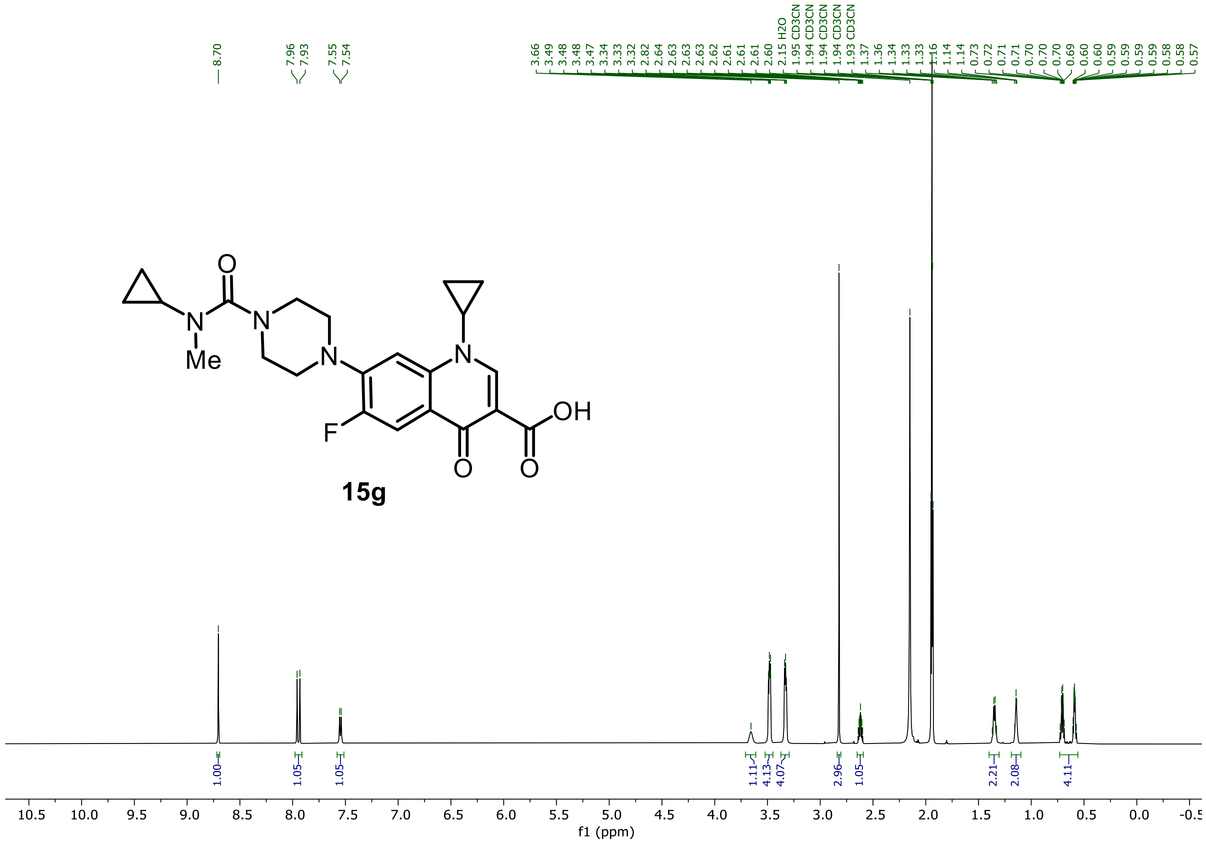

^{1}HNMR spectrum of **15g** run in CD_3CN at 500 MHz.

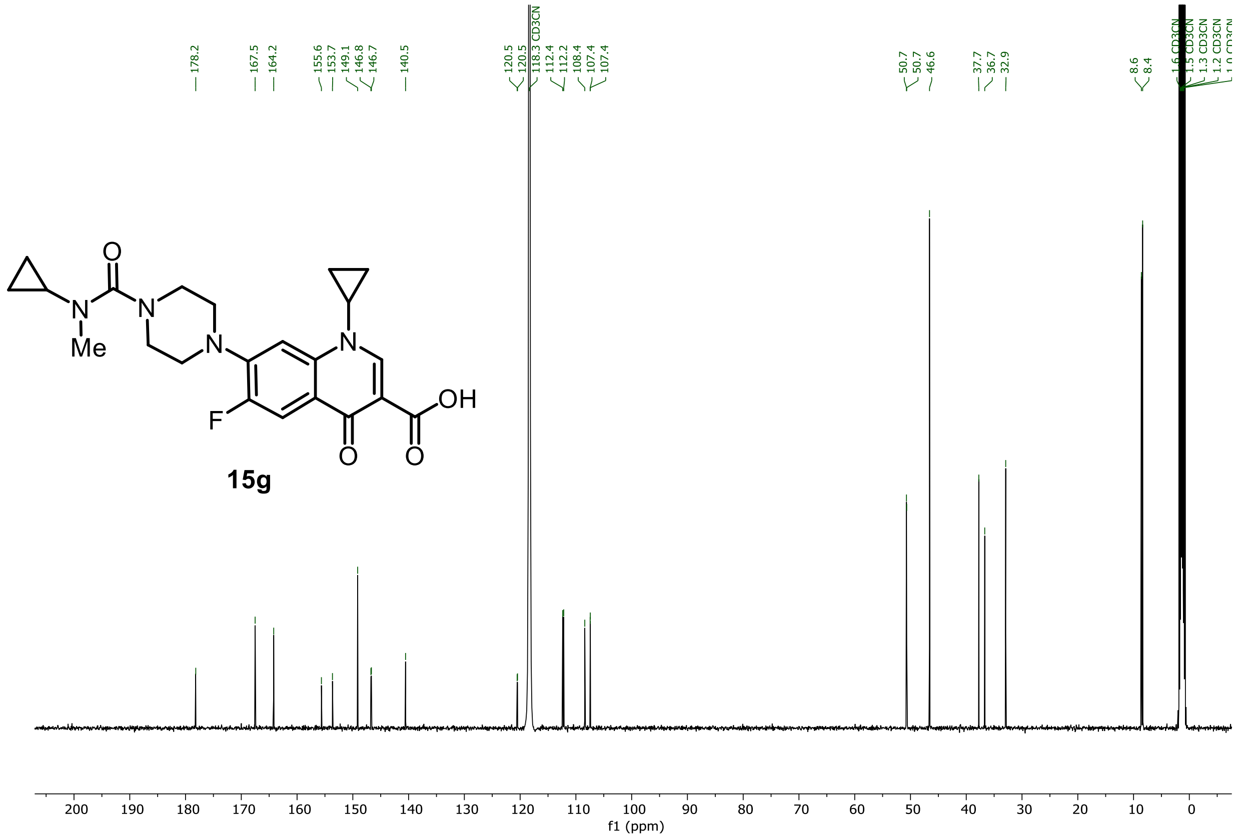

^{13}CNMR spectrum of **15g** run in CD_3CN at 126 MHz.

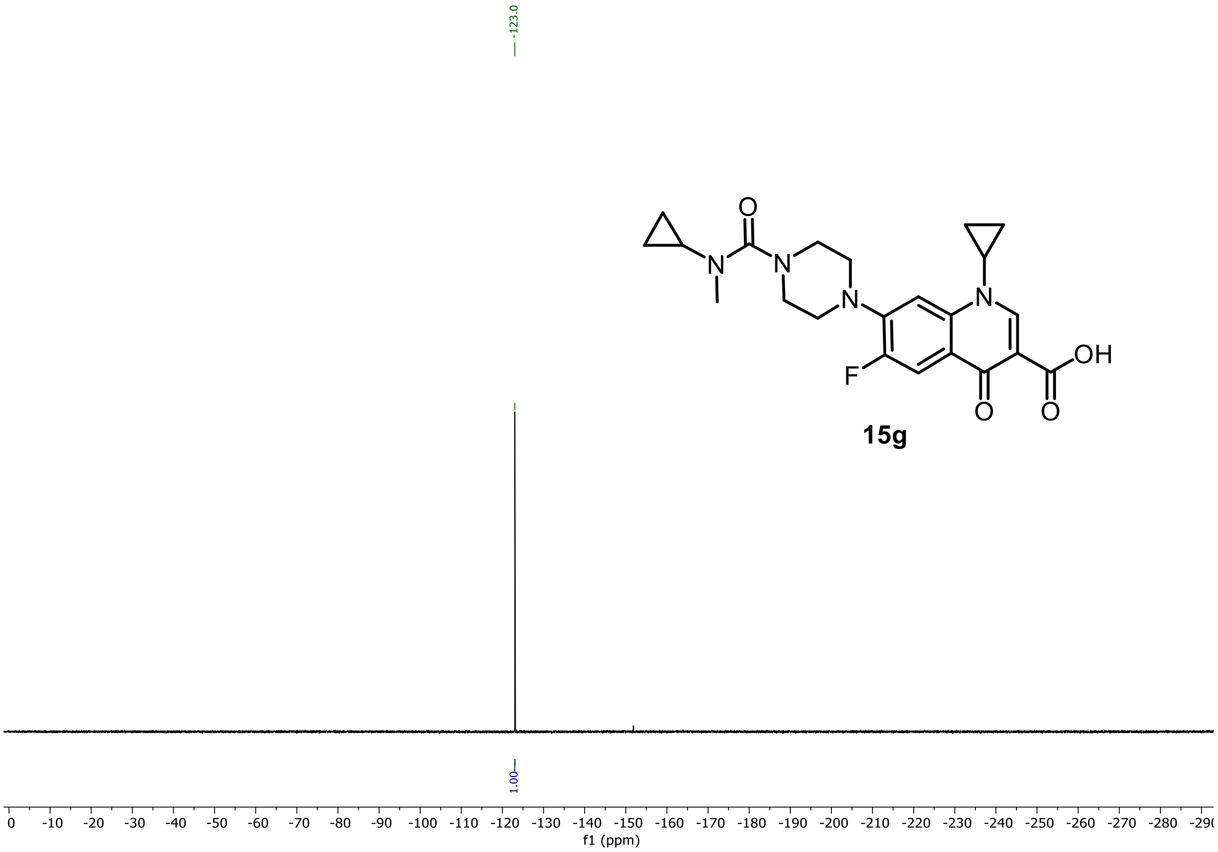

^{19}FNMR spectrum of **15g** run in CD_3CN at 470 MHz.

1-Cyclopropyl-7-(4-(cyclopropyl(isopropyl)carbamoyl)piperazin-1-yl)-6-fluoro-4-oxo-1,4-dihydroquinoline-3-carboxylic acid (15h)

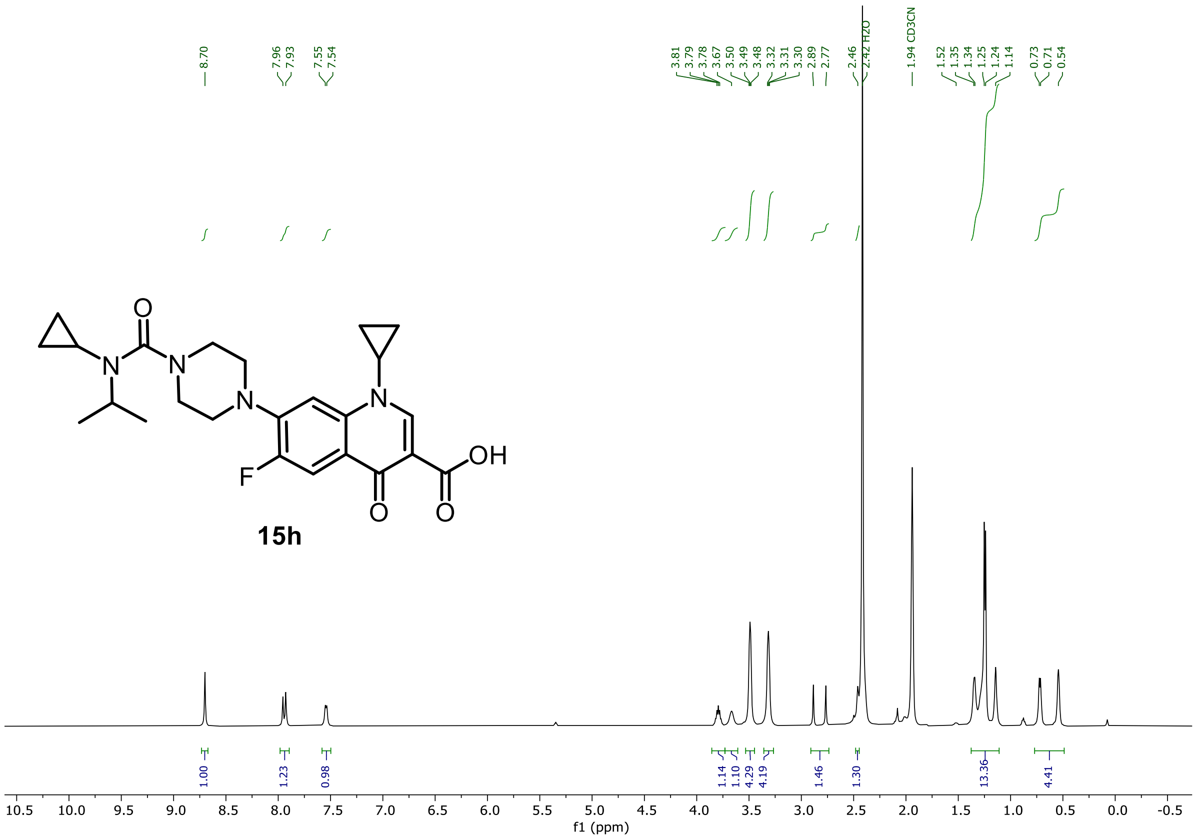

^{1}HNMR spectrum of **15h** run in CD_3CN at 500 MHz.

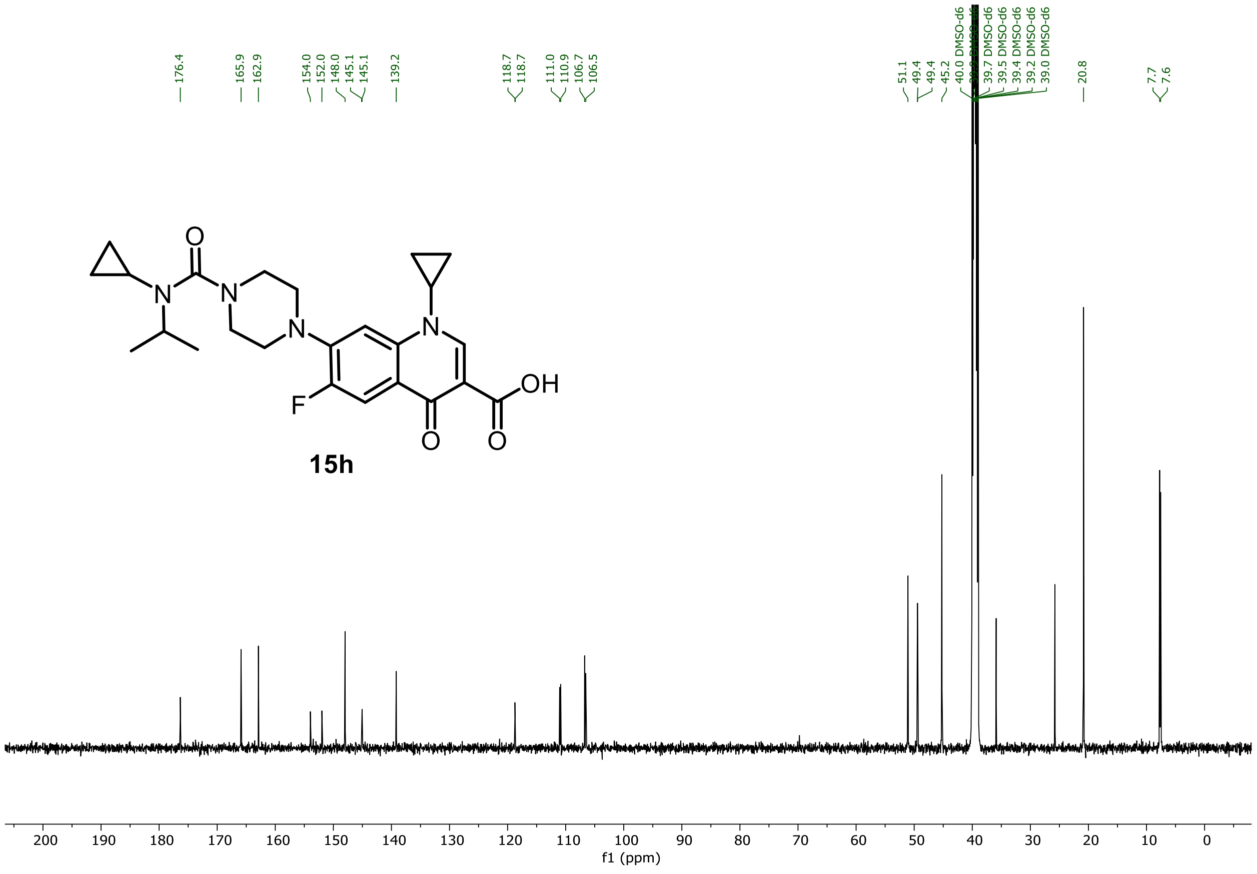

^{13}CNMR spectrum of **15h** run in DMSO-d_6 at 126 MHz.

N-Methyl-4-(morpholinomethyl)-*N*-(3,4,5-trimethoxyphenyl)benzamide (3b)

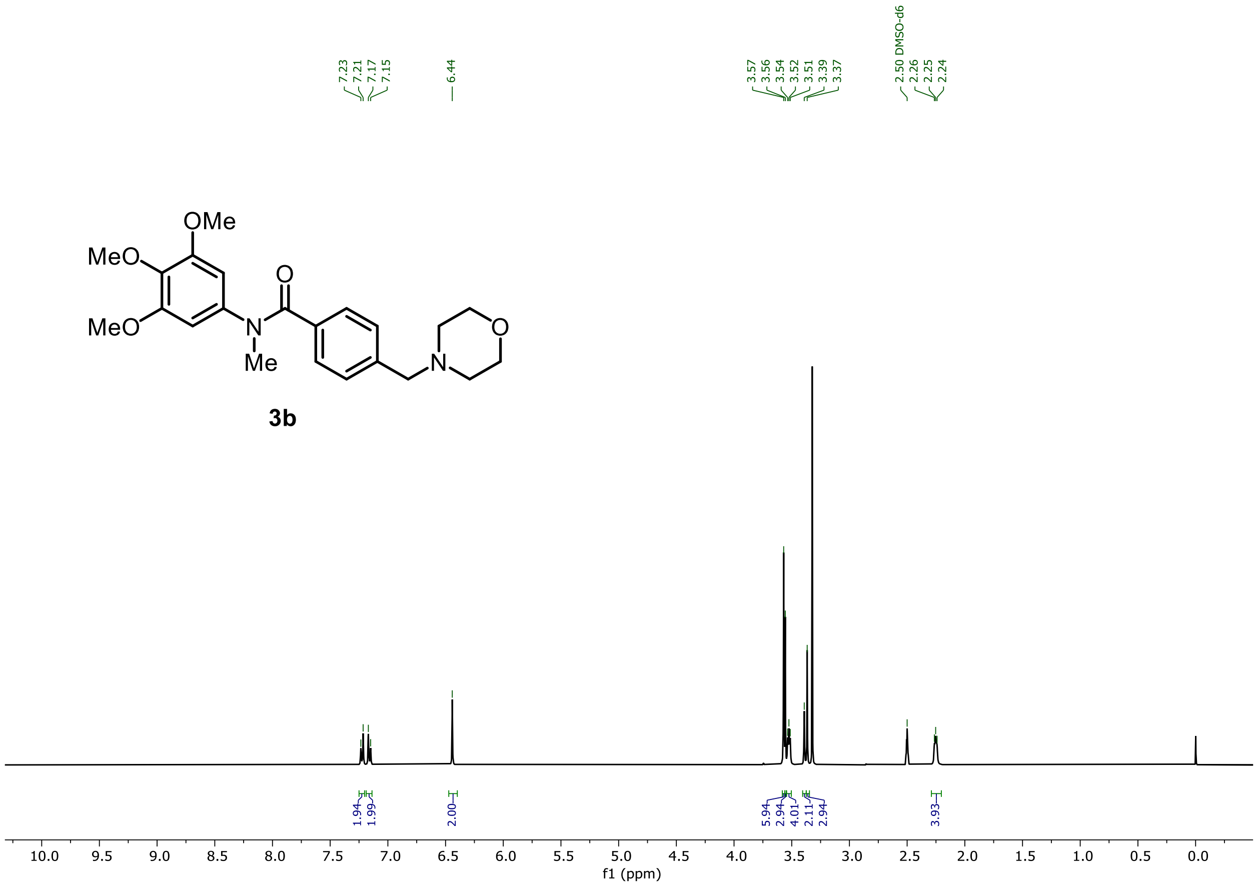

^{1}HNMR spectrum of **3b** run in DMSO-d_6 at 400 MHz.

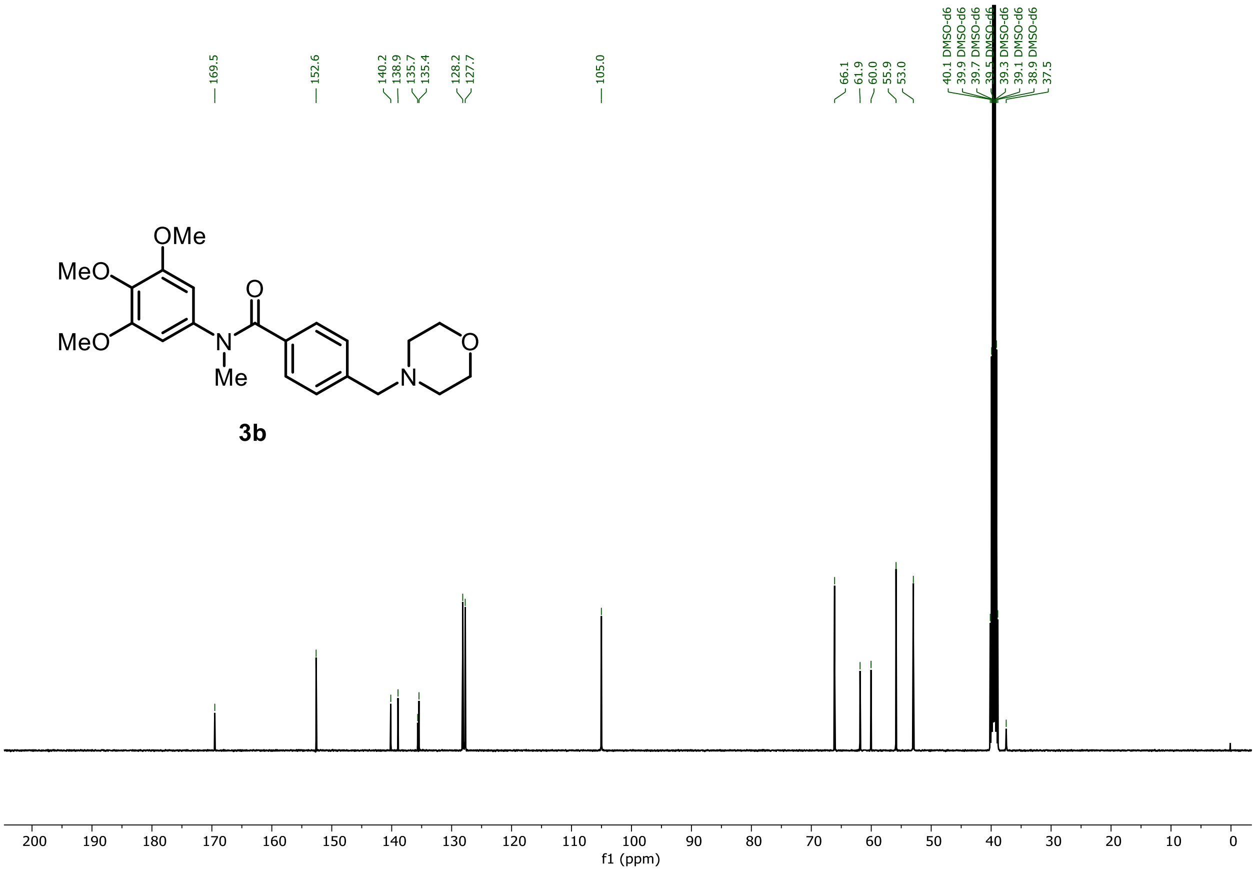

^{13}CNMR spectrum of **3b** run in DMSO-d_6 at 101 MHz.

N-Methyl-*N*-(3,4,5-trimethoxyphenyl)cyclopropanecarboxamide (4b)

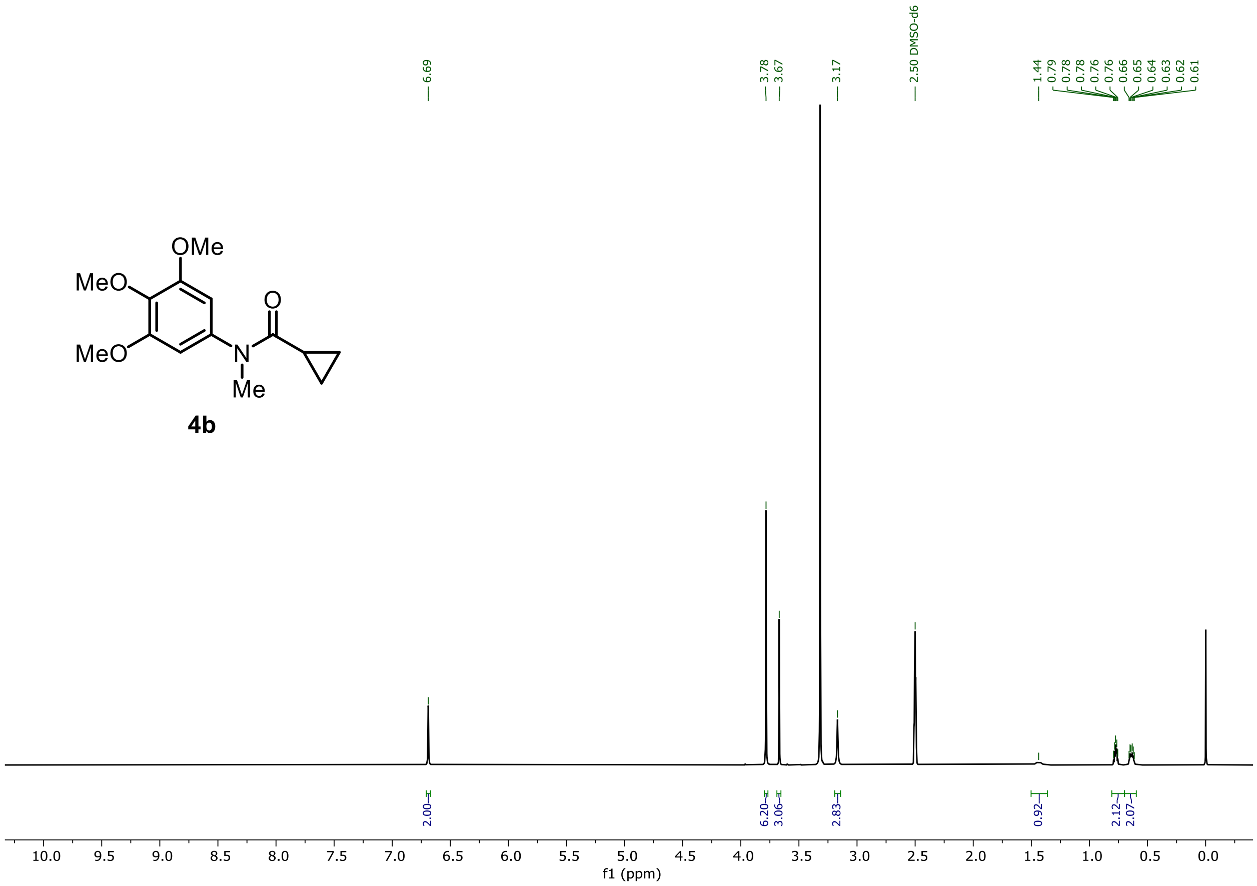

^{1}HNMR spectrum of **4b** run in DMSO-d_6 at 400 MHz.

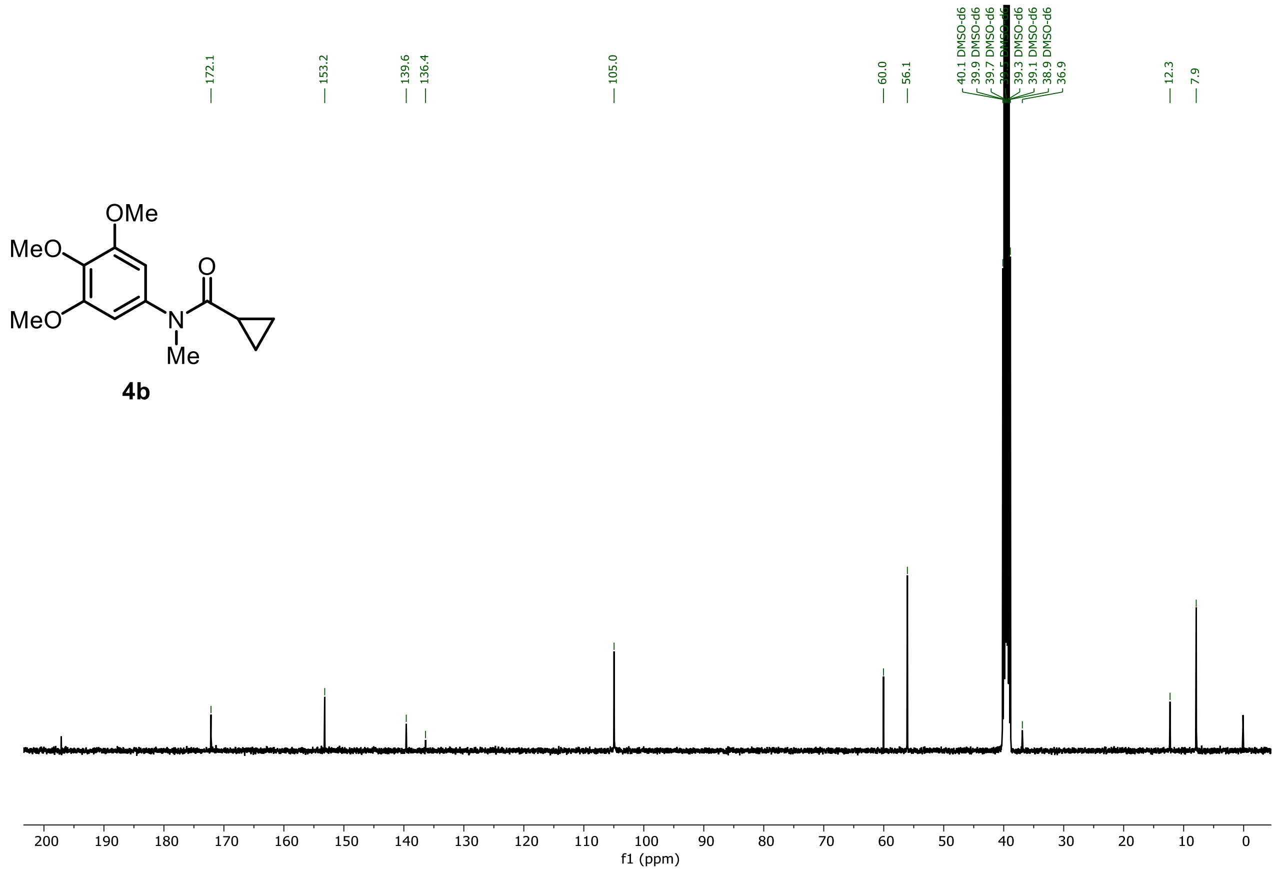

^{13}CNMR spectrum of **4b** run in DMSO-d_6 at 101 MHz.

3-Hydroxy-*N*-(4-methoxybenzyl)-*N*-methylazetidine-1-carboxamide (5b)

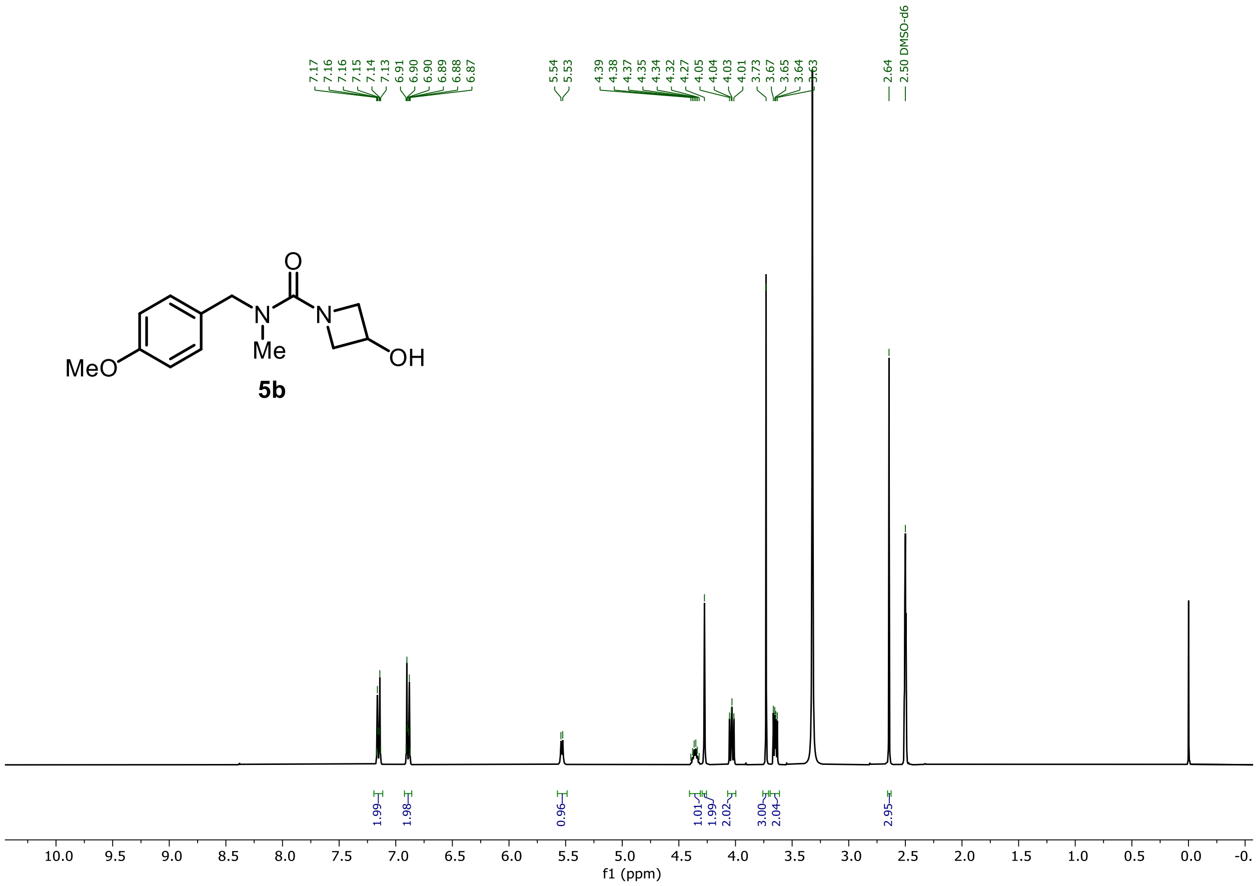

^{1}HNMR spectrum of **5b** run in DMSO-d_6 at 400 MHz.

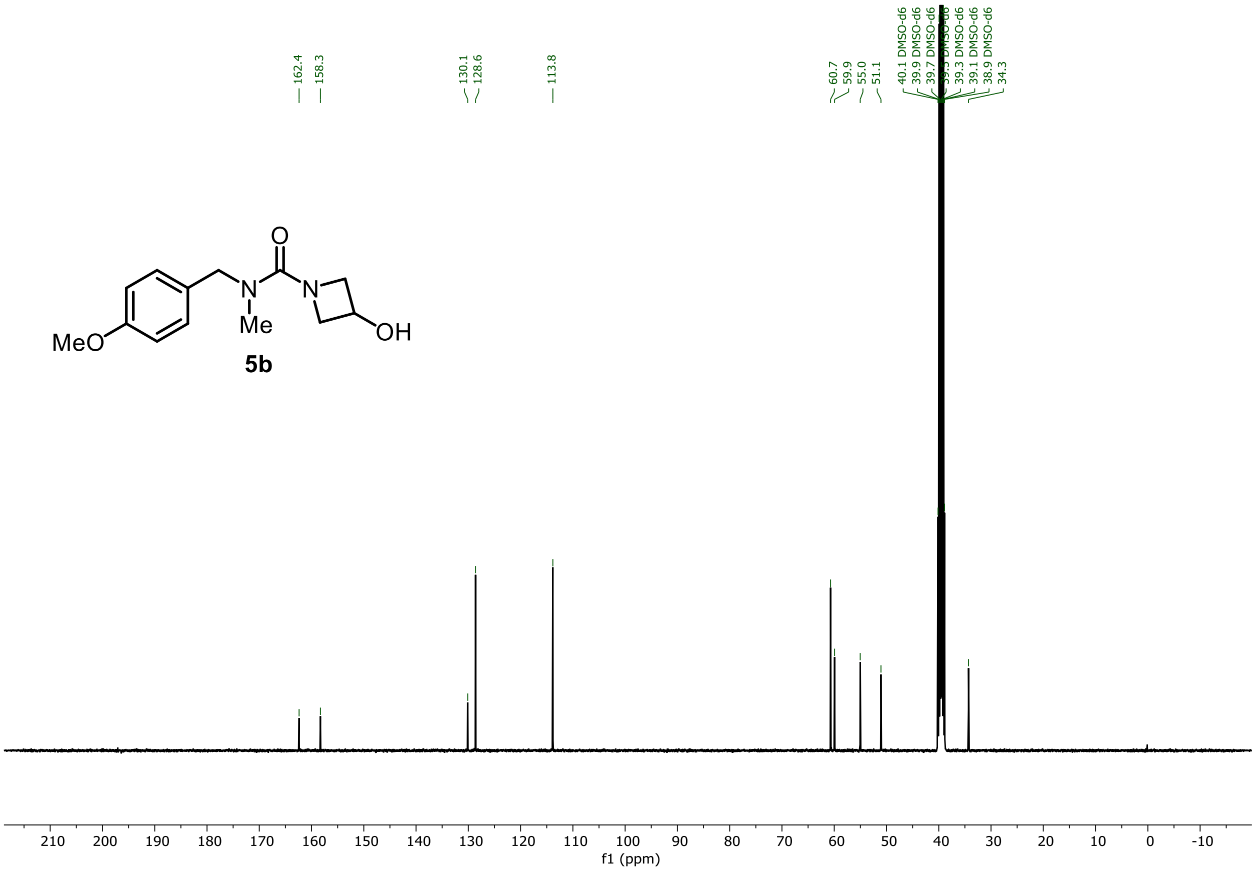

^{13}CNMR spectrum of **5b** run in DMSO-d_6 at 101 MHz.

3-Hydroxy-*N*-(4-methoxybenzyl)-*N*-methylpiperidine-1-carboxamide (6b)

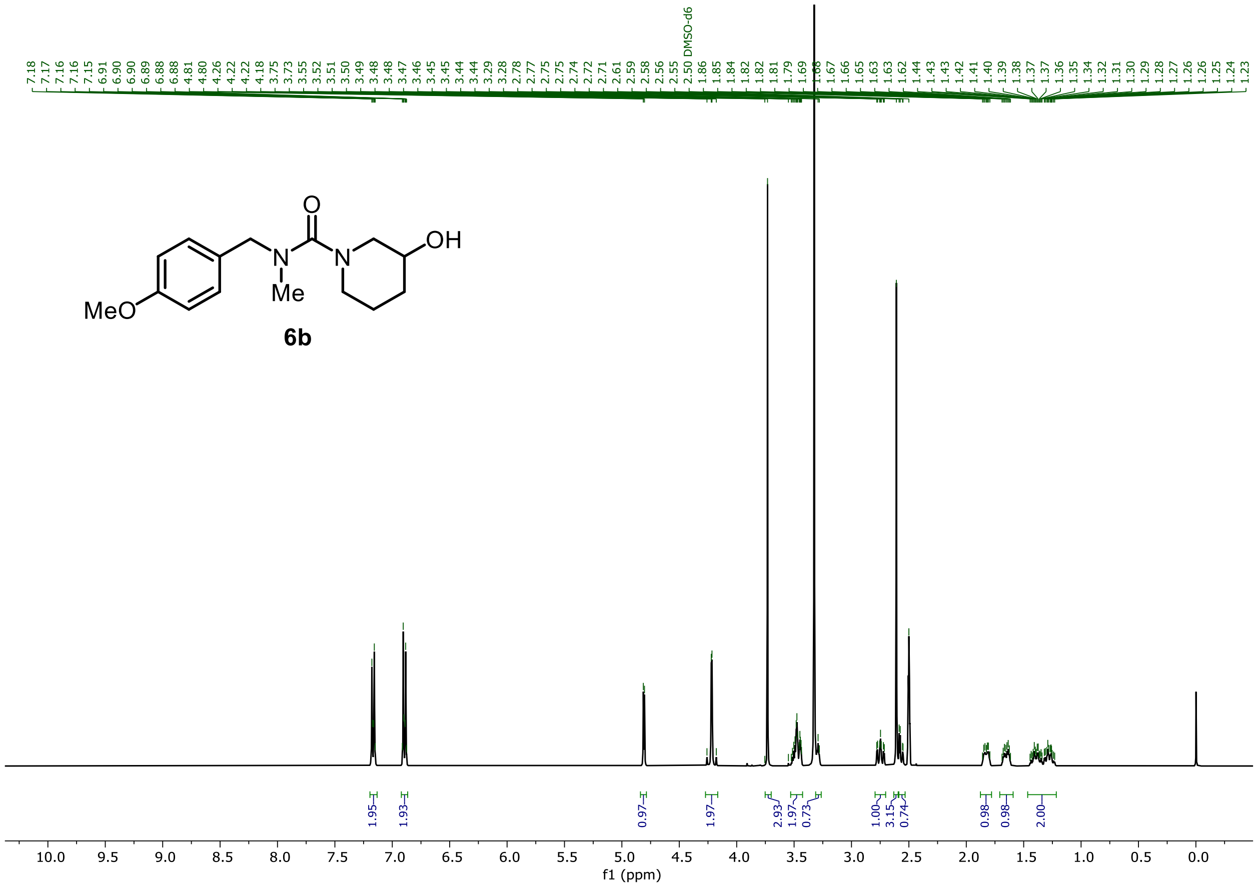

^{1}HNMR spectrum of **6b** run in DMSO-d_6 at 400 MHz.

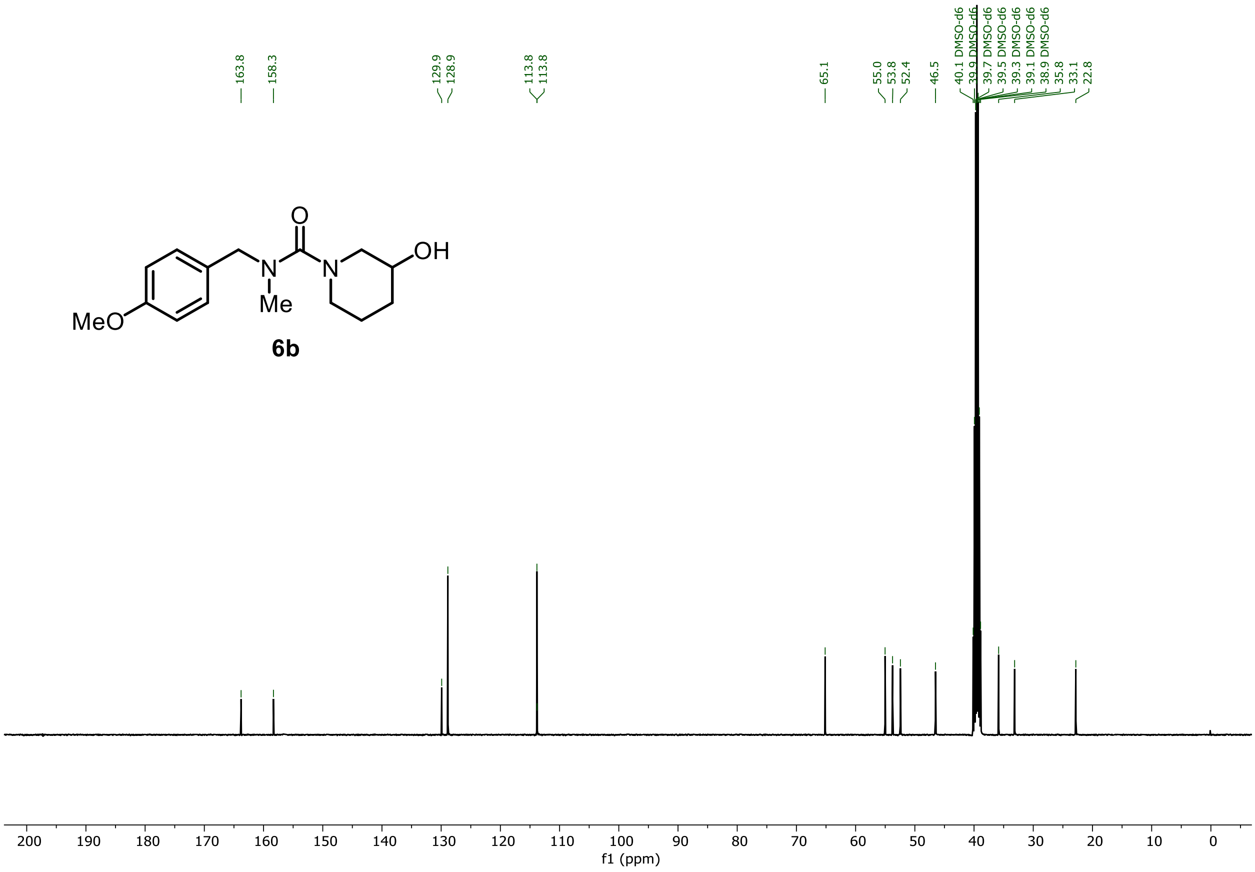

^{13}CNMR spectrum of **6b** run in DMSO-d_6 at 101 MHz.

N-(4-Methoxybenzyl)-*N*,4-dimethyl-3-oxopiperazine-1-carboxamide (7b)

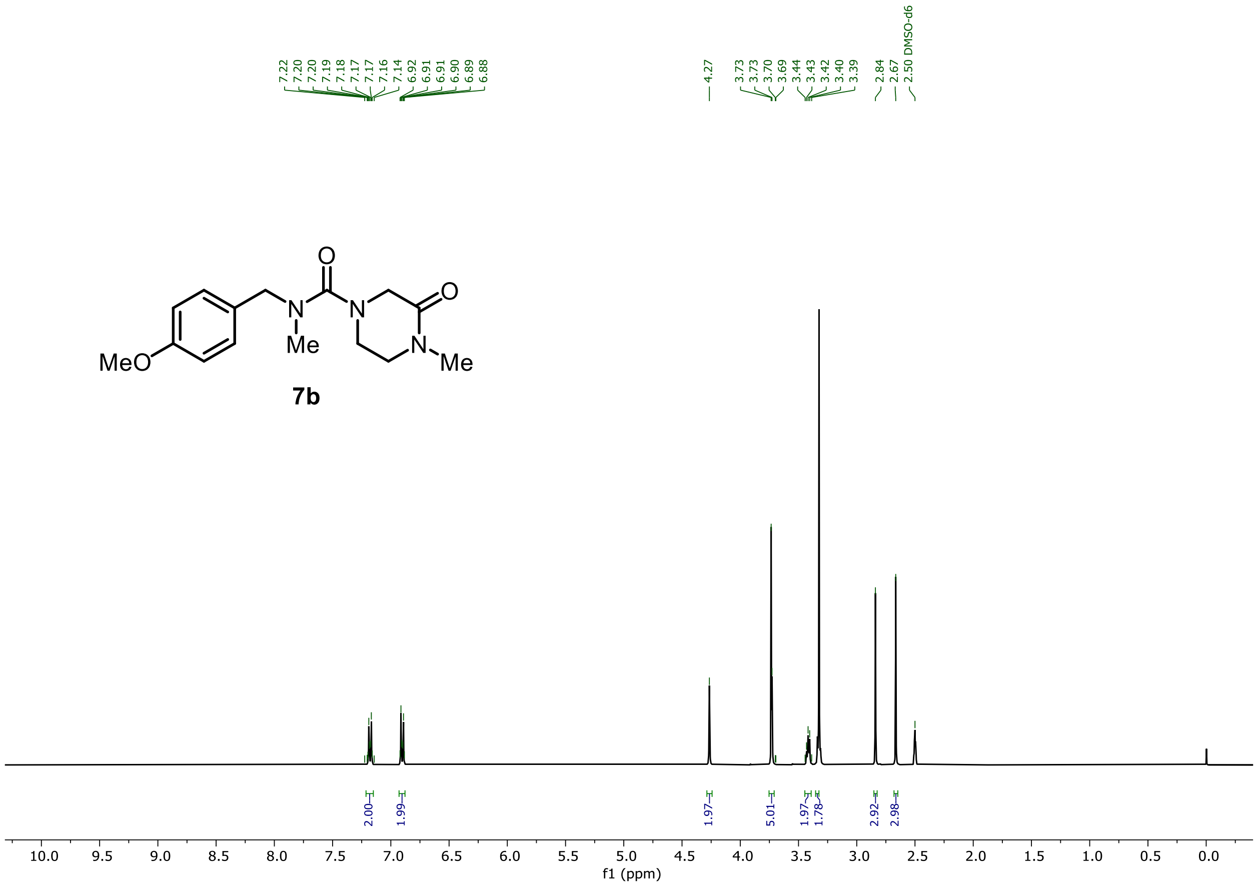

^{1}HNMR spectrum of **7b** run in DMSO-d_6 at 400 MHz.

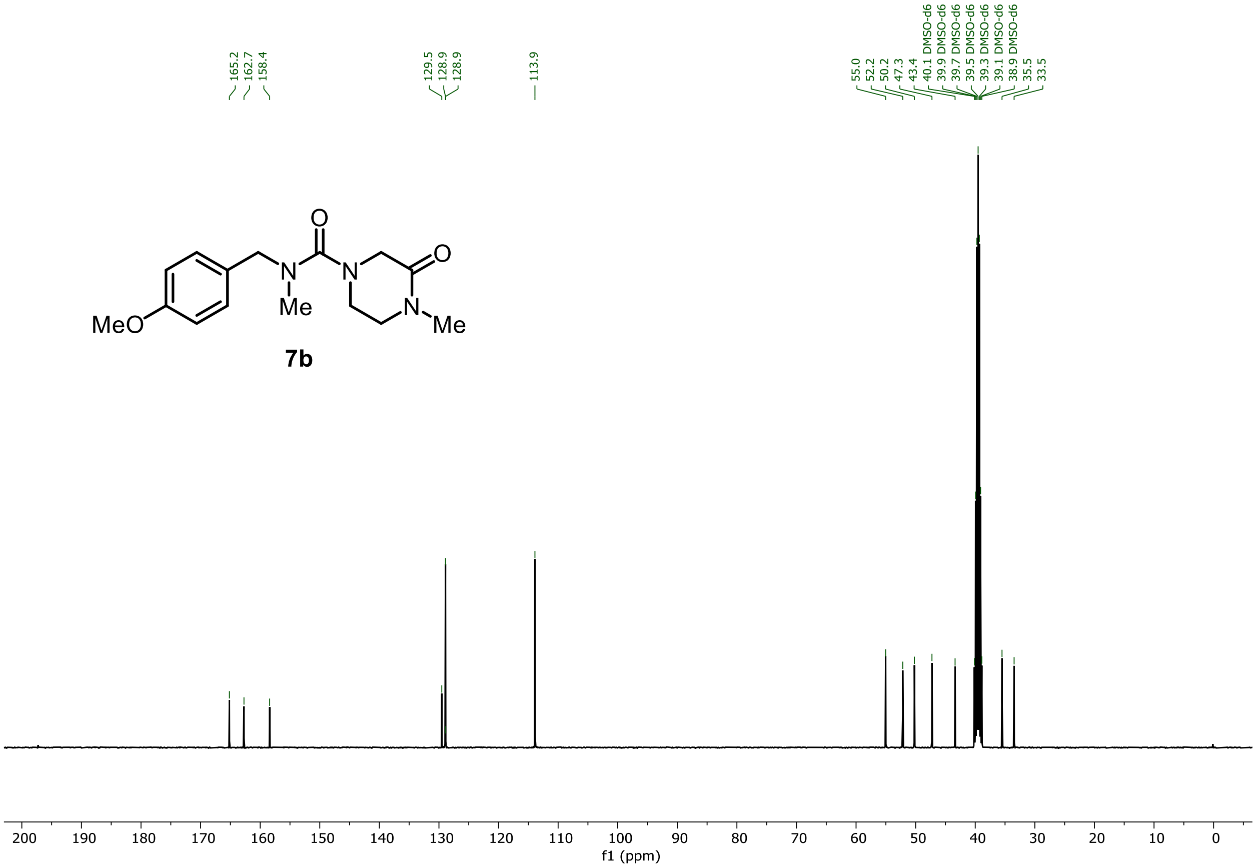

^{13}CNMR spectrum of **7b** run in DMSO-d_6 at 101 MHz.

3-(Cyanomethyl)-1-(4-methoxybenzyl)-1-methylurea (8b)

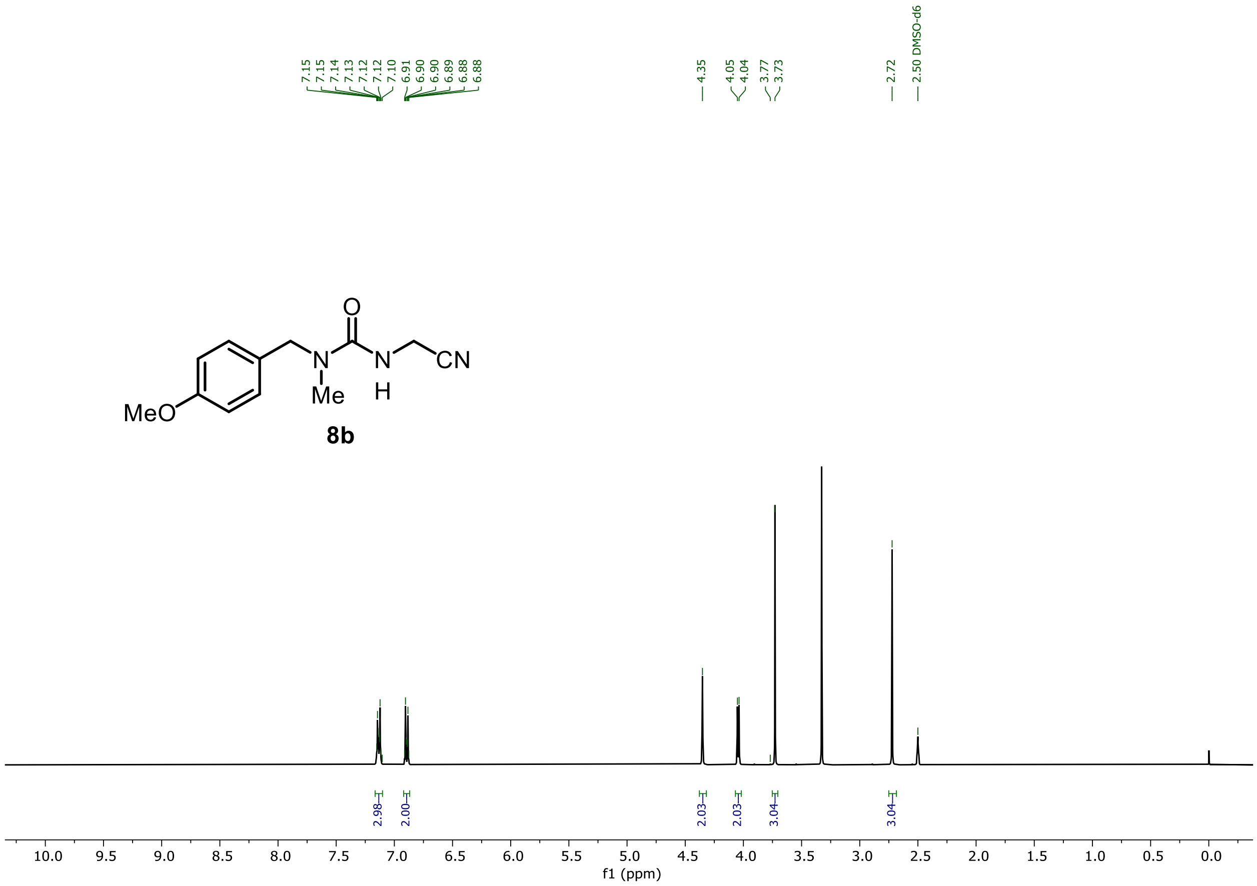

^{1}HNMR spectrum of **8b** run in DMSO-d_6 at 400 MHz.

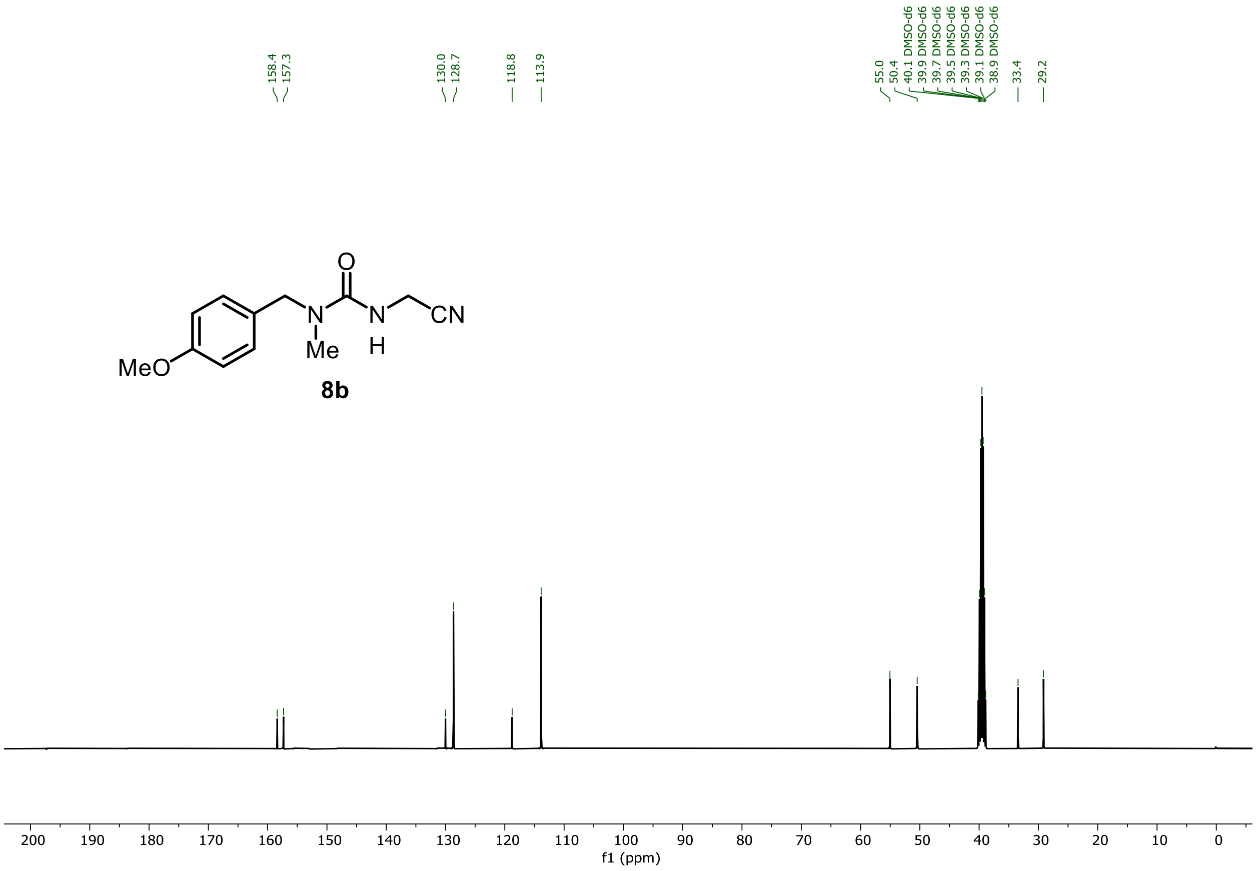

^{13}CNMR spectrum of **8b** run in DMSO-d_6 at 101 MHz.

Pyridin-3-ylmethyl (*S*)-methyl(2-oxotetrahydrofuran-3-yl)carbamate (9a)

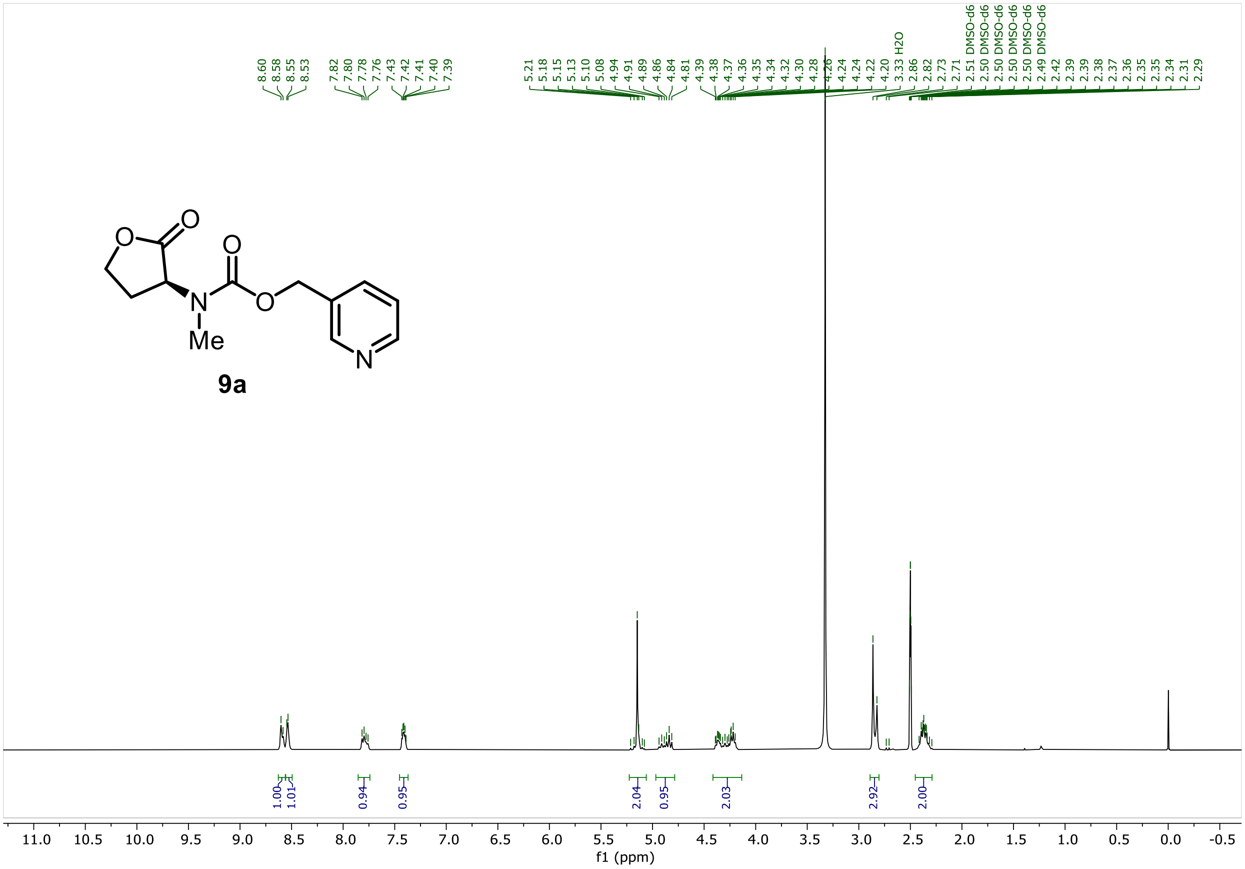

^{1}HNMR spectrum of **9a** run in DMSO-d_6 at 400 MHz.

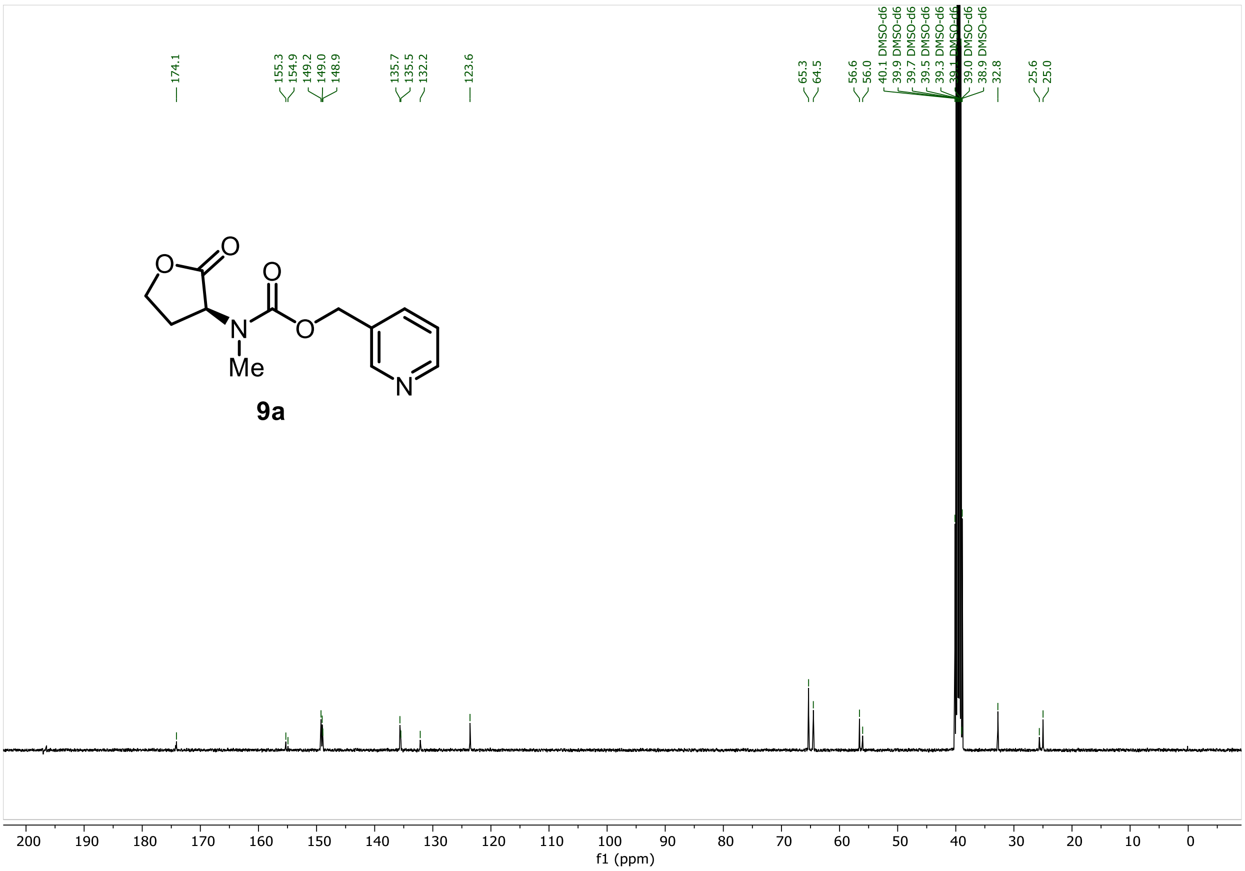

^{13}CNMR spectrum of **9a** run in DMSO-d_6 at 101 MHz.

(*S*)-*N*-Methyl-*N*-((2-oxo-3-(4-(3-oxomorpholino)phenyl)oxazolidin-5-yl)methyl)-2-phenylacetamide (S14)

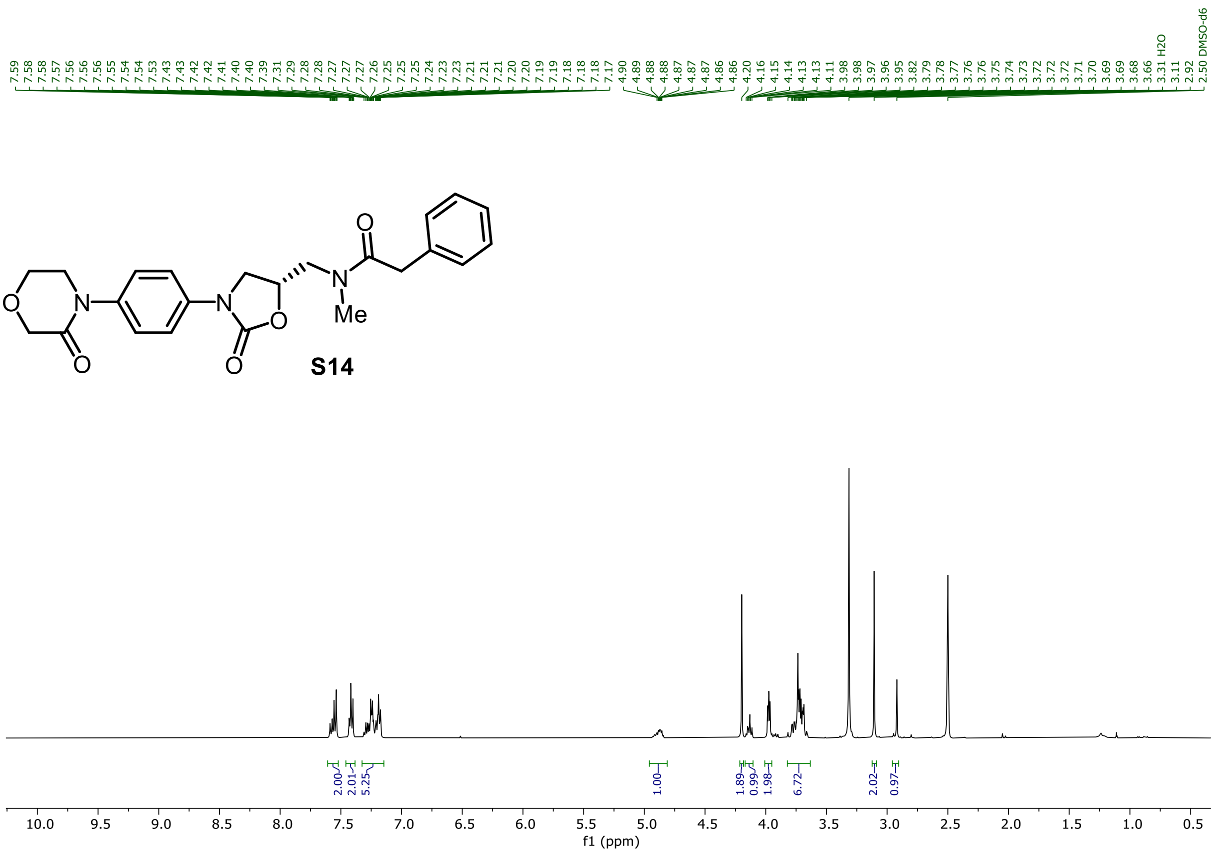

^{1}HNMR spectrum of **S14** run in DMSO-d_6 at 500 MHz.

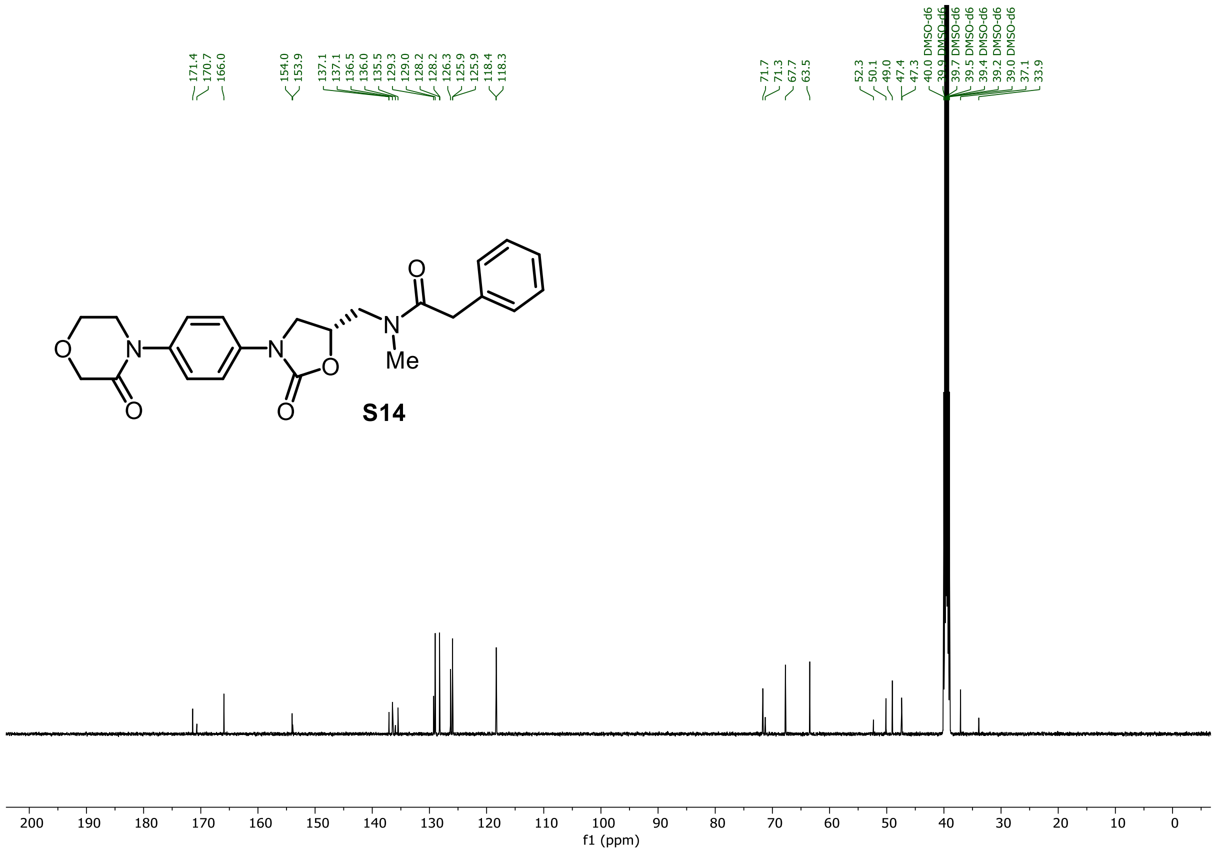

^{13}CNMR spectrum of **S14** run in DMSO-d_6 at 126 MHz.

(*S*)-*N*-Methyl-*N*-((2-oxo-3-(4-(3-oxomorpholino)phenyl)oxazolidin-5-yl)methyl)benzamide (*S*15)

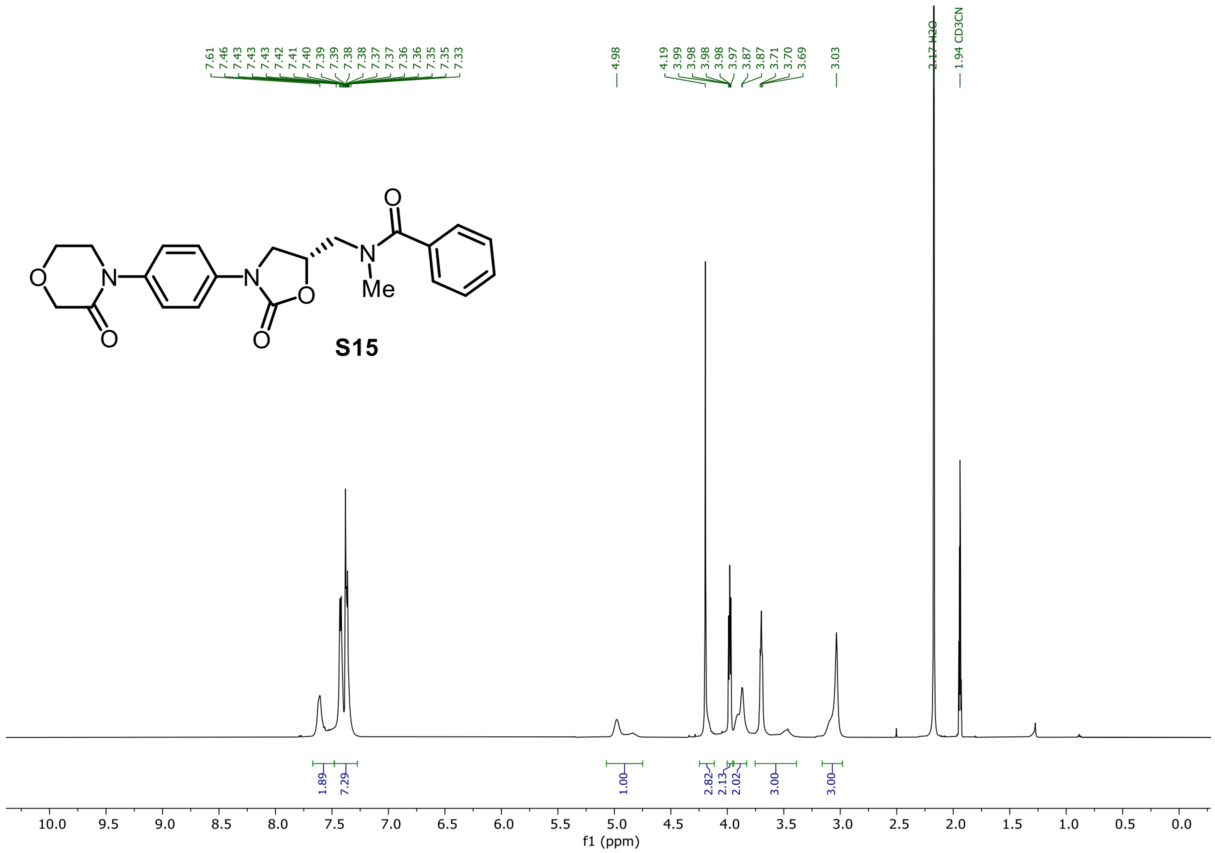

^{1}HNMR spectrum of **S15** run in CD_3CN at 500 MHz.

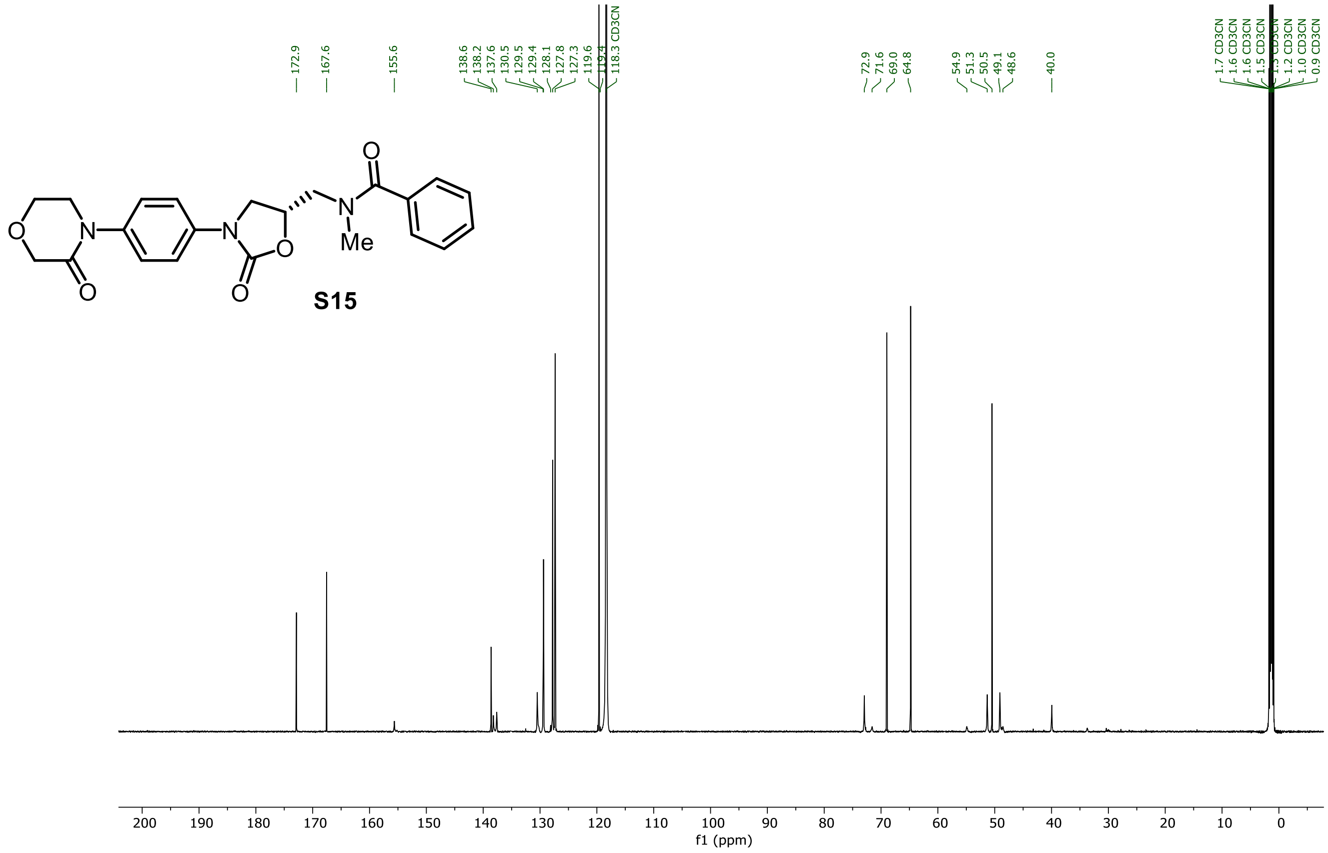

^{13}CNMR spectrum of **S15** run in CD_3CN at 126 MHz.

2-(2-Methyl-5-nitro-1*H*-imidazol-1-yl)ethyl benzyl(methyl)carbamate (S17)

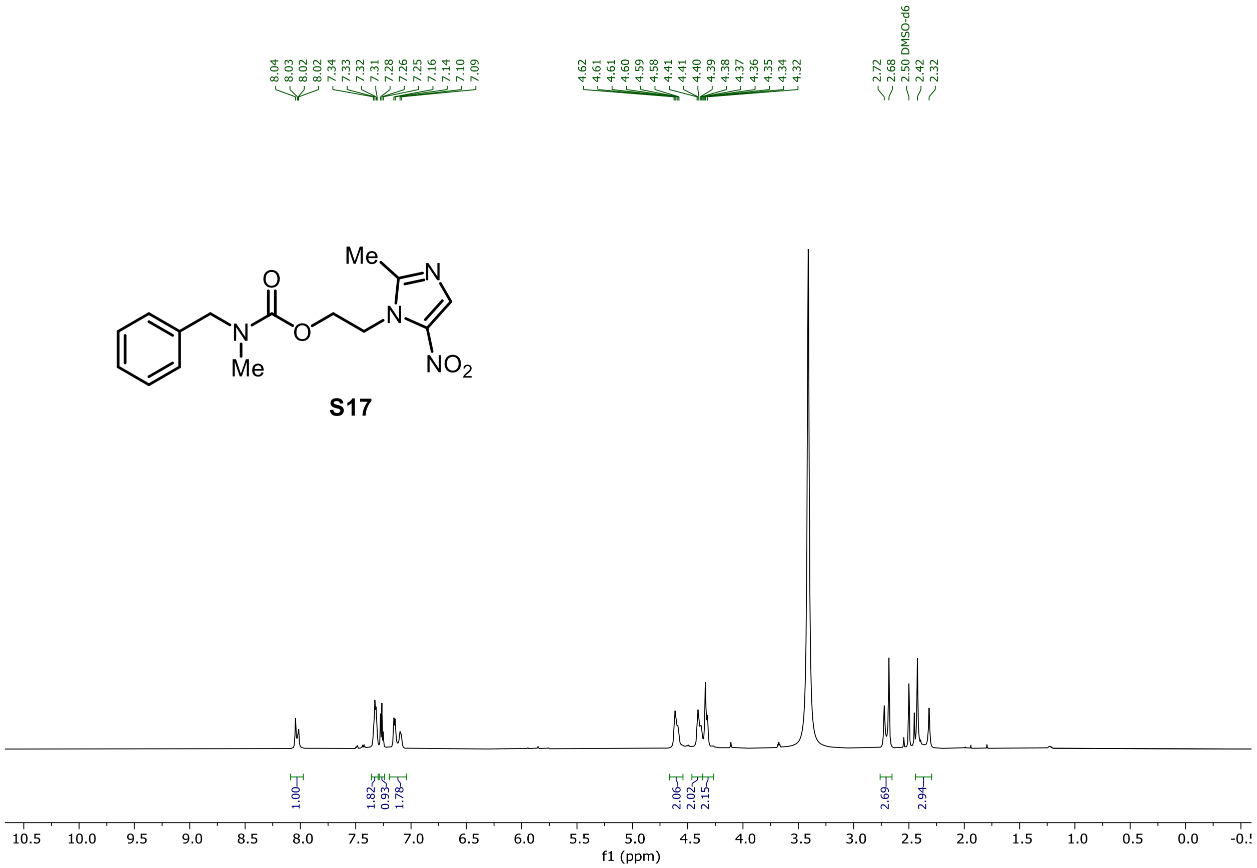

^{1}HNMR spectrum of **S17** run in DMSO-d_6 600 MHz.

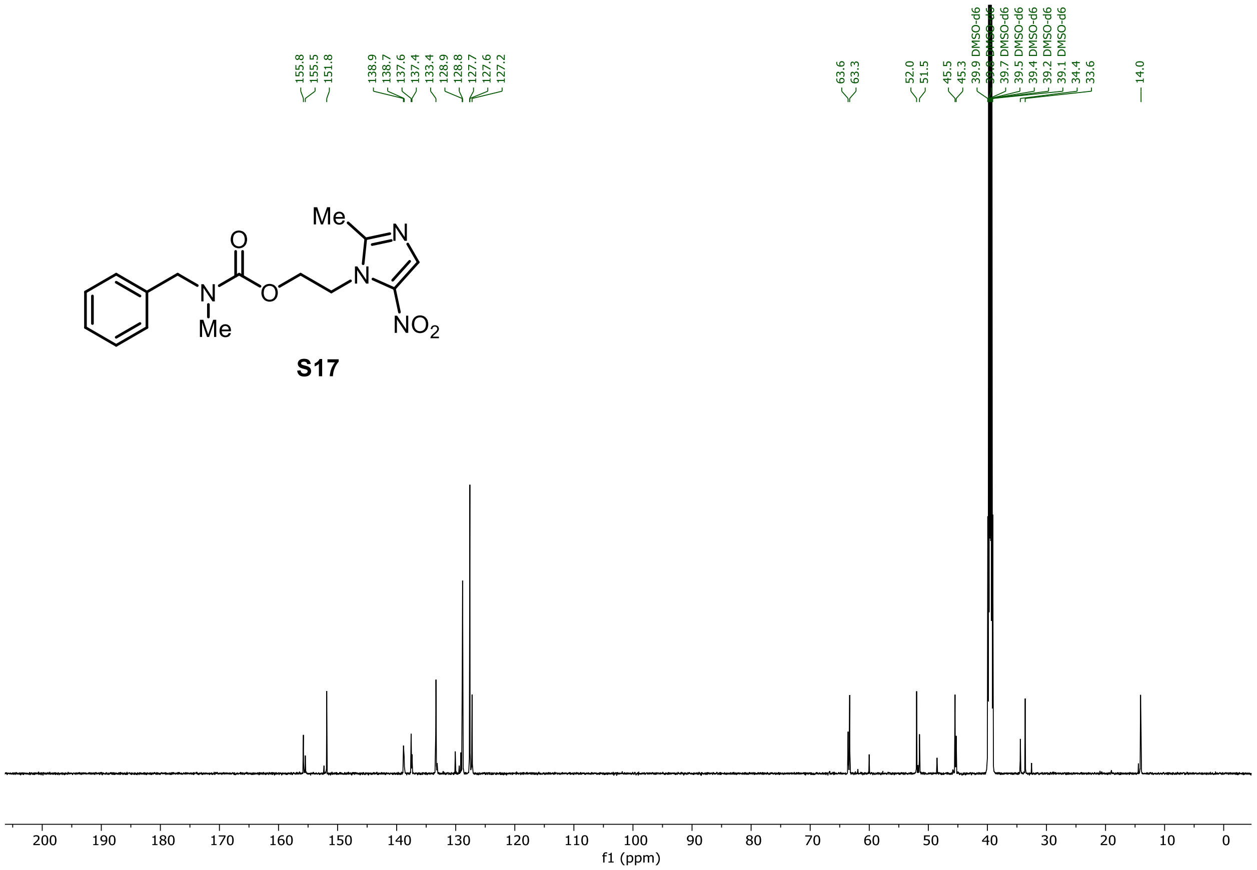

^{13}CNMR spectrum of **S17** run in DMSO-d_6 at 151 MHz.

2-(2-Methyl-5-nitro-1*H*-imidazol-1-yl)ethyl methyl(phenyl)carbamate (S18)

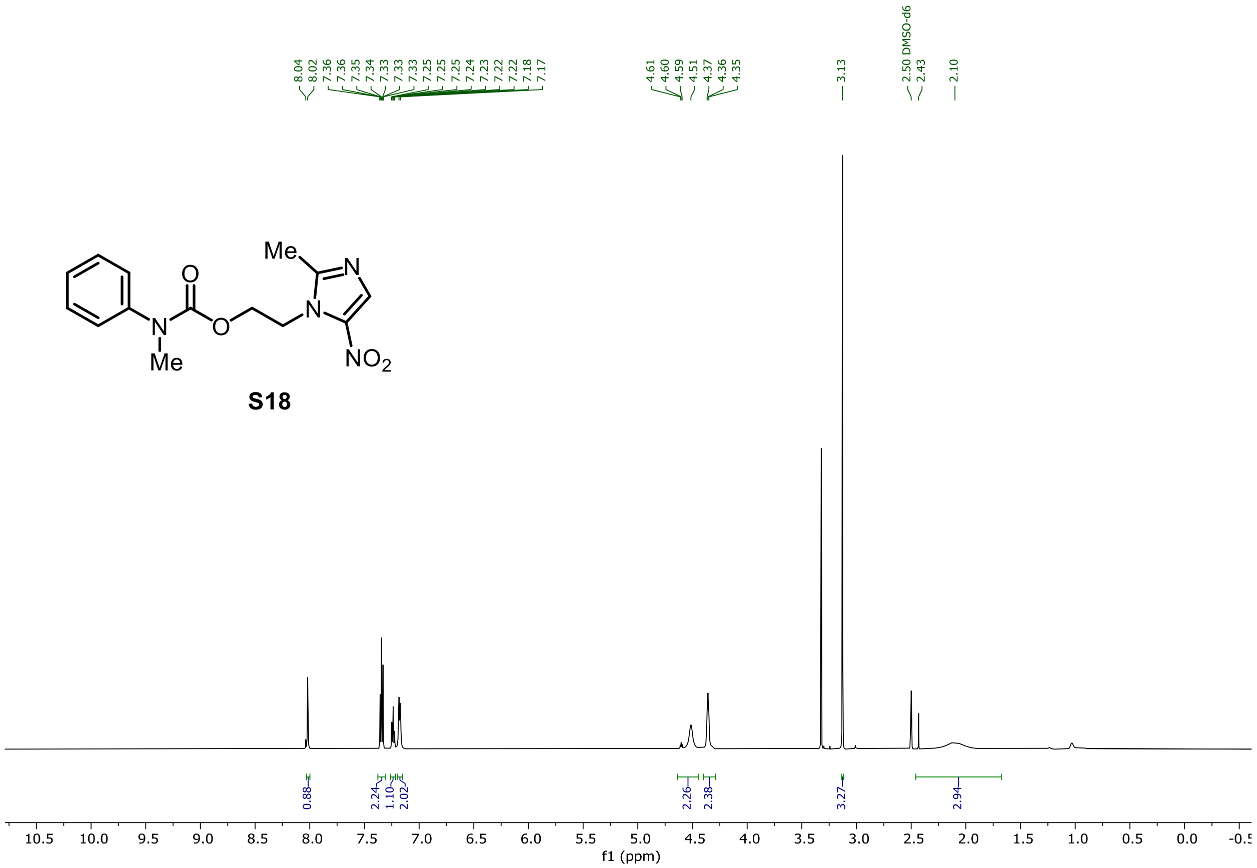

^{1}HNMR spectrum of **S18** run in DMSO-d_6 600 MHz.

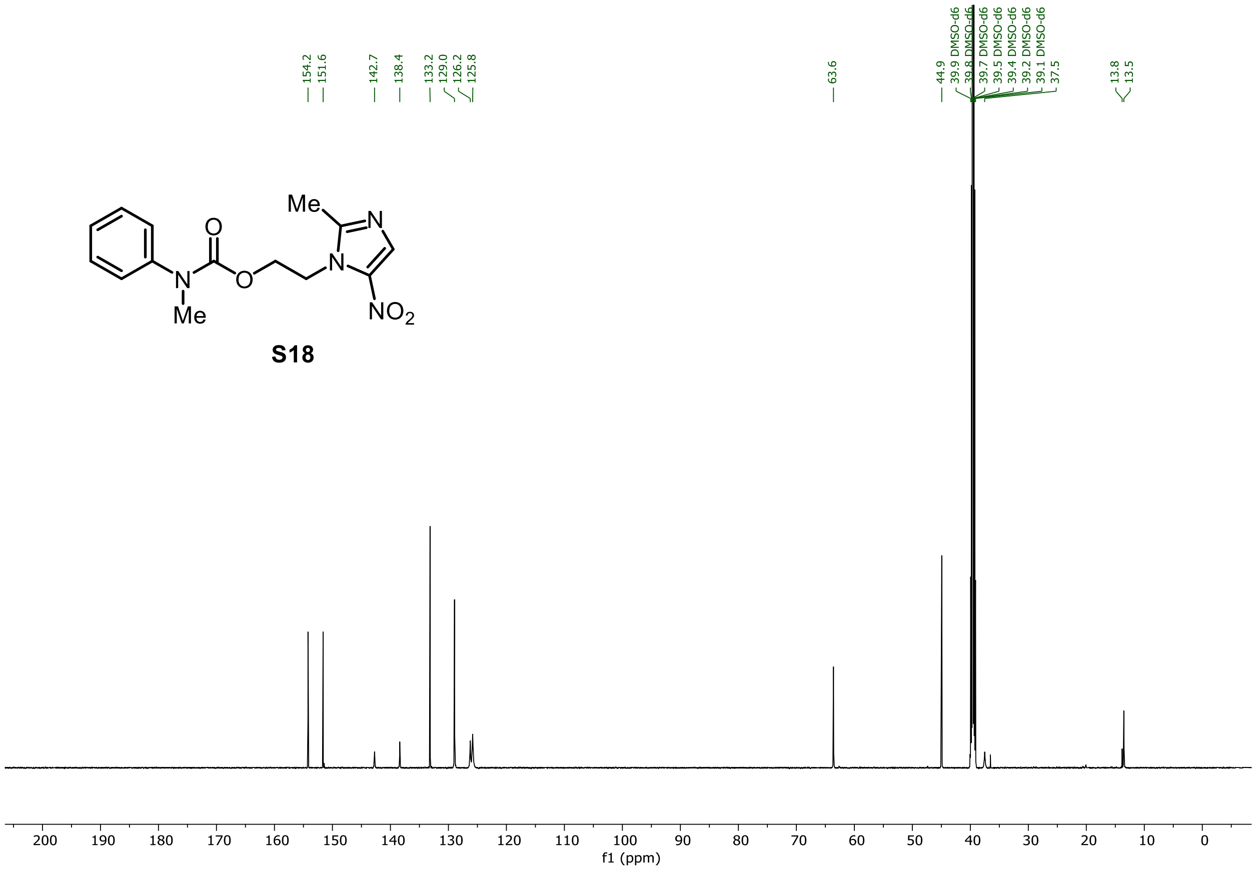

^{13}CNMR spectrum of **S18** run in DMSO-d_6 at 151 MHz.

2-(4-(2-(5-Chloro-2-oxobenzo[*d*]thiazol-3(2*H*)-yl)acetyl)piperazin-1-yl)ethyl dimethylcarbamate (S19)

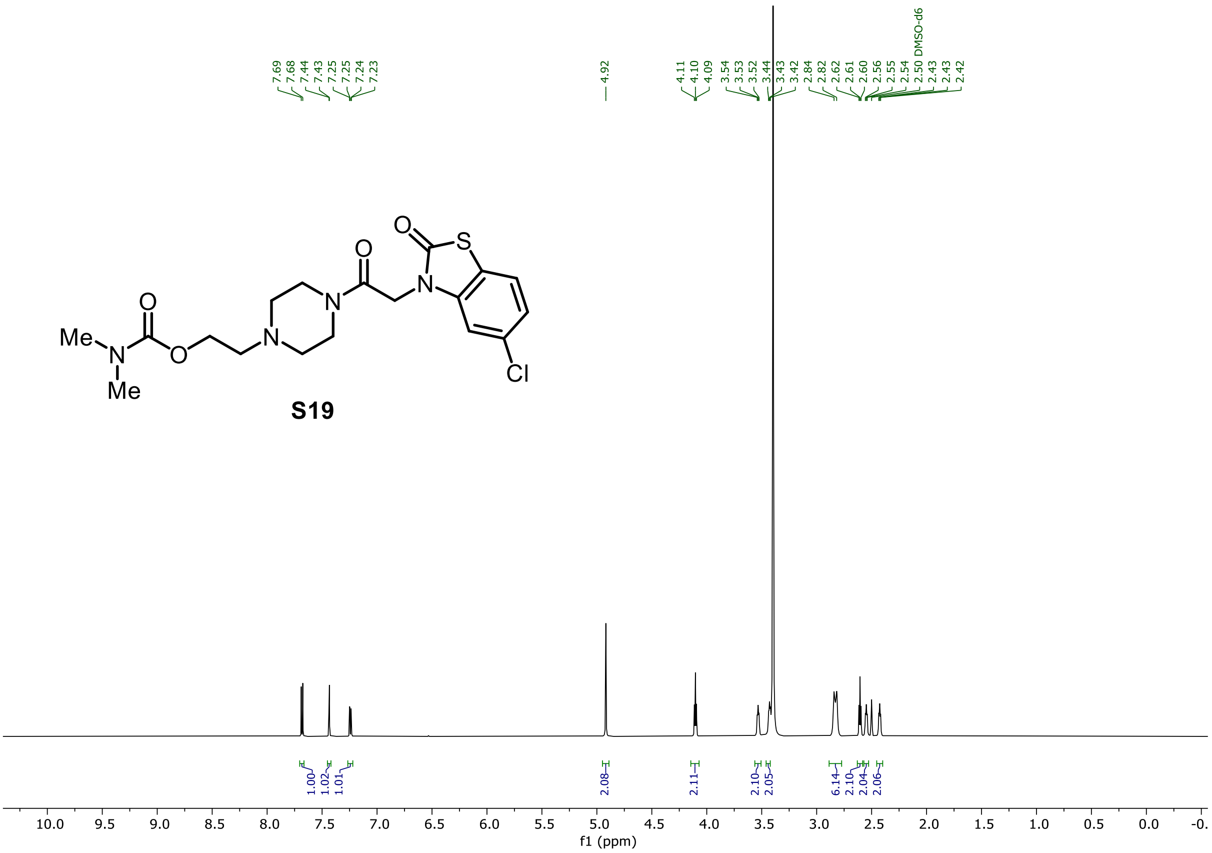

^{1}HNMR spectrum of **S19** run in DMSO-d_6 600 MHz.

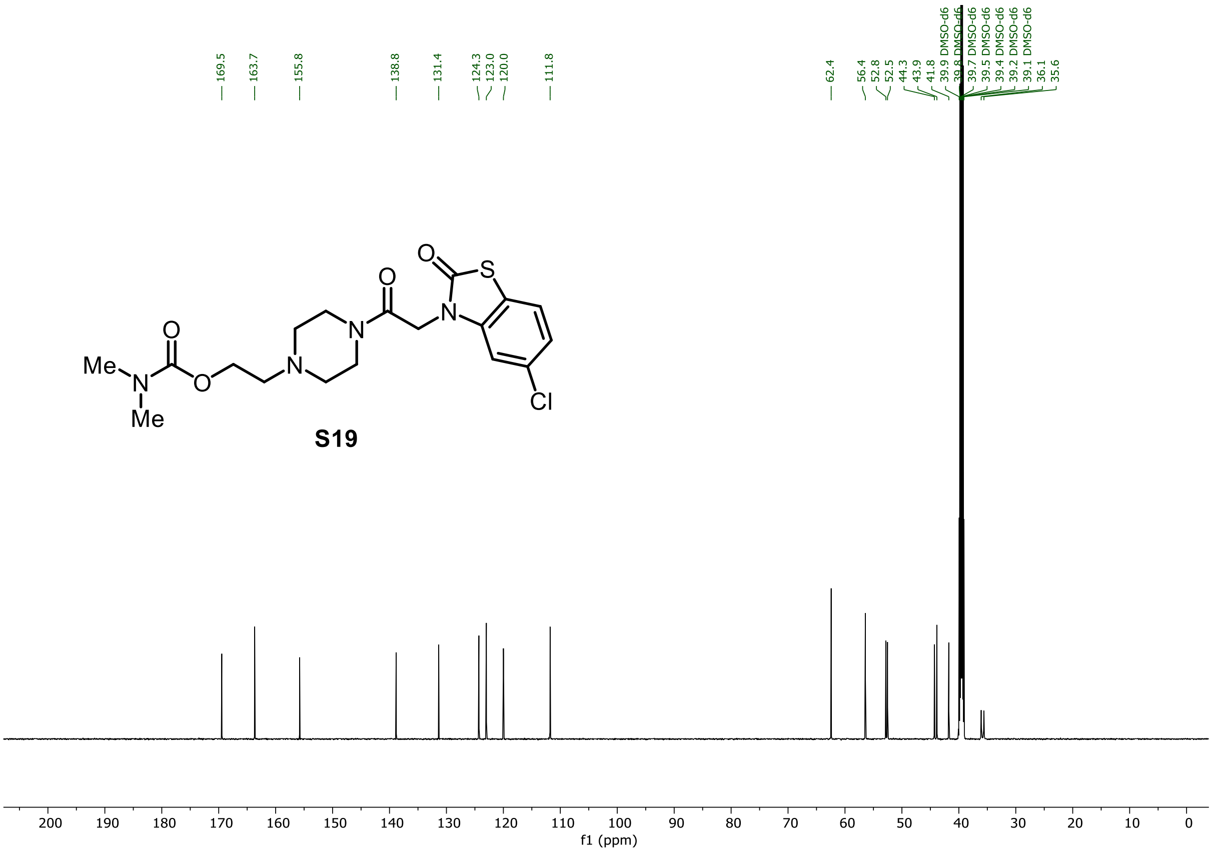

^{13}CNMR spectrum of **S19** run in DMSO-d_6 at 151 MHz.

2-(4-(2-(5-Chloro-2-oxobenzo[d]thiazol-3(2*H*)-yl)acetyl)piperazin-1-yl)ethyl cyclopropyl(methyl)carbamate (*S*20)

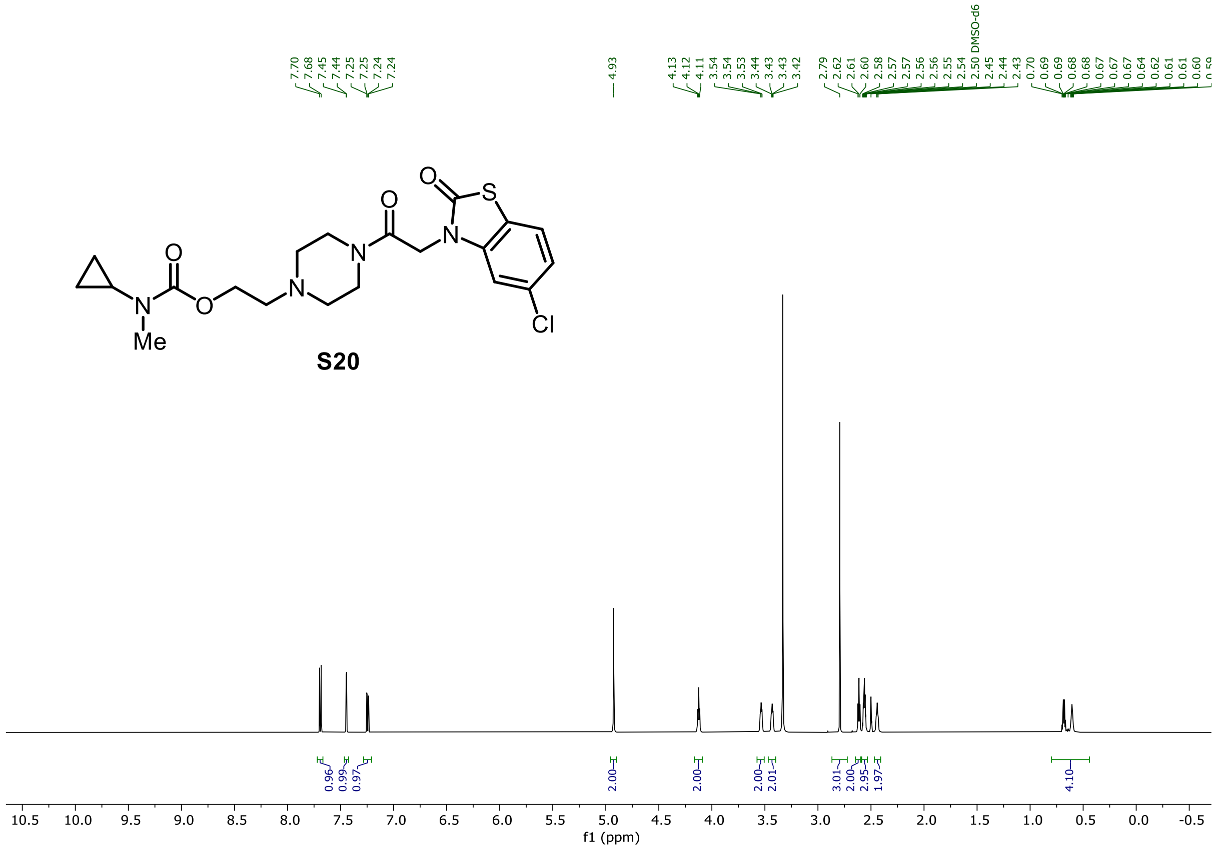

^{1}HNMR spectrum of **S20** run in DMSO-d_6 600 MHz.

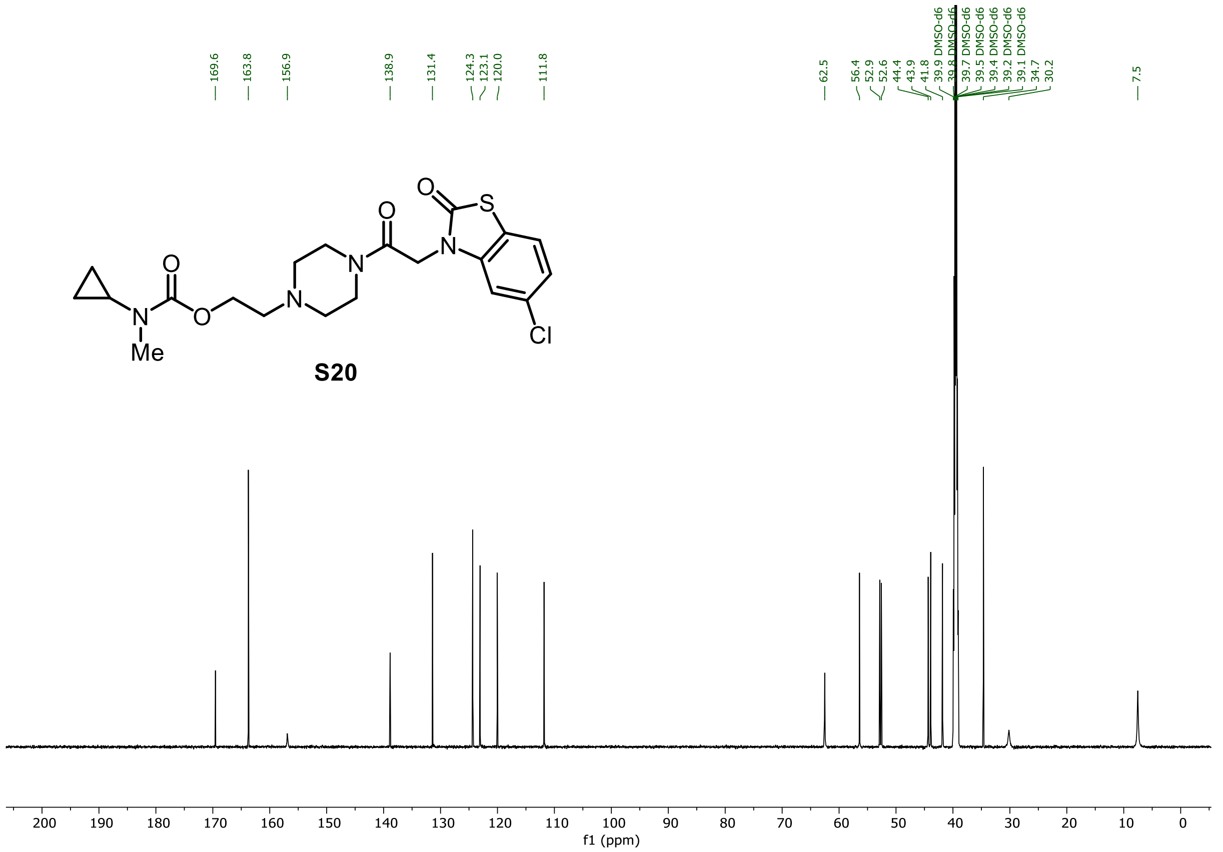

^{13}CNMR spectrum of **S20** run in DMSO-d_6 at 151 MHz.

2-(4-(2-(5-Chloro-2-oxobenzo[*d*]thiazol-3(2*H*)-yl)acetyl)piperazin-1-yl)ethyl benzyl(methyl)carbamate (*S*21)

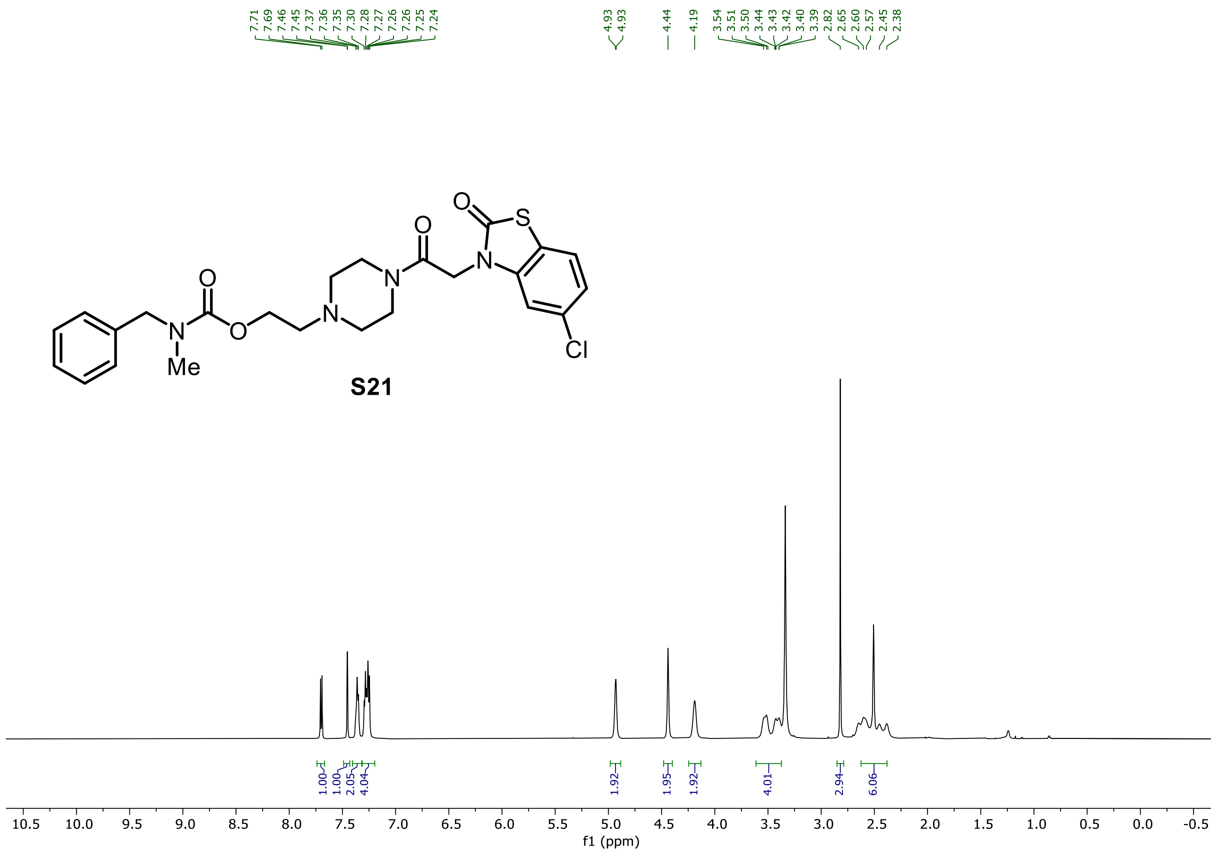

^{1}HNMR spectrum of **S21** run in DMSO-d_6 600 MHz.

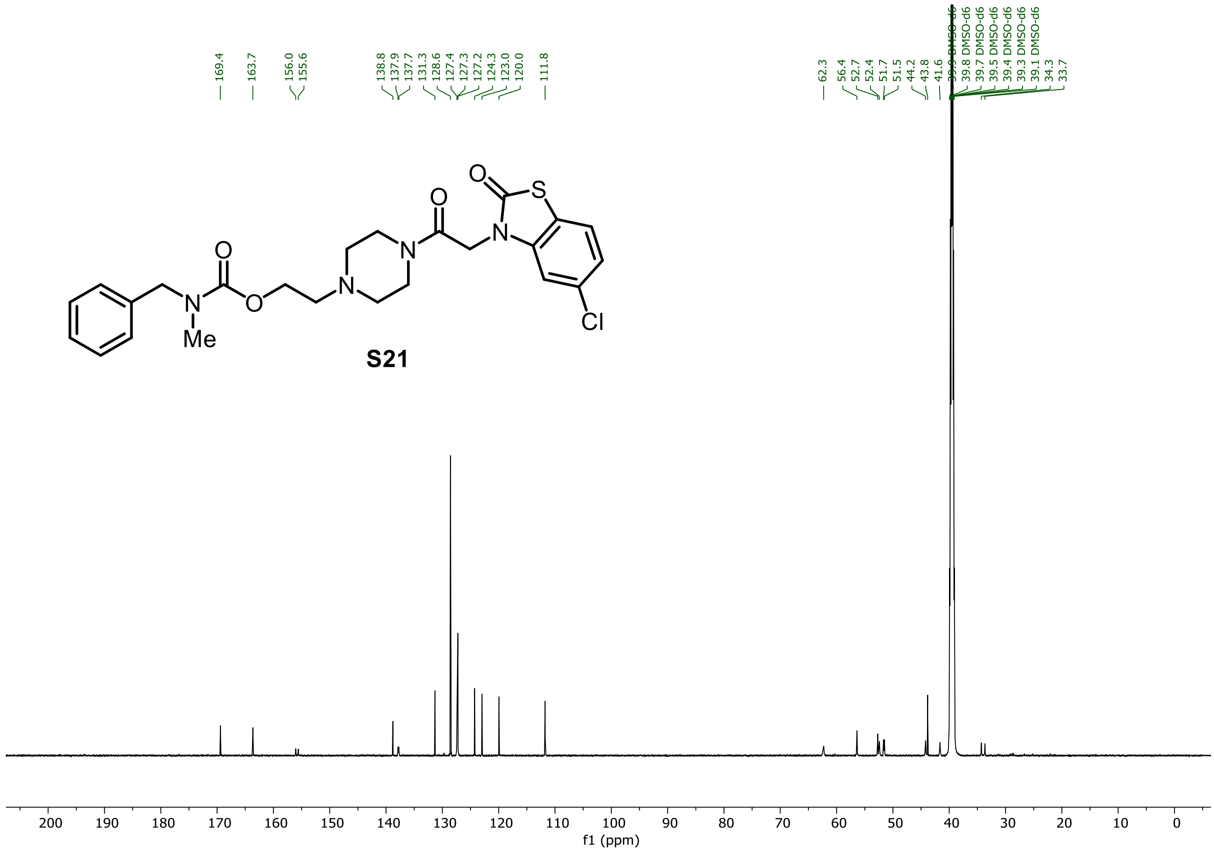

^{13}CNMR spectrum of **S21** run in DMSO-d_6 at 151 MHz.

2-(4-(2-(5-Chloro-2-oxobenzo[*d*]thiazol-3(2*H*)-yl)acetyl)piperazin-1-yl)ethyl methyl(phenyl)carbamate (S22)

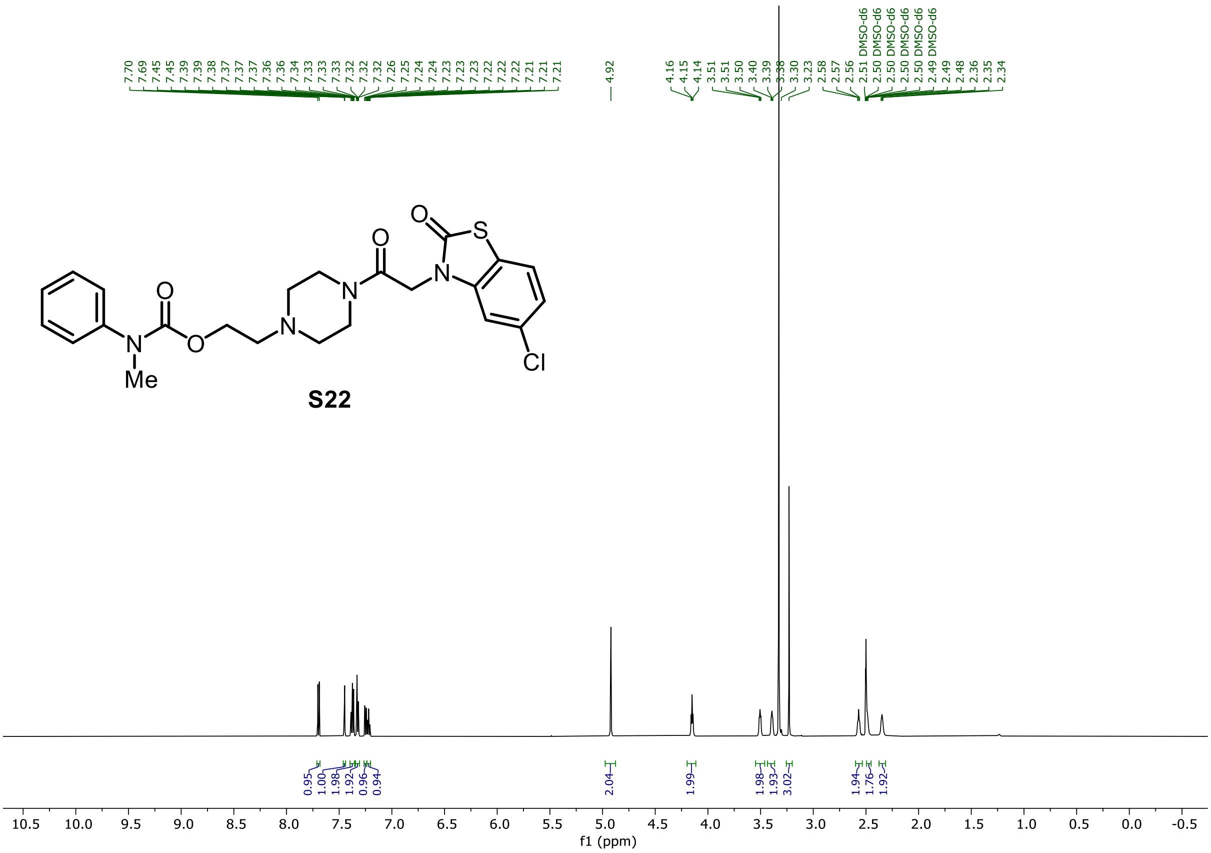

^{1}HNMR spectrum of **S22** run in DMSO-d_6 600 MHz.

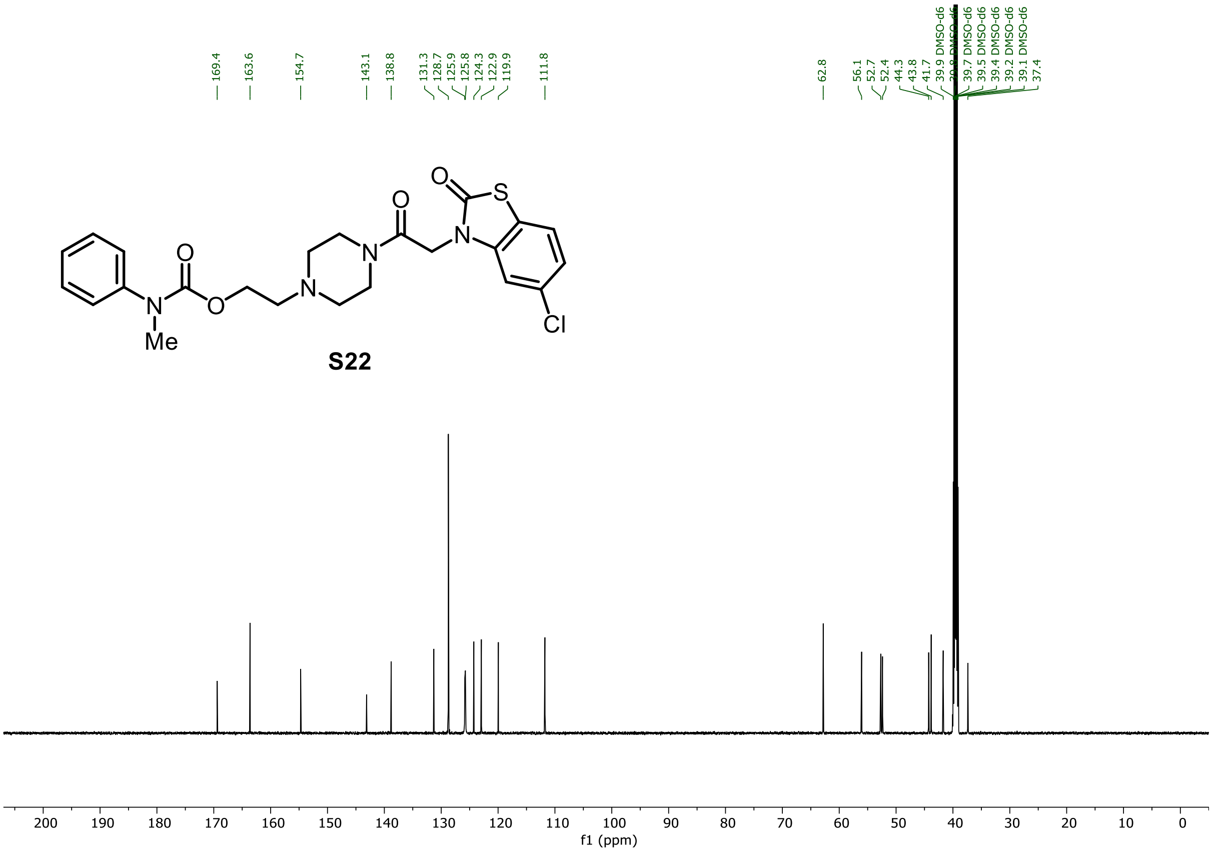

^{13}CNMR spectrum of **S22** run in DMSO-d_6 at 151 MHz.

4-Acetamidophenyl dimethylcarbamate (S23)

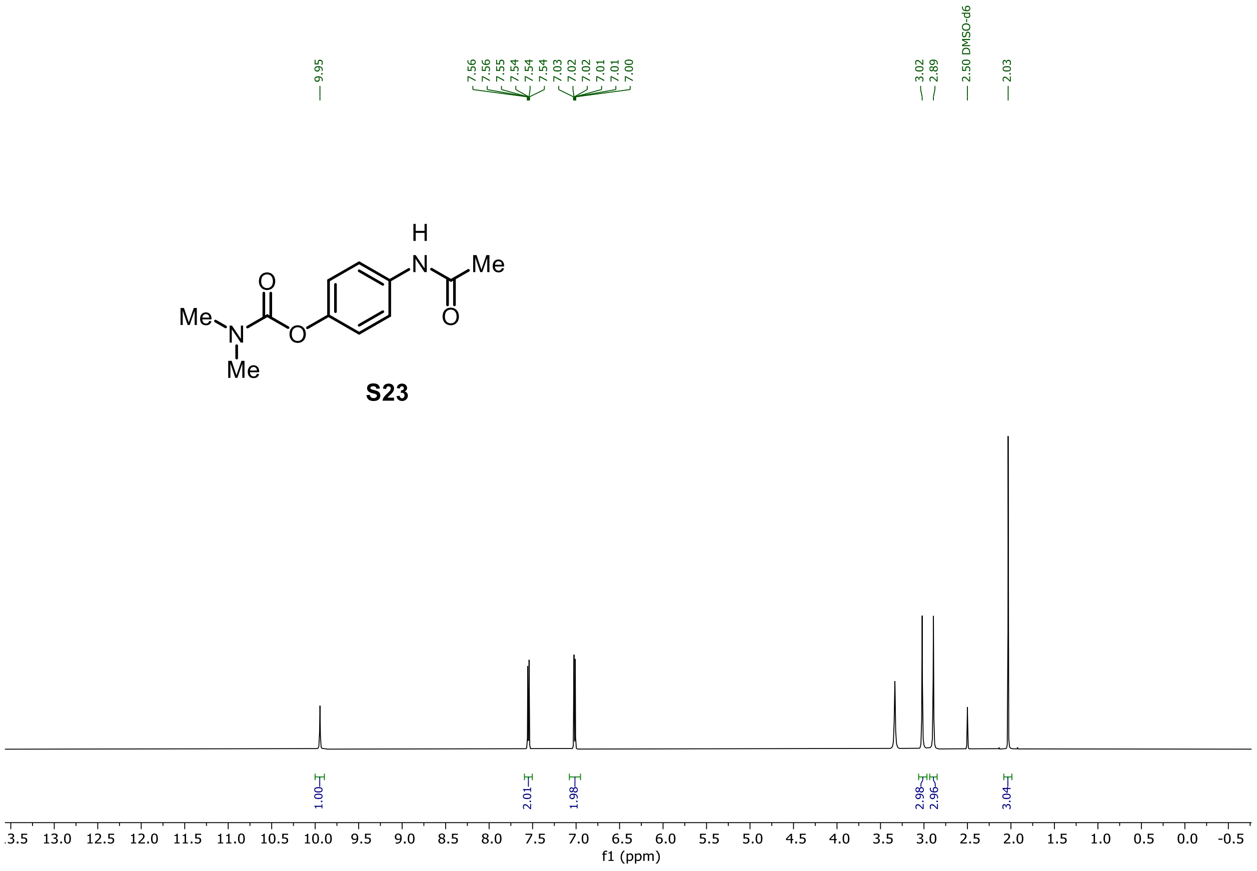

^{1}HNMR spectrum of **S23** run in DMSO-d_6 600 MHz.

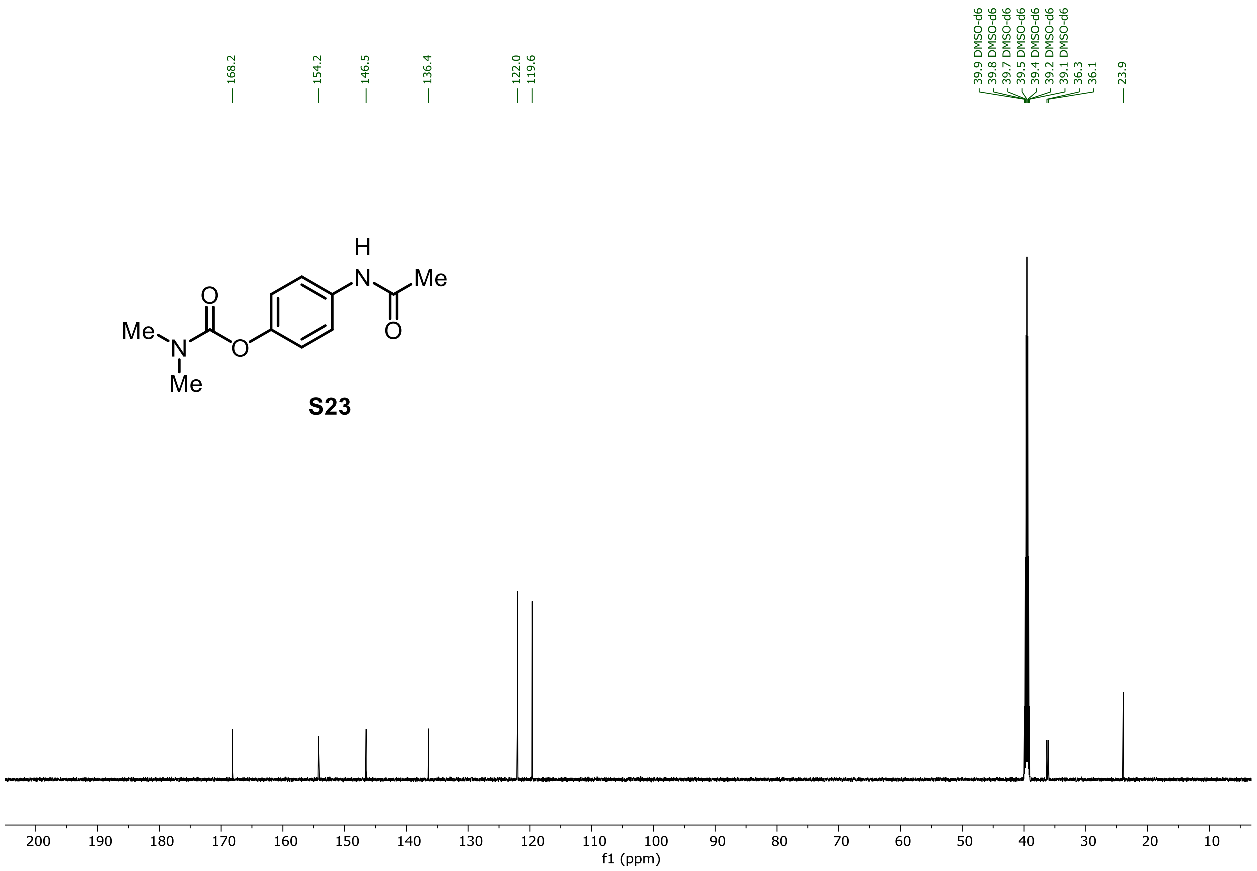

^{13}CNMR spectrum of **S23** run in DMSO-d_6 at 151 MHz.

4-Acetamidophenyl cyclopropyl(methyl)carbamate (S24)

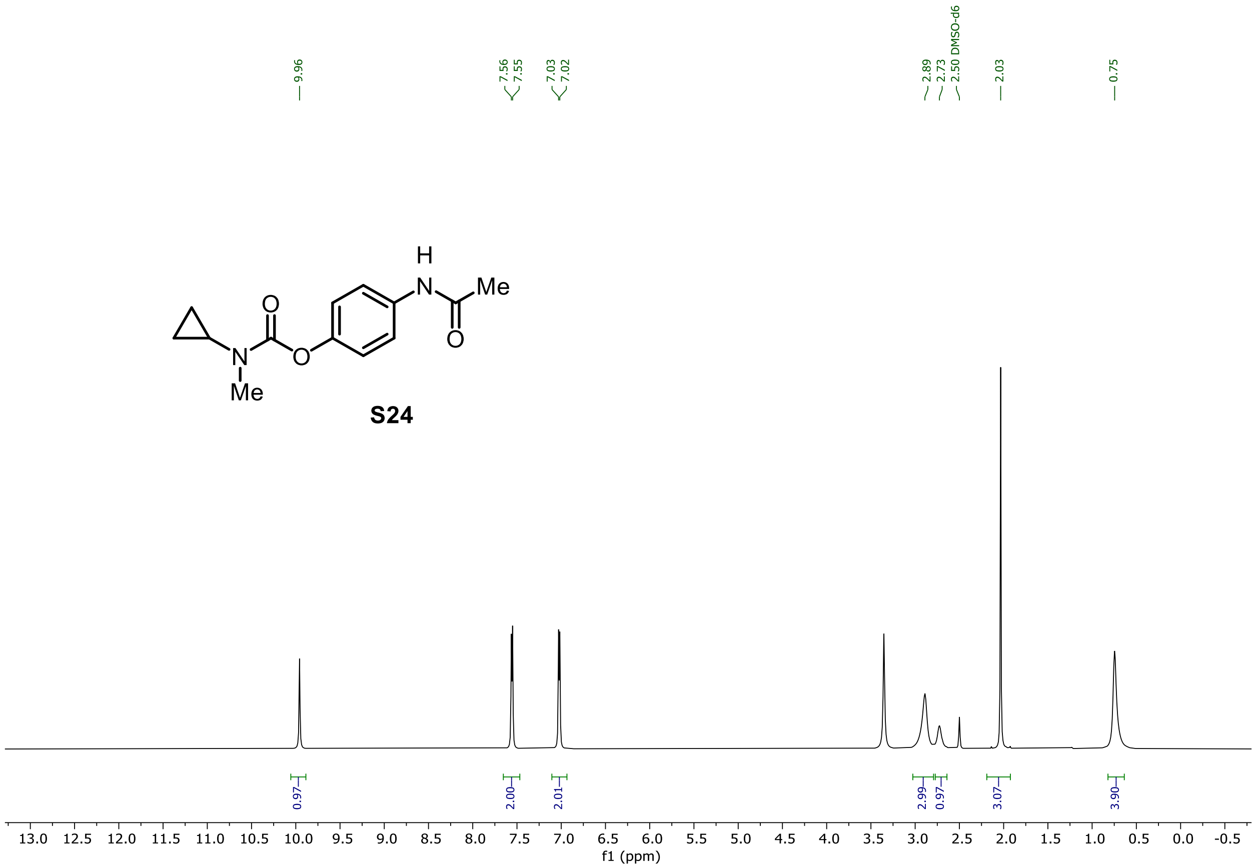

^{1}HNMR spectrum of **S24** run in DMSO-d_6 600 MHz.

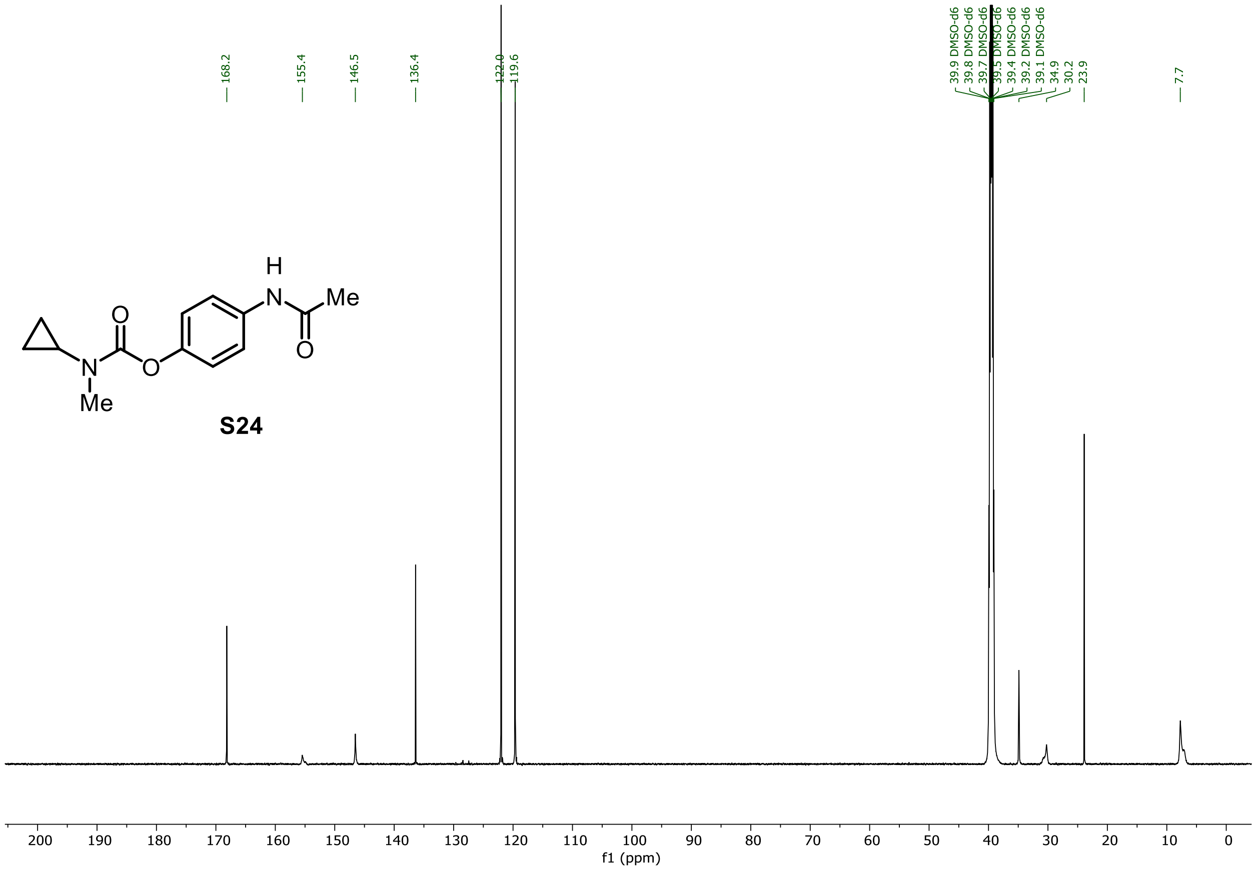

^{13}CNMR spectrum of **S24** run in DMSO-d_6 at 151 MHz.

4-Acetamidophenyl benzyl(methyl)carbamate (*S*25)

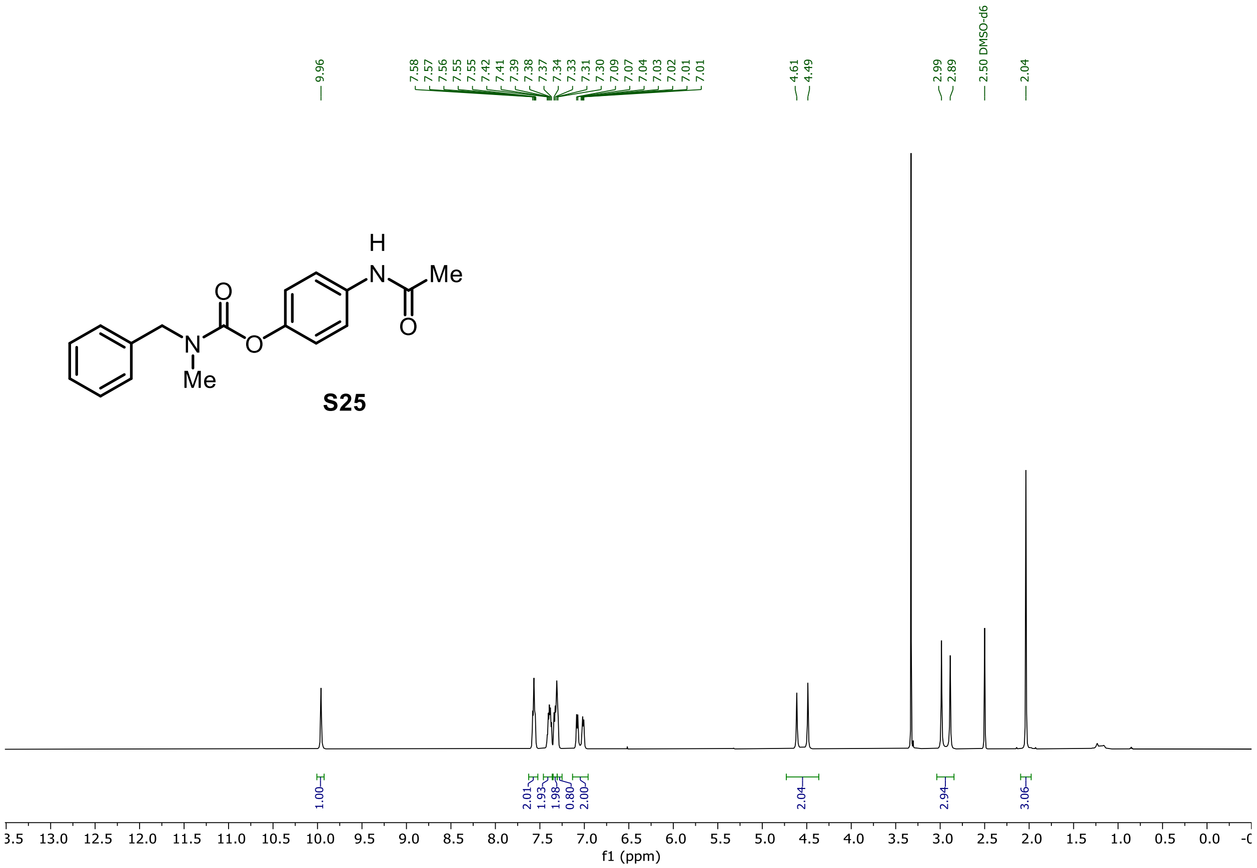

^{1}HNMR spectrum of **S25** run in DMSO-d_6 600 MHz.

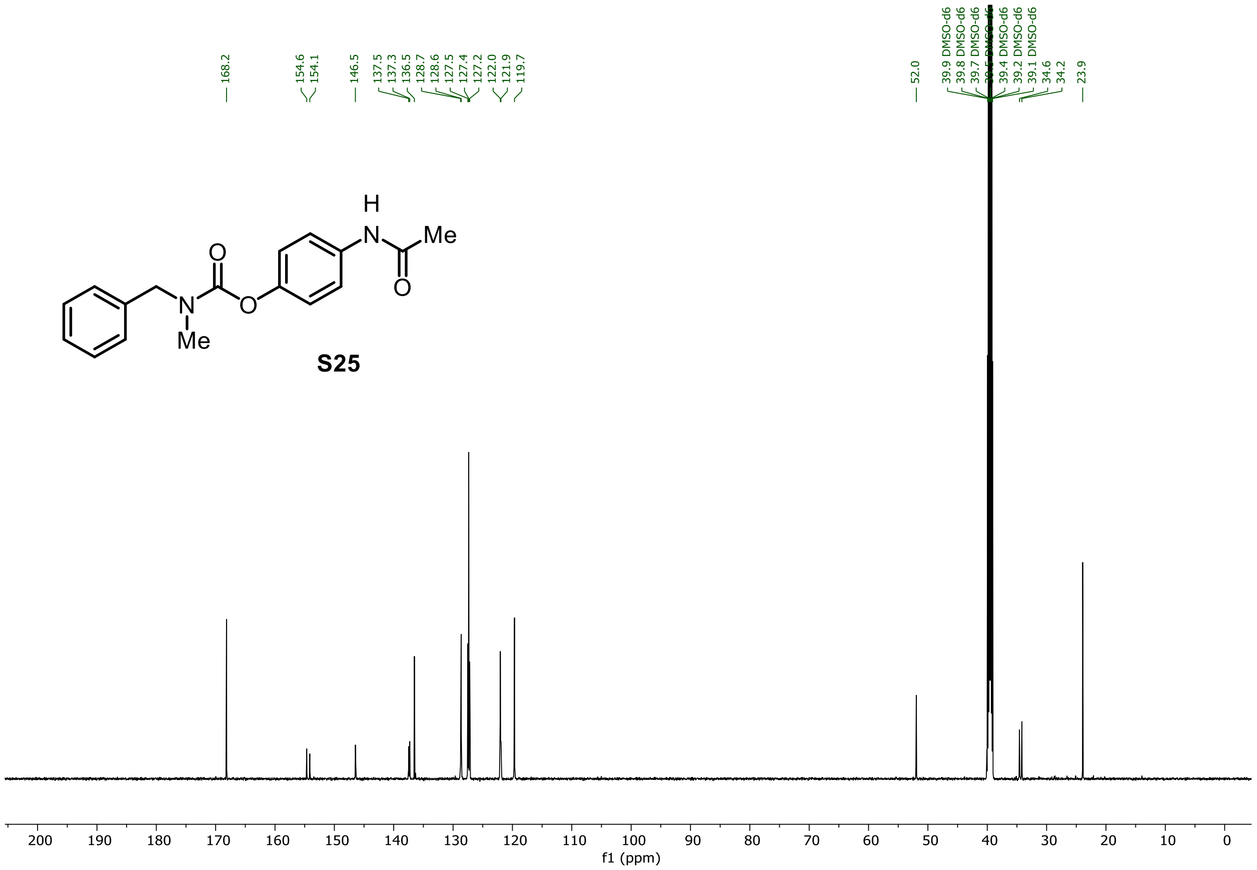

^{13}CNMR spectrum of **S25** run in DMSO-d_6 at 151 MHz.

4-Acetamidophenyl methyl(phenyl)carbamate (S26)

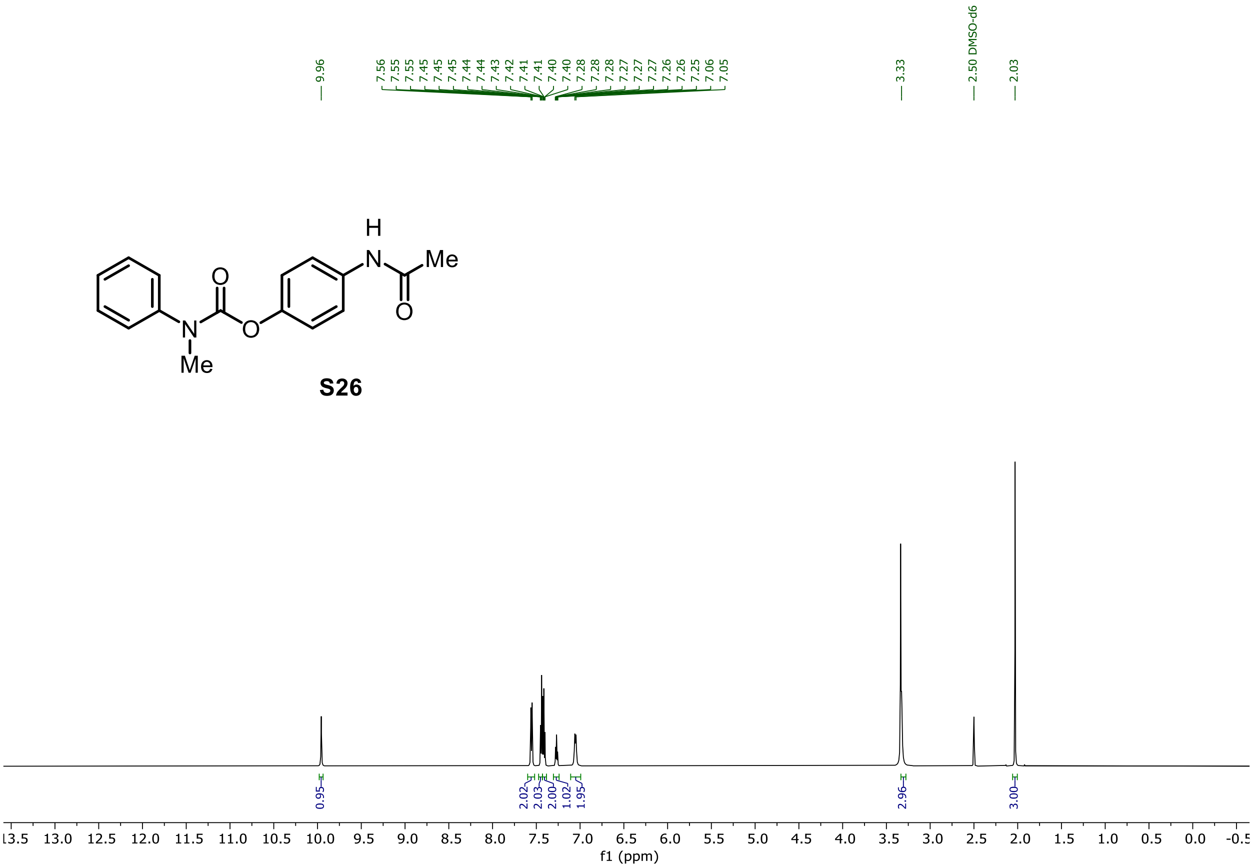

^{1}HNMR spectrum of **S26** run in DMSO-d_6 600 MHz.

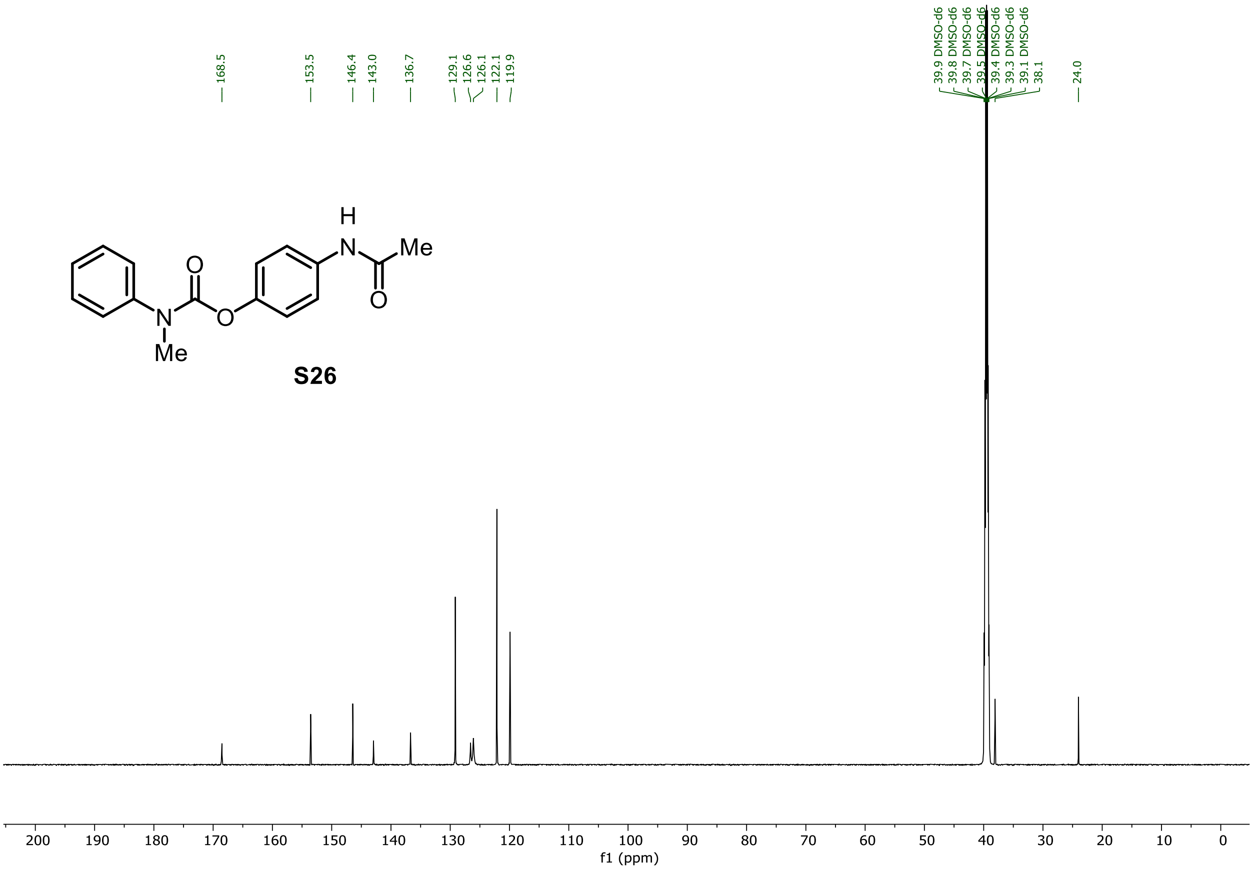

^{13}CNMR spectrum of **S26** run in DMSO-d_6 at 151 MHz.

3.6 *N*-H analogues

4-(Morpholinomethyl)-*N*-(3,4,5-trimethoxyphenyl)benzamide (3a)

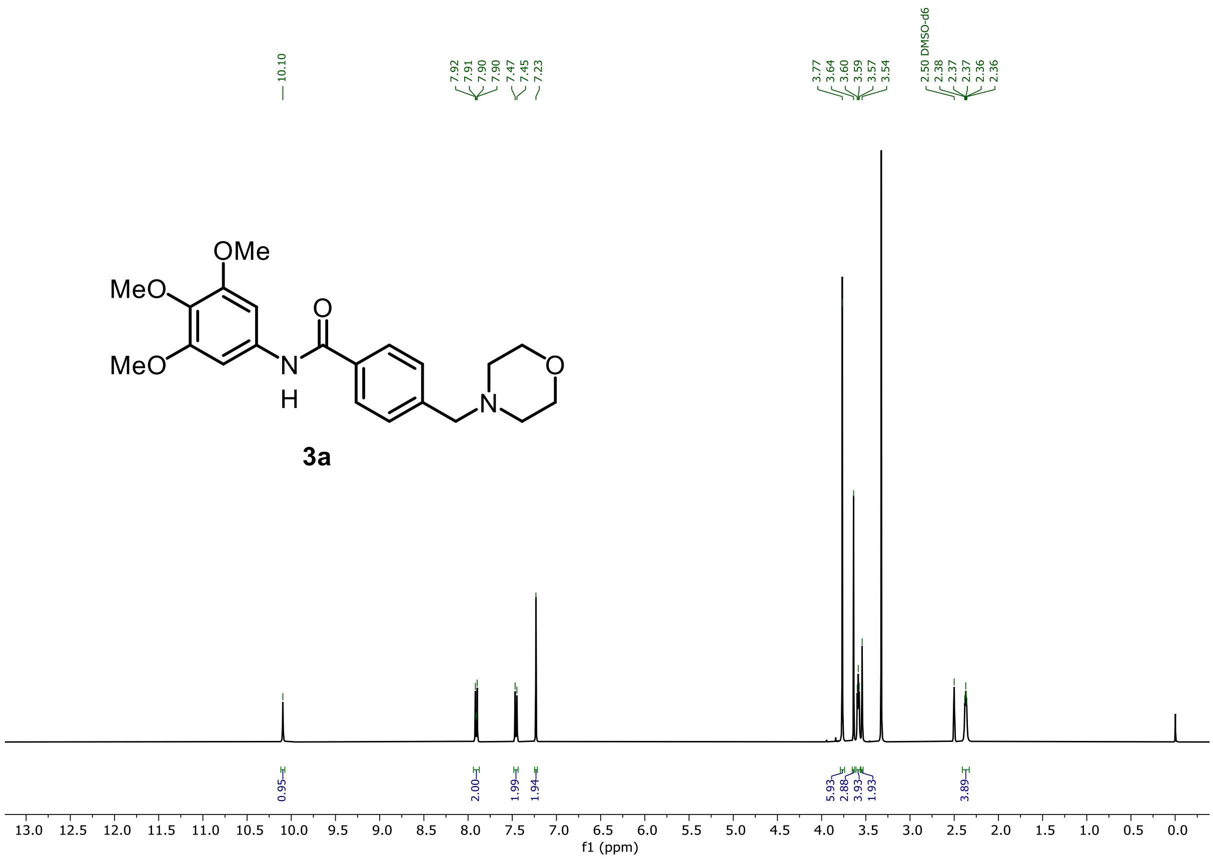

^{1}HNMR spectrum of **3a** run in DMSO-d_6 at 400 MHz.

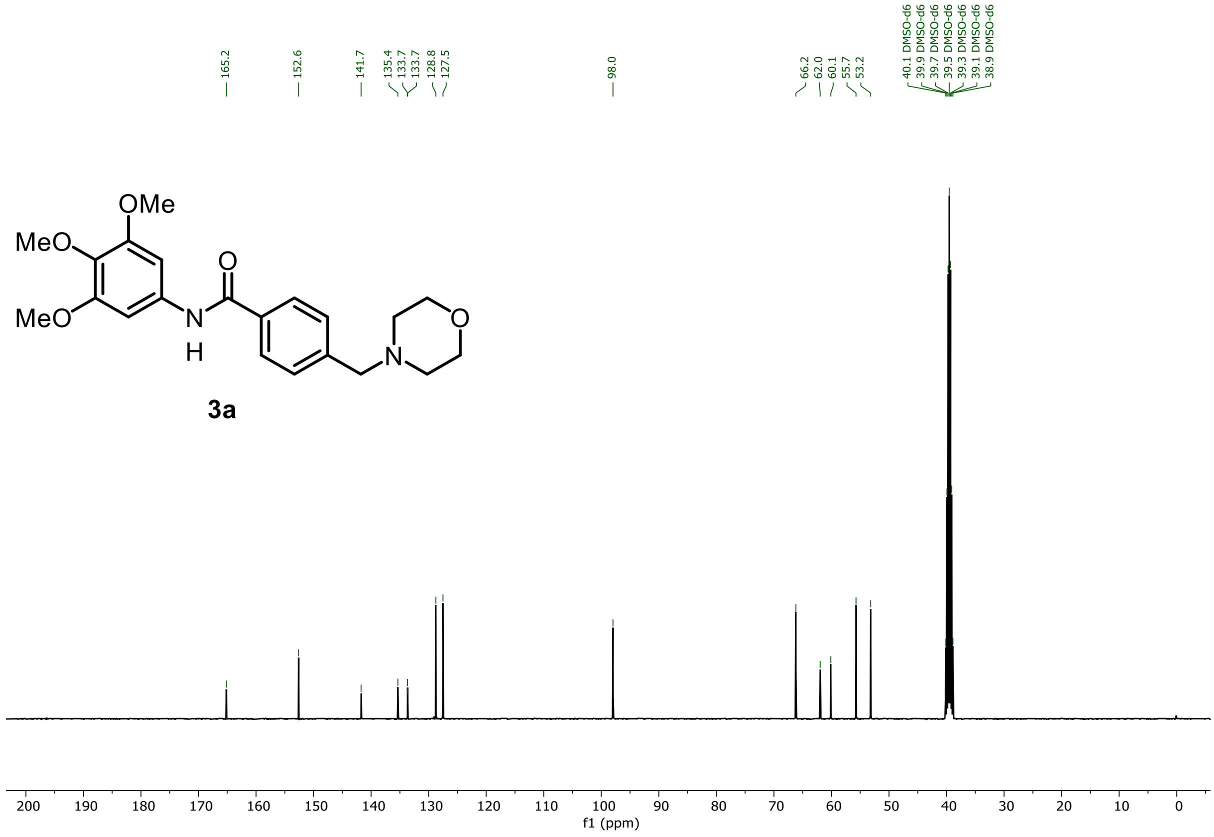

^{13}CNMR spectrum of **3a** run in DMSO-d_6 at 101 MHz.

N-(3,4,5-Trimethoxyphenyl)cyclopropanecarboxamide (4a)

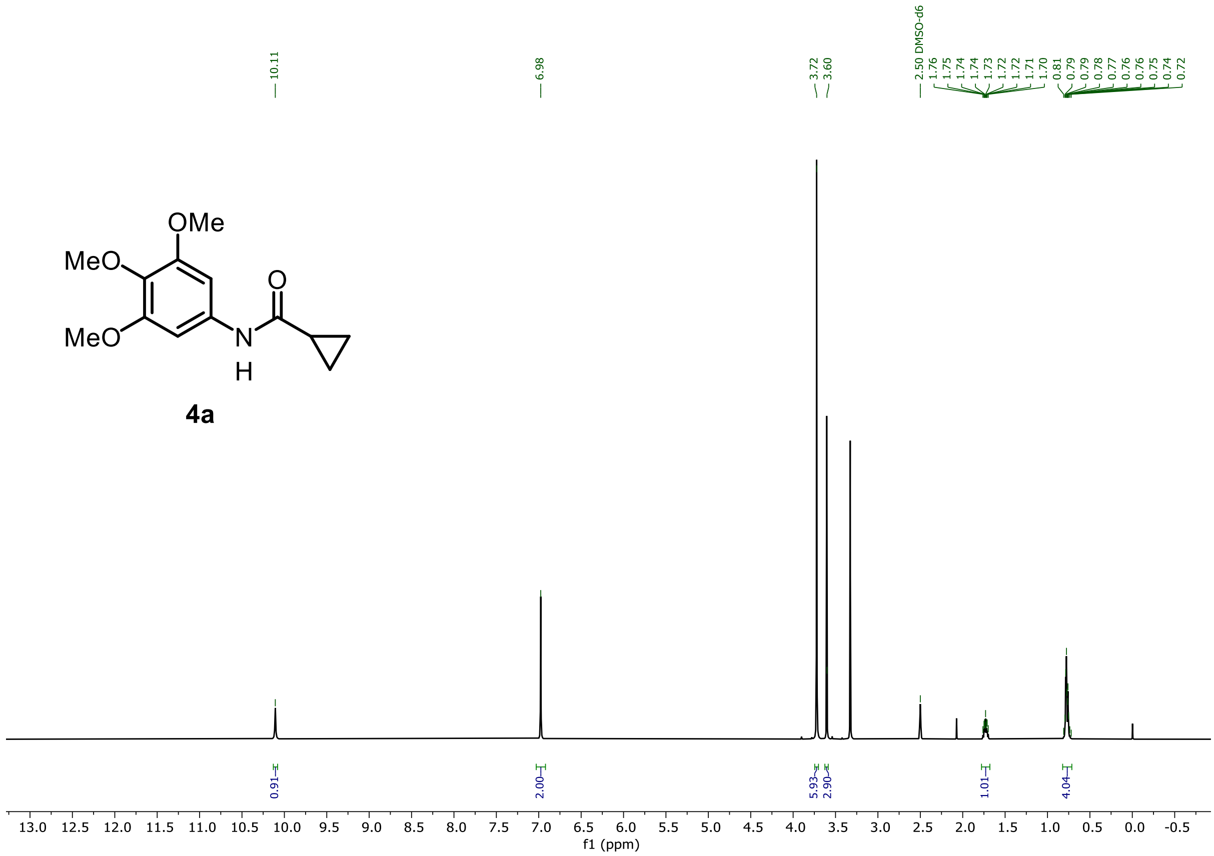

^{1}HNMR spectrum of **4a** run in DMSO-d_6 at 400 MHz.

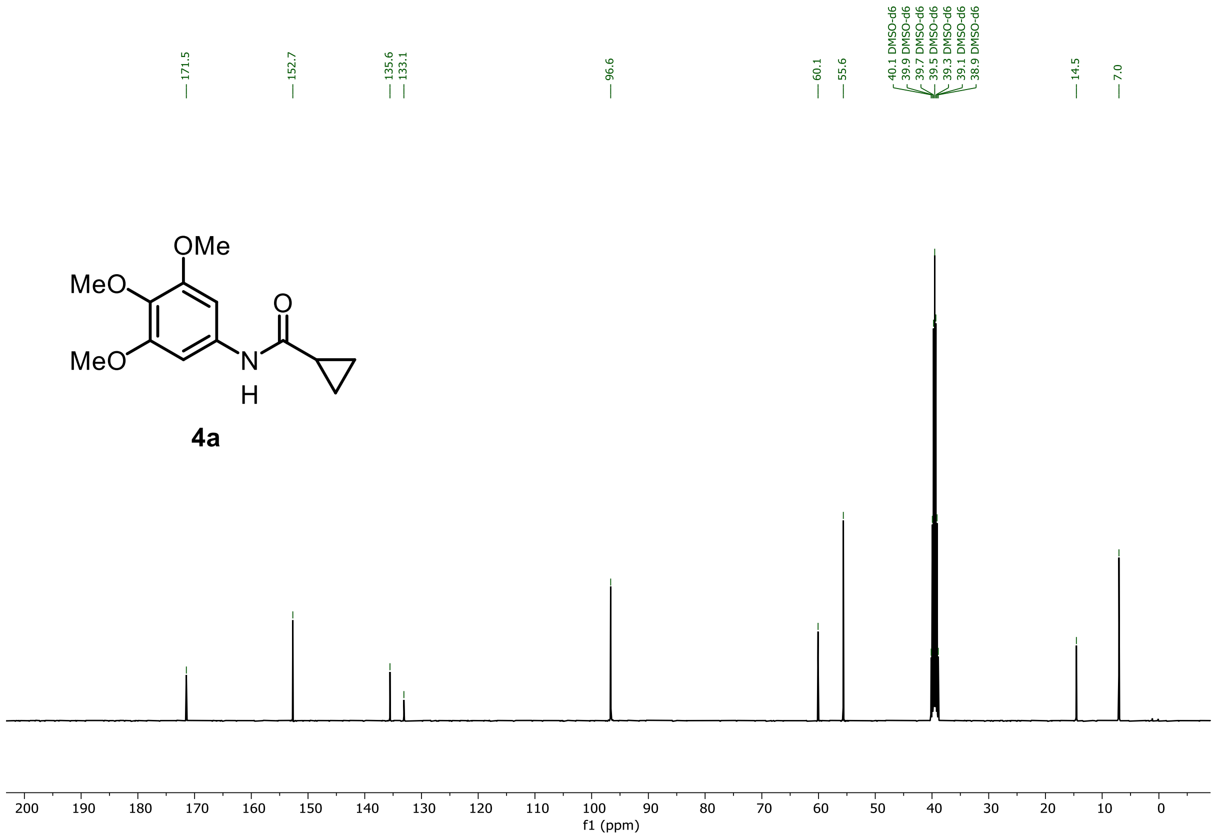

^{13}CNMR spectrum of **4a** run in DMSO-d_6 at 101 MHz.

3-Hydroxy-*N*-(4-methoxybenzyl)azetidine-1-carboxamide (5a)

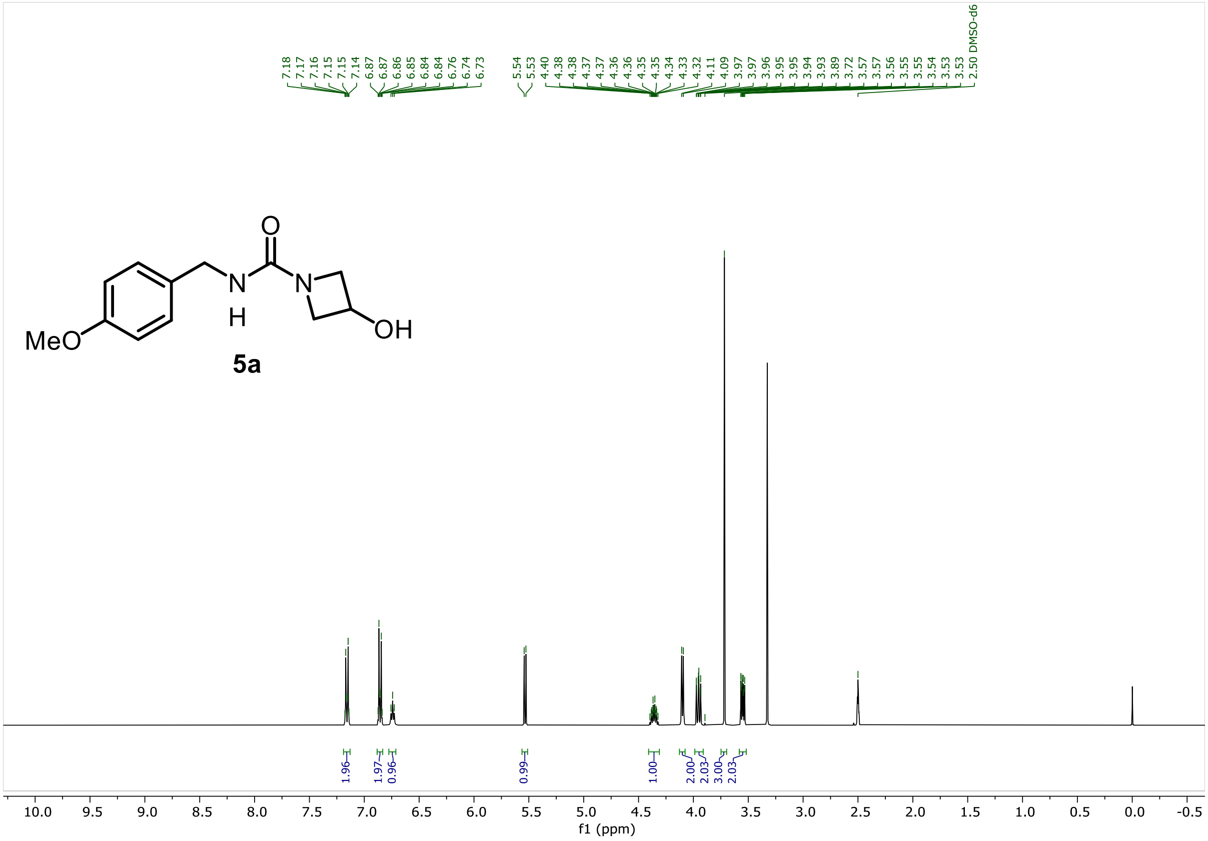

^{1}HNMR spectrum of **5a** run in DMSO-d_6 at 400 MHz.

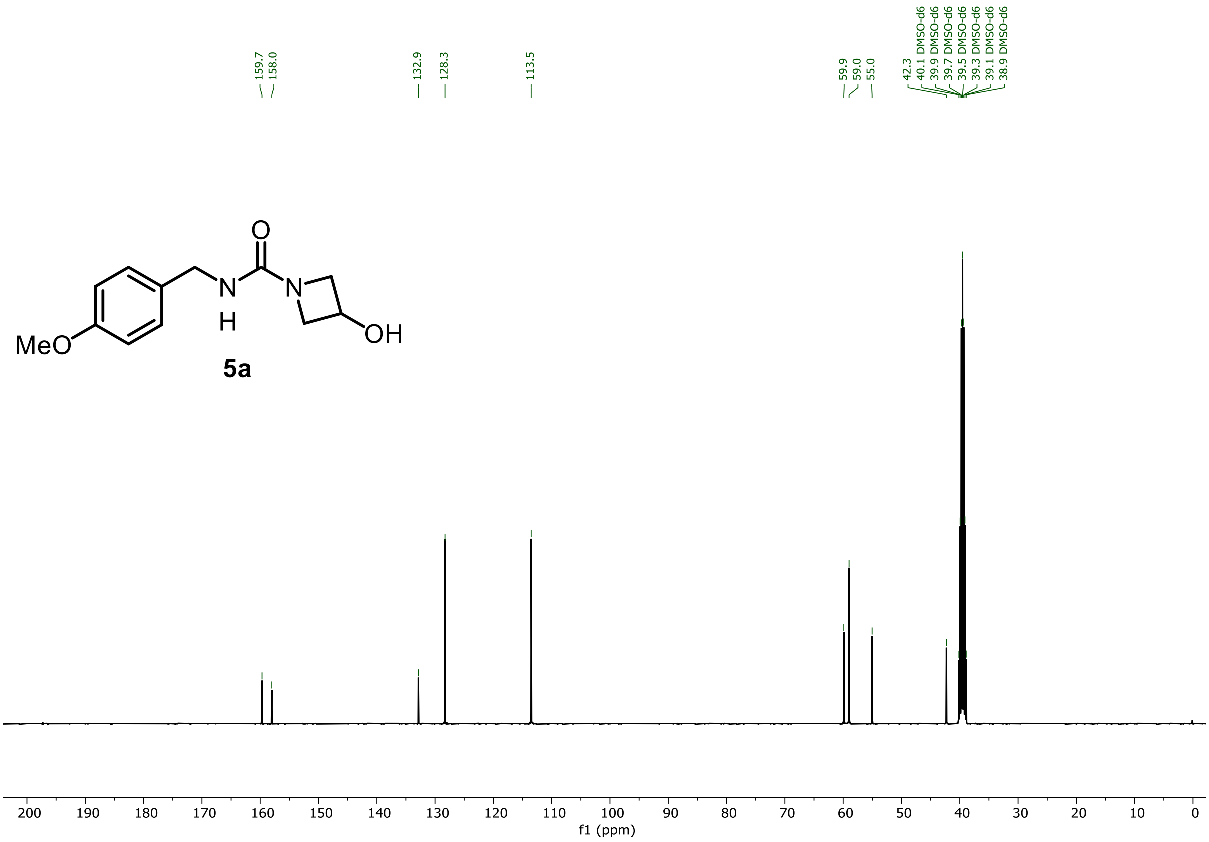

^{13}CNMR spectrum of **5a** run in DMSO-d_6 at 101 MHz.

3-Hydroxy-*N*-(4-methoxybenzyl)piperidine-1-carboxamide (6a)

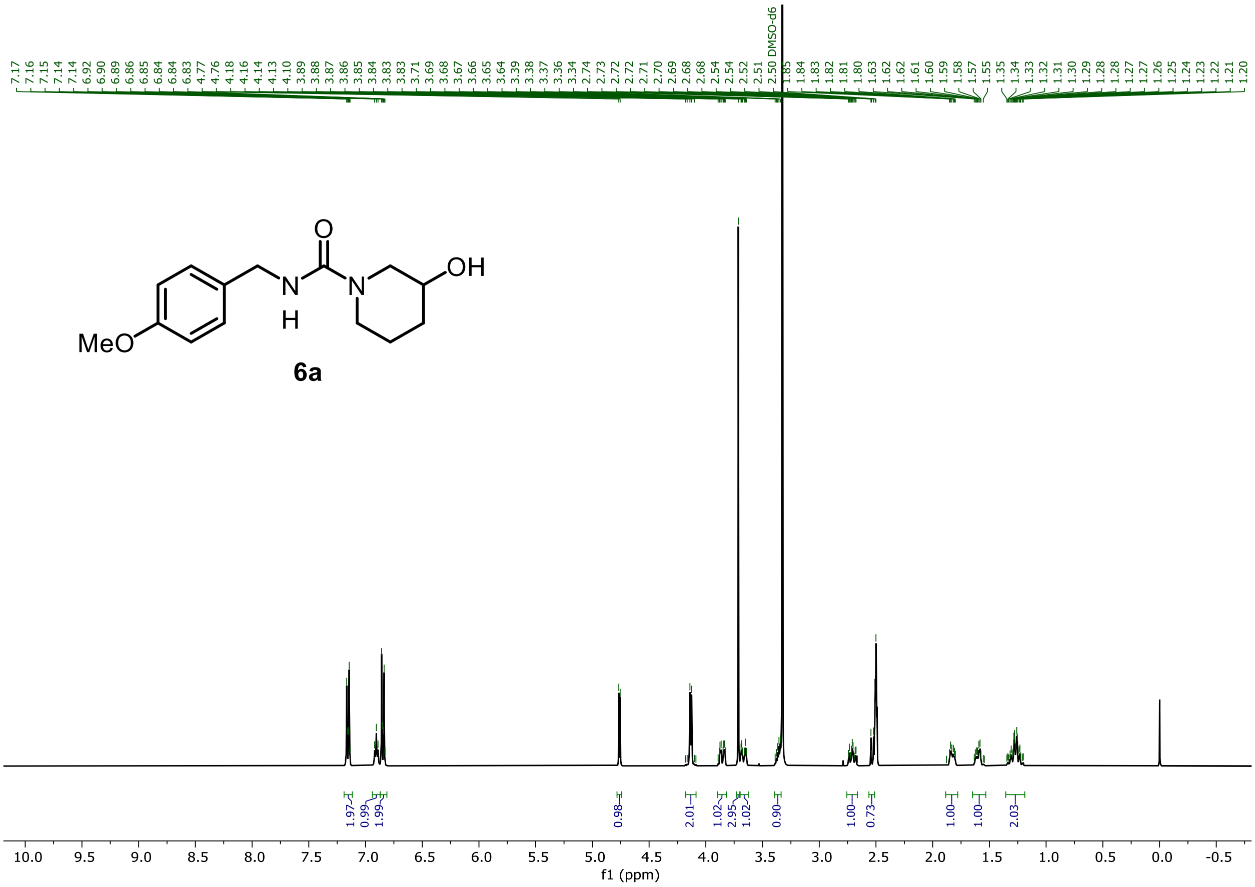

^{1}HNMR spectrum of **6a** run in DMSO-d_6 at 400 MHz.

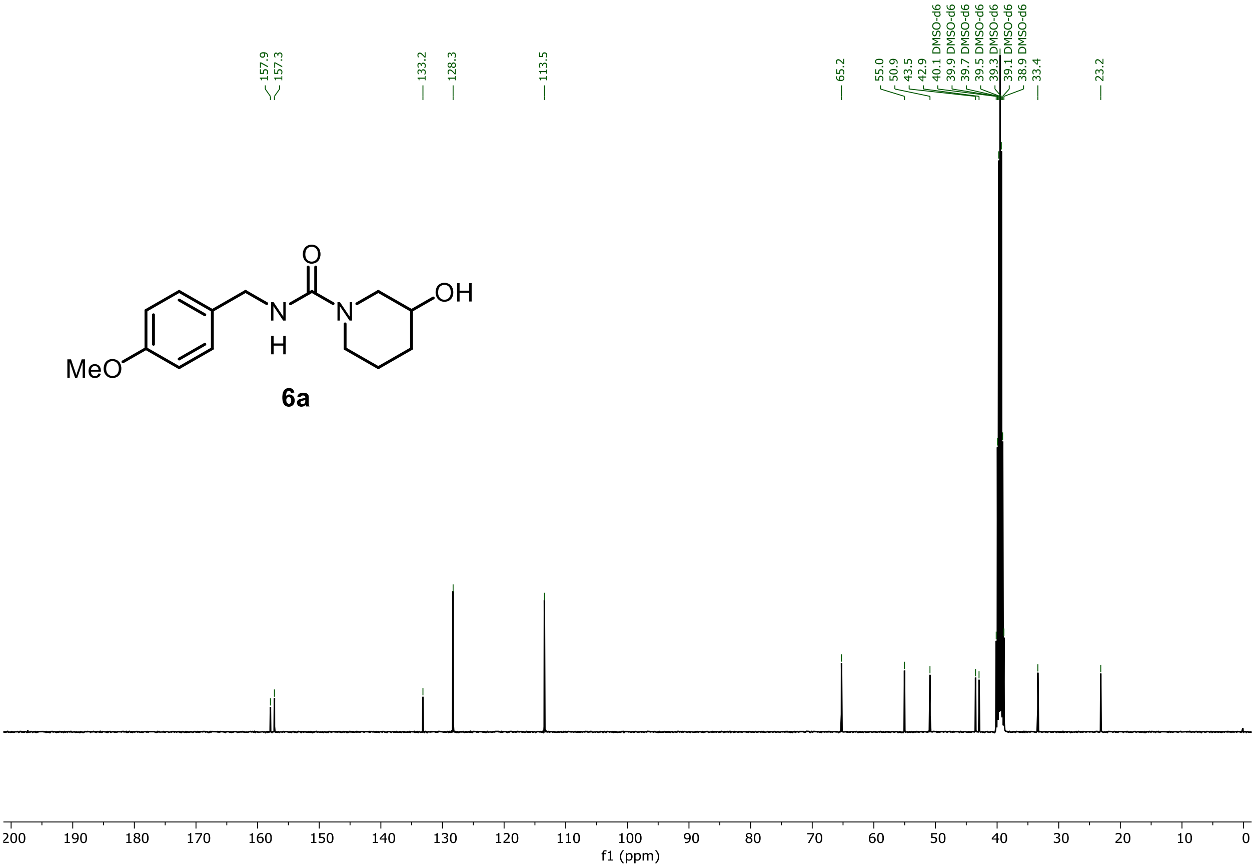

^{13}CNMR spectrum of **6a** run in DMSO-d_6 at 101 MHz.

N-(4-Methoxybenzyl)-4-methyl-3-oxopiperazine-1-carboxamide (7a)

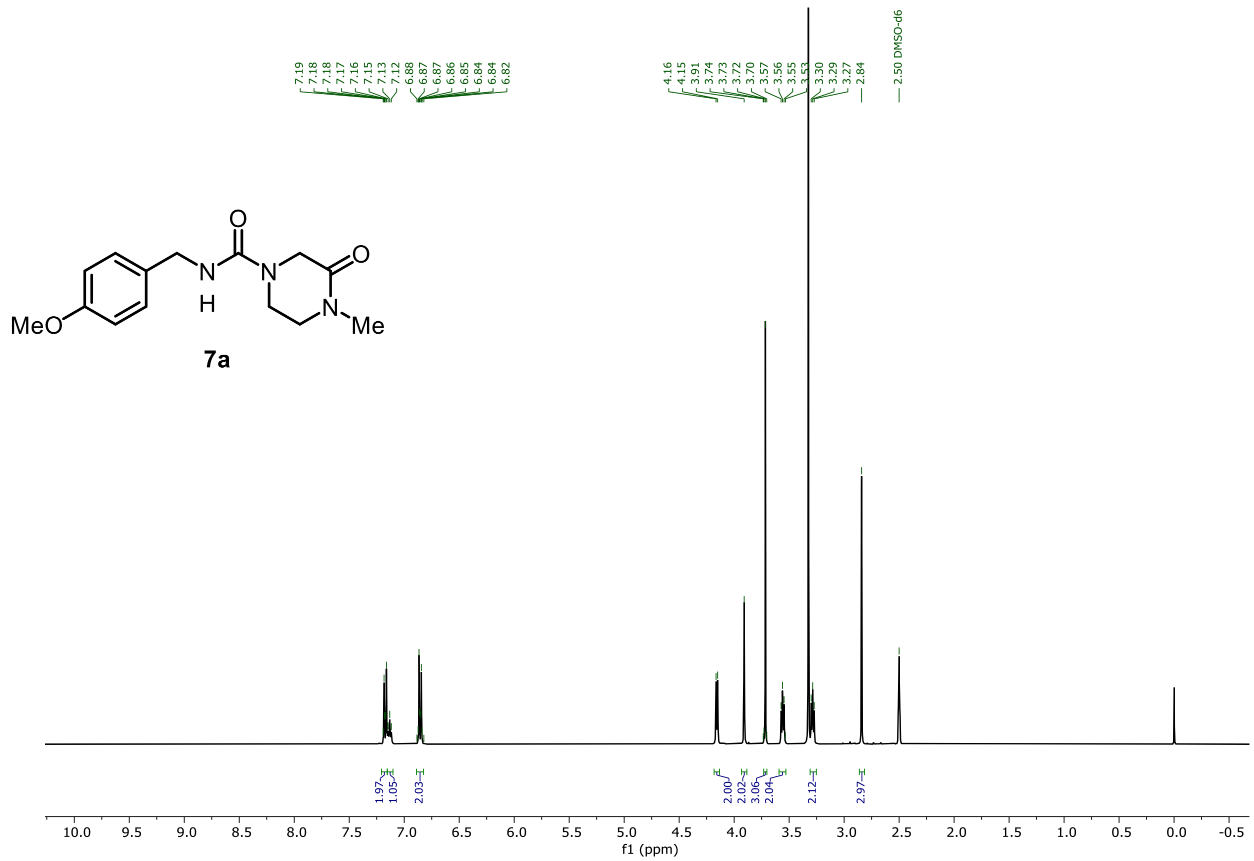

^{1}HNMR spectrum of **7a** run in DMSO-d_6 at 400 MHz.

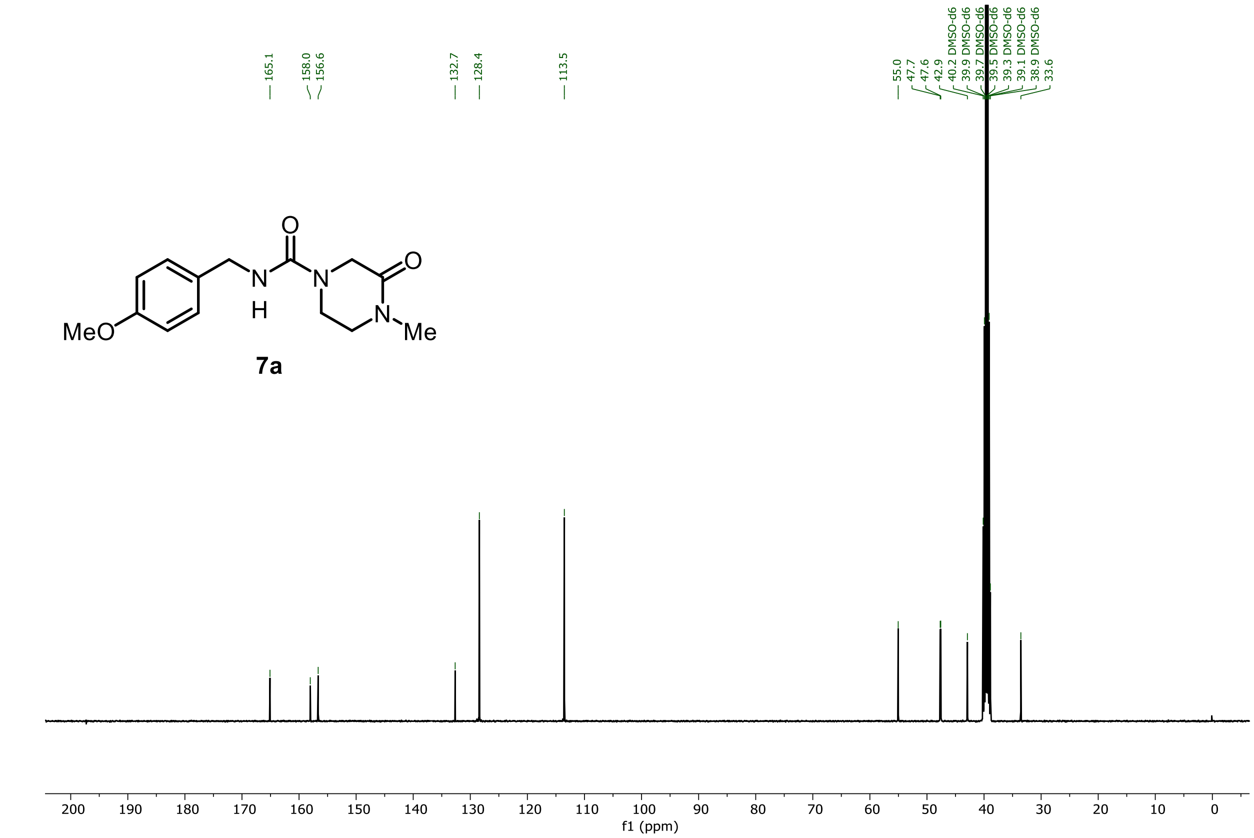

^{13}CNMR spectrum of **7a** run in DMSO-d_6 at 101 MHz.

1-(Cyanomethyl)-3-(4-methoxybenzyl)urea (8a)

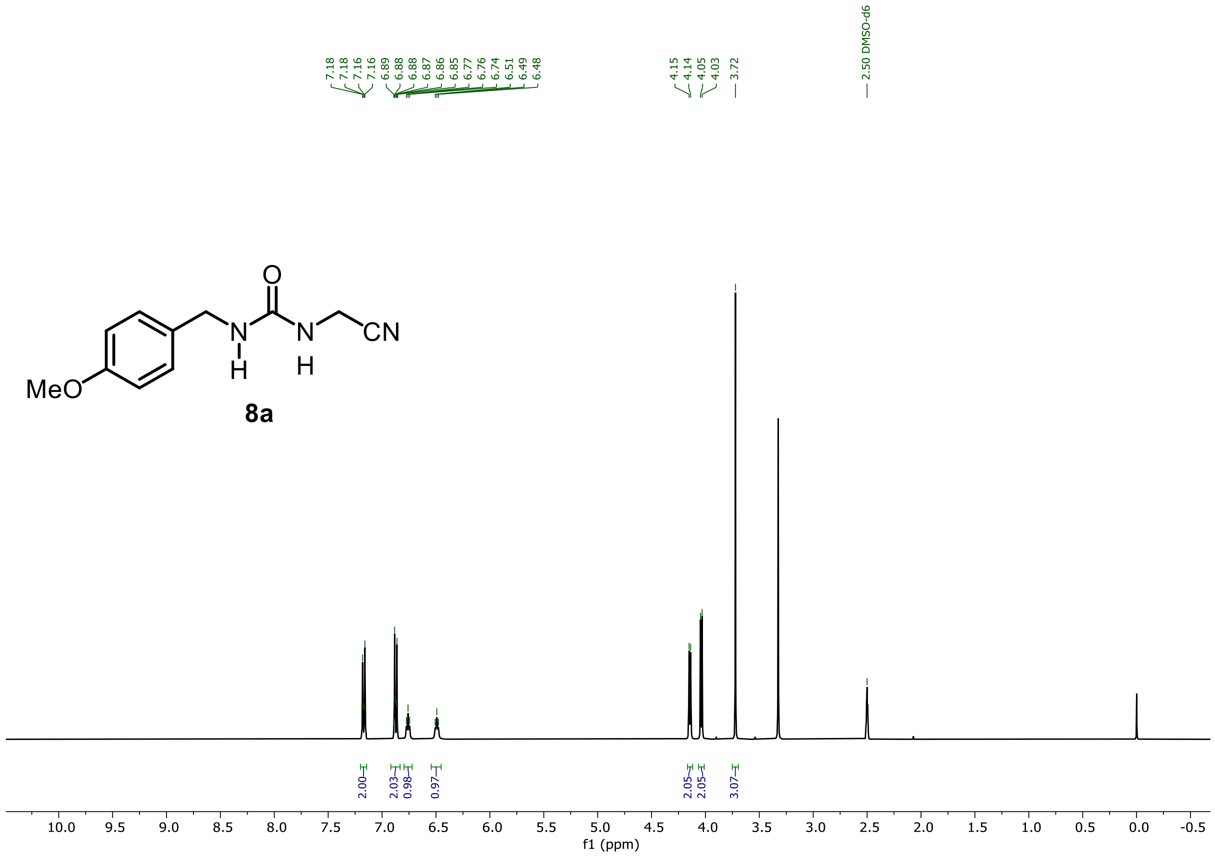

^{1}HNMR spectrum of **8a** run in DMSO-d_6 at 400 MHz.

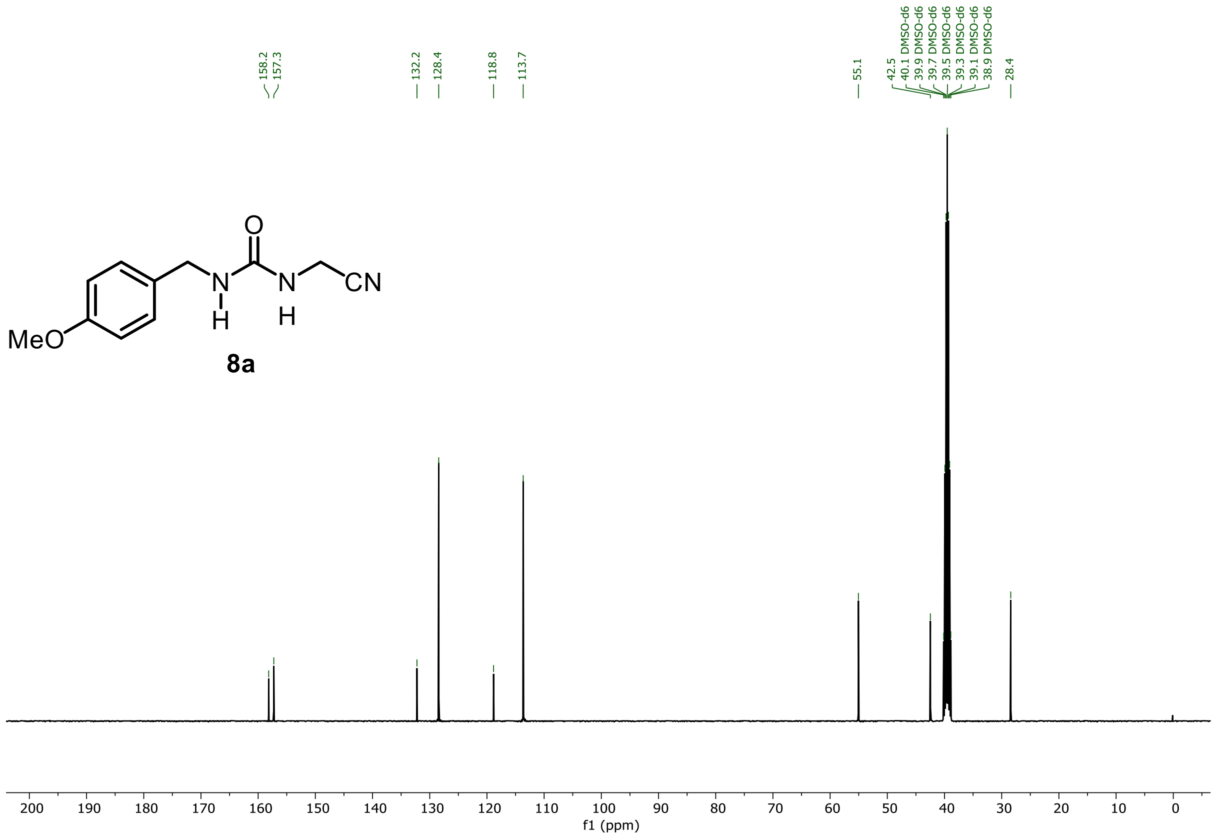

^{13}CNMR spectrum of **8a** run in DMSO-d_6 at 101 MHz.

3.7 Miscellaneous

(*S*)-*N*-((2-Oxo-3-(4-(3-oxomorpholino)phenyl)oxazolidin-5-yl)methyl)acetamide (*S*10)

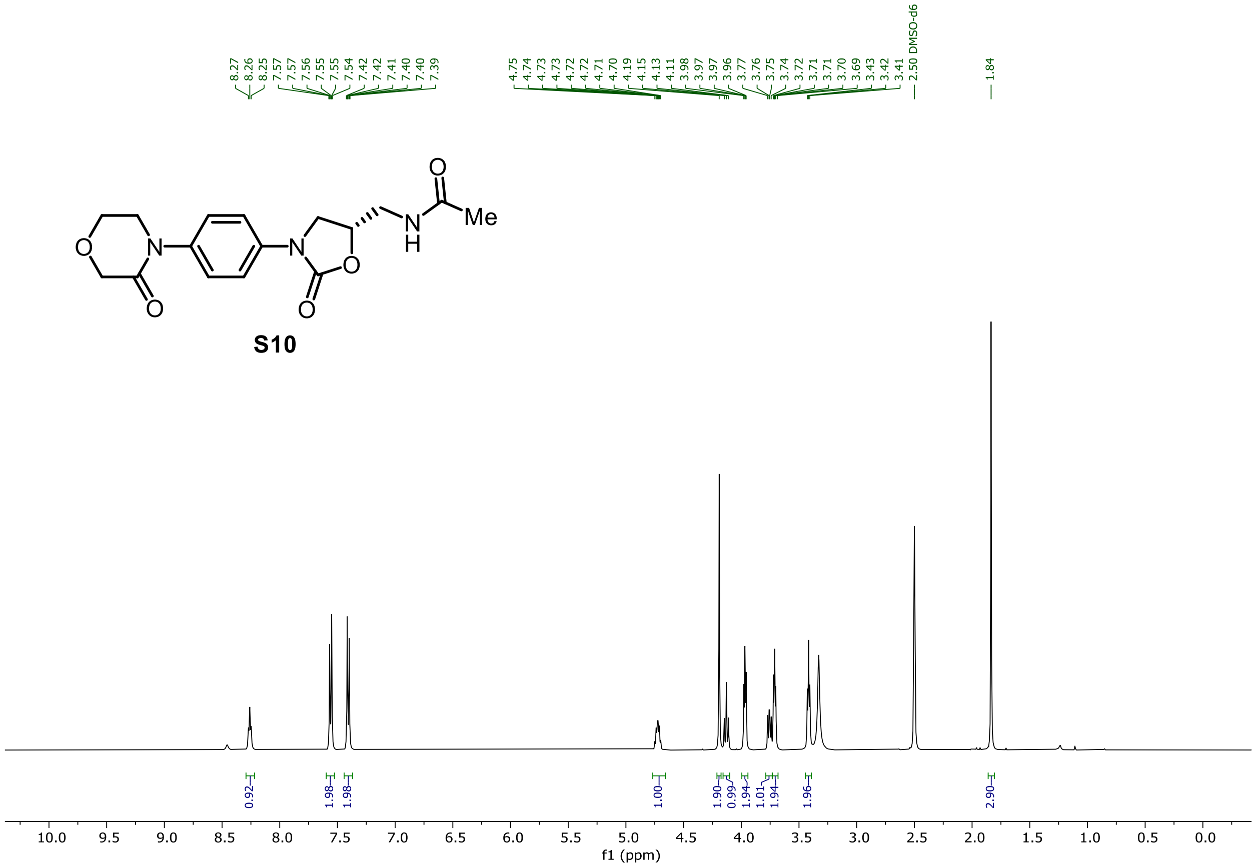

^{1}HNMR spectrum of **S10** run in DMSO-d_6 at 500 MHz.

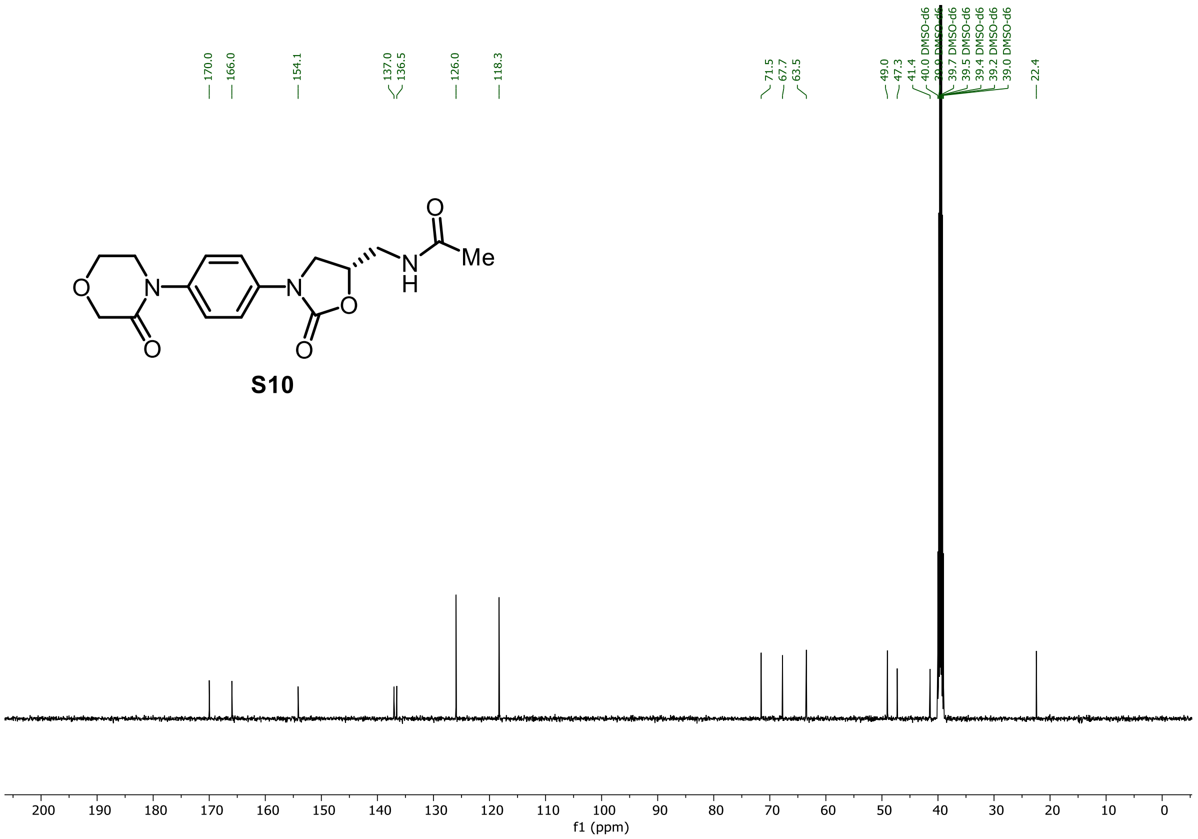

^{13}CNMR spectrum of **S10** run in DMSO-d_6 at 126 MHz.

(*S*)-*N*-((2-Oxo-3-(4-(3-oxomorpholino)phenyl)oxazolidin-5-yl)methyl)cyclopropanecarbox-amide (S11)

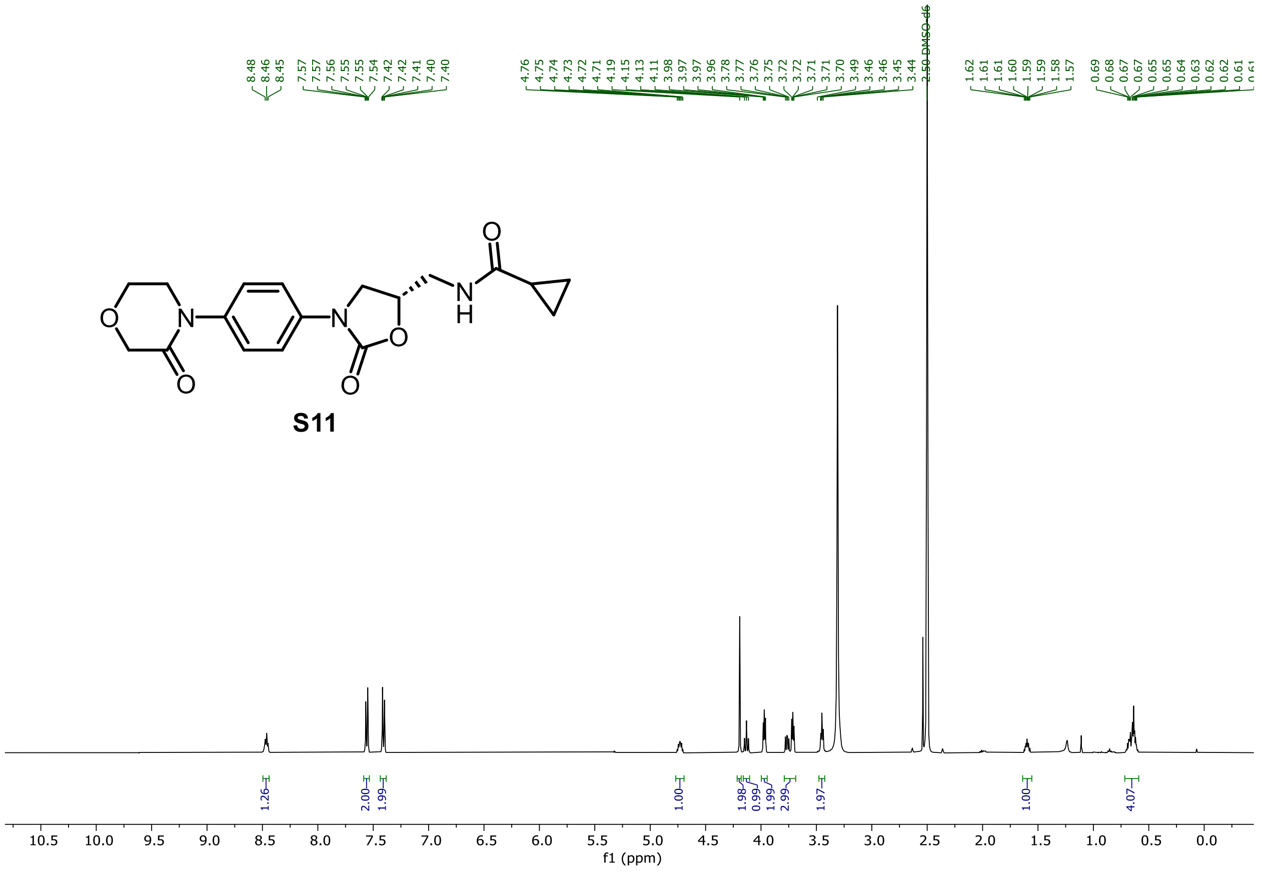

^{1}HNMR spectrum of **S11** run in DMSO-d_6 at 500 MHz.

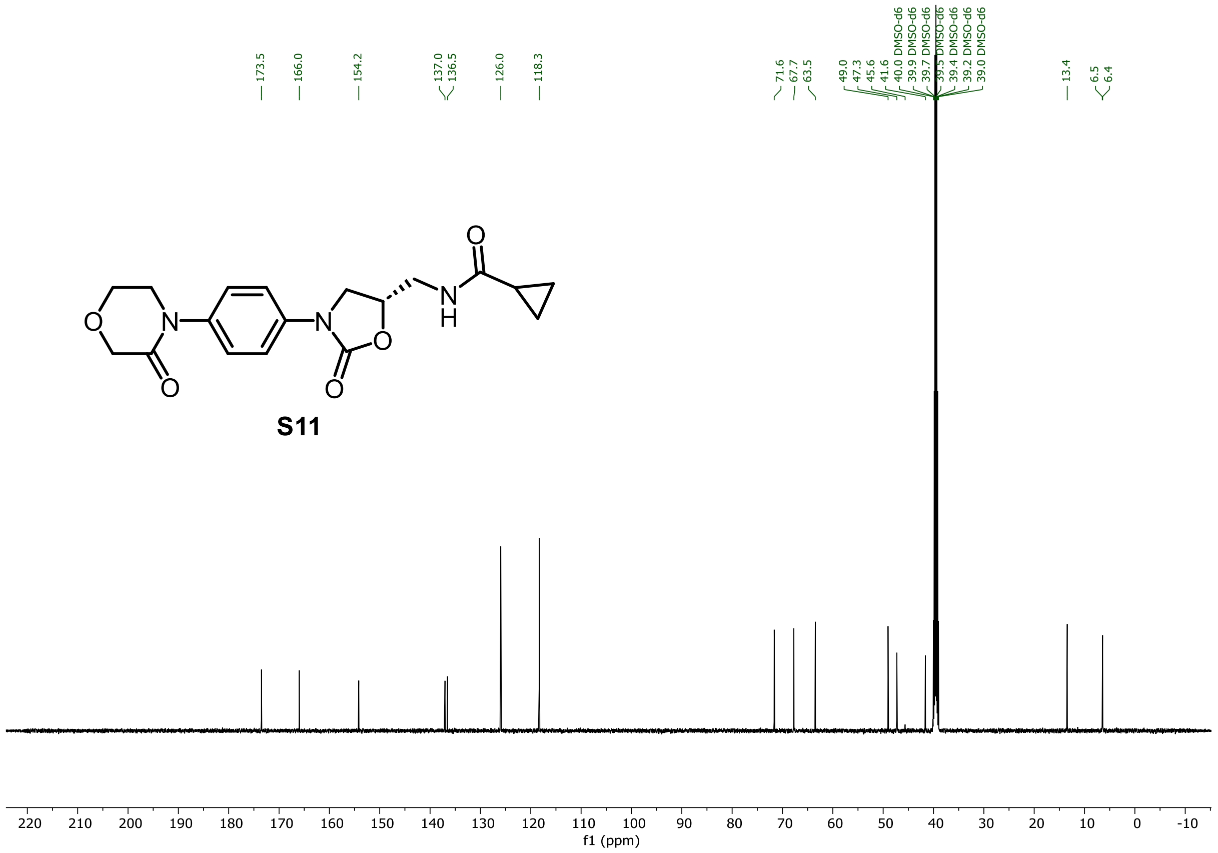

^{13}CNMR spectrum of **S11** run in DMSO-d_6 at 126 MHz.

(*S*)-*N*-((2-Oxo-3-(4-(3-oxomorpholino)phenyl)oxazolidin-5-yl)methyl)-2-phenylacetamide (S12)

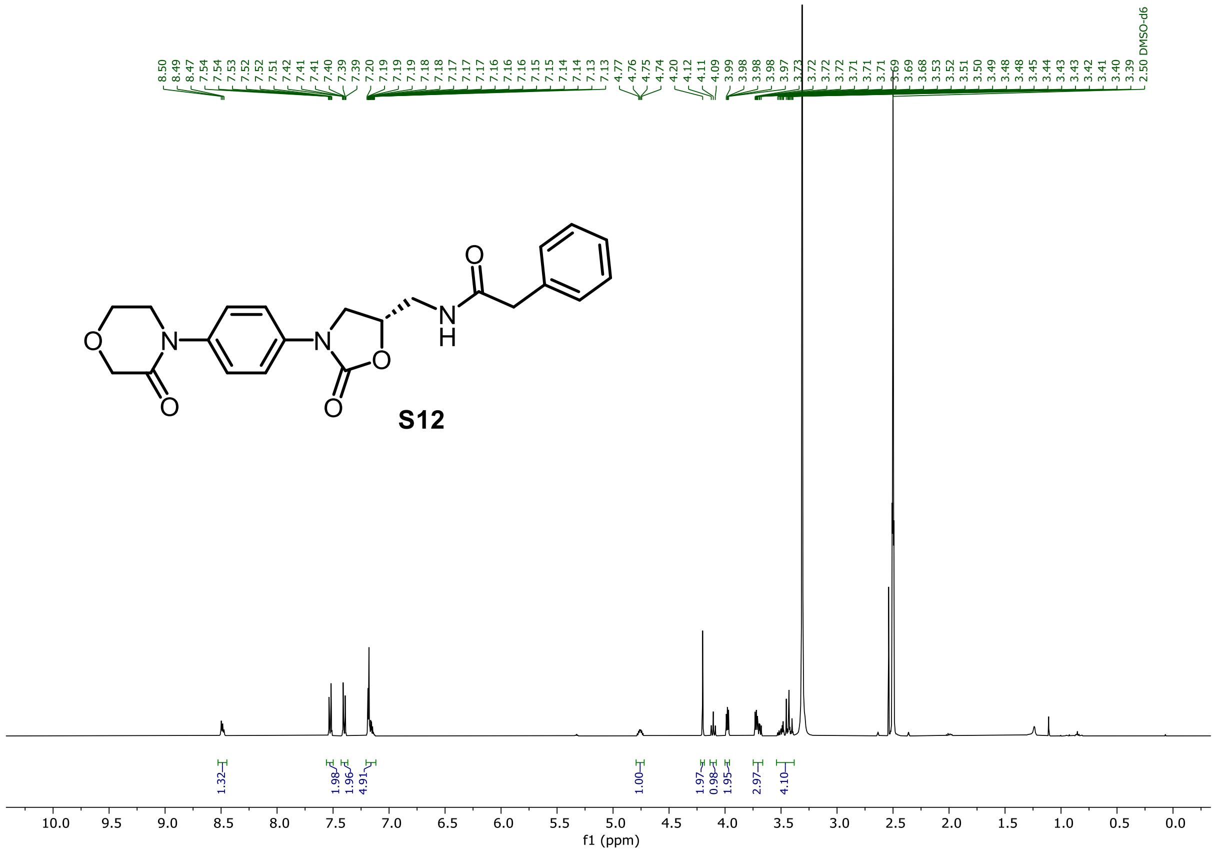

^{1}HNMR spectrum of **S12** run in DMSO-d_6 at 500 MHz.

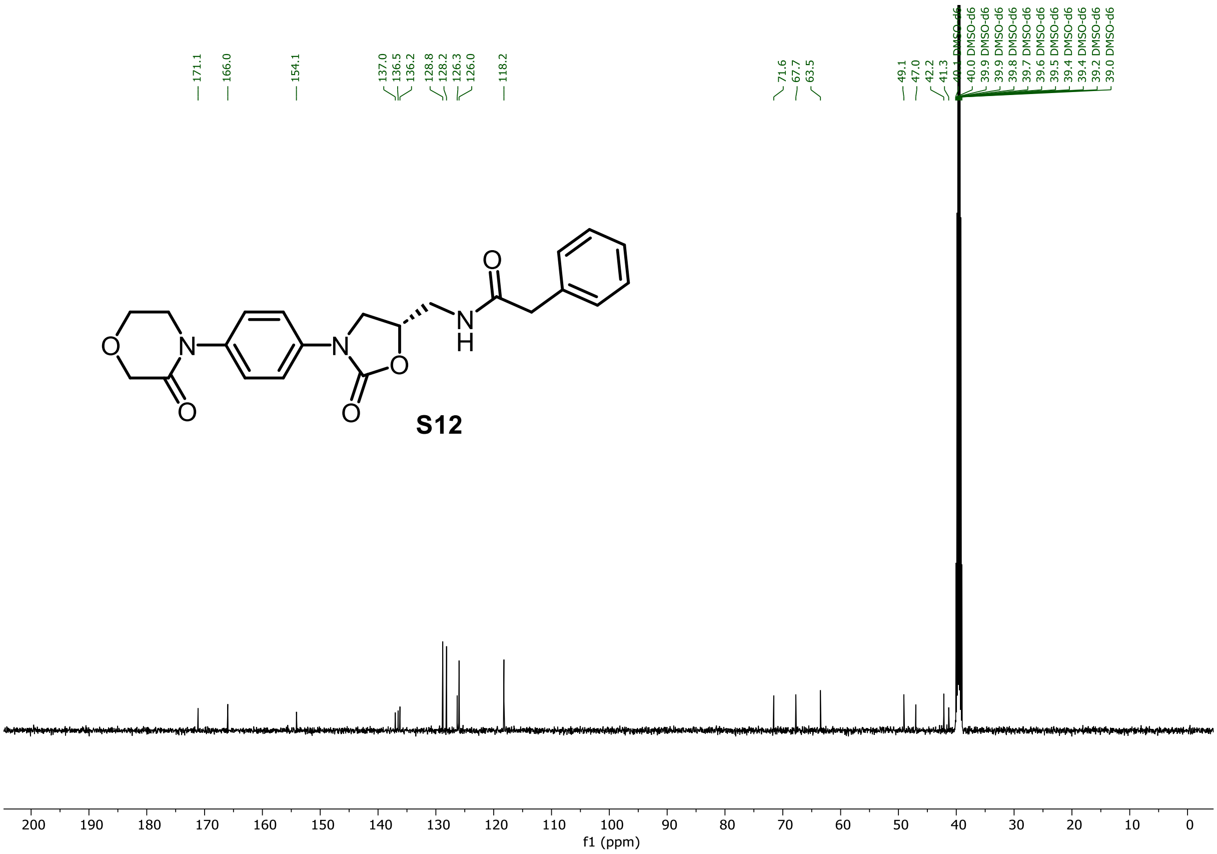

^{13}CNMR spectrum of **S12** run in DMSO-d_6 at 126 MHz.

(*S*)-*N*-((2-Oxo-3-(4-(3-oxomorpholino)phenyl)oxazolidin-5-yl)methyl)benzamide (S13)

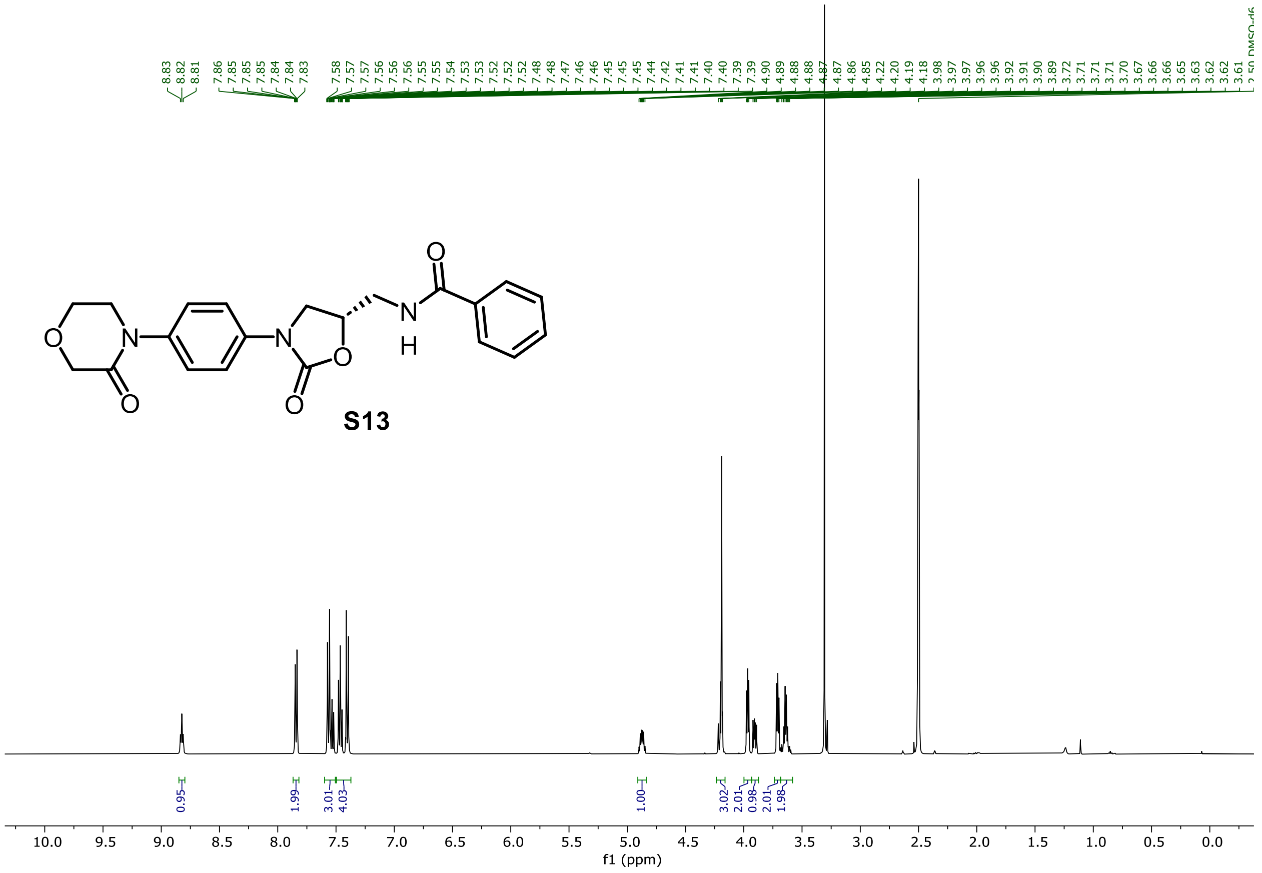

^{1}HNMR spectrum of **S13** run in DMSO-d_6 at 500 MHz.

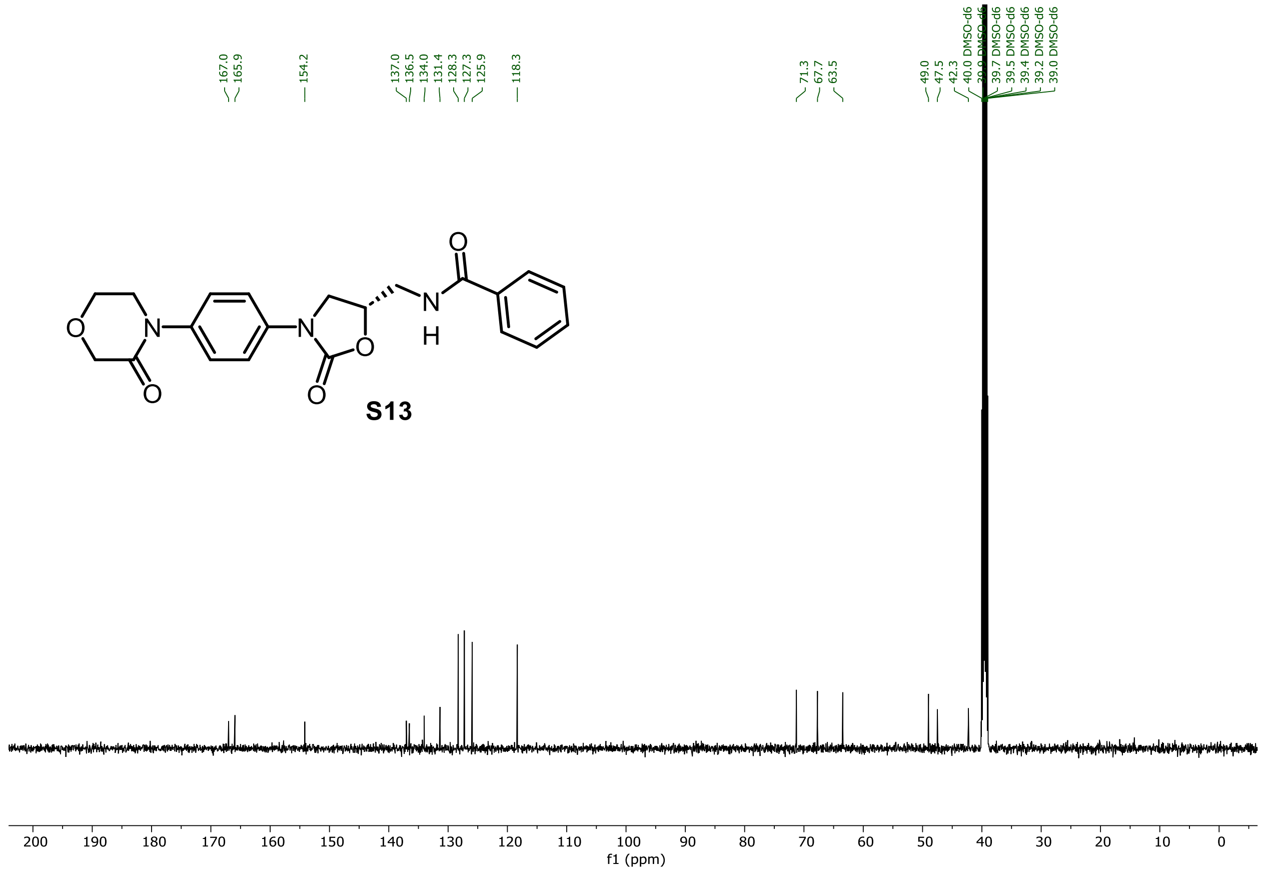

^{13}CNMR spectrum of **S13** run in DMSO-d_6 at 126 MHz.

(*S*)-4-(4-(5-((Isopropylamino)methyl)-2-oxooxazolidin-3-yl)phenyl)morpholin-3-one (S16)

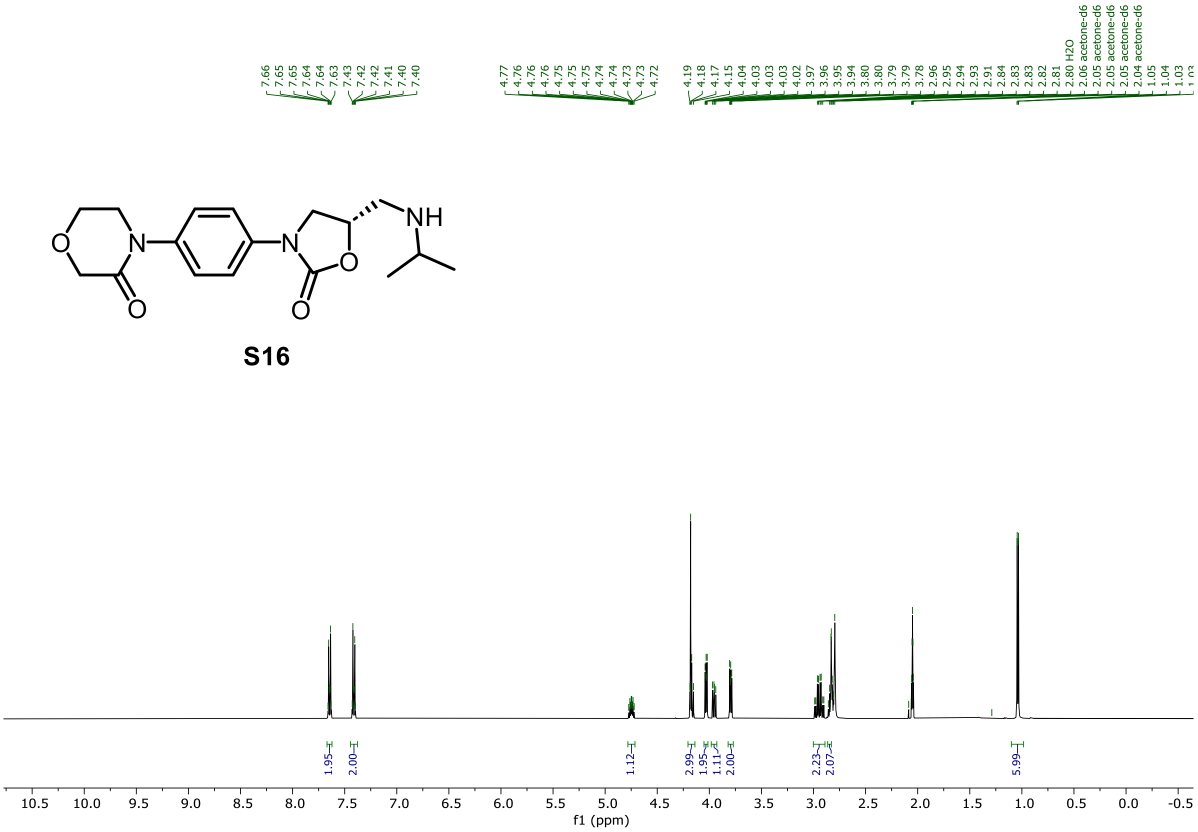

^{1}HNMR spectrum of **S16** run in acetone-d_6 at 500 MHz.

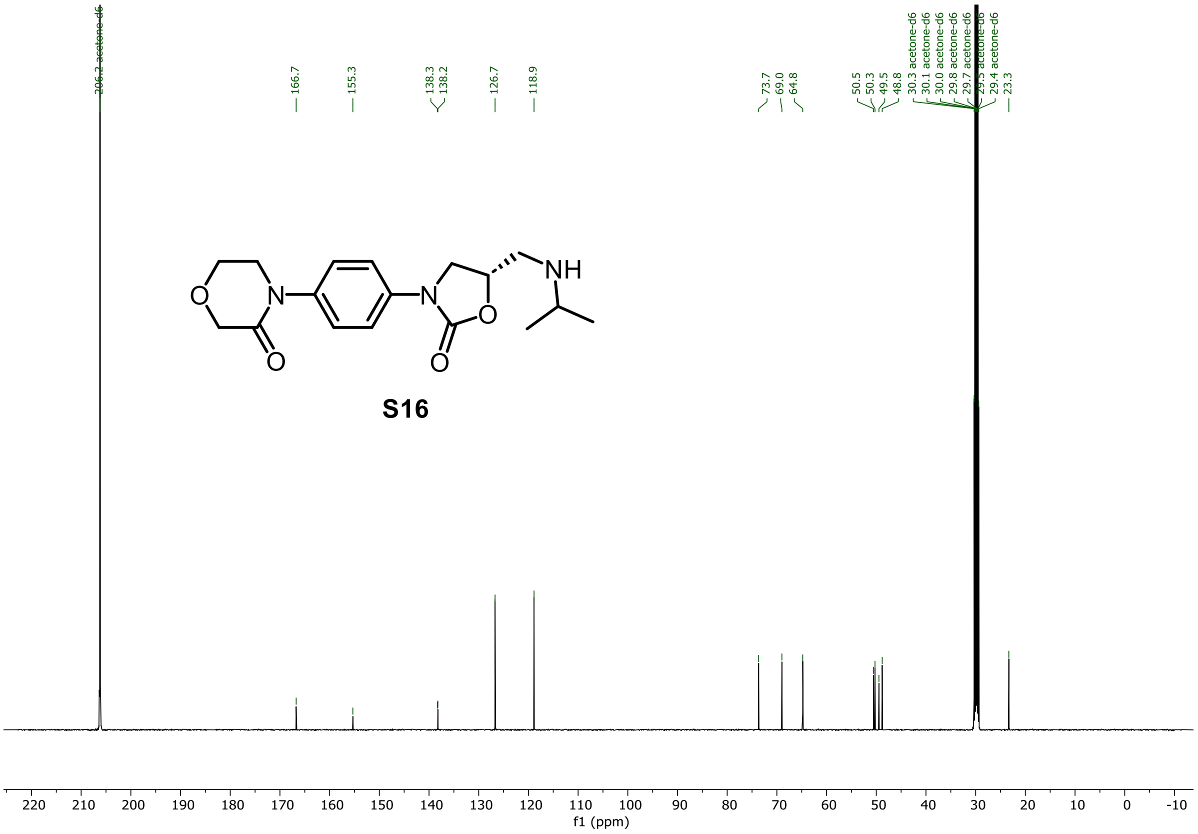

^{13}CNMR spectrum of **S16** run in acetone-d_6 at 126 MHz.

4. Purity analysis

(*R*)-*N*-((2-Oxo-3-(4-(3-oxomorpholino)phenyl)oxazolidin-5-yl)methyl)-*N*-trifluoromethyl acetamide (10a)

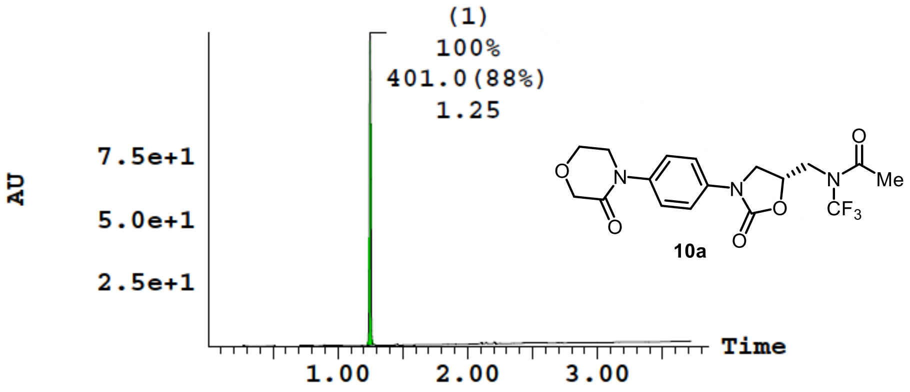

(*R*)-*N*-((2-Oxo-3-(4-(3-oxomorpholino)phenyl)oxazolidin-5-yl)methyl)-*N*-(trifluoromethyl) cyclopropanecarboxamide (10b)

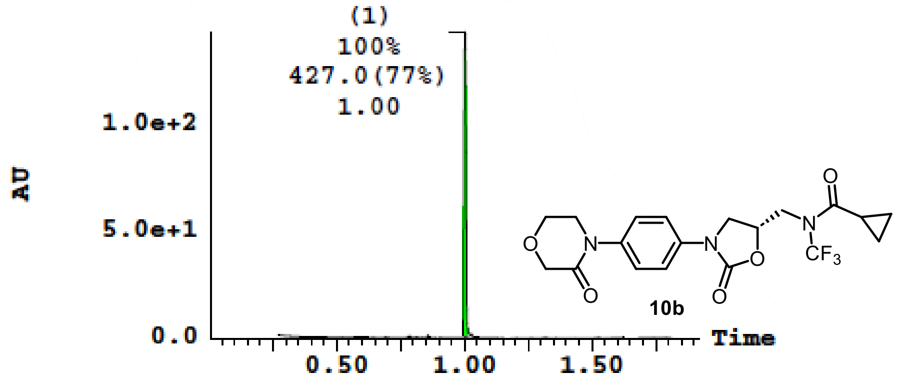

(*R*)-*N*-((2-Oxo-3-(4-(3-oxomorpholino)phenyl)oxazolidin-5-yl)methyl)-2-phenyl-*N*-(trifluoromethyl)acetamide (10c)

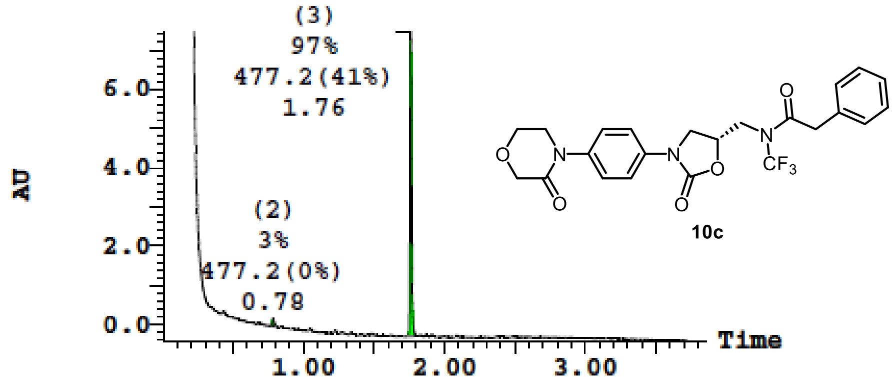

(*R*)-*N*-((2-Oxo-3-(4-(3-oxomorpholino)phenyl)oxazolidin-5-yl)methyl)-*N*-trifluoromethyl benzamide (10d)

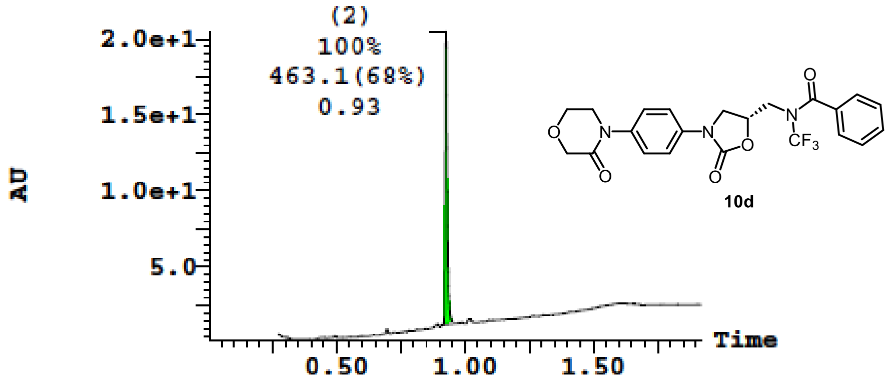

(*S*)-*N*-Methyl-*N*-((2-oxo-3-(4-(3-oxomorpholino)phenyl)oxazolidin-5-yl)methyl)acetamide (10e)

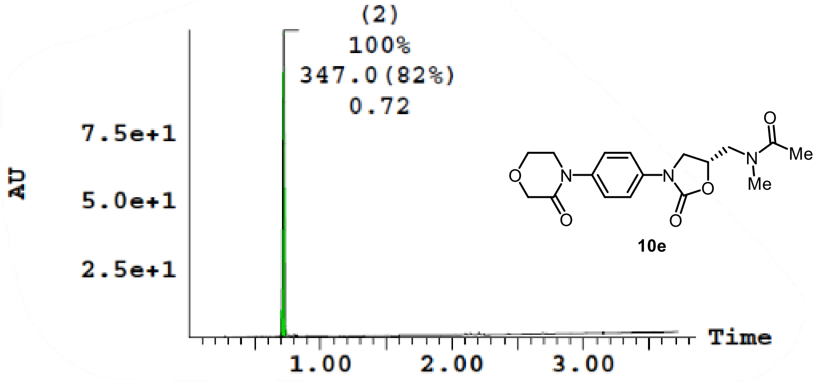

(*S*)-*N*-Isopropyl-*N*-((2-oxo-3-(4-(3-oxomorpholino)phenyl)oxazolidin-5-yl)methyl)acetamide (10f)

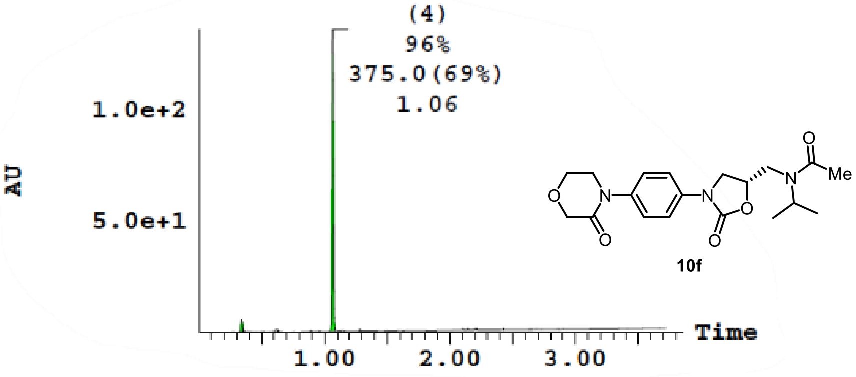

(*S*)-*N*-Methyl-*N*-((2-oxo-3-(4-(3-oxomorpholino)phenyl)oxazolidin-5-yl) methyl) cyclopropanecarboxamide (10g)

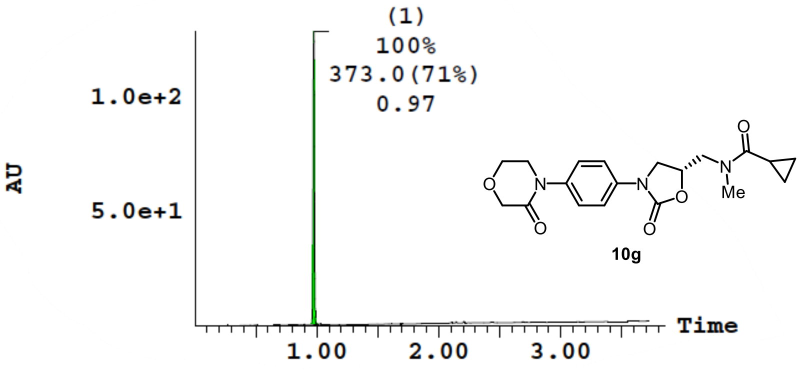

(*S*)-*N*-Isopropyl-*N*-((2-oxo-3-(4-(3-oxomorpholino)phenyl)oxazolidin-5-yl) methyl) cyclopropane carboxamide (10h)

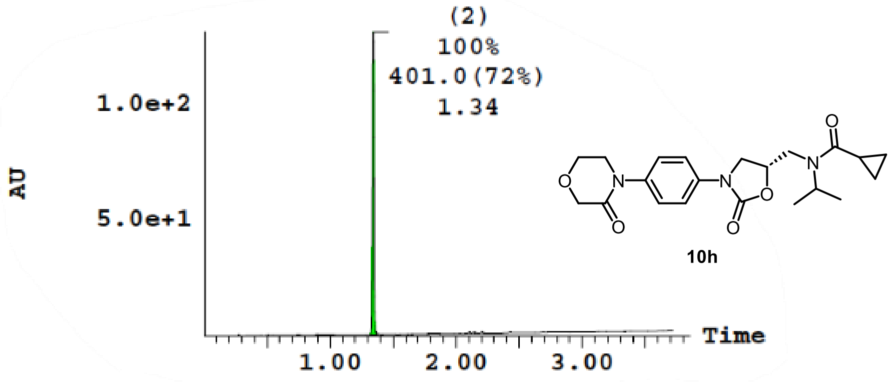

***N*-(4-Hydroxyphenyl)-*N*-(trifluoromethyl)acetamide (11a)**

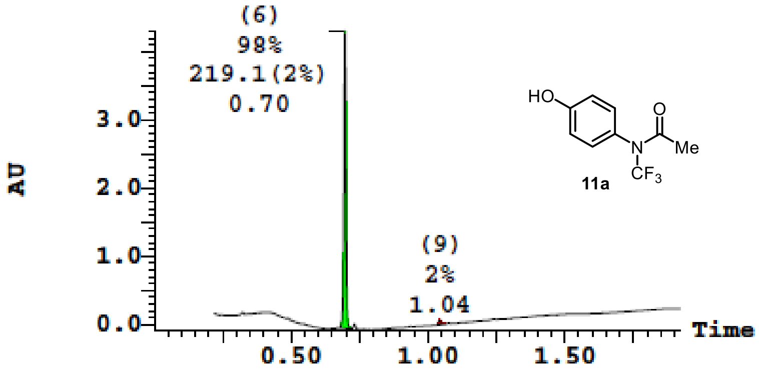

N-(4-Hydroxyphenyl)-N-(trifluoromethyl)cyclopropanecarboxamide (11b)

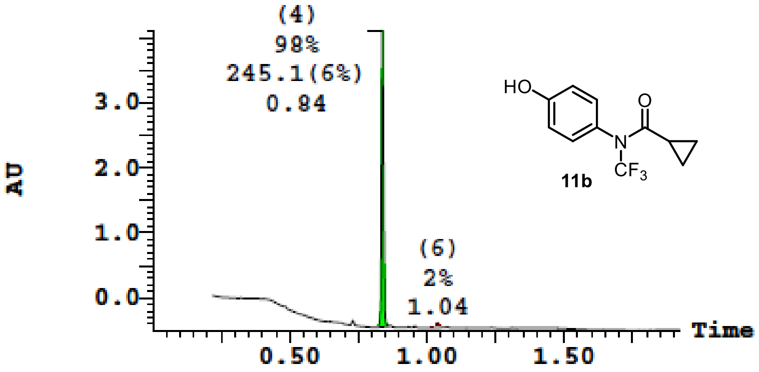

N-(4-Hydroxyphenyl)-2-phenyl-N-(trifluoromethyl)acetamide (11c)

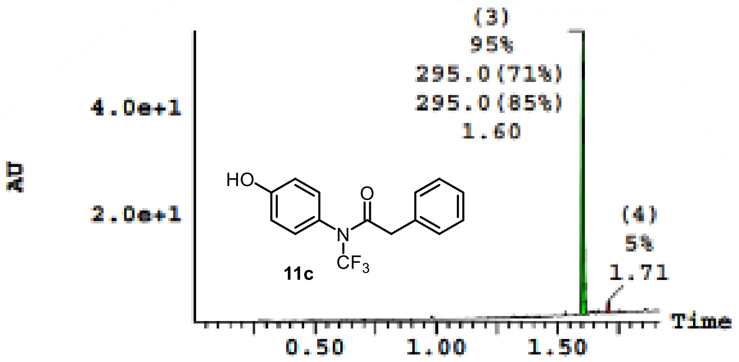

N-(4-Hydroxyphenyl)-N-(trifluoromethyl)benzamide (11d)

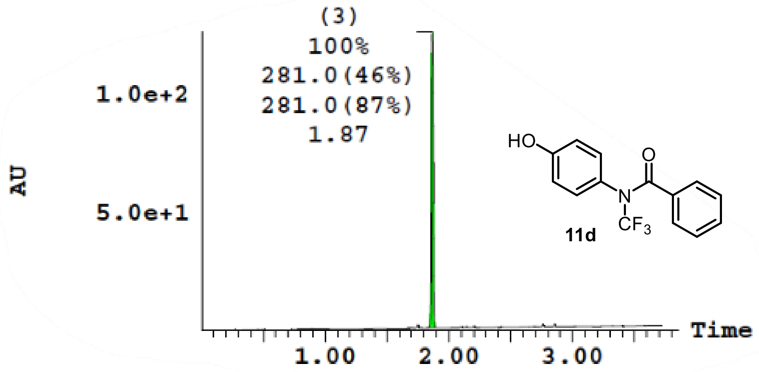

2-(2-Methyl-5-nitro-1*H*-imidazol-1-yl)ethyl methyl(trifluoromethyl)carbamate (12a)

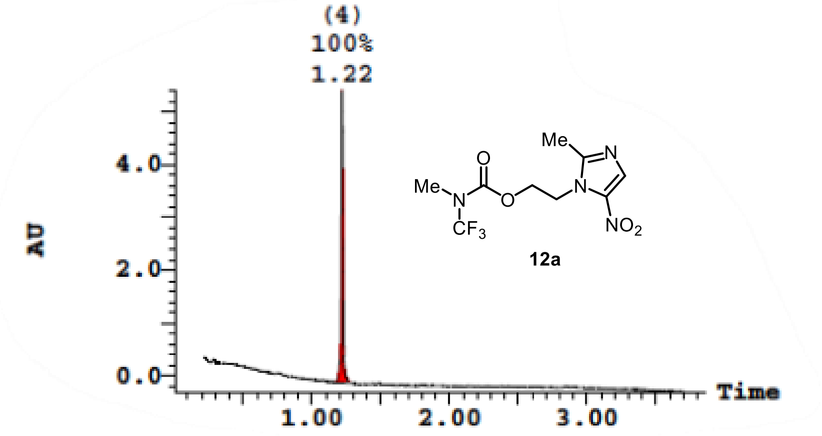

2-(2-Methyl-5-nitro-1*H*-imidazol-1-yl)ethyl cyclopropyl(trifluoromethyl)carbamate (12b)

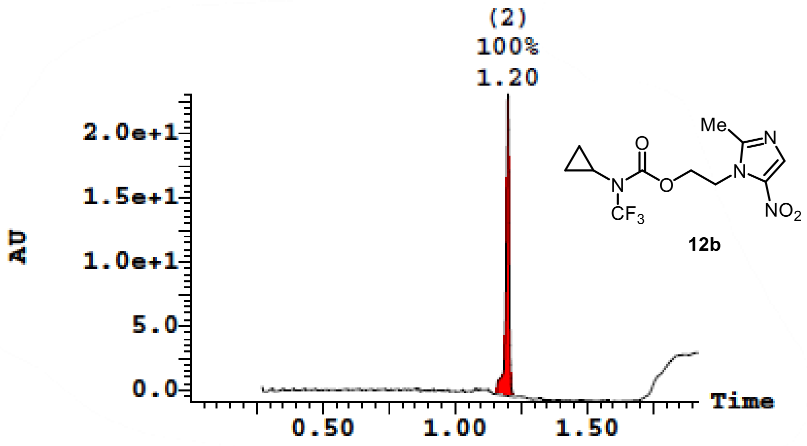

2-(2-Methyl-5-nitro-1*H*-imidazol-1-yl)ethyl benzyl(trifluoromethyl)carbamate (12c)

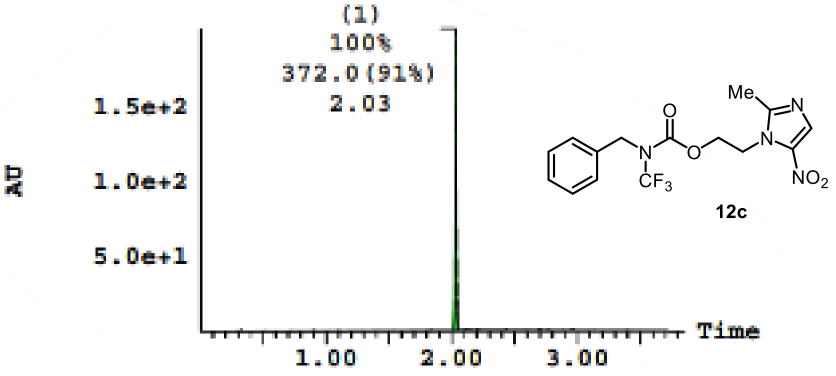

2-(2-Methyl-5-nitro-1*H*-imidazol-1-yl)ethyl phenyl(trifluoromethyl)carbamate (12d)

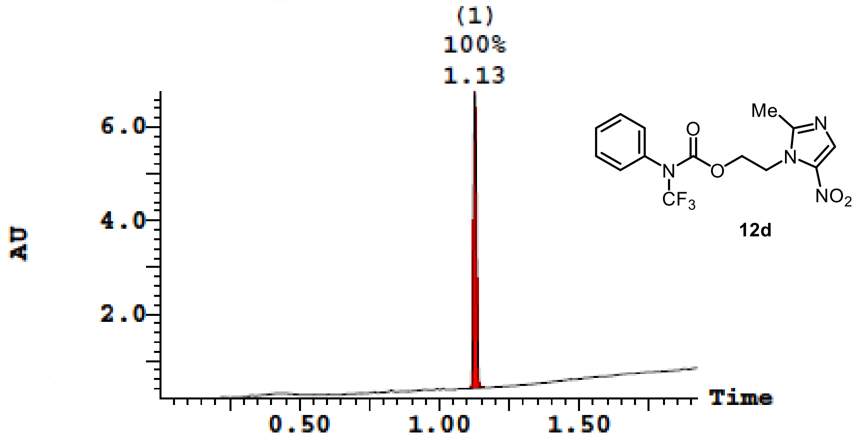

2-(2-Methyl-5-nitro-1*H*-imidazol-1-yl)ethyl dimethylcarbamate (12e)

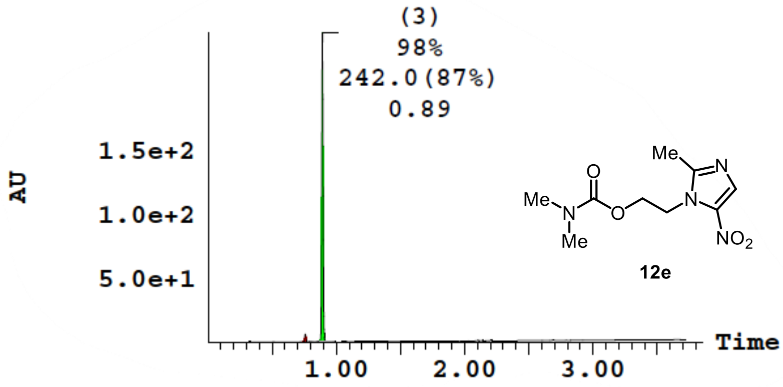

2-(2-Methyl-5-nitro-1*H*-imidazol-1-yl)ethyl isopropyl(methyl)carbamate (12f)

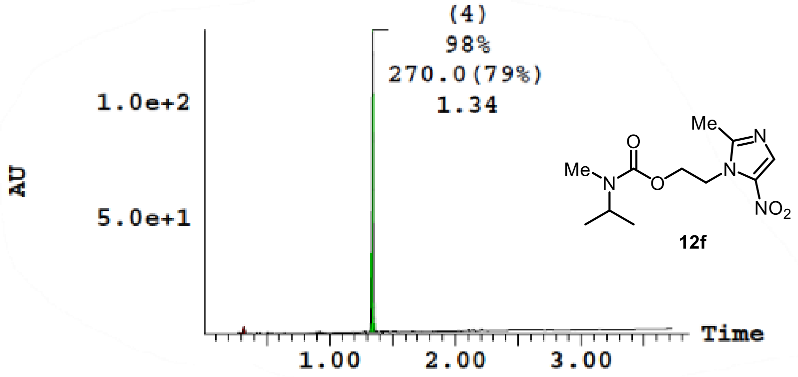

2-(2-Methyl-5-nitro-1*H*-imidazol-1-yl)ethyl cyclopropyl(methyl)carbamate (12g)

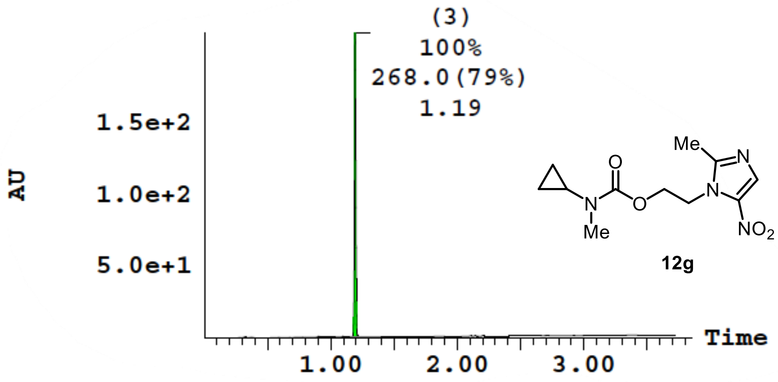

2-(2-Methyl-5-nitro-1*H*-imidazol-1-yl)ethyl cyclopropyl(isopropyl)carbamate (12h)

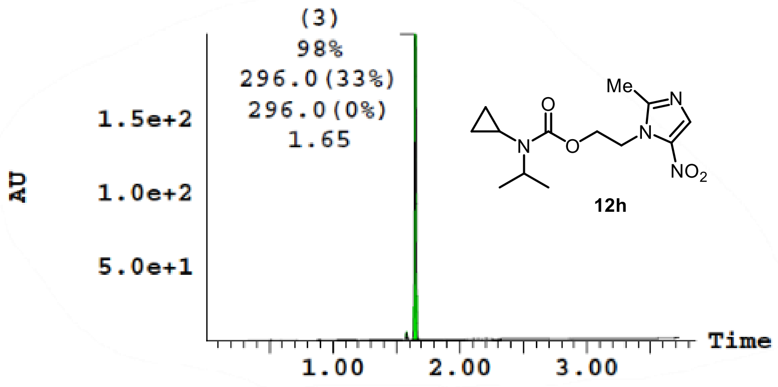

2-(4-(2-(5-Chloro-2-oxobenzo[*d*]thiazol-3(2*H*)-yl)acetyl)piperazin-1-yl)ethyl methyl (trifluoromethyl)carbamate (13a)

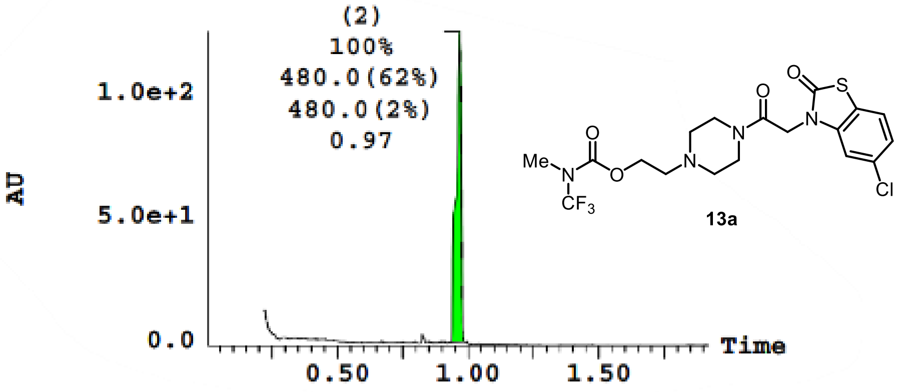

2-(4-(2-(5-Chloro-2-oxobenzo[*d*]thiazol-3(2*H*)-yl)acetyl)piperazin-1-yl)ethyl cyclopropyl (trifluoromethyl)carbamate (13b)

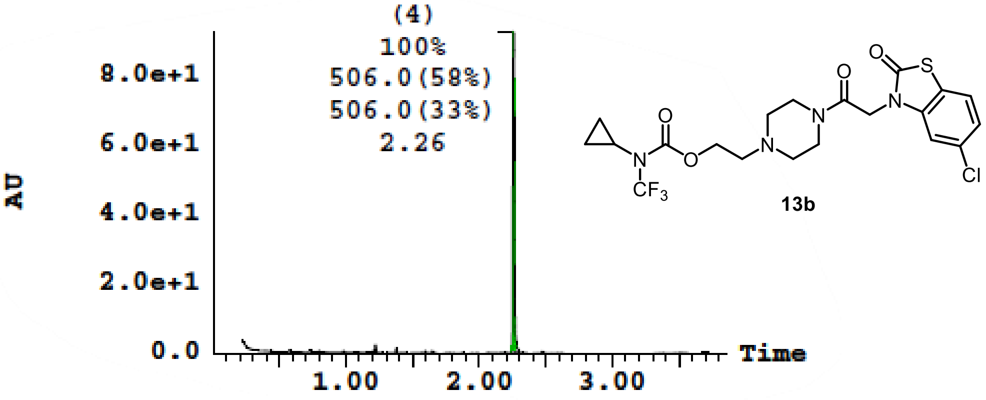

13b

2-(4-(2-(5-Chloro-2-oxobenzo[*d*]thiazol-3(2*H*)-yl)acetyl)piperazin-1-yl)ethyl benzyl (trifluoromethyl)carbamate (13c)

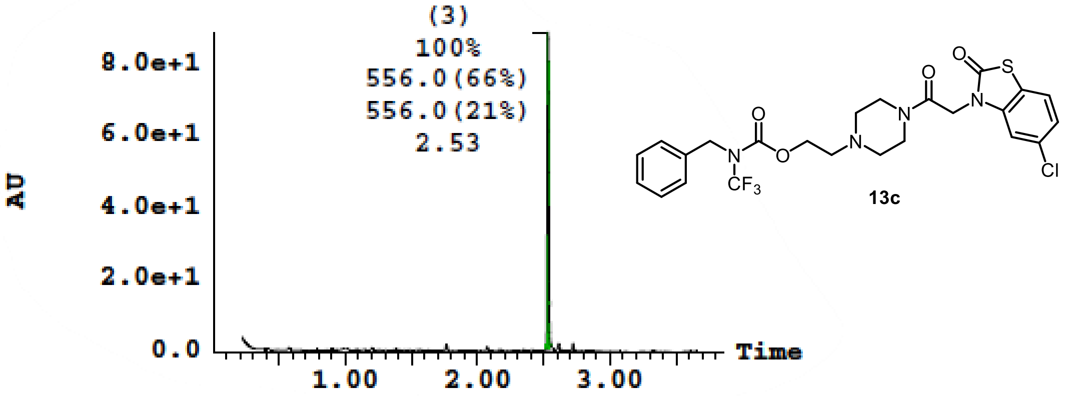

13c

2-(4-(2-(5-Chloro-2-oxobenzo[*d*]thiazol-3(2*H*)-yl)acetyl)piperazin-1-yl)ethyl phenyl (trifluoromethyl)carbamate (13d)

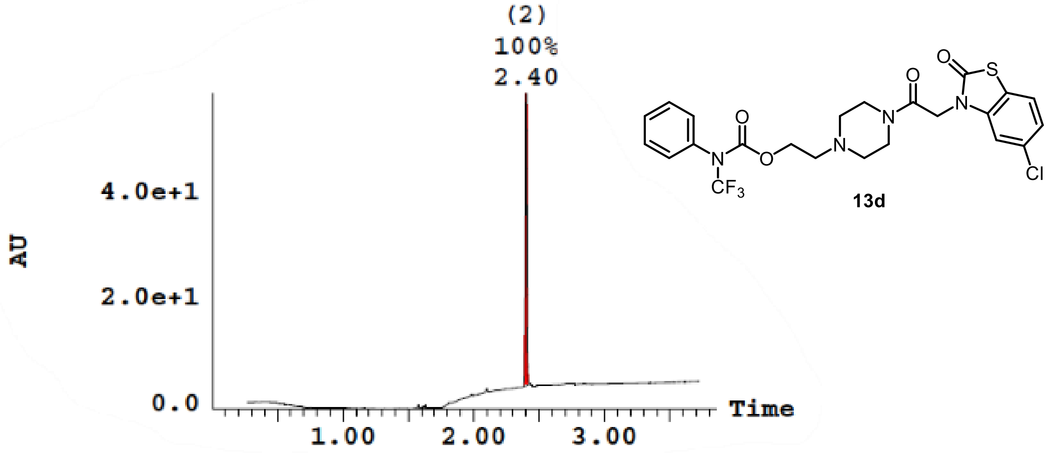

13d

4-Acetamidophenyl methyl(trifluoromethyl)carbamate (14a)

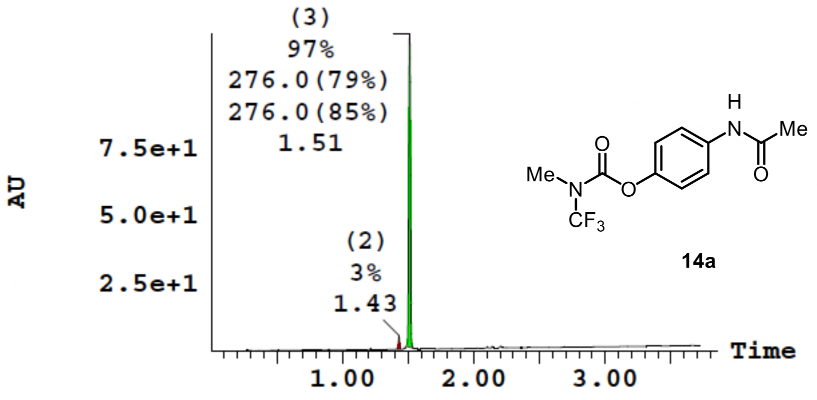

14a

4-Acetamidophenyl cyclopropyl(trifluoromethyl)carbamate (14b)

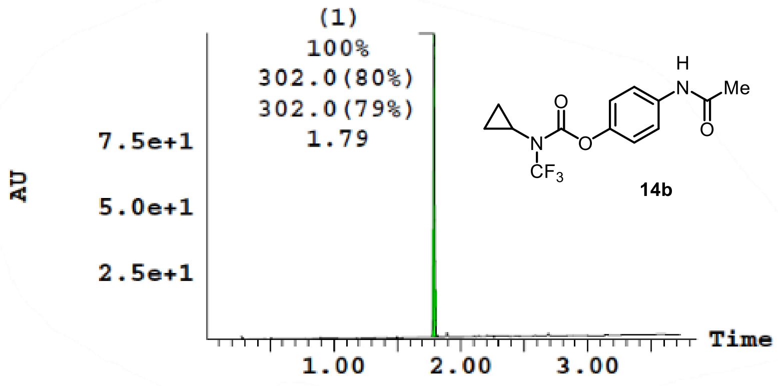

14b

4-Acetamidophenyl benzyl(trifluoromethyl)carbamate (14c)

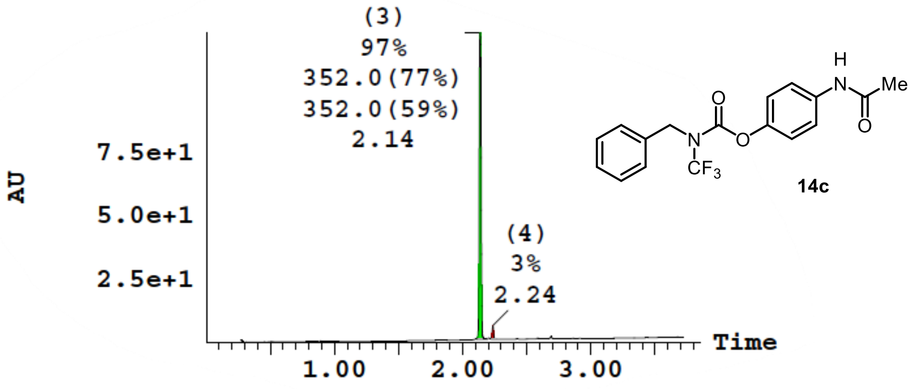

14c

4-Acetamidophenyl phenyl(trifluoromethyl)carbamate (14d)

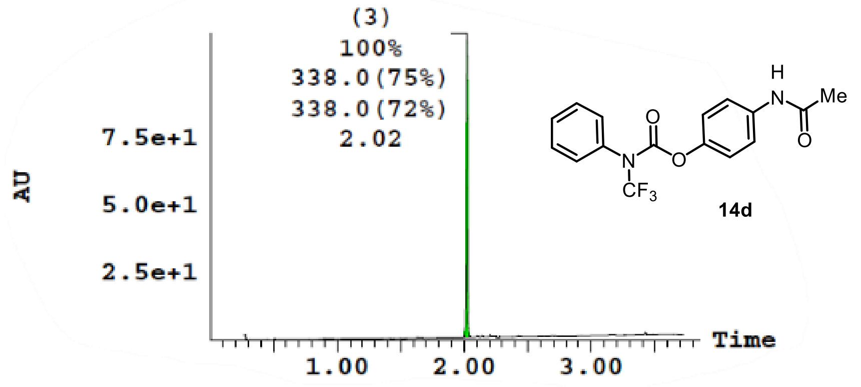

1-Cyclopropyl-6-fluoro-7-(4-(methyl(trifluoromethyl)carbamoyl)piperazin-1-yl)-4-oxo-1,4-dihydroquinoline-3-carboxylic acid (15a)

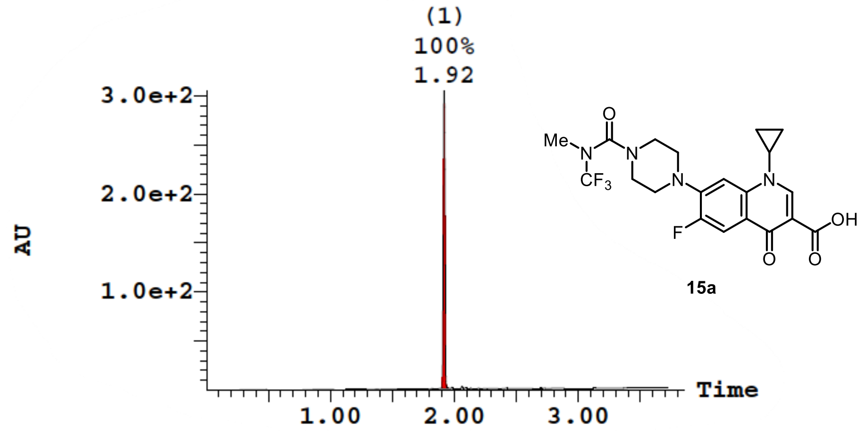

1-Cyclopropyl-7-(4-(cyclopropyl(trifluoromethyl)carbamoyl)piperazin-1-yl)-6-fluoro-4-oxo-1,4-dihydroquinoline-3-carboxylic acid (15b)

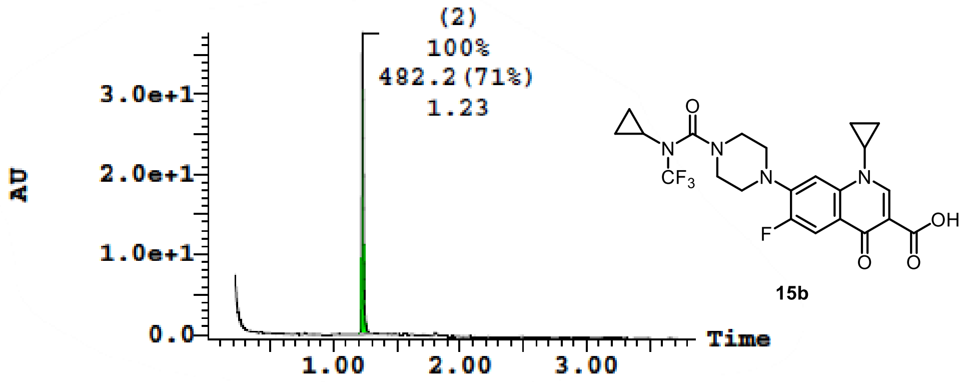

7-(4-(Benzyl(trifluoromethyl)carbamoyl)piperazin-1-yl)-1-cyclopropyl-6-fluoro-4-oxo-1,4-dihydroquinoline-3-carboxylic acid (15c)

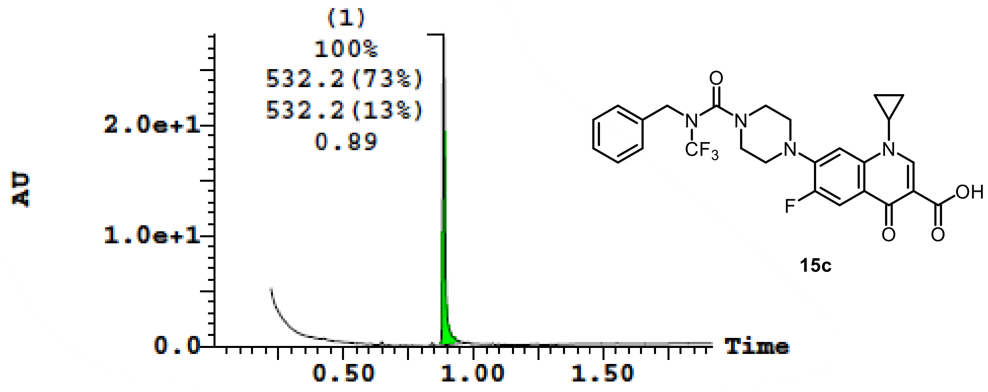

15c

1-Cyclopropyl-6-fluoro-4-oxo-7-(4-(phenyl(trifluoromethyl)carbamoyl)piperazin-1-yl)-1,4-dihydroquinoline-3-carboxylic acid (15d)

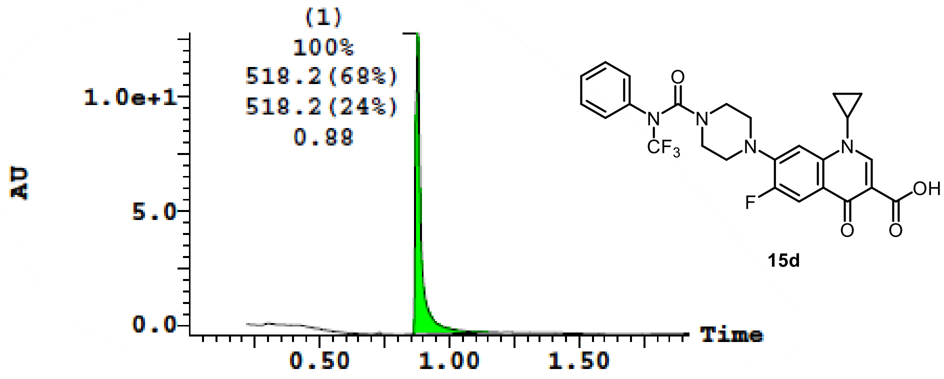

15d

1-Cyclopropyl-7-(4-(dimethylcarbamoyl)piperazin-1-yl)-6-fluoro-4-oxo-1,4-dihydroquinoline-3-carboxylic acid (15e)

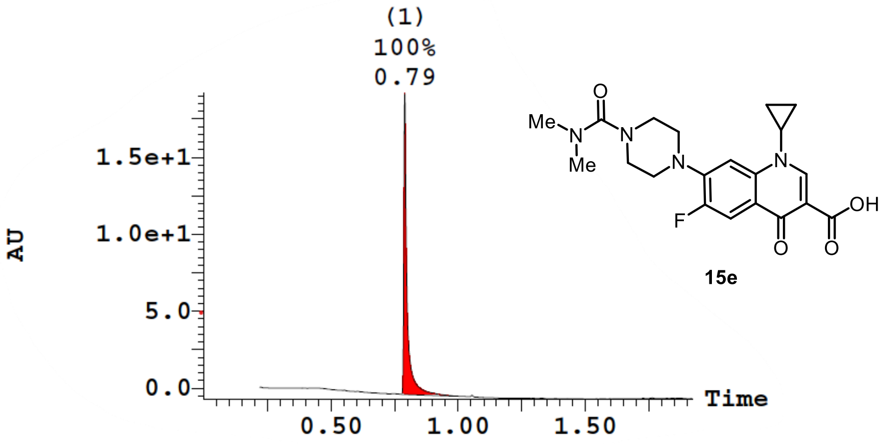

15e

1-Cyclopropyl-6-fluoro-7-(4-(isopropyl(methyl)carbamoyl)piperazin-1-yl)-4-oxo-1,4-dihydroquinoline-3-carboxylic acid (15f)

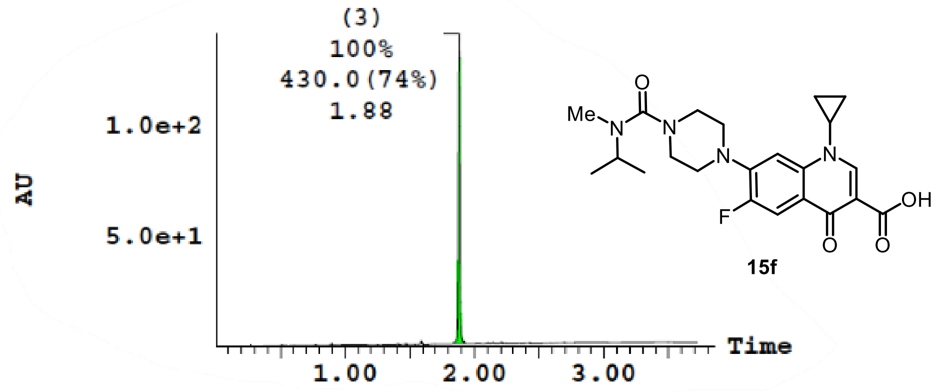

1-Cyclopropyl-7-(4-(cyclopropyl(methyl)carbamoyl)piperazin-1-yl)-6-fluoro-4-oxo-1,4-dihydroquinoline-3-carboxylic acid (15g)

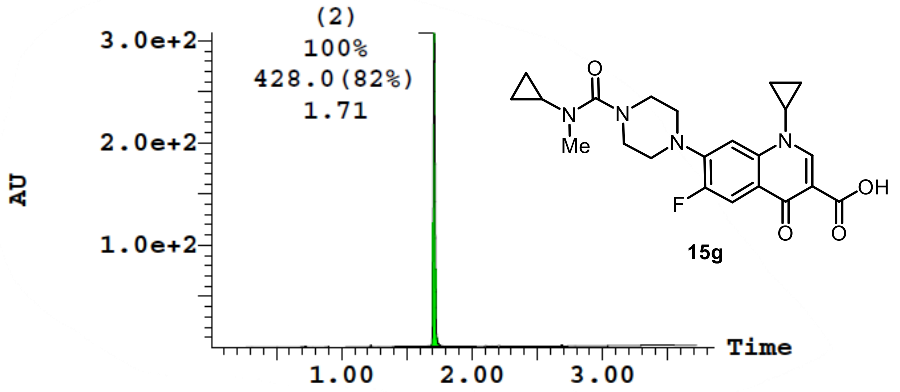

1-Cyclopropyl-7-(4-(cyclopropyl(isopropyl)carbamoyl)piperazin-1-yl)-6-fluoro-4-oxo-1,4-dihydroquinoline-3-carboxylic acid (15h)

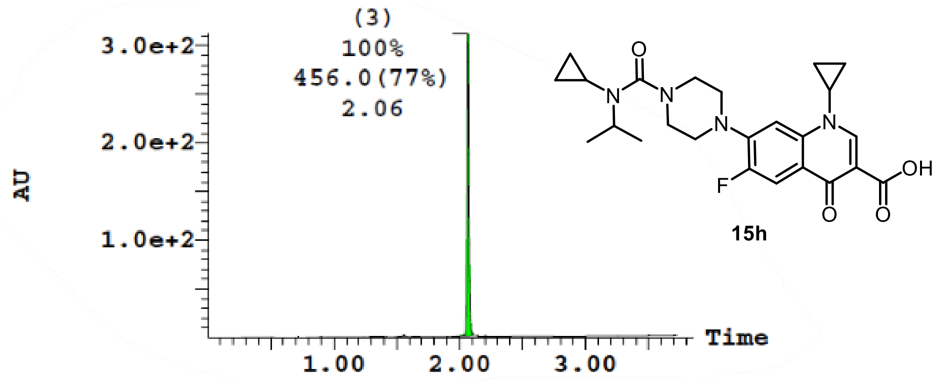

4-((4-Ethoxy-3-(1-methyl-7-oxo-3-propyl-6,7-dihydro-1*H*-pyrazolo[4,3-*d*]pyrimidin-5-yl)phenyl)sulfonyl)-*N*-methyl-*N*-(trifluoromethyl)piperazine-1-carboxamide (16a)

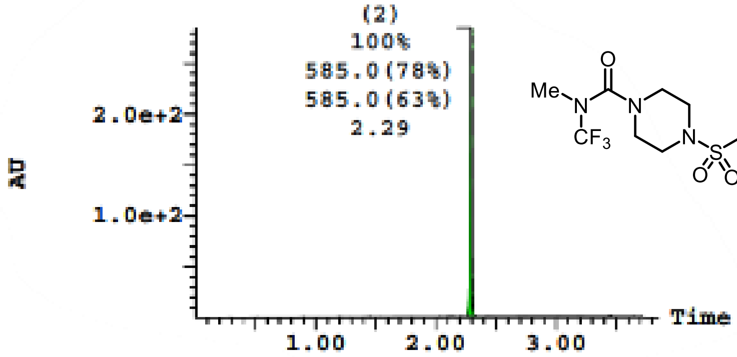

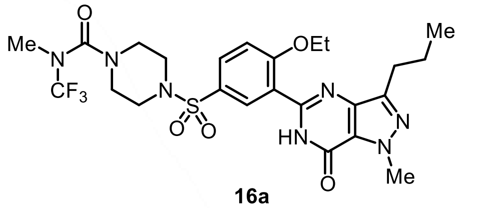

16a

***N*-Cyclopropyl-4-((4-ethoxy-3-(1-methyl-7-oxo-3-propyl-6,7-dihydro-1*H*-pyrazolo[4,3-*d*]pyrimidin-5-yl)phenyl)sulfonyl)-*N*-(trifluoromethyl)piperazine-1-carboxamide (16b)**

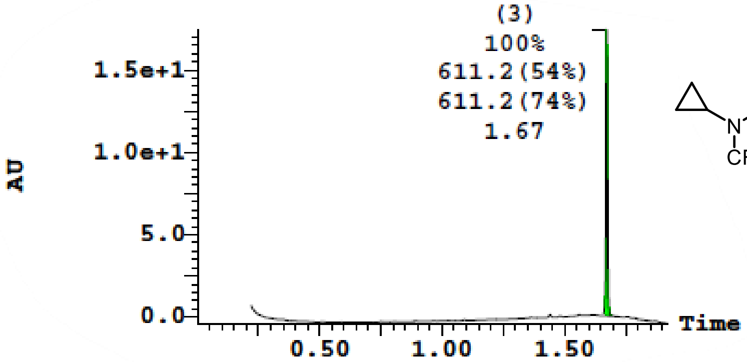

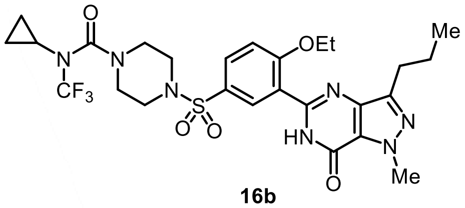

16b

***N*-Benzyl-4-((4-ethoxy-3-(1-methyl-7-oxo-3-propyl-6,7-dihydro-1*H*-pyrazolo[4,3-*d*]pyrimidin-5-yl)phenyl)sulfonyl)-*N*-(trifluoromethyl)piperazine-1-carboxamide (16c)**

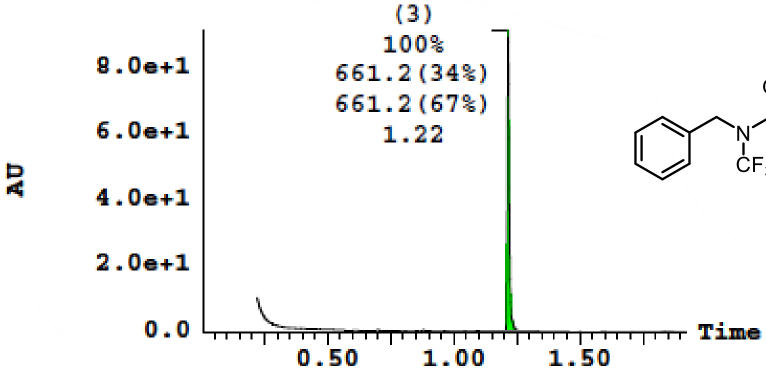

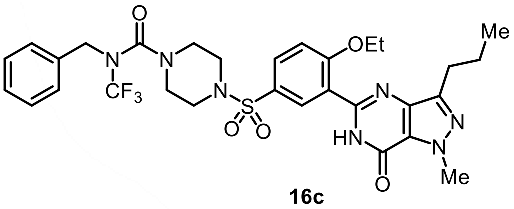

16c

4-((4-Ethoxy-3-(1-methyl-7-oxo-3-propyl-6,7-dihydro-1*H*-pyrazolo[4,3-*d*]pyrimidin-5-yl)phenyl)sulfonyl)-*N*-phenyl-*N*-(trifluoromethyl)piperazine-1-carboxamide (16d)

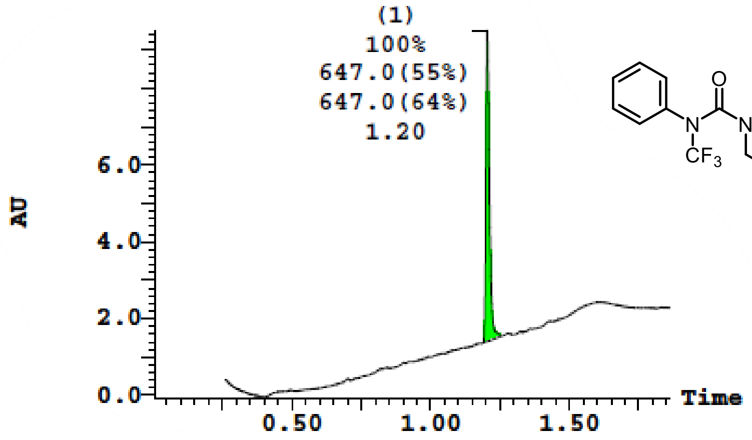

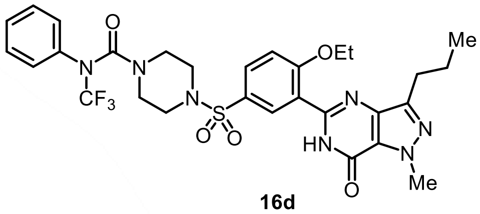

16d

5. References

(1) Scattolin, T.; Bouayad-Gervais, S.; Schoenebeck, F. Straightforward access to *N*-trifluoromethyl amides, carbamates, thiocarbamates and ureas. *Nature* **2019**, *573*, 102 – 107.

(2) Zivkovic, F. G.; Wycich, G.; Liu, L.; Schoenebeck, F. Access to *N*-Difluoromethyl Amides, (Thio)Carbamates, Ureas, and Formamides. *J. Am. Chem. Soc.* **2024**, *146*, 1276 – 1281.

(3) Leung, C.; Liu, J.; Cunico, K.; Johnson, K.; Yan, Z.; Cai, J. An Integrated Hepatocyte Stability Assay for Simultaneous Metabolic Stability Assessment and Metabolite Profiling. *Drug Metab. Dispos.* **2024**, *52*, 377 – 389.

(4) Zamora, I.; Fontaine, F.; Serra, B.; Plasencia, G. High-throughput, computer assisted, specific MetID. A revolution for drug discovery. *Drug Discov. Today Technol.* **2013**, *10*, e199 – e205.
